# Implementation research to scale up the women and infants integrated interventions for growth study (WINGS) in Himachal Pradesh: Protocol for a quasi-experimental, mixed-methods study

**DOI:** 10.1371/journal.pone.0341048

**Published:** 2026-02-17

**Authors:** Arun Singh Jadaun, Jaideep Kumar, Barsha Gadapani Pathak, Sudha Devi, Pradeep Kumar Thakur, Gopal Beri, Mohan Dutt, Seema Thakur, Sanjiv Kumar Verma, Sanjay Mankotia, Anjali Chauhan, Ravinder Kumar, Ankit Chaudhary, Richa Kalia, Devinder Sharma, Ranadip Chowdhury, Neeta Dhabhai, Vinod Kumar Anand, Sunita Taneja, Nita Bhandari, Sarmila Mazumder

**Affiliations:** 1 Society for Applied Studies, New Delhi, India; 2 Centre for Intervention Science in Maternal and Child Health, Centre for International Health, Department of Global Public Health and Primary Care, University of Bergen, Bergen, Norway; 3 Office of Chief Medical Officer Una (HFW), Una, Himachal Pradesh, India; 4 National Health Mission, Shimla, Himachal Pradesh, India; 5 Women and Child Development, Shimla, Himachal Pradesh, India; 6 Regional Hospital Una, Una, Himachal Pradesh, India; PLOS: Public Library of Science, UNITED KINGDOM OF GREAT BRITAIN AND NORTHERN IRELAND

## Abstract

**Background:**

Maternal undernutrition, anaemia, low birthweight (LBW) and childhood stunting remain major public health challenges in India. Evidence from the Women and Infants Integrated Growth Study (WINGS) randomised controlled trial in Delhi showed that an integrated package of health, nutrition, psychosocial and water, sanitation and hygiene (WaSH) interventions delivered from preconception through early childhood substantially reduced LBW and stunting. However, national programmes largely emphasise pregnancy and early childhood, with limited attention to preconception care, and there is little evidence on government-led scale-up models. This protocol describes government-led implementation research to scale up WINGS in Himachal Pradesh.

**Methods:**

We will conduct a 36-month mixed-methods study using implementation science principles and a theory-driven process evaluation. Implementation will begin in a learning block, followed by expansion to remaining blocks. The design is anchored in the Consolidated Framework for Implementation Research (CFIR), RE-AIM (Reach, Effectiveness, Adoption, Implementation, Maintenance) and COM-B (Capability, Opportunity, Motivation → Behaviour). Evaluation will use an Interrupted Time Series (ITS) design. Participants include preconception women, pregnant women, postpartum mothers, children under 24 months, frontline workers (ASHAs, AWWs, ANMs) and facility staff. Interventions span health, nutrition, psychosocial and WaSH domains, integrated within ICDS and National Health Mission platforms. The objective is to develop an optimised model for scale-up that is sustainable, scalable and achieves high-quality population coverage. Outcomes include anaemia, low BMI and inadequate weight gain among women and children, with process indicators assessing compliance. Quantitative data will be analysed using segmented regression in R; qualitative data will undergo framework analysis; and economic evaluation will estimate incremental cost-effectiveness (S4 File: costing forms).

**Discussion:**

This implementation research will generate evidence on the feasibility and scalability of integrating preconception-to-early-childhood interventions within government systems to inform national policy.

**Registration:**

CTRI/2023/10/058538 (Registered on 11/10/2023).

## Introduction

India continues to face a high burden of low birth weight (LBW), small‑for‑gestational age (SGA) and stunting, conditions strongly linked to maternal undernutrition and anaemia. [1] While major national programs (Anemia Mukt Bharat (AMB), Prime Minister’s Overarching Scheme for Holistic Nutrition (POSHAN 2.0), Reproductive, Maternal, Newborn, Child and Adolescent Health (RMNCH+A) have improved coverage of pregnancy and child services, the preconception period remains weakly addressed, despite evidence that maternal nutritional status before conception and in early gestation critically determines fetal growth and neurodevelopment [2–7].

Recent global analyses indicate that nearly one in five Indian children are stunted, and over 20% of newborns are born with LBW, reflecting persistent intergenerational cycles of undernutrition and inadequate maternal care. [8] Evidence from large cohort and intervention studies has underscored that interventions initiated only during pregnancy are often too late to reverse growth faltering, whereas those starting before conception yield greater benefits for birth outcomes and child growth. [2, 9, 10] Addressing this preconception window therefore represents a key opportunity for accelerating progress towards the Sustainable Development Goals (SDG 2 and SDG 3) on nutrition and health. [11] The Women and Infants Integrated Growth Study *(*WINGS) randomized controlled trial conducted in Delhi demonstrated that an integrated package spanning preconception, pregnancy and early childhood-covering health, nutrition, psychosocial and WaSH) domains, reduced LBW and stunting at 24 months. [12] These results underscore the value of multi‑domain, longitudinal support rather than single‑touchpoint interventions during pregnancy or childhood alone [13–15].

However, there is limited evidence on how such a package can be delivered at scale by government systems, especially in geographies with difficult terrain and multisectoral coordination needs. Himachal Pradesh presents an opportunity to co‑develop and test a government‑led model with strong state ownership and a commitment to preconception care under National Institution for Transforming India (NITI)Aayog’s broader equity agenda [16,17].

This protocol describes a theory‑driven, government‑led scale‑up of WINGS in Una district of Himachal Pradesh. We aim to optimise delivery strategies through co‑design, evaluate effects using an interrupted time series design, and generate a replicable blueprint for scale‑up.

### Research question

How can an integrated intervention model addressing maternal and child health, nutrition, psychosocial well-being, early child development and WaSH, successfully tested in the WINGS trial, be scaled up and sustainably implemented through existing government health and nutrition systems in Himachal Pradesh?

### Objectives

The aim is to develop a framework and action plan to guide government partners in scaling up WINGS integrated interventions in Una district, Himachal Pradesh. The model will be government-led, delivered through Integrated childhood development scheme/National Health Mission (ICDS/NHM) platforms, co-designed for sustainability, scalability, and high-quality population coverage.

The primary outcomes of this study are focused on strengthening health systems and streamlining processes by identifying existing gaps and implementing innovative, sustainable solutions to improve maternal and child health, alongside enhancing commitment and mobilizing resources through evidence-informed advocacy. A secondary outcome is to estimate the incremental cost of implementing WINGS interventions within government health systems.

## Materials and methods

### Study design and rationale

**Fig 1:**
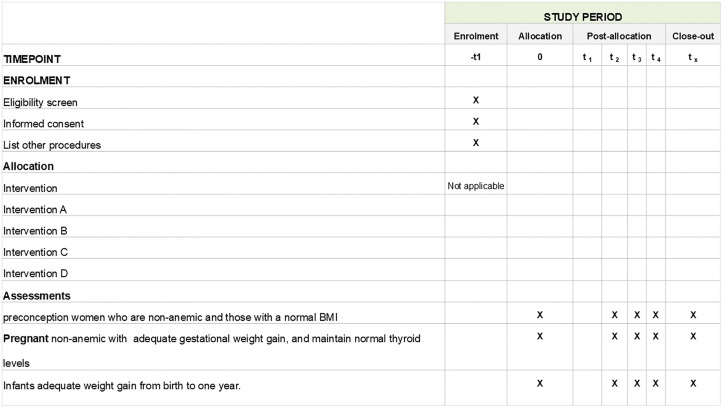
SPIRIT checklist for WINGS implementation research.

We will use a convergent mixed‑methods design using principles of implementation science. [18] Implementation will occur in the ‘learning block’ first, with iterative optimization over two to three adaptation cycles, followed by simultaneous expansion to the remaining blocks. A theory‑driven process evaluation will run in parallel to explain the observed effects and inform ongoing adaptation. Additionally, an interrupted time-series (ITS) design will be applied across all study blocks to evaluate temporal changes in outcomes before and after implementation. [19] This quasi-experimental approach is particularly suitable for evaluating large-scale policy and programme interventions where randomization is not feasible and allows separation of underlying trends from those associated with the intervention. [20] Fig 1 presents the SPIRIT checklist for this research and detailed information in Supporting information (SI File)-SPIRIT Checklist.

#### Implementation science framework.

This study will adopt a dynamic, context-responsive implementation approach that emphasizes continuous stakeholder engagement, systematic assessment of contextual enablers and barriers, and responsive resource mobilization. The implementation process will be guided by the RE-AIM framework to evaluate reach, adoption, fidelity, quality, and maintenance, while contextual determinants will be explored using the Consolidated Framework for Implementation Research (CFIR) and behavioural drivers interpreted through the Capability, Opportunity, Motivation–Behaviour (COM-B) model [21–23].

#### Operational definitions.

- Pre-conception women: A married woman aged18–35 years, who has not completed her family and is not currently pregnant.
- Pregnant woman: A woman with confirmed pregnancy residing in the study area.
- Child: A child aged 0–24 months, born to a resident woman.
- Under-nourished woman: Woman with body mass index (BMI) <18.5 kg/m² as per the WHO BMI definition.
- Inadequate weight gain (IWG): A preconception woman is categorized under IWG when she gains less than 500gms per month in women with low BMI.
- Inadequate weight gain in pregnancy: This at each trimester of pregnancy according to the BMI categories as defined by the Institute of Medicine Guidelines [Pregnancy weight gain of 12.5 to 18kgs in women with BMI < 18.5 kg/m2; 11.5 to 16kgs in women with BMI of 18.5 to 24.9 kg/m2; 7 to 11.5kgs in women with BMI of 25.0 to 29.9 kg/m2 and weight gain of 5–9kgs in women with BMI ≥ 30.0 kg/m2 [12].

### Study setting

The study will be conducted in Una district, located in the northern Indian state of Himachal Pradesh, identified as the pilot site for the Women and Infants Integrated Growth Study (WINGS) scale-up initiative. Una was purposively selected owing to its diverse topography comprising both plains and hilly terrain, moderate performance on key maternal and child health indicators, and strong state commitment to preconception and maternal health. The district also represents a government-priority aspirational district, with functional ICDS and NHM platforms actively implementing national programmes on maternal, newborn, and child health.

Administratively, Una comprises five development blocks, i.e., Una (Basdehra), Amb, Gagret, Dhundla (Thanakalan), and Haroli, covering an area of approximately 1549 square kilometres with an estimated population of 521,173 (Census 2011). [24] The district includes 1364 Anganwadi Centres, 138 Health and Wellness centres/Sub-centres, 24 Primary Health Centres (PHCs), 9 Community Health Centres (CHCs), 5 Civil Hospitals, and one Regional Hospital, collectively forming the public health infrastructure that will serve as the backbone for the WINGS scale-up [24].

The implementation will be carried out through existing government service delivery channels, primarily ICDS Anganwadi Centres, Health and Wellness Centres (HWCs), and PHCs, under the NHM. These facilities will provide preconception, antenatal, and early childhood services, supported by frontline health workers including Accredited Social Health Activists (ASHAs), Auxiliary Nurse Midwives (ANMs), Community Health Officers (CHOs), and Anganwadi Workers (AWWs). The study will be jointly implemented by the Government of Himachal Pradesh, through the Departments of Health and Family Welfare and Women and Child Development, with technical support from the Society for Applied Studies (SAS).

### Intervention package

The interventions are in four domains during the preconception, pregnancy, and early childhood (0–24 months) periods. The detail interventions and strategies are described in S1 Table.

### Outcomes

#### Outcome indicators.

Specific outcome indicators include the proportion of preconception women (married, reproductive age, not completed families) who are non-anemic and those with a normal BMI (18.5–24.99 kg/m²). For pregnant women, outcomes include proportions who are non-anemic, achieve adequate gestational weight gain, and maintain normal thyroid levels. For infants, outcomes focus on adequate weight gain from birth to one year. The outcome indicators will be assessed through quarterly monitoring during implementation and three rounds, each of pre and post-intervention surveys.

### Sample size and sampling

Data on key outcomes will be aggregated at block level across five blocks, with measurements at three pre-intervention and three post-intervention time points.(Table 1) The study is powered to detect a 20% change in outcomes with 88.3% power (95% CI: 86.3–90.3), estimated using the *itspower* package in Stata 17. Outcomes of interest include adequate gestational weight gain (IOM standards), non-anemia (Hb ≥ 11 g/dl), normal BMI (18.5–25 kg/m²), and adequate child weight gain (WHO standards). Based on NFHS-5 and WINGS baseline data, expected improvements are from 55% to 66% for adequate GWG and normal BMI, 45% to 54% for non-anemia, and 60% to 72% for child weight gain. A sample size of 600 women and children per survey round will be adequate, with 400 as the minimum feasible requirement.

**Table 1:** Summarizes primary, secondary and process outcomes with definitions, data sources and measurement frequency.

Outcome	Indicator/Definition	Population	Data source	Timing
Anaemia among women	Hb < 11.0 g/dL (pregnancy)/ < 12.0 g/dL (non‑pregnant)	Women preconception & pregnant	Household surveys; facility labs	Quarterly subsample surveys
Maternal BMI	BMI (kg/m²) categories; underweight <18.5	Preconception women	Household surveys	Quarterly subsample surveys
Adequate GWG	GWG by IOM targets (1^st^ trimester/pre‑pregnancy BMI)	Pregnant women	ANC register; cohort subsample	During pregnancy
Infant growth	Birthweight; LAZ at 12 months	Infants 0–12 m	Facility records; household surveys	Quarterly subsample surveys
Maternal depressive symptoms	PHQ: ≥ 3	Preconception pregnant & postnatal women	Household surveys	Quarterly subsample surveys
Exclusive breastfeeding	EBF till 6 months	0– < 6 m (At 5 m;150–170 days)	Household surveys	Quarterly subsample surveys
Process – Reach	Preconception, pregnant, postnatal women, infants 0–12 months	Women and infants	Routine data from facility/ community; surveys	Monthly/quarterly
Process – Fidelity	Compliance to interventions delivery and intake across domains	Facilities/CHWs/AWWs	Registers & records, surveys, interviews.	Quarterly
Maintenance	Inclusion in PIP; routine review frequency	Health and ICDS System	Govt documents	Endline/post

Households for surveys will be randomly selected to identify eligible women/infants, from the line listings of all households at village level. Villages selected proportional to size in each block.

**Fig 2:**
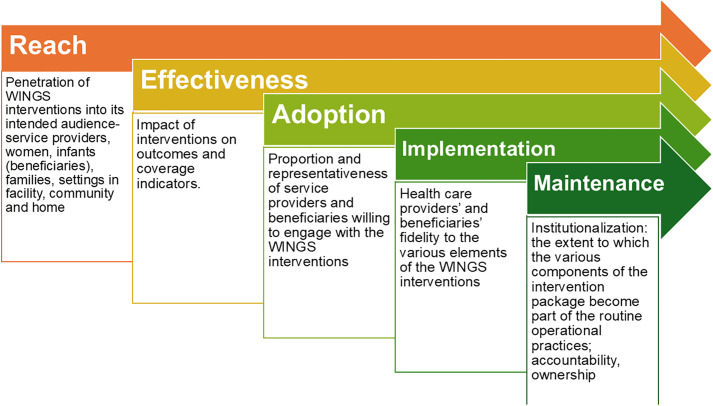
RE-AIM Figure. Figure adapted from RE-AIM – Home – Reach Effectiveness Adoption Implementation Maintenance [25].

Coverage and process indicators will track, among others: consumption of IFA/MMS, nutritional supplementation for undernourished women, screening and management of health conditions (hypertension, RTI, diabetes, hypothyroidism), early ANC registration, recommended ANC visits, identification of high-risk pregnancies, and adherence to IFA, albendazole, and MMS protocols. Postnatal indicators include mothers receiving nutritional supplements and infants completing at least six HBNC visits. Infant care outcomes cover exclusive breastfeeding, kangaroo mother care, and receiving IFA. Cross-cutting measures will assess SBCC activities and exposure, psychosocial support, and WaSH interventions received by eligible women and infants. Fig 2 illustrates the implementation research framework for capturing the outcomes.

### Project implementation process

The WINGS interventions will be integrated within existing government programs, with additional preconception interventions adopted through state-level policy decisions. Implementation in Una district will be supported by SAS, using the CFIR framework to guide iterative cycles of implementation, monitoring, learning, and adaptation. This will include stakeholder engagement, process learning through quantitative and qualitative feedback, and government-led review meetings to optimize strategies. Monitoring will apply RE-AIM (reach, effectiveness, adoption, implementation, maintenance) and COM-B to address behavior change, informing IEC materials and counselling guides.(Fig 2) An interrupted time-series design will be used across all five blocks to assess outcomes, with three monthly block‑level outcome measurement.

#### Research teams.

Implementation will be supported by three coordinated research teams, each with complementary roles to ensure rigour and responsiveness throughout the study. The Implementation Support Team (IST) will provide intensive technical and operational assistance to government staff during the initial phase, helping to establish processes, build capacity, and troubleshoot field-level challenges. As the intervention stabilises, the IST will transition to an advisory role, supporting the government in scaling up and maintaining programme fidelity. The Programme Learning Team (PLT) will apply qualitative and participatory methods to assess implementation fidelity, undertake root-cause analyses to identify barriers and facilitators, and generate insights for real-time corrective actions.(S3 File: Qualitative forms) The Outcome Monitoring Team (OMT) will be responsible for tracking coverage and predefined outcome indicators through quarterly monitoring and three survey rounds each at pre- and post-intervention stages, enabling the assessment of both implementation performance and population-level changes over time.

### Study phases

Although the study is planned to be conducted in a phased manner, a considerable overlap and concurrent activities are anticipated. The initial preparatory phase (formative research), lasting approximately six months, will focus on conducting situational analyses and system level diagnosis to identify barriers and facilitators and assess contextual readiness within existing government systems. The model optimization will be conducted in the learning block, which will be concurrently scaled up in the remaining blocks with continuous monitoring, adaptive learning cycles, and iterative refinements based on implementation feedback as per contextual needs. spanning two years. Data analysis, synthesis, will be an ongoing process. Final report preparation and dissemination, in the final six months, will focus on analyzing and compiling the overall quantitative and qualitative data across all blocks, consolidating lessons learned, and generating evidence to inform state and national policy translation for scale-up of the WINGS model.

#### Formative phase.

The formative phase will be led by the PLT in the first implementation block using mixed methods to conduct a system-level diagnosis and identify barriers and facilitators to implementing the WINGS intervention. Quantitative assessments will include facility and community surveys, while qualitative components will comprise in-depth interviews, focus group discussions and structured observations. (S6 File: Facility assessment and Household survey forms) The formative research will explore key domains such as population profile and potential beneficiaries, health and ICDS infrastructure and human resources, service availability and utilization across the four WINGS domains (health, nutrition, psychosocial care and WaSH), referral mechanisms and transport availability, coverage and quality of relevant national programs, digital platforms and data systems, SBCC and community engagement platforms, awareness and utilization of government schemes and the role of local non-governmental organizations in maternal and child health. In parallel, the OMT will conduct baseline surveys using an interrupted time-series (ITS) design, with three survey rounds planned to capture key outcome indicators. The overall duration of the formative phase, including baseline data collection, will be approximately nine months.

#### Model optimization phase.

Implementation will begin with a base model (“Model 0”) integrating existing pregnancy and childhood interventions, plus additional WINGS components. Model 0 will evolve into Model 1 by incorporating formative research, followed by rapid cycles of implementation, monitoring, and refinement through co-design workshops with government partners. High coverage, defined with government as at least a 20% improvement over baseline, will serve as the benchmark for success. Considering the complexity of the multiple interventions and domains, the target coverage of each outcome may not be achieved concurrently at the same point. This may happen in a phased manner.

**Fig 3:**
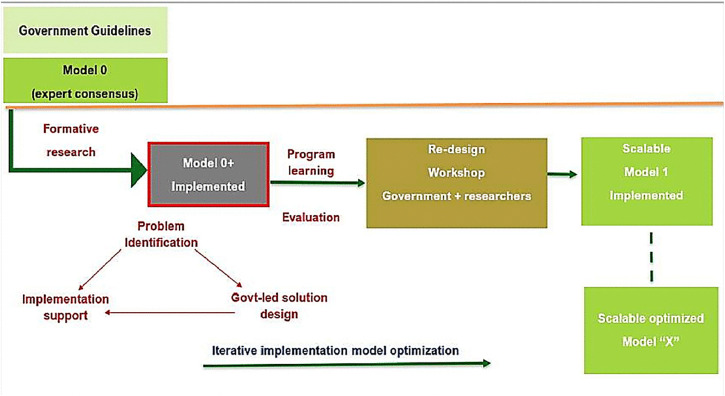
Model optimization using iterative cycles. Figure adapted from World Health Organization and re-used from published paper [26]*.*

Model optimization in the first block (~9 months) will involve 2–3 iterative cycles. Subsequent blocks will require ~6 months each, with contextual adaptations as needed. All government facilities and frontline workers (district hospital, CHCs, PHCs, HWCs, SCs, medical college hospital, and ICDS/AWCs) will be engaged. The full project, including preparatory work, optimization, rollout across five blocks, outcome monitoring, reporting, and dissemination, is expected to be completed in ~36 months. Fig 3 illustrates the process of model optimization.

### Implementation strategies

Implementation strategies are mapped to the Expert Recommendation for Implementing Change (ERIC) taxonomy and grouped under capacity‑building, systems strengthening and behavior change:

- Educational meetings and dynamic training for ICDS workers, CHWs and facility staff; refresher micro‑modules; on‑the‑job coaching, use of job aids.
- Continuous evaluation and feedback via monthly review meetings at block and facility levels.
- Practice facilitation and supportive supervision visits using structured checklists.
- Develop and support clinical champions (obstetric and pediatric leads) and opinion leaders (senior AWWs/ASHAs).
- Tailor strategies to context using COM‑B diagnostics (capability gaps → training; opportunity constraints → space/supplies/rosters; motivation gaps → recognition, peer norms).
- Strengthen inter‑organizational networks through joint Health–ICDS reviews and integrated micro‑plans.
- Revise roles/workflows to enable preconception enrolment, ANC early registration <12 weeks, and proactive postnatal care (PNC) and Early Childhood Development (ECD) counselling.
- Enhance information systems (harmonized registers, digital tally sheets) and introduce reminder/recall for follow‑up using an Application that will be designed for the scale up.
- Formalise policies through government orders to the extent possible, to embed the model and sustain maintenance (RE‑AIM ‘M’).

We will use CFIR to explain contextual determinants; RE‑AIM to structure implementation evaluation; and COM‑B to diagnose and address behaviour for key actors [23, 25, 27].

### Study participants and capacity building

The study will include preconception women, pregnant women across all trimesters, and infants at different ages, entering the intervention phase as roll-out progresses across blocks. Cross-sectional data will be captured through quarterly surveys, while longitudinal follow-up of women (from preconception through pregnancy) and their infants will be conducted by government personnel (ASHAs, AWWs, and facility staff), thereby strengthening routine follow up and data systems using a cloud-based server [28]. Research teams will review these data jointly with government counterparts.

Simultaneously, capacity building of local government partners will ensure sustainability beyond the research phase. One or two local technical institutions, if identified by the government, will also be engaged to observe, learn, and subsequently support implementation in other districts.

#### Co‑design workshops and adaptive learning cycles.

Co‑design is central to this protocol. Each quarter, the PLT will convene structured workshops with frontline workers (ASHAs, AWWs, ANMs), facility staff, block/district managers and state officials. Using human‑centred design tools (journey mapping of women/infant dyads, service blueprinting, and ‘bright spots’ identification), the group will review monitoring data and qualitative insights, prioritise bottlenecks, and agree time‑bound adaptations. Adaptations will be classified (content, context, intensity, or delivery modifications) and fed back to the Implementation units constituted as a part of governance for authorisation. Each adaptation constitutes a ‘mini‑cycle’ within the iterative processes, enabling explicit linkage between changes in implementation and outcome trends.

#### Convergence between line departments and development partners.

WINGS will be implemented through close collaboration between government line departments and development partners to deliver a comprehensive package of health and nutrition interventions covering the preconception period, pregnancy, and the first 24 months of life. This integrated approach aims to maximize synergies across sectors and ensure better outcomes for mothers and children. Convergence will be operationalized through existing platforms at the state, district, and block levels, supported by dedicated implementation units.

#### State-level support units.

At the state level, a Technical Support Unit (TSU) chaired by the Secretary of Health will bring together representatives from key government departments, medical institutions, development partners (WHO, BIRAC, BMGF), SAS, and civil society. Meeting twice a year, the TSU will function as the nodal technical resource centre, strengthen monitoring systems, promote advocacy, and share best practices. A parallel State Implementation Unit, also chaired by the Secretary of Health, will meet quarterly to oversee administration, coordinate key performance indicators, and implement corrective actions suggested by the TSU.

#### District and block-level units.

At the district level, the District Implementation Unit, chaired by the Deputy Commissioner, will meet monthly to assess needs across sectors such as water, sanitation, food, health, and immunization. It will plan rollouts, allocate responsibilities, and ensure resource availability. At the block level, the Block Implementation Support Unit, chaired by the Sub-Divisional Magistrate, will meet twice a month to assess village-level needs, coordinate essential interventions, and assign departmental responsibilities to avoid overlaps.

#### Project monitoring committee (PMC).

In addition, a PMC convened by BIRAC with experts from NITI Aayog, ICMR, and other institutions will periodically review progress with the Himachal Pradesh government and research teams, providing high-level guidance and oversight.

### Data collection, management and analysis

#### Study status.

The study is currently ongoing and advancing through its planned phases. The preparatory phase activities by the government stakeholders are underway. The formative research) is ongoing, alongside baseline quantitative surveys in the learning block. Currently, the participant recruitment has not yet started, and this will be completed in October 2027, data collection will be completed October 2027 and results are expected by end of year 2027 or beginning of 2028.

### Ethics and dissemination

This study will be conducted in accordance with the principles of Good Clinical Practice (GCP) and the ethical standards for implementation research. Ethical approval has been obtained from the Ethics Review Committee of the Society for Applied Studies (SAS/ERC/WINGS Scale up-HP/2023). The Biotechnology Industry Research Assistance Council (BIRAC) (GCI-13012/3/2024-GCI) will provide national oversight for this project. Necessary administrative approvals have also been secured from the Government of Himachal Pradesh through the Departments of Health and Family Welfare and Women and Child Development, of Himachal Pradesh state (Memo no Shimla-2/1June/2023/8483).

Before enrolment, all participants, including pre-conception and pregnant women, mothers or primary caregivers of infants and young children, and healthcare providers, will be provided with written informed-consent forms in the local language (Hindi). For participants who are non-literate, the consent form will be read aloud in the presence of an impartial witness, and consent will be documented using a thumb impression. Participation will be entirely voluntary, and individuals may withdraw at any stage without consequence.

This protocol follows relevant reporting guidelines of STROBE (attached as a S2 File: STROBE annexure) for reporting and while implementation the Standards for Reporting Implementation Studies (StaRI) statement [28, 29].

High priority will be placed on participant confidentiality and data security. Data custody will remain with the Data Management Centre at the field site, supervised by the SAS data coordinator. All data-collection devices will be password-protected, and real-time data will be securely transmitted via [30] SurveyCTO to a cloud-based server accessible only to authorised research personnel. Identifiable personal information (such as names, addresses, or contact numbers) will be stored separately from analytical datasets in encrypted, access-restricted folders. De-identified datasets may be shared with BIRAC and collaborating institutions for secondary analyses after appropriate data-sharing agreements and approvals.

Prior to finalising this protocol, an extensive stakeholder engagement process was conducted in Himachal Pradesh, involving state and district officials, and frontline health workers (ASHAs, ANMs, AWWs). Insights from this engagement informed the design, and adaptation of data-collection tools to ensure cultural appropriateness and operational feasibility.

*Dissemination*: Open‑access publications; presentations at national/global conferences; quarterly/annual government review meetings; state and national policy briefs; and a publicly accessible synopsis of lessons and tools.

## Discussion

This study highlights the critical importance of intervening across the continuum of preconception, pregnancy, and early childhood to address maternal undernutrition, adverse birth outcomes, and child stunting. Adequate growth and development in the first 1,000 days of life lay the foundation for long-term health, human capital, and economic productivity, and are central to achieving the Sustainable Development Goals (SDGs).(1, 11)The WINGS model extends this continuum to addresses “1000 days plus” by including the preconception period, a stage increasingly recognized as pivotal for improving pregnancy and child outcomes. [3, 31] Consistent with the 2013 and 2021 *Lancet* Nutrition Series, our findings reaffirm that maternal nutritional status is a key determinant of fetal growth restriction, LBW) and SGA births, which in turn increase risks of stunting, morbidity, and mortality [10, 32].

The WINGS randomized controlled trial provides robust evidence that integrated interventions beginning before conception, sustained through pregnancy and early childhood, substantially reduce LBW and stunting at 24 months.(12)Notably, interventions initiated only during pregnancy or early childhood produced smaller, though still important, effects, while preconception-only interventions improved birth outcomes but did not influence child stunting. [9, 13, 33] These findings underscore the value of integrated, multi-domain strategies that start early, continue across life stages, and combine health, nutrition, psychosocial, and WASH interventions. This life-course approach aligns with global recommendations for nurturing care, which emphasize combining biomedical, behavioural, and environmental actions to achieve sustainable impact. [34, 35]

From an implementation perspective, the WINGS scale-up initiative operationalizes these principles within existing government systems, addressing both intervention delivery and the implementation strategies required for scale and sustainability. Guided by robust implementation frameworks like CFIR, RE-AIM, and COM-B, the study applies a structured process of adaptation, co-design, and fidelity monitoring, approaches increasingly endorsed in large-scale implementation studies. [23, 27, 36] The use of a quasi-experimental ITS design further allows real-time evaluation of system-level effects where randomization is not feasible [37]. Anticipated challenges include ensuring multi‑sectoral convergence; staff turnover and competing priorities; supply‑chain instability; data quality; and geographic barriers. These challenges mirror those reported in other implementation research initiatives across India and low- and middle-income countries (LMICs), underscoring the need for adaptive learning systems and distributed leadership. [22, 38, 39]. Planned mitigation includes government‑led governance, formal administrative orders, integrated micro‑plans, continuous audit‑and‑feedback, and documentation of responsive adaptations. [40, 41] Limitations include one‑district scope and a finite implementation window; nonetheless, the learning block and subsequent roll‑out increase generalizability, and the blueprint will facilitate replication across similar settings.

Our proposed scale-up in Una district translates this evidence into practice by embedding WINGS interventions within government health and nutrition systems. The model emphasizes co-design with state partners, iterative optimization, and convergence across multiple departments, reflecting real-world implementation challenges and opportunities. Lessons from this process will inform how best to adapt and institutionalize such models in diverse contexts across India and other LMICs, contributing to both the implementation-science evidence base and policy translation. The complete proposal can be assessed in S7 File: Detailed WINGS proposal.

### Policy implications

1. **Prioritizing Preconception Care**

Evidence from WINGS shows that interventions before pregnancy improve fetal growth and birth outcomes. National programs such as Anemia Mukt Bharat, Mission Parivar Vikas, and Poshan Abhiyaan should integrate targeted preconception care, including screening, micronutrient supplementation, and weight management, into routine health services, with ASHAs and ANMs leveraging existing eligible couple registers to identify beneficiaries.

1. **Strengthening Multisectoral Convergence**

Stunting prevention requires coordinated inputs across health, WCD, and Jal Shakti departments. Mechanisms such as state and district implementation units, project monitoring committee, and local coordination groups can institutionalize convergence. Alignment with existing platforms (VHSNDs, Poshan Tracker, HMIS) will reduce duplication and maximize resource use.

1. **Enhancing Health System Readiness**

Scaling integrated interventions will require investments in infrastructure, human resources, training, supply chains, and supervisory systems. Strengthening HMIS to incorporate WINGS indicators, ensuring interoperability across digital platforms (RCH, ICDS-CAS, Poshan Tracker), and leveraging ABHA IDs can enable longitudinal tracking and accountability.

1. **Sustainability through Institutionalization**

Integration of WINGS indicators into HMIS and routine government review systems is essential for sustainability. Documentation of processes, SOPs, job aids, and manuals, alongside capacity-building of frontline workers, will facilitate adoption. Establishing technical support units with local medical colleges and ICMR networks can provide sustained technical assistance beyond the project period.

1. **Financing and Resource Mobilization**

Implementation feasibility depends on adequate financing. While many interventions are already included in national guidelines, effective delivery will require mobilizing funds through state PIPs, flexi-pools, PPPs, and leveraging untied funds. Donor and development partner support may be essential during transition phases, but long-term sustainability rests on integration into government budgets.

1. **Building Resilience Against Political and Administrative Change**

Sustained political will is critical. Formal agreements such as MoUs with state governments, engagement of NITI Aayog and ICMR, and involvement of high-level review committees can safeguard continuity in the face of leadership changes.

## Conclusion

The prevention of stunting demands early, integrated, and sustained interventions that go beyond the traditional 1,000-day window and begin before conception. The WINGS model demonstrates both efficacy and feasibility of such an approach when embedded within government systems with strong multisectoral convergence. Scaling this model in Una district will generate implementation evidence for state and national scale-up, with significant potential to accelerate progress toward India’s SDG targets on maternal and child health, nutrition, and human capital.

## Supporting information

S1 Table Interventions details of WINGS-scale up. (DOCX)

S1 File SPIRIT checklist. (DOCX)

S2 File STROBE annexure. (DOCX)

S3 File Qualitative forms. (PDF)

S4 File Costing forms. (PDF)

S5 File Facility assessment forms. (PDF)

S6 File Household survey forms. (PDF)

S7 File Detailed WINGS proposal. (PDF)
