# Supplementary material for: Implementation research to scale up the women and infants integrated interventions for growth study (WINGS) in Himachal Pradesh: Protocol for a quasi-experimental, mixed-methods study
Source: PLoS One. 2026 Feb 17;21(2):e0341048. doi: 10.1371/journal.pone.0341048 (PMC12912596; doi:10.1371/journal.pone.0341048)
Supplement: S1 Table — (DOCX) [file pone.0341048.s001.docx]

**Implementation Research to scale up the women and infants integrated interventions for growth study (WINGS) in Himachal Pradesh: Protocol for a quasi-experimental, mixed-methods study.**

**CONTENT
Supplementary Table 1:** Interventions details of WINGS-scale-up

**Supplementary Table 1: Interventions details of WINGS-scale up**

The interventions are in four domains during the preconception, pregnancy, and early childhood (0-24 months) periods are as under. The detail interventions and strategies are described below

**Preconception (18-35 years women)**

**Screening**

| **Domain** | **Screening** | **Existing** | **Intervention through WINGS** |
| --- | --- | --- | --- |
| Health | Medical Conditions known to affect foetal/infant growth; RTI/STI; TB, hypothyroidism, hypertension, diabetes | On-demand in an OPD basis | Women will be called for the Group meetings which will be organized at the nearest facility with the purpose of collection of Samples, and clinical examination. The Screening will be done annually except for ascertain signs/symptoms of RTI which will be done every 3 months. (2 meetings at AWC and 2 will be at HWC-SC, In all the meetings ASHA, AWW and other concern staff from WCD and Health should be present) |
| Nutrition | Screening of BMI | On-demand in an OPD basis | Women will be called for the Group meetings which will be organized at the nearest facility with the purpose of collection of Samples, and clinical examination. Collection of samples will be done annually, only if clinically indicated, samples will be collected every 3 months. The Screening will be done every 3 months. (2 meetings at AWC and 2 will be at HWC-SC) |
| Psychosocial Care | Screening of depressive symptoms | Not done | Screening will be conducted at the nearest facility using the Patient Health Questionnaire (PHQ-2), which is currently being used in facilities. This will be done every 3 months when conducting RTI screening. |
| WaSH |  | Swachh Bharat Program; Menstrual Health Management; hand washing | Counselling will be done in Group Meetings and during home visits to improve awareness regarding personal hygiene, hand washing, and safe drinking water and appropriate sanitation practices. |

**Prophylaxis and Treatment**

| **Domain** | **Treatment** | **Existing** | **Intervention through WINGS** |
| --- | --- | --- | --- |
| Health | Clinically identified with medical conditions like- Reproductive Tract Infection /Sexually Transmitted Disease, TB, diabetes, hypothyroidism, and hypertension. | Treated as per standard treatment guidelines at the nearest health facility. | - Ensuring **treatment and individual follow-up of cases.** For STI, the husband will also be treated. One dose can be observed in facilities. - Providing contraceptive measures to delay pregnancy until women are infection-free, nutritionally replete and in a positive state of mental health when they conceive. |
|  | If clinically identified with anaemia | Treatment with IFA | Prophylactic doses will be given to non-anemic women once a week.  For women who are anemic, treatment with IFA (current preparation in the health systems of iron and folic acid) delivered by AWW & ASHA   - Prophylactic doses will be given for 15 days (Fortnightly Home visit). - Observed intake will be done on the day of home visit. - The rest of the day's intake photos will be uploaded on the digital app which will be verified by ASHA.   Vitamin B12 will be provided through multiple micronutrients having a higher dose of vitamin B12 |
| Nutrition | Micronutrient supplementations | Not given | Multi micronutrients to be given for 15 days by AWW & ASHA. This is for prophylaxis and will be given thrice a week.  The rest of the day's intake photos will be uploaded on the digital app which will be verified by ASHA. |
| Nutrition  WCD | BMI<18.5 kg/m2 | Women in the age group of 18-35 years are not being covered. | Supplementary nutrition/food supplements will be provided to married women in the age group of 18-35 years having BMI less than 18.5 kg/m2.   - **BMI<16** : 1000kcal + 11.5-16gm protein as locally prepared snacks and egg/milk (70 kcal +6gm protein). Referred to hospital for screening and management. - **BMI 16-18.49** :500 Kcal + 4.5 -7gm protein as locally prepared snacks and egg/milk (70 kcal +6gm protein). egg/milk - Supplementary Calorie Nutrition and Protein Nutrition will be provided weekly by AWC with one compulsory observed intake during visit. - Women will be weighed 3 monthly till recovery   The rest of the day's intake photos will be uploaded on the digital app which will be verified by ASHA. |
|  |  |  | Procurement of Dietary measuring scale at Anganwadi centres. (Supplementary nutrition/Food item measuring equipment) |
|  |  |  | Procurement of storage containers for food material. |
| Psychosocial Care |  | Mental Health helpline: Tele MANAS along with 104 call centres | Counselling services on psychosocial care through trained healthcare providers (ASHA/CHO, Teleconsultation by Medical officers and Nai Disha Kendra (NDK) Counsellor) |

**Pregnant women**

**Screening**

| **Domain** | **Screening** | **Existing** | **Intervention through WINGS** |
| --- | --- | --- | --- |
| Health | Complete blood count, Oral Glucose tolerance test, Blood Grouping, HIV, TSH, VDRL, Urine routine examination and blood pressure monitoring | ANC: 4 ANC | ANC: 8 ANC |
|  |  | OGTT: 1 time | OGTT: 1 time |
|  |  | Urine routine & microscopy: 2-3 times | Urine routine & microscopy: 4 times For asymptomatic bacteriuria, culture sensitivity will be done |
|  |  | Hb: 1 time | Hb: 4 time during pregnancy i.e. at the time of registration, 20, 28, 34-35 weeks sufficient. |
|  |  | CBC: none | At registration |
| Health | Screening of Thyroid disorder | Thyroid Stimulating Hormone test (only if required) | Only once at registration |
| Nutrition | For BMI, GWG | ANC | ANC: BMI in the first trimester; tracking of GWG at all ANC visits |
| Psychosocial Care | Screening of depressive symptoms | Not done | Screening using the Patients Health Questionnaire (PHQ-2 or local instrument currently in use) in each trimester health facility at ANC contact |

**Prophylaxis and Treatment**

| **Domain** | **Treatment** | **Existing** | **Intervention through WINGS** |
| --- | --- | --- | --- |
| Health | Clinically identified with medical conditions like- diabetes, hypothyroidism, hypertension. | Treated as per standard treatment guidelines at the nearest health facility | Ensuring treatment and individual follow-up of cases by CHO, AWW & ASHA (The rest of the day's intake photos will be uploaded on the digital app which will be verified by ASHA. ) |
| Health | Prophylactic supplementation with Calcium (1000 mg) and vit D (400 IU) | During pregnancy and lactation | Strengthening, treatment by CHO, AWW & ASHA   - Prophylactic doses will be given for 15 days (Fortnightly Home visit). |
| Health | Antihelminth at 20 weeks. | During pregnancy after 1st trimester | To be given at 20th week of antenatal contact  The rest of the day's intake photos will be uploaded on the digital app which will be verified by ASHA. |
| Nutrition | Micronutrient supplements  IFA | Not given | Multi-micronutrient to all PW for 9 months daily for prevention of Nutrition deficiency  IFA/FA will be given daily based on the anaemia status   - The rest of the day's intake photos will be uploaded on the digital app which will be verified by ASHA. |
| Nutrition  WCD | Pregnant Women | Supplementary nutrition is provided to the pregnant women under the Supplementary Nutrition Programme viz 600 Kcal energy and protein 18-20 gms enrolled in the Anganwadi Centres | Supplementary nutrition/food supplements to all pregnant women in the District customised based on the gestational age and gestational weight gain.   - The rest of the day's intake photos will be uploaded on the digital app which will be verified by ASHA. |
|  | PW with Inadequate Weight gain |  | Extra Provision of supplementary nutrition to pregnant women having inadequate gestational weight gain   - The rest of the day's intake photos will be uploaded on the digital app which will be verified by ASHA. |
|  |  |  | AWWs to encourage the intake of food supplements. |
| Psychosocial Care |  | Mental Health Helpline: Tele MANAS along with 104 call centre | Counselling services through trained healthcare providers |
| WaSH |  | Swachh Bharat Program; hand washing | Counselling to improve awareness regarding personal hygiene, hand washing, safe drinking water practices, appropriate sanitation practices |

**Postnatal Mothers till 6 months**

**Screening**

| **Domain** | **Screening** | **Existing** | **Intervention through WINGS** |
| --- | --- | --- | --- |
| Health | Identification of Danger Signs in postnatal period till 42 days | Under HBNC:6 visits (Institutional Delivery) and 7 visits (home deliveries) within 42 days of delivery (3, 7, 14, 21, 28 and 42) | Empower family to identify danger signs and seek early care by ASHA & AWW. |
|  |  |  | ASHA will be mapped to the medical officers/ specialist for home-based consultation |
| Psychosocial Care | Screening of depressive symptoms | Not done | Screening using Patients Health Questionnaire (PHQ-2) by AWW & ASHA during the HBNC on day 7, 28 42 and may be at 2 and 6 months for late onset |

**Treatment**

| **Domain** | **Treatment** | **Existing** | **Intervention through WINGS** |
| --- | --- | --- | --- |
| Nutrition (Health) | Nutritional supplements | Daily, 1 Iron and Folic Acid tablet for 180 days, post-partum | Provision of Multi micronutrient & Calcium/ IFA and Vitamin D   - Prophylactic doses will be given for 15 days (Fortnightly Home visit). - The rest of the day's intake photos will be uploaded on the digital app which will be verified by ASHA. |
|  |  | 360 tablets of calcium in the first six months of the postnatal period (@ 2 tablets per day for 6 months). |  |
| Nutrition (WCD) |  | Supplementary nutrition is provided to the lactating mothers under Supplementary Nutrition Programme viz 600 Kcal energy and protein 18-20 gms enrolled in the Anganwadi Centres. | Supplementary nutrition/food supplement to all lactating mothers in the District.   - The rest of the day's intake photos will be uploaded on the digital app which will be verified by ASHA. |
| WaSH |  | Swachh Bharat Program; hand washing | Counselling to improve awareness regarding personal hygiene, hand washing, safe drinking water practices, appropriate sanitation practices.   - The rest of the day's intake photos will be uploaded on the digital app which will be verified by ASHA. |

**Early Childhood (0-24 months): (0-6m)**

**Screening**

| **Domain** | **Screening** | **Existing** | **Intervention through WINGS** |
| --- | --- | --- | --- |
| Health | Identification of Danger Signs of 0-24 months infant and child | **Under HBNC: 6 visits** (Institutional Delivery) and 7 visits (home deliveries) within 42 days of child-birth by ASHA.  Additional visits will be made for Low Birth Weight (LBW) or sick babies being discharged from SNCUs | Empower family to identify danger signs and seek early health care by AWW & ASHA. |
|  |  | **Under HBYC: 5 visits** within 3 to 15 months of child-birth by ASHA | ASHA will be mapped to the specialist for home-based consultation. |
|  |  | **Under ICDS: 9 visits** from 7months - 22 months after child-birth by AWW  Weights will be measured at each visit to identify IWG |  |
| Nutrition  WCD | Child development | Measurement of height and weight of 0-2 years children in AWCs. | Measurement of height & weight of 0-2 years children every month as per the ICDS schedule |
|  |  |  | Counselling on clean play area for he children in age group of 1-2 years. |

**Prophylaxis and Treatment**

| **Domain** | **Treatment** | **Existing** | **Intervention through WINGS** |
| --- | --- | --- | --- |
| Nutrition | For Nutrition support | Early initiation and exclusive breast feeding for 6 months | **0-6 months:** support exclusive breast feeding by rewarding mothers for their good practices that will achieve doubling of birth weight. |
|  |  |  | **6-24 months**: support complementary feeding and continuous breast feeding by rewarding mothers for their good practices that will achieve tripling of birth weight. |
| Nutrition | For all 0-6 months & Low Birth Weight | Not given | **For all infants:** |
|  |  |  | Vitamin D 400 IU daily up to 6 months |
|  |  |  | Iron Supplementation: IFA (10 mg iron and 100 mcg folic acid) supplementation daily up to 24 months for VLBW from 2 weeks and LBW from 6 weeks as prescribe   - The rest of the day's intake photos will be uploaded on the digital app which will be verified by ASHA. |
| Nutrition | For preterm and Low Birth Weight | Family participatory care and KMC at health facility level, MCP card, ECD call center to focus on first 1000 days | Support kangaroo mother care **at home** |
|  |  | Growth Monitoring through HBNC and HBYC visit |  |
| Nutrition  WCD | Inadequate weight gain | Supplementary nutrition is provided to children in the age group of 6 months to 3 years & 3-6 years under Supplementary Nutrition Programme viz 500 Kcal energy and protein 12-15 gms | Supplementary nutrition/food supplement to be provided to all children ages 6 months to 1 year (2 years) and additional food to those with In-adequate Wight Gain.   - The rest of the day's intake photos will be uploaded on the digital app which will be verified by ASHA. |
|  |  |  | 1-2 years: Management of inadequate weight gain. |
|  |  |  | Responsive feeding (By AWW) |
| Psychosocial care: Promote early child play and responsive care  Identification of delayed development and timely referral. | Observation of the child development. | -Saksham Anganwadi & Poshan 2.0 **HBYC**  -MCP Card with ECD component  -Guiding Note for Early Childhood -Development **(ECD**) Call Center  -Operational Guidelines on ECD | As per the HBNC , HBYC visits and ICDS visits the delay in any development will be identified and timely referrals will be done by the ASHA and AWW. |
| WaSH:  Provide play mat and potty.  Continuation of wash interventions provided in pregnancy. | - | - | Counselling on clean play area for children in age group of 6months-2 years.  Hand hygiene, safe disposal of faeces, safe drinking water and personal hygiene during the contacts and home visits will be done by AWW and ASHA. |
