# Supplementary material for: Implementation research to scale up the women and infants integrated interventions for growth study (WINGS) in Himachal Pradesh: Protocol for a quasi-experimental, mixed-methods study
Source: PLoS One. 2026 Feb 17;21(2):e0341048. doi: 10.1371/journal.pone.0341048 (PMC12912596; doi:10.1371/journal.pone.0341048)
Supplement: S1 File — (DOCX) [file pone.0341048.s002.docx]

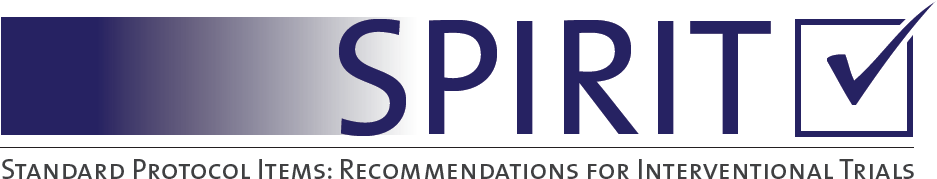


SPIRIT 2013 Checklist: Recommended items to address in a clinical trial protocol and

related documents*

| **Section/item** | **Item No** | **Description** | |
| --- | --- | --- | --- |
| **Administrative information** | | | |
| Title | 1 | | Implementation Research to scale up the Women and Infants integrated interventions for Growth Study (WINGS) in Himachal Pradesh: Protocol for a quasi-experimental, mixed-methods study |
| Trial registration | 2a | | This study has been registered prospectively with Clinical Trials Registry- India, CTRI/2023/10/058538 (Registered on 11/10/2023). |
|  | 2b | | All items from the World Health Organization Trial Registration Data Set |
| Protocol version | 3 | | Date: 12 September 2024, and version identifier: Version 2.0 |
| Funding | 4 | | The current study has been funded by the Indian Council of Medical Research (ICMR) which is a central coordinating unit for this multi-centric study. ICMR personnel have been involved along with other co-authors in the conceptualization and preparation of the manuscript |
| Roles and responsibilities | 5a | | Names, affiliations, and roles of protocol contributors   1. Arun Singh Jadaun, Society for Applied Studies, New Delhi, roles: Project administration, final approval of manuscript, email: [arun.jadaun@sas.org.in](mailto:arun.jadaun@sas.org.in) 2. Jaideep Kumar, Society for Applied Studies, New Delhi, roles: Project administration, final approval of manuscript, email: [jaideep.kumar@sas.org.in](mailto:jaideep.kumar@sas.org.in) 3. Barsha Gadapani Pathak, Scientist, Society for Applied Studies, New Delhi, roles: Conceptualization, methodology, Writing – review & editing, final approval of manuscript, email: [barsha.pathak@sas.org.in](mailto:barsha.pathak@sas.org.in) 4. Sudha Devi, Office of the Chief Medical Officer Una (HFW), roles: Project resources, Supervision, final approval of manuscript, email: [healthsecy-hp@nic.in](mailto:healthsecy-hp@nic.in) 5. Dr. Pradeep Kumar Thakur, National Health Mission, India, roles: Project resources, final approval of manuscript, [md-hp-nrhm@nic.in](mailto:md-hp-nrhm@nic.in) 6. Gopal Beri, Office of Chief Medical Officer Una (HFW),India roles: Project resources, final approval of manuscript, email:   email: [dirhealthdhs@gmail.com](mailto:dirhealthdhs@gmail.com)   1. Mohan Dutt, Women and Child Department, Himachal Pradesh, roles: Project resources, final approval of manuscript, email:   email: [wcd-hp@nic.in](mailto:wcd-hp@nic.in)   1. Seema Thakur, Women and Child Department, Himachal Pradesh, roles: Project resources, final approval of manuscript, email:   email: [seemathakur398@gmail.com](mailto:seemathakur398@gmail.com)   1. Sanjiv Kumar Verma, Office of Chief Medical Officer, Himachal Pradesh, roles: Project resources, final approval of manuscript, email:   email: [sanjivkrverma1967@gmail.com](mailto:sanjivkrverma1967@gmail.com)   1. Sanjay Mankotia, Office of Chief Medical Officer, Himachal Pradesh, roles: Project resources, final approval of manuscript, email:   email: [sanjivkrverma1967@gmail.com](mailto:sanjivkrverma1967@gmail.com)   1. Anjali Chauhan, National Health Mission, Himachal Pradesh, roles: Project resources, Supervision, final approval of manuscript, email: [spomhhp@gmail.com](mailto:spomhhp@gmail.com) 2. Ravinder Kuma^3^, National Health Mission, Himachal Pradesh, roles: Project resources, final approval of manuscript, email:   email: [ravindermph@gmail.com](mailto:ravindermph@gmail.com)   1. Ankit Chaudhary, Office of Chief Medical Officer, Himachal Pradesh, roles: Project resources, final approval of manuscript, email:   email: [sanjivkrverma1967@gmail.com](mailto:sanjivkrverma1967@gmail.com)   1. Richa Kalia, Office of Chief Medical Officer, Himachal Pradesh, roles: Project resources, final approval of manuscript, email:   email: [richakalia1987@gmail.com](mailto:richakalia1987@gmail.com)   1. Devinder Sharma, Society for Applied Studies, New Delhi, roles: Project Administration, final approval of manuscript, email: [devinder.sharma@sas.org.in](mailto:devinder.sharma@sas.org.in) 2. Ranadip Chowdhury, Society for Applied Studies, New Delhi, roles: Conceptualization, final approval of manuscript, email: [ranadip.chowdhury@sas.org.in](mailto:ranadip.chowdhury@sas.org.in) 3. Neeta Dhabhai, Society for Applied Studies, New Delhi, roles: Conceptualization, final approval of manuscript, email: [neeta.dhabhai@sas.org.in](mailto:neeta.dhabhai@sas.org.in) 4. Vinod Kumar Anand, Society for Applied Studies, New Delhi, roles:, Conceptualization, final approval of manuscript, email: [vinod.anand@sas.org.in](mailto:vinod.anand@sas.org.in) 5. Sunita Taneja, Society for Applied Studies, New Delhi, roles: Conceptualization, final approval of manuscript, email: [sunita.taneja@sas.org.in](mailto:sunita.taneja@sas.org.in) 6. Nita Bhandari, Society for Applied Studies, New Delhi, roles: Conceptualization, final approval of manuscript, email: [nita.bhandari@sas.org.in](mailto:nita.bhandari@sas.org.in) 7. Sarmila Mazumder, Society for Applied Studies, New Delhi, roles: Conceptualization, Project Administration, Supervision, Methodology, Writing – original draft , Writing – review & editing, final approval of manuscript, email: [sarmila.mazumder@sas.org.in](mailto:sarmila.mazumder@sas.org.in) |
|  | 5b | | Name and contact information for the trial sponsor:  The study was funded by the Bill & Melinda Gates Foundation through a grant to the GCI BIRAC. BIRAC contact info: Dr. Debanjana Dey , Senior Program Manager, Biotechnology Industry Research Assistance Council (BIRAC), New Delhi, India; [user-23@birac.nic.in](mailto:user-23@birac.nic.in) |
|  | 5c | | Role of study sponsor and funders, if any, in study design; collection, management, analysis, and interpretation of data; writing of the report; and the decision to submit the report for publication, including whether they will have ultimate authority over any of these activities  The study was funded by the Bill & Melinda Gates Foundation through a grant to the Grand Challenges India (GCI) Biotechnology Industry Research Assistance Council (BIRAC). This study is supported by the Government of Himachal Pradesh in partnership with the Society for Applied Studies under the leadership of NITI Aayog, Indian Council of Medical Research (ICMR) and GCI BIRAC. The funders had no role in the study design or the collection, analysis or interpretation of the data. Funders also have no role in writing the manuscript and the decision to submit the manuscript for publication |
|  | 5d | | Composition, roles, and responsibilities of the coordinating centre, steering committee, endpoint adjudication committee, data management team, and other individuals or groups overseeing the trial, if applicable (see Item 21a for data monitoring committee)  BIRAC, the co-ordinating center, will review the protocol, tools and strategy with the assistance of external experts. The co-ordinating Senior Program Manager of BIRAC, will monitor the process of data collection and have quarterly review meetings. The summary of findings as report will be shared by research team to BIRAC. |
| **Introduction** |  | |  |
| Background and rationale | 6a | | India continues to experience a substantial burden of low birth weight (LBW), small-for-gestational age (SGA), stunting, and poor early childhood growth conditions closely linked to maternal undernutrition and anaemia. Despite the existence of major national programs such as Anemia Mukt Bharat, POSHAN 2.0 and RMNCH+A, the preconception period remains inadequately addressed. Evidence consistently shows that maternal nutritional status before conception and in very early gestation is a critical determinant of fetal growth, birth outcomes, and neurodevelopment. Interventions initiated only during pregnancy often miss this biologically sensitive window and fail to reverse growth faltering.  Recent national and global estimates demonstrate that nearly one in five children in India remain stunted, and more than one-fifth of newborns are born with LBW, signalling persistent intergenerational undernutrition and gaps in maternal care. Multiple longitudinal cohorts and intervention studies have shown that preconception interventions, particularly those addressing anaemia, micronutrient deficiencies, nutrition, mental health, and health behaviours, produce greater improvements in birth weight, gestational growth, and child development than pregnancy-only approaches.  The Women and Infants Integrated Growth Study (WINGS) trial in Delhi provided robust evidence that an integrated package spanning preconception, pregnancy, and early childhood, delivered through health, nutrition, psychosocial, and WaSH domains, significantly reduced LBW and stunting at 24 months. The success of WINGS highlights that multi-domain, continuous support, rather than isolated, pregnancy-stage interventions, is necessary to break the cycle of undernutrition.  However, there is limited understanding of how such an integrated package can be delivered at scale through government systems, especially in hilly or hard-to-reach settings requiring strong multisectoral coordination. Himachal Pradesh, with its demonstrated commitment to strengthening preconception care and its inclusion in national equity initiatives, presents an ideal context to co-develop and test a scalable government-led model.  This trial therefore seeks to address the following research question: Can a government-led, co-designed scale-up of a multi-domain preconception-to-childhood intervention (adapted from WINGS) improve maternal nutritional status, birth outcomes, and child growth when integrated into routine public health and nutrition services in Himachal Pradesh?  To answer this, the study proposes a theory-driven scale-up model implemented in Una district, optimisation of strategies through participatory co-design with government stakeholders, and evaluation of population-level effects using an interrupted time series design. Findings will provide a replicable implementation blueprint for other states aiming to institutionalise high-impact preconception interventions.  No significant harms are anticipated, as the intervention components, including nutrition supplementation, counselling, psychosocial support, and WaSH practices, are all evidence-based and already recommended within national programs. The trial instead focuses on optimising delivery, strengthening multisectoral coordination, and demonstrating effectiveness at scale, thereby addressing a major gap in India’s maternal and child health strategy. |
|  | 6b | | In this implementation research, no parallel control arm is used because the objective is to optimise and scale an integrated government-led intervention across all blocks of Una district. A quasi-experimental Interrupted Time Series (ITS) design is therefore the most appropriate comparator framework. The comparator is the pre-implementation period, which reflects the routine standard of care currently delivered through existing government health and nutrition platforms.  This pre-intervention phase provides a stable baseline to assess population-level trends in anaemia, gestational weight gain, and infant growth prior to rollout. By collecting three pre-intervention and three post-intervention survey rounds, the ITS design allows differentiation between underlying secular trends and changes attributable to the WINGS implementation model. |
| Objectives | 7 | | The primary outcomes of this study are focused on strengthening health systems and streamlining processes by identifying existing gaps and implementing innovative, sustainable solutions to improve maternal and child health, alongside enhancing commitment and mobilizing resources through evidence-informed advocacy. A secondary outcome is to estimate the incremental cost of implementing WINGS interventions within government health systems. |
| Trial design | 8 | | Description of trial design including type of trial (eg, parallel group, crossover, factorial, single group), allocation ratio, and framework (eg, superiority, equivalence, noninferiority, exploratory):  We will use a convergent mixed‑methods design using principles of implementation science. Implementation will occur in the ‘learning block’ first, with iterative optimization over two to three adaptation cycles, followed by simultaneous expansion to the remaining blocks. A theory‑driven process evaluation will run in parallel to explain the observed effects and inform ongoing adaptation. Additionally, an interrupted time-series (ITS) design will be applied across all study blocks to evaluate temporal changes in outcomes before and after implementation. This quasi-experimental approach is particularly suitable for evaluating large-scale policy and programme interventions where randomization is not feasible and allows separation of underlying trends from those associated with the intervention. |

| **Methods: Participants, interventions, and outcomes** | | | |
| --- | --- | --- | --- |
|  |  |  | |
| Study setting | 9 | Description of study settings (eg, community clinic, academic hospital) and list of countries where data will be collected. Reference to where list of study sites can be obtained:  This study will be conducted entirely in India, within the public health and nutrition system of Una district, Himachal Pradesh. Una has been purposively selected as the pilot site for scaling up the Women and Infants Integrated Growth Study (WINGS) implementation model due to its mixed terrain (plains and hilly areas), moderate health indicators, and strong government commitment to strengthening preconception and maternal health services.  Data will be collected across all five development blocks of Una district—Una (Basdehra), Amb, Gagret, Dhundla (Thanakalan), and Haroli. The study setting comprises routine government service delivery platforms under the National Health Mission (NHM) and Integrated Child Development Services (ICDS). These include:   - 1364 Anganwadi Centres (AWCs) - 138 Health and Wellness Centres/Sub-centres (HWCs/SCs) - 24 Primary Health Centres (PHCs) - 9 Community Health Centres (CHCs) - 5 Civil Hospitals - 1 Regional Hospital   These facilities deliver preconception, antenatal, postnatal, and early childhood services. Frontline health workers—Accredited Social Health Activists (ASHAs), Auxiliary Nurse Midwives (ANMs), Community Health Officers (CHOs), and Anganwadi Workers (AWWs)—will support implementation and data collection activities.  The study sites also include selected households in all five blocks, where quarterly community surveys and outcome assessments (anthropometry, anaemia status, service utilisation, and infant growth) will be conducted. Facility assessments (readiness, supplies, service quality) will be carried out at PHCs, CHCs, hospitals, and AWCs. | |
| Eligibility criteria | 10 | Inclusion and exclusion criteria for participants. If applicable, eligibility criteria for study centres and individuals who will perform the interventions (eg, surgeons, psychotherapist)  **Eligibility of Participants**  **Preconception women:** Eligible participants include married women aged 18–35 years who have not completed their families, are not currently pregnant, and are permanent residents of the study area. Women will be excluded if they are temporary residents, decline consent, or have medical conditions that prevent participation.  **Pregnant women:** Pregnant women residing in the study blocks with a confirmed pregnancy will be included. Exclusion criteria include refusal or withdrawal of consent and non-residency in the study area. Women identified postpartum before baseline assessments will not be included in pregnancy-specific analyses.  **Infants and young children (0–24 months):** Children aged 0–24 months born to resident women in Una district will be eligible for growth and outcome assessments. Infants will be excluded if a primary caregiver is unavailable to provide informed consent.  **Frontline and facility workers:** ASHAs, AWWs, ANMs, CHOs, and facility staff will be included for qualitative interviews, process evaluations, and fidelity assessments. Only those posted within the study blocks and willing to participate will be eligible. There are no clinical exclusion criteria for this group apart from refusal to participate.  **Eligibility Criteria for Study Centres**  All public-sector service delivery platforms under the National Health Mission (NHM) and Integrated Child Development Services (ICDS) in Una district are eligible, including Anganwadi Centres, Health and Wellness Centres/Sub-centres, Primary Health Centres, Community Health Centres, Civil Hospitals, and the Regional Hospital. Centres must be operational, located within the five selected blocks, and engaged in maternal and child health and nutrition service delivery. No study centres are excluded, as full district-wide implementation is integral to the design.  **Eligibility Criteria for Individuals Delivering the Interventions**  Interventions will be delivered exclusively through existing government health and nutrition cadres. Eligible personnel include ASHAs, AWWs, ANMs, CHOs, medical officers, and district/block programme managers involved in maternal and child health services. Individuals must be currently employed under NHM or ICDS, posted within an intervention block, and trained in WINGS implementation protocols. No private providers or external organisations will deliver interventions. Exclusion applies only to those unwilling to participate in implementation training or supervisory processes. | |
| Interventions | 11a | The interventions are in four domains during the preconception, pregnancy, and early childhood (0-24 months) periods. The detail interventions and strategies are described in **S1 Table.** | |
|  | 11b | Criteria for discontinuing or modifying allocated interventions for a given trial participant (eg, drug dose change in response to harms, participant request, or improving/worsening disease):  Not Applicable in this study | |
|  | 11c | Strategies to improve adherence to intervention protocols, and any procedures for monitoring adherence (eg, drug tablet return, laboratory tests)  Monitoring and RE-AIM Indicators, listed in the study protocol, will be used to assess adherence to intervention protocol. To ensure high-quality and consistent delivery of the WINGS intervention package, multiple strategies will be implemented across government health and nutrition platforms. Adherence will be strengthened through capacity-building, including structured training, refresher micro-modules, on-the-job coaching, and use of standardized job aids for ASHAs, AWWs, ANMs, CHOs, and facility staff. Supportive supervision and practice facilitation will be conducted through scheduled field visits using structured checklists, complemented by monthly block- and district-level review meetings to reinforce expectations, identify deviations, and implement corrective actions. Behavioural strategies guided by the COM-B framework—including addressing capability gaps through skill-building, ensuring opportunity through strengthened supply chains and workflow adjustments, and enhancing motivation via recognition and peer-learning—will support consistent practice.  Adherence will be continuously monitored using a combination of routine programme data, quarterly process indicators, and field verification. This will include tracking IFA/MMS distribution and consumption, nutritional supplementation for undernourished women, ANC registration and contact schedules, gestational weight gain monitoring, screening for anaemia and thyroid disorders, postnatal follow-up, and infant growth assessments. Supervisors will review CHW registers, digital tally sheets, and harmonised monitoring tools to assess fidelity to counselling protocols and intervention components. The Programme Learning Team will conduct concurrent qualitative assessments, observations, and rapid feedback cycles to identify bottlenecks and inform adaptations. Together, these mechanisms will ensure robust adherence monitoring and support ongoing optimisation of intervention delivery. | |
|  | 11d | Relevant concomitant care and interventions that are permitted or prohibited during the trial : Not applicable | |
| Outcomes | 12 | Primary, secondary, and other outcomes, including the specific measurement variable (eg, systolic blood pressure), analysis metric (eg, change from baseline, final value, time to event), method of aggregation (eg, median, proportion), and time point for each outcome. Explanation of the clinical relevance of chosen efficacy and harm outcomes is strongly recommended  Specific outcome indicators include the proportion of preconception women (married, reproductive age, not completed families) who are non-anemic and those with a normal BMI (18.5–24.99 kg/m²). For pregnant women, outcomes include proportions who are non-anemic, achieve adequate gestational weight gain, and maintain normal thyroid levels. For infants, outcomes focus on adequate weight gain from birth to one year. The outcome indicators will be assessed through quarterly monitoring during implementation and three rounds, each of pre and post-intervention surveys | |
| Participant timeline | 13 | Time schedule of enrolment, interventions (including any run-ins and washouts), assessments, and visits for participants. A schematic diagram is highly recommended (see Figure)  Although the study is planned to be conducted in a phased manner, considerable overlap and concurrent activities are anticipated. The initial preparatory phase (formative research), lasting approximately six months, will focus on conducting situational analyses and system level diagnosis to identify barriers and facilitators and assess contextual readiness within existing government systems. The model optimization will be conducted in the learning block, which will be concurrently scaled up in the remaining blocks with continuous monitoring, adaptive learning cycles, and iterative refinements based on implementation feedback as per contextual needs. spanning two years. Data analysis and synthesis, will be an ongoing process. Final report preparation and dissemination, in the final six months, will focus on analyzing and compiling the overall quantitative and qualitative data across all blocks, consolidating lessons learned, and generating evidence to inform state and national policy translation for scale-up of the WINGS model.  **Formative phase**  The formative phase will be led by the PLT in the first implementation block using mixed methods to conduct a system-level diagnosis and identify barriers and facilitators to implementing the WINGS intervention. Quantitative assessments will include facility and community surveys, while qualitative components will comprise in-depth interviews, focus group discussions and structured observations. The formative research will explore key domains such as population profile and potential beneficiaries, health and ICDS infrastructure and human resources, service availability and utilization across the four WINGS domains (health, nutrition, psychosocial care and WaSH), referral mechanisms and transport availability, coverage and quality of relevant national programs, digital platforms and data systems, SBCC and community engagement platforms, awareness and utilization of government schemes and the role of local non-governmental organizations in maternal and child health. In parallel, the OMT will conduct baseline surveys using an interrupted time-series (ITS) design, with three survey rounds planned to capture key outcome indicators. The overall duration of the formative phase, including baseline data collection, will be approximately nine months.  **Model Optimization phase** Implementation will begin with a base model (“Model 0”) integrating existing pregnancy and childhood interventions, plus additional WINGS components. Model 0 will evolve into Model 1 by incorporating formative research, followed by rapid cycles of implementation, monitoring, and refinement through co-design workshops with government partners. High coverage, defined with government as at least a 20% improvement over baseline, will serve as the benchmark for success. Considering the complexity of the multiple interventions and domains, the target coverage of each outcome may not be achieved concurrently at the same point. This may happen in a phased manner.  Model optimization in the first block (~9 months) will involve 2–3 iterative cycles. Subsequent blocks will require ~6 months each, with contextual adaptations as needed. All government facilities and frontline workers (district hospital, CHCs, PHCs, HWCs, SCs, medical college hospital, and ICDS/AWCs) will be engaged. The full project, including preparatory work, optimization, rollout across five blocks, outcome monitoring, reporting, and dissemination, is expected to be completed in ~36 months.  **Figure 2: Model optimization using iterative cycles.**  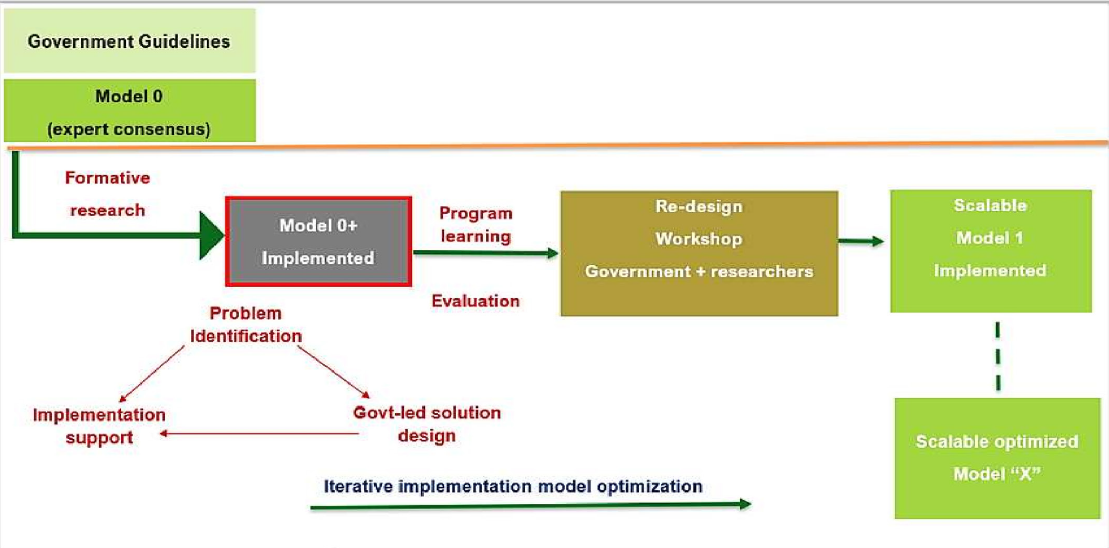  *Figure adapted from World Health Organization and re-used from published paper.(25).* | |
| Sample size | 14 | Estimated number of participants needed to achieve study objectives and how it was determined, including clinical and statistical assumptions supporting any sample size calculations.  Data on key outcomes will be aggregated at block level across five blocks, with measurements at three pre-intervention and three post-intervention time points. The study is powered to detect a 20% change in outcomes with 88.3% power (95% CI: 86.3–90.3), estimated using the *itspower* package in Stata 17. Outcomes of interest include adequate gestational weight gain (IOM standards), non-anemia (Hb ≥11 g/dl), normal BMI (18.5–25 kg/m²), and adequate child weight gain (WHO standards). Based on NFHS-5 and WINGS baseline data, expected improvements are from 55% to 66% for adequate GWG and normal BMI, 45% to 54% for non-anemia, and 60% to 72% for child weight gain. A sample size of 600 women and children per survey round will be adequate, with 400 as the minimum feasible requirement. | |
| Recruitment | 15 | Strategies for achieving adequate participant enrolment to reach target sample size”  Households for surveys will be randomly selected to identify eligible women/infants, from the line listings of all households at village level. Villages selected proportional to size in each block. | |
| **Methods: Assignment of interventions (for controlled trials)** | | | |
| Allocation: |  | |  |
| Sequence generation | 16a | | Method of generating the allocation sequence (eg, computer- generated random numbers), and list of any factors for stratification. To reduce predictability of a random sequence, details of any planned restriction (eg, blocking) should be provided in a separate document that is unavailable to those who enrol participants or assign  Interventions  Not applicable for this study |

| Allocation concealment mechanism | 16b | Mechanism of implementing the allocation sequence (eg, central telephone; sequentially numbered, opaque, sealed envelopes), describing any steps to conceal the sequence until interventions are assigned  Not applicable for this study |
| --- | --- | --- |
| Implementation | 16c | Who will generate the allocation sequence, who will enrol participants, and who will assign participants to interventions  Not applicable for this study |
| Blinding (masking) | 17a | Who will be blinded after assignment to interventions (eg, trial participants, care providers, outcome assessors, data analysts), and how  Not applicable for this study |
|  | 17b | If blinded, circumstances under which unblinding is permissible, and procedure for revealing a participant’s allocated intervention during the trial  Not applicable for this study |
| **Methods: Data collection, management, and analysis** | | |
| Data collection methods | 18a | Data collection will include both quantitative and qualitative components using harmonised tools across government platforms. Quantitative data will be obtained through quarterly household surveys conducted during pre- and post-intervention periods to capture maternal nutrition indicators such as diet diversity and IFA adherence, anthropometry, morbidity, service utilisation, and infant feeding and growth outcomes. Facility assessments at baseline and endline will document service availability, readiness, equipment, consumables, counselling space, and record quality, while surveys with frontline workers will assess their knowledge, skills (including observed counselling practices), workload, and supervision. Routine programme data will also be compiled monthly from Health and ICDS systems, including ANC registration before 12 weeks, beneficiary footfall, distribution of food supplements, IFA and MMS supplies, counselling contacts, referrals, and outcome counts.  Qualitative data collection will include in-depth interviews with mothers and families across the preconception, pregnancy and postnatal periods; interviews with ASHAs, AWWs, ANMs, facility staff and managers; focus group discussions with community groups and CHWs; non-participant observations of counselling sessions, outreach activities and facility workflow; and systematic documentation of implementation adaptations. Audio recordings and field notes will be de-identified during transcription, managed within NVivo project files, and stored on encrypted drives with restricted access. |
|  | 18b | Plans to promote participant retention and complete follow-up, including list of any outcome data to be collected for participants who discontinue or deviate from intervention protocols  Not Appplicable |
| Data management | 19 | Plans for data entry, coding, security, and storage, including any related processes to promote data quality (eg, double data entry; range checks for data values). Reference to where details of data management procedures can be found, if not in the protocol  Robust data quality assurance procedures will ensure accuracy and reliability. Field teams will undergo double training and standardisation for anthropometric measurements to minimise inter-observer variability. Tablet-based data collection with built-in validation checks will reduce entry errors at source. Supervisors will conduct periodic back-checks and repeat measurements for a subsample of participants. Monthly data audits performed by the DMC will assess completeness, internal consistency and outlier patterns, with timely feedback issued for corrective action. De-identified datasets and analysis code will be shared upon reasonable request following ethics approvals and data-sharing agreements.  All quantitative data workflows will be overseen by the Data Management Centre (DMC) located at the field office in Himachal Pradesh. Data will be captured electronically using SurveyCTO with enforced range and consistency checks, skip logic, and mandatory response fields. This platform enables secure, offline-capable data collection with real-time quality controls suited to diverse and resource-constrained settings. Household, facility, and staff survey data will upload in near real time to a local server, where automated query lists will be generated, reviewed, and resolved by trained coordinators under DMC supervision. A version-controlled data dictionary and SOPs for coding, cleaning, merging, and ITS aggregation will be maintained. All participant records will use pseudonymised unique identifiers; any direct identifiers required for linkage will be stored separately in encrypted, access-restricted files. For the interrupted time-series analysis, the DMC will construct time-stamped, block-level analytic datasets representing three pre-intervention and three post-intervention rounds. |
| Statistical methods | 20a | Statistical methods for analysing primary and secondary outcomes. Reference to where other details of the statistical analysis plan can be found, if not in the protocol  Quantitative data will be analysed using segmented linear regression models in R, drawing on three pre-intervention and three post-intervention survey rounds over nine months. Descriptive statistics and plots will explore trends, seasonality and outliers. Models will estimate immediate level changes and slope changes using time, intervention phase and interaction terms. Generalised linear models will provide effect estimates with 95% confidence intervals for binary, continuous and count outcomes. Autocorrelation will be examined using Durbin–Watson statistics and ACF/PACF plots, with moving averages applied to stabilise extreme values. Qualitative data will be coded and analysed concurrently using descriptive, analytical and theoretical coding approaches. Framework analysis with matrix displays will allow comparison across themes, participant groups and implementation contexts, capturing both frequency and narrative depth to inform iterative programme adaptation. |
|  | 20b | Methods for any additional analyses (eg, subgroup and adjusted analyses)  Not applicable |
|  | 20c | Definition of analysis population relating to protocol non-adherence (eg, as randomised analysis), and any statistical methods to handle missing data (eg, multiple imputation)  Not applicable |
| **Methods: Monitoring** | | |
| Data monitoring | 21a | Composition of data monitoring committee (DMC); summary of its role and reporting structure; statement of whether it is independent from the sponsor and competing interests; and reference to where further details about its charter can be found, if not in the protocol.  Alternatively, an explanation of why a DMC is not needed  There will be a data monitoring committee (DMC) consisting of external experts, Project monitoring committee (PMC) members and BIRAC scientists. Once baseline data collection starts, DMC would visit the site and prepare a brief report stating the work flow, challenges and other relevant issues in. Another visit will be made when interventions will be introduced to document the progress made and challenges |

|  | 21b | Description of any interim analyses and stopping guidelines, including who will have access to these interim results and make the final decision to terminate the trial  Not Applicable as it is an implementation research study protocol |
| --- | --- | --- |
| Harms | 22 | Plans for collecting, assessing, reporting, and managing solicited and spontaneously reported adverse events and other unintended effects of trial interventions or trial conduct  Not Applicable as it is an implementation research study protocol |
| Auditing | 23 | Frequency and procedures for auditing trial conduct, if any, and whether the process will be independent from investigators and the sponsor  In the current study, monitoring visits will be organized with experts from PMC . These visits will document the work flow, challenges and other relevant issues. |
| **Ethics and dissemination** | | |
| Research ethics approval | 24 | Plans for seeking research ethics committee/institutional review board (REC/IRB) approval  Ethical approval from respective Institutional review board has already been taken. |
| Protocol amendments | 25 | Plans for communicating important protocol modifications (eg, changes to eligibility criteria, outcomes, analyses) to relevant parties (eg, investigators, REC/IRBs, trial participants, trial registries, journals, regulators)  This protocol has been finalized after being reviewed by external experts for 2-3 rounds. In case, there will be changes in the protocol, a document stating the same will be circulated with experts ( for their approval), site-investigator and their teams. |
| Consent or assent | 26a | Who will obtain informed consent or assent from potential trial participants or authorised surrogates, and how (see Item 32)  In this implementation research study, before enrolment, all participants, including pre-conception and pregnant women, mothers or primary caregivers of infants and young children, and healthcare providers, will be provided with written informed-consent forms in the local language (Hindi). For participants who are non-literate, the consent form will be read aloud in the presence of an impartial witness, and consent will be documented using a thumb impression. Participation will be entirely voluntary, and individuals may withdraw at any stage without consequence. |
|  | 26b | Additional consent provisions for collection and use of participant data and biological specimens in ancillary studies, if applicable  Not applicable. |
| Confidentiality | 27 | How personal information about potential and enrolled participants will be collected, shared, and maintained in order to protect confidentiality before, during, and after the trial  Data handling and storage will adhere to strict confidentiality and privacy regulations. All personal identifiers will be removed, and data will be anonymized before analysis. Additionally, research team staff and health system staff involved in the study will be trained in good clinical practices (GCP) which is an international ethical and scientific quality standard for the design, conduct, performance, monitoring, auditing, recording, analyses, and reporting of trials and will be standardized for case-records forms, study checklist and data collection and data entry process. |
| Declaration of interests | 28 | Financial and other competing interests for principal investigators for the overall trial and each study site  We do not have any financial and non-financial competing interest. |
| Access to data | 29 | Statement of who will have access to the final trial dataset, and disclosure of contractual agreements that limit such access for investigators  The study investigators. |
| Ancillary and post-trial care | 30 | Provisions, if any, for ancillary and post-trial care, and for compensation to those who suffer harm from trial participation  Not Applicable for the current study. |
| Dissemination policy | 31a | Plans for investigators and sponsor to communicate trial results to participants, healthcare professionals, the public, and other relevant groups (eg, via publication, reporting in results databases, or other data sharing arrangements), including any publication restrictions  The investigators and sponsors will communicate the study findings through publication as well as dissemination seminar/meeting with other healthcare professionals and stakeholders. Open‑access publications; presentations at national/global conferences; quarterly/annual government review meetings; state and national policy briefs; and a publicly accessible synopsis of lessons and tools. |
|  | 31b | Authorship eligibility guidelines and any intended use of professional writers  Not applicable |
|  | 31c | Plans, if any, for granting public access to the full protocol, participant- level dataset, and statistical code  The current study data belongs to state and study investigators and BIRAC. The data will be shared on public domain after both grant the permission for the same. |

| **Appendices** |  |  |
| --- | --- | --- |
| Informed consent materials | 32 | Model consent form and other related documentation given to participants and authorized surrogates:  In addition to consent form, Participant Information sheet will be shared with the participants. |
| Biological specimens | 33 | Plans for collection, laboratory evaluation, and storage of biological specimens for genetic or molecular analysis in the current trial and for future use in ancillary studies, if applicable:  Not applicable for this study. |

*It is strongly recommended that this checklist be read in conjunction with the SPIRIT 2013 Explanation & Elaboration for important clarification on the items. Amendments to the protocol should be tracked and dated. The SPIRIT checklist is copyrighted by the SPIRIT Group under the Creative Commons “[Attribution-NonCommercial-NoDerivs 3.0 Unported](http://www.creativecommons.org/licenses/by-nc-nd/3.0/)” license.
