# Supplementary material for: Implementation research to scale up the women and infants integrated interventions for growth study (WINGS) in Himachal Pradesh: Protocol for a quasi-experimental, mixed-methods study
Source: PLoS One. 2026 Feb 17;21(2):e0341048. doi: 10.1371/journal.pone.0341048 (PMC12912596; doi:10.1371/journal.pone.0341048)
Supplement: S3 File — (PDF) [file pone.0341048.s004.pdf]

A. INTERVIEW GUIDE WITH HEALTH FACILITY STAFF (CMO, SMO, MO, CHO, STAFF NURSES, ANMs, supervisors)

Participant information
Name _______
Age ____ Sex ____
Facility ______ Designation_______ Years of service in the facility
Address of the facility_______
Place of residence/distance from the facility____
How do you commute______________

Interview guide

1. What is the catchment area that your facility caters to? What is the total population of the catchment area? Do you have an updated enumeration of the population in the catchment area? How frequently is this updated? Is there an electronic database of the total population?
2. What is the distance of your facility from the farthest point of your catchment area? Is transportation available/accessible to the community, how do they commute? Are there any other health facilities or sources of care in the catchment area?
3. Are there any vacancies in any cadre at your facility? If yes, please provide the details. [cadre, number, since how long, any plan to fill vacancies]
4. What are the timings of your facility when services are provided? If 24x7, do women and children visit your facility in the evening/night/holidays? If not, where do they go and why?
5. What are the services provided for married reproductive age group women and children 0 to 24 months of age in your facility?
   [Probes:-
   Preconception women
   - Family planning services- contraceptives offered. If any woman is weak, anemic or has any illness, are these women advised to delay pregnancy till their health improves? Are they counselled to use contraceptive device to delay pregnancy or to maintain at least 2 years interval between 2 pregnancies? [these will be probes and not asked as leading questions]
   - Any screening services for women in preconception period (define preconception)- screening for medical conditions such as anemia, RTI/STI, TB, thyroid status, hypertension, diabetes; screening for depressive symptoms; How are these conditions assessed? Counselling on WaSH; if yes by whom, where, how frequently, what messages given?
   - Any treatment provided to preconception women if diagnosed with any morbidity or other conditions requiring treatment- for example, treatment for anemia, RTI/STI, TB, hypothyroidism, hypertension, diabetes, depressive symptoms etc. Are women referred for any condition, if yes for what?
   - Are height weight measured? Is BMI calculated? Is weight of preconception women tracked?
   - Are preconception women given any nutrition supplement from ICDS, if yes elaborate (type, amount, frequency, place of distribution etc). If women are very weak or thin (BMI <18.5kg/m2), is any additional supplement given? If yes, what? How do women access these food (AWW gives at home/a central place/women visit AWCs)
   - Any Multiple micronutrients given to the preconception women? If yes, what, how frequently and process of distribution.
   - How many preconception women utilize services (average/month) from your facility? Do you think all preconception women in your catchment area avail services from your

facility? If yes, for which services? Why do you say so? What are their preferred sources of care seeking?
- Do you face any challenges in delivering these interventions, please elaborate.
- What are the mitigation strategies
- If we need to reach out to preconception women to deliver health, nutrition services, WaSH and psychosocial care, what are your suggestions to do so effectively?

Pregnant and postnatal women
- Are MCP cards given to women?
- ANC – number of ANC visits per woman that is recommended and practiced; content of ANC at each visit, weight, BP, per abdomen examination, clinical examination for RTI, lab investigations, USG, screened for morbidities such as diabetes, thyroid status, hypertension, depressive symptoms, others.
- Anthropometry: height, weight (functional equipment); is BMI calculated in first trimester and gestational weight gain tracked. Management of inadequate gestational weight gain (IGWG)
- Laboratory tests offered and methods- Hb (CBC/PoC), CBC, serum ferritin; thyroid function tests-TSH, tests for RTI-NAAT RTPCR, VDRL, blood sugar-random, fasting, PP; OGTT, HbA1c, urine dipstick; KFT, LFT, urine routine/microscopic/culture)
- If any investigations are not available, how do you manage? If you refer, where do you refer- private/government? Do you have any private laboratory empaneled with your facility or with the government health systems? If yes, is this being utilized with reasons.
- Do women get the investigations done from outside and come back with reports? Elaborate.
- USG- if not available, where do you refer- private/government? Do you have any private USG center empaneled? Do women get the USG done and come back with reports? If not, the reasons?
- Do you anticipate any challenges (from provider and views about beneficiary perspectives) if the number of visits is increased to 8 instead of 4?
- If diagnosed with any morbidity how are pregnant women managed? Are they referred? If yes under what condition and where?
- What proportion of women visiting the facility are high risk? What are the common high risk conditions? How are they managed
- Are pregnant women getting any supplementary nutrition from the ICDS? If yes, what do they get, describe.
- Is there any provision for psychosocial care for pregnant women? Any psychologist or psychiatrists available in your facility? What do you do if you identify the need? Referral? Where?
- How many pregnant women utilize services (average/month) from your facility? Do you think all pregnant women in your catchment area avail services from your facility? Why do you say so? What are their preferred sources of care seeking?
- Are pregnant women counselled on WaSH?(these will be probes to prevent leading questions: personal hygiene, handwashing and safe drinking water and appropriate sanitation practice) If yes, by whom, where, how frequently, what messages given?
- Intra-partum services- normal delivery, CS
- Immediate postnatal care- period of stay in facility postdelivery, KMC, lactation support.
- Nutritional supplement for postnatal women; SNP from ICDS, IFA, Calcium,
- Postnatal care and child health care services; home based postnatal care (HBPNC) by ASHAs, identification and management of danger signs in mother; screening and management of depressive symptoms.
- Do you face any challenges in delivering these interventions, please elaborate.
- What are the mitigation strategies

Children 0 to 24 months

- Are children screened for danger signs 0-24 months? If yes, how (for 0 to 42 days and >42 days till 2 years) and by whom? If identified management of danger signs?
- Anthropometry measurements done for children, by whom and where? Is weight gain tracked over time and any action taken for IWG?
- Are mothers counselled on care of children such as early initiation of BF, EBF till 6 months, immunization, ENC (cord care, warmth, identification of danger signs and referral), KMC and expressed breast milk feeding for preterm and LBW, CF at 6 months, type, frequency, quantity, consistency, responsive feeding etc
- Is any additional care for LBW/preterm babies- EBM, warmth, KMC, SNCU/NICU admission
- Are children given any micronutrient supplementation (calcium, iron, phosphorus, vitamin D)? Are the any supplementation given to LBW or VLBW babies? If yes, by whom? Availability of these medicines?
- Are children getting any supplementary nutrition from the ICDS, if yes, please describe
- Promotion of psychosocial care, early child play, responsive care, identification of delayed development and timely referral
- What is done for WaSH; what messages given, by whom, when and how
- Do you face any challenges in delivering these interventions, please elaborate.
- What are the mitigation strategies

6. **Medicines and supplies, equipment**

What are the medicines available in your facility? Are these given to pregnant women?

- IFA- composition
- Anthelmintics
- Calcium
- Vitamin D
- Multiple micronutrients , multivitamins
- Antihypertensives
- Thyroid medicines
- Antibiotics
- Are there stockouts, how often; what is the procurement system.

7. **Transportation**

- What are the methods of transport to visit facility, come back home after discharge, for referral? Probe for Ambulance services/public transport/private transport- types, availability, accessibility, road condition, time of availability, cost.
- Do the women and families in the community face any challenges in commuting, what are these
- What are the mitigation strategies

8. **Record maintenance**

- Can you show me how the records are maintained at the facility- for reproductive age group married women, pregnant women and children [check register, note observations].
- Do you have any electronic data base? How do you track the beneficiaries during the preconception period, antenatal period- GWG, Hb, others; till delivery and postnatal period. How do you follow up women, ensure that pregnant women come for all ANC visits, comply with the recommended treatment and care?

- Tell me about the referral mechanisms- what are the conditions when you refer? What is the arrangement for referral transportation? How far is the nearest referral facility- private and public? What is preferred by the community for women and children?

9. **Knowledge perception**
   - Are you aware of the health conditions or nutritional status when a preconception woman needs to visit health facility? Are any investigations required for these women? Do they need any supplements (nutrition or medicines)? If yes, what?
   - Do you know what is high risk pregnancy? If yes, tell me the HRP conditions and what needs to be done
   - Are you aware of the health conditions or nutritional status when an under-two child needs to visit health facility? What are the danger signs that need referral
   - Are you aware of the BMI categories? Please explain
   - Are you aware of the gestational weight gain/how much a pregnant woman needs to gain during each trimester of pregnancy and total weight gain
   - Do you know what is inadequate weight gain in preconception women and children, please explain
10. **Are you aware of the national programs** and schemes related to women and children? Are these being followed/implemented at your facility and catchment area? Can you tell me about the activities under each program that are being implemented? If not, why so? What are the challenges?

   Probe if unable to respond spontaneously.
   - JSSK/JSY
   - Mission Parivar Vikas
   - Anemia Mukt Bharat- do preconception women get IFA routinely in this program?
   - Adolescent health program
   - Swachh Bharat Mission Gramin
   - SUMAN
   - PMMVY
   - PMSMA
   - National guidelines for screening hypothyroidism in pregnancy
   - National guidelines for diagnosis and management of GDM
   - National guidelines for calcium supplementation during pregnancy and lactation
   - MAA
   - HBYC
   - HBNC
   - Operational guidelines KMC and optimal feeding of LBW infants
   - Facility based care of SAM
   - Any state specific program
11. **How is the data managed-** electronic/manual and status of HMIS. How is information captured and processed on coverage and quality?

   [Probes: who has access, how difficult or easy is the process, is the data updated and accurate, are data entered directly into tablets/phones or first manually entered and then entered into electronic database? Is it possible to create electronic trackers- what are the challenges and possible solutions?]
12. **What is the reporting system** at your facility, in the district and state- review meetings, frequency, content (data reviewed), actions taken, stakeholders attending.
13. **What is the system of supervision** and monitoring in your facility? What are the barriers and challenges? What are the solutions? Any facilitating factors? Any routine quality control activities?
14. **What is the system of training** for the health staff and CHWs? Who trains, frequency, duration, content? Is there a pool of trainers?

15. **What are the platforms currently being used for SBCC** activities? Please elaborate on the content, frequency, attendance by the community? What can be done to spread awareness in the community for any new programs?
16. What are the **food preferences in your community** by women and young children? Do the women like or dislike the SNP provided through ICDS? Is there any SNP that they like or dislike? Reasons
    - What is the community perception about egg consumption? Is it accepted or are there any barriers due to cultural or religious reasons, taste issue etc.
    - Is there any particular food preference/culturally accepted by the community women (preconception and pregnant women)

B. INTERVIEW GUIDE WITH ICDS STAFF- CDPO AND SUPERVISORS.

Participant information
Name _______
Age ____ Sex ____
Department______ Designation_______ Years of service _______
Address of the office ___________(for ICDS supervisors, mention the AWCs being supervised)
Place of residence/distance from the workplace____
How do you commute______________

Interview guide

1. What is the catchment area population on average covered by each anaganwadi centre in the district? Is there any pocket without any AWC? What is the plan to recruit AWWs and fill up the vacancies?
2. Is the complete population under each AWC enumerated? How is the database maintained, updated and accessed? Is there an electronic database of the total population under each AWC? If database is in place, do you have these by age specific and other categories, for example all eligible couples, married women in reproductive age group with one or no child, pregnant women, 0 to 6 months, >= 6 months to 2 years?
3. What is the usual distance of any AWC from the farthest point of its catchment area? Is it within walking distance or there is a need to avail transportation? How do the beneficiaries commute?
4. Do the Anganwadi centers have adequate infrastructure to support all activities?
5. What are the days and timings of services provision?
6. What are the services provided for married reproductive age group women in the preconception period, pregnant women and children 0 to 24 months of age?
   [Probes:-
   Preconception women
   - Any services for married women in reproductive age group in preconception period; any routine contact with these women.
   - Are their height weight measured? Is BMI calculated? Is weight of preconception women tracked? Do you know the BMI categories/what is low and normal BMI
   - Do you know what is inadequate weight gain in preconception women, please explain
   - Are preconception women given any nutrition supplement from ICDS, if yes elaborate. what (type, amount, cooked or dry, nutrient content, any animal protein, THR and hot cooked, quality, taste, variety by day, frequency etc).
   - place of distribution etc). If women are very weak or thin (BMI <18.5kg/m2), is any additional supplement given? If yes, what? How do women access these food (AWW gives at home/a central place/women visit AWCs)
   - If weight not recorded at your center or even otherwise, how do you come to know the special nutritional needs of a preconception woman? What is the mechanism of communicating/linkage with the health department
   - Any IFA and multiple micronutrients given to women, is yes, what dose, how, when and to which women. Do you measure compliance?
   - Do you face any challenges in delivering these interventions, please elaborate.
   - What are the mitigation strategies
   - If we need to reach out to preconception women to deliver nutrition services, what are your suggestions to do so effectively?

   Pregnant women and postnatal women
   - Anthropometry: height, weight (functional equipment); is BMI calculated in first trimester and gestational weight gain tracked. Management of inadequate gestational weight gain (IGWG).

- Are you aware of the gestational weight gain/how much a pregnant woman needs to gain during each trimester of pregnancy and total weight gain
- Do pregnant and postnatal women get any SNP from your center? If yes, what (type, amount, cooked or dry, nutrient content, any animal protein, THR and hot cooked, quality, taste, variety by day, frequency etc).
- Any special food for women in any particular category: inadequate GWG, IWG, low BMI? Please describe. Are these women referred and where?
- If weight not recorded at your center or even otherwise, how do you come to know the special nutritional needs of a pregnant woman? How do you come to know or decide what kind of food and in what quantity needs to be given to women who do not have adequate GWG.
- Any IFA and multiple micronutrients given to women, is yes, what dose, how, when and to which women. Do you measure compliance?

Children 0 to 24 months

- Anthropometry measurements done for children, by whom and where? Is weight gain tracked over time and any action taken for IWG/growth faltering? Do you know what is inadequate weight gain in children, please explain
- How do you come to know that child needs special food.
- Do children get any SNP from your center? If yes, what (type, amount, cooked or dry, nutrient content, any animal protein, THR and hot cooked, quality, taste, variety by day, frequency etc). Which age group children get what?
- Is there any specific food for any particular category, such as MAM or SAM children? How is this being monitored? How to you track them? Are they further getting referred and where?
- What are the activities for young children, particularly those related to ECD? What are these activities? Are these being implemented? What are the challenges?
- If any child does not avail services, do you do anything? What
- Are mothers counselled on care of children at the AWC? If yes, please describe; early initiation of BF, EBF till 6 months, immunization, ENC (cord care, warmth, identification of danger signs and referral), KMC and expressed breast milk feeding for preterm and LBW, CF at 6 months, type, frequency, quantity, consistency, responsive feeding etc
- Are children given any micronutrient supplementation (calcium, iron, phosphorus, vitamin D)? Is any supplementation given to LBW or VLBW babies? If yes, by whom?
- Do you experience any challenges in growth monitoring? Please describe. What are the possible solutions?

Service utilization from the ICDS

[Probes:-

- Beneficiaries in each category who are in the catchment area and who are registered with the ICDS
- How many of them utilize services? How frequently? Do the AWWs maintain records/registers?
- Do the AWWs follow up those who are not registered or those who do not visit the centers?
- If low utilization is low, what do you think are the reasons? What can be the solutions?

Supervision and monitoring

7. What is the system of supervision and monitoring? What is the reporting system at AWCs, in the district and state- review meetings, frequency, content, actions taken, stakeholders attending. What are the challenges and possible solutions

Data management and data review

11. What is the system of data management and data review (electronic/manual; are data entered directly into tablets/phones or first manually entered and then entered into electronic database)?
12. How are records maintained at the AWCs? Do you have any electronic data base? What are the records that are maintained? How do you track the beneficiaries?
13. Is it possible to create electronic trackers- what are the challenges and solutions? Please describe the use of POSHAN tracker and ICDS CAS. What are the challenges and mitigation strategies?
14. Please describe the linkages with the health systems? If any ICDS beneficiary requires medical care, what do you do? Similarly, are beneficiaries referred from the health systems to ICDS for nutritional needs? Is there any challenge? What are the possible mitigation strategies?

National programs and schemes

15. Are you aware of the national programs and schemes related to nutrition in women and children? Are these being followed/implemented? Can you tell me about the activities under each program that are being implemented at your facility and in the community of the catchment area? If not, why so? What are the challenges?

    Probe if unable to respond spontaneously.
    - JSSK/JSY
    - Anemia Mukt Bharat
    - Swachh Bharat Mission Gramin
    - PMMVY
    - Jan Andolan POSHAN Abhiyan
    - National guidelines for young child feeding
    - Any state specific program
    - Operational guidelines KMC and optimal feeding of LBW infants
    - Facility based care of SAM

Training

16. What is the system of training in the ICDS? Who trains, frequency, duration, content? Is there a pool of trainers? When were you last trained and in what?

SBCC

17. What platforms are currently being used for SBCC activities? Please elaborate on the content, frequency, attendance by the community. What can be done to spread awareness in the community for any new programs?

Supplies and general functioning of AWCs

19. Are any medicines/supplements available at the centers- IFA, anthelmintic, Calcium, vit D etc?
20. Is there any issue related to supplies, stock outs? Is the SNP sufficient for all beneficiaries in the catchment area? What are the challenges in procurement or ensuring regular supplies?
21. Who prepares the food? Do you have SHGs in place? Please describe how the system functions? What is the budget allocation for SNP? Do you/SHG have food measuring scales, utensils for cooking and storing food?
22. How are the following being procurement, budget, distribution and delivery?
    - food for preconception women
    - additional food to the pregnant and lactating women and children
    - animal proteins – milk, eggs
    - for special conditions and beneficiary categories such as inadequate GWG, IWG, low BMI
23. Do the centers have functional anthropometry equipment for length/height, weight, for women and children?

Challenges in providing optimum services

24. What are the challenges in providing optimum services in the AWCs as recommended for women and young children? What according to you would be the mitigation strategies to overcome these barriers?

Food preferences

25. What are the **food preferences in your community** by women and young children? Do the women like or dislike the SNP provided through ICDS? Is there any SNP that they like or dislike? Reasons
    - What is the community perception about egg consumption? Is it accepted or are there any barriers due to cultural or religious reasons, taste issue etc.
    - Is there any particular food preference/culturally accepted by the community women (preconception, pregnant women and children)

C. FGD GUIDE WITH ASHAS

Participants' information (for each participant)
Name_______
Age ____
Facility (PHC, sub-center) with which attached______ Years of service in the health systems___
Address of facility location- area, village of the ASHA catchment area_________
Place of residence/distance from the facility/community where they work____
How do they commute to work______________

FGD guide
General information

1. What is the population covered by each one of you? Do you have an updated enumeration of the population in your catchment area? How frequently is this updated? Is there an electronic database of the total population?
2. What is the maximum distance that you need to cover in your catchment area? How do you commute within your area of work?
3. How many women and children are in your catchment area (preconception, pregnant and young children 0 to 2 years)? How frequently do you contact them and why, for what?
4. Do you know whether all ASHAs are in place or are there vacancies? What are the reasons for vacancies? How is the work managed in these pockets?
5. What is the usual distance that need to be travelled by women in your catchment area to access ANC, PNC, immunization etc.? Is there transportation available/accessible to the community, how do they commute?
6. What are the services available for married women in preconception period with one or no child? What are the points of contact with these women?
7. What are the common sources for health care seeking for women and children (govt/private; formal/informal; primary/secondary/tertiary)? What are the reasons for their preference?
8. What are your thoughts about the general perceptions of women and their family members in your catchment area? Do they have faith in the government health systems? How is their behavior with you, are they welcoming, do they have any grievances? Please elaborate with reasons?

Description of regular duties and activities related to WINGS Scale Up

9. What are your duty timings? What are your job responsibilities and from where do you operate? Describe your daily chores on a typical day (probe for activities on a typical day, during HBNC visits; frequency, timing, etc. Is any day different or same activities are conducted daily).
10. What do you do during HBNC visits? detail activities, including weighing, examination of baby and mother, counselling (on what), identification of conditions requiring referral, where do you refer; do you follow up after referral?
11. Do you face any challenges or difficulties in completing your daily work?
12. What medicines/supplies do you have in your kits? Please describe the process of replenishment, any problems/challenges related to supplies? What else do you carry during your field visit (portable anthro equipment, registers, tablets, etc.)
13. Are you involved in any of the following activities
    - Encourage preconception women to visit health or ICDS facility. If yes, what do you advise, how and where do you meet and advise women.
    - Encourage pregnant women for ANC; what all do you advise. how and where do you meet and advise women?)
    - Counsel on young child feeding (0 to 6 and 6 to 24). If yes, what counselling do you do? how and where do you meet and advise women

- Providing any medicines/supplements to women and under-two children in your community
- Ensuring compliance or observing intake of supplements or medicines
- Measuring height/length and weight of women and under-two children. Why are these measurements taken?
- Counselling on health related issues, water sanitation and hygiene, nutrition, or any mental condition (if women are depressed/sad/upset); if yes can you tell about each of these; what do you counsel on, what are the messages, where do you counsel, how frequently
- Do you conduct any meetings such as VHSNDs, community meetings; if yes where, how frequently, what do you discuss in these meetings

Records, documentation, reporting, supervision and training

14. Can you show me how you maintain your records? Are you using the ANMOL or any other app? Tell me about your experiences of using this. How do you track pregnant women and children for their scheduled visits? How do you ensure that pregnant women come for all ANC visits, comply with the recommended treatment and care?
15. What is the system of supervision, reporting, review meetings (frequency, content, actions taken, stakeholders attending, etc.).
16. When did you last receive any training? Who trained, frequency, duration, content? Do you feel the need for additional training, if yes in what?

Referral

17. Tell me about the referral mechanisms- what are the conditions when you refer? What is the arrangement for referral transportation? How far is the nearest referral facility- private and public? What is preferred by the community for women and children? What are the challenges around referral and solutions

Challenges and mitigation strategies

18. What are the challenges that you encounter in your work? What are possible solutions? What are the facilitators?
19. What do you think will be needed to reach out to preconception women to deliver health, nutrition services, WaSH and psychosocial care, what are your suggestions to do so effectively?
20. If you are told to make more frequent visits to some women who are non-compliant for any reason, or children, what are the challenges that you anticipate; for e.g., women and children with IWG, anemic, etc. What can be the possible solutions?

Knowledge, perceptions

21. Are you aware of the health conditions or nutritional status when a preconception woman needs to visit health facility? Are any investigations required for these women? Do they need any supplements (nutrition or medicines)? If yes, what?
22. Do you know what is high risk pregnancy? If yes, tell me the HRP conditions and what needs to be done
23. Are you aware of the health conditions or nutritional status when an under-two child needs to visit health facility? What are the danger signs that need referral
24. Are you aware of the BMI categories? Please explain
25. Are you aware of the gestational weight gain/how much a pregnant woman needs to gain during each trimester of pregnancy and total weight gain
26. Do you know what is inadequate weight gain in preconception women and children, please explain
27. Are you aware of the schemes and national programs related to women and children being implemented in your area? Please tell me about these; what are the challenges in implementing these and possible mitigation strategies?
    [Probes:-

- Family planning services
- Any services for women in preconception period
- ANC and management of morbidities during pregnancy (hypertension, GDM, thyroid disorders, anaemia, etc.)
- HBNC
- Immunization
- Mission Parivar Vikas
- Anemia Mukt Bharat
- Adolescent health program
- Swachh Bharat Mission Gramin
- SUMAN
- PMMVY
- PMSMA
- MAA
- HBYC
- Operational guidelines KMC and optimal feeding of LBW infants

28. Are you aware on Supplementary Nutrition Program (SNP) and Foods provided through the SNP for preconception women and pregnant women

29. Food liked or disliked by the target group provided through SNP, Reasons
30. Community perception on egg consumption (not accepted due to cultural or religion, taste issue etc)
31. Any particular food preference/culturally accepted by the community women (preconception and pregnant women)

D. FGD GUIDE WITH AWWS

Participants' information (for each participant)
Name_______
Age ____
Facility (AWC) with which attached______Years of service in the ICDS___
Address of AWC- area, village of the AWW catchment area_________
Place of residence/distance from the AWC/community where they work____
How do they commute to work______________

FGD guide
General information

1. What is the catchment area population covered by your Anganwadi centre? Are there any pockets without AWWs? Please provide details. How are these pockets managed/covered by ICDS?
2. Is the complete population under each AWC enumerated? How is the database maintained, updated and accessed? Is there an electronic database of the total population under each AWC? If database is in place, do you have these by age specific and other categories, for example all adolescent girls and boys, eligible couples, married women in reproductive age group with one or no child, pregnant women, 0 to 6 months, >= 6 months to 2 years?
3. What is the maximum distance of the AWC from the farthest point of its catchment area? Is it within walking distance or a need to transportation? How do they commute?
4. Do the anganwadi centers have adequate infrastructure to support all activities?
5. Please describe the linkages with the health systems? If any ICDS beneficiary requires medical care, what do you do? Similarly, are beneficiaries referred from the health systems to ICDS for nutritional needs? Is there any challenge? What are the possible mitigation strategies?
6. What are your thoughts about the general perceptions of women and their family members in your catchment area? Do they have faith in the government health systems? How is their behavior with you, are they welcoming, do they have any grievances? Please elaborate with reasons.
7. **Description of regular duties and activities related to WINGS Scale Up**
   - What are the days and timings of services provision? What are your duty timings? What are your job responsibilities? Describe your daily chores on a typical day.
   - Do you face any challenges or difficulties in completing your daily work? What is the maximum distance that you need to cover in your catchment area? How do you commute?
8. Are you involved in any of the following activities
   - Encourage preconception women to visit health or ICDS facility. If yes, what do you advise, how and where do you meet and advise women.
   - Encourage pregnant women for ANC; what all do you advise. how and where do you meet and advise women?)
   - Counsel on young child feeding (0 to 6 and 6 to 24). If yes, what counselling do you do? how and where do you meet and advise women
   - Providing any medicines/supplements to women and under-two children in your community
   - Ensuring compliance or observing intake of supplements or medicines
   - Measuring height/length and weight of women and under-two children. Why are these measurements taken?
   - Counselling on nutrition related issues, water sanitation and hygiene, health, or any mental condition (if women are depressed/sad/upset); if yes can you tell about each of these; what do you counsel on, what are the messages, where do you counsel, how frequently

- Do you conduct any meetings such as VHSNDs, community meetings; if yes where, how frequently, what do you discuss in these meetings

9. What are the services provided for married reproductive age group women in the preconception period, pregnant women and children 0 to 6 months of age?

   [Probes:-

   - Any services for married women in preconception period with no or one child, in reproductive age group; pregnant women and children; any routine contact with preconception women. Do they visit the AWCs? If yes, for which services? If not, why do you think they do not come? What could be the possible contacts of these women with ICDS. According to you what would it take to provide services to these women and what are your suggestions to do so effectively?
   - SNP for adolescent girls, preconception women, pregnant women and children.
   - What are the food that are provided as SNP- please provide details (type, amount, cooked or dry, nutrient content, any animal protein, THR and hot cooked, quality, taste, variety by day, any other.]
   - Does the SNP vary by age group? (6-12 months, 12-24 months). Why do you say so?
   - Is there any specific food for any particular category, such as MAM or SAM children? Any special food for women in any particular category.. IWG, low BMI or inadequate GWG? Please describe. Definition of BMI, IWG
   - Is there any issue related to supplies, stock outs? Is the SNP sufficient for all beneficiaries in the catchment area? What are the challenges in procurement or ensuring regular supplies?
   - Who prepares the food? Do you have a SHG? Please describe how the system functions? What is the budget allocation for SNP?
   - What are the activities for young children and those related to ECD? Are these being implemented? What are the challenges?

Service utilization from the ICDS

10. Please tell me about the service utilization from the ICDS

    [Probes:-

    - Beneficiaries in each category who are in the catchment area and who are registered with the ICDS, including adolescent girls and preconception women.
    - How many of them utilize services? How frequently? Do the AWWs maintain records/registers?
    - Do the AWWs follow up those who are not registered or those who do not visit the centers?
    - If low utilization, what do you think are the reasons?
    - Are any medicines available at the centers- IFA, anthelmintic, Calcium, vit D etc?
    - Do the centers have anthropometry equipment for height, weight, MUAC for women and children? Is the growth of children monitored? Please explain how this is done? What are the actions taken if there is growth faltering? Do you experience any challenges in growth monitoring? Please describe. What are the possible solutions?

Records, documentation, reporting, supervision and training

11. What is the system of supervision and monitoring? What is the reporting system at AWCs, in the district and state- review meetings, frequency, content, actions taken, stakeholders attending. Are these useful, can these be made more effective?
12. What is the system of data management and data review? How are records maintained at the AWCs? How do you track the beneficiaries? Please describe the utilization of POSHAN tracker and ICDS CAS. What are the challenges in using these and possible mitigation strategies?
13. What is the system of training in the ICDS? Who trains, frequency, duration, content? Is there a pool of trainers? When were the AWW last trained? Have they received training on the 17 modules? Do you feel the need for additional training, if yes in what?

SBCC

14. What are the platforms currently being used for SBCC activities? Please elaborate on the content, frequency, attendance by the community? What can be done to spread awareness in the community for any new programs?

Referral

15. Tell me about the referral mechanisms- what are the conditions when you refer? What is the arrangement for referral transportation? How far is the nearest referral facility- private and public? What is preferred by the community for women and children?

Challenges and mitigation strategies

16. What are the challenges that you encounter in providing optimum services in the AWCs as recommended for women and young children? What according to you would be the mitigation strategies to overcome these barriers? What are the facilitators?
17. What do you think will be needed to reach out to preconception women to deliver health, nutrition services, WaSH and psychosocial care, what are your suggestions to do so effectively?
18. If you are told to make more frequent visits to some women who are non-compliant for any reason, or children, what are the challenges that you anticipate; for e.g., women and children with IWG, anemic, etc. What can be the possible solutions?

Knowledge, perceptions

19. Are you aware of the health conditions or nutritional status when a preconception woman needs to visit health facility? Are any investigations required for these women? Do they need any supplements (nutrition or medicines)? If yes, what?
20. Do you know what is high risk pregnancy? If yes, tell me the HRP conditions and what needs to be done
21. Are you aware of the health conditions or nutritional status when an under-two child needs to visit health facility? What are the danger signs that need referral
22. Are you aware of the BMI categories? Please explain
23. Are you aware of the gestational weight gain/how much a pregnant woman needs to gain during each trimester of pregnancy and total weight gain
24. Do you know what is inadequate weight gain in preconception women and children, please explain
25. IFA and Micronutrient Supplementation: are you aware of the dosage, frequency, and method of delivery (e.g., directly observed consumption) and compliance to supplementation intake monitored
26. Are you aware of the national programs and schemes related to nutrition in women and children? Are these being followed/implemented? Can you tell me about the activities under each program that are being implemented at your facility and in the community of the catchment area? If not, why so? What are the challenges?
    [Probe in unable to respond spontaneously.
    - JSSK/JSY
    - Anemia Mukt Bharat
    - Swachh Bharat Mission Gramin
    - PMMVY
    - Jan Andolan POSHAN Abhiyan
    - National guidelines for young child feeding
    - Any state specific program
    - Operational guidelines KMC and optimal feeding of LBW infants
    - Facility based care of SAM
27. Are you aware of the food liked or disliked by the target group provided through SNP, Reasons

28. Community perception on egg consumption (not accepted due to cultural or religion, taste issue etc)
29. Any particular food preference/culturally accepted by the community women (preconception and pregnant women)

E. (1, 2 and 3) FGD GUIDE WITH MARRIED WOMEN IN PRECONCEPTION PERIOD, PREGNANT WOMEN AND WOMEN WITH CHILDREN AGED BETWEEN 1 TO 2 YEARS (groups to be homogeneous).

Participants' information (for each participant)
Name________ Age ____ Number of children with age_____
Fertility intentions_____Currently pregnant, gestational age______
Place of residence____Type of family- joint/nuclear__ Own or rented house_______
Total number of family members in the household__
Main occupation of the earning members___
Average monthly income___

FGD guide
PRECONCEPTION WOMEN (E.1)

1. What are the conditions for which you seek care for yourself, the facilities from where you seek care and reasons for your preference.
2. What is the distance that you need to travel? How do you commute?
3. Are you called for any routine services at health facilities? What are these? Which are the facilities where you are called? Describe- frequency, content, activities, etc. Do you comply with the advice to visit facilities?
4. Does any health worker or Anganwadi worker visit your home? If yes, who, for what, how frequently?
5. What are the services that are available or services that you utilize, at the government health centers.
   [Probes:-
   a. Family planning services- contraceptives offered. If any woman in the community is weak, anemic or has any illness, are these women advised to delay pregnancy till their health improves? Are they counselled to use contraceptive device to delay pregnancy or to maintain at least 2 years interval between 2 pregnancies? Types of contraceptives [these will be probes and not asked as leading questions]
   b. Health conditions- screening and management of anaemia, thyroid problems, RTI, TB, high blood pressure and diabetes (use local terms) [; identification, lab investigations, management, prevention, treatment, referral, follow up
   c. Was any laboratory tests done? If yes, for what, when and from where? Reasons for choosing this place.
   d. Measurement of weight and height? If yes, when (how frequently) and where were these measurements taken? Were you told whether you are weak (about BMI) and you need to take care of your diet (Check for any documentation/card, where height/weight/BMI are recorded.)
   e. Did you receive any counselling on diet, hand hygiene, menstrual hygiene and personal hygiene, safe drinking water, clean toilets etc. From whom, where, at what frequency? What do you use as menstrual adsorbents?
   f. Were you screened for mental and psychological conditions? Did you receive any counselling in case you feel stressed, sad, upset, due to any reason (probe carefully on sensitive issues) such as alcoholism/domestic violence/financial hardships etc. (psychosocial). Who counselled and where?
   g. Any medicines or supplements such as IFA, Multiple micronutrients,or if you were diagnosed with any health issues, did you receive any other medicines for treatment of the condition? From where did you receive and how? Did you have to buy these?
6. What are the services that are available or services that you utilize, at the anganwadi centers.

a) Do you visit the Anganwadi centers? Are you given any nutrition supplement from ICDS, if yes elaborate (type, amount, frequency, place of distribution etc). Probe for milk and eggs. If you were weak (as told to you based on your weight and height, was any additional food supplement given? If yes, what?
b) How do women access these food (AWW gives at home/a central place/women visit AWCs)
c) Any particular food preference/culturally accepted by the community women. Community perception on egg consumption (not accepted due to cultural or religion, taste issue etc)

7. Does any health care provider or AWW visit you at your home? If yes, who are they, frequency, and reason for visits, what are the activities at the visits?
8. Do you face any challenges in availing the services from the government health facilities or the Anaganwadi centers? Please elaborate. What could be the possible solutions/mitigation strategies?
9. Have you ever been referred for any condition? Where and why were you referred? Did you comply with referral advice? Why/why not? What is the transport system for referral? What was your experience at the referral facility?
10. What is the source of drinking water? What is the water quality? Do you use any water purification system at home (filter/RO/chlorine tabs etc). How do you store drinking water and how do you dispense it? Is the quantity of water sufficient for the family?
11. What is the place of defecation for adults and children- probe for open defecation and disposal of feces.
12. Do you use any disinfectants to mop the floor- what do you use, how do you use and how frequently? Is this always available at home? If you are not using anything, how are you mopping the floor, do you use any detergent- regularly/sometimes/rarely?
13. What do you use to clean latrines, how do you use and how frequently? Is this always available at home? If you are not using anything, how are you cleaning latrine, do you use any detergent- regularly/sometimes/rarely?
14. What hand hygiene do you follow? What do you use to wash hands- is this always available at home? Do you have source of running water/hand washing basin?
15. Do you have a BPL card? What are the benefits that you get?
16. Do you have PDS in your locality? What all is distributed through PDS? Are you entitled to PDS? What is the criteria for entitlement?
17. Have you been exposed to any SBCC activity in your locality. Please describe…place, messages, frequency, by whom, utility etc.
18. Are you aware of the schemes that you are entitled to- JSSK, JSY, government insurance schemes etc. (Is it common for women to go to their mother's house after giving birth, and if so, when do they usually go? What happens to postpartum follow-up visits by health workers if a woman goes to her mother's house?

PREGNANT WOMEN (PREFERABLY IN THE 3RD TRIMESTER) (E.2)

1. Months of gestation/LMP
2. From where are you getting your ANC checkup done? What is the reason for selecting this place. Tell me about your ANC visit experience- month of first registration, number of ANC visits you had and in which month of pregnancy, frequency, timing of ANC visits, physical examination, measurement of height, weight, BP, per abdomen examination, lab investigations (blood tests- check records; urine test), USG, immunization, any medicines given, for what, any counselling done, past obstetric history, others. Is everything available at the same center or you are referred elsewhere for USG/investigations etc. What is the distance that you need to travel? How do you commute?

3. If diagnosed with any morbidity, how were you managed? Did they refer? If yes under what condition and where?
4. Did you receive any card [check the MCP cards for entry of records, completion, tracking across all ANC visits esp for Hb, BP, weight, missing information]
5. Measurement of weight and height? If yes, when (how frequently) and where were these measurements taken? Is weight gain tracked over time and any action taken for IGWG? Were you told whether you are weak (about BMI) and you need to take care of your diet (Check for any documentation/card, where height/weight/GWG are recorded.)
6. Did you face any challenges in availing ANC services from the facility? What are these? What are the possible solutions/mitigation strategies? In case you are told to visit facility for 8 ANC check-up throughout your pregnancy (first visit ideally occurring in the first trimester, up to 12 weeks, followed by visits at 20, 26, 30, 34, 36, 38, and 40 weeks of gestation, what are your thoughts regarding this?
7. Did any health care provider or AWW visit you at your home? If yes, who are they, frequency of visit, and reason for visits, what were the activities at the visits?
8. Were you told that you have any high risk condition in your pregnancy? If yes, what was the condition? Did you receive any special care for this condition or did you do anything additional to take care of this condition?
9. Did you receive any counselling on diet, hand hygiene, personal hygiene, safe drinking water, clean toilets etc. From whom, where, at what frequency?
10. Did you receive any counselling in case you feel stressed, sad, upset, due to any reason (probe carefully on sensitive issues) such as alcoholism/domestic violence/financial hardships etc. (psychosocial). Who counselled and where?
11. Where did you deliver or plan to deliver? Any specific reasons for this preference?
12. What are the services that are available or services that you utilize, at the anganwadi centers.
    d) Do you visit the Anganwadi centers? Are you given any nutrition supplement from ICDS, if yes elaborate (type, hot cooked or take-home ration, amount, frequency, place of distribution etc). Probe for milk and eggs.
    e) Tell me about the food that is distributed at the AWC (type, variety, quality, taste, amount, hygiene, others).
    f) If you were weak (as told to you based on your weight and height, and your weight gain during pregnancy, was any additional food supplement given? If yes, what?
    g) How do women access these food (AWW gives at home/a central place/women visit AWCs)
    h) Any particular food preference/culturally accepted by the community women. Community perception on egg consumption (not accepted due to cultural or religion, taste issue etc)
    i) Any counselling done at the AWCs- during pregnancy, early initiation of BF and EBF
13. Are you getting any medicines/supplements? Probe for IFA, multiple micronutrients, or if you were diagnosed with any health issues, did you receive any other medicines for treatment of the condition? From where did you receive and how? Did you have to buy these?
14. Have you ever been referred for any condition? Where and why were you referred? Did you comply with referral advice? Why/why not? What is the transport system for referral? What was your experience at the referral facility?
15. Did you face any challenges in availing of the services from the government health facilities or the Anganwadi centers? Please elaborate. What could be the possible solutions/mitigation strategies?
16. What is the source of drinking water? What is the water quality? Do you use any water purification system at home (filter/RO/chlorine tabs etc). How do you store drinking water and how do you dispense it? Is the quantity of water sufficient for the family?

17. What is the place of defecation for adults and children- probe for open defecation and disposal of feces.
18. Do you use any disinfectants to mop the floor- what do you use, how do you use and how frequently? Is this always available at home? If you are not using anything, how are you mopping the floor, do you use any detergent- regularly/sometimes/rarely?
19. What do you use to clean latrines, how do you use and how frequently? Is this always available at home? If you are not using anything, how are you cleaning latrine, do you use any detergent- regularly/sometimes/rarely?
20. What hand hygiene do you follow? What do you use to wash hands- is this always available at home? Do you have source of running water/hand washing basin?
21. Do you have a BPL card? What are the benefits that you get?
22. Do you have PDS in your locality? What all is distributed through PDS? Are you entitled to PDS? What is the criteria for entitlement?
23. Have you been exposed to any SBCC activity in your locality. Please describe…place, messages, frequency, by whom, utility etc.
24. Are you aware of the schemes that you are entitled to- JSSK, JSY, government insurance schemes etc.

CHILDREN 0 TO 24 MONTHS (AND WOMEN WITH CHILDREN AGED 1 TO 2 YEARS). [E-3]

25. For women who delivered in the last 6 months: Tell me about your experience in the postnatal period- birth weight of baby, gestation age at birth, lactation support in the facility, KMC if baby born small (preterm/LBW); home visits in the postnatal period (time points, frequency, content, activities)
26. For women with children less than 2 years of age
    a. Are children screened for illnesses and danger signs that require immediate medical attention? If yes, how, by whom, how frequently?
    b. Height, weight measurements done for children, by whom and where, how frequently? Is weight gain tracked over time and any action taken for IWG?
    c. Were you counselled on care of children such as early initiation of BF, EBF till 6 months, immunizations, ENC (cord care, warmth, identification of danger signs and referral), KMC and expressed breast milk feeding for preterm and LBW, CF at 6 months, type, frequency, quantity, consistency, responsive feeding, micronutrient supplementation (calcium, iron, phosphorus, vitamin D) supplementation given to LBW or VLBW babies (If yes, by whom) etc
    d. Did anyone talk to you about promotion early child play, responsive care, identification of delayed development and timely referral
    e. Was any counselling done for WaSH; what messages were given, by whom, when and how
27. Do you visit the Anganwadi centers? Are you given any nutrition supplement from ICDS, if yes elaborate (type, hot cooked or take-home ration, amount, frequency, place of distribution etc). Probe for milk and eggs.
28. Tell me about the food that is distributed at the AWC (type, variety, quality, taste, amount, hygiene, others).
    a. If your child was weak- (IWG, MAM, SAM, or as told to you based on his/her weight and height, and weight gain, was any additional food supplement given? If yes, what?
    b. How do you access these food (AWW gives at home/a central place/women visit AWCs)
    c. Any particular food preference/culturally accepted by the community women. Community perception on egg consumption (not accepted due to cultural or religion, taste issue etc)
    d. Any counselling done at the AWCs- during pregnancy, early initiation of BF and EBF

29. What are the other services that are available or services that you utilize, at the Anganwadi centers.
    a) Growth monitoring for children
    b) Play activities for young children or mothers taught ECD activities?
    c) Any other
30. Have your child ever been referred for any condition? Where and why were you referred? Did you comply with referral advice? Why/why not? What is the transport system for referral? What was your experience at the referral facility?
31. Did you face any challenges in availing the services from the government health facilities or the Anganwadi centers? Please elaborate. What could be the possible solutions/mitigation strategies?
32. What is the source of drinking water? What is the water quality? Do you use any water purification system at home (filter/RO/chlorine tabs etc). How do you store drinking water and how do you dispense it? Is the quantity of water sufficient for the family?
33. What is the place of defecation for children- probe for open defecation and disposal of feces.
34. Do you have a clean play area for children
35. Do you use any disinfectants to mop the floor- what do you use, how do you use and how frequently? Is this always available at home? If you are not using anything, how are you mopping the floor, do you use any detergent- regularly/sometimes/rarely?
36. What do you use to clean latrines, how do you use and how frequently? Is this always available at home? If you are not using anything, how are you cleaning latrine, do you use any detergent- regularly/sometimes/rarely?
37. What hand hygiene do you follow? What do you use to wash hands- is this always available at home? Do you have source of running water/hand washing basin?
38. Do you have a BPL card? What are the benefits that you get?
39. Do you have PDS in your locality? What all is distributed through PDS? Are you entitled to PDS? What is the criteria for entitlement?
40. Have you been exposed to any SBCC activity in your locality. Please describe…place, messages, frequency, by whom, utility etc.
41. Are you aware of the schemes that you are entitled to- JSSK, JSY, government insurance schemes etc.

F. IDI GUIDE WITH FAMILY MEMBERS AND KEY INFORMANTS

Participant information
Participant information
Name _______ Age ____ Sex ____
Place of residence____ Resident of the area since ____ years
Occupation ___
Is there any woman of reproductive age group or children less than 2 years of age in the HH? _____

Interview guide

1. What are the government and private health facilities catering to women and young children, in your area? Are you aware of the services provided at these facilities?
2. Do you think health of a married non-pregnant woman is important? Why do you say so? What are the sources of care seeking for such women and what are the conditions that required care seeking?
3. Are you aware of any services that are available for such women? Where do women in the community prefer to seek care from? Why do you say so? What are the challenges or gaps in the existing sources? Possible solutions
4. Describe the private facilities; whether they belong to the informal sector or qualified doctors, quality of services. For any specific services provided by the private facilities, is the utilization higher compared to other services? If yes, elaborate.
5. What are the referral facilities? When/for what conditions are women and children referred? What is the compliance with referral? Do you see any barriers/challenges?
6. What about the transport facilities in your community- public, private; how do people commute?
7. Have you heard of the ICDS/Anganwadis? Do you know of the services provided there? Do beneficiaries utilize these? Why do you say so?
8. Do health workers or AWWs visit homes to provide services? Please describe.
9. What are your thoughts regarding the services at the government health facilities and the Anganwadi centers? Please describe the barriers to utilization and how these can be overcome- mitigation strategies.
   [probe regarding lab investigations, USG, medicines, HR, skills and counselling, quality of services, supplementary nutrition, transportation, access, etc.]
10. What is the system of PDS in your community? Please describe- who are entitled, what products are available through PDS, what is the utilization, the barriers to utilization, etc.]
11. What are the sources of drinking water? [Probe: availability, quantity, quality of drinking water, any purification done at source, at point of use, how water is collected, stored and dispensed, etc.]
12. What are the common places of defecation for adults and young children? [Probe: disposal of feces of young children, disinfectants used at home for latrines and floor, is it similar across all HHs or there are HHs that do not used disinfectants, reasons for not using, availability/affordability, etc.]
13. Is there awareness about appropriate hand washing [Probe; handwashing station, flowing water, use of soaps/handwash, availability/affordability, etc]
14. Is there any government health insurance that you are aware of [Probe: heard of PMJAY, JSY, JSSK etc.; utilization of these schemes, barriers to use; etc]
15. What are the activities/platform to inform the community or create awareness for any new or existing government program [probe: any fixed days, the strategies such as announcements on loudspeakers, posters/pamphlets/hoardings, wall paintings, community meetings-venue, frequency, content, who conducts, attendance, etc]. Any new ideas of creating awareness and generating demand for services related to pre-conception and pregnant women and young children.]

16. What are the possible strategies through which all preconception and pregnant women and young children in the community can be reached and covered effectively under the government program targeting them? What according to you can be the solutions to the challenges you have mentioned?
17. Food preferences of the community Any particular food preference/culturally accepted by the community women. Community perception on egg consumption (not accepted due to cultural or religion, taste issue etc) . Perception about SNP provided at the AWCs
