# Supplementary material for: Implementation research to scale up the women and infants integrated interventions for growth study (WINGS) in Himachal Pradesh: Protocol for a quasi-experimental, mixed-methods study
Source: PLoS One. 2026 Feb 17;21(2):e0341048. doi: 10.1371/journal.pone.0341048 (PMC12912596; doi:10.1371/journal.pone.0341048)
Supplement: S4 File — (PDF) [file pone.0341048.s005.pdf]

Anganwadi Cost Form

Field	Question	Answer	
worker *(required)*	Field Investigator	151	Abuhamza
		152	Anmol Saini
		153	Anshika Sahota
		154	Ekta
		155	Jyoti Devi
		156	Kritika Thakur
		157	Mehak Thakur
		158	Poonam Devi
		159	Riya Puri
		160	Shivanshi
		161	Varsha Kumari
		162	Anchal Walia
		163	Harshali
		164	Kritika Puri
blocks *(required)*	Block Names	block_1	Amb
		block_2	Thanakalan
		block_3	Gagret
		block_4	Haroli
		block_5	Basdehra
circle_selected *(required)*	Circle of area	circle_1	Amb
		circle_2	Bhaira
		circle_3	Chaksarai
		circle_4	Chintpurni
		circle_5	Chururu
		circle_6	Jubehar
		circle_7	Kalruhi
		circle_8	Kharoh
		circle_9	Nandpur
		circle_10	Nehrian
		circle_11	Sapouri
		circle_12	Sidhchalehar
		circle_13	Chowki
		circle_14	Dhundla
		circle_15	Jasana
		circle_16	Jol
		circle_17	Khurwain
		circle_18	Lathiani
		circle_19	Piploo
		circle_20	Raipur
		circle_21	Thanaklan
		circle_22	Ambota
		circle_23	Badeda Rajputan
		circle_24	Bhaderkali
		circle_25	Bhanjal
		circle_26	Gagret
		circle_27	Ganu Madwada
		circle_28	Ghanari
		circle_29	Mawa Kaholan
		circle_30	Mawa Sindhia
		circle_31	Pirtipur
		circle_32	Bathri
		circle_33	Dulehar
		circle_34	Ghaluwal
		circle_35	Haroli
		circle_36	Ispur
		circle_37	Kanger
		circle_38	Kungrat
		circle_39	Lalri
		circle_40	Palakwah

Field	Question	Answer
		circle_41 Panjawar<br>circle_42 Pubowal<br>circle_43 Santoshgarh<br>circle_44 Tahliwal<br>circle_45 Abada Barana<br>circle_46 Babarudru<br>circle_47 Bahdala<br>circle_48 Barnoh<br>circle_49 Basal<br>circle_50 Bhatoli<br>circle_51 Dehlan<br>circle_52 Fatehpur<br>circle_53 Ghandawal<br>circle_54 Jhalera<br>circle_55 Lower Arniala<br>circle_56 Raipur<br>circle_57 Rakkad<br>circle_58 Sanoli<br>circle_59 Una
awc_center *(required)*	Anganwadi Center	1 Amb-I<br>2 Amb-II<br>3 Amb-III<br>4 Amb-IV<br>5 Amb-V<br>6 Sham Nagar-I<br>7 Sham Nagar-II<br>8 Partap Nagar-I<br>9 Partap Nagar-II<br>10 Hira Nagar-I<br>11 Hira Nagar-II<br>12 Adarash Nagar<br>13 Andoura Upper-I<br>14 Andoura Upper-II<br>15 Andoura Upper-III<br>16 Andoura Upper Middle<br>17 Andora Gujjar Basti<br>18 Andora Lower-II<br>19 Andora Lower-III<br>20 Kuthera Kherla<br>21 Kuthera Kherla-IV<br>22 Bhaira<br>23 Bhaira-II<br>24 Bhaira Upper<br>25 Bhaira Middle<br>26 Bhaira Lower<br>27 Dhusara<br>28 Dhusara Abble<br>29 Dhusara Doam-I<br>30 Dhusara Doam-II<br>31 Saluri<br>32 Diara<br>33 Diara-II<br>34 Diara-III<br>35 Dilwan<br>36 Dilwan-II<br>37 Chhambah<br>38 Satother<br>39 Satother-II<br>40 Satother Teli Muhalla<br>41 Gijjar Cho<br>42 Chaksrai

Field	Question	Answer		
			43	Gathroon
			44	Jhager
			45	Ripoh Misran
			46	Ripoh Muchlian-II
			47	Danguhi
			48	Jhamber
			49	Karap Kotla
			50	Tiai
			51	Baroh
			52	Polian Parohitan
			53	Kuthera Kherla-II
			54	Kuthera Kherla-III
			55	Kherla-I
			56	Kherla-II
			57	Paloh
			58	Bharobadsar
			59	Lander Tikkari
			60	Majhar
			61	Dhar Gujaran
			62	Nari
			63	Nari 2
			64	Nari Harijan Basti
			65	Nari Chobe Basti
			66	Badhmana
			67	Badhmana-II
			68	Badhmana Harijan Basti
			69	Jawal
			70	Jawal-II
			71	Jawal Chang Basti
			72	Chhaproh
			73	Chhaproh 2
			74	Rehi
			75	Duhal Bhatwala
			76	Mirgu (Amokla Pritam)
			77	Dhalwari
			78	Dhalwari Dehlwan
			79	Chalol Behar
			80	Duhal Bangwala
			81	Paplehra
			82	Chururu-I
			83	Chururu-II
			84	Chururu-III
			85	Chururu-IV
			86	Hamboli
			87	Upper Hamboli
			88	Bandukian Da Behra
			89	Baheri
			90	Singhan Da Behra
			91	Seri-I
			92	Seri-ii
			93	Dhandri-I
			94	Dhandri-II
			95	Shiv Nagar-I
			96	Shiv Nagar-II
			97	Thathal-III
			98	Thathal-IV
			99	Thathal-V
			100	Thathal Ram nagar
			101	Kathiari Ward No. 3
			102	Jubehar
			103	Saroi

Field	Question	Answer		
			104	Jandoh
			105	Lander Landian
			106	Suri-I
			107	Suri-II
			108	Suri-III
			109	Behar Jaswan
			110	Ladial Chuk
			111	Bagru
			112	Akrot
			113	Chak
			114	Chak Bella
			115	Takarla Lower
			116	Takarla-I
			117	Takarla-II
			118	Takarla-III
			119	Gondpur
			120	Thathal Nakki
			121	Lohara Lower
			122	Bhagra
			123	Tikkari-I
			124	Tikkari-II
			125	Mubarikpur
			126	Ghebat behar
			127	Shivpur
			128	Kashipur-I
			129	Saloi
			130	Pramb
			131	Alehar
			132	Karluhi-I
			133	Karluhi-II
			134	Athwan-I
			135	Athwan-II
			136	Jhangoli
			137	Mandholi
			138	Bringal
			139	Mather
			140	Channi Devi
			141	Takoli
			142	Chowar-I
			143	Chowar-2
			144	Kwah
			145	Ghangret-I
			146	Ghangret-II
			147	Ghangret-III
			148	Gindpur
			149	Maloun-I
			150	Maloun-II
			151	Kharoh-I
			152	Kharoh-II
			153	Behar Bhater
			154	Bhater
			155	Behar-I
			156	Behar-II
			157	Baret
			158	Chanourian
			159	Harijan Basti
			160	Dharamshala Mahanta-I
			161	Dharamshala Mahanta-II
			162	Bharar Bar
			163	Badsla Basti
			164	Baba Nakodar Dass

Field	Question	Answer		
			165	Nandpur-I
			166	Nandpur-II
			167	Gadiale
			168	Muhalla Lambran
			169	Thathal-I
			170	Thathal-II
			171	Kathiari
			172	Kathiari Harijan Basti
			173	Thakur Dwara
			174	Kathiari par Bela
			175	Katohar Kalan-I
			176	Katohar Kalan-II
			177	Talwal
			178	Bijapur
			179	Katohar Khurd-I
			180	Katohar Khurd-II
			181	Pucca Paroh
			182	Andora Lower-IV
			183	Badaun-I
			184	Badaun-II
			185	Bajigar Muhalla
			186	Andora lower
			187	Nehari Nauranga
			188	Nehari Khas
			189	Santo Tilla
			190	Bagga Brota
			191	Karar Behar
			192	Duhki
			193	Januhi
			194	Mairi Khas
			195	Mairi-II
			196	Mairi-III
			197	Gawalsar
			198	Panjoa Khurd
			199	Panjoa kalan
			200	Ladoli-I
			201	Thara-I
			202	Thara-II
			203	Thara-III
			204	Kangruhi
			205	Naloh Miyor
			206	Lower Poliyan Purohitan
			207	Spouri
			208	Gangoti
			209	Mughal
			210	Nouhan
			211	Amb Tilla
			212	Band Bakhshi
			213	Kaniari
			214	Ardoh
			215	Rajpur Jaswan
			216	Jaman Kuwali
			217	Maslana
			218	Guliar
			219	Kohar Chhan
			220	Basantpur
			221	Lamba sail
			222	Lahar
			223	Jawar-I
			224	Jawar-II
			225	Jawar Harijan Basti

Field	Question	Answer		
			226	Patehar
			227	Lohara Upper
			228	Kotli
			229	Guret
			230	Aranwal Chahbag
			231	Thanikpur
			232	Sarda
			233	Aloh
			234	Mawa
			235	Jholan Bhatolan
			236	Chhaprohian
			237	Kashipur-II
			238	Behar
			239	Sidh Chaler
			240	Suhin
			241	Khariali
			242	Jandour-I
			243	Jandour-II
			244	Rampur-I
			245	Rampur-II
			246	Bane Di Hatti
			247	Pinjore-I
			248	Pinjore-II
			249	Chowki -1
			250	Chowki -2
			251	Chowki-4
			252	Chowki -5
			253	Beerian-1
			254	Beerian -2
			255	Beerian -3
			256	Ladila
			257	Bhaloun
			258	Bharmar
			259	Baduha -1
			260	Baduha -2
			261	Baldoh
			262	Kuder
			263	Sohari
			264	Chauli
			265	Baderah
			266	Basapatti
			267	Bhindla
			268	Baduhi-I
			269	Baduhi-Ii
			270	Baduhi-Iii
			271	Pallian
			272	Panjoda
			273	Chowki-Iii
			274	Kachhyari
			275	Amrera
			276	Sasoli
			277	Malanger
			278	Naroonh
			279	Nanawin-I
			280	Nanawin-Ii
			281	Dhundla-1
			282	Dhundla-Ii
			283	Dhundla-Iii
			284	Dhatol-I
			285	Dhatol-Ii
			286	Kusan Ranauta

Field	Question	Answer		
			287	Beri Hatli
			288	Beri-Ii
			289	Kotla
			290	Dohgi
			291	Upper Dohgi
			292	Bhugdiyan
			293	Baut
			294	Bangana
			295	Upper Nayali
			296	Bhaleti
			297	Muchhali
			298	Chilli
			299	Jakhola
			300	Jandoor
			301	Hatli
			302	Rivar
			303	Danoh
			304	Hathloun
			305	Aisan
			306	Samlara
			307	Lakhroon
			308	Majhiani
			309	Arloo
			310	Arloo Gurmukh
			311	Karor
			312	Arloo Khas
			313	Bharmot
			314	Karmali
			315	Nahri
			316	Baggi
			317	Dagru
			318	Khadol
			319	Sai
			320	Charara
			321	Badoa
			322	Raonkhar
			323	Vahi
			324	Talmera
			325	Deehar-1
			326	Deehar-2
			327	Rajpura
			328	Chadoli
			329	Buhana
			330	Chaplah
			331	Thathoon
			332	Talmet
			333	Nalwari
			334	Dumkhar
			335	Takoli-1
			336	Takoli-2
			337	Behla
			338	Jol
			339	Baslehar
			340	Harsa Jandora
			341	Bagnal
			342	Amroh
			343	Dhroon
			344	Ghaneti Mini Awc
			345	Khurwin
			346	Samoor Khurd
			347	Boul

Field	Question	Answer		
			348	Boul Har
			349	Jogi Panga
			350	Moh Khass
			351	Tyar-1
			352	Tyar-2
			353	Kubadi
			354	Ambehera Ramkishan
			355	Ambeheradeeraj
			356	Kukhera
			357	Harot
			358	Chakdoa
			359	Kud
			360	Gehra Kothi
			361	Gughan Kalan
			362	Kakrna
			363	Kyara
			364	Jagatkhana
			365	Handola-1
			366	Handola-2
			367	Kamoon
			368	Sanhal
			369	Tanda
			370	Tanoh
			371	Kaihwin
			372	Karsai
			373	Dughar
			374	Tureta
			375	Dadiyar
			376	Lathiani
			377	U.Rajli
			378	Rajli Baniyala
			379	Tyasar
			380	Bilgran
			381	Aliyana
			382	Naloot
			383	Budhan-1
			384	Budhan-2
			385	Turkal
			386	Kughal
			387	Neri
			388	Padyola
			389	Kohdra
			390	Dhret Dam
			391	Daihan
			392	Rachhoh
			393	Kheri
			394	Saroh
			395	Chamyari
			396	Basaatar
			397	Kot
			398	Jandana
			399	Dolu
			400	Jarola
			401	Hatwana
			402	Piploo
			403	Nichla Thana
			404	Ghaloon
			405	Alsaan
			406	Bhyambhi
			407	Chataihar
			408	Tehi

Field	Question	Answer		
			409	Hatli Patiyalan
			410	Marot
			411	Nargru
			412	Chamukha Mini Awc
			413	Raipur-I
			414	Raipur-Ii
			415	Raipur-Iii
			416	Androli
			417	Dobar-I
			418	Dobar-Ii
			419	Proian-I
			420	Proian-Ii
			421	Kusiala
			422	Chaugath
			423	Lidkot
			424	Chulhari
			425	Muslim Kheri
			426	Gharwasra
			427	Kyor
			428	Makrair
			429	Makrair Sidh
			430	Balh Saili
			431	Changer
			432	Anokha Tanda
			433	Bihru Kalan
			434	Nughrari
			435	Changreri
			436	Mandli
			437	Thanakalan
			438	Majher
			439	Jhorkhar
			440	Rachhol
			441	Chhaproh
			442	Boosal
			443	Narghota
			444	Balh
			445	Kholi
			446	Budwar
			447	Braal
			448	Doh
			449	Tihra-1
			450	Tihra-Ii
			451	Aghlaur
			452	Sakaun
			453	New Sakaun
			454	Dhwala
			455	Dohak
			456	Kolka
			457	Lower Chatehar
			458	Upper Chatehar
			459	Polytechnical Ambota
			460	Dawali Ambota
			461	Jogdehi
			462	Jhalowali
			463	Handiyala
			464	Parla Behra
			465	Mahila Mandal
			466	Kharasi Mohalla
			467	Lambardara Mohalla
			468	Panchyat Ghar
			469	Jindwad Mahila Mandal

Field	Question	Answer

	470	Ambota Gumma
	471	Nagnath Ambota
	472	Chatehar Sant Mohalla
	473	Matyalika Saghnai
	474	Gujjar Khad
	475	Nangal Panga
	476	Panchyat Ghar
	477	Torewala Saghnai
	478	Chang Basti
	479	Mahila Mandal
	480	Kala Panga
	481	Guglehar
	482	Upper Guglehar
	483	Mahila Mandal
	484	Thakur Dwara
	485	Lath Muhalla
	486	Upper Sarai
	487	Teli Mohalla
	488	Harizan Basti
	489	Jat Sarai
	490	Obc Mohalla
	491	Sarai Jadla Keori
	492	Jaswal Mohalla
	493	Bazar-11
	494	Dehra Mohalla
	495	Swan Mohalla
	496	Mahila Mandal
	497	Shiv Dwala Upper Nagar Wala
	498	Pathani Mohalla
	499	Dharamshala Mohalla
	500	Kolar Mohalla
	501	Kuan Bala Mohalla
	502	Ara Mohalla
	503	Sadak Mohalla
	504	Piplu-1
	505	Bandu-11
	506	Ambi-111
	507	Mahila Mandal
	508	Bhat Basti
	509	Fatehpur
	510	Brahampur-1
	511	Brahampur-11
	512	Lower Baneda-1
	513	Lower Baneda -11
	514	Dharma-111
	515	Harwal
	516	Gondpur Baneda Upper -1
	517	Baneda -11
	518	Baneda -111
	519	Upper Baneda Roda-4
	520	Kuneran-1
	521	Sanoli
	522	Kuneran-4
	523	Kuneran-11
	524	Harizan Basti
	525	Bazigar Basti
	526	Bagan Mohalla
	527	Julaha Basti
	528	Bilu Di Talai
	529	Kailash Nagar -1

Field	Question	Answer		
			530	Kailashnagar-11 Tundkhuri
			531	Hariyala Kuteda
			532	Kamali Ram Nagar-1
			533	Ram Nagar -2 Haled
			534	Amlehar-1 Rampur Kuteda
			535	Amlehar-11 Harizan Basti
			536	Amlehar-111 Sunkali
			537	Amlehar Khas
			538	Gokal Nagar
			539	Upper Bhanjal Shankar Nagar
			540	Bhanjal Upper Guga Basti
			541	Bhanjal Upper Harizan Basti
			542	Lower Bhanjal -1 Sarai
			543	Lower Bhanjal -11 Bhatwal
			544	Bada Talab
			545	Lower Bhanjal -4
			546	Lower Bhanjal-5 Kadd
			547	Jit Pur Behari-1
			548	Jitpur Behri-11
			549	Thaplan
			550	Dakha Bala Kuan
			551	Braham Sarai
			552	Lower Bhathia Wala
			553	Panwada
			554	Nai Mohalla
			555	Badoh Depot
			556	Upper Bhathia Bala
			557	Shiv Mandir Kaloh
			558	Harizan Basti
			559	Bumbaloo
			560	Kaloh Behli
			561	Chang Basti
			562	Kaloh Beli
			563	Harizan Basti
			564	Beli Gagret
			565	Brahmin Basti Gagret
			566	Panchyat Ghar
			567	Gujjar Khad
			568	Dev Nagar
			569	Purana Amb Road Gagret
			570	Bharwai Road Gagret
			571	Hanuman Mandir
			572	Purana Amb Road Chakki
			573	Pakka Paroh
			574	Shiv Mandir
			575	Raipur Mohalla
			576	Rania Talab
			577	Miidle Depot
			578	Harizan Basti
			579	Kumhar Basti
			580	Bukhaipuir
			581	Brahaman Basti
			582	Chang Pukhar
			583	Obc Mohalla
			584	Marwadi Behal
			585	Marwadi Lower
			586	Lower Madwada
			587	Middle Madwada
			588	Upper Madwada

Field	Question	Answer		
			589	Tilla Takka
			590	Salohberi Samadi
			591	Lohar Muhalla
			592	Harizan Basti
			593	Salohberi Kothi
			594	Kurialai
			595	Joh Khas
			596	Panchyat Ghar
			597	Tillu Chaunta
			598	Joh Beh
			599	Joh Beh Khad
			600	Deoli Wadi
			601	Cylinder Factory
			602	Shiv Mandir Deoli
			603	Harizan Basti Deoli
			604	High School Deoli
			605	Deoli Chada Basti
			606	Tubewell Deoli
			607	Ghanari Moni Baba
			608	Ayrvedic Dispensary Ghanari
			609	Harizan Basti Ghanari
			610	Upper Ghanari
			611	Ghanri Chang Basti
			612	Upper Chang Basti
			613	Nangal Jarialan Talab
			614	Nangal Jarialan Middle School
			615	Nangal Jarialan Harizan Basti
			616	Nangal Jarialan Moru Panga
			617	Nangal Jariaan Theda
			618	Nangal Jarialan Kandi Bhavan
			619	Nangaj Jarialan Andwad
			620	Nangal Jariaalan Shivali
			621	Nangal Jarialan Jhalera
			622	Amboa Sub Centre
			623	Amboa Upper
			624	Amboa Primary School
			625	Amboa Harizan Basti
			626	Swan Par Harwall
			627	Bus Stand Mawa Kaholan
			628	Health Centre
			629	Mawa Kaholan Middle
			630	Tarali
			631	Bedha Mohalla
			632	Harizan Basti
			633	Shiv Badi
			634	Badhiakha
			635	Mahila Mandal
			636	Bagan Bala Kuan Chalet
			637	Simli Bala Kuan
			638	Pukhari Pur
			639	Mande Chalet
			640	Harizan Basti
			641	Girl School
			642	Tapriala
			643	Daulatpur Chowk
			644	Dav Daulatpur Chowk

Field	Question	Answer		
			645	Dholwaha Road
			646	Kua Devi
			647	Tuta Bala Kuan
			648	Bai Bhatha
			649	Babehad Mahila Mandal
			650	Middle School
			651	Lawana Mohalla
			652	Panchyat Ghar
			653	Maralu Mohalla
			654	Khad Gujaran
			655	Lambar Dar Mohalla
			656	Obc Mohalla
			657	Upper Gram
			658	Sc Basti
			659	Upper Society
			660	Seth Coloney
			661	Upper Khad Gujaran
			662	Bus Stand
			663	Upper Sarai Tateda
			664	Panchyat Ghar
			665	Factory Coloney
			666	Sc Basti
			667	Obc Mohalla
			668	Nepali Coloney
			669	Mahila Mandal
			670	Oel Ashram
			671	Upper Sarai
			672	Harizan Basti
			673	Haroti Mohalla
			674	Pirthipur Faquti
			675	Mahila Mandal
			676	Maidangarh
			677	Harizan Basti
			678	Lohar Basti
			679	Bank Ghar
			680	Pirthipur Chaua
			681	Pirthipur Khas
			682	Dangoh Khurd
			683	Upper Pirthipur
			684	Gujjar Khad Dangohkhas
			685	Setha Mohalla
			686	Mahila Mandal
			687	Dangoh Pirthipur Road
			688	Harizan Basti
			689	Gumma Dangohkhas
			690	Mohalla Kavirpanthi
			691	Dodua Dangoh
			692	Abhaypur Chang Basti
			693	Abhaypur Upper
			694	Abhaypur Middle
			695	Bathri (Present)
			696	Bathri I (Present)
			697	Lohar Muhalla
			698	Bahti Muhalla
			699	Rajput Muhalla
			700	Changare Muhalla
			701	Brahmin Muhalla
			702	Beetan (Present)
			703	Jakhewal (Present)
			704	Jakhewal I
			705	Gujjar Basti I

Field	Question	Answer		
			706	Gujjar Basti Iii
			707	Gujjar Basti Ii
			708	Harijan Muhalla
			709	Lohar Muhalla
			710	Singan Present
			711	Singan I
			712	Havelli Vala
			713	Brahmin Muhalla
			714	Rajput Muhalla
			715	Harijan Basti
			716	Kotli Vala
			717	Pangyan Vala
			718	Heera Nagar
			719	Sahoowal 1
			720	Sahowal Ii
			721	Rajputan Polician
			722	Brahman Muhalla
			723	Lohar Muhalla
			724	Dulehar
			725	Gaua
			726	Muslim Muhalla
			727	Mishra Muhalla
			728	Beebar Muhalla
			729	Bhagtan Muhalla
			730	Harijan Muhalla
			731	Brahman Muhalla
			732	Gondpur Jai Chand 1
			733	Gondpur Jai Chand Ii
			734	Gondpurjaichand Iii
			735	Kabir Panthi Muhalla
			736	Beldar Basati
			737	Brahman Muhalla
			738	Rajput Tarkhan Muhalla
			739	Gondpur Bullan 1
			740	Gandpur Bullan Ii
			741	Brahman Muhalla I
			742	Brahman Muhalla Ii
			743	Gongpur
			744	Pathak Muhalla
			745	Bank Ghar
			746	Harijan Basti
			747	Saini Basti
			748	Panchayat Ghar
			749	Bhadsali Harijan Basti
			750	Bagru Muhalla
			751	Bhuliyan Muhalla
			752	Manguwal Muhalla
			753	Shah Muhalla
			754	Bhadsali Varatmaan
			755	Bhadsali Haar
			756	Kalla Muhalla
			757	Bada Haar
			758	Chota Haar
			759	Jejon Moad
			760	Saloh Bhatta
			761	Jejon Moad Ii
			762	Ghaluwal Bazar
			763	Kyar Muhalla
			764	Saloh Haar
			765	Harijan Basti
			766	Saloh Mahadev I

Field	Question	Answer

	767	Saloh Mahadev Ii
	768	Shiv Mandir Saloh
	769	Dharampur Lower
	770	Gurduara Basti
	771	Sidh Channo Mandir
	772	Bada Beda
	773	Shiv Mandir
	774	Sansowal (Present)
	775	Samnal (Present)
	776	Kuruwala Samnal
	777	Bati Muhalla
	778	Harijan Basti
	779	Rahar Muhalla
	780	Main Bazar Samnal
	781	Rora (Present)
	782	Nichla Mazra
	783	Chande Muhalla
	784	Palaki Vala
	785	Haroli I(Present)
	786	Haroli Ii
	787	Roleyan Muhalla
	788	Harijan Basti
	789	Lohar Basti
	790	Bati Muhalla Dhol
	791	Haar Khera
	792	Kali Badi
	793	Saini Bati Muhalla
	794	Haijan Basti Khad I
	795	Harijan Basti Ii
	796	Jangle Panehra
	797	Pandoga Barrier
	798	Upper Pandoga
	799	Pandoga Lower
	800	Pandoga Kharoian Muhalla
	801	Pandoga Barrier Ii
	802	Chakk Muhalla Pandoga
	803	Tahlia Muhalla
	804	Atava Muhalla
	805	Pandoga Pathak Muhalla
	806	Saini Muhalla
	807	Jole Harijan Basti
	808	Pandoga Kyarian Muhalla
	809	Bag Bharwal Muhalla
	810	Brahman Basti
	811	Ispur Hoshiarpur Road
	812	Lawana Majra I
	813	Ispur 'Gagret Road
	814	Labana Majra -Ii
	815	Pathak Muhalla
	816	Ravidas Mandir
	817	Saruain Bag
	818	Tippar Muhalla
	819	Saloh Harijan Basti I
	820	Purian Muhalla
	821	Sr Sec. School Saloh
	822	Bholian Muhalla
	823	Jaat Muhalla
	824	Upper Badehra Phc
	825	Middle School Badehra
	826	Upper Harijan Basti
	827	Upper Lohar Muhalla

Field	Question	Answer		
			828	Katwal Muhalla
			829	Thakaran Muhalla
			830	Lavana Muhalla
			831	Gujjar Pahari Muhalla
			832	Lower Badera
			833	Shiv Mandir Badhera
			834	Jatt Muhalla
			835	Bhai Da Moad
			836	Jaswal Muhalla
			837	Upper Kanger
			838	Lower Kanger
			839	Bakralu Muhalla
			840	Kanger Haar
			841	Bharwal Muhalla
			842	Harijan Basti
			843	Dharampur (Present Upper)
			844	Harijan Basti
			845	Brahmin Satta Muhalla
			846	Malluwal Purana
			847	Polia Beet I
			848	Janni I
			849	Majra Jhole
			850	Polian Beet Ii
			851	Polian Iii
			852	Janni Ii
			853	Chhetran I
			854	Biderwal
			855	Harijan Dita
			856	Tarkhan Muhalla
			857	Khatri Muhalla
			858	Chhetran Ii
			859	Kungrat Vartman
			860	Rana Muhalla
			861	Ambi Muhalla
			862	Chowki Muhalla
			863	Bhandiara Ii
			864	Brahmin Kash Muhalla
			865	Bhandiara I
			866	Lalri I(Present)
			867	Lalri Ii (Present)
			868	Lalri Iii (Present)
			869	Lalri Iv (Present)
			870	Kelian Muhalla
			871	Brahmin Muhalla
			872	Dhanaru Muhalla
			873	Bati Muhalla
			874	Jhave Muhalla
			875	Bakralu Muhalla
			876	Tarkhan Muhalla
			877	Bharwal Muhalla
			878	Brahmin, Bati Muhalla
			879	Bati, Saini Muhalla
			880	Lohar Bati Muhalla
			881	Kalehra (Present)
			882	Saini Chuhowal Muhalla
			883	Kalehra Ii
			884	Khatta Muhalla
			885	Heera I (Present)
			886	Heera Ii (Present)
			887	Loothre Muhalla
			888	Dhugge Muhalla

Field	Question	Answer		
			889	Subboana
			890	Bhadhori (Present)
			891	Lohar Muhalla
			892	Brahmin Harijan Muhalla
			893	Bati Muhalla
			894	Nichli Jatt Basti
			895	Upper Palakwah
			896	Kante Varatman
			897	Jatt Muhalla
			898	Brahmin Muhalla
			899	Pandit Tarkhan, Naee, Lavana Muhalla
			900	Harijan Muhalla
			901	Tarkhan Muhalla
			902	Kumhaar Muhalla
			903	Bati Muhalla
			904	Gill Behra Thakra
			905	Thakaran Muhalla
			906	Gangtho (Present)
			907	Sutre Saini Muhalla
			908	Pulvari Muhalla
			909	Karampur
			910	Tholle Muhalla
			911	Kharwal Muhalla
			912	Karampur Lower
			913	Nangnoli (Present)
			914	Nangnoli (Haar)
			915	Lavana Majra
			916	Harijan Basti
			917	Panjawar I (Present)
			918	Panjawar Ii
			919	Jole I
			920	Panjawar Iii
			921	Jangle Muhalla
			922	Khatri Basti
			923	Jole Ii
			924	Tarkhana Mohalla
			925	Thhakki I
			926	Daulatpur Haar
			927	Thhakki Ii
			928	Upper Daulatpur
			929	Sain Basti
			930	Main Baazar Panjawar
			931	Master Muhalla
			932	Khad Present
			933	Bhaini Muhalla
			934	Hoshiarpur Road Khad
			935	Swanpar Malagarh
			936	Khad Centre
			937	Dangewala Muhalla
			938	Pubowal (Present)
			939	Pubowal (Center)
			940	Harijan Basti I
			941	Gurudwara Basti
			942	Harijan Basti Ii
			943	Padyan Muhalla
			944	Ramsar Muhalla
			945	Baliwal
			946	Jatt Muhalla
			947	Brahmin Muhalla
			948	Harijan Muhalla

Field	Question	Answer		
			949	Rajput Muhalla
			950	Panjuana I
			951	Panjuana Ii
			952	Pubowal Ii
			953	Badewala
			954	Gurudwara Muhalla Bilna
			955	Halera
			956	Kuthar I
			957	Makkorgarh
			958	Aperlipali
			959	Mishra Brahmin Muhala
			960	Kutharbeet Ii
			961	Kumhar Kabir Panthi Muhalla
			962	Ward No.I Santoshgarh
			963	Ward No.Ii Santoshgarh
			964	Ward No.Iii Santoshgarh
			965	Ward No.Iv Santoshgarh
			966	Ward No.V Santoshgarh
			967	Ward No.Vi Santoshgarh
			968	Ward No.Vii Santoshgarh
			969	Ward No.Ix Santoshgarh
			970	Ward No Viii Santoshgarh
			971	Ward No I+Ii Santoshgarh
			972	Ward No Vii, Viii, Ix
			973	Chhaterpur(Present)
			974	Bahti Muhalla
			975	Dada
			976	Bathu (Present)
			977	Gurplah (Present)
			978	Gurplah Ii
			979	Harijan Basti I
			980	Harijan Basti Ii
			981	Kelluan Muhalla
			982	Lamber Luvana Muhalla
			983	Upperla Muhalla
			984	Morvadi I
			985	Morvadi Ii
			986	Bathu Khas
			987	Nangal Kalan (Present)
			988	Jattpura (Present)
			989	Harijan Basti I
			990	Bharare Mohalla
			991	Brahmin Mohalla
			992	Jattpura Ii
			993	Nangal Kalan Ii
			994	Rajput Muhalla (Nangal Kalan)
			995	Tahliwal
			996	Nangal Khurd(Present)
			997	Manuwal
			998	Upper Manuwal
			999	Tibba Muhalla
			1000	Bahti Muhalla I
			1001	Bahti Muhalla Ii
			1002	Upper Rajput Muhalla
			1003	Lower Rajput Muhalla
			1004	Kiduan Muhalla
			1005	Batkalan (Present)
			1006	Upper Basti I
			1007	Upper Basti Ii

Field	Question	Answer

	1008	Nichli Basti
	1009	Saini Basti
	1010	Abada Barana
	1011	Abada Barana-Lohar Basti
	1012	Abada Barana-Brahman Khatri Mohalla
	1013	Jankaur
	1014	Barsada
	1015	Barsada-Bahti Jat Mohalla
	1016	Jankaur Saini Mohalla
	1017	Jankaur Tarkhan Mohalla
	1018	Sunehara
	1019	Sunehara-Kabir Panthi Moh.
	1020	Nangran
	1021	Nangran-Harijan Moh.
	1022	Nangran-Bahati Moh.
	1023	Nangran-Bahati Moh.-2
	1024	Nangran-Bahati Moh.-3
	1025	Nangran-Bahati Moh.-4
	1026	Nangran-Bahati Moh.-5
	1027	Nangran-Bahati Moh.-6
	1028	Badehar
	1029	Badehar-Swar Nai-1
	1030	Badehar-Swar Nai-2
	1031	Badehar-Chilawala
	1032	Jhurowal-1
	1033	Jhurowal-2
	1034	Khui Pekhu Bela
	1035	Jhurowal Bahati Moh.
	1036	Jhurowal Bahati Moh.-2
	1037	Nangal Slangri-1
	1038	Lehad
	1039	Parla Sanjhot
	1040	Nangal Salangri-2
	1041	Sanjhot
	1042	Nari
	1043	Nari- Lower -1
	1044	Nari- 2
	1045	Dhadhial-2
	1046	Dhadhial-
	1047	Nari-3
	1048	Chalola
	1049	Chalola-2
	1050	Chalola-3
	1051	Dhamandri-1
	1052	Dhamandri-2
	1053	Dhamandri-Satleta
	1054	Dhamandri-Mansoh
	1055	Dhamandri-3
	1056	Dhamandri-4
	1057	Dathwada
	1058	Dathwara-2
	1059	Barera
	1060	Barera-2
	1061	Behdala-1
	1062	Behdala-2
	1063	Vasdev Khidri Mohalla
	1064	Behdala-Harijan Mohalla-1
	1065	Behdala-Harijan Mohalla-2
	1066	Behdala-Khidri Mohalla

Field	Question	Answer		
			1067	Vasdev Harijan Mohalla
			1068	Behdala-Rajput Mohalla-1
			1069	Behdala-Rajput Mohalla-2
			1070	Behdala-Rajput Mohalla-3
			1071	Behdala-Valmiki Mohalla-1
			1072	Behdala-Valmiki Mohalla-2
			1073	Chatara
			1074	Chatara Mahadev
			1075	Chatara Brahaman Mohalla
			1076	Chatara Harijan Mohalla
			1077	Chatara Lohar Mohalla
			1078	Chatara Khatri Mohalla
			1079	Chatara Brahaman Mohalla-2
			1080	Chatara Labana Mohalla
			1081	Bharolian Kalan
			1082	Bharolian Kalan Jhingla Behda
			1083	Bharolian Kalan Bade Wala Mohalla
			1084	Bharolian Kalan Harijan Mohalla
			1085	Barnoh
			1086	Barnoh Jat Saini Mohalla
			1087	Dangera-1
			1088	Dangera-2
			1089	Dangoli
			1090	Dangoli Tarkhan Mohalla
			1091	Dangoli Harijan Mohalla
			1092	Dangoli Brahaman Mohalla
			1093	Dangoli Jatt Mohalla
			1094	Samoor
			1095	Bhaur
			1096	Samoor Bhaur Brahaman Mohalla
			1097	Samoor Bhaur Saur Mohalla
			1098	Samoor Bhaur Chhalwad Mohalla
			1099	Kuriala-1
			1100	Kuriala Bhaur
			1101	Kuriala Haled
			1102	Kuriala-2
			1103	Jhambar-1
			1104	Jhambar Chilliyan Harijan
			1105	Sarjehra
			1106	Laam
			1107	Jhambar Lower
			1108	Basal Upper
			1109	Basal Harijan Basti
			1110	Basal Brahaman Basti
			1111	Basal Khwaja Basti-1
			1112	Basal Bada Behda
			1113	Basal Bugde Bablu
			1114	Basal Khwaja Basti-2
			1115	Lower Basal
			1116	Lower Basal Harijan Basti
			1117	Lower Basal Dhiman Bahati Mohalla
			1118	Lower Basalbahati Mohalla-2

Field	Question	Answer		
			1119	Lower Basalbahati Mohalla-3
			1120	Lower Basalbahati Mohalla-4
			1121	Takka Bishna
			1122	Takka Ramsahay
			1123	Takka Bishna Saini Mohalla
			1124	Takka Bahati Mohalla
			1125	Takka Harijan Mohalla
			1126	Takka Ramsahay Saini Moh.
			1127	Takka Ramsahay Brahaman Harijan Moh.
			1128	Kotla Khurd
			1129	Kotla Khurd Khatri Mohalla
			1130	Kotla Khurd Lohar Mohalla
			1131	Bhatoli -1
			1132	Bhatoli -2
			1133	Bhatoli Khrtikre Mohlla
			1134	Bhatoli Chirbe Julahe Mohlla
			1135	Morbar-1
			1136	Morbar-2
			1137	Bhatoli Jatt Moh.-1
			1138	Bhatoli Jatt Moh.-2
			1139	Bhatoli Harijan Basti-1
			1140	Bhatoli Harijan Basti-2
			1141	Jakhera -1
			1142	Jakhera -2
			1143	Jakhera Hari Basti
			1144	Jakhera Basdev Moh.
			1145	Jakhera Brahman Moh.-1
			1146	Jakhera Brahman Moh.-2
			1147	Jakhera Balmiki Moh.
			1148	Bangrah Purana Kander
			1149	Fatewal
			1150	Bangrah -1
			1151	Bangrah -2
			1152	Bangrah Pukhru Moh.
			1153	Dehlan-Upper-1
			1154	Dehlan-Upper-2
			1155	Uppar Dehlan Mahldarji-1
			1156	Uppar Dehlan Mahldarji-2
			1157	Uppar Dehlan Negi Budu Mohlla
			1158	Uppar Dehlan Harjin Basti
			1159	Uppar Dehlan Grewal Mohlla
			1160	Uppar Dehlan Kavir Panthi Nai Mohlla
			1161	Upper Dehlan Bans Bansre Moh.
			1162	Uppar Dehlan Bade Wale Mohalla
			1163	Lower Dehla-1
			1164	Lower Dehla-2
			1165	Lower Dehla-3
			1166	Lower Dehlan Tarkhan Moh.
			1167	Lower Dehlan Bahti Moh.
			1168	Lower Dehlan Harijan Basti

Field	Question	Answer		
			1169	Lower Dehlan Brahman Moh.
			1170	Lower Dehlan Bahti Moh.-1
			1171	Lower Dehlan Bahti Moh.-11
			1172	Lower Dehlan Bahti Moh.-111
			1173	Lower Dehlan Bats Tikre
			1174	Mehtpur
			1175	Mehatpur Dwedi Mohalla
			1176	Shri Lanka Mohlla Mehtpur
			1177	Fateh Pur-1
			1178	Fateh Pur Harijan Basti-1
			1179	Fateh Pur Harijan Basti-2
			1180	Fateh Pur Bahati Mohalla
			1181	Fateh Pur -2
			1182	Khanpur-1
			1183	Khanpur Bahati Saini Mohalla
			1184	Khanpur Bahati Mohalla
			1185	Khanpur Harijan Basti
			1186	Khanpur -2
			1187	Uday Pur
			1188	Uday Pur Dhiman/ Rajput Mohalla
			1189	Sasan
			1190	Sasan Harijan Basti
			1191	Sasan Bahati Mohalla
			1192	Sasan Jatt Mohalla
			1193	Charat Garh-1
			1194	Charat Garh-2
			1195	Charat Garh-Lahar Mohalla
			1196	Charat Garh-Aeri Mohalla
			1197	Charat Garh-Harijan Basti
			1198	Charat Garh-Bajit Pur
			1199	Kuthar Kalan
			1200	Kuthar Kalan Bahati Mohalla
			1201	Kuthar Kalan Tarkhan/Rajput Mohalla
			1202	Tyuri-1
			1203	Tyuri-2
			1204	Tyuri-3
			1205	Panoh-1
			1206	Panoh-2
			1207	Panoh-3
			1208	Bhalola
			1209	Baduhi
			1210	Baduhi-1
			1211	Bhaloh
			1212	Ghandawal-1
			1213	Ghandawal-2
			1214	Badoli-1
			1215	Badoli Hari Basti
			1216	Badoli-2
			1217	Badsala
			1218	Badsala Khambuya Da Mohlla
			1219	Badsala Bankeya Da Mohlla
			1220	Jhalera Upper
			1221	Jhalera Lower

Field	Question	Answer		
			1222	Jhalera Banga Bala Mohlla
			1223	Jhlera Partap Mohlla
			1224	Jhlera New Beli Kloni
			1225	Rampur-1
			1226	Rampur-2
			1227	Rampur Jatt Mohlla
			1228	Rampur Harijan Mohlla
			1229	Rampur Bahti Brahman Mohlla
			1230	Kuthar Harijan Ghabre Mohlla
			1231	Kuthar Khurd Brahman Bati Mohlla
			1232	Kuthar Khurd
			1233	Lal Singi
			1234	Lal Singi Rajput Brahman Mohlla
			1235	Lal Singi Rajputsaini Mohlla
			1236	Rainsari Mdhey
			1237	Rainsari Purb
			1238	Rainsari Brahman Mohlla
			1239	Rainsari Harijan Moh.
			1240	Rainsari Bahti Moh.-1
			1241	Rainsari Brahman Jatt Moh.
			1242	Rainsari Bahti Mohlla-2
			1243	Lower Arniala
			1244	Lower Arniala Sharma Saini Mohalla
			1245	Lower Arniala Bahati Saini Mohalla
			1246	Upper Arniala
			1247	Upper Arniala Saini Harijan Mohalla
			1248	Upper Arniala Adarsh Nagar
			1249	Upper Arniala Rajput Lohar Mohalla
			1250	Upper Arniala Jhangri Mohalla
			1251	Upper Arniala Tarkhan Rajput Mohalla
			1252	Lower Kotla Kalan
			1253	Lower Kotla Kalan Saini Mohalla
			1254	Lower Kotla Kalan Brahaman Mohalla
			1255	Lower Kotla Kalan Tarkhan Mohalla
			1256	Upper Kotla Kalan
			1257	Upper Kotla Kalan Brahaman Mohalla
			1258	Upper Kotla Kalan Lohar Mohalla
			1259	Upper Kotla Kalan Tarkhan Mohalla
			1260	Upper Kotla Kalan Harijan Mohalla
			1261	Ajnoli
			1262	Ajnoli Saini Mohalla
			1263	Ajnoli Brahman Sood Mohalla
			1264	Ajnoli Upper
			1265	Lamlahri

Field	Question	Answer		
			1266	Lamlahri Brahman Mohalla
			1267	Lamlahri Upper
			1268	Lamlahri Badla Mohalla
			1269	Raypur-1
			1270	Raypur-2
			1271	Raypur-3
			1272	Raypur-4
			1273	Raypur Buje Bahti Mohlla
			1274	Raypur Hatti Bale Mohlla
			1275	Raypur Tarkhan Mohlla
			1276	Raypur Gabla Mohlla
			1277	Raypur Mehar Mohlla
			1278	Raypur Braman Mohlla
			1279	Raypur Jatt Behra
			1280	Raypur Bhatha Lekhraj
			1281	Basdera Kendr No.13
			1282	Basdera Kendr No.14
			1283	Basdera Kendr No.15
			1284	Basdera Kendr No.16
			1285	Basdera Kendr No.17
			1286	Basdera Kendr No.18
			1287	Basdera Bard No.3+5
			1288	Basdera Bard No.6
			1289	Basdera Bard No.9+8
			1290	Basdera Bard No.1
			1291	Lamlehra Purana
			1292	Lamlehra Brahman Mohlla
			1293	Lamlehra -2
			1294	Madan Pur
			1295	Madan Pur-Saini Mohalla
			1296	Madan Pur-2
			1297	Basoli-1
			1298	Basoli-2
			1299	Basoli-Dhesi Jat Mohalla
			1300	Basoli-Bhat Jat Mohalla
			1301	Basoli-Brahaman Mohalla
			1302	Basoli-Dhiman Mohalla
			1303	Basoli-Nala Mohalla
			1304	Malahat
			1305	Bharolian Khurd
			1306	Parli Patti Malahat
			1307	Brahaman Patti Malahat
			1308	Harijan Patti Malahat
			1309	Rajput Patti Malahat
			1310	Bharolian Khurd Brahanana-1
			1311	Bharolian Khurd Brahanana-2
			1312	Tabba-2
			1313	Rakkar
			1314	Tabba Rajput Mohalla-2
			1315	Tabba Rajput Mohalla-3
			1316	Tabba Harijan Mohalla
			1317	Tabba Lohar Mohalla
			1318	Tabba -1
			1319	Sanoli-1
			1320	Sanoli-2
			1321	Sanoli-Rajput Jat Mohalla-1
			1322	Sanoli-Rajput Jat Mohalla-2
			1323	Sanoli-Rajput Mohalla
			1324	Sanoli-Harijan Mohalla-1

Field	Question	Answer
		1325 Sanoli-Harijan Mohalla-2<br>1326 Sanoli-Brahaman Mohalla<br>1327 Majara<br>1328 Majara Jat Mohalla-1<br>1329 Majara Jat Mohalla-2<br>1330 Majara Jat Mohalla-3<br>1331 Malukpur<br>1332 Malukpur Jat Mohalla<br>1333 Binewal<br>1334 Puhna-1<br>1335 Puhna-2<br>1336 Puhna-3<br>1337 Ajoli<br>1338 Ajauli Brahman Mohalla-1<br>1339 Ajauli Brahman Mohalla-2<br>1340 Ajauli Harijan Basti<br>1341 Ajauli Bahati Jat Mohalla-1<br>1342 Ajauli Bahati Jat Mohalla-2<br>1343 Prem Nagar<br>1344 Gursar Mohalla<br>1345 Vikas Nagar<br>1346 Vivek Nagar<br>1347 Pulwala Bazar<br>1348 Purana Dakkhana<br>1349 Shiv Nagar<br>1350 Nagraj Mohalla<br>1351 Dc Colony<br>1352 Behli Mohalla-<br>1353 Neela Ghat<br>1354 Sabji Mandi W.No-1<br>1355 W.No-1 Centre-2<br>1356 Galua-1<br>1357 Galua-2<br>1358 Ward (2+7)<br>1359 Ward (4+8)<br>1360 Behli Mohalla-2<br>1361 Ward 7&11 Centre-2<br>1362 Chanderlok Colony<br>1363 Neelaghat Colony<br>1364 Friends Colony
Costing - Anganwadi		
Costing - Anganwadi > Human Resources Cost		
aw_k_1 *(required)*	Post (staff in place)	1 Aganwadi Worker<br>2 Aganwadi Helper
j_3 *(required)*	Sanctioned (no.)<br>*Anganwadi Worker*<br>*Question relevant when: selected(${aw_k_1} ,'1')*<br>*Response constrained to: . >= 0 and . <= 2*	
j_3_in *(required)*	In-position (No.)<br>*Anganwadi Worker*<br>*Response constrained to: . >= 0 and . <= ${j_3}*	
Costing - Anganwadi > Human Resources Cost > Anganwadi Worker (1)<br>*Group relevant when: selected(${aw_k_1} ,'1')*		(Repeated group)
j_4 *(required)*	Monthly Salary (INR) per person<br>*Fill 999 if data not available*<br>*Response constrained to: . >= 0 and . <= 30000*	
j_5 *(required)*	Daily Duty Hours on an average<br>*Response constrained to: . >= 0 and . <= 10*	
j_6 *(required)*	Duty Days per Week<br>*Response constrained to: . >= 0 and . <= 7*	
j_3_h *(required)*	Sanctioned (no.)<br>*Anganwadi Helper*<br>*Question relevant when: selected(${aw_k_1} ,'2')*	

Field	Question	Answer
	Response constrained to: . >= 0 and . <= 2	
j_3_h_in *(required)*	In-position (No.)<br>*Anganwadi Helper*<br>*Response constrained to: . >= 0 and . <= ${j_3_h}*	
Costing - Anganwadi > Human Resources Cost > Anganwadi Helper (1)<br>*Group relevant when: selected(${aw_k_1} ,'2')*		(Repeated group)
j_4_h *(required)*	Monthly Salary (INR) per person<br>*Fill 999 if data not available*<br>*Response constrained to: . >= 0 and . <= 30000*	
j_5_h *(required)*	Daily Duty Hours on an average<br>*Response constrained to: . >= 0 and . <= 10*	
j_6_h *(required)*	Duty Days per Week<br>*Response constrained to: . >= 0 and . <= 7*	
j_7_h *(required)*	Broad Job Responsibilities(Additional responsibilities for WINGS Scale-up)	
j_8_h *(required)*	Performance-Based Incentives<br>*Fill 999 if data not available*	
k_1 *(required)*	Are any training sessions conducted by this facility?	1 Yes<br>2 No
Costing - Anganwadi > Human Resources Cost > k_1_group<br>*Group relevant when: ${k_1} =1*		
k_2 *(required)*	Number of training session conducted in last one year<br>*Fill 999 if data not available*	
Costing - Anganwadi > Human Resources Cost > k_1_group > Training session (1)<br>*Group relevant when: ${k_2} != 999*		(Repeated group)
k_2_1 *(required)*	Name of training session conducted?<br>*Response constrained to: not(regex(.,'^(.*)\d(.*)$'))*	
k_2_2 *(required)*	Who were trainees<br>*Response constrained to: not(regex(.,'^(.*)\d(.*)$'))*	
k_2_3 *(required)*	Total no of sessions/batch conducted in the last one year<br>*Response constrained to: . >= 0 and . <= 10*	
k_2_4 *(required)*	Total number of personnel trained last year?<br>*Response constrained to: . >= 0 and . <= 100*	
k_2_4_cost *(required)*	Total cost of each training session (Including TA,DA,honorarium,food and lodging,training matarials venue cost,cost for trainers ,opportuinty cost ,other logistics)	
Costing - Anganwadi > Human Resources Cost > iec_cost		
k_4_1 *(required)*	What SBCC (Social and Behavioral Change Communication) activities are conducted in the facility or catered to by your facility?	1 Nutrition<br>2 WasH/Hygiene<br>3 Family Planning<br>4 Health screening and treatment<br>5 Mental Health<br>99 Other (specify)
Costing - Anganwadi > Human Resources Cost > iec_cost > [k_4_count1] (1)		(Repeated group)
k_4_2 *(required)*	How many of these activities were held in last 3 months?<br>*Response constrained to: . >= 0 and . <= 15*	
k_4_3 *(required)*	Unit cost of each activity (transportation, logistic support, others)	
k_5 *(required)*	Do you refer any of the following beneficiary groups?<br>*Response constrained to: not(selected(${k_5} , '6') and count-selected(${k_5}) > 1)*	1 Preconception women (18-35 Years)<br>2 Pregnant women<br>3 Postnatal/ lactating women<br>4 0 to 6 Months Infants<br>5 6-24 Months Infants & Children<br>6 No
Costing - Anganwadi > Human Resources Cost > [k_5_1count1] (1)		(Repeated group)
Costing - Anganwadi > Human Resources Cost > [k_5_1count1] (1) > beneficiary_referral<br>*Group relevant when: ${k_5} !=6*		
k_5_1 *(required)*	[k_5_1count1] - Common Reasons for Referral<br>*Response constrained to: not(regex(.,'^(.*)\d(.*)$'))*	
k_5_2 *(required)*	[k_5_1count1] - Name of Facility where typically Referred<br>*Response constrained to: not(regex(.,'^(.*)\d(.*)$'))*	
k_5_3 *(required)*	[k_5_1count1] - Total Numbers of Referrals / in last one year<br>*Response constrained to: . >= 0 and . <= 100*	
k_5_4 *(required)*	[k_5_1count1] - Total cost of each referral (including fuel, driver, and any additional support during the referral)	

Field	Question	Answer
	Response constrained to: . >= 0 and . <= 2500	
k_6 *(required)*	What equipment is used specifically for the beneficiary groups?	1 Weighing scales adult(Digital)<br>2 Weighing scales baby(Digital)<br>3 Weighing scales adult(Analog)<br>4 Weighing scales baby(Analog)<br>5 Stadiometers<br>6 Infantometers<br>7 Computers<br>8 Printers<br>9 Tablets<br>10 Smartphones<br>11 Tracking systems(POSHAN)<br>12 Registers<br>13 Mobile Application-WINGS<br>14 Toys for ECD<br>15 Food storage containers<br>16 Food weighing scale<br>17 Cooking vessels<br>18 Serving utensils<br>19 Cooking fuel<br>99 Other (Specify)
Costing - Anganwadi > [k_6_1count1] (1)		(Repeated group)
Costing - Anganwadi > [k_6_1count1] (1) > equipment_wise_cost		
k_6_5 *(required)*	Total number of Units<br>*If no data available fill 999*	
k_6_1 *(required)*	What were the initial cost including accessories for - [k_6_1count1]?<br>*If no data available fill 999*	
k_6_2 *(required)*	Maintenance costs or operational costs for - [k_6_1count1]<br>*If no data available fill 999*	
k_6_3 *(required)*	How many [k_6_1count1] have been procured in last one year ?<br>*If no data available fill 999*	
k_6_4 *(required)*	How many [k_6_1count1] have been supplied to the facility in last one year ?<br>*If no data available fill 999*	
k_8 *(required)*	Remarks of Respondent on the challenges faced while delivering services.<br>*Response constrained to: not(regex(.,'^(.*)\d(.*)$'))*	
k_8_1	Possible solution.<br>*Response constrained to: not(regex(.,'^(.*)\d(.*)$'))*	
k_9 *(required)*	Remarks by Investigator.<br>*Response constrained to: not(regex(.,'^(.*)\d(.*)$'))*	

HWC Cost Form

Field	Question	Answer
worker *(required)*	Worker Name	151 Abuhamza<br>152 Anmol Saini<br>153 Anshika Sahota<br>154 Ekta<br>155 Jyoti Devi<br>156 Kritika Thakur<br>157 Mehak Thakur<br>158 Poonam Devi<br>159 Riya Puri<br>160 Shivanshi<br>161 Varsha Kumari<br>162 Anchal Walia<br>163 Harshali<br>164 Kritika Puri
blocks *(required)*	Block Names	block_1 Amb<br>block_2 Basdehra<br>block_3 Gagret<br>block_4 Haroli<br>block_5 Thanakalan
hwc_selected *(required)*	Health wellness centre (HWC)	hwc_1 HWC-HSC KatoharKalan<br>hwc_2 HWC-HSC KatoharKhurd<br>hwc_3 HWC-HSC Kuthiari<br>hwc_4 HWC-HSC Neharian<br>hwc_5 HWC-HSC Panjoa<br>hwc_6 HWC-HSC Jagannath Mandir<br>hwc_7 HWC-HSC Naloh<br>hwc_8 HWC-HSC PolianProhitan<br>hwc_9 HWC-HSC Ripooh Misran<br>hwc_10 HWC-HSC Behra<br>hwc_11 HWC-HSC Diara<br>hwc_12 HWC-HSC Hamboli<br>hwc_13 HWC-HSC Takarala<br>hwc_14 HWC-HSC Thathal<br>hwc_15 HWC-HSC Badwana<br>hwc_16 HWC-HSC BaherBatehar<br>hwc_17 HWC-HSC Dilwari<br>hwc_18 HWC-HSC Ghangret<br>hwc_19 HWC-HSC GindpurMaloun<br>hwc_20 HWC-HSC Kharoh<br>hwc_21 HWC-HSC Thanikpura<br>hwc_22 HWC-HSC Chahbag<br>hwc_23 HWC-HSC Chowar<br>hwc_24 HWC-HSC Daloh<br>hwc_25 HWC-HSC Gangoti (sapouri)<br>hwc_26 HWC-HSC Lohara<br>hwc_27 HWC-HSC Andora<br>hwc_28 HWC-HSC Saloi<br>hwc_29 HWC-HSC Sidhchalher<br>hwc_30 HWC-HSC Suin<br>hwc_31 HWC-HSC Basal<br>hwc_32 HWC-HSC KotlaKhurd

Field	Question	Answer		
			hwc_33	HWC-HSC Rainsary
			hwc_34	HWC-HSC Takka
			hwc_35	HWC-HSC Basoli
			hwc_36	HWC-HSC Dangoli
			hwc_37	HWC-HSC Kotla Kalan
			hwc_38	HWC-HSC Lamlehri
			hwc_39	HWC-HSC SamoorKalan
			hwc_40	HWC-HSC Badoli
			hwc_41	HWC-HSC Badsala
			hwc_42	HWC-HSC Ghandwal
			hwc_43	HWC-HSC Kuriala
			hwc_44	HWC-HSC NangalSalangri
			hwc_45	HWC-HSC Panoh
			hwc_46	HWC-HSC Teuri
			hwc_47	HWC-HSC Bedehar
			hwc_48	HWC-HSC Behdala
			hwc_49	HWC-HSC BhadolianKalan
			hwc_50	HWC-HSC Charatgarh
			hwc_51	HWC-HSC Chattara
			hwc_52	HWC-HSC Chattarpur
			hwc_53	HWC-HSC Fatehwal
			hwc_54	HWC-HSC Jalgran
			hwc_55	HWC-HSC Jankaur
			hwc_56	HWC-HSC Jakhera
			hwc_57	HWC-HSC Jhudowal
			hwc_58	HWC-HSC Malahat
			hwc_59	HWC-HSC Nangran
			hwc_60	HWC-HSC Rakkar
			hwc_61	HWC-HSC Rampur
			hwc_62	HWC-HSC Sanoli
			hwc_63	HWC-HSC Sassan
			hwc_64	HWC-HSC Gondpur Banera
			hwc_65	HWC-HSC Kuneran
			hwc_66	HWC-HSC Nakroh
			hwc_67	HWC-HSC Ambota
			hwc_68	HWC-HSC Guglehar
			hwc_69	HWC-HSC Keori
			hwc_70	HWC-HSC KutheraJaswalan
			hwc_71	HWC-HSC Loharli
			hwc_72	HWC-HSC MawaSindhian
			hwc_73	HWC-HSC Oel
			hwc_74	HWC-HSC Pambra
			hwc_75	HWC-HSC Saghnai
			hwc_76	HWC-HSC Amboa
			hwc_77	HWC-HSC Babehar
			hwc_78	HWC-HSC Bhaderkali
			hwc_79	HWC-HSC Chalet
			hwc_80	HWC-HSC Dangoh
			hwc_81	HWC-HSC Deoli
			hwc_82	HWC-HSC Ghanari
			hwc_83	HWC-HSC Mandwara
			hwc_84	HWC-HSC MawaKohlan
			hwc_85	HWC-HSC Nangal Jariyala
			hwc_86	HWC-HSC Pirthipur

Field	Question	Answer		
			hwc_87	HWC-HSC Salohberri
			hwc_88	HWC-HSC Baliwal
			hwc_89	HWC-HSC Dharampur
			hwc_90	HWC-HSC Sainsowal
			hwc_91	HWC-HSC Bathri
			hwc_92	HWC-HSC Bathu
			hwc_93	HWC-HSC Beetan
			hwc_94	HWC-HSC Nangal Kalan
			hwc_95	HWC-HSC Singan
			hwc_96	HWC-HSC Ispur
			hwc_97	HWC-HSC Chhetran
			hwc_98	HWC-HSC Gondpur Bulla
			hwc_99	HWC-HSC Janani
			hwc_100	HWC-HSC Kuthar Beet
			hwc_101	HWC-HSC Polian Beet
			hwc_102	HWC-HSC Pubowal
			hwc_103	HWC-HSC Bhadauri
			hwc_104	HWC-HSC Lalehri
			hwc_105	HWC-HSC Nangal Khurd
			hwc_106	HWC-HSC Palakwah
			hwc_107	HWC-HSC Pandoga
			hwc_108	HWC-HSC Nangnoli
			hwc_109	HWC-HSC Panjawar
			hwc_110	HWC-HSC Kangar
			hwc_111	HWC-HSC Chamiari
			hwc_112	HWC-HSC Jarola
			hwc_113	HWC-HSC Piploo
			hwc_114	HWC-HSC Bharmout
			hwc_115	HWC-HSC Charoli
			hwc_116	HWC-HSC Charara
			hwc_117	HWC-HSC Deehar
			hwc_118	HWC-HSC Dhanet
			hwc_119	HWC-HSC Dhundla
			hwc_120	HWC-HSC Jassana
			hwc_121	HWC-HSC Kodra
			hwc_122	HWC-HSC Tanoh
			hwc_123	HWC-HSC Balh
			hwc_124	HWC-HSC Bihroo
			hwc_125	HWC-HSC Boul
			hwc_126	HWC-HSC Budhwar
			hwc_127	HWC-HSC Chugath
			hwc_128	HWC-HSC Chulari
			hwc_129	HWC-HSC Harot
			hwc_130	HWC-HSC Karian
			hwc_131	HWC-HSC Paroin
			hwc_132	HWC-HSC Saili
			hwc_133	HWC-HSC Talai
			hwc_134	HWC-HSC Ambehra
			hwc_135	HWC-HSC Baduhi
			hwc_136	HWC-HSC Bhindla
			hwc_137	HWC-HSC Jol
Costing - HWC				
Costing - HWC > Human Resources Cost				
hwc_k_1 *(required)*	Post (staff in place)		1	CHO
			2	Health Worker (Female)/ ANM
			3	Health Worker/Health Assistant (Male)
			4	ASHA

Field	Question	Answer
		99 Other (Specify)
Costing - HWC > Human Resources Cost > [hwc_k_1_count1] (1)		(Repeated group)
hwc_k_1_1 *(required)*	Number of staff in place - [hwc_k_1_count1]	
Costing - HWC > Human Resources Cost > [hwc_k_1_count1] (1) > [hwc_k_1_count1] (1)		(Repeated group)
Costing - HWC > Human Resources Cost > [hwc_k_1_count1] (1) > [hwc_k_1_count1] (1) > hwc_hr1		
hwc_k_1_2 *(required)*	Monthly Salary (INR) per person - [hwc_k_1_count1]	
hwc_k_1_3 *(required)*	Daily Duty Hours on an average - [hwc_k_1_count1]	
hwc_k_1_4 *(required)*	Duty Days per Week - [hwc_k_1_count1]	
hwc_k_1_5 *(required)*	Broad Job Responsibilities- [hwc_k_1_count1] (Additional responsibilities for WINGS Scale-up)	
hwc_k_1_6 *(required)*	Performance-Based Incentives - [hwc_k_1_count1]	
hwc_k_2 *(required)*	Are any training sessions conducted by this facility?	1 Yes<br>2 No
hwc_k_count *(required)*	Number of training session conducted in last one year<br>*If data not available fill 999*<br>*Question relevant when: ${hwc_k_2} =1*	
Costing - HWC > Training Costs (1)<br>*Group relevant when: ${hwc_k_count} != 999*		(Repeated group)
hwc_k_2_1 *(required)*	Name of training session conducted at Facility/community level<br>*Response constrained to: not(regex(.,'^(.*)\d(.*)$'))*	
hwc_k_2_2 *(required)*	Who were/are the trainees<br>*Response constrained to: not(regex(.,'^(.*)\d(.*)$'))*	
hwc_k_2_3 *(required)*	Total no of sessions/batch conducted in the last one year	
hwc_k_2_4 *(required)*	Total number of personnel trained last year?	
hwc_k_2_5 *(required)*	Total cost of each training session(TA/DA, Honorarium ,Food and lodging,Training materials,Venue cost,Cost for trainers,Opportunity cost,Other logistics.)<br>*fill 999 if data not available*	
hwc_k_4_2 *(required)*	Are beneficiaries referred for scans outside the facility?	1 Yes<br>2 No
hwc_k_4_type *(required)*	In which type of facility<br>*Question relevant when: ${hwc_k_4_2} =1*	1 Government<br>2 Private<br>3 Private/Empanelled/JSSK
hwc_k_4_6 *(required)*	Is this cost covered by the government or is it out-of-pocket?<br>*Question relevant when: ${hwc_k_4_2} =1*	1 Government<br>2 Out of Pocket
hwc_k_4_7 *(required)*	How many beneficiaries referred for scan in last one year<br>*fill 999 if data not available*<br>*Question relevant when: ${hwc_k_4_2} =1*	
hwc_k_7 *(required)*	What was the investment in IEC (Information, Education, and Communication) materials in the past 1 year?<br>*fill 999 if data not available*	
hwc_k_8 *(required)*	What SBCC (Social and Behavioral Change Communication) activities are conducted in the facility or catered to by your facility?	1 Nutrition<br>2 WasH/Hygiene<br>3 Family Planning<br>4 Health screening and treatment<br>5 Mental Health<br>99 Other (specify)
Costing - HWC > [hwc_k_8_count1] (1)		(Repeated group)
hwc_8_1 *(required)*	How many of these activities are held in the last three months?	
hwc_8_2 *(required)*	What is the unit cost of each activity (including transportation, logistics, and other costs)?	
Costing - HWC > Referral Transportation Cost		
hwc_k_9 *(required)*	Do you refer any of the below beneficiary groups?<br>*Tick those that are referred*<br>*Response constrained to: not(selected(${hwc_k_9} , '6') and count-selected(${hwc_k_9}) > 1)*	1 Preconception women (18-35 Years)<br>2 Pregnant women<br>3 Postnatal/ lactating women<br>4 0 to 6 Months Infants<br>5 6-24 Months Infants & Children<br>6 No
Costing - HWC > Referral Transportation Cost > [hwc_k_9_count1] (1)		(Repeated group)
Costing - HWC > Referral Transportation Cost > [hwc_k_9_count1] (1) > Beneficiary Group Referral<br>*Group relevant when: ${hwc_k_9} !=6*		
hwc_k_9_1 *(required)*	Common Reasons for Referral	
hwc_k_9_2 *(required)*	Name of Facility where typically Referred	
hwc_k_9_3 *(required)*	Total Numbers of Referrals in last one year	

Field	Question	Answer
hwc_k_9_4 *(required)*	Total cost of each referral (including fuel, driver, and any additional support during the referral)<br>*fill 999 if data not available*	
hwc_k_12 *(required)*	Which of the following supplies are used specifically for these beneficiary groups?	1 Weighing Scale Adult (Digital)<br>2 Weighing Scale Adult (Analog)<br>3 Baby weighing Scale (Digital)<br>4 Baby weighing Scale (Analog)<br>5 Salter scale<br>6 POC equipments (blood glucose meters, rapid strep tests, urine dipsticks, and portable blood gas analyzer,etc)<br>7 Stadiometers<br>8 Infantometers<br>9 Lab equipment<br>10 SNCU/NBSU equipment<br>11 Computers<br>12 Printers<br>13 Tablets<br>14 Smartphones<br>15 Tracking systems<br>16 Registers<br>99 Other (Specify)
Costing - HWC > [hwc_k_12_count1] (1)		(Repeated group)
Costing - HWC > [hwc_k_12_count1] (1) > Additional Equipment Cost		
hwc_k_12_1 *(required)*	Total number of Units	
hwc_k_12_2 *(required)*	What were the initial cost including accessories for - [hwc_k_12_count1]?<br>*If no data available fill 999*	
hwc_k_12_3 *(required)*	Maintenance costs or operational costs for - [hwc_k_12_count1]<br>*If no data available fill 999*	
hwc_k_12_4 *(required)*	How many [hwc_k_12_count1] have been procured in last one year ?<br>*If no data available fill 999*	
hwc_k_12_5 *(required)*	How many [hwc_k_12_count1] have been supplied to the facility in last one year ?<br>*If no data available fill 999*	
hwc_k_14_r *(required)*	Remarks of Respondent on the challenges faced while delivering services and possible solution:	
hwc_k_15_r *(required)*	Remarks by Investigator:	
hwc_k_16_r *(required)*	Remarks by respondent	
photo1	Photo	
photo2	Photo	
photo3	Photo	
photo4	Photo	
photo5	Document	

PHC Cost Form

Field	Question	Answer	
worker	Worker Name	151	Abuhamza
		152	Anmol Saini
		153	Anshika Sahota
		154	Ekta
		155	Jyoti Devi
		156	Kritika Thakur
		157	Mehak Thakur
		158	Poonam Devi
		159	Riya Puri
		160	Shivanshi
		161	Varsha Kumari
		162	Anchal Walia
		163	Harshali
		164	Kritika Puri
blocks *(required)*	Block Names	block_1	Amb
		block_2	Basdehra
		block_3	Gagret
		block_4	Haroli
		block_5	Thanakalan
phc_selected *(required)*	PHC	phc_1	PHC Akrot
		phc_2	PHC Chaksrai
		phc_3	PHC Chururu
		phc_4	PHC Dharamshala Mahanta
		phc_5	PHC Lohara
		phc_6	PHC Shivpur
		phc_7	PHC Basal
		phc_8	PHC Basoli
		phc_9	PHC Chalola
		phc_10	PHC Dehlan
		phc_11	PHC Amlehar
		phc_12	PHC Badehra Rajputan
		phc_13	PHC Marwari
		phc_14	PHC Badehra
		phc_15	PHC Bathri
		phc_16	PHC Khad
		phc_17	PHC Kuthar Beet
		phc_18	PHC Palkwah
		phc_19	PHC Panjawar
		phc_20	PHC Saloh
		phc_25	PHC Baliwal
		phc_21	PHC Chamiari
		phc_22	PHC Lathiani
		phc_23	PHC Raipur Maidan
		phc_24	PHC Sohari Takoli
		phc_999_1	None
		phc_999_2	None
		phc_999_3	None
		phc_999_4	None
		phc_999_5	None
Costing - phc			
Costing - phc > Human Resources Cost			
phc_k_1 *(required)*	Post (staff in place)	1	MO MBBS
		2	Staff Nurses
		3	Pharmacist
		4	Storekeeper
		5	Lab Technician
		6	Health Worker (Female)/ ANM

Field	Question	Answer
		7 Health Worker/Health Assistant (Male)<br>8 Female health supervisor<br>9 Health Educator/ Counsellor<br>10 Data Entry Operator<br>99 Other (Specify)
Costing - phc > Human Resources Cost > [phc_k_1_count1] (1)		(Repeated group)
phc_k_1_1 *(required)*	Number of staff in place - [phc_k_1_count1]	
Costing - phc > Human Resources Cost > [phc_k_1_count1] (1) > [phc_k_1_count1] (1)<br>*Group relevant when: ${phc_k_1_1} != 999*		(Repeated group)
Costing - phc > Human Resources Cost > [phc_k_1_count1] (1) > [phc_k_1_count1] (1) > phc_hr1		
phc_k_1_2 *(required)*	Monthly Salary (INR) per person - [phc_k_1_count1]	
phc_k_1_3 *(required)*	Daily Duty Hours on an average - [phc_k_1_count1]	
phc_k_1_4 *(required)*	Duty Days per Week - [phc_k_1_count1]	
phc_k_1_5 *(required)*	Broad Job Responsibilities (Endline: Additional responsibilities for WINGS Scale-up) - [phc_k_1_count1]	
phc_k_1_6 *(required)*	Performance-Based Incentives- [phc_k_1_count1]	
phc_k_2 *(required)*	Are any training sessions conducted by this facility?	1 Yes<br>2 No
phc_k_count *(required)*	Number of training session conducted in last one year<br>*If data not available fill 999*<br>*Question relevant when: ${phc_k_2} =1*	
Costing - phc > Training Costs (1)<br>*Group relevant when: ${phc_k_2} =1 and ${phc_k_count} != 999*		(Repeated group)
phc_k_2_1 *(required)*	Name of training session conducted at Facility/community level<br>*Response constrained to: not(regex(.,'^(.*)\d(.*)$'))*	
phc_k_2_2 *(required)*	Who were/are the trainees<br>*Response constrained to: not(regex(.,'^(.*)\d(.*)$'))*	
phc_k_2_3 *(required)*	Total no of sessions/batch conducted in the last one year	
phc_k_2_4 *(required)*	Total number of personnel trained last year?	
phc_k_2_5 *(required)*	Total cost of each training session(TA/DA, Honorarium ,Food and lodging,Training materials,Venue cost,Cost for trainers,Opportunity cost,Other logistics.)	
phc_k_3_8 *(required)*	Is there a USG machine installed at the facility?	1 Yes<br>2 No
Costing - phc > phc_k_group		
phc_k_3_9 *(required)*	Please specify year of installation of the USG Machine<br>*fill 999 if respondent don't know the data*<br>*Question relevant when: ${phc_k_3_8} =1*	
phc_k_3_10 *(required)*	Please specify the cost of the USG Machine<br>*fill 999 if respondent don't know the data*<br>*Question relevant when: ${phc_k_3_8} =1*	
phc_k_3_11 *(required)*	Are beneficiaries referred for scans outside the facility?	1 Yes<br>2 No
phc_k_4 *(required)*	What is the total cost per scan when referred outside the facility?<br>*fill 999 if respondent don't know the data*<br>*Question relevant when: ${phc_k_3_11} =1*	
phc_k_4_1 *(required)*	In which type of facility<br>*Question relevant when: ${phc_k_3_11} =1*	1 Government<br>2 Private<br>3 Private/Empanelled/JSSK
phc_k_4_2 *(required)*	Is this cost covered by the government or is it out-of-pocket?<br>*Question relevant when: ${phc_k_3_11} =1*	1 Government<br>2 Out of Pocket
phc_k_5_1 *(required)*	How many total deliveries (normal) take place in your facility in a year?<br>*fill 999 if respondent don't know the data*	
phc_k_5_2 *(required)*	How many total deliveries (CS) take place in your facility in a year?<br>*fill 999 if respondent don't know the data*	
Costing - phc > phc_k_group1		
phc_k_5_3 *(required)*	Would you be able to tell me a rough cost estimate for each normal delivery including the HR cost, consumables, any other cost?<br>*fill 999 if respondent don't know the data*<br>*Question relevant when: ${phc_k_5_1} >0*	
phc_k_5_4 *(required)*	Would you be able to tell me a rough cost estimate for each CS delivery including the HR cost, consumables, any other cost?<br>*fill 999 if respondent don't know the data*<br>*Question relevant when: ${phc_k_5_2} >0*	
Costing - phc > phc_k_group2		

Field	Question	Answer
phc_k_7 *(required)*	What was the investment in IEC (Information, Education, and Communication) materials in the past 1 year?<br>*fill 999 if respondent don't know the data*	
phc_k_8 *(required)*	What SBCC (Social and Behavioral Change Communication) activities are conducted in the facility or catered to by your facility?	1 Nutrition<br>2 WasH/Hygiene<br>3 Family Planning<br>4 Health screening and treatment<br>5 Mental Health<br>99 Other (specify)<br>9999 None
Costing - phc > phc_k_group2 > [phc_k_8_count1] (1)<br>*Group relevant when: ${phc_k_8_count} != 999*		(Repeated group)
phc_8_1 *(required)*	How many of these activities are held each month or every three months?<br>*fill 999 if respondent don't know the data*	
phc_8_2 *(required)*	What is the unit cost of each activity (including transportation, logistics, and other costs)?<br>*fill 999 if respondent don't know the data*	
Costing - phc > Referral Transportation Cost		
phc_k_9 *(required)*	Do you refer any of the above beneficiary groups?<br>*Tick those that are referred*<br>*Response constrained to: not(selected(${phc_k_9} , '6') and count-selected(${phc_k_9}) > 1)*	1 Preconception women (18-35 Years)<br>2 Pregnant women<br>3 Postnatal/ lactating women<br>4 0 to 6 Months Infants<br>5 6-24 Months Infants & Children<br>6 No
Costing - phc > Referral Transportation Cost > [phc_k_9_count1] (1)		(Repeated group)
Costing - phc > Referral Transportation Cost > [phc_k_9_count1] (1) > Beneficiary Group Referral<br>*Group relevant when: ${phc_k_9} !=6*		
phc_k_9_1 *(required)*	Common Reasons for Referral<br>*Response constrained to: not(regex(.,'^(.*)\d(.*)$'))*	
phc_k_9_2 *(required)*	Name of Facility where typically Referred<br>*Response constrained to: not(regex(.,'^(.*)\d(.*)$'))*	
phc_k_9_3 *(required)*	Total Numbers of Referrals in last one year	
phc_k_9_4 *(required)*	Total cost of each referral (including fuel, driver, and any additional support during the referral)	
phc_k_12 *(required)*	Which of the following equipments are used specifically for these beneficiary groups?	1 Weighing Scale Adult (Digital)<br>2 Weighing Scale Adult (Analog)<br>3 Baby weighing Scale (Digital)<br>4 Baby weighing Scale (Analog)<br>5 Salter scale<br>6 POC equipments (blood glucose meters, rapid strep tests, urine dipsticks, and portable blood gas analyzer,etc)<br>7 Stadiometers<br>8 Infantometers<br>9 Lab equipment<br>10 SNCU/NBSU equipment<br>11 Computers<br>12 Printers<br>13 Tablets<br>14 Smartphones<br>15 Tracking systems<br>16 Registers<br>99 Other (Specify)
Costing - phc > [phc_k_12_count1] (1)		(Repeated group)
Costing - phc > [phc_k_12_count1] (1) > Additional Equipment Cost		
phc_k_12_1 *(required)*	Total number of Units	
phc_k_12_2 *(required)*	What were the initial cost including accessories for - [phc_k_12_count1]?<br>*If no data available fill 999*	
phc_k_12_3 *(required)*	Maintenance costs or operational costs for - [phc_k_12_count1]<br>*If no data available fill 999*	
phc_k_12_4 *(required)*	How many [phc_k_12_count1] have been procured in last one year ?	

Field	Question	Answer
	If no data available fill 999	
phc_k_12_5 *(required)*	How many [phc_k_12_count1] have been supplied to the facility in last one year ?<br>*If no data available fill 999*	
phc_k_14 *(required)*	Remarks of Respondent on the challenges faced while delivering services and possible solution:	
phc_k_15 *(required)*	Remarks by Investigator:	
photo1	Photo	
photo2	Photo	
photo3	Photo	
photo4	Photo	
photo5	Document	

CHC Cost Form

Field	Question	Answer
worker	Worker Name	151 Abuhamza<br>152 Anmol Saini<br>153 Anshika Sahota<br>154 Ekta<br>155 Jyoti Devi<br>156 Kritika Thakur<br>157 Mehak Thakur<br>158 Poonam Devi<br>159 Riya Puri<br>160 Shivanshi<br>161 Varsha Kumari<br>162 Anchal Walia<br>163 Harshali<br>164 Kritika Puri
blocks *(required)*	Block Names	block_1 Amb<br>block_2 Basdehra<br>block_3 Gagret<br>block_4 Haroli<br>block_5 Thanakalan
civil_hospital *(required)*	CHC	ch_3 CHC Dussara<br>ch_4 CHC Basdehra<br>ch_5 CHC Santoshgarh<br>ch_7 CHC Daulatpur Chowk<br>ch_9 CHC Beeton<br>ch_10 CHC Bhadsali<br>ch_11 CHC Dulehar<br>ch_12 CHC Kungrath<br>ch_14 CHC Thanakalan<br>ch_999_1 None<br>ch_999_2 None<br>ch_999_3 None<br>ch_999_4 None<br>ch_999_5 None
Costing - CHC		
Costing - CHC > Human Resources Cost		
chc_k_1 *(required)*	Post (staff in place)	1 MO MBBS<br>2 Nursing officer<br>3 Pharmacist<br>4 Storekeeper<br>5 Lab Technician<br>6 Health Worker (Female)/ ANM<br>7 Health Worker/Health Assistant (Male)<br>8 Female health supervisor<br>9 Health Educator/ Counsellor<br>10 Data Entry Operator<br>11 Obstetrician<br>12 Pediatrician<br>13 Anesthetist<br>14 Radiologist<br>15 Psychiatrist<br>16 Psychologist<br>17 Lactation counsellor<br>99 Other (Specify)
Costing - CHC > Human Resources Cost > [chc_k_1_count1] (1)		(Repeated group)
chc_k_1_1 *(required)*	Number of staff in place - [chc_k_1_count1]<br>*Fill 999 if no data available*	
Costing - CHC > Human Resources Cost > [chc_k_1_count1] (1) > [chc_k_1_count1] (1)<br>*Group relevant when: ${chc_k_1_1} != 999*		(Repeated group)

Field	Question	Answer
Costing - CHC > Human Resources Cost > [chc_k_1_count1] (1) > [chc_k_1_count1] (1) > chc_hr1		
chc_k_1_2 *(required)*	Monthly Salary (INR) per person - [chc_k_1_count1]<br>*Fill 999 if no data available*	
chc_k_1_3 *(required)*	Daily Duty Hours on an average - [chc_k_1_count1]<br>*Fill 999 if no data available*	
chc_k_1_4 *(required)*	Duty Days per Week - [chc_k_1_count1]<br>*Fill 999 if no data available*	
chc_k_1_5 *(required)*	Broad Job Responsibilities (Endline: Additional responsibilities for WINGS Scale-up) - [chc_k_1_count1]<br>*Fill NA if no data available*<br>*Response constrained to: not(regex(.,'^(.*)\d(.*)$'))*	
chc_k_1_6 *(required)*	Performance-Based Incentives - [chc_k_1_count1]<br>*Fill 999 if no data available*	
chc_k_2 *(required)*	Are any training sessions conducted by this facility?	1 Yes<br>2 No
k_2 *(required)*	Number of training session conducted in last one year<br>*Question relevant when: ${chc_k_2} =1*	
Costing - CHC > Training Costs (1)<br>*Group relevant when: ${chc_k_2} =1 and ${k_2} != 999*		(Repeated group)
chc_k_2_1 *(required)*	Name of training session conducted at Facility/community level<br>*Fill NA if no data available*<br>*Response constrained to: not(regex(.,'^(.*)\d(.*)$'))*	
chc_k_2_2 *(required)*	Who were/are the trainees<br>*Fill NA if no data available*<br>*Response constrained to: not(regex(.,'^(.*)\d(.*)$'))*	
chc_k_2_3 *(required)*	Total no of sessions/batch conducted in the last one year<br>*Fill 999 if no data available*	
chc_k_2_4 *(required)*	Total number of personnel trained last year?<br>*Fill 999 if no data available*	
chc_k_2_5 *(required)*	Total cost of each training session(TA/DA, Honorarium ,Food and lodging,Training materials,Venue cost,Cost for trainers,Opportunity cost,Other logistics.)	
chc_k_3_8 *(required)*	Is there a USG machine installed at the facility?	1 Yes<br>2 No
Costing - CHC > USG Machine		
chc_k_3_9 *(required)*	Please specify year of installation of the USG Machine<br>*Fill 999 if no data available*<br>*Question relevant when: ${chc_k_3_8} =1*	
chc_k_3_10 *(required)*	Please specify the cost of the USG Machine<br>*Fill 999 if no data available*<br>*Question relevant when: ${chc_k_3_8} =1*	
chc_k_3_11 *(required)*	How many scans for the below mentioned beneficiary groups are done in a month?<br>*Question relevant when: ${chc_k_3_8} =1*	
chc_k_3_12 *(required)*	Preconception women (aged 18-35 years)<br>*Fill 999 if no data available*<br>*Question relevant when: ${chc_k_3_8} =1*	
chc_k_3_13 *(required)*	Pregnant women<br>*Fill 999 if no data available*<br>*Question relevant when: ${chc_k_3_8} =1*	
chc_k_3_14 *(required)*	Postnatal/ lactating women<br>*Fill 999 if no data available*<br>*Question relevant when: ${chc_k_3_8} =1*	
chc_k_3_15 *(required)*	0-24 Months Infants & Children<br>*Fill 999 if no data available*<br>*Question relevant when: ${chc_k_3_8} =1*	
chc_k_3_16 *(required)*	Are benficiaries referred for scans outside the facility?	1 Yes<br>2 No
Costing - CHC > chc_usg_scan_cost		
chc_k_4_1 *(required)*	What is the total cost per scan when referred outside the facility?<br>*Fill 999 if no data available*<br>*Question relevant when: ${chc_k_3_16} =1*	
chc_k_4_2 *(required)*	In which type of facility<br>*Question relevant when: ${chc_k_3_16} =1*	1 Government<br>2 Private<br>3 Private/Empanelled/JSSK
chc_k_4_3 *(required)*	Is this cost covered by the government or is it out-of-pocket?<br>*Question relevant when: ${chc_k_3_16} =1*	1 Government<br>2 Out of Pocket
chc_k_5_1 *(required)*	How many deliveries (normal) take place in your facility in a year?<br>*Fill 999 if no data available*	
chc_k_5_2 *(required)*	How many deliveries (CS) take place in your facility in a year?	

Field	Question	Answer
	Fill 999 if no data available	
Costing - CHC > chc_k_group1		
chc_k_5_3 *(required)*	Would you be able to tell me a rough cost estimate for each normal delivery including the HR cost, consumables, any other cost?<br>*Fill 999 if no data available*<br>*Question relevant when: ${chc_k_5_1} >0*	
chc_k_5_4 *(required)*	Would you be able to tell me a rough cost estimate for each CS delivery including the HR cost, consumables, any other cost?<br>*Fill 999 if no data available*<br>*Question relevant when: ${chc_k_5_2} >0*	
chc_k_6 *(required)*	Is a NICU, SNCU, or NBSU functional in the facility?	1 Yes<br>2 No
Costing - CHC > chc_k_group2		
chc_k_7 *(required)*	What was the investment in IEC (Information, Education, and Communication) materials in the past 1 year?<br>*Fill 999 if no data available*	
chc_k_8 *(required)*	What SBCC (Social and Behavioral Change Communication) activities are conducted in the facility or catered to by your facility?<br>*Response constrained to: not(selected(${chc_k_8} , '9999') and count-selected(${chc_k_8}) > 1)*	1 Nutrition<br>2 WasH/Hygiene<br>3 Family Planning<br>4 Health screening and treatment<br>5 Mental Health<br>99 Other (specify)<br>9999 None
Costing - CHC > chc_k_group2 > [chc_k_8_count1] (1)<br>*Group relevant when: ${chc_k_8_count} != 999*		(Repeated group)
chc_8_1 *(required)*	How many of these activities are held each month or every three months?<br>*Fill 999 if no data available*	
chc_8_2 *(required)*	What is the unit cost of each activity (including transportation, logistics, and other costs)?<br>*Fill 999 if no data available*	
Costing - CHC > Referral Transportation Cost		
chc_k_9 *(required)*	Do you refer any of the above beneficiary groups?<br>*Tick those that are referred*	1 Preconception women (aged 18-35 years)<br>2 Pregnant women<br>3 Postnatal/ lactating women<br>4 0-24 Months Infants & Children<br>5 No referrals
Costing - CHC > Referral Transportation Cost > [chc_k_9_count1] (1)<br>*Group relevant when: if(${chc_k_9} !=5,1,0)*		(Repeated group)
Costing - CHC > Referral Transportation Cost > [chc_k_9_count1] (1) > Beneficiary Group Referral<br>*Group relevant when: if(${chc_k_9} !=5,1,0)*		
chc_k_9_1 *(required)*	Common Reasons for Referral<br>*Fill NA if no data available*<br>*Response constrained to: not(regex(.,'^(.*)\d(.*)$'))*	
chc_k_9_2 *(required)*	Name of Facility where typically Referred<br>*Fill NA if no data available*<br>*Response constrained to: not(regex(.,'^(.*)\d(.*)$'))*	
chc_k_9_3 *(required)*	Total Numbers of Referrals / Month<br>*Fill 999 if no data available*	
chc_k_9_4 *(required)*	Total cost of each referral (including fuel, driver, and any additional support during the referral)<br>*Fill NA if no data available*	
chc_k_12 *(required)*	Which of the following equipments are used specifically for these beneficiary groups?	1 Weighing Scale Adult (Digital)<br>2 Weighing Scale Adult (Analog)<br>3 Baby weighing Scale (Digital)<br>4 Baby weighing Scale (Analog)<br>5 Salter scale<br>6 POC equipments (blood glucose meters, rapid strep tests, urine dipsticks, and portable blood gas analyzer,etc)<br>7 Stadiometers<br>8 Infantometers<br>9 Lab equipment<br>10 SNCU/NBSU equipment

Field	Question	Answer
		11 Computers<br>12 Printers<br>13 Tablets<br>14 Smartphones<br>15 Tracking systems<br>16 Registers<br>99 Other (Specify)
Costing - CHC > [chc_k_12_count1] (1)		(Repeated group)
Costing - CHC > [chc_k_12_count1] (1) > Additional Equipment Cost		
chc_k_12_1 *(required)*	Total number of Units<br>*If no data available fill 999*	
chc_k_12_2 *(required)*	What were the initial cost including accessories for - [chc_k_12_count1]?<br>*If no data available fill 999*	
chc_k_12_3 *(required)*	Maintenance costs or operational costs for - [chc_k_12_count1]<br>*If no data available fill 999*	
chc_k_12_4 *(required)*	How many [chc_k_12_count1] have been procured in last one year ?<br>*If no data available fill 999*	
chc_k_12_5 *(required)*	How many [chc_k_12_count1] have been supplied to the facility in last one year ?<br>*If no data available fill 999*	
chc_k_14 *(required)*	Remarks of Respondent on the challenges faced while delivering services and possible solution:<br>*Fill NA if no data available*	
chc_k_15 *(required)*	Remarks by Investigator<br>*Fill NA if no data available*	
photo1	Photo	
photo2	Photo	
photo3	Photo	
photo4	Photo	
photo5	Document	

Hospital Cost Form

Field	Question	Answer
worker	Worker Name	151 Abuhamza<br>152 Anmol Saini<br>153 Anshika Sahota<br>154 Ekta<br>155 Jyoti Devi<br>156 Kritika Thakur<br>157 Mehak Thakur<br>158 Poonam Devi<br>159 Riya Puri<br>160 Shivanshi<br>161 Varsha Kumari<br>162 Anchal Walia<br>163 Harshali<br>164 Kritika Puri
blocks *(required)*	Block Names	block_1 Amb<br>block_2 Basdehra<br>block_3 Gagret<br>block_4 Haroli<br>block_5 Thanakalan
civil_hospital *(required)*	Civil hospital	ch_1 CH Amb<br>ch_2 CH Chintpurni<br>ch_3 CH Cum ESI Gagret<br>ch_4 CH Haroli<br>ch_5 CH Bangana<br>ch_999_1 None<br>ch_999_2 None<br>ch_999_3 None<br>ch_999_4 None<br>ch_999_5 None
Costing - Hospital		
Costing - Hospital > Human Resources Cost		
hosp_k_1 *(required)*	Post (staff in place)	1 MO MBBS<br>2 Nursing officer<br>3 Pharmacist<br>4 Storekeeper<br>5 Lab Technician<br>6 Health Worker (Female)/ ANM<br>7 Health Worker/Health Assistant (Male)<br>8 Female health supervisor<br>9 Health Educator/ Counsellor<br>10 Data Entry Operator<br>11 Obstetrician<br>12 Pediatrician<br>13 Anesthetist<br>14 Radiologist<br>15 Psychiatrist<br>16 Psychologist<br>17 Lactation counsellor<br>99 Other (Specify)
Costing - Hospital > Human Resources Cost > [hosp_k_1_count1] (1)		(Repeated group)
hosp_k_1_1 *(required)*	Number of staff in place - [hosp_k_1_count1]<br>*Fill 999 if no data available*	
Costing - Hospital > Human Resources Cost > [hosp_k_1_count1] (1) > [hosp_k_1_count1] (1)		(Repeated group)
Costing - Hospital > Human Resources Cost > [hosp_k_1_count1] (1) > [hosp_k_1_count1] (1) > hosp_hr1		
hosp_k_1_2 *(required)*	Monthly Salary (INR) per person - [hosp_k_1_count1]<br>*Fill 999 if no data available*	
hosp_k_1_3 *(required)*	Daily Duty Hours on an average - [hosp_k_1_count1]<br>*Fill 999 if no data available*	
hosp_k_1_4 *(required)*	Duty Days per Week - [hosp_k_1_count1]	

Field	Question	Answer
	Fill 999 if no data available	
hosp_k_1_5 *(required)*	Broad Job Responsibilities (Endline: Additional responsibilities for WINGS Scale-up) - [hosp_k_1_count1]<br>*Fill NA if no data available*<br>*Response constrained to: not(regex(.,'^(.*)\d(.*)$'))*	
hosp_k_1_6 *(required)*	Performance-Based Incentives - [hosp_k_1_count1]<br>*Fill 999 if no data available*	
hosp_k_2 *(required)*	Are any training sessions conducted by this facility?	1 Yes<br>2 No
hosp_count *(required)*	Number of training session conducted in last one year<br>*Question relevant when: ${hosp_k_2} =1*	
Costing - Hospital > Training Costs (1)<br>*Group relevant when: ${hosp_count} != 999*		(Repeated group)
hosp_k_2_1 *(required)*	Name of training session conducted at Facility/community level<br>*Fill NA if no data available*	
hosp_k_2_2 *(required)*	Who were/are the trainees<br>*Fill NA if no data available*	
hosp_k_2_3 *(required)*	Total no of sessions/batch conducted in the last one year<br>*Fill 999 if no data available*	
hosp_k_2_4 *(required)*	Total number of personnel trained last year?<br>*Fill 999 if no data available*	
hosp_k_2_5 *(required)*	Total cost of each training session(TA/DA, Honorarium ,Food and lodging,Training materials,Venue cost,Cost for trainers,Opportunity cost,Other logistics.)	
hosp_k_3_8 *(required)*	Is there a USG machine installed at the facility?	1 Yes<br>2 No
Costing - Hospital > USG Machine		
hosp_k_3_9 *(required)*	Please specify year of installation of the USG Machine<br>*Fill 999 if no data available*<br>*Question relevant when: ${hosp_k_3_8} =1*	
hosp_k_3_10 *(required)*	Please specify the cost of the USG Machine<br>*Fill 999 if no data available*<br>*Question relevant when: ${hosp_k_3_8} =1*	
hosp_k_3_11 *(required)*	How many scans for the below mentioned beneficiary groups are done in a month?<br>*Fill 999 if no data available*<br>*Question relevant when: ${hosp_k_3_8} =1*	
hosp_k_3_12 *(required)*	Preconception women (aged 18-35 years)<br>*Fill 999 if no data available*<br>*Question relevant when: ${hosp_k_3_8} =1*	
hosp_k_3_13 *(required)*	Pregnant women<br>*Fill 999 if no data available*<br>*Question relevant when: ${hosp_k_3_8} =1*	
hosp_k_3_14 *(required)*	Postnatal/ lactating women<br>*Fill 999 if no data available*<br>*Question relevant when: ${hosp_k_3_8} =1*	
hosp_k_3_15 *(required)*	0-24 Months Infants & Children<br>*Fill 999 if no data available*<br>*Question relevant when: ${hosp_k_3_8} =1*	
hosp_k_3_16	Are benficiaries referred for scans outside the facility?<br>*Question relevant when: ye*	1 Yes<br>2 No
Costing - Hospital > hosp_usg_scan_cost		
hosp_k_4_1 *(required)*	What is the average cost per scan when referred outside the facility?<br>*Fill 999 if no data available*<br>*Question relevant when: ${hosp_k_3_16} =1*	
hosp_k_4_2 *(required)*	In which type of facility<br>*Question relevant when: ${hosp_k_3_16} =1*	1 Government<br>2 Private<br>3 Private/Empanelled/JSSK
hosp_k_4_3 *(required)*	Is this cost covered by the government or is it out-of-pocket?<br>*Question relevant when: ${hosp_k_3_16} =1*	1 Government<br>2 Out of Pocket
hosp_k_5_1 *(required)*	How many deliveries (normal) take place in your facility in a year on an average?<br>*Fill 999 if no data available*	
hosp_k_5_2 *(required)*	How many deliveries (CS) take place in your facility in a year on an average?<br>*Fill 999 if no data available*	
Costing - Hospital > hosp_k_group1		
hosp_k_5_3 *(required)*	Would you be able to tell me a rough cost estimate for each normal delivery including the HR cost, consumables, any other cost?<br>*Fill 999 if no data available*<br>*Question relevant when: ${hosp_k_5_1} >0*	

Field	Question	Answer
hosp_k_5_4	Would you be able to tell me a rough cost estimate for each CS delivery including the HR cost, consumables, any other cost?<br>*Fill 999 if no data available*<br>*Question relevant when: ${hosp_k_5_2} >0*	
hosp_k_6 *(required)*	Is a NICU, SNCU, or NBSU functional in the facility?	1 Yes<br>2 No
Costing - Hospital > hosp_k_group2		
hosp_k_7 *(required)*	What was the investment in IEC (Information, Education, and Communication) materials in the past 1 year?<br>*Fill 999 if no data available*	
hosp_k_8 *(required)*	What SBCC (Social and Behavioral Change Communication) activities are conducted in the facility or catered to by your facility?<br>*Fill NA if no data available*<br>*Response constrained to: not(selected(${hosp_k_8} , '9999') and count-selected(${hosp_k_8}) > 1)*	1 Nutrition<br>2 WasH/Hygiene<br>3 Family Planning<br>4 Health screening and treatment<br>5 Mental Health<br>99 Other (specify)<br>9999 None
Costing - Hospital > hosp_k_group2 > [hosp_k_8_count1] (1)		(Repeated group)
hosp_8_1 *(required)*	How many of these activities are held each month or every three months?<br>*Fill 999 if no data available*	
hosp_8_2 *(required)*	What is the unit cost of each activity (including transportation, logistics, and other costs)?<br>*Fill 999 if no data available*	
Costing - Hospital > Referral Transportation Cost		
hosp_k_9 *(required)*	Do you refer any of the above beneficiary groups?<br>*Tick those that are referred*	1 Preconception women (aged 18-35 years)<br>2 Pregnant women<br>3 Postnatal/ lactating women<br>4 0-24 Months Infants & Children<br>5 No referrals
Costing - Hospital > Referral Transportation Cost > [hosp_k_9_count1] (1)<br>*Group relevant when: if(${hosp_k_9} !=5,1,0)*		(Repeated group)
Costing - Hospital > Referral Transportation Cost > [hosp_k_9_count1] (1) > Beneficiary Group Referral<br>*Group relevant when: if(${hosp_k_9} !=5,1,0)*		
hosp_k_9_1 *(required)*	Common Reasons for Referral<br>*Fill NA if no data available*<br>*Response constrained to: not(regex(.,'^(.*)\d(.*)$'))*	
hosp_k_9_2 *(required)*	Name of Facility where typically Referred<br>*Fill NA if no data available*<br>*Response constrained to: not(regex(.,'^(.*)\d(.*)$'))*	
hosp_k_9_3 *(required)*	Average Numbers of Referrals / Month<br>*Fill 999 if no data available*	
hosp_k_9_4 *(required)*	Average cost of each referral (including fuel, driver, and any additional support during the referral)<br>*Fill NA if no data available*	
hosp_k_12 *(required)*	Which of the following equipments are used specifically for these beneficiary groups?	1 Weighing Scale Adult (Digital)<br>2 Weighing Scale Adult (Analog)<br>3 Baby weighing Scale (Digital)<br>4 Baby weighing Scale (Analog)<br>5 Salter scale<br>6 POC equipments (blood glucose meters, rapid strep tests, urine dipsticks, and portable blood gas analyzer,etc)<br>7 Stadiometers<br>8 Infantometers<br>9 Lab equipment<br>10 SNCU/NBSU equipment<br>11 Computers<br>12 Printers<br>13 Tablets<br>14 Smartphones<br>15 Tracking systems<br>16 Registers

Field	Question	Answer
		99 Other (Specify)
Costing - Hospital > [hosp_k_12_count1] (1)		(Repeated group)
Costing - Hospital > [hosp_k_12_count1] (1) > Additional Equipment Cost		
hosp_k_12_1 *(required)*	Total number of Units<br>*Fill 999 if no data available*	
hosp_k_12_2 *(required)*	What were the initial cost including accessories for - [hosp_k_12_count1]?<br>*Fill 999 if no data available*	
hosp_k_12_3 *(required)*	Maintenance costs or operational costs for - [hosp_k_12_count1]<br>*Fill 999 if no data available*	
hosp_k_12_4 *(required)*	How many [hosp_k_12_count1] have been procured in last one year ?<br>*Fill 999 if no data available*	
hosp_k_12_5 *(required)*	How many [hosp_k_12_count1] have been supplied to the facility in last one year ?<br>*Fill 999 if no data available*	
hosp_k_14 *(required)*	Remarks of Respondent on the challenges faced while delivering services.<br>*Fill NA if no data available*	
hosp_k_15 *(required)*	Possible solution.<br>*Fill NA if no data available*	
hosp_k_16 *(required)*	Remarks by Investigator<br>*Fill NA if no data available*	
hosp_k_17 *(required)*	Remarks by respondent	
photo1	Photo	
photo2	Photo	
photo3	Photo	
photo4	Photo	
photo5	Document	

RH Cost Form

Field	Question	Answer
worker	Worker Name	151 Abuhamza
		152 Anmol Saini
		153 Anshika Sahota
		154 Ekta
		155 Jyoti Devi
		156 Kritika Thakur
		157 Mehak Thakur
		158 Poonam Devi
		159 Riya Puri
		160 Shivanshi
		161 Varsha Kumari
		162 Anchal Walia
		163 Harshali
		164 Kritika Puri
Costing - Hospital		
Costing - Hospital > Human Resources Cost		
hosp_k_1 *(required)*	Post (staff in place)	1 MO MBBS
		2 Nursing officer
		3 Pharmacist
		4 Storekeeper
		5 Lab Technician
		6 Health Worker (Female)/ ANM
		7 Health Worker/Health Assistant (Male)
		8 Female health supervisor
		9 Health Educator/ Counsellor
		10 Data Entry Operator
		11 Obstetrician
		12 Pediatrician
		13 Anesthetist
		14 Radiologist
		15 Psychiatrist
		16 Psychologist
		17 Lactation counsellor
		99 Other (Specify)
Costing - Hospital > Human Resources Cost > [hosp_k_1_count1] (1)		(Repeated group)
hosp_k_1_1 *(required)*	Number of staff in place - [hosp_k_1_count1]<br>*Fill 999 if no data available*	
Costing - Hospital > Human Resources Cost > [hosp_k_1_count1] (1) > [hosp_k_1_count1] (1)		(Repeated group)
Costing - Hospital > Human Resources Cost > [hosp_k_1_count1] (1) > [hosp_k_1_count1] (1) > hosp_hr1		
hosp_k_1_2 *(required)*	Monthly Salary (INR) per person - [hosp_k_1_count1]<br>*Fill 999 if no data available*	
hosp_k_1_3 *(required)*	Daily Duty Hours on an average - [hosp_k_1_count1]<br>*Fill 999 if no data available*	
hosp_k_1_4 *(required)*	Duty Days per Week - [hosp_k_1_count1]<br>*Fill 999 if no data available*	
hosp_k_1_5 *(required)*	Broad Job Responsibilities (Endline: Additional responsibilities for WINGS Scale-up) - [hosp_k_1_count1]<br>*Fill NA if no data available*<br>*Response constrained to: not(regex(.,'^(.*)\d(.*)$'))*	
hosp_k_1_6 *(required)*	Performance-Based Incentives - [hosp_k_1_count1]<br>*Fill 999 if no data available*	
hosp_k_2 *(required)*	Are any training sessions conducted by this facility?	1 Yes
		2 No
hosp_count *(required)*	Number of training session conducted in last one year<br>*Question relevant when: ${hosp_k_2} =1*	
Costing - Hospital > Training Costs (1)<br>*Group relevant when: ${hosp_count} != 999*		(Repeated group)
hosp_k_2_1 *(required)*	Name of training session conducted at Facility/community level<br>*Fill NA if no data available*	
hosp_k_2_2 *(required)*	Who were/are the trainees<br>*Fill NA if no data available*	
hosp_k_2_3 *(required)*	Total no of sessions/batch conducted in the last one year	

Field	Question	Answer
	Fill 999 if no data available	
hosp_k_2_4 *(required)*	Total number of personnel trained last year?<br>*Fill 999 if no data available*	
hosp_k_2_5 *(required)*	Total cost of each training session(TA/DA, Honorarium ,Food and lodging,Training materials,Venue cost,Cost for trainers,Opportunity cost,Other logistics.)	
hosp_k_3_8 *(required)*	Is there a USG machine installed at the facility?	1 Yes<br>2 No
Costing - Hospital > USG Machine		
hosp_k_3_9 *(required)*	Please specify year of installation of the USG Machine<br>*Fill 999 if no data available*<br>*Question relevant when: ${hosp_k_3_8} =1*	
hosp_k_3_10 *(required)*	Please specify the cost of the USG Machine<br>*Fill 999 if no data available*<br>*Question relevant when: ${hosp_k_3_8} =1*	
hosp_k_3_11 *(required)*	How many scans for the below mentioned beneficiary groups are done in a month?<br>*Fill 999 if no data available*<br>*Question relevant when: ${hosp_k_3_8} =1*	
hosp_k_3_12 *(required)*	Preconception women (aged 18-35 years)<br>*Fill 999 if no data available*<br>*Question relevant when: ${hosp_k_3_8} =1*	
hosp_k_3_13 *(required)*	Pregnant women<br>*Fill 999 if no data available*<br>*Question relevant when: ${hosp_k_3_8} =1*	
hosp_k_3_14 *(required)*	Postnatal/ lactating women<br>*Fill 999 if no data available*<br>*Question relevant when: ${hosp_k_3_8} =1*	
hosp_k_3_15 *(required)*	0-24 Months Infants & Children<br>*Fill 999 if no data available*<br>*Question relevant when: ${hosp_k_3_8} =1*	
hosp_k_3_16	Are benficiaries referred for scans outside the facility?<br>*Question relevant when: ye*	1 Yes<br>2 No
Costing - Hospital > hosp_usg_scan_cost		
hosp_k_4_1 *(required)*	What is the average cost per scan when referred outside the facility?<br>*Fill 999 if no data available*<br>*Question relevant when: ${hosp_k_3_16} =1*	
hosp_k_4_2 *(required)*	In which type of facility<br>*Question relevant when: ${hosp_k_3_16} =1*	1 Government<br>2 Private<br>3 Private/Empanelled/JSSK
hosp_k_4_3 *(required)*	Is this cost covered by the government or is it out-of-pocket?<br>*Question relevant when: ${hosp_k_3_16} =1*	1 Government<br>2 Out of Pocket
hosp_k_5_1 *(required)*	How many deliveries (normal) take place in your facility in a year on an average?<br>*Fill 999 if no data available*	
hosp_k_5_2 *(required)*	How many deliveries (CS) take place in your facility in a year on an average?<br>*Fill 999 if no data available*	
Costing - Hospital > hosp_k_group1		
hosp_k_5_3 *(required)*	Would you be able to tell me a rough cost estimate for each normal delivery including the HR cost, consumables, any other cost?<br>*Fill 999 if no data available*<br>*Question relevant when: ${hosp_k_5_1} >0*	
hosp_k_5_4	Would you be able to tell me a rough cost estimate for each CS delivery including the HR cost, consumables, any other cost?<br>*Fill 999 if no data available*<br>*Question relevant when: ${hosp_k_5_2} >0*	
hosp_k_6 *(required)*	Is a NICU, SNCU, or NBSU functional in the facility?	1 Yes<br>2 No
Costing - Hospital > hosp_k_group2		
hosp_k_7 *(required)*	What was the investment in IEC (Information, Education, and Communication) materials in the past 1 year?<br>*Fill 999 if no data available*	
hosp_k_8 *(required)*	What SBCC (Social and Behavioral Change Communication) activities are conducted in the facility or catered to by your facility?<br>*Fill NA if no data available*<br>*Response constrained to: not(selected(${hosp_k_8} , '9999') and count-selected(${hosp_k_8}) > 1)*	1 Nutrition<br>2 WasH/Hygiene<br>3 Family Planning<br>4 Health screening and treatment<br>5 Mental Health<br>99 Other (specify)<br>9999 None

Field	Question	Answer
Costing - Hospital > hosp_k_group2 > [hosp_k_8_count1] (1)		(Repeated group)
hosp_8_1 *(required)*	How many of these activities are held each month or every three months?<br>*Fill 999 if no data available*	
hosp_8_2 *(required)*	What is the unit cost of each activity (including transportation, logistics, and other costs)?<br>*Fill 999 if no data available*	
Costing - Hospital > Referral Transportation Cost		
hosp_k_9 *(required)*	Do you refer any of the above beneficiary groups?<br>*Tick those that are referred*	1 Preconception women (aged 18-35 years)<br>2 Pregnant women<br>3 Postnatal/ lactating women<br>4 0-24 Months Infants & Children<br>5 No referrals
Costing - Hospital > Referral Transportation Cost > [hosp_k_9_count1] (1)<br>*Group relevant when: if(${hosp_k_9} !=5,1,0)*		(Repeated group)
Costing - Hospital > Referral Transportation Cost > [hosp_k_9_count1] (1) > Beneficiary Group Referral<br>*Group relevant when: if(${hosp_k_9} !=5,1,0)*		
hosp_k_9_1 *(required)*	Common Reasons for Referral<br>*Fill NA if no data available*<br>*Response constrained to: not(regex(.,'^(.*)\d(.*)$'))*	
hosp_k_9_2 *(required)*	Name of Facility where typically Referred<br>*Fill NA if no data available*<br>*Response constrained to: not(regex(.,'^(.*)\d(.*)$'))*	
hosp_k_9_3 *(required)*	Average Numbers of Referrals / Month<br>*Fill 999 if no data available*	
hosp_k_9_4 *(required)*	Average cost of each referral (including fuel, driver, and any additional support during the referral)<br>*Fill NA if no data available*	
hosp_k_12 *(required)*	Which of the following equipments are used specifically for these beneficiary groups?	1 Weighing Scale Adult (Digital)<br>2 Weighing Scale Adult (Analog)<br>3 Baby weighing Scale (Digital)<br>4 Baby weighing Scale (Analog)<br>5 Salter scale<br>6 POC equipments (blood glucose meters, rapid strep tests, urine dipsticks, and portable blood gas analyzer,etc)<br>7 Stadiometers<br>8 Infantometers<br>9 Lab equipment<br>10 SNCU/NBSU equipment<br>11 Computers<br>12 Printers<br>13 Tablets<br>14 Smartphones<br>15 Tracking systems<br>16 Registers<br>99 Other (Specify)
Costing - Hospital > [hosp_k_12_count1] (1)		(Repeated group)
Costing - Hospital > [hosp_k_12_count1] (1) > Additional Equipment Cost		
hosp_k_12_1 *(required)*	Total number of Units<br>*Fill 999 if no data available*	
hosp_k_12_2 *(required)*	What were the initial cost including accessories for - [hosp_k_12_count1]?<br>*Fill 999 if no data available*	
hosp_k_12_3 *(required)*	Maintenance costs or operational costs for - [hosp_k_12_count1]<br>*Fill 999 if no data available*	
hosp_k_12_4 *(required)*	How many [hosp_k_12_count1] have been procured in last one year ?<br>*Fill 999 if no data available*	
hosp_k_12_5 *(required)*	How many [hosp_k_12_count1] have been supplied to the facility in last one year ?<br>*Fill 999 if no data available*	
hosp_k_14 *(required)*	Remarks of Respondent on the challenges faced while delivering services.<br>*Fill NA if no data available*	
hosp_k_15 *(required)*	Possible solution.<br>*Fill NA if no data available*	
hosp_k_16 *(required)*	Remarks by Investigator<br>*Fill NA if no data available*	

Field	Question	Answer
hosp_k_17 *(required)*	Remarks by respondent	
photo1	Photo	
photo2	Photo	
photo3	Photo	
photo4	Photo	
photo5	Document	
