# Supplementary material for: Implementation research to scale up the women and infants integrated interventions for growth study (WINGS) in Himachal Pradesh: Protocol for a quasi-experimental, mixed-methods study
Source: PLoS One. 2026 Feb 17;21(2):e0341048. doi: 10.1371/journal.pone.0341048 (PMC12912596; doi:10.1371/journal.pone.0341048)
Supplement: S5 File — (PDF) [file pone.0341048.s006.pdf]

Aganwadi Assessment form

Field	Question	Answer
location	Capture GPS location<br>*GPS coordinates can only be collected when outside.*	
worker	Worker Name	151 Abuhamza<br>152 Anmol Saini<br>153 Anshika Sahota<br>154 Ekta<br>155 Jyoti Devi<br>156 Kritika Thakur<br>157 Mehak Thakur<br>158 Poonam Devi<br>159 Riya Puri<br>160 Shivanshi<br>161 Varsha Kumari<br>162 Anchal Walia<br>163 Harshali<br>164 Kritika Puri
blocks *(required)*	Block Names	block_1 Amb<br>block_2 Thanakalan<br>block_3 Gagret<br>block_4 Haroli<br>block_5 Basdehra
circle_selected *(required)*	Circle of area	circle_1 Amb<br>circle_2 Bhaira<br>circle_3 Chaksarai<br>circle_4 Chintpurni<br>circle_5 Chururu<br>circle_6 Jubehar<br>circle_7 Kalruhi<br>circle_8 Kharoh<br>circle_9 Nandpur<br>circle_10 Nehrian<br>circle_11 Sapouri<br>circle_12 Sidhchalehar<br>circle_13 Chowki<br>circle_14 Dhundla<br>circle_15 Jasana<br>circle_16 Jol<br>circle_17 Khurwain<br>circle_18 Lathiani<br>circle_19 Piploo<br>circle_20 Raipur<br>circle_21 Thanaklan<br>circle_22 Ambota<br>circle_23 Badeda Rajputan<br>circle_24 Bhaderkali<br>circle_25 Bhanjal<br>circle_26 Gagret<br>circle_27 Ganu Madwada<br>circle_28 Ghanari<br>circle_29 Mawa Kaholan<br>circle_30 Mawa Sindhia<br>circle_31 Pirtipur<br>circle_32 Bathri<br>circle_33 Dulehar<br>circle_34 Ghaluwal<br>circle_35 Haroli<br>circle_36 Ispur<br>circle_37 Kanger<br>circle_38 Kungrat

Field	Question	Answer
		circle_39 Lalri<br>circle_40 Palakwah<br>circle_41 Panjawar<br>circle_42 Pubowal<br>circle_43 Santoshgarh<br>circle_44 Tahliwal<br>circle_45 Abada Barana<br>circle_46 Babarudru<br>circle_47 Bahdala<br>circle_48 Barnoh<br>circle_49 Basal<br>circle_50 Bhatoli<br>circle_51 Dehlan<br>circle_52 Fatehpur<br>circle_53 Ghandawal<br>circle_54 Jhalera<br>circle_55 Lower Arniala<br>circle_56 Raipur<br>circle_57 Rakkad<br>circle_58 Sanoli<br>circle_59 Una
awc_center *(required)*	Anganwadi Center	1 Amb-I<br>2 Amb-II<br>3 Amb-III<br>4 Amb-IV<br>5 Amb-V<br>6 Sham Nagar-I<br>7 Sham Nagar-II<br>8 Partap Nagar-I<br>9 Partap Nagar-II<br>10 Hira Nagar-I<br>11 Hira Nagar-II<br>12 Adarash Nagar<br>13 Andoura Upper-I<br>14 Andoura Upper-II<br>15 Andoura Upper-III<br>16 Andoura Upper Middle<br>17 Andora Gujjar Basti<br>18 Andora Lower-II<br>19 Andora Lower-III<br>20 Kuthera Kherla<br>21 Kuthera Kherla-IV<br>22 Bhaira<br>23 Bhaira-II<br>24 Bhaira Upper<br>25 Bhaira Middle<br>26 Bhaira Lower<br>27 Dhusara<br>28 Dhusara Abble<br>29 Dhusara Doam-I<br>30 Dhusara Doam-II<br>31 Saluri<br>32 Diara<br>33 Diara-II<br>34 Diara-III<br>35 Dilwan<br>36 Dilwan-II<br>37 Chhambah<br>38 Satother<br>39 Satother-II<br>40 Satother Teli Muhalla

Field	Question	Answer		
			41	Gijjar Cho
			42	Chaksrai
			43	Gathroon
			44	Jhager
			45	Ripoh Misran
			46	Ripoh Muchlian-II
			47	Danguhi
			48	Jhamber
			49	Karap Kotla
			50	Tiai
			51	Baroh
			52	Polian Parohitan
			53	Kuthera Kherla-II
			54	Kuthera Kherla-III
			55	Kherla-I
			56	Kherla-II
			57	Paloh
			58	Bharobadsar
			59	Lander Tikkari
			60	Majhar
			61	Dhar Gujaran
			62	Nari
			63	Nari 2
			64	Nari Harijan Basti
			65	Nari Chobe Basti
			66	Badhmana
			67	Badhmana-II
			68	Badhmana Harijan Basti
			69	Jawal
			70	Jawal-II
			71	Jawal Chang Basti
			72	Chhaproh
			73	Chhaproh 2
			74	Rehi
			75	Duhal Bhatwala
			76	Mirgu (Amokla Pritam)
			77	Dhalwari
			78	Dhalwari Dehlwan
			79	Chalol Behar
			80	Duhal Bangwala
			81	Paplehra
			82	Chururu-I
			83	Chururu-II
			84	Chururu-III
			85	Chururu-IV
			86	Hamboli
			87	Upper Hamboli
			88	Bandukian Da Behra
			89	Baheri
			90	Singhan Da Behra
			91	Seri-I
			92	Seri-ii
			93	Dhandri-I
			94	Dhandri-II
			95	Shiv Nagar-I
			96	Shiv Nagar-II
			97	Thathal-III
			98	Thathal-IV
			99	Thathal-V
			100	Thathal Ram nagar
			101	Kathiari Ward No. 3

Field	Question	Answer		
			102	Jubehar
			103	Saroi
			104	Jandoh
			105	Lander Landian
			106	Suri-I
			107	Suri-II
			108	Suri-III
			109	Behar Jaswan
			110	Ladial Chuk
			111	Bagru
			112	Akrot
			113	Chak
			114	Chak Bella
			115	Takarla Lower
			116	Takarla-I
			117	Takarla-II
			118	Takarla-III
			119	Gondpur
			120	Thathal Nakki
			121	Lohara Lower
			122	Bhagra
			123	Tikkari-I
			124	Tikkari-II
			125	Mubarikpur
			126	Ghebat behar
			127	Shivpur
			128	Kashipur-I
			129	Saloi
			130	Pramb
			131	Alehar
			132	Karluhi-I
			133	Karluhi-II
			134	Athwan-I
			135	Athwan-II
			136	Jhangoli
			137	Mandholi
			138	Bringal
			139	Mather
			140	Channi Devi
			141	Takoli
			142	Chowar-I
			143	Chowar-2
			144	Kwah
			145	Ghangret-I
			146	Ghangret-II
			147	Ghangret-III
			148	Gindpur
			149	Maloun-I
			150	Maloun-II
			151	Kharoh-I
			152	Kharoh-II
			153	Behar Bhater
			154	Bhater
			155	Behar-I
			156	Behar-II
			157	Baret
			158	Chanourian
			159	Harijan Basti
			160	Dharamshala Mahanta-I
			161	Dharamshala Mahanta-II
			162	Bharar Bar

Field	Question	Answer

	163	Badsla Basti
	164	Baba Nakodar Dass
	165	Nandpur-I
	166	Nandpur-II
	167	Gadiale
	168	Muhalla Lambran
	169	Thathal-I
	170	Thathal-II
	171	Kathiari
	172	Kathiari Harijan Basti
	173	Thakur Dwara
	174	Kathiari par Bela
	175	Katohar Kalan-I
	176	Katohar Kalan-II
	177	Talwal
	178	Bijapur
	179	Katohar Khurd-I
	180	Katohar Khurd-II
	181	Pucca Paroh
	182	Andora Lower-IV
	183	Badaun-I
	184	Badaun-II
	185	Bajigar Muhalla
	186	Andora lower
	187	Nehari Nauranga
	188	Nehari Khas
	189	Santo Tilla
	190	Bagga Brota
	191	Karar Behar
	192	Duhki
	193	Januhi
	194	Mairi Khas
	195	Mairi-II
	196	Mairi-III
	197	Gawalsar
	198	Panjoa Khurd
	199	Panjoa kalan
	200	Ladoli-I
	201	Thara-I
	202	Thara-II
	203	Thara-III
	204	Kangruhi
	205	Naloh Miyor
	206	Lower Poliyan Purohitan
	207	Spouri
	208	Gangoti
	209	Mughal
	210	Nouhan
	211	Amb Tilla
	212	Band Bakhshi
	213	Kaniari
	214	Ardoh
	215	Rajpur Jaswan
	216	Jaman Kuwali
	217	Maslana
	218	Guliar
	219	Kohar Chhan
	220	Basantpur
	221	Lamba sail
	222	Lahar
	223	Jawar-I

Field	Question	Answer

	224	Jawar-II
	225	Jawar Harijan Basti
	226	Patehar
	227	Lohara Upper
	228	Kotli
	229	Guret
	230	Aranwal Chahbag
	231	Thanikpur
	232	Sarda
	233	Aloh
	234	Mawa
	235	Jholan Bhatolan
	236	Chhaprohian
	237	Kashipur-II
	238	Behar
	239	Sidh Chaler
	240	Suhin
	241	Khariali
	242	Jandour-I
	243	Jandour-II
	244	Rampur-I
	245	Rampur-II
	246	Bane Di Hatti
	247	Pinjore-I
	248	Pinjore-II
	249	Chowki -1
	250	Chowki -2
	251	Chowki-4
	252	Chowki -5
	253	Beerian-1
	254	Beerian -2
	255	Beerian -3
	256	Ladila
	257	Bhaloun
	258	Bharmar
	259	Baduha -1
	260	Baduha -2
	261	Baldoh
	262	Kuder
	263	Sohari
	264	Chauli
	265	Baderah
	266	Basapatti
	267	Bhindla
	268	Baduhi-I
	269	Baduhi-Ii
	270	Baduhi-Iii
	271	Pallian
	272	Panjoda
	273	Chowki-Iii
	274	Kachhyari
	275	Amrera
	276	Sasoli
	277	Malanger
	278	Naroonh
	279	Nanawin-I
	280	Nanawin-Ii
	281	Dhundla-1
	282	Dhundla-Ii
	283	Dhundla-Iii
	284	Dhatol-I

Field	Question	Answer		
			285	Dhatol-Ii
			286	Kusan Ranauta
			287	Beri Hatli
			288	Beri-Ii
			289	Kotla
			290	Dohgi
			291	Upper Dohgi
			292	Bhugdiyan
			293	Baut
			294	Bangana
			295	Upper Nayali
			296	Bhaleti
			297	Muchhali
			298	Chilli
			299	Jakhola
			300	Jandoor
			301	Hatli
			302	Rivar
			303	Danoh
			304	Hathloun
			305	Aisan
			306	Samlara
			307	Lakhroon
			308	Majhiani
			309	Arloo
			310	Arloo Gurmukh
			311	Karor
			312	Arloo Khas
			313	Bharmot
			314	Karmali
			315	Nahri
			316	Baggi
			317	Dagru
			318	Khadol
			319	Sai
			320	Charara
			321	Badoa
			322	Raonkhar
			323	Vahi
			324	Talmera
			325	Deehar-1
			326	Deehar-2
			327	Rajpura
			328	Chadoli
			329	Buhana
			330	Chaplah
			331	Thathoon
			332	Talmet
			333	Nalwari
			334	Dumkhar
			335	Takoli-1
			336	Takoli-2
			337	Behla
			338	Jol
			339	Baslehar
			340	Harsa Jandora
			341	Bagnal
			342	Amroh
			343	Dhroon
			344	Ghaneti Mini Awc
			345	Khurwin

Field	Question	Answer		
			346	Samoor Khurd
			347	Boul
			348	Boul Har
			349	Jogi Panga
			350	Moh Khass
			351	Tyar-1
			352	Tyar-2
			353	Kubadi
			354	Ambehera Ramkishan
			355	Ambeheradeeraj
			356	Kukhera
			357	Harot
			358	Chakdoa
			359	Kud
			360	Gehra Kothi
			361	Gughan Kalan
			362	Kakrna
			363	Kyara
			364	Jagatkhana
			365	Handola-1
			366	Handola-2
			367	Kamoon
			368	Sanhal
			369	Tanda
			370	Tanoh
			371	Kaihwin
			372	Karsai
			373	Dughar
			374	Tureta
			375	Dadiyar
			376	Lathiani
			377	U.Rajli
			378	Rajli Baniyala
			379	Tyasar
			380	Bilgran
			381	Aliyana
			382	Naloot
			383	Budhan-1
			384	Budhan-2
			385	Turkal
			386	Kughal
			387	Neri
			388	Padyola
			389	Kohdra
			390	Dhret Dam
			391	Daihan
			392	Rachhoh
			393	Kheri
			394	Saroh
			395	Chamyari
			396	Basaatar
			397	Kot
			398	Jandana
			399	Dolu
			400	Jarola
			401	Hatwana
			402	Piploo
			403	Nichla Thana
			404	Ghaloon
			405	Alsaan
			406	Bhyambhi

Field	Question	Answer		
			407	Chataihar
			408	Tehi
			409	Hatli Patiyalan
			410	Marot
			411	Nargru
			412	Chamukha Mini Awc
			413	Raipur-I
			414	Raipur-Ii
			415	Raipur-Iii
			416	Androli
			417	Dobar-I
			418	Dobar-Ii
			419	Proian-I
			420	Proian-Ii
			421	Kusiala
			422	Chaugath
			423	Lidkot
			424	Chulhari
			425	Muslim Kheri
			426	Gharwasra
			427	Kyor
			428	Makrair
			429	Makrair Sidh
			430	Balh Saili
			431	Changer
			432	Anokha Tanda
			433	Bihru Kalan
			434	Nughrari
			435	Changreri
			436	Mandli
			437	Thanakalan
			438	Majher
			439	Jhorkhar
			440	Rachhol
			441	Chhaproh
			442	Boosal
			443	Narghota
			444	Balh
			445	Kholi
			446	Budwar
			447	Braal
			448	Doh
			449	Tihra-1
			450	Tihra-Ii
			451	Aghlaur
			452	Sakaun
			453	New Sakaun
			454	Dhwala
			455	Dohak
			456	Kolka
			457	Lower Chatehar
			458	Upper Chatehar
			459	Polytechnical Ambota
			460	Dawali Ambota
			461	Jogdehi
			462	Jhalowali
			463	Handiyala
			464	Parla Behra
			465	Mahila Mandal
			466	Kharasi Mohalla
			467	Lambardara Mohalla

Field	Question	Answer

	468	Panchyat Ghar
	469	Jindwad Mahila Mandal
	470	Ambota Gumma
	471	Nagnath Ambota
	472	Chatehar Sant Mohalla
	473	Matyalika Saghnai
	474	Gujjar Khad
	475	Nangal Panga
	476	Panchyat Ghar
	477	Torewala Saghnai
	478	Chang Basti
	479	Mahila Mandal
	480	Kala Panga
	481	Guglehar
	482	Upper Guglehar
	483	Mahila Mandal
	484	Thakur Dwara
	485	Lath Muhalla
	486	Upper Sarai
	487	Teli Mohalla
	488	Harizan Basti
	489	Jat Sarai
	490	Obc Mohalla
	491	Sarai Jadla Keori
	492	Jaswal Mohalla
	493	Bazar-11
	494	Dehra Mohalla
	495	Swan Mohalla
	496	Mahila Mandal
	497	Shiv Dwala Upper Nagar Wala
	498	Pathani Mohalla
	499	Dharamshala Mohalla
	500	Kolar Mohalla
	501	Kuan Bala Mohalla
	502	Ara Mohalla
	503	Sadak Mohalla
	504	Piplu-1
	505	Bandu-11
	506	Ambi-111
	507	Mahila Mandal
	508	Bhat Basti
	509	Fatehpur
	510	Brahampur-1
	511	Brahampur-11
	512	Lower Baneda-1
	513	Lower Baneda -11
	514	Dharma-111
	515	Harwal
	516	Gondpur Baneda Upper -1
	517	Baneda -11
	518	Baneda -111
	519	Upper Baneda Roda-4
	520	Kuneran-1
	521	Sanoli
	522	Kuneran-4
	523	Kuneran-11
	524	Harizan Basti
	525	Bazigar Basti
	526	Bagan Mohalla
	527	Julaha Basti

Field	Question	Answer		
			528	Bilu Di Talai
			529	Kailash Nagar -1
			530	Kailashnagar-11 Tundkhuri
			531	Hariyala Kuteda
			532	Kamali Ram Nagar-1
			533	Ram Nagar -2 Haled
			534	Amlehar-1 Rampur Kuteda
			535	Amlehar-11 Harizan Basti
			536	Amlehar-111 Sunkali
			537	Amlehar Khas
			538	Gokal Nagar
			539	Upper Bhanjal Shankar Nagar
			540	Bhanjal Upper Guga Basti
			541	Bhanjal Upper Harizan Basti
			542	Lower Bhanjal -1 Sarai
			543	Lower Bhanjal -11 Bhatwal
			544	Bada Talab
			545	Lower Bhanjal -4
			546	Lower Bhanjal-5 Kadd
			547	Jit Pur Behari-1
			548	Jitpur Behri-11
			549	Thaplan
			550	Dakha Bala Kuan
			551	Braham Sarai
			552	Lower Bhathia Wala
			553	Panwada
			554	Nai Mohalla
			555	Badoh Depot
			556	Upper Bhathia Bala
			557	Shiv Mandir Kaloh
			558	Harizan Basti
			559	Bumbaloo
			560	Kaloh Behli
			561	Chang Basti
			562	Kaloh Beli
			563	Harizan Basti
			564	Beli Gagret
			565	Brahmin Basti Gagret
			566	Panchyat Ghar
			567	Gujjar Khad
			568	Dev Nagar
			569	Purana Amb Road Gagret
			570	Bharwai Road Gagret
			571	Hanuman Mandir
			572	Purana Amb Road Chakki
			573	Pakka Paroh
			574	Shiv Mandir
			575	Raipur Mohalla
			576	Rania Talab
			577	Miidle Depot
			578	Harizan Basti
			579	Kumhar Basti
			580	Bukhaipuir
			581	Brahaman Basti
			582	Chang Pukhar
			583	Obc Mohalla
			584	Marwadi Behal
			585	Marwadi Lower
			586	Lower Madwada

Field	Question	Answer

	587	Middle Madwada
	588	Upper Madwada
	589	Tilla Takka
	590	Salohberi Samadi
	591	Lohar Muhalla
	592	Harizan Basti
	593	Salohberi Kothi
	594	Kurialai
	595	Joh Khas
	596	Panchyat Ghar
	597	Tillu Chaunta
	598	Joh Beh
	599	Joh Beh Khad
	600	Deoli Wadi
	601	Cylinder Factory
	602	Shiv Mandir Deoli
	603	Harizan Basti Deoli
	604	High School Deoli
	605	Deoli Chada Basti
	606	Tubewell Deoli
	607	Ghanari Moni Baba
	608	Ayrvedic Dispensary Ghanari
	609	Harizan Basti Ghanari
	610	Upper Ghanari
	611	Ghanri Chang Basti
	612	Upper Chang Basti
	613	Nangal Jarialan Talab
	614	Nangal Jarialan Middle School
	615	Nangal Jarialan Harizan Basti
	616	Nangal Jarialan Moru Panga
	617	Nangal Jariaan Theda
	618	Nangal Jarialan Kandi Bhavan
	619	Nangaj Jarialan Andwad
	620	Nangal Jariaalan Shivali
	621	Nangal Jarialan Jhalera
	622	Amboa Sub Centre
	623	Amboa Upper
	624	Amboa Primary School
	625	Amboa Harizan Basti
	626	Swan Par Harwall
	627	Bus Stand Mawa Kaholan
	628	Health Centre
	629	Mawa Kaholan Middle
	630	Tarali
	631	Bedha Mohalla
	632	Harizan Basti
	633	Shiv Badi
	634	Badhiakha
	635	Mahila Mandal
	636	Bagan Bala Kuan Chalet
	637	Simli Bala Kuan
	638	Pukhari Pur
	639	Mande Chalet
	640	Harizan Basti
	641	Girl School
	642	Tapriala

Field	Question	Answer		
			643	Daulatpur Chowk
			644	Dav Daulatpur Chowk
			645	Dholwaha Road
			646	Kua Devi
			647	Tuta Bala Kuan
			648	Bai Bhatha
			649	Babehad Mahila Mandal
			650	Middle School
			651	Lawana Mohalla
			652	Panchyat Ghar
			653	Maralu Mohalla
			654	Khad Gujaran
			655	Lambar Dar Mohalla
			656	Obc Mohalla
			657	Upper Gram
			658	Sc Basti
			659	Upper Society
			660	Seth Coloney
			661	Upper Khad Gujaran
			662	Bus Stand
			663	Upper Sarai Tateda
			664	Panchyat Ghar
			665	Factory Coloney
			666	Sc Basti
			667	Obc Mohalla
			668	Nepali Coloney
			669	Mahila Mandal
			670	Oel Ashram
			671	Upper Sarai
			672	Harizan Basti
			673	Haroti Mohalla
			674	Pirthipur Faquti
			675	Mahila Mandal
			676	Maidangarh
			677	Harizan Basti
			678	Lohar Basti
			679	Bank Ghar
			680	Pirthipur Chaua
			681	Pirthipur Khas
			682	Dangoh Khurd
			683	Upper Pirthipur
			684	Gujjar Khad Dangohkhas
			685	Setha Mohalla
			686	Mahila Mandal
			687	Dangoh Pirthipur Road
			688	Harizan Basti
			689	Gumma Dangohkhas
			690	Mohalla Kavirpanthi
			691	Dodua Dangoh
			692	Abhaypur Chang Basti
			693	Abhaypur Upper
			694	Abhaypur Middle
			695	Bathri (Present)
			696	Bathri I (Present)
			697	Lohar Muhalla
			698	Bahti Muhalla
			699	Rajput Muhalla
			700	Changare Muhalla
			701	Brahmin Muhalla
			702	Beetan (Present)
			703	Jakhewal (Present)

Field	Question	Answer

	704	Jakhewal I
	705	Gujjar Basti I
	706	Gujjar Basti Iii
	707	Gujjar Basti Ii
	708	Harijan Muhalla
	709	Lohar Muhalla
	710	Singan Present
	711	Singan I
	712	Havelli Vala
	713	Brahmin Muhalla
	714	Rajput Muhalla
	715	Harijan Basti
	716	Kotli Vala
	717	Pangyan Vala
	718	Heera Nagar
	719	Sahoowal 1
	720	Sahowal Ii
	721	Rajputan Polician
	722	Brahman Muhalla
	723	Lohar Muhalla
	724	Dulehar
	725	Gaua
	726	Muslim Muhalla
	727	Mishra Muhalla
	728	Beebar Muhalla
	729	Bhagtan Muhalla
	730	Harijan Muhalla
	731	Brahman Muhalla
	732	Gondpur Jai Chand 1
	733	Gondpur Jai Chand Ii
	734	Gondpurjaichand Iii
	735	Kabir Panthi Muhalla
	736	Beldar Basati
	737	Brahman Muhalla
	738	Rajput Tarkhan Muhalla
	739	Gondpur Bullan 1
	740	Gandpur Bullan Ii
	741	Brahman Muhalla I
	742	Brahman Muhalla Ii
	743	Gongpur
	744	Pathak Muhalla
	745	Bank Ghar
	746	Harijan Basti
	747	Saini Basti
	748	Panchayat Ghar
	749	Bhadsali Harijan Basti
	750	Bagru Muhalla
	751	Bhuliyan Muhalla
	752	Manguwal Muhalla
	753	Shah Muhalla
	754	Bhadsali Varatmaan
	755	Bhadsali Haar
	756	Kalla Muhalla
	757	Bada Haar
	758	Chota Haar
	759	Jejon Moad
	760	Saloh Bhatta
	761	Jejon Moad Ii
	762	Ghaluwal Bazar
	763	Kyar Muhalla
	764	Saloh Haar

Field	Question	Answer		
			765	Harijan Basti
			766	Saloh Mahadev I
			767	Saloh Mahadev Ii
			768	Shiv Mandir Saloh
			769	Dharampur Lower
			770	Gurduara Basti
			771	Sidh Channo Mandir
			772	Bada Beda
			773	Shiv Mandir
			774	Sansowal (Present)
			775	Samnal (Present)
			776	Kuruwala Samnal
			777	Bati Muhalla
			778	Harijan Basti
			779	Rahar Muhalla
			780	Main Bazar Samnal
			781	Rora (Present)
			782	Nichla Mazra
			783	Chande Muhalla
			784	Palaki Vala
			785	Haroli I(Present)
			786	Haroli Ii
			787	Roleyan Muhalla
			788	Harijan Basti
			789	Lohar Basti
			790	Bati Muhalla Dhol
			791	Haar Khera
			792	Kali Badi
			793	Saini Bati Muhalla
			794	Haijan Basti Khad I
			795	Harijan Basti Ii
			796	Jangle Panehra
			797	Pandoga Barrier
			798	Upper Pandoga
			799	Pandoga Lower
			800	Pandoga Kharoian Muhalla
			801	Pandoga Barrier Ii
			802	Chakk Muhalla Pandoga
			803	Tahlia Muhalla
			804	Atava Muhalla
			805	Pandoga Pathak Muhalla
			806	Saini Muhalla
			807	Jole Harijan Basti
			808	Pandoga Kyarian Muhalla
			809	Bag Bharwal Muhalla
			810	Brahman Basti
			811	Ispur Hoshiarpur Road
			812	Lawana Majra I
			813	Ispur 'Gagret Road
			814	Labana Majra -Ii
			815	Pathak Muhalla
			816	Ravidas Mandir
			817	Saruain Bag
			818	Tippar Muhalla
			819	Saloh Harijan Basti I
			820	Purian Muhalla
			821	Sr Sec. School Saloh
			822	Bholian Muhalla
			823	Jaat Muhalla
			824	Upper Badehra Phc
			825	Middle School Badehra

Field	Question	Answer

	826	Upper Harijan Basti
	827	Upper Lohar Muhalla
	828	Katwal Muhalla
	829	Thakaran Muhalla
	830	Lavana Muhalla
	831	Gujjar Pahari Muhalla
	832	Lower Badera
	833	Shiv Mandir Badhera
	834	Jatt Muhalla
	835	Bhai Da Moad
	836	Jaswal Muhalla
	837	Upper Kanger
	838	Lower Kanger
	839	Bakralu Muhalla
	840	Kanger Haar
	841	Bharwal Muhalla
	842	Harijan Basti
	843	Dharampur (Present Upper)
	844	Harijan Basti
	845	Brahmin Satta Muhalla
	846	Malluwal Purana
	847	Polia Beet I
	848	Janni I
	849	Majra Jhole
	850	Polian Beet Ii
	851	Polian Iii
	852	Janni Ii
	853	Chhetran I
	854	Biderwal
	855	Harijan Dita
	856	Tarkhan Muhalla
	857	Khatri Muhalla
	858	Chhetran Ii
	859	Kungrat Vartman
	860	Rana Muhalla
	861	Ambi Muhalla
	862	Chowki Muhalla
	863	Bhandiara Ii
	864	Brahmin Kash Muhalla
	865	Bhandiara I
	866	Lalri I(Present)
	867	Lalri Ii (Present)
	868	Lalri Iii (Present)
	869	Lalri Iv (Present)
	870	Kelian Muhalla
	871	Brahmin Muhalla
	872	Dhanaru Muhalla
	873	Bati Muhalla
	874	Jhave Muhalla
	875	Bakralu Muhalla
	876	Tarkhan Muhalla
	877	Bharwal Muhalla
	878	Brahmin, Bati Muhalla
	879	Bati, Saini Muhalla
	880	Lohar Bati Muhalla
	881	Kalehra (Present)
	882	Saini Chuhowal Muhalla
	883	Kalehra Ii
	884	Khatta Muhalla
	885	Heera I (Present)

Field	Question	Answer		
			886	Heera Ii (Present)
			887	Loothre Muhalla
			888	Dhugge Muhalla
			889	Subboana
			890	Bhadhori (Present)
			891	Lohar Muhalla
			892	Brahmin Harijan Muhalla
			893	Bati Muhalla
			894	Nichli Jatt Basti
			895	Upper Palakwah
			896	Kante Varatman
			897	Jatt Muhalla
			898	Brahmin Muhalla
			899	Pandit Tarkhan, Naee, Lavana Muhalla
			900	Harijan Muhalla
			901	Tarkhan Muhalla
			902	Kumhaar Muhalla
			903	Bati Muhalla
			904	Gill Behra Thakra
			905	Thakaran Muhalla
			906	Gangtho (Present)
			907	Sutre Saini Muhalla
			908	Pulvari Muhalla
			909	Karampur
			910	Tholle Muhalla
			911	Kharwal Muhalla
			912	Karampur Lower
			913	Nangnoli (Present)
			914	Nangnoli (Haar)
			915	Lavana Majra
			916	Harijan Basti
			917	Panjawar I (Present)
			918	Panjawar Ii
			919	Jole I
			920	Panjawar Iii
			921	Jangle Muhalla
			922	Khatri Basti
			923	Jole Ii
			924	Tarkhana Mohalla
			925	Thhakki I
			926	Daulatpur Haar
			927	Thhakki Ii
			928	Upper Daulatpur
			929	Sain Basti
			930	Main Baazar Panjawar
			931	Master Muhalla
			932	Khad Present
			933	Bhaini Muhalla
			934	Hoshiarpur Road Khad
			935	Swanpar Malagarh
			936	Khad Centre
			937	Dangewala Muhalla
			938	Pubowal (Present)
			939	Pubowal (Center)
			940	Harijan Basti I
			941	Gurudwara Basti
			942	Harijan Basti Ii
			943	Padyan Muhalla
			944	Ramsar Muhalla
			945	Baliwal

Field	Question	Answer

	946	Jatt Muhalla
	947	Brahmin Muhalla
	948	Harijan Muhalla
	949	Rajput Muhalla
	950	Panjuana I
	951	Panjuana Ii
	952	Pubowal Ii
	953	Badewala
	954	Gurudwara Muhalla Bilna
	955	Halera
	956	Kuthar I
	957	Makkorgarh
	958	Aperlipali
	959	Mishra Brahmin Muhala
	960	Kutharbeet Ii
	961	Kumhar Kabir Panthi Muhalla
	962	Ward No.I Santoshgarh
	963	Ward No.Ii Santoshgarh
	964	Ward No.Iii Santoshgarh
	965	Ward No.Iv Santoshgarh
	966	Ward No.V Santoshgarh
	967	Ward No.Vi Santoshgarh
	968	Ward No.Vii Santoshgarh
	969	Ward No.Ix Santoshgarh
	970	Ward No Viii Santoshgarh
	971	Ward No I+Ii Santoshgarh
	972	Ward No Vii, Viii, Ix
	973	Chhaterpur(Present)
	974	Bahti Muhalla
	975	Dada
	976	Bathu (Present)
	977	Gurplah (Present)
	978	Gurplah Ii
	979	Harijan Basti I
	980	Harijan Basti Ii
	981	Kelluan Muhalla
	982	Lamber Luvana Muhalla
	983	Upperla Muhalla
	984	Morvadi I
	985	Morvadi Ii
	986	Bathu Khas
	987	Nangal Kalan (Present)
	988	Jattpura (Present)
	989	Harijan Basti I
	990	Bharare Mohalla
	991	Brahmin Mohalla
	992	Jattpura Ii
	993	Nangal Kalan Ii
	994	Rajput Muhalla (Nangal Kalan)
	995	Tahliwal
	996	Nangal Khurd(Present)
	997	Manuwal
	998	Upper Manuwal
	999	Tibba Muhalla
	1000	Bahti Muhalla I
	1001	Bahti Muhalla Ii
	1002	Upper Rajput Muhalla
	1003	Lower Rajput Muhalla
	1004	Kiduan Muhalla

Field	Question	Answer

	1005	Batkalan (Present)
	1006	Upper Basti I
	1007	Upper Basti Ii
	1008	Nichli Basti
	1009	Saini Basti
	1010	Abada Barana
	1011	Abada Barana-Lohar Basti
	1012	Abada Barana-Brahman Khatri Mohalla
	1013	Jankaur
	1014	Barsada
	1015	Barsada-Bahti Jat Mohalla
	1016	Jankaur Saini Mohalla
	1017	Jankaur Tarkhan Mohalla
	1018	Sunehara
	1019	Sunehara-Kabir Panthi Moh.
	1020	Nangran
	1021	Nangran-Harijan Moh.
	1022	Nangran-Bahati Moh.
	1023	Nangran-Bahati Moh.-2
	1024	Nangran-Bahati Moh.-3
	1025	Nangran-Bahati Moh.-4
	1026	Nangran-Bahati Moh.-5
	1027	Nangran-Bahati Moh.-6
	1028	Badehar
	1029	Badehar-Swar Nai-1
	1030	Badehar-Swar Nai-2
	1031	Badehar-Chilawala
	1032	Jhurowal-1
	1033	Jhurowal-2
	1034	Khui Pekhu Bela
	1035	Jhurowal Bahati Moh.
	1036	Jhurowal Bahati Moh.-2
	1037	Nangal Slangri-1
	1038	Lehad
	1039	Parla Sanjhot
	1040	Nangal Salangri-2
	1041	Sanjhot
	1042	Nari
	1043	Nari- Lower -1
	1044	Nari- 2
	1045	Dhadhial-2
	1046	Dhadhial-
	1047	Nari-3
	1048	Chalola
	1049	Chalola-2
	1050	Chalola-3
	1051	Dhamandri-1
	1052	Dhamandri-2
	1053	Dhamandri-Satleta
	1054	Dhamandri-Mansoh
	1055	Dhamandri-3
	1056	Dhamandri-4
	1057	Dathwada
	1058	Dathwara-2
	1059	Barera
	1060	Barera-2
	1061	Behdala-1
	1062	Behdala-2
	1063	Vasdev Khidri Mohalla

Field	Question	Answer

	1064	Behdala-Harijan Mohalla-1
	1065	Behdala-Harijan Mohalla-2
	1066	Behdala-Khidri Mohalla
	1067	Vasdev Harijan Mohalla
	1068	Behdala-Rajput Mohalla-1
	1069	Behdala-Rajput Mohalla-2
	1070	Behdala-Rajput Mohalla-3
	1071	Behdala-Valmiki Mohalla-1
	1072	Behdala-Valmiki Mohalla-2
	1073	Chatara
	1074	Chatara Mahadev
	1075	Chatara Brahaman Mohalla
	1076	Chatara Harijan Mohalla
	1077	Chatara Lohar Mohalla
	1078	Chatara Khatri Mohalla
	1079	Chatara Brahaman Mohalla-2
	1080	Chatara Labana Mohalla
	1081	Bharolian Kalan
	1082	Bharolian Kalan Jhingla Behda
	1083	Bharolian Kalan Bade Wala Mohalla
	1084	Bharolian Kalan Harijan Mohalla
	1085	Barnoh
	1086	Barnoh Jat Saini Mohalla
	1087	Dangera-1
	1088	Dangera-2
	1089	Dangoli
	1090	Dangoli Tarkhan Mohalla
	1091	Dangoli Harijan Mohalla
	1092	Dangoli Brahaman Mohalla
	1093	Dangoli Jatt Mohalla
	1094	Samoor
	1095	Bhaur
	1096	Samoor Bhaur Brahaman Mohalla
	1097	Samoor Bhaur Saur Mohalla
	1098	Samoor Bhaur Chhalwad Mohalla
	1099	Kuriala-1
	1100	Kuriala Bhaur
	1101	Kuriala Haled
	1102	Kuriala-2
	1103	Jhambar-1
	1104	Jhambar Chilliyan Harijan
	1105	Sarjehra
	1106	Laam
	1107	Jhambar Lower
	1108	Basal Upper
	1109	Basal Harijan Basti
	1110	Basal Brahaman Basti
	1111	Basal Khwaja Basti-1
	1112	Basal Bada Behda
	1113	Basal Bugde Bablu
	1114	Basal Khwaja Basti-2
	1115	Lower Basal
	1116	Lower Basal Harijan Basti

Field	Question	Answer		
			1117	Lower Basal Dhiman Bahati Mohalla
			1118	Lower Basalbahati Mohalla-2
			1119	Lower Basalbahati Mohalla-3
			1120	Lower Basalbahati Mohalla-4
			1121	Takka Bishna
			1122	Takka Ramsahay
			1123	Takka Bishna Saini Mohalla
			1124	Takka Bahati Mohalla
			1125	Takka Harijan Mohalla
			1126	Takka Ramsahay Saini Moh.
			1127	Takka Ramsahay Brahaman Harijan Moh.
			1128	Kotla Khurd
			1129	Kotla Khurd Khatri Mohalla
			1130	Kotla Khurd Lohar Mohalla
			1131	Bhatoli -1
			1132	Bhatoli -2
			1133	Bhatoli Khrtikre Mohlla
			1134	Bhatoli Chirbe Julahe Mohlla
			1135	Morbar-1
			1136	Morbar-2
			1137	Bhatoli Jatt Moh.-1
			1138	Bhatoli Jatt Moh.-2
			1139	Bhatoli Harijan Basti-1
			1140	Bhatoli Harijan Basti-2
			1141	Jakhera -1
			1142	Jakhera -2
			1143	Jakhera Hari Basti
			1144	Jakhera Basdev Moh.
			1145	Jakhera Brahman Moh.-1
			1146	Jakhera Brahman Moh.-2
			1147	Jakhera Balmiki Moh.
			1148	Bangrah Purana Kander
			1149	Fatewal
			1150	Bangrah -1
			1151	Bangrah -2
			1152	Bangrah Pukhru Moh.
			1153	Dehlan-Upper-1
			1154	Dehlan-Upper-2
			1155	Uppar Dehlan Mahldarji-1
			1156	Uppar Dehlan Mahldarji-2
			1157	Uppar Dehlan Negi Budu Mohlla
			1158	Uppar Dehlan Harjin Basti
			1159	Uppar Dehlan Grewal Mohlla
			1160	Uppar Dehlan Kavir Panthi Nai Mohlla
			1161	Upper Dehlan Bans Bansre Moh.
			1162	Uppar Dehlan Bade Wale Mohalla
			1163	Lower Dehla-1
			1164	Lower Dehla-2
			1165	Lower Dehla-3

Field	Question	Answer		
			1166	Lower Dehlan Tarkhan Moh.
			1167	Lower Dehlan Bahti Moh.
			1168	Lower Dehlan Harijan Basti
			1169	Lower Dehlan Brahman Moh.
			1170	Lower Dehlan Bahti Moh.-1
			1171	Lower Dehlan Bahti Moh.-11
			1172	Lower Dehlan Bahti Moh.-111
			1173	Lower Dehlan Bats Tikre
			1174	Mehtpur
			1175	Mehatpur Dwedi Mohalla
			1176	Shri Lanka Mohlla Mehtpur
			1177	Fateh Pur-1
			1178	Fateh Pur Harijan Basti-1
			1179	Fateh Pur Harijan Basti-2
			1180	Fateh Pur Bahati Mohalla
			1181	Fateh Pur -2
			1182	Khanpur-1
			1183	Khanpur Bahati Saini Mohalla
			1184	Khanpur Bahati Mohalla
			1185	Khanpur Harijan Basti
			1186	Khanpur -2
			1187	Uday Pur
			1188	Uday Pur Dhiman/ Rajput Mohalla
			1189	Sasan
			1190	Sasan Harijan Basti
			1191	Sasan Bahati Mohalla
			1192	Sasan Jatt Mohalla
			1193	Charat Garh-1
			1194	Charat Garh-2
			1195	Charat Garh-Lahar Mohalla
			1196	Charat Garh-Aeri Mohalla
			1197	Charat Garh-Harijan Basti
			1198	Charat Garh-Bajit Pur
			1199	Kuthar Kalan
			1200	Kuthar Kalan Bahati Mohalla
			1201	Kuthar Kalan Tarkhan/Rajput Mohalla
			1202	Tyuri-1
			1203	Tyuri-2
			1204	Tyuri-3
			1205	Panoh-1
			1206	Panoh-2
			1207	Panoh-3
			1208	Bhalola
			1209	Baduhi
			1210	Baduhi-1
			1211	Bhaloh
			1212	Ghandawal-1
			1213	Ghandawal-2
			1214	Badoli-1
			1215	Badoli Hari Basti
			1216	Badoli-2

Field	Question	Answer

	1217	Badsala
	1218	Badsala Khambuya Da Mohlla
	1219	Badsala Bankeya Da Mohlla
	1220	Jhalera Upper
	1221	Jhalera Lower
	1222	Jhalera Banga Bala Mohlla
	1223	Jhlera Partap Mohlla
	1224	Jhlera New Beli Kloni
	1225	Rampur-1
	1226	Rampur-2
	1227	Rampur Jatt Mohlla
	1228	Rampur Harijan Mohlla
	1229	Rampur Bahti Brahman Mohlla
	1230	Kuthar Harijan Ghabre Mohlla
	1231	Kuthar Khurd Brahman Bati Mohlla
	1232	Kuthar Khurd
	1233	Lal Singi
	1234	Lal Singi Rajput Brahman Mohlla
	1235	Lal Singi Rajputsaini Mohlla
	1236	Rainsari Mdhey
	1237	Rainsari Purb
	1238	Rainsari Brahman Mohlla
	1239	Rainsari Harijan Moh.
	1240	Rainsari Bahti Moh.-1
	1241	Rainsari Brahman Jatt Moh.
	1242	Rainsari Bahti Mohlla-2
	1243	Lower Arniala
	1244	Lower Arniala Sharma Saini Mohalla
	1245	Lower Arniala Bahati Saini Mohalla
	1246	Upper Arniala
	1247	Upper Arniala Saini Harijan Mohalla
	1248	Upper Arniala Adarsh Nagar
	1249	Upper Arniala Rajput Lohar Mohalla
	1250	Upper Arniala Jhangri Mohalla
	1251	Upper Arniala Tarkhan Rajput Mohalla
	1252	Lower Kotla Kalan
	1253	Lower Kotla Kalan Saini Mohalla
	1254	Lower Kotla Kalan Brahaman Mohalla
	1255	Lower Kotla Kalan Tarkhan Mohalla
	1256	Upper Kotla Kalan
	1257	Upper Kotla Kalan Brahaman Mohalla
	1258	Upper Kotla Kalan Lohar Mohalla

Field	Question	Answer

	1259	Upper Kotla Kalan Tarkhan Mohalla
	1260	Upper Kotla Kalan Harijan Mohalla
	1261	Ajnoli
	1262	Ajnoli Saini Mohalla
	1263	Ajnoli Brahman Sood Mohalla
	1264	Ajnoli Upper
	1265	Lamlahri
	1266	Lamlahri Brahman Mohalla
	1267	Lamlahri Upper
	1268	Lamlahri Badla Mohalla
	1269	Raypur-1
	1270	Raypur-2
	1271	Raypur-3
	1272	Raypur-4
	1273	Raypur Buje Bahti Mohlla
	1274	Raypur Hatti Bale Mohlla
	1275	Raypur Tarkhan Mohlla
	1276	Raypur Gabla Mohlla
	1277	Raypur Mehar Mohlla
	1278	Raypur Braman Mohlla
	1279	Raypur Jatt Behra
	1280	Raypur Bhatha Lekhraj
	1281	Basdera Kendr No.13
	1282	Basdera Kendr No.14
	1283	Basdera Kendr No.15
	1284	Basdera Kendr No.16
	1285	Basdera Kendr No.17
	1286	Basdera Kendr No.18
	1287	Basdera Bard No.3+5
	1288	Basdera Bard No.6
	1289	Basdera Bard No.9+8
	1290	Basdera Bard No.1
	1291	Lamlehra Purana
	1292	Lamlehra Brahman Mohlla
	1293	Lamlehra -2
	1294	Madan Pur
	1295	Madan Pur-Saini Mohalla
	1296	Madan Pur-2
	1297	Basoli-1
	1298	Basoli-2
	1299	Basoli-Dhesi Jat Mohalla
	1300	Basoli-Bhat Jat Mohalla
	1301	Basoli-Brahaman Mohalla
	1302	Basoli-Dhiman Mohalla
	1303	Basoli-Nala Mohalla
	1304	Malahat
	1305	Bharolian Khurd
	1306	Parli Patti Malahat
	1307	Brahaman Patti Malahat
	1308	Harijan Patti Malahat
	1309	Rajput Patti Malahat
	1310	Bharolian Khurd Brahanana-1
	1311	Bharolian Khurd Brahanana-2
	1312	Tabba-2
	1313	Rakkar
	1314	Tabba Rajput Mohalla-2

Field	Question	Answer
		1315 Tabba Rajput Mohalla-3<br>1316 Tabba Harijan Mohalla<br>1317 Tabba Lohar Mohalla<br>1318 Tabba -1<br>1319 Sanoli-1<br>1320 Sanoli-2<br>1321 Sanoli-Rajput Jat Mohalla-1<br>1322 Sanoli-Rajput Jat Mohalla-2<br>1323 Sanoli-Rajput Mohalla<br>1324 Sanoli-Harijan Mohalla-1<br>1325 Sanoli-Harijan Mohalla-2<br>1326 Sanoli-Brahaman Mohalla<br>1327 Majara<br>1328 Majara Jat Mohalla-1<br>1329 Majara Jat Mohalla-2<br>1330 Majara Jat Mohalla-3<br>1331 Malukpur<br>1332 Malukpur Jat Mohalla<br>1333 Binewal<br>1334 Puhna-1<br>1335 Puhna-2<br>1336 Puhna-3<br>1337 Ajoli<br>1338 Ajauli Brahman Mohalla-1<br>1339 Ajauli Brahman Mohalla-2<br>1340 Ajauli Harijan Basti<br>1341 Ajauli Bahati Jat Mohalla-1<br>1342 Ajauli Bahati Jat Mohalla-2<br>1343 Prem Nagar<br>1344 Gursar Mohalla<br>1345 Vikas Nagar<br>1346 Vivek Nagar<br>1347 Pulwala Bazar<br>1348 Purana Dakkhana<br>1349 Shiv Nagar<br>1350 Nagraj Mohalla<br>1351 Dc Colony<br>1352 Behli Mohalla-<br>1353 Neela Ghat<br>1354 Sabji Mandi W.No-1<br>1355 W.No-1 Centre-2<br>1356 Galua-1<br>1357 Galua-2<br>1358 Ward (2+7)<br>1359 Ward (4+8)<br>1360 Behli Mohalla-2<br>1361 Ward 7&11 Centre-2<br>1362 Chanderlok Colony<br>1363 Neelaghat Colony<br>1364 Friends Colony
ANGANWADI		
ANGANWADI > Anganwadi Centre Identification		
a_1	Date of Assessment:<br>*Response constrained to: . = today()*	
a_6 *(required)*	Anganwadi Centre id:<br>*Response constrained to: regex(., '^[0-9]{10}$')*	
a_7 *(required)*	Anganwadi Centre Address (Village, Block):	
ANGANWADI > Anganwadi Centre Identification > facility_details		
fac_1	Name and address, Nearest HWC-SHC	

<table>
<tr><th>Field</th><th>Question</th><th>Answer</th></tr>
<tr><td>fac_2 (required)</td><td>Name of HWC-SHC
Fill NA if data is not available
Response constrained to: not(regex(.,'^(.*)\d(.*)$'))</td><td></td></tr>
<tr><td>fac_3 (required)</td><td>Address of HWC-SHC
Fill NA if data is not available</td><td></td></tr>
<tr><td>fac_4 (required)</td><td>Distance of nearest HWC-SHC
Please enter the value in decimal</td><td></td></tr>
<tr><td>fac_5</td><td>Name and address ,Nearest PHC</td><td></td></tr>
<tr><td>fac_6 (required)</td><td>Name of PHC
Fill NA if data is not available
Response constrained to: not(regex(.,'^(.*)\d(.*)$'))</td><td></td></tr>
<tr><td>fac_7 (required)</td><td>Address of PHC
Fill NA if data is not available</td><td></td></tr>
<tr><td>fac_8 (required)</td><td>Distance of nearest PHC
Please enter the value in decimal</td><td></td></tr>
<tr><td>fac_9</td><td>Name and address ,Nearest CHC</td><td></td></tr>
<tr><td>fac_10 (required)</td><td>Name of CHC
Fill NA if data is not available
Response constrained to: not(regex(.,'^(.*)\d(.*)$'))</td><td></td></tr>
<tr><td>fac_11 (required)</td><td>Address of CHC
Fill NA if data is not available</td><td></td></tr>
<tr><td>fac_12 (required)</td><td>Distance of nearest CHC
Please enter the value in decimal</td><td></td></tr>
<tr><td>fac_9_ch</td><td>Name and address ,Nearest CH</td><td></td></tr>
<tr><td>fac_10_ch (required)</td><td>Name of CH
Fill NA if data is not available
Response constrained to: not(regex(.,'^(.*)\d(.*)$'))</td><td></td></tr>
<tr><td>fac_11_ch (required)</td><td>Address of CH
Fill NA if data is not available</td><td></td></tr>
<tr><td>fac_12_ch (required)</td><td>Distance of nearest CH
Please enter the value in decimal</td><td></td></tr>
<tr><td colspan="3">ANGANWADI > Anganwadi Centre Identification > aganwadi_details</td></tr>
<tr><td>a_11 (required)</td><td>Total Population Covered by the Anganwadi Centre:</td><td></td></tr>
<tr><td>a_12 (required)</td><td>Anganwadi Worker Name:
Response constrained to: not(regex(.,'^(.*)\d(.*)$'))</td><td></td></tr>
<tr><td>a_13 (required)</td><td>Anganwadi Worker Contact No.:
Response constrained to: regex(.,"^[6|7|8|9]\d{9}$")</td><td></td></tr>
<tr><td>a_14 (required)</td><td>ICDS Supervisor Name:
Response constrained to: not(regex(.,'^(.*)\d(.*)$'))</td><td></td></tr>
<tr><td>a_15 (required)</td><td>ICDS Supervisor Contact No.:
Response constrained to: regex(.,"^[6|7|8|9]\d{9}$")</td><td></td></tr>
<tr><td colspan="3">ANGANWADI > Infrastructure Status</td></tr>
<tr><td>b_1 (required)</td><td>Located within Main Habitation (*where the majority of the population resides)
If No, Distance in Km</td><td>1 Yes
2 No</td></tr>
<tr><td>b_2 (required)</td><td>Connected to Motorable Road</td><td>1 Yes
2 No</td></tr>
<tr><td>b_3 (required)</td><td>Designated Government aganwadi centre Building</td><td>1 Yes
2 No
99 Other</td></tr>
<tr><td>b_4 (required)</td><td>General Structure of Building</td><td>1 Newly Built
2 Newly Renovated
3 Old Structure
4 Falling apart</td></tr>
<tr><td>b_5 (required)</td><td>Maintenance of Building</td><td>1 No maintenance
2 Once a year
3 Once in 3 years
99 Other (specify)</td></tr>
<tr><td>b_6 (required)</td><td>Is electrical supply available?</td><td>1 24/7
3 Average duration of electricity supply per day
2 No Backup</td></tr>
<tr><td>b_6_or</td><td>Is power backup available?</td><td>1 Generator
2 Inverter
3 Solar
99 Other source please specify</td></tr>
</table>

Field	Question	Answer
		4 No Backup
b_7 *(required)*	Main source of water supply?	1 Borewell<br>2 Piped water<br>99 Other with duration
b_8 *(required)*	Type of toilet facility available	1 Yes<br>2 No
b_8_1_others *(required)*	Littering	1 Yes<br>2 No
ANGANWADI > anganwadi_toilet		
b_8_1 *(required)*	Facility available inside the toilet<br>*Question relevant when: selected(${b_8} , '1')*<br>*Response constrained to: not(selected(${b_8_1} , '9999') and count-selected(${b_8_1}) > 1)*	1 Cleanliness<br>2 Toilet tap with running water<br>3 Flush working properly<br>4 Water bucket and mug inside the toilet<br>5 Washbasin with running water<br>6 Soap/liquid items handwash/hand rub<br>7 Disable friendly<br>8 Dustbin with yellow line in (female washroom)<br>9999 None
b_8_toilet *(required)*	Gender specific toilet facility<br>*Question relevant when: selected(${b_8} , '1')*	1 Both (male & female separate) Available<br>2 Only Male Available<br>3 Only Female Available<br>4 Common Toilet Available<br>5 Availability of a disabled-friendly toilet
ANGANWADI > anganwadi_infra2		
b_8_2 *(required)*	Rooms available<br>*Response constrained to: .>= 0 and .<=5*	
b_8_3 *(required)*	Separate kitchen space for cooking	1 Yes<br>2 No
b_8_4 *(required)*	Storage facility for food	1 Yes<br>2 No
b_8_4_1 *(required)*	Storage facility for equipment	1 Yes<br>2 No
b_8_5 *(required)*	Space for at least 20 beneficiaries	1 Yes<br>2 No
ANGANWADI > anganwadi_infra3		
b_8_5_1 *(required)*	How many beneficiaries can be accomodated comfortably?<br>*Question relevant when: ${b_8_5} =2*<br>*Response constrained to: .>= 0 and .<=150*	
ANGANWADI > anganwadi_infra3 > accomodated_group		
reserved_name_for_field_list_labels_79		1 Yes<br>2 No
b_8_6_1 *(required)*	Play area for children	1 Yes<br>2 No
b_8_6_2 *(required)*	Adequate table	1 Yes<br>2 No
b_8_6_3 *(required)*	Adequate Bench	1 Yes<br>2 No
b_8_6_4 *(required)*	Adequate Stool	1 Yes<br>2 No
b_8_6_5 *(required)*	Adequate Chairs	1 Yes<br>2 No
b_8_6_6 *(required)*	Adequate Floor mat available	1 Yes<br>2 No

Field	Question	Answer
b_8_9 *(required)*	Other (Specify)	
b_8_10 *(required)*	Is PALNA scheme operational at the AWC?	1 Yes<br>2 No
ANGANWADI > Human Resource & Training Status for last one year		
c_1 *(required)*	Type of Worker - HR & Training status	1 Anganwadi Worker<br>2 Anganwadi Helper<br>99 Other (Specify)
ANGANWADI > Human Resource & Training Status for last one year > [c_1_count1] (1)		(Repeated group)
c_2 *(required)*	Sanctioned (no.)<br>*Response constrained to: . >= 0 and . <= 2*	
c_3 *(required)*	In-position (No.)<br>*Response constrained to: . >= 0 and . <= ${c_2}*	
ANGANWADI > Human Resource & Training Status for last one year > [c_1_count1] (1) > Status (1)<br>*Group relevant when: ${c_3} > 0*		(Repeated group)
c_4 *(required)*	Available/Long leave/Deputed somewhere else	1 Available<br>2 Long leave<br>3 Deputed Somewhere else
ANGANWADI > Human Resource & Training Status for last one year > [c_1_count1] (1) > Training Status (1)		(Repeated group)
ANGANWADI > Human Resource & Training Status for last one year > [c_1_count1] (1) > Training Status (1) > hr_training_status1<br>*Group relevant when: ${c_3} > 0*		
reserved_name_for_field_list_labels_102		1 Yes<br>2 No
c_5 *(required)*	Training Status - Nutrition screening and counseling	1 Yes<br>2 No
c_6 *(required)*	Training Status - Growth monitoring	1 Yes<br>2 No
c_7 *(required)*	Training Status - Micronutrient supplementation	1 Yes<br>2 No
c_8 *(required)*	Training Status - Hygiene maintenance and hand washing	1 Yes<br>2 No
c_9 *(required)*	Training Status - Poshan abhiyan application supplementary nutrition program	1 Yes<br>2 No
ANGANWADI > Equipment Availability Status		
d_1 *(required)*	Adult Weighing Scale (Digital)<br>*If Yes, Number of Functional Equipment(s)*	1 Yes<br>2 No
d_1_a *(required)*	Adult Weighing Scale (Analog)<br>*If Yes, Number of Functional Equipment(s)*	1 Yes<br>2 No
d_2 *(required)*	Stadiometer<br>*If Yes, Number of Functional Equipment(s)*	1 Yes<br>2 No
d_3 *(required)*	Measuring Tape<br>*If Yes, Number of Functional Equipment(s)*	1 Yes<br>2 No
d_4 *(required)*	Baby Weighing Scale(Digital)<br>*If Yes, Number of Functional Equipment(s)*	1 Yes<br>2 No
d_4_a1 *(required)*	Baby Weighing Scale(Analog)<br>*If Yes, Number of Functional Equipment(s)*	1 Yes<br>2 No
d_5 *(required)*	Infantometer<br>*If Yes, Number of Functional Equipment(s)*	1 Yes<br>2 No
d_6 *(required)*	Clinical Thermometer<br>*If Yes, Number of Functional Equipment(s)*	1 Yes<br>2 No
d_6_t *(required)*	Digital Thermometer<br>*If Yes, Number of Functional Equipment(s)*	1 Yes<br>2 No
d_7 *(required)*	Mid-upper-arm-circumference (MUAC) Tape<br>*If Yes, Number of Functional Equipment(s)*	1 Yes<br>2 No
d_8 *(required)*	MCP Cards<br>*If Yes, Number of cards available*	1 Yes<br>2 No
d_9 *(required)*	Indoor Play Equipment for ECD, specify	1 Adequate<br>2 Inadequate
d_10 *(required)*	Cooking Vessels	1 Adequate<br>2 Inadequate

Field	Question	Answer
d_10_s *(required)*	Serving utensils	1 Adequate<br>2 Inadequate
d_11 *(required)*	Water Storage Vessel<br>*If Yes, Number of Functional Equipment(s)*	1 Yes<br>2 No
d_12 *(required)*	Fuel for cooking, gas cylinder.<br>*If Yes, Number of Functional Equipment(s)*	1 Yes<br>2 No
ANGANWADI > anganwadi_iec_status		
e1_note	IEC Material/algorithms Availability Status of Programmes	
reserved_name_for_field_list_labels_144		1 Program being implemented<br>2 IEC material Available<br>3 IEC material displayed<br>4 Program not being implemented
e_1 *(required)*	Anemia - AMB charts and algorithms	1 Program being implemented<br>2 IEC material Available<br>3 IEC material displayed<br>4 Program not being implemented
e_2 *(required)*	Nutrition- POSHAN Abhiyan	1 Program being implemented<br>2 IEC material Available<br>3 IEC material displayed<br>4 Program not being implemented
e_3 *(required)*	Supplementary nutrition program	1 Program being implemented<br>2 IEC material Available<br>3 IEC material displayed<br>4 Program not being implemented
e_4 *(required)*	Sanitation and Hygiene: Swachh Bharat Mission Gramin, handwashing	1 Program being implemented<br>2 IEC material Available<br>3 IEC material displayed<br>4 Program not being implemented
e_5 *(required)*	Immunization	1 Program being implemented<br>2 IEC material Available<br>3 IEC material displayed<br>4 Program not being implemented
e_6 *(required)*	Breastfeeding	1 Program being implemented<br>2 IEC material Available<br>3 IEC material displayed<br>4 Program not being implemented
e_7 *(required)*	Diarrhea management- Zinc, ORS	1 Program being implemented<br>2 IEC material Available<br>3 IEC material displayed<br>4 Program not being implemented
e_8 *(required)*	ECD	1 Program being implemented<br>2 IEC material Available<br>3 IEC material displayed<br>4 Program not being implemented
e_9 *(required)*	PMMVY	1 Program being implemented<br>2 IEC material Available<br>3 IEC material displayed<br>4 Program not being implemented
e_other *(required)*	Please specify other	
ANGANWADI > anganwadi_register		

Field	Question	Answer
e_10 *(required)*	Preconception Health Register	1 Yes<br>2 No
e_11 *(required)*	Pregnant Women Health Register	1 Yes<br>2 No
e_12 *(required)*	Lactating Mothers Health Register	1 Yes<br>2 No
e_13 *(required)*	Children Growth and Development Register	1 Yes<br>2 No
e_14 *(required)*	Food Distribution & Stock Registers	1 Yes<br>2 No
e_15 *(required)*	Home Visit Register	1 Yes<br>2 No
e_16 *(required)*	Referral and Follow-up Register	1 Yes<br>2 No
e_17 *(required)*	Poshan Tracker Register	1 Yes<br>2 No
ANGANWADI > Maintenance of Record & data		
ANGANWADI > Maintenance of Record & data > anganwadi_records_phr<br>*Group relevant when: ${e_10} =1*		
generated_table_list_label_166	Preconception Health Register	
reserved_name_for_field_list_labels_167		1 Indicator is filled<br>2 Indicator Not filled<br>3 Indicator not available
f_2_1_1 *(required)*	Date of Visit	1 Indicator is filled<br>2 Indicator Not filled<br>3 Indicator not available
f_2_1_2 *(required)*	Unique Beneficiary ID	1 Indicator is filled<br>2 Indicator Not filled<br>3 Indicator not available
f_2_1_3 *(required)*	Women's Name	1 Indicator is filled<br>2 Indicator Not filled<br>3 Indicator not available
f_2_1_4 *(required)*	Husband's Name	1 Indicator is filled<br>2 Indicator Not filled<br>3 Indicator not available
f_2_1_5 *(required)*	Age	1 Indicator is filled<br>2 Indicator Not filled<br>3 Indicator not available
f_2_1_6 *(required)*	Address	1 Indicator is filled<br>2 Indicator Not filled<br>3 Indicator not available
f_2_1_7 *(required)*	Contact Number	1 Indicator is filled<br>2 Indicator Not filled<br>3 Indicator not available
f_2_1_8 *(required)*	Pregnancy History	1 Indicator is filled<br>2 Indicator Not filled<br>3 Indicator not available
f_2_1_9 *(required)*	Height	1 Indicator is filled<br>2 Indicator Not filled<br>3 Indicator not available
f_2_1_10 *(required)*	Weight	1 Indicator is filled<br>2 Indicator Not filled<br>3 Indicator not available
f_2_1_11 *(required)*	Tracking of weight gain	1 Indicator is filled<br>2 Indicator Not filled<br>3 Indicator not available
f_2_1_12 *(required)*	BMI Category	1 Indicator is filled<br>2 Indicator Not filled<br>3 Indicator not available

Field	Question	Answer
f_2_1_13 *(required)*	Anemia status	1 Indicator is filled<br>2 Indicator Not filled<br>3 Indicator not available
f_2_1_14 *(required)*	Counseling Provided for nutrition and WASH intervention	1 Indicator is filled<br>2 Indicator Not filled<br>3 Indicator not available
f_2_1_15 *(required)*	SNP provided	1 Indicator is filled<br>2 Indicator Not filled<br>3 Indicator not available
ANGANWADI > Maintenance of Record & data > anganwadi_records_pregreg<br>*Group relevant when: ${e_11} =1*		
generated_table_list_label_183	Pregnant Women Health Register	
reserved_name_for_field_list_labels_184		1 Indicator is filled<br>2 Indicator Not filled<br>3 Indicator not available
f_2_2_1 *(required)*	Date of Visit	1 Indicator is filled<br>2 Indicator Not filled<br>3 Indicator not available
f_2_2_2 *(required)*	Unique Beneficiary ID	1 Indicator is filled<br>2 Indicator Not filled<br>3 Indicator not available
f_2_2_3 *(required)*	Women's Name	1 Indicator is filled<br>2 Indicator Not filled<br>3 Indicator not available
f_2_2_4 *(required)*	Husband's Name	1 Indicator is filled<br>2 Indicator Not filled<br>3 Indicator not available
f_2_2_5 *(required)*	Age	1 Indicator is filled<br>2 Indicator Not filled<br>3 Indicator not available
f_2_2_6 *(required)*	Address	1 Indicator is filled<br>2 Indicator Not filled<br>3 Indicator not available
f_2_2_7 *(required)*	Contact Number	1 Indicator is filled<br>2 Indicator Not filled<br>3 Indicator not available
f_2_2_16 *(required)*	Family Number	1 Indicator is filled<br>2 Indicator Not filled<br>3 Indicator not available
f_2_2_17 *(required)*	Serial Number in Family	1 Indicator is filled<br>2 Indicator Not filled<br>3 Indicator not available
f_2_2_18 *(required)*	GPLA	1 Indicator is filled<br>2 Indicator Not filled<br>3 Indicator not available
f_2_2_8 *(required)*	Gestational Age	1 Indicator is filled<br>2 Indicator Not filled<br>3 Indicator not available
f_2_2_9 *(required)*	LMP	1 Indicator is filled<br>2 Indicator Not filled<br>3 Indicator not available
f_2_2_31 *(required)*	EDD	1 Indicator is filled<br>2 Indicator Not filled<br>3 Indicator not available
f_2_2_10 *(required)*	Height	1 Indicator is filled<br>2 Indicator Not filled<br>3 Indicator not available
f_2_2_10_1 *(required)*	Weight	1 Indicator is filled<br>2 Indicator Not filled

Field	Question	Answer
		3 Indicator not available
f_2_2_11 *(required)*	BMI Category	1 Indicator is filled<br>2 Indicator Not filled<br>3 Indicator not available
f_2_2_13 *(required)*	GWG Status	1 Indicator is filled<br>2 Indicator Not filled<br>3 Indicator not available
f_2_2_12 *(required)*	Anemia status	1 Indicator is filled<br>2 Indicator Not filled<br>3 Indicator not available
f_2_2_14 *(required)*	SNP Provided	1 Indicator is filled<br>2 Indicator Not filled<br>3 Indicator not available
f_2_2_15 *(required)*	Counseling Provided for nutrition	1 Indicator is filled<br>2 Indicator Not filled<br>3 Indicator not available
f_2_2_15_1 *(required)*	Counseling Provided for WASH intervention	1 Indicator is filled<br>2 Indicator Not filled<br>3 Indicator not available
f_2_2_22 *(required)*	IFA (Iron Folic Acid) Tablets (Number) – Add the number of tablets given each time	1 Indicator is filled<br>2 Indicator Not filled<br>3 Indicator not available
f_2_2_23 *(required)*	Pregnancy Check-ups (Date) – Four times	1 Indicator is filled<br>2 Indicator Not filled<br>3 Indicator not available
f_2_2_24 *(required)*	Place of Delivery	1 Indicator is filled<br>2 Indicator Not filled<br>3 Indicator not available
f_2_2_25 *(required)*	Child Birth Status (Live/Dead)	1 Indicator is filled<br>2 Indicator Not filled<br>3 Indicator not available
f_2_2_26 *(required)*	Child Gender	1 Indicator is filled<br>2 Indicator Not filled<br>3 Indicator not available
f_2_2_27 *(required)*	First Weight Date of Child	1 Indicator is filled<br>2 Indicator Not filled<br>3 Indicator not available
f_2_2_28 *(required)*	Serial Number of Child in Family Distribution	1 Indicator is filled<br>2 Indicator Not filled<br>3 Indicator not available
f_2_2_29 *(required)*	Number of Other Living Boys (Total No./Youngest Child)	1 Indicator is filled<br>2 Indicator Not filled<br>3 Indicator not available
f_2_2_30 *(required)*	Number of Other Living Girls (Total No./Youngest Child)	1 Indicator is filled<br>2 Indicator Not filled<br>3 Indicator not available
f_2_2_19 *(required)*	LMP (Last Menstrual Period)	1 Indicator is filled<br>2 Indicator Not filled<br>3 Indicator not available
f_2_2_20 *(required)*	EDD (Expected Date of Delivery)	1 Indicator is filled<br>2 Indicator Not filled<br>3 Indicator not available
f_2_2_21 *(required)*	Td Vaccination (Date)	1 Indicator is filled<br>2 Indicator Not filled<br>3 Indicator not available
ANGANWADI > Maintenance of Record & data > anganwadi_records_lacreg<br>*Group relevant when: ${e_12} =1*		
generated_table_list_label_218	Lactating Mothers Health Register	
reserved_name_for_field_list_labels_219		1 Indicator is filled

Field	Question	Answer
		2 Indicator Not filled<br>3 Indicator not available
f_2_3_1 *(required)*	Date of Visit	1 Indicator is filled<br>2 Indicator Not filled<br>3 Indicator not available
f_2_3_2 *(required)*	Unique Beneficiary ID	1 Indicator is filled<br>2 Indicator Not filled<br>3 Indicator not available
f_2_3_3 *(required)*	Husband's Name	1 Indicator is filled<br>2 Indicator Not filled<br>3 Indicator not available
f_2_3_4 *(required)*	Age	1 Indicator is filled<br>2 Indicator Not filled<br>3 Indicator not available
f_2_3_5 *(required)*	Address	1 Indicator is filled<br>2 Indicator Not filled<br>3 Indicator not available
f_2_3_6 *(required)*	Contact Number	1 Indicator is filled<br>2 Indicator Not filled<br>3 Indicator not available
f_2_3_7 *(required)*	Delivery Date	1 Indicator is filled<br>2 Indicator Not filled<br>3 Indicator not available
f_2_3_8 *(required)*	SNP Provided	1 Indicator is filled<br>2 Indicator Not filled<br>3 Indicator not available
f_2_3_9 *(required)*	Counseling Provided for nutrition and WASH intervention	1 Indicator is filled<br>2 Indicator Not filled<br>3 Indicator not available
f_2_3_10 *(required)*	Counselling provided for breastfeeding	1 Indicator is filled<br>2 Indicator Not filled<br>3 Indicator not available
ANGANWADI > Maintenance of Record & data > anganwadi_records_childreg<br>*Group relevant when: ${e_13} =1*		
generated_table_list_label_230	Children Growth and Development Register	
reserved_name_for_field_list_labels_231		1 Indicator is filled<br>2 Indicator Not filled<br>3 Indicator not available
f_2_4_1 *(required)*	Date of Visit	1 Indicator is filled<br>2 Indicator Not filled<br>3 Indicator not available
f_2_4_2 *(required)*	Unique Child ID	1 Indicator is filled<br>2 Indicator Not filled<br>3 Indicator not available
f_2_4_3 *(required)*	Child's Name	1 Indicator is filled<br>2 Indicator Not filled<br>3 Indicator not available
f_2_4_4 *(required)*	Mother's Name	1 Indicator is filled<br>2 Indicator Not filled<br>3 Indicator not available
f_2_4_5 *(required)*	Father's Name	1 Indicator is filled<br>2 Indicator Not filled<br>3 Indicator not available
f_2_4_6 *(required)*	Date of Birth	1 Indicator is filled<br>2 Indicator Not filled<br>3 Indicator not available
f_2_4_7 *(required)*	Age	1 Indicator is filled<br>2 Indicator Not filled<br>3 Indicator not available

Field	Question	Answer
f_2_4_8 *(required)*	Birth weight & Tracking of weight gain	1 Indicator is filled<br>2 Indicator Not filled<br>3 Indicator not available
f_2_4_9 *(required)*	Tracking of length	1 Indicator is filled<br>2 Indicator Not filled<br>3 Indicator not available
f_2_4_10 *(required)*	Tracking of MUAC	1 Indicator is filled<br>2 Indicator Not filled<br>3 Indicator not available
f_2_4_11 *(required)*	Weight Gain Status	1 Indicator is filled<br>2 Indicator Not filled<br>3 Indicator not available
f_2_4_12 *(required)*	SNP Provided	1 Indicator is filled<br>2 Indicator Not filled<br>3 Indicator not available
ANGANWADI > Maintenance of Record & data > anganwadi_records_foodreg<br>*Group relevant when: ${e_14} =1*		
generated_table_list_label_244	Food Distribution & Stock Registers	
reserved_name_for_field_list_labels_245		1 Indicator is filled<br>2 Indicator Not filled<br>3 Indicator not available
f_2_5_1 *(required)*	Date Received	1 Indicator is filled<br>2 Indicator Not filled<br>3 Indicator not available
f_2_5_2 *(required)*	Type of SNP for each beneficiary	1 Indicator is filled<br>2 Indicator Not filled<br>3 Indicator not available
f_2_5_3 *(required)*	Other food items	1 Indicator is filled<br>2 Indicator Not filled<br>3 Indicator not available
f_2_5_4 *(required)*	Quantity Received	1 Indicator is filled<br>2 Indicator Not filled<br>3 Indicator not available
f_2_5_5 *(required)*	Date of Distribution	1 Indicator is filled<br>2 Indicator Not filled<br>3 Indicator not available
f_2_5_6 *(required)*	Quantity Distributed (Beneficiary-wise)	1 Indicator is filled<br>2 Indicator Not filled<br>3 Indicator not available
f_2_5_7 *(required)*	Remaining Stock	1 Indicator is filled<br>2 Indicator Not filled<br>3 Indicator not available
ANGANWADI > Maintenance of Record & data > anganwadi_records_homereg<br>*Group relevant when: ${e_15} =1*		
generated_table_list_label_253	Home Visit Register	
reserved_name_for_field_list_labels_254		1 Indicator is filled<br>2 Indicator Not filled<br>3 Indicator not available
f_2_6_1 *(required)*	Date of Visit	1 Indicator is filled<br>2 Indicator Not filled<br>3 Indicator not available
f_2_6_2 *(required)*	Address of House Visited	1 Indicator is filled<br>2 Indicator Not filled<br>3 Indicator not available
f_2_6_3 *(required)*	Beneficiary Details (Name, Age, Sex, and Beneficiary Type)	1 Indicator is filled<br>2 Indicator Not filled<br>3 Indicator not available
f_2_6_4 *(required)*	Purpose of Visit (screening, counseling, food distribution, food intake supervision, etc.)	1 Indicator is filled<br>2 Indicator Not filled<br>3 Indicator not available

Field	Question	Answer
f_2_6_5 *(required)*	Follow-up Actions	1 Indicator is filled<br>2 Indicator Not filled<br>3 Indicator not available
ANGANWADI > Maintenance of Record & data > anganwadi_records_referral<br>*Group relevant when: ${e_16} =1*		
generated_table_list_label_260	Referral and Follow-up Register	
reserved_name_for_field_list_labels_261		1 Indicator is filled<br>2 Indicator Not filled<br>3 Indicator not available
f_2_7_1 *(required)*	Unique Referral ID	1 Indicator is filled<br>2 Indicator Not filled<br>3 Indicator not available
f_2_7_2 *(required)*	Beneficiary Name	1 Indicator is filled<br>2 Indicator Not filled<br>3 Indicator not available
f_2_7_3 *(required)*	Reason for Referral	1 Indicator is filled<br>2 Indicator Not filled<br>3 Indicator not available
f_2_7_4 *(required)*	Facility Referred	1 Indicator is filled<br>2 Indicator Not filled<br>3 Indicator not available
f_2_7_5 *(required)*	Date of Referral	1 Indicator is filled<br>2 Indicator Not filled<br>3 Indicator not available
f_2_7_6 *(required)*	Follow up action	1 Indicator is filled<br>2 Indicator Not filled<br>3 Indicator not available
ANGANWADI > Maintenance of Record & data > poshan_register<br>*Group relevant when: ${e_17} =1*		
generated_table_list_label_268	Poshan tracker Register	
reserved_name_for_field_list_labels_269		1 Indicator is filled<br>2 Indicator Not filled<br>3 Indicator not available
f_2_8_1 *(required)*	S.NO	1 Indicator is filled<br>2 Indicator Not filled<br>3 Indicator not available
f_2_8_2 *(required)*	Family Number	1 Indicator is filled<br>2 Indicator Not filled<br>3 Indicator not available
f_2_8_3 *(required)*	Women's Name	1 Indicator is filled<br>2 Indicator Not filled<br>3 Indicator not available
f_2_8_4 *(required)*	Date Received	1 Indicator is filled<br>2 Indicator Not filled<br>3 Indicator not available
f_2_8_5 *(required)*	Number of days of supplementary nutrition received in a month	1 Indicator is filled<br>2 Indicator Not filled<br>3 Indicator not available
f_2_8_6 *(required)*	Disability	1 Indicator is filled<br>2 Indicator Not filled<br>3 Indicator not available
ANGANWADI > anganwadi_ess_med		
h_1 *(required)*	Name of the drug/item - Essential Medicines<br>*Response constrained to: not(selected(${h_1} , '9999') and count-selected(${h_1}) > 1)*	1 IFA Tablets<br>2 IFA Syrup<br>3 IFA Drops<br>4 Calcium (Tablets)<br>5 Anthelmintic<br>6 Multi Micronutrient Tablets<br>7 Multi Micronutrient Syrup<br>8 Zinc Tablets

Field	Question	Answer
		9 Zinc Drops<br>10 ORS Sachets<br>99 Other Medicine (Specify)<br>9999 No medicine available
ANGANWADI > anganwadi_ess_med > [h_1_count1] (1)		(Repeated group)
ANGANWADI > anganwadi_ess_med > [h_1_count1] (1) > drugs_group<br>*Group relevant when: ${h_1} !=9999*		
h_1_t *(required)*	Target beneficiary	1 Preconception women (18-35 Years)<br>2 Pregnant women<br>3 Postnatal/ lactating women<br>4 0-6 Months Infants<br>5 6-24 Months Infants & Children
h_1_2 *(required)*	Strength	
h_1_3 *(required)*	Specification	
h_1_4 *(required)*	Projected monthly requirements based on beneficiary numbers	
h_1_5 *(required)*	Current numbers in stock	
h_1_6 *(required)*	Stock out	
h_1_7 *(required)*	Remarks	
h_1_8 *(required)*	Challenges	
ANGANWADI > anganwadi_beneficiary		
ANGANWADI > anganwadi_beneficiary > Facility's Beneficiaries (Patient) Load (In Last One Year)		
i_2 *(required)*	Preconception women (18-35 years)<br>*Response constrained to: (.>=0 and .<=150) or (.=999)*	
i_3 *(required)*	Pregnant women<br>*Response constrained to: (.>=0 and .<=150) or (.=999)*	
i_4 *(required)*	Lactating women<br>*Response constrained to: (.>=0 and .<=150) or (.=999)*	
i_5 *(required)*	6 to 12 months infants<br>*Response constrained to: (.>=0 and .<=150) or (.=999)*	
i_6 *(required)*	12 to 24 months children<br>*Response constrained to: (.>=0 and .<=150) or (.=999)*	
ANGANWADI > anganwadi_beneficiary > i_7<br>*Group relevant when: ${i_2} > 0*		
i_7_1	In last 1 year, how many Preconception women screened<br>*Fill 999 if no information available*	
i_7_2 *(required)*	Height measured(Number)<br>*Response constrained to: (.>=0 and .<=150) or (.=999)*	
i_7_3 *(required)*	Weight measured (Number)<br>*Response constrained to: (.>=0 and .<=150) or (.=999)*	
i_7_4 *(required)*	Identified with BMI<16 kg/m2(Number)<br>*Response constrained to: (.>=0 and .<=150) or (.=999)*	
i_7_5 *(required)*	Identified with BMI 16-18.49 kg/m2(Number)<br>*Response constrained to: (.>=0 and .<=150) or (.=999)*	
i_7_6 *(required)*	Identified with BMI 18.5- <21 kg/m2(Number)<br>*Response constrained to: (.>=0 and .<=150) or (.=999)*	
ANGANWADI > anganwadi_beneficiary > i_9<br>*Group relevant when: ${i_3} > 0*		
i_9_1	In last 1 year, how many Pregnant women screened<br>*Fill 999 if no information available*	
i_9_2 *(required)*	Height measured in First Trimester(Number)<br>*Response constrained to: (.>=0 and .<=150) or (.=999)*	
i_9_3 *(required)*	Weight measured in All Trimester(Number)<br>*Response constrained to: (.>=0 and .<=150) or (.=999)*	
i_9_4 *(required)*	Whom Gestational Weight Gain (GWG) was tracked at all ANC visits(Number)<br>*Response constrained to: (.>=0 and .<=150) or (.=999)*	
i_9_5 *(required)*	Identified with BMI < 18.5 kg/m2 in First Trimester(Number)<br>*Response constrained to: (.>=0 and .<=150) or (.=999)*	
i_9_6 *(required)*	Identified with BMI < 25 kg/m2 in First Trimester(Number)<br>*Response constrained to: (.>=0 and .<=150) or (.=999)*	
i_9_7 *(required)*	Pregnant women with inadequate gestational weight gain(Number)<br>*Response constrained to: (.>=0 and .<=150) or (.=999)*	

Field	Question	Answer
ANGANWADI > anganwadi_beneficiary > i_11<br>*Group relevant when: ${i_4} > 0 or ${i_5} > 0 or ${i_6} > 0*		
i_11_1	Postnatal/Lactation Women (0-6 Months) & 0-24 Months Infants & Child<br>*Fill 999 if no information available*	
i_11_2 *(required)*	0-6 months Infants exclusively breastfed (EBF)(Number)<br>*Response constrained to: (.>=0 and .<=150) or (.=999)*	
i_11_3 *(required)*	Infants 6-12 months with inadequate weight gain(Number)<br>*Response constrained to: (.>=0 and .<=150) or (.=999)*	
i_11_4 *(required)*	Children 12-24 months with inadequate weight gain(Number)<br>*Response constrained to: (.>=0 and .<=150) or (.=999)*	
i_11_5 *(required)*	Children (0-24 months) with adequate weight gain from birth till 2 years of age(Number)<br>*Response constrained to: (.>=0 and .<=150) or (.=999)*	
remarks_b	Remarks for facility Beneficiary load	
j_1 *(required)*	Do you provide SNP to any of the following group?	1 Preconception women (18-35 years)<br>2 Pregnant women<br>3 Lactating women<br>4 6 to 12 months infants<br>5 12 to 24 months children<br>6 3 to 6 year children
ANGANWADI > i_8<br>*Group relevant when: selected(${j_1} ,1)*		
i_8_1	Nutrition Support Provided to Preconception women (aged 18-35 years) Currently<br>*Fill 999 if no information available*	
i_8_2 *(required)*	BMI<16 kg/m2(Number)<br>*Response constrained to: (.>=0 and .<=150) or (.=999)*	
i_8_3 *(required)*	BMI 16-18.49 kg/m2(Number)<br>*Response constrained to: (.>=0 and .<=150) or (.=999)*	
i_8_4 *(required)*	BMI 18.5- <21 kg/m2(Number)<br>*Response constrained to: (.>=0 and .<=150) or (.=999)*	
ANGANWADI > i_10<br>*Group relevant when: selected(${j_1} ,2)*		
i_10_1	Nutrition Support Provided to Pregnant women Currently<br>*Fill 999 if no information available*	
i_10_2 *(required)*	Pregnant women(Number)<br>*Response constrained to: (.>=0 and .<=150) or (.=999)*	
i_10_3 *(required)*	BMI < 18.5 kg/m2(Number)<br>*Response constrained to: (.>=0 and .<=150) or (.=999)*	
i_10_4 *(required)*	Woman in 2nd trimster(Number)<br>*Response constrained to: (.>=0 and .<=150) or (.=999)*	
i_10_6 *(required)*	Woman in 3rd trimster(Number)<br>*Response constrained to: (.>=0 and .<=150) or (.=999)*	
i_10_5 *(required)*	Inadequate gestational weight gain (IGWG)(Number)<br>*Response constrained to: (.>=0 and .<=150) or (.=999)*	
ANGANWADI > i_12<br>*Group relevant when: selected(${j_1} ,4) or selected(${j_1} ,5) or selected(${j_1} ,6)*		
i_12_1	Nutrition Support Provided to Postnatal/Lactation Women (0-6 Months) & 0-24 Months Infants & Child Currently<br>*Fill 999 if no information available*	
i_12_2 *(required)*	Lactation women(Number)<br>*Response constrained to: (.>=0 and .<=150) or (.=999)*	
i_12_3 *(required)*	Infants 6-12 months with adequate weight gain(Number)<br>*Response constrained to: (.>=0 and .<=150) or (.=999)*	
i_12_4 *(required)*	Children 12-24 months adequate weight gain(Number)<br>*Response constrained to: (.>=0 and .<=150) or (.=999)*	
i_12_5 *(required)*	Infants 6-12 months with inadequate weight gain (Number)<br>*Response constrained to: (.>=0 and .<=150) or (.=999)*	
i_12_6 *(required)*	Children 12-24 months with inadequate weight gain (Number)<br>*Response constrained to: (.>=0 and .<=150) or (.=999)*	
ANGANWADI > [j_1_count1] (1)		(Repeated group)
ANGANWADI > [j_1_count1] (1) > Supplementary Nutrition		
ANGANWADI > [j_1_count1] (1) > Supplementary Nutrition > food_practices2_1_1_awc		
j_1_3_1_awc *(required)*	Type of Food Served<br>प्रदान किए गए भोजन का प्रकार	1 Hot Cooked Meal<br>2 THR

Field	Question	Answer
		3 WINGS(Egg)<br>4 WINGS(Milk)<br>5 Oil
ANGANWADI > [j_1_count1] (1) > Supplementary Nutrition > food_practices2_1_1_awc > WINGS Eggs<br>*Group relevant when: selected(${j_1_3_1_awc} ,3)*		
egg_1 *(required)*	Total Quantity Served in a month(in number)<br>*Response constrained to: .>= 0 and .<=31*	
egg_2 *(required)*	Served for how many days in a week<br>*Response constrained to: .>= 0 and .<=7*	
egg_3 *(required)*	Quantity Served per day(in number)<br>*Response constrained to: .>= 0 and .<=2*	
egg_4 *(required)*	Variation in Quantity/Type Based on Beneficiary Condition<br>लाभार्थी की स्थिति के आधार पर मात्रा/प्रकार में भिन्नता<br>*If yes, specify*	1 Yes<br>2 No
ANGANWADI > [j_1_count1] (1) > Supplementary Nutrition > food_practices2_1_1_awc > WINGS Milk<br>*Group relevant when: selected(${j_1_3_1_awc} ,4)*		
milk_1 *(required)*	Total Quantity Served in a month(in ml)	
milk_2 *(required)*	Served for how many days in a week	
milk_3 *(required)*	Quantity Served per day(in ml)<br>प्रति दिन कितना मिला (ग्राम में)	
milk_5 *(required)*	Variation in Quantity/Type Based on Beneficiary Condition<br>लाभार्थी की स्थिति के आधार पर मात्रा/प्रकार में भिन्नता<br>*If yes, specify*	1 Yes<br>2 No
ANGANWADI > [j_1_count1] (1) > Supplementary Nutrition > food_practices2_1_1_awc > OIL<br>*Group relevant when: selected(${j_1_3_1_awc} ,5)*		
oil_1 *(required)*	Total Quantity Served in a month(in ml)	
oil_2 *(required)*	Served for how many days in a week	
oil_3 *(required)*	Quantity Served per day(in ml)<br>प्रति दिन कितना मिला (ग्राम में)	
oil_5 *(required)*	Variation in Quantity/Type Based on Beneficiary Condition<br>लाभार्थी की स्थिति के आधार पर मात्रा/प्रकार में भिन्नता<br>*If yes, specify*	1 Yes<br>2 No
ANGANWADI > [j_1_count1] (1) > Supplementary Nutrition > food_practices2_1_1_awc > hcm_1_awc<br>*Group relevant when: selected(${j_1_3_1_awc} , '1')*		
hcm_1_note_awc	Hot Cooked Meal<br>गर्म पका हुआ भोजन	
hot_cooke_name *(required)*	Specify Hot cooked meal name	
j_1_14_1_awc *(required)*	Variation in Quantity/Type Based on Beneficiary Condition<br>लाभार्थी की स्थिति के आधार पर मात्रा/प्रकार में भिन्नता<br>*If yes, specify*	1 Yes<br>2 No
ANGANWADI > [j_1_count1] (1) > Supplementary Nutrition > food_practices2_1_1_awc > thr_1_awc<br>*Group relevant when: selected(${j_1_3_1_awc} , '2')*		
thr_text1_awc *(required)*	THR (Take home ration) THR	1 Channa<br>2 Dalia<br>3 Jaggary<br>4 Panjiri<br>5 Rajma<br>6 Rice<br>7 Salt<br>8 Sevia<br>9 Soya<br>10 Salt Biscuit<br>11 Sweet Biscuit<br>12 WMP(Milk)<br>13 Black Chana<br>14 Chane Ki Daal<br>15 WINGS(Snacks)<br>16 Wings2<br>17 Wings3<br>18 Wings4<br>19 Wings5<br>20 Wings6<br>21 Wings7

Field	Question	Answer
		22 Wings8<br>23 Wings9<br>24 Wings10<br>99 Other
ANGANWADI > [j_1_count1] (1) > Supplementary Nutrition > food_practices2_1_1_awc > thr_1_awc > THR [thr_cal_repeat] (1)		(Repeated group)
hot_total_awc *(required)*	Total Quantity Served in a month(in gram)<br>*Response constrained to: .>= 0*	
week_awc	Served for how many days in a week<br>*Response constrained to: .>= 0 and .<=7*	
quantity_p3	Quantity Served per day(in gram)<br>प्रति दिन कितना मिला (ग्राम में)<br>*Response constrained to: .>= 0 and .<=500*	
quantity_p4 *(required)*	Quantity Served per day(in ML)<br>*Question relevant when: selected(${thr_text1_awc} ,'13')*<br>*Response constrained to: .>= 0 and .<=500*	
j_1_18_1_awc *(required)*	Variation in Quantity/Type Based on Beneficiary Condition<br>लाभार्थी की स्थिति के आधार पर मात्रा/प्रकार में भिन्नता<br>*If yes, specify*	1 Yes<br>2 No
remark_obs	Observational Remarks	
ANGANWADI > photo_group		
photo1	Photo Upload register 1	
photo2	Photo Upload register 2	
photo3	Photo Upload register 3	
photo4	Photo Upload register 4	
photo5	Photo Upload register 5	
photo6	Photo Upload register 6	
photo7	Photo Upload register 7	
photo8	Photo Upload register 8	
document	Please Upload PDF document	

HWC Assessment form

Field	Question	Answer
worker *(required)*	Worker Name	151 Abuhamza<br>152 Anmol Saini<br>153 Anshika Sahota<br>154 Ekta<br>155 Jyoti Devi<br>156 Kritika Thakur<br>157 Mehak Thakur<br>158 Poonam Devi<br>159 Riya Puri<br>160 Shivanshi<br>161 Varsha Kumari<br>162 Anchal Walia<br>163 Harshali<br>164 Kritika Puri
blocks *(required)*	Block Names	block_1 Amb<br>block_2 Basdehra<br>block_3 Gagret<br>block_4 Haroli<br>block_5 Thanakalan
hwc_selected *(required)*	Health wellness centre (HWC)	hwc_1 HWC-HSC KatoharKalan<br>hwc_2 HWC-HSC KatoharKhurd<br>hwc_3 HWC-HSC Kuthiari<br>hwc_4 HWC-HSC Neharian<br>hwc_5 HWC-HSC Panjoa<br>hwc_6 HWC-HSC Jagannath Mandir<br>hwc_7 HWC-HSC Naloh<br>hwc_8 HWC-HSC PolianProhitan<br>hwc_9 HWC-HSC Ripooh Misran<br>hwc_10 HWC-HSC Behra<br>hwc_11 HWC-HSC Diara<br>hwc_12 HWC-HSC Hamboli<br>hwc_13 HWC-HSC Takarala<br>hwc_14 HWC-HSC Thathal<br>hwc_15 HWC-HSC Badwana<br>hwc_16 HWC-HSC BaherBatehar<br>hwc_17 HWC-HSC Dilwari<br>hwc_18 HWC-HSC Ghangret<br>hwc_19 HWC-HSC GindpurMaloun<br>hwc_20 HWC-HSC Kharoh<br>hwc_21 HWC-HSC Thanikpura<br>hwc_22 HWC-HSC Chahbag<br>hwc_23 HWC-HSC Chowar<br>hwc_24 HWC-HSC Daloh<br>hwc_25 HWC-HSC Gangoti (sapouri)<br>hwc_26 HWC-HSC Lohara<br>hwc_27 HWC-HSC Andora<br>hwc_28 HWC-HSC Saloi<br>hwc_29 HWC-HSC Sidhchalher<br>hwc_30 HWC-HSC Suin<br>hwc_31 HWC-HSC Basal<br>hwc_32 HWC-HSC KotlaKhurd

Field	Question	Answer		
			hwc_33	HWC-HSC Rainsary
			hwc_34	HWC-HSC Takka
			hwc_35	HWC-HSC Basoli
			hwc_36	HWC-HSC Dangoli
			hwc_37	HWC-HSC Kotla Kalan
			hwc_38	HWC-HSC Lamlehri
			hwc_39	HWC-HSC SamoorKalan
			hwc_40	HWC-HSC Badoli
			hwc_41	HWC-HSC Badsala
			hwc_42	HWC-HSC Ghandwal
			hwc_43	HWC-HSC Kuriala
			hwc_44	HWC-HSC NangalSalangri
			hwc_45	HWC-HSC Panoh
			hwc_46	HWC-HSC Teuri
			hwc_47	HWC-HSC Bedehar
			hwc_48	HWC-HSC Behdala
			hwc_49	HWC-HSC BhadolianKalan
			hwc_50	HWC-HSC Charatgarh
			hwc_51	HWC-HSC Chattara
			hwc_52	HWC-HSC Chattarpur
			hwc_53	HWC-HSC Fatehwal
			hwc_54	HWC-HSC Jalgran
			hwc_55	HWC-HSC Jankaur
			hwc_56	HWC-HSC Jakhera
			hwc_57	HWC-HSC Jhudowal
			hwc_58	HWC-HSC Malahat
			hwc_59	HWC-HSC Nangran
			hwc_60	HWC-HSC Rakkar
			hwc_61	HWC-HSC Rampur
			hwc_62	HWC-HSC Sanoli
			hwc_63	HWC-HSC Sassan
			hwc_64	HWC-HSC Gondpur Banera
			hwc_65	HWC-HSC Kuneran
			hwc_66	HWC-HSC Nakroh
			hwc_67	HWC-HSC Ambota
			hwc_68	HWC-HSC Guglehar
			hwc_69	HWC-HSC Keori
			hwc_70	HWC-HSC KutheraJaswalan
			hwc_71	HWC-HSC Loharli
			hwc_72	HWC-HSC MawaSindhian
			hwc_73	HWC-HSC Oel
			hwc_74	HWC-HSC Pambra
			hwc_75	HWC-HSC Saghnai
			hwc_76	HWC-HSC Amboa
			hwc_77	HWC-HSC Babehar
			hwc_78	HWC-HSC Bhaderkali
			hwc_79	HWC-HSC Chalet
			hwc_80	HWC-HSC Dangoh
			hwc_81	HWC-HSC Deoli
			hwc_82	HWC-HSC Ghanari
			hwc_83	HWC-HSC Mandwara
			hwc_84	HWC-HSC MawaKohlan
			hwc_85	HWC-HSC Nangal Jariyala
			hwc_86	HWC-HSC Pirthipur

Field	Question	Answer		
			hwc_87	HWC-HSC Salohberri
			hwc_88	HWC-HSC Baliwal
			hwc_89	HWC-HSC Dharampur
			hwc_90	HWC-HSC Sainsowal
			hwc_91	HWC-HSC Bathri
			hwc_92	HWC-HSC Bathu
			hwc_93	HWC-HSC Beetan
			hwc_94	HWC-HSC Nangal Kalan
			hwc_95	HWC-HSC Singan
			hwc_96	HWC-HSC Ispur
			hwc_97	HWC-HSC Chhetran
			hwc_98	HWC-HSC Gondpur Bulla
			hwc_99	HWC-HSC Janani
			hwc_100	HWC-HSC Kuthar Beet
			hwc_101	HWC-HSC Polian Beet
			hwc_102	HWC-HSC Pubowal
			hwc_103	HWC-HSC Bhadauri
			hwc_104	HWC-HSC Lalehri
			hwc_105	HWC-HSC Nangal Khurd
			hwc_106	HWC-HSC Palakwah
			hwc_107	HWC-HSC Pandoga
			hwc_108	HWC-HSC Nangnoli
			hwc_109	HWC-HSC Panjawar
			hwc_110	HWC-HSC Kangar
			hwc_111	HWC-HSC Chamiari
			hwc_112	HWC-HSC Jarola
			hwc_113	HWC-HSC Piploo
			hwc_114	HWC-HSC Bharmout
			hwc_115	HWC-HSC Charoli
			hwc_116	HWC-HSC Charara
			hwc_117	HWC-HSC Deehar
			hwc_118	HWC-HSC Dhanet
			hwc_119	HWC-HSC Dhundla
			hwc_120	HWC-HSC Jassana
			hwc_121	HWC-HSC Kodra
			hwc_122	HWC-HSC Tanoh
			hwc_123	HWC-HSC Balh
			hwc_124	HWC-HSC Bihroo
			hwc_125	HWC-HSC Boul
			hwc_126	HWC-HSC Budhwar
			hwc_127	HWC-HSC Chugath
			hwc_128	HWC-HSC Chulari
			hwc_129	HWC-HSC Harot
			hwc_130	HWC-HSC Karian
			hwc_131	HWC-HSC Paroin
			hwc_132	HWC-HSC Saili
			hwc_133	HWC-HSC Talai
			hwc_134	HWC-HSC Ambehra
			hwc_135	HWC-HSC Baduhi
			hwc_136	HWC-HSC Bhindla
			hwc_137	HWC-HSC Jol
HWC				
HWC > HWC Identification [hwc_label]				
hwc_a_1 *(required)*	Date of Assessment: *Response constrained to: .= today()*			
hwc_a_6 *(required)*	Facility id: *Response constrained to: regex(., '^[0-9]{10}$')*			
hwc_a_8 *(required)*	Facility Address with landmark			

Field	Question	Answer
hwc_a_10 *(required)*	Total Population covered by the facility	
hwc_a_11 *(required)*	Facility In-charge Name:<br>*Response constrained to: not(regex(.,'^(.*)\d(.*)$'))*	
hwc_a_12 *(required)*	Facility In-charge Designation:	
hwc_a_13 *(required)*	Facility In-charge Contact No.:<br>*Response constrained to: regex(.,"^[6|7|8|9]\d{9}$")*	
hwc_a_14	Facility In-charge Email Id.:	
hwc_a_15	Any Additional Respondent?	1 Yes<br>2 No
HWC > hwc_add_informant<br>*Group relevant when: ${hwc_a_15} =1*		
hwc_a_15_1	Additional Respondent	
hwc_a_15_2 *(required)*	Additional Respondent Name<br>*Response constrained to: not(regex(.,'^(.*)\d(.*)$'))*	
hwc_a_15_3 *(required)*	Additional Respondent Designation	
HWC > hwc_infra		
hwc_b_1 *(required)*	Located within Main Habitation (*where the majority of the population resides)<br>*If No, Distance in Km*	1 Yes<br>2 No
hwc_b_2 *(required)*	Connected to Motorable Road	1 Yes<br>2 No
hwc_b_3 *(required)*	Designated Government HWC-SHC Building	1 Yes<br>2 No
hwc_b_4 *(required)*	General Structure of Building	1 Newly Built<br>2 Newly Renovated<br>3 Old Structure<br>4 Falling apart
hwc_b_5 *(required)*	Maintenance of Building	1 No maintenance<br>2 Once a year<br>3 Once in 3 years<br>99 Other (specify)
hwc_b_6 *(required)*	Is electrical supply available?	1 24/7<br>3 Average duration of electricity supply per day<br>2 No
hwc_b_6_or	Is power backup available?	1 Generator<br>2 Inverter<br>3 Solar<br>99 Other (Specify)<br>9999 None
hwc_b_7 *(required)*	Main source of water supply?	1 Borewell<br>2 Piped water<br>99 Other with duration
hwc_b_8 *(required)*	Toilet Facility	1 Yes<br>2 No
hwc_b_9 *(required)*	Littering	1 Yes<br>2 No
HWC > Toilet & Other facilities		
hwc_b_8_1 *(required)*	Facility available inside the toilet<br>*Question relevant when: ${hwc_b_8} =1*<br>*Response constrained to: not(selected(${hwc_b_8_1} , '9999') and count-selected(${hwc_b_8_1}) > 1)*	1 Cleanliness<br>2 Toilet tap with running water<br>3 Flush working properly<br>4 Water bucket and mug inside the toilet<br>5 Washbasin with running water<br>6 Soap/liquid items handwash/hand rub<br>7 Disable friendly<br>8 Dustbin with yellow line (in female washroom)<br>9999 None

Field	Question	Answer
hwc_b_8_2 *(required)*	Gender specific toilet facility<br>*Question relevant when: ${hwc_b_8} =1*	1 Both (male & female separate) Available<br>2 Only Male Available<br>3 Only Female Available<br>4 Common Toilet Available<br>5 Availability of a disabled-friendly toilet
hwc_b_10 *(required)*	Availability of general waste bins	1 Green<br>2 Blue<br>3 Both<br>99 Other
hwc_b_11 *(required)*	Management (collection, segregation, storage, transportation, treatment and disposal) of BMW as per Bio-Medical Waste Management Rules, 2018	1 Yes<br>2 No
hwc_b_11_bins *(required)*	Availability of colour coded BMW bins	1 Red<br>2 Yellow<br>3 Blue Puncture proof<br>4 White Puncture proof
HWC > hwc_infra2		
hwc_b_12	Quality Assurance certification/services	
reserved_name_for_field_list_labels_60		1 Yes<br>2 No
hwc_b_13 *(required)*	Kayakalp	1 Yes<br>2 No
hwc_b_14 *(required)*	NQAS	1 Yes<br>2 No
hwc_b_16 *(required)*	SUMAN	1 Yes<br>2 No
HWC > Emergency area and Triage services		
hwc_b_17 *(required)*	Emergency services available	1 Yes<br>2 No
hwc_b_17_1 *(required)*	Triage services available	1 Yes<br>2 No
hwc_b_18 *(required)*	OPD Room	1 Yes<br>2 No
hwc_b_28 *(required)*	Common Room for all type of services	1 Yes<br>2 No
hwc_b_20 *(required)*	Laboratory	1 Yes<br>2 No
hwc_b_19 *(required)*	OPD Room - Available facilities<br>*Question relevant when: ${hwc_b_18} =1*<br>*Response constrained to: not(selected(${hwc_b_19} , '9999') and count-selected(${hwc_b_19}) > 1)*	1 Adequate table, chairs, bench and stool<br>2 Examination table<br>5 Footstep<br>3 Washbasin with Running Water Supply<br>4 Waiting area with sitting facility<br>9999 None of the above
hwc_b_28_c *(required)*	Common Room Available facilites<br>*Question relevant when: ${hwc_b_28} =1*<br>*Response constrained to: not(selected(${hwc_b_28_c} , '9999') and count-selected(${hwc_b_28_c}) > 1)*	1 Adequate table, chairs, bench and stool<br>2 Examination table<br>5 Footstep<br>3 Washbasin with Running Water Supply<br>4 Waiting area with sitting facility<br>9999 None of the above
hwc_b_21 *(required)*	Laboratory - Available facilities<br>*Question relevant when: ${hwc_b_20} =1*<br>*Response constrained to: not(selected(${hwc_b_21} , '9999') and count-selected(${hwc_b_21}) > 1)*	1 Adequate table, chairs, bench and stool<br>2 Washbasin with Running Water Supply

Field	Question	Answer
		3 Adequate area for sample collection area<br>4 Adequate areas for carrying out diagnostic activities<br>5 Adequate area for keeping equipment and storage of drugs and records<br>9999 None of the above
hwc_b_26 *(required)*	Availability of bed<br>*If Yes, total number of beds available*	1 Yes<br>2 No
HWC > Availability Status of Human Resource at HWC-SHC		
hwc_c_2 *(required)*	Sanctioned (No.) CHO	
hwc_c_3 *(required)*	In position (No.) CHO<br>*Question relevant when: ${hwc_c_2} > 0*<br>*Response constrained to: . >= 0 and . <= ${hwc_c_2}*	
HWC > Availability Status of Human Resource at HWC-SHC > CHO (1)<br>*Group relevant when: ${hwc_c_2} > 0 and ${hwc_c_3} > 0*		(Repeated group)
hwc_c_4	Status	1 Available<br>2 Long Leave<br>3 Deputed Somewhere Else
HWC > Availability Status of Human Resource at HWC-SHC > Training Status of CHO (1)		(Repeated group)
HWC > Availability Status of Human Resource at HWC-SHC > Training Status of CHO (1) > hwc_cho_training		
generated_table_list_label_83	Training Status of CHO	
reserved_name_for_field_list_labels_84		1 Yes<br>2 No
hwc_c_1_1 *(required)*	BMW Training	1 Yes<br>2 No
hwc_c_1_2 *(required)*	NCD Training	1 Yes<br>2 No
hwc_c_1_3 *(required)*	Routine Immunisation Training	1 Yes<br>2 No
hwc_c_1_4 *(required)*	NDD Training	1 Yes<br>2 No
hwc_c_1_5 *(required)*	JAS Training	1 Yes<br>2 No
hwc_c_1_6 *(required)*	Quality management	1 Yes<br>2 No
hwc_c_1_7 *(required)*	DVDMS Training	1 Yes<br>2 No
hwc_c_1_8 *(required)*	NTEP Training	1 Yes<br>2 No
hwc_c_1_9 *(required)*	U-Win App Training	1 Yes<br>2 No
hwc_c_1_10 *(required)*	Other	1 Yes<br>2 No
hwc_c_1_11	Please specify Training (others)<br>*Fill NA if no data*<br>*Question relevant when: ${hwc_c_1_10} =1*	
hwc_c_6 *(required)*	Sanctioned (No.) Health Worker (Female)/ANM	
hwc_c_7 *(required)*	In position (No.) Health Worker (Female)/ANM<br>*Question relevant when: ${hwc_c_6} > 0*<br>*Response constrained to: . >=0 and . <= ${hwc_c_6}*	
HWC > Availability Status of Human Resource at HWC-SHC > Health Worker (Female)/ANM (1)<br>*Group relevant when: ${hwc_c_6} > 0 and ${hwc_c_7} > 0*		(Repeated group)
hwc_c_8 *(required)*	Status	1 Available<br>2 Long Leave<br>3 Deputed Somewhere Else
HWC > Availability Status of Human Resource at HWC-SHC > Training Status of Health Worker (1)		(Repeated group)
HWC > Availability Status of Human Resource at HWC-SHC > Training Status of Health Worker (1) > hwc_hwf_training		
generated_table_list_label_103	Training Status of Health Worker (Female)/ANM	

Field	Question	Answer
reserved_name_for_field_list_labels_104		1 Yes<br>2 No
hwc_c_5_1 *(required)*	Routine immunization	1 Yes<br>2 No
hwc_c_5_2 *(required)*	SAANS Training (Under 5 Pneumonia management)	1 Yes<br>2 No
hwc_c_5_3 *(required)*	HBNC Training	1 Yes<br>2 No
hwc_c_5_4 *(required)*	Diarrhoea management Training (IDCF)	1 Yes<br>2 No
hwc_c_5_5 *(required)*	MAA program	1 Yes<br>2 No
hwc_c_5_6 *(required)*	IUCD	1 Yes<br>2 No
hwc_c_5_7 *(required)*	NCD	1 Yes<br>2 No
hwc_c_5_8 *(required)*	VHND	1 Yes<br>2 No
hwc_c_5_9 *(required)*	VHSNC	1 Yes<br>2 No
hwc_c_5_10 *(required)*	Eat right tool	1 Yes<br>2 No
hwc_c_5_11 *(required)*	Other	1 Yes<br>2 No
hwc_c_5_12	Please specify Training (others)<br>*Fill NA if no data*<br>*Question relevant when: ${hwc_c_5_11} =1*	
hwc_c_10 *(required)*	Sanctioned (No.) Male Healh Worker	
hwc_c_11 *(required)*	In position (No.) Male Health Worker<br>*Question relevant when: ${hwc_c_10} > 0*<br>*Response constrained to: . >=0 and . <= ${hwc_c_10}*	
HWC > Availability Status of Human Resource at HWC-SHC > Male Health Worker (1)<br>*Group relevant when: ${hwc_c_10} > 0 and ${hwc_c_11} > 0*		(Repeated group)
hwc_c_12 *(required)*	Status	1 Available<br>2 Long Leave<br>3 Deputed Somewhere Else
HWC > Availability Status of Human Resource at HWC-SHC > Training Status of Male Health Worker (1)		(Repeated group)
HWC > Availability Status of Human Resource at HWC-SHC > Training Status of Male Health Worker (1) > hwc_hwm_training		
generated_table_list_label_124	Training Status of Male Health Worker	
reserved_name_for_field_list_labels_125		1 Yes<br>2 No
hwc_c_9_1 *(required)*	TB	1 Yes<br>2 No
hwc_c_9_2 *(required)*	Malaria	1 Yes<br>2 No
hwc_c_9_3 *(required)*	Routine Immunization	1 Yes<br>2 No
hwc_c_9_4 *(required)*	Water testing	1 Yes<br>2 No
hwc_c_9_5 *(required)*	Other	1 Yes<br>2 No
hwc_c_9_6	Please specify Training (others)<br>*Fill NA if no data*<br>*Question relevant when: ${hwc_c_9_5} =1*	
hwc_c_14 *(required)*	Sanctioned (No.) ASHA	
hwc_c_15 *(required)*	In position (No.) ASHA<br>*Question relevant when: ${hwc_c_14} > 0*<br>*Response constrained to: . >=0 and . <= ${hwc_c_14}*	
HWC > Availability Status of Human Resource at HWC-SHC > ASHA (1)<br>*Group relevant when: ${hwc_c_14} > 0 and ${hwc_c_15} > 0*		(Repeated group)

Field	Question	Answer
hwc_c_16 *(required)*	Status	1 Available<br>2 Long Leave<br>3 Deputed Somewhere Else
HWC > Availability Status of Human Resource at HWC-SHC > Training Status of ASHA (1)		(Repeated group)
HWC > Availability Status of Human Resource at HWC-SHC > Training Status of ASHA (1) > hwc_asha_training		
generated_table_list_label_139	Training Status of ASHA	
reserved_name_for_field_list_labels_140		1 Yes<br>2 No
hwc_c_13_1 *(required)*	VHND	1 Yes<br>2 No
hwc_c_13_2 *(required)*	IPC (Interpersonal communication)	1 Yes<br>2 No
hwc_c_13_3 *(required)*	Basic RI	1 Yes<br>2 No
hwc_c_13_4 *(required)*	HBNC	1 Yes<br>2 No
hwc_c_13_5 *(required)*	HBYC	1 Yes<br>2 No
hwc_c_13_6 *(required)*	Sanitation and hygiene	1 Yes<br>2 No
hwc_c_13_7	Communicable disease	1 Yes<br>2 No
hwc_c_13_8	Non communicable disease	1 Yes<br>2 No
hwc_c_13_9	Module 6 and 7	1 Yes<br>2 No
hwc_c_13_10	Other (specify)	1 Yes<br>2 No
hwc_c_13_11 *(required)*	Please specify Training (others)<br>*Fill NA if no data*<br>*Question relevant when: ${hwc_c_13_10} =1*	
hwc_c_17 *(required)*	Any other staff?	1 Yes<br>2 No
HWC > Availability Status of Human Resource at HWC-SHC > any_other<br>*Group relevant when: ${hwc_c_17} =1*		
hwc_c_17_o1	Name<br>*Response constrained to: not(regex(.,'^(.*)\d(.*)$'))*	
hwc_c_17_o2	Designation<br>*Response constrained to: not(regex(.,'^(.*)\d(.*)$'))*	
HWC > Availability Status of Equipment at HWC-SHC		
hwc_d_11 *(required)*	ANC Services	1 Yes<br>2 No
HWC > Availability Status of Equipment at HWC-SHC > hwc_anc_service<br>*Group relevant when: ${hwc_d_11} =1*		
hwc_d_12 *(required)*	Blood Pressure Monitor (Digital)<br>*If yes, please specify number of functional equipments*	1 Yes<br>2 No
hwc_d_12_1 *(required)*	Blood Pressure Monitor (Manual)<br>*If yes, please specify number of functional equipments*	1 Yes<br>2 No
hwc_d_13 *(required)*	Stethoscope<br>*If yes, please specify number of functional equipments*	1 Yes<br>2 No
hwc_d_14 *(required)*	Clinical Thermometer<br>*If yes, please specify number of functional equipments*	1 Yes<br>2 No
hwc_d_14_d *(required)*	Digital Thermometer<br>*If yes, please specify number of functional equipments*	1 Yes<br>2 No
hwc_d_15 *(required)*	Adult Weighing Scale (Digital)<br>*If yes, please specify number of functional equipments*	1 Yes<br>2 No
hwc_d_15_1 *(required)*	Adult Weighing Scale (Analog)<br>*If yes, please specify number of functional equipments*	1 Yes<br>2 No
hwc_d_16 *(required)*	Stadiometer<br>*If yes, please specify number of functional equipments*	1 Yes<br>2 No

Field	Question	Answer
hwc_d_17 *(required)*	Measuring Tape<br>*If yes, please specify number of functional equipments*	1 Yes<br>2 No
hwc_d_18 *(required)*	Examination Lamp with White Light<br>*If yes, please specify number of functional equipments*	1 Yes<br>2 No
hwc_d_19 *(required)*	Fetoscope Monitor<br>*If yes, please specify number of functional equipments*	1 Yes<br>2 No
hwc_d_19_f *(required)*	Doppler Fetal Monitor<br>*If yes, please specify number of functional equipments*	1 Yes<br>2 No
hwc_d_20 *(required)*	Cusco's Speculum<br>*If yes, please specify number of functional equipments*	1 Yes<br>2 No
hwc_d_20_s *(required)*	Sims's Speculum<br>*If yes, please specify number of functional equipments*	1 Yes<br>2 No
hwc_d_21 *(required)*	Pulse oximeter (adult)<br>*If yes, please specify number of functional equipments*	1 Yes<br>2 No
hwc_d_22 *(required)*	Pediatric Services	1 Yes<br>2 No
HWC > Availability Status of Equipment at HWC-SHC > Pediatric Services<br>*Group relevant when: ${hwc_d_22} =1*		
hwc_d_23 *(required)*	Pediatric Stethoscope<br>*If yes, please specify number of functional equipments*	1 Yes<br>2 No
hwc_d_24 *(required)*	Digital Thermometer<br>*If yes, please specify number of functional equipments*	1 Yes<br>2 No
hwc_d_24_i *(required)*	Infrared Thermometer<br>*If yes, please specify number of functional equipments*	1 Yes<br>2 No
hwc_d_25 *(required)*	Infantometer<br>*If yes, please specify number of functional equipments*	1 Yes<br>2 No
hwc_d_26 *(required)*	Baby Weighing Scale (Electronic)<br>*If yes, please specify number of functional equipments*	1 Yes<br>2 No
hwc_d_26_1 *(required)*	Baby Weighing Scale (Manual)<br>*If yes, please specify number of functional equipments*	1 Yes<br>2 No
hwc_d_27 *(required)*	Pulse oximeter (Pediatric)<br>*If yes, please specify number of functional equipments*	1 Yes<br>2 No
hwc_d_29 *(required)*	Pediatric Resuscitation Kit<br>*If no, please specify missing equipments*	1 Yes<br>2 No
hwc_d_30 *(required)*	Breast pump (Manual)<br>*If yes, please specify number of functional equipments*	1 Yes<br>2 No
hwc_d_30_1 *(required)*	Breast pump (Electric)<br>*If yes, please specify number of functional equipments*	1 Yes<br>2 No
hwc_d_60 *(required)*	Immunization Services	1 Yes<br>2 No
HWC > Availability Status of Equipment at HWC-SHC > Immunization Services<br>*Group relevant when: ${hwc_d_60} =1*		
hwc_d_65 *(required)*	Vaccine Carriers with Ice Packs<br>*If yes, please specify number of functional equipments*	1 Yes<br>2 No
hwc_d_66 *(required)*	Cold Box<br>*If yes, please specify number of functional equipments*	1 Yes<br>2 No
lab_facility	Is lab service available ?	1 Yes<br>2 No
lab_services	Type of facility<br>*Question relevant when: ${lab_facility} =1*	1 Goverment<br>2 KRSNAA/Empanelled
HWC > Availability Status of Diagnostic tests/services at HWC-SHC<br>*Group relevant when: selected(${lab_services} ,1) or selected(${lab_services} ,2)*		
hwc_e_1 *(required)*	Hemoglobin - Required Equipment & Reagents are available in the Laboratory?<br>*If Yes, what method was used?*	1 Tested in facility<br>3 Only sample collection<br>2 Not Available
hwc_e_2 *(required)*	Platelet Count - Required Equipment & Reagents are available in the Laboratory?<br>*If Yes, what method was used?*	1 Tested in facility<br>3 Only sample collection<br>2 Not Available
hwc_e_4 *(required)*	Blood Group And Rh Typing - Required Equipment & Reagents are available in the Laboratory?	1 Tested in facility

Field	Question	Answer
	If Yes, what method was used?	3 Only sample collection<br>2 Not Available
hwc_e_5 *(required)*	Blood Cross Matching - Required Equipment & Reagents are available in the Laboratory?<br>*If Yes, what method was used?*	1 Tested in facility<br>3 Only sample collection<br>2 Not Available
hwc_e_7 *(required)*	MP slide method - Required Equipment & Reagents are available in the Laboratory?<br>*If Yes, what method was used?*	1 Tested in facility<br>3 Only sample collection<br>2 Not Available
hwc_e_8 *(required)*	Malaria rapid test - Required Equipment & Reagents are available in the Laboratory?<br>*If Yes, what method was used?*	1 Tested in facility<br>3 Only sample collection<br>2 Not Available
hwc_e_23 *(required)*	Urine test for pregnancy(UPT)<br>*If Yes, what method was used?*	1 Tested in facility<br>3 Only sample collection<br>2 Not Available
hwc_e_11 *(required)*	Urine for microalbumin - Required Equipment & Reagents are available in the Laboratory?<br>*If Yes, what method was used?*	1 Tested in facility<br>3 Only sample collection<br>2 Not Available
hwc_e_14 *(required)*	RPR/VDRL test for syphilis - Required Equipment & Reagents are available in the Laboratory?<br>*If Yes, what method was used?*	1 Tested in facility<br>3 Only sample collection<br>2 Not Available
hwc_e_15 *(required)*	HIV test (Antibodies 1/2 and HIV 1/2) - Required Equipment & Reagents are available in the Laboratory?<br>*If Yes, what method was used?*	1 Tested in facility<br>3 Only sample collection<br>2 Not Available
hwc_e_16 *(required)*	Hepatitis B surface antigen test - Required Equipment & Reagents are available in the Laboratory?<br>*If Yes, what method was used?*	1 Tested in facility<br>3 Only sample collection<br>2 Not Available
hwc_e_18 *(required)*	HCV Antibody Test (Anti HCV) - Required Equipment & Reagents are available in the Laboratory?<br>*If Yes, what method was used?*	1 Tested in facility<br>3 Only sample collection<br>2 Not Available
hwc_e_19 *(required)*	Sputum, pus etc. for AFB - Required Equipment & Reagents are available in the Laboratory?<br>*If Yes, what method was used?*	1 Tested in facility<br>3 Only sample collection<br>2 Not Available
hwc_e_20 *(required)*	Typhoid test (IgM) - Required Equipment & Reagents are available in the Laboratory?<br>*If Yes, what method was used?*	1 Tested in facility<br>3 Only sample collection<br>2 Not Available
hwc_e_21 *(required)*	Blood sugar -RBS (Random blood sugar) - Required Equipment & Reagents are available in the Laboratory?<br>*If Yes, what method was used?*	1 Tested in facility<br>3 Only sample collection<br>2 Not Available
hwc_e_22 *(required)*	Oral Glucose Tolerance Test (OGTT) - Required Equipment & Reagents are available in the Laboratory?<br>*If Yes, what method was used?*	1 Tested in facility<br>3 Only sample collection<br>2 Not Available
hwc_e_27 *(required)*	Urine Dipstick Test<br>*If Yes, what method was used?*	1 Tested in facility<br>3 Only sample collection<br>2 Not Available
hwc_e_22_remarks *(required)*	Remarks (if any)	
HWC > hwc_iec		
generated_table_list_label_263	Availability Status of IEC Material/algorithms and management charts related to the national/state programs in local script.	
reserved_name_for_field_list_labels_264		1 Program being implemented<br>2 IEC material Available<br>3 IEC material displayed<br>4 Program not being implemented
hwc_f_1	Family Planning- Mission Parivar Vikas	1 Program being implemented<br>2 IEC material Available<br>3 IEC material displayed<br>4 Program not being implemented
hwc_f_2	Anemia- AMB charts and algorithms	1 Program being implemented<br>2 IEC material Available

Field	Question	Answer
		3 IEC material displayed<br>4 Program not being implemented
hwc_f_3	Adolescent health programs	1 Program being implemented<br>2 IEC material Available<br>3 IEC material displayed<br>4 Program not being implemented
hwc_f_4	Nutrition- POSHAN Abhiyan	1 Program being implemented<br>2 IEC material Available<br>3 IEC material displayed<br>4 Program not being implemented
hwc_f_5	Sanitation and Hygiene: Swachh Bharat Mission Gramin, handwashing	1 Program being implemented<br>2 IEC material Available<br>3 IEC material displayed<br>4 Program not being implemented
hwc_f_6	ANC	1 Program being implemented<br>2 IEC material Available<br>3 IEC material displayed<br>4 Program not being implemented
hwc_f_7	PMSMA	1 Program being implemented<br>2 IEC material Available<br>3 IEC material displayed<br>4 Program not being implemented
hwc_f_8	Management of PPH, Shock	1 Program being implemented<br>2 IEC material Available<br>3 IEC material displayed<br>4 Program not being implemented
hwc_f_9	Newborn Resuscitation, NSSK	1 Program being implemented<br>2 IEC material Available<br>3 IEC material displayed<br>4 Program not being implemented
hwc_f_10	HBNC	1 Program being implemented<br>2 IEC material Available<br>3 IEC material displayed<br>4 Program not being implemented
hwc_f_11	Childhood care- MAA, HBYC	1 Program being implemented<br>2 IEC material Available<br>3 IEC material displayed<br>4 Program not being implemented
hwc_f_12	Immunization	1 Program being implemented<br>2 IEC material Available<br>3 IEC material displayed<br>4 Program not being implemented
hwc_f_13	Breastfeeding	1 Program being implemented<br>2 IEC material Available<br>3 IEC material displayed<br>4 Program not being implemented
hwc_f_14	Diarrhea management- Zinc, ORS	1 Program being implemented<br>2 IEC material Available<br>3 IEC material displayed

Field	Question	Answer
		4 Program not being implemented
hwc_f_15	Pneumonia Management	1 Program being implemented<br>2 IEC material Available<br>3 IEC material displayed<br>4 Program not being implemented
hwc_f_16	JSY	1 Program being implemented<br>2 IEC material Available<br>3 IEC material displayed<br>4 Program not being implemented
hwc_f_17	JSSK	1 Program being implemented<br>2 IEC material Available<br>3 IEC material displayed<br>4 Program not being implemented
hwc_f_18	Ambulance (National Ambulance Services)	1 Program being implemented<br>2 IEC material Available<br>3 IEC material displayed<br>4 Program not being implemented
hwc_f_19	Telemedicine	1 Program being implemented<br>2 IEC material Available<br>3 IEC material displayed<br>4 Program not being implemented
hwc_f_20	Danger Signs (Maternal and Neonatal care)	1 Program being implemented<br>2 IEC material Available<br>3 IEC material displayed<br>4 Program not being implemented
HWC > hwc_register_group		
hwc_f_10_r *(required)*	General OPD Register	1 Yes<br>2 No
hwc_f_11_r *(required)*	Pre-conception Women Register	1 Yes<br>2 No
hwc_f_12_r *(required)*	ANC Register	1 Yes<br>2 No
hwc_f_13_r *(required)*	Pediatric/0-24 Months Infants & Children Register	1 Yes<br>2 No
hwc_f_14_r *(required)*	Labour Room Register	1 Yes<br>2 No
hwc_f_15_r *(required)*	Laboratory Record	1 Yes<br>2 No
hwc_f_16_r *(required)*	Referral Register	1 Yes<br>2 No
HWC > hwc_g_group		
HWC > hwc_g_group > hwc_maintenance_record_data<br>*Group relevant when: ${hwc_f_10_r} =1*		
generated_table_list_label_295	General OPD Register	
reserved_name_for_field_list_labels_296		1 Indicator is filled<br>2 Indicator Not filled<br>3 Indicator not available
hwc_g_1_1 *(required)*	Date	1 Indicator is filled<br>2 Indicator Not filled<br>3 Indicator not available
hwc_g_1_2 *(required)*	Patient ID	1 Indicator is filled<br>2 Indicator Not filled<br>3 Indicator not available

Field	Question	Answer
hwc_g_1_3 *(required)*	Name	1 Indicator is filled<br>2 Indicator Not filled<br>3 Indicator not available
hwc_g_1_4 *(required)*	Age	1 Indicator is filled<br>2 Indicator Not filled<br>3 Indicator not available
hwc_g_1_5 *(required)*	Sex	1 Indicator is filled<br>2 Indicator Not filled<br>3 Indicator not available
hwc_g_1_6 *(required)*	Address & Contact details	1 Indicator is filled<br>2 Indicator Not filled<br>3 Indicator not available
hwc_g_1_7 *(required)*	Marital status	1 Indicator is filled<br>2 Indicator Not filled<br>3 Indicator not available
hwc_g_1_8 *(required)*	Condition for which visiting OPD	1 Indicator is filled<br>2 Indicator Not filled<br>3 Indicator not available
hwc_g_1_9 *(required)*	Physical Examination	1 Indicator is filled<br>2 Indicator Not filled<br>3 Indicator not available
hwc_g_1_10 *(required)*	Treatment provided	1 Indicator is filled<br>2 Indicator Not filled<br>3 Indicator not available
hwc_g_1_11 *(required)*	Investigations done	1 Indicator is filled<br>2 Indicator Not filled<br>3 Indicator not available
hwc_g_1_12 *(required)*	Follow-up visit date	1 Indicator is filled<br>2 Indicator Not filled<br>3 Indicator not available
hwc_g_1_13 *(required)*	Referrals made.	1 Indicator is filled<br>2 Indicator Not filled<br>3 Indicator not available
HWC > hwc_g_group > hwc_preconception_record_data<br>*Group relevant when: ${hwc_f_11_r} =1*		
generated_table_list_label_310	Pre-conception Women Register	
reserved_name_for_field_list_labels_311		1 Indicator is filled<br>2 Indicator Not filled<br>3 Indicator not available
hwc_g_2_1 *(required)*	Date of visit	1 Indicator is filled<br>2 Indicator Not filled<br>3 Indicator not available
hwc_g_2_2 *(required)*	Woman ID	1 Indicator is filled<br>2 Indicator Not filled<br>3 Indicator not available
hwc_g_2_3 *(required)*	Name	1 Indicator is filled<br>2 Indicator Not filled<br>3 Indicator not available
hwc_g_2_4 *(required)*	Age	1 Indicator is filled<br>2 Indicator Not filled<br>3 Indicator not available
hwc_g_2_5 *(required)*	Address & Contact details	1 Indicator is filled<br>2 Indicator Not filled<br>3 Indicator not available
hwc_g_2_6 *(required)*	Height	1 Indicator is filled<br>2 Indicator Not filled<br>3 Indicator not available
hwc_g_2_7 *(required)*	Weight	1 Indicator is filled<br>2 Indicator Not filled

Field	Question	Answer
		3 Indicator not available
hwc_g_2_8 *(required)*	BMI	1 Indicator is filled<br>2 Indicator Not filled<br>3 Indicator not available
hwc_g_2_9 *(required)*	Medical history (e.g.Anemia,DM,HT,RTI,Thyroid) and screening results of the same conditions	1 Indicator is filled<br>2 Indicator Not filled<br>3 Indicator not available
hwc_g_2_10 *(required)*	Place/facility where the conditions tracked during follow up visits	1 Indicator is filled<br>2 Indicator Not filled<br>3 Indicator not available
hwc_g_2_12 *(required)*	GPLA Status	1 Indicator is filled<br>2 Indicator Not filled<br>3 Indicator not available
hwc_g_2_13 *(required)*	IFA Supplementaion	1 Indicator is filled<br>2 Indicator Not filled<br>3 Indicator not available
hwc_g_2_14 *(required)*	Calcium Supplementation	1 Indicator is filled<br>2 Indicator Not filled<br>3 Indicator not available
hwc_g_2_15 *(required)*	Albendazole	1 Indicator is filled<br>2 Indicator Not filled<br>3 Indicator not available
HWC > hwc_g_group > hwc_anc_record_data<br>*Group relevant when: ${hwc_f_12_r} =1*		
generated_table_list_label_326	ANC Register	
reserved_name_for_field_list_labels_327		1 Indicator is filled<br>2 Indicator Not filled<br>3 Indicator not available
hwc_g_3_1 *(required)*	Date of visit	1 Indicator is filled<br>2 Indicator Not filled<br>3 Indicator not available
hwc_g_3_2 *(required)*	Woman ID	1 Indicator is filled<br>2 Indicator Not filled<br>3 Indicator not available
hwc_g_3_3 *(required)*	Name	1 Indicator is filled<br>2 Indicator Not filled<br>3 Indicator not available
hwc_g_3_4 *(required)*	Age	1 Indicator is filled<br>2 Indicator Not filled<br>3 Indicator not available
hwc_g_3_5 *(required)*	Address & Contact details	1 Indicator is filled<br>2 Indicator Not filled<br>3 Indicator not available
hwc_g_3_6 *(required)*	Gestational age (GA) at first registration	1 Indicator is filled<br>2 Indicator Not filled<br>3 Indicator not available
hwc_g_3_17 *(required)*	Height	1 Indicator is filled<br>2 Indicator Not filled<br>3 Indicator not available
hwc_g_3_18 *(required)*	Weight	1 Indicator is filled<br>2 Indicator Not filled<br>3 Indicator not available
hwc_g_3_7 *(required)*	BMI in first trimester	1 Indicator is filled<br>2 Indicator Not filled<br>3 Indicator not available
hwc_g_3_8 *(required)*	Records/columns of all ANC visits (no. of columns) along with provision to track GWG & Hb	1 Indicator is filled<br>2 Indicator Not filled<br>3 Indicator not available
hwc_g_3_9 *(required)*	Blood Pressure	1 Indicator is filled

Field	Question	Answer
		2 Indicator Not filled
		3 Indicator not available
hwc_g_3_10 *(required)*	Blood investigation-CBC	1 Indicator is filled
		2 Indicator Not filled
		3 Indicator not available
hwc_g_3_16 *(required)*	Blood investigation-TSH at the time of registeration	1 Indicator is filled
		2 Indicator Not filled
		3 Indicator not available
hwc_g_3_11 *(required)*	OGTT- 1 time	1 Indicator is filled
		2 Indicator Not filled
		3 Indicator not available
hwc_g_3_12 *(required)*	Hb-4 times	1 Indicator is filled
		2 Indicator Not filled
		3 Indicator not available
hwc_g_3_13 *(required)*	Urine routine & microscopy- 4 times	1 Indicator is filled
		2 Indicator Not filled
		3 Indicator not available
hwc_g_3_14 *(required)*	Ultrasound	1 Indicator is filled
		2 Indicator Not filled
		3 Indicator not available
hwc_g_3_15 *(required)*	Td vaccination in place	1 Indicator is filled
		2 Indicator Not filled
		3 Indicator not available
HWC > hwc_g_group > hwc_anc_record_ped<br>*Group relevant when: ${hwc_f_13_r} =1*		
generated_table_list_label_346	Pediatric/0-24 Months Infants & Children Register	
reserved_name_for_field_list_labels_347		1 Indicator is filled
		2 Indicator Not filled
		3 Indicator not available
hwc_g_4_1 *(required)*	Date of visit	1 Indicator is filled
		2 Indicator Not filled
		3 Indicator not available
hwc_g_4_2 *(required)*	Child ID	1 Indicator is filled
		2 Indicator Not filled
		3 Indicator not available
hwc_g_4_3 *(required)*	Name	1 Indicator is filled
		2 Indicator Not filled
		3 Indicator not available
hwc_g_4_4 *(required)*	Age	1 Indicator is filled
		2 Indicator Not filled
		3 Indicator not available
hwc_g_4_5 *(required)*	Sex	1 Indicator is filled
		2 Indicator Not filled
		3 Indicator not available
hwc_g_4_6 *(required)*	Mother Name	1 Indicator is filled
		2 Indicator Not filled
		3 Indicator not available
hwc_g_4_7 *(required)*	Father Name	1 Indicator is filled
		2 Indicator Not filled
		3 Indicator not available
hwc_g_4_8 *(required)*	Address & Contact details	1 Indicator is filled
		2 Indicator Not filled
		3 Indicator not available
hwc_g_4_9 *(required)*	Weight	1 Indicator is filled
		2 Indicator Not filled
		3 Indicator not available
hwc_g_4_10 *(required)*	Length	1 Indicator is filled
		2 Indicator Not filled
		3 Indicator not available

Field	Question	Answer
hwc_g_4_11 *(required)*	Tracking of IWG	1 Indicator is filled<br>2 Indicator Not filled<br>3 Indicator not available
hwc_g_4_12 *(required)*	Identification of danger signs	1 Indicator is filled<br>2 Indicator Not filled<br>3 Indicator not available
hwc_g_4_13 *(required)*	Morbidities Reported	1 Indicator is filled<br>2 Indicator Not filled<br>3 Indicator not available
hwc_g_4_14 *(required)*	Treatment Provided	1 Indicator is filled<br>2 Indicator Not filled<br>3 Indicator not available
hwc_g_4_15 *(required)*	immunization status	1 Indicator is filled<br>2 Indicator Not filled<br>3 Indicator not available
hwc_g_4_16 *(required)*	Follow-up visit Date	1 Indicator is filled<br>2 Indicator Not filled<br>3 Indicator not available
hwc_g_4_17 *(required)*	Referrals	1 Indicator is filled<br>2 Indicator Not filled<br>3 Indicator not available
HWC > hwc_g_group > hwc_labour_room_record_data<br>*Group relevant when: ${hwc_f_14_r} =1*		
generated_table_list_label_365	Labour Room Register	
reserved_name_for_field_list_labels_366		1 Indicator is filled<br>2 Indicator Not filled<br>3 Indicator not available
hwc_g_5_1 *(required)*	Date of admission	1 Indicator is filled<br>2 Indicator Not filled<br>3 Indicator not available
hwc_g_5_2 *(required)*	Patient ID	1 Indicator is filled<br>2 Indicator Not filled<br>3 Indicator not available
hwc_g_5_3 *(required)*	Name	1 Indicator is filled<br>2 Indicator Not filled<br>3 Indicator not available
hwc_g_5_4 *(required)*	Age	1 Indicator is filled<br>2 Indicator Not filled<br>3 Indicator not available
hwc_g_5_5 *(required)*	Husband's Name	1 Indicator is filled<br>2 Indicator Not filled<br>3 Indicator not available
hwc_g_5_6 *(required)*	Address & Contact details	1 Indicator is filled<br>2 Indicator Not filled<br>3 Indicator not available
hwc_g_5_7 *(required)*	Time of admission	1 Indicator is filled<br>2 Indicator Not filled<br>3 Indicator not available
hwc_g_5_8 *(required)*	Status at the time of admission	1 Indicator is filled<br>2 Indicator Not filled<br>3 Indicator not available
hwc_g_5_9 *(required)*	Type of delivery (e.g. normal,cesarean)	1 Indicator is filled<br>2 Indicator Not filled<br>3 Indicator not available
hwc_g_5_10 *(required)*	Complications before delivery	1 Indicator is filled<br>2 Indicator Not filled<br>3 Indicator not available
hwc_g_5_11 *(required)*	Complications during delivery	1 Indicator is filled<br>2 Indicator Not filled

Field	Question	Answer
		3 Indicator not available
hwc_g_5_12 *(required)*	Birth weight	1 Indicator is filled<br>2 Indicator Not filled<br>3 Indicator not available
hwc_g_5_13 *(required)*	APGAR score	1 Indicator is filled<br>2 Indicator Not filled<br>3 Indicator not available
hwc_g_5_14 *(required)*	Time of discharge	1 Indicator is filled<br>2 Indicator Not filled<br>3 Indicator not available
hwc_g_5_15 *(required)*	Referral information.	1 Indicator is filled<br>2 Indicator Not filled<br>3 Indicator not available
HWC > hwc_g_group > hwc_lab_record<br>*Group relevant when: ${hwc_f_15_r} =1*		
generated_table_list_label_382	Laboratory Record	
reserved_name_for_field_list_labels_383		1 Indicator is filled<br>2 Indicator Not filled<br>3 Indicator not available
hwc_g_6_1 *(required)*	Date	1 Indicator is filled<br>2 Indicator Not filled<br>3 Indicator not available
hwc_g_6_2 *(required)*	Sample ID	1 Indicator is filled<br>2 Indicator Not filled<br>3 Indicator not available
hwc_g_6_3 *(required)*	Patient ID	1 Indicator is filled<br>2 Indicator Not filled<br>3 Indicator not available
hwc_g_6_4 *(required)*	Name	1 Indicator is filled<br>2 Indicator Not filled<br>3 Indicator not available
hwc_g_6_5 *(required)*	Type of investigation	1 Indicator is filled<br>2 Indicator Not filled<br>3 Indicator not available
hwc_g_6_6 *(required)*	Investigation results	1 Indicator is filled<br>2 Indicator Not filled<br>3 Indicator not available
hwc_g_6_7 *(required)*	Remarks	1 Indicator is filled<br>2 Indicator Not filled<br>3 Indicator not available
hwc_g_6_8 *(required)*	Date of report	1 Indicator is filled<br>2 Indicator Not filled<br>3 Indicator not available
HWC > hwc_g_group > hwc_referral_record<br>*Group relevant when: ${hwc_f_16_r} =1*		
generated_table_list_label_392	Referral Register	
reserved_name_for_field_list_labels_393		1 Indicator is filled<br>2 Indicator Not filled<br>3 Indicator not available
hwc_g_8_1 *(required)*	Date of referral	1 Indicator is filled<br>2 Indicator Not filled<br>3 Indicator not available
hwc_g_8_2 *(required)*	Patient ID	1 Indicator is filled<br>2 Indicator Not filled<br>3 Indicator not available
hwc_g_8_3 *(required)*	Name	1 Indicator is filled<br>2 Indicator Not filled<br>3 Indicator not available
hwc_g_8_4 *(required)*	Address	1 Indicator is filled<br>2 Indicator Not filled

Field	Question	Answer
		3 Indicator not available
hwc_g_8_5 *(required)*	Reason for referral	1 Indicator is filled<br>2 Indicator Not filled<br>3 Indicator not available
hwc_g_8_6 *(required)*	Referring facility	1 Indicator is filled<br>2 Indicator Not filled<br>3 Indicator not available
hwc_g_8_7 *(required)*	Receiving facility	1 Indicator is filled<br>2 Indicator Not filled<br>3 Indicator not available
hwc_g_8_8 *(required)*	Date of receiving feedback	1 Indicator is filled<br>2 Indicator Not filled<br>3 Indicator not available
hwc_g_8_9 *(required)*	Follow-up visit date	1 Indicator is filled<br>2 Indicator Not filled<br>3 Indicator not available
HWC > hwc_i_group		
hwc_i_1_1 *(required)*	Iron, Vitamin, and Nutritional Supplements<br>*Response constrained to: not(selected(${hwc_i_1_1} , '9999') and count-selected(${hwc_i_1_1}) > 1)*	1 Tab. Folic Acid (5 mg/Tab)<br>2 Tab. Iron Folic Acid (IFA) (60 mg elemental Iron + 500 mcg Folic Acid, sugar coated)<br>3 Syp. Iron Folic Acid (IFA) (20 mg of elemental iron and 100 mcg of folic acid)<br>4 Inj. Iron Sucrose (50 mg/2.5 ml)<br>5 Tab. Calcium & Vitamin D (Calcium (500 mg) and Vit-D (250 IU))<br>6 Vitamin D Oral Solution (400 IU (10 mcg))<br>7 Multi-micronutrients (With higher dose of Vitamin B12)<br>99 Other Specify<br>9999 None
HWC > hwc_i_group > [hwc_i_1_cal1] (1)		(Repeated group)
HWC > hwc_i_group > [hwc_i_1_cal1] (1) > Availability Status of Essential Medicines Required at HWC-SHC for MCH Care [hwc_i_1_cal1]]<br>*Group relevant when: ${hwc_i_1_1} !=9999*		
hwc_i_1_t *(required)*	Target beneficiary	1 Preconception women (18-35 Years)<br>2 Pregnant women<br>3 Postnatal/ lactating women<br>4 0-6 Months Infants<br>5 6-24 Months Infants & Children
hwc_i_1_2 *(required)*	Projected Monthly Requirements	
hwc_i_1_3 *(required)*	Current Numbers in Stock	
hwc_i_1_4 *(required)*	Stock Out in the last 3 month<br>*If Yes type Remarks/Challenges*	1 Yes<br>2 No
hwc_i_2_1 *(required)*	Antibiotics<br>*Response constrained to: not(selected(${hwc_i_2_1} , '9999') and count-selected(${hwc_i_2_1}) > 1)*	1 Inj. Ampicillin Sodium (500 mg or 1 gm/Vial)<br>2 Cap. Amoxicillin (500 mg/Cap)<br>3 Tab. Augmentin (500 mg/Tab)<br>4 Tab. Cefixime (500 mg/Tab)<br>5 Inj. Gentamicin Sulphate (40 mg/ml)

Field	Question	Answer
		6 Inj. Cefotaxime + Sulbactam (Cefotaxime 1 gm + Sulbactam 0.5 gm/Vial)<br>7 Tab. Cefadroxil (500 mg/Tab)<br>8 Tab. Metronidazole (Coated) (400 mg/Tab)<br>9 Tab. Nitrofurantoin (100 mg/Tab)<br>10 Azithromycin oral liquid (200mg)<br>11 Amoxicillin powder for suspension (125mg)<br>12 Tab.Azithromycin (500mg)<br>13 Tab.Azithromycin (250mg)<br>14 Tab.Amoxicillin (250mg)<br>99 Other Specify<br>9999 None
HWC > hwc_i_group > [hwc_i_2_cal1] (1)		(Repeated group)
HWC > hwc_i_group > [hwc_i_2_cal1] (1) > Availability Status of Essential Medicines Required at HWC-SHC for MCH Care [hwc_i_2_cal1]}<br>*Group relevant when: ${hwc_i_2_1} !=9999*		
hwc_i_2_t *(required)*	Target beneficiary	1 Preconception women (18-35 Years)<br>2 Pregnant women<br>3 Postnatal/ lactating women<br>4 0-6 Months Infants<br>5 6-24 Months Infants & Children
hwc_i_2_2 *(required)*	Projected Monthly Requirements	
hwc_i_2_3 *(required)*	Current Numbers in Stock	
hwc_i_2_4 *(required)*	Stock Out in the last 3 month<br>*If Yes type Remarks/Challenges*	1 Yes<br>2 No
hwc_i_3_1 *(required)*	Antihypertensives and Seizure Management<br>*Response constrained to: not(selected(${hwc_i_3_1} , '9999') and count-selected(${hwc_i_3_1}) > 1)*	1 Tab. Methyldopa (250 mg/Tab)<br>2 Cap. Nifedipine (Soft gelatin) (10 mg/Cap)<br>3 Tab. Nifedipine SR (10 mg/Tab)<br>4 Tab. Labetalol (100 mg/Tab)<br>5 Inj. Labetalol (20 mg/4 ml Amp)<br>6 Inj. Magnesium Sulphate (500 mg/ml)<br>7 Tab. Phenytoin Sodium (100 mg/Tab)<br>8 Inj. Lorazepam (1 mg/ml)<br>9 Inj. Diazepam (5 mg/ml)<br>10 Amlodipine (5mg)<br>11 Telmisartan (40mg)<br>99 Other Specify<br>9999 None
HWC > hwc_i_group > [hwc_i_3_cal1] (1)		(Repeated group)
HWC > hwc_i_group > [hwc_i_3_cal1] (1) > Availability Status of Essential Medicines Required at HWC-SHC for MCH Care [hwc_i_3_cal1]}<br>*Group relevant when: ${hwc_i_3_1} !=9999*		
hwc_i_3_t *(required)*	Target beneficiary	1 Preconception women (18-35 Years)<br>2 Pregnant women<br>3 Postnatal/ lactating women<br>4 0-6 Months Infants<br>5 6-24 Months Infants & Children
hwc_i_3_2 *(required)*	Projected Monthly Requirements	

Field	Question	Answer
hwc_i_3_3 *(required)*	Current Numbers in Stock	
hwc_i_3_4 *(required)*	Stock Out in the last 3 month<br>*If Yes type Remarks/Challenges*	1 Yes<br>2 No
hwc_i_4_1 *(required)*	Analgesics/Antipyretic and Antiemetic<br>*Response constrained to: not(selected(${hwc_i_4_1} , '9999') and count-selected(${hwc_i_4_1}) > 1)*	1 Tab. Paracetamol (500 mg/Tab)<br>2 Tab. Ibuprofen (400 mg/Tab)<br>3 Tab. Domperidone (10 mg/Tab)<br>4 Syrup. Paracetamol (250ml)<br>5 Syrup. Paracetamol (150ml)<br>6 Syrup.Ibuprofen oral liquid (100mg)<br>7 Tab. Diclofenac 50mg<br>99 Other Specify<br>9999 None
HWC > hwc_i_group > [hwc_i_4_cal1] (1)		(Repeated group)
HWC > hwc_i_group > [hwc_i_4_cal1] (1) > Availability Status of Essential Medicines Required at HWC-SHC for MCH Care [hwc_i_4_cal1]]<br>*Group relevant when: ${hwc_i_4_1} !=9999*		
hwc_i_4_t *(required)*	Target beneficiary	1 Preconception women (18-35 Years)<br>2 Pregnant women<br>3 Postnatal/ lactating women<br>4 0-6 Months Infants<br>5 6-24 Months Infants & Children
hwc_i_4_2 *(required)*	Projected Monthly Requirements	
hwc_i_4_3 *(required)*	Current Numbers in Stock	
hwc_i_4_4 *(required)*	Stock Out in the last 3 month<br>*If Yes type Remarks/Challenges*	1 Yes<br>2 No
hwc_i_5_1 *(required)*	Anthelmintics<br>*Response constrained to: not(selected(${hwc_i_5_1} , '9999') and count-selected(${hwc_i_5_1}) > 1)*	1 Tab. Albendazole (Chewable) 400 mg/Tab<br>2 Albendazole Oral Solution 200 mg/5 ml<br>3 Oral Antimalarial<br>99 Other Specify<br>9999 None
HWC > hwc_i_group > [hwc_i_5_cal1] (1)		(Repeated group)
HWC > hwc_i_group > [hwc_i_5_cal1] (1) > Availability Status of Essential Medicines Required at HWC-SHC for MCH Care [hwc_i_5_cal1]]<br>*Group relevant when: ${hwc_i_5_1} !=9999*		
hwc_i_5_t *(required)*	Target beneficiary	1 Preconception women (18-35 Years)<br>2 Pregnant women<br>3 Postnatal/ lactating women<br>4 0-6 Months Infants<br>5 6-24 Months Infants & Children
hwc_i_5_2 *(required)*	Projected Monthly Requirements	
hwc_i_5_3 *(required)*	Current Numbers in Stock	
hwc_i_5_4 *(required)*	Stock Out in the last 3 month<br>*If Yes type Remarks/Challenges*	1 Yes<br>2 No
hwc_i_6_1 *(required)*	Diabetic & Thyroid Medications<br>*Response constrained to: not(selected(${hwc_i_6_1} , '9999') and count-selected(${hwc_i_6_1}) > 1)*	1 Inj. Human Soluble Insulin (40 IU/ml)<br>2 Tab. Metformin HCl (Coated) (500 mg/Tab)<br>3 Tab. Eltroxin / Thyroxine (100 mcg)<br>99 Other Specify<br>9999 None
HWC > hwc_i_group > [hwc_i_6_cal1] (1)		(Repeated group)
HWC > hwc_i_group > [hwc_i_6_cal1] (1) > Availability Status of Essential Medicines Required at HWC-SHC for MCH Care [hwc_i_6_cal1]]		

Field	Question	Answer
Group relevant when: ${hwc_i_6_1} !=9999		
hwc_i_6_t *(required)*	Target beneficiary	1 Preconception women (18-35 Years)<br>2 Pregnant women<br>3 Postnatal/ lactating women<br>4 0-6 Months Infants<br>5 6-24 Months Infants & Children
hwc_i_6_2 *(required)*	Projected Monthly Requirements	
hwc_i_6_3 *(required)*	Current Numbers in Stock	
hwc_i_6_4 *(required)*	Stock Out in the last 3 month<br>*If Yes type Remarks/Challenges*	1 Yes<br>2 No
hwc_i_7_1 *(required)*	Other Miscellaneous<br>*Response constrained to: not(selected(${hwc_i_7_1} , '9999') and count-selected(${hwc_i_7_1}) > 1)*	1 Inj. Td (0.5 ml/Amp)<br>2 Tab. Digoxin (0.25 mg/Tab)<br>3 Tab. Frusemide (40 mg/Tab)<br>4 Inj. Frusemide (10 mg/1 ml)<br>5 Inj. Betamethasone Sod. Phosphate (4 mg/ml)<br>6 Inj. Human Anti-D Immunoglobulin (300 mcg/1.5 ml)<br>7 IV Fluids<br>8 ORS Sachet<br>9 Zinc Tablet<br>10 Povidone Iodine Ointment (5% w/v)<br>11 Sanitary Napkins<br>12 Vaccines (As per National Programme)<br>99 Other Medicine (Specify)<br>9999 None
HWC > hwc_i_group > [hwc_i_7_cal1] (1)		(Repeated group)
HWC > hwc_i_group > [hwc_i_7_cal1] (1) > Availability Status of Essential Medicines Required at HWC-SHC for MCH Care [hwc_i_7_cal1]}<br>*Group relevant when: ${hwc_i_7_1} !=9999*		
hwc_i_7_t *(required)*	Target beneficiary	1 Preconception women (18-35 Years)<br>2 Pregnant women<br>3 Postnatal/ lactating women<br>4 0-6 Months Infants<br>5 6-24 Months Infants & Children
hwc_i_7_2 *(required)*	Projected Monthly Requirements	
hwc_i_7_3 *(required)*	Current Numbers in Stock	
hwc_i_7_4 *(required)*	Stock Out in the last 3 month<br>*If Yes type Remarks/Challenges*	1 Yes<br>2 No
hwc_7_1_other	Other Medicine (Specify)<br>*Question relevant when: ${hwc_i_7_1} =99*	
HWC > hwc_j_group		
HWC > hwc_j_group > Facility's Beneficiaries (Patient) Load		
hwc_j_note	Please mention the Numbers (Last 12 Month Data) against each<br>*Please fill 999 if no record available*	
hwc_j_1 *(required)*	Eligible couples	
hwc_j_2 *(required)*	Preconception women (18-35 years)	
hwc_j_3 *(required)*	Pregnant women	
hwc_j_4 *(required)*	Postnatal women	
hwc_j_5 *(required)*	0 to 6 months infants	
hwc_j_6 *(required)*	6 to 12 months infants	
hwc_j_7 *(required)*	12 to 24 months children	
hwc_j_8 *(required)*	Preconception women (18-35 years) screened for medical conditions	
hwc_j_9 *(required)*	Preconception women (18-35 years) screened for depressive symptoms-	
HWC > hwc_j_group > Preconception women (18-35 years) screened for medical conditions and depressive symptoms-		

Field	Question	Answer
Group relevant when: ${hwc_j_2} > 1		
hwc_j_8_note	Please mention the Number of Preconception women (18-35 years) screened (Last 12 Month Data) against each<br>*Please fill 999 if no record available*	
hwc_j_8_1 *(required)*	RTI	
hwc_j_8_1_1 *(required)*	STI	
hwc_j_8_2 *(required)*	TB	
hwc_j_8_3 *(required)*	Epilepsy	
hwc_j_8_4 *(required)*	Syphilis	
hwc_j_8_5 *(required)*	Thyroid status	
hwc_j_8_6 *(required)*	Blood pressure	
hwc_j_8_7 *(required)*	Blood sugar	
hwc_j_8_8 *(required)*	Height measured	
hwc_j_8_9 *(required)*	Weight measured	
hwc_j_8_10 *(required)*	Hemoglobin tested	
hwc_j_8_11 *(required)*	PHQ 2/Local instruments	
HWC > hwc_j_group > Preconception women (aged 18-35 years) identified with medical conditions & depressive symptoms following screening-<br>*Group relevant when: ${hwc_j_2} > 0*		
hwc_j_9_note	Please mention the Number of Preconception women (aged 18-35 years) identified with medical conditions & depressive symptoms following screening (Last 12 Month Data) against each<br>*Please fill 999 if no record available*	
hwc_j_9_1 *(required)*	RTI	
hwc_j_9_1_1 *(required)*	STI	
hwc_j_9_2 *(required)*	TB	
hwc_j_9_3 *(required)*	Epilepsy	
hwc_j_9_4 *(required)*	Syphilis	
hwc_j_9_5 *(required)*	Hypothyroidism (TSH > 5.5 IU/mL)	
hwc_j_9_6 *(required)*	Hyperthyroidism (TSH <0.4 IU/mL)	
hwc_j_9_7 *(required)*	Hypertension	
hwc_j_9_7_1 *(required)*	Hypotension	
hwc_j_9_8 *(required)*	Prediabetes (HbA1c 5.7% to 6.4%)	
hwc_j_9_9 *(required)*	Diabetes mellitus (HbA1c ≥6.5%)	
hwc_j_9_10 *(required)*	BMI<16 kg/m2	
hwc_j_9_11 *(required)*	BMI 16-18.49 kg/m2	
hwc_j_9_12 *(required)*	BMI 18.5-21 kg/m2	
hwc_j_9_13 *(required)*	Severe anemia (Hb <8 g/dL)	
hwc_j_9_14 *(required)*	Moderate anemia (Hb 8 to 10.9 g/dL)	
hwc_j_9_15 *(required)*	Depressive symptoms	
HWC > hwc_j_group > Preconception women (aged 18-35 years) managed<br>*Group relevant when: ${hwc_j_2} > 0*		
hwc_j_10_note	Please mention the Number of Preconception women (aged 18-35 years) managed for (Last 12 Month Data) the following conditions<br>*Please fill 999 if no record available*	
hwc_j_10_1 *(required)*	RTI	
hwc_j_10_1_1 *(required)*	STI	
hwc_j_10_2 *(required)*	TB	
hwc_j_10_3 *(required)*	Epilepsy	
hwc_j_10_4 *(required)*	Syphilis	
hwc_j_10_5 *(required)*	Hypothyroidism (TSH > 5.5 IU/mL)	
hwc_j_10_6 *(required)*	Hyperthyroidism (TSH <0.4 IU/mL)	
hwc_j_10_7 *(required)*	Hypertension	
hwc_j_10_7_1 *(required)*	Hypotension	
hwc_j_10_8 *(required)*	Prediabetes (HbA1c 5.7% to 6.4%)	
hwc_j_10_9 *(required)*	Diabetes mellitus (HbA1c ≥6.5%)	
hwc_j_10_10 *(required)*	BMI<16 kg/m2	
hwc_j_10_11 *(required)*	BMI 16-18.49 kg/m2	
hwc_j_10_12 *(required)*	BMI 18.5- 21 kg/m2	
hwc_j_10_13 *(required)*	Severe anemia (Hb <8 g/dL)	
hwc_j_10_14 *(required)*	Moderate anemia (Hb 8 to 10.9 g/dL)	
hwc_j_10_15 *(required)*	Depressive symptoms	
hwc_j_10_16 *(required)*	No. of preconception women who were given IFA as per recommendation	
hwc_j_10_17 *(required)*	No. of preconception women who were counselled for Nutrition	
hwc_j_10_18 *(required)*	No. of preconception women who were counselled for WASH intervention	
HWC > hwc_j_group > Pregnant women screened/tested for -		

Field	Question	Answer
Group relevant when: ${hwc_j_2} > 0 generated_note_name_569	Please mention the Number of Pregnant women screened for the following conditions (Last 12 Month Data) against each<br>*Please fill 999 if no record available*	
hwc_j_11_1 *(required)*	Blood grouping	
hwc_j_11_2 *(required)*	Complete Blood Count (CBC) at the time of registration	
hwc_j_11_3 *(required)*	HIV	
hwc_j_11_4 *(required)*	Syphilis	
hwc_j_11_5 *(required)*	Urine routine & microscopy	
hwc_j_11_6 *(required)*	Thyroid status / TSH	
hwc_j_11_7 *(required)*	Gestational Diabetes Mellitus (GDM) / OGTT	
hwc_j_11_8 *(required)*	Blood pressure	
hwc_j_11_9 *(required)*	Height measured in First Trimester	
hwc_j_11_10 *(required)*	Weight measured in First Trimester	
hwc_j_11_11 *(required)*	Weight measured in all trimesters	
hwc_j_11_12 *(required)*	Patients Health Questionnaire (PHQ-2) in each trimester	
hwc_j_11_13 *(required)*	Tested for Hemoglobin (Hb) 4 time during pregnancy	
HWC > hwc_j_group > Pregnant women identified with medical conditions & depressive symptoms following screening- (Last 12 Month Data) against each		
hwc_j_11 *(required)*	Number of Pregnant Women Registered for ANC Care in the last 12 months	
hwc_j_12_note	Please mention the Number of Pregnant women identified with medical conditions & depressive symptoms following screening (Last 12 Month Data)<br>*Please fill 999 if no record available*	
hwc_j_12_1 *(required)*	HIV	
hwc_j_12_2 *(required)*	Syphilis	
hwc_j_12_3 *(required)*	Hypothyroidism	
hwc_j_12_4 *(required)*	Hyperthyroidism	
hwc_j_12_5 *(required)*	Hypertension	
hwc_j_12_5_1 *(required)*	Hypotension	
hwc_j_12_6 *(required)*	Diabetes mellitus	
hwc_j_12_7 *(required)*	BMI < 18.5 kg/m2	
hwc_j_12_8 *(required)*	BMI < 25 kg/m2	
hwc_j_12_9 *(required)*	Pregnant women with inadequate gestational weight gain	
hwc_j_12_10 *(required)*	Severe anemia (Hb <7 g/dL)	
hwc_j_12_11 *(required)*	Moderate anemia (Hb 7 to 9.9 g/dL)	
hwc_j_12_12 *(required)*	Depressive symptoms	
HWC > hwc_j_group > Pregnant women managed for treatment		
hwc_j_14_note	Please mention the Number of Pregnant women managed for following conditions(Last 12 Month Data)<br>*Please fill 999 if no record available*	
hwc_j_14_1 *(required)*	HIV	
hwc_j_14_2 *(required)*	Syphilis	
hwc_j_14_3 *(required)*	Hypothyroidism	
hwc_j_14_4 *(required)*	Hyperthyroidism	
hwc_j_14_5 *(required)*	Hypertension	
hwc_j_14_5_1 *(required)*	Hypotension	
hwc_j_14_6 *(required)*	Diabetes mellitus	
hwc_j_14_7 *(required)*	Inadequate gestational weight gain (IGWG)	
hwc_j_14_8 *(required)*	Severe anemia (Hb <7 g/dL)	
hwc_j_14_9 *(required)*	Moderate anemia (Hb 7 to 9.9 g/dL)	
hwc_j_14_10 *(required)*	Depressive symptoms	
hwc_j_14_11 *(required)*	Other medical conditions	
hwc_j_14_12 *(required)*	No. of pregnant women who were given IFA as per recommendation	
hwc_j_14_13 *(required)*	No. of pregnant women who counselled for Nutrition	
hwc_j_14_14 *(required)*	No. of pregnant women who counselled for WASH intervention	
HWC > hwc_j_group > hwc_j_1_group		
hwc_j_15 *(required)*	Pregnant women referred during ANC	
hwc_j_16 *(required)*	Pregnant women with atleast 4-ANC check-ups	
hwc_j_17 *(required)*	Pregnant women with atleast 8-ANC check-ups	
hwc_j_18 *(required)*	Pregnant women given corticosteroids for preterm labor	
hwc_j_19 *(required)*	Registered antenatal mothers who came for delivery	
hwc_j_20 *(required)*	Institutional deliveries (In facility)	
hwc_j_20_1	Institutional deliveries(in other facility)	
hwc_j_21 *(required)*	Home deliveries	
hwc_j_22 *(required)*	Maternal deaths	

Field	Question	Answer
hwc_j_23 *(required)*	Live births	
hwc_j_24 *(required)*	Babies born with Very low birth weight (VLBW) < 1,500 grams	
hwc_j_25 *(required)*	Babies born with low birth weight (LBW) < 2,500 grams	
hwc_j_26 *(required)*	Preterm babies born (<37 weeks)	
hwc_j_27 *(required)*	Early neonatal deaths (within 7 days of birth)	
HWC > hwc_j_group > Other high-risk pregnancies identified during ANC-		
hwc_j_13_note	Please mention the Number of Other high-risk pregnancies identified during ANC- (Last 12 Month Data)<br>*Please fill 999 if no record available*	
hwc_j_13_1 *(required)*	Pregnant women with multiple pregnancy	
hwc_j_13_2 *(required)*	Pregnant women with previous bad obstetric history (BOH)*	
hwc_j_13_3 *(required)*	Pregnant women with other infections (Hepatitis, Malaria, etc.)	
hwc_j_13_4 *(required)*	Pregnant women with other medical conditions (Asthma, COPD, heart disease, etc.)	
HWC > hwc_j_group > Postnatal Women (0-6 Months) & 0-24 Months Infants & Child		
hwc_j_28_note	Please mention the Number of Postnatal Women (0-6 Months) & 0-24 Months Infants & Child (Last 12 Month Data)<br>*Please fill 999 if no record available*	
hwc_j_28_1 *(required)*	Newborns screened for birth defects (as per RBSK)	
hwc_j_28_2 *(required)*	Women received postpartum checkup between 48 hours and 14 days after institutional delivery	
hwc_j_28_3 *(required)*	HBNC visits performed as per guidelines	
hwc_j_28_4 *(required)*	Postnatal mothers consumed nutritional supplements as per recommendation	
hwc_j_28_5 *(required)*	Postnatal mothers screened for depressive symptoms	
hwc_j_28_6 *(required)*	Postnatal mothers who received appropriate psychosocial support	
hwc_j_28_7 *(required)*	Postnatal mothers who counselled for WASH intervention	
hwc_j_28_8 *(required)*	Postnatal mothers who were given IFA as per recommendation	
hwc_j_28_9 *(required)*	Postnatal mothers who counselled for Nutrition	
hwc_j_28_10 *(required)*	0-6 months Infants received kangaroo mother care (KMC) as applicable	
hwc_j_28_11 *(required)*	0-6 months Infants exclusively breastfed (EBF)	
hwc_j_28_12 *(required)*	0-6 months Infants (VLBW and LBW) who consumed Iron, as per recommendation	
hwc_j_28_13 *(required)*	0-6 months Infants who consumed Vitamin D 400 IU daily	
hwc_j_28_14 *(required)*	0-6 months Infants (Pre-term) who consumed calcium and vitamin D, as per recommendation	
hwc_j_28_15 *(required)*	6-24 months infants & children who consumed IFA, as per recommendation	
hwc_j_28_16 *(required)*	Infants 6-12 months with inadequate weight gain (<25th centile according to WHO weight velocity/month)	
hwc_j_28_17 *(required)*	Children 12-24 months with inadequate weight gain (<25th centile according to WHO weight velocity/month)	
hwc_j_28_18 *(required)*	Children (0-24 months) with adequate weight gain from birth till 2 years of age	
hwc_j_28_19 *(required)*	Family/Parents having 0-24 months infants & child counselled for WASH interventions	
hwc_j_28_20 *(required)*	Children (12-23 months) fully immunized	
hwc_j_28_21	Observational Remarks	
hwc_j_28_22	Respondent Remarks	
HWC > photo_group		
hwc_j_28_photo1	Document1	
hwc_j_28_photo2	Document2	
hwc_j_28_photo3	Document3	
hwc_j_28_photo4	Document4	
hwc_j_28_photo5	Document5	
hwc_j_28_photo6	Document6	
hwc_j_28_photo7	Document7	
hwc_j_28_photo8	Document8	
document	Please upload pdf document	

PHC Assessment form

<table>
<tr><th>Field</th><th>Question</th><th>Answer</th></tr>
<tr><td>worker</td><td>Worker Name</td><td>151 Abuhamza
152 Anmol Saini
153 Anshika Sahota
154 Ekta
155 Jyoti Devi
156 Kritika Thakur
157 Mehak Thakur
158 Poonam Devi
159 Riya Puri
160 Shivanshi
161 Varsha Kumari
162 Anchal Walia
163 Harshali
164 Kritika Puri</td></tr>
<tr><td>blocks (required)</td><td>Block Names</td><td>block_1 Amb
block_2 Basdehra
block_3 Gagret
block_4 Haroli
block_5 Thanakalan</td></tr>
<tr><td>phc_selected (required)</td><td>PHC</td><td>phc_1 PHC Akrot
phc_2 PHC Chaksrai
phc_3 PHC Chururu
phc_4 PHC Dharamshala Mahanta
phc_5 PHC Lohara
phc_6 PHC Shivpur
phc_7 PHC Basal
phc_8 PHC Basoli
phc_9 PHC Chalola
phc_10 PHC Dehlan
phc_11 PHC Amlehar
phc_12 PHC Badehra Rajputan
phc_13 PHC Marwari
phc_14 PHC Badehra
phc_15 PHC Bathri
phc_16 PHC Khad
phc_17 PHC Kuthar Beet
phc_18 PHC Palkwah
phc_19 PHC Panjawar
phc_20 PHC Saloh
phc_25 PHC Baliwal
phc_21 PHC Chamiari
phc_22 PHC Lathiani
phc_23 PHC Raipur Maidan
phc_24 PHC Sohari Takoli</td></tr>
<tr><td colspan="3">PHC</td></tr>
<tr><td colspan="3">PHC > PHC Identification[phc_label]</td></tr>
<tr><td>phc_a_1 (required)</td><td>Date of Assessment:
Response constrained to: .= today()</td><td></td></tr>
<tr><td>phc_a_6 (required)</td><td>Facility id:
Response constrained to: regex(., '^[0-9]{10}$')</td><td></td></tr>
<tr><td>phc_a_8 (required)</td><td>Facility Address with landmark</td><td></td></tr>
<tr><td>phc_a_10 (required)</td><td>Total Population covered by the facility</td><td></td></tr>
<tr><td>phc_a_11 (required)</td><td>Facility In-charge Name:
Response constrained to: not(regex(.,'^(.*)\d(.*)$'))</td><td></td></tr>
<tr><td>phc_a_12 (required)</td><td>Facility In-charge Designation:</td><td></td></tr>
<tr><td>phc_a_13 (required)</td><td>Facility In-charge Contact No.:
Response constrained to: regex(.,"^[6|7|8|9]\d{9}$")</td><td></td></tr>
<tr><td>phc_a_14</td><td>Facility In-charge Email Id.:</td><td></td></tr>
</table>

Field	Question	Answer
phc_a_15 *(required)*	Any Additional Respondent?	1 Yes<br>2 No
PHC > Additional Respondent<br>*Group relevant when: ${phc_a_15} =1*		
phc_a_15_2 *(required)*	Additional Respondent Name<br>*Response constrained to: not(regex(.,'^(.*)\d(.*)$'))*	
phc_a_15_3 *(required)*	Additional Respondent Designation	
PHC > phc_infra		
phc_b_1 *(required)*	Located within Main Habitation (*where the majority of the population resides)<br>*If No, Distance in Km*	1 Yes<br>2 No
phc_b_2 *(required)*	Connected to Motorable Road	1 Yes<br>2 No
phc_b_3 *(required)*	Designated Government PHC Building	1 Yes<br>2 No
phc_b_4 *(required)*	General Structure of Building	1 Newly Built<br>2 Newly Renovated<br>3 Old Structure<br>4 Falling apart
phc_b_5 *(required)*	Maintenance of Building	1 No maintenance<br>2 Once a year<br>3 Once in 3 years<br>99 Other (specify)
phc_b_6 *(required)*	Is electrical supply available?	1 24/7<br>3 Average duration of electricity supply per day<br>2 No
phc_b_6_or	Is power backup available?	1 Generator<br>2 Inverter<br>3 Solar<br>99 Other (Specify)<br>9999 None
phc_b_7 *(required)*	Main source of water supply?	1 Borewell<br>2 Piped water<br>99 Other with duration
phc_b_8 *(required)*	Toilet Facility	1 Yes<br>2 No
phc_b_9 *(required)*	Littering	1 Yes<br>2 No
PHC > Toilet & Other facilities		
phc_b_8_1 *(required)*	Facility available inside the toilet<br>*Question relevant when: selected(${phc_b_8} , '1')*<br>*Response constrained to: not(selected(${phc_b_8_1} , '9999') and count-selected(${phc_b_8_1}) > 1)*	1 Cleanliness<br>2 Toilet tap with running water<br>3 Flush working properly<br>4 Water bucket and mug inside the toilet<br>5 Washbasin with running water<br>6 Soap/liquid items handwash/hand rub<br>7 Disable friendly<br>8 Dustbin with yellow line (in female washroom)<br>9999 None
phc_b_8_2 *(required)*	Gender specific toilet facility<br>*Question relevant when: selected(${phc_b_8} , '1')*	1 Both (male & female separate) Available<br>2 Only Male Available<br>3 Only Female Available<br>4 Common Toilet Available<br>5 Availability of a disabled-friendly toilet

Field	Question	Answer
phc_b_10 *(required)*	Availability of general waste bins	1 Green<br>2 Blue<br>3 Both<br>99 Other
phc_b_11 *(required)*	Management (collection, segregation, storage, transportation, treatment and disposal) of BMW as per Bio-Medical Waste Management Rules, 2018	1 Yes<br>2 No
phc_b_11_bins *(required)*	Availability of colour coded BMW bins	1 Red<br>2 Yellow<br>3 Blue Puncture proof<br>4 White Puncture proof
PHC > Availability Status of Human Resource at PHC		
phc_c_2 *(required)*	Sanctioned (No.) MO MBBS	
phc_c_3 *(required)*	In position (No.) MO MBBS<br>*Question relevant when: ${phc_c_2} > 0*<br>*Response constrained to: . >= 0 and . <= ${phc_c_2}*	
PHC > Availability Status of Human Resource at PHC > MO MBBS (1)<br>*Group relevant when: ${phc_c_2} > 0 and ${phc_c_3} > 0*		(Repeated group)
phc_c_4 *(required)*	Status	1 Available<br>2 Long Leave<br>3 Deputed Somewhere Else
PHC > Availability Status of Human Resource at PHC > Training Status Of MO MBBS (1)		(Repeated group)
PHC > Availability Status of Human Resource at PHC > Training Status Of MO MBBS (1) > phc_mo_training		
generated_table_list_label_64	Training Status Of MO MBBS	
reserved_name_for_field_list_labels_65		1 Yes<br>2 No
phc_c_1_1 *(required)*	IUCD Training	1 Yes<br>2 No
phc_c_1_2 *(required)*	Breastfeeding Training	1 Yes<br>2 No
phc_c_1_3 *(required)*	Routine Immunization	1 Yes<br>2 No
phc_c_1_4 *(required)*	Emergency medicine	1 Yes<br>2 No
phc_c_1_5 *(required)*	NSSK	1 Yes<br>2 No
phc_c_1_6 *(required)*	Neonatal care Training	1 Yes<br>2 No
phc_c_1_7 *(required)*	SAANS Training (Under 5 Pneumonia management)	1 Yes<br>2 No
phc_c_1_13 *(required)*	National Health Program Training	1 Yes<br>2 No
phc_c_1_14 *(required)*	Anemia Mukt Bharat	1 Yes<br>2 No
phc_c_1_15 *(required)*	Infection Prevention and Control	1 Yes<br>2 No
phc_c_1_16 *(required)*	Other	1 Yes<br>2 No
PHC > Availability Status of Human Resource at PHC > Training Status Of MO MBBS (1) > phc_mo_training_others<br>*Group relevant when: ${phc_c_1_16} =1*		
phc_c_1_8	Please specify Training (others)<br>*Response constrained to: not(regex(.,'^(.*)\d(.*)$'))*	
phc_c_10 *(required)*	Sanctioned (No.) Staff Nurses	
phc_c_11 *(required)*	In position (No.) Staff Nurses<br>*Question relevant when: ${phc_c_10} > 0*<br>*Response constrained to: . >= 0 and . <= ${phc_c_10}*	
PHC > Availability Status of Human Resource at PHC > Staff Nurses (1)<br>*Group relevant when: ${phc_c_10} > 0 and ${phc_c_11} > 0*		(Repeated group)
phc_c_12 *(required)*	Status	1 Available<br>2 Long Leave<br>3 Deputed Somewhere Else

Field	Question	Answer
PHC > Availability Status of Human Resource at PHC > Training Status Of Staff Nurse (1)		(Repeated group)
PHC > Availability Status of Human Resource at PHC > Training Status Of Staff Nurse (1) > phc_nurse_training		
generated_table_list_label_87	Training Status Of Staff Nurse	
reserved_name_for_field_list_labels_88		1 Yes<br>2 No
phc_c_9_1 *(required)*	SBA	1 Yes<br>2 No
phc_c_9_2 *(required)*	NSSK	1 Yes<br>2 No
phc_c_9_3 *(required)*	IUCD	1 Yes<br>2 No
phc_c_9_4 *(required)*	Routine Immunization (Birth Dose)	1 Yes<br>2 No
phc_c_9_5 *(required)*	Cold chain management Training	1 Yes<br>2 No
phc_c_9_6 *(required)*	SAANS Training	1 Yes<br>2 No
phc_c_9_7 *(required)*	F-IMNCI	1 Yes<br>2 No
phc_c_9_9 *(required)*	Other	1 Yes<br>2 No
PHC > Availability Status of Human Resource at PHC > Training Status Of Staff Nurse (1) > phc_nurse_training_others<br>*Group relevant when: ${phc_c_9_9} =1*		
phc_c_9_8	Please specify Training (others)<br>*Response constrained to: not(regex(.,'^(.*)\d(.*)$'))*	
phc_c_14 *(required)*	Sanctioned (No.) Pharmacist	
phc_c_15 *(required)*	In position (No.) Pharmacist<br>*Question relevant when: ${phc_c_14} > 0*<br>*Response constrained to: . >= 0 and . <= ${phc_c_14}*	
PHC > Availability Status of Human Resource at PHC > Pharmacist (1)<br>*Group relevant when: ${phc_c_14} > 0 and ${phc_c_15} > 0*		(Repeated group)
phc_c_16 *(required)*	Status	1 Available<br>2 Long Leave<br>3 Deputed Somewhere Else
PHC > Availability Status of Human Resource at PHC > Training Status Of Pharmacist (1)		(Repeated group)
PHC > Availability Status of Human Resource at PHC > Training Status Of Pharmacist (1) > phc_pharmacist_training		
generated_table_list_label_107	Training Status Of Pharmacist	
reserved_name_for_field_list_labels_108		1 Yes<br>2 No
phc_c_13_1 *(required)*	DVDMS Training	1 Yes<br>2 No
phc_c_13_2 *(required)*	Stock management	1 Yes<br>2 No
phc_c_13_3 *(required)*	Cold chain management	1 Yes<br>2 No
phc_c_13_4 *(required)*	Tuberculosis (TB) Training	1 Yes<br>2 No
phc_c_13_5 *(required)*	BMW Management	1 Yes<br>2 No
phc_c_13_6 *(required)*	Sanitation Training	1 Yes<br>2 No
phc_c_13_7 *(required)*	E-Sanjeevni	1 Yes<br>2 No
phc_c_13_9 *(required)*	Other	1 Yes<br>2 No
PHC > Availability Status of Human Resource at PHC > Training Status Of Pharmacist (1) > phc_pharmacist_training_others<br>*Group relevant when: ${phc_c_13_9} =1*		
phc_c_13_8	Please specify Training (others)<br>*Response constrained to: not(regex(.,'^(.*)\d(.*)$'))*	
phc_c_18 *(required)*	Sanctioned (No.) Storekeeper	

Field	Question	Answer
phc_c_19 *(required)*	In position (No.) Storekeeper<br>*Question relevant when: ${phc_c_18} > 0*<br>*Response constrained to: . >= 0 and . <= ${phc_c_18}*	
PHC > Availability Status of Human Resource at PHC > Storekeeper (1)<br>*Group relevant when: ${phc_c_18} > 0 and ${phc_c_19} > 0*		(Repeated group)
phc_c_20 *(required)*	Status	1 Available<br>2 Long Leave<br>3 Deputed Somewhere Else
PHC > Availability Status of Human Resource at PHC > Training Status Of Storekeeper (1)		(Repeated group)
PHC > Availability Status of Human Resource at PHC > Training Status Of Storekeeper (1) > store_training		
generated_table_list_label_127	Training Status Of Storekeeper	
reserved_name_for_field_list_labels_128		1 Yes<br>2 No
phc_c_18_1 *(required)*	DVDMS Training	1 Yes<br>2 No
phc_c_18_2 *(required)*	Others	1 Yes<br>2 No
PHC > Availability Status of Human Resource at PHC > Training Status Of Storekeeper (1) > store_training_others<br>*Group relevant when: ${phc_c_18_2} =1*		
phc_c_18_3	Please specify Training (others)<br>*Response constrained to: not(regex(.,'^(.*)\d(.*)$'))*	
phc_c_22 *(required)*	Sanctioned (No.) Lab Technician	
phc_c_23 *(required)*	In position (No.) Lab Technician<br>*Question relevant when: ${phc_c_22} > 0*<br>*Response constrained to: . >= 0 and . <= ${phc_c_22}*	
PHC > Availability Status of Human Resource at PHC > Lab Technician (1)<br>*Group relevant when: ${phc_c_22} > 0 and ${phc_c_23} > 0*		(Repeated group)
phc_c_24 *(required)*	Status	1 Available<br>2 Long Leave<br>3 Deputed Somewhere Else
PHC > Availability Status of Human Resource at PHC > Training Status Of Lab Technician (1)		(Repeated group)
PHC > Availability Status of Human Resource at PHC > Training Status Of Lab Technician (1) > phc_labtech_training		
generated_table_list_label_141	Training Status Of Lab Technician	
reserved_name_for_field_list_labels_142		1 Yes<br>2 No
phc_c_21_1 *(required)*	NTEP Training	1 Yes<br>2 No
phc_c_21_2 *(required)*	Malaria Training	1 Yes<br>2 No
phc_c_21_3 *(required)*	Use of Rapid Test Kits	1 Yes<br>2 No
phc_c_21_4 *(required)*	Others	1 Yes<br>2 No
PHC > Availability Status of Human Resource at PHC > Training Status Of Lab Technician (1) > phc_labtech_training_others<br>*Group relevant when: ${phc_c_21_4} =1*		
phc_c_21_5	Please specify Training (others)<br>*Response constrained to: not(regex(.,'^(.*)\d(.*)$'))*	
phc_c_26 *(required)*	Sanctioned (No.) Health Worker (Female)/ANM	
phc_c_27 *(required)*	In position (No.) Health Worker (Female)/ANM<br>*Question relevant when: ${phc_c_26} > 0*<br>*Response constrained to: . >= 0 and . <= ${phc_c_26}*	
PHC > Availability Status of Human Resource at PHC > Health Worker (Female)/ANM (1)<br>*Group relevant when: ${phc_c_26} > 0 and ${phc_c_27} > 0*		(Repeated group)
phc_c_28 *(required)*	Status	1 Available<br>2 Long Leave<br>3 Deputed Somewhere Else
PHC > Availability Status of Human Resource at PHC > Training Status Of Health worker (Female)/ANM (1)		(Repeated group)
PHC > Availability Status of Human Resource at PHC > Training Status Of Health worker (Female)/ANM (1) > phc_hwf_training		
generated_table_list_label_157	Training Status Of Health worker (Female)/ANM	
reserved_name_for_field_list_labels_158		1 Yes<br>2 No

Field	Question	Answer
phc_c_25_1 *(required)*	Routine immunization	1 Yes<br>2 No
phc_c_25_2 *(required)*	SAANS Training (Under 5 Pneumonia management)	1 Yes<br>2 No
phc_c_25_3 *(required)*	HBNC Training	1 Yes<br>2 No
phc_c_25_4 *(required)*	Diarrhoea management Training (IDCF)	1 Yes<br>2 No
phc_c_25_5 *(required)*	MAA program	1 Yes<br>2 No
phc_c_25_6 *(required)*	IUCD	1 Yes<br>2 No
phc_c_25_7 *(required)*	NCD	1 Yes<br>2 No
phc_c_25_8 *(required)*	VHND	1 Yes<br>2 No
phc_c_25_9 *(required)*	VHSNC	1 Yes<br>2 No
phc_c_25_10 *(required)*	Eat right tool kit	1 Yes<br>2 No
phc_c_25_11 *(required)*	Other	1 Yes<br>2 No
PHC > Availability Status of Human Resource at PHC > Training Status Of Health worker (Female)/ANM (1) > phc_hwf_training_others<br>*Group relevant when: ${phc_c_25_11} =1*		
phc_c_25_12	Please specify Training (others)<br>*Response constrained to: not(regex(.,'^(.*)\d(.*)$'))*	
phc_c_30 *(required)*	Sanctioned (No.) Health Worker (Male)/ Health Assistant (Male)	
phc_c_31 *(required)*	In position (No.) Health Worker (Male)/ Health Assistant (Male)<br>*Question relevant when: ${phc_c_30} > 0*<br>*Response constrained to: . >= 0 and . <= ${phc_c_30}*	
PHC > Availability Status of Human Resource at PHC > Health Worker (Male)/ Health Assistant (Male) (1)<br>*Group relevant when: ${phc_c_30} > 0 and ${phc_c_31} > 0*		(Repeated group)
phc_c_32 *(required)*	Status	1 Available<br>2 Long Leave<br>3 Deputed Somewhere Else
PHC > Availability Status of Human Resource at PHC > Training Status Of Health Worker (Male)/Health Assistant (Male) (1)		(Repeated group)
PHC > Availability Status of Human Resource at PHC > Training Status Of Health Worker (Male)/Health Assistant (Male) (1) > phc_hwm_training		
generated_table_list_label_180	Training Status Of Health Worker (Male)/Health Assistant (Male)	
reserved_name_for_field_list_labels_181		1 Yes<br>2 No
phc_c_29_1 *(required)*	TB	1 Yes<br>2 No
phc_c_29_2 *(required)*	Malaria	1 Yes<br>2 No
phc_c_29_3 *(required)*	Routine Immunization	1 Yes<br>2 No
phc_c_29_4 *(required)*	Water testing	1 Yes<br>2 No
phc_c_29_5 *(required)*	Other	1 Yes<br>2 No
PHC > Availability Status of Human Resource at PHC > Training Status Of Health Worker (Male)/Health Assistant (Male) (1) > phc_hwm_training_others<br>*Group relevant when: ${phc_c_29_5} =1*		
phc_c_29_6 *(required)*	Please specify Training (others)<br>*Response constrained to: not(regex(.,'^(.*)\d(.*)$'))*	
phc_c_34 *(required)*	Sanctioned (No.) Female health supervisor	
phc_c_35 *(required)*	In position (No.) Female health supervisor<br>*Question relevant when: ${phc_c_34} > 0*<br>*Response constrained to: . >= 0 and . <= ${phc_c_34}*	
PHC > Availability Status of Human Resource at PHC > Female health supervisor (1)<br>*Group relevant when: ${phc_c_34} > 0 and ${phc_c_35} > 0*		(Repeated group)

Field	Question	Answer
phc_c_36 *(required)*	Status	1 Available<br>2 Long Leave<br>3 Deputed Somewhere Else
PHC > Availability Status of Human Resource at PHC > Female health supervisor (1)		(Repeated group)
PHC > Availability Status of Human Resource at PHC > Female health supervisor (1) > phc_lhv_training		
generated_table_list_label_197	Female health supervisor	
reserved_name_for_field_list_labels_198		1 Yes<br>2 No
phc_c_33_1 *(required)*	Cold chain management	1 Yes<br>2 No
phc_c_33_2 *(required)*	Others	1 Yes<br>2 No
PHC > Availability Status of Human Resource at PHC > Female health supervisor (1) > phc_lhv_training_others<br>*Group relevant when: ${phc_c_33_2} =1*		
phc_c_33_3 *(required)*	Please specify Training (others)<br>*Response constrained to: not(regex(.,'^(.*)\d(.*)$'))*	
phc_c_38 *(required)*	Sanctioned (No.) Health Educator/ Counsellor	
phc_c_39 *(required)*	In position (No.) Health Educator/ Counsellor<br>*Question relevant when: ${phc_c_38} > 0*<br>*Response constrained to: . >= 0 and . <= ${phc_c_38}*	
PHC > Availability Status of Human Resource at PHC > Health Educator/ Counsellor (1)<br>*Group relevant when: ${phc_c_38} > 0 and ${phc_c_39} > 0*		(Repeated group)
phc_c_40 *(required)*	Status	1 Available<br>2 Long Leave<br>3 Deputed Somewhere Else
PHC > Availability Status of Human Resource at PHC > Training Status Of Health Educator/ Counsellor (1)		(Repeated group)
PHC > Availability Status of Human Resource at PHC > Training Status Of Health Educator/ Counsellor (1) > phc_hec_training		
generated_table_list_label_211	Training Status Of Health Educator/ Counsellor	
reserved_name_for_field_list_labels_212		1 Yes<br>2 No
phc_c_37_1 *(required)*	HBNC	1 Yes<br>2 No
phc_c_37_2 *(required)*	HBYC	1 Yes<br>2 No
phc_c_37_3 *(required)*	VHND	1 Yes<br>2 No
phc_c_37_4 *(required)*	HIV	1 Yes<br>2 No
phc_c_37_5 *(required)*	Routine Immunization	1 Yes<br>2 No
phc_c_37_6 *(required)*	Adolescent Health	1 Yes<br>2 No
phc_c_37_7 *(required)*	Maternal health	1 Yes<br>2 No
phc_c_37_8 *(required)*	Anemia Mukt Bharat	1 Yes<br>2 No
phc_c_37_9 *(required)*	Under 5 Pneumonia Management Program	1 Yes<br>2 No
phc_c_37_10 *(required)*	Intensive Diarrhoea Control Fortnight (IDCF)	1 Yes<br>2 No
phc_c_37_11 *(required)*	MAA program	1 Yes<br>2 No
phc_c_37_12 *(required)*	IEC Planning & Implementation Training	1 Yes<br>2 No
phc_c_37_13 *(required)*	Other	1 Yes<br>2 No
PHC > Availability Status of Human Resource at PHC > Training Status Of Health Educator/ Counsellor (1) > phc_hec_training_others<br>*Group relevant when: ${phc_c_37_13} =1*		
phc_c_37_14 *(required)*	Please specify Training (others)<br>*Response constrained to: not(regex(.,'^(.*)\d(.*)$'))*	

Field	Question	Answer
phc_c_42 *(required)*	Sanctioned (No.) Data Entry Operator	
phc_c_43 *(required)*	In position (No.) Data Entry Operator<br>*Question relevant when: ${phc_c_42} > 0*<br>*Response constrained to: . >= 0 and . <= ${phc_c_42}*	
PHC > Availability Status of Human Resource at PHC > Data Entry Operator (1)<br>*Group relevant when: ${phc_c_42} > 0 and ${phc_c_43} > 0*		(Repeated group)
phc_c_44 *(required)*	Status	1 Available<br>2 Long Leave<br>3 Deputed Somewhere Else
phc_c_45 *(required)*	Any other staff?	1 Yes<br>2 No
PHC > Availability Status of Human Resource at PHC > anyother_group<br>*Group relevant when: ${phc_c_45} =1*		
any_other1	Name<br>*Response constrained to: not(regex(.,'^(.*)\d(.*)$'))*	
any_other2	Designation<br>*Response constrained to: not(regex(.,'^(.*)\d(.*)$'))*	
PHC > phc_infra2		
phc_b_12	Quality Assurance certification/services	
reserved_name_for_field_list_labels_243		1 Yes<br>2 No
phc_b_13 *(required)*	Kayakalp	1 Yes<br>2 No
phc_b_14 *(required)*	NQAS	1 Yes<br>2 No
phc_b_16 *(required)*	SUMAN	1 Yes<br>2 No
PHC > phc_infra3		
phc_b_17 *(required)*	Emergency area and Triage services available	1 Yes<br>2 No
phc_b_18 *(required)*	OPD Room services<br>*Response constrained to: not(selected(${phc_b_18} , '9999') and count-selected(${phc_b_18}) > 1)*	1 Yes<br>2 No
PHC > OPD room Infrastructure Status		
phc_b_19 *(required)*	OPD Room services - Available facilities<br>*Question relevant when: ${phc_b_18} =1*<br>*Response constrained to: not(selected(${phc_b_19} , '9999') and count-selected(${phc_b_19}) > 1)*	1 Adequate table, chairs, bench and stool<br>2 Examination table<br>3 Footstep<br>4 Washbasin with Running Water Supply<br>5 Waiting area with sitting facility<br>9999 None of the above
phc_b_20 *(required)*	Laboratory	1 Yes<br>2 No
PHC > Laboratory Infrastructure Status		
phc_b_21 *(required)*	Laboratory - Available facilities<br>*Question relevant when: ${phc_b_20} =1*<br>*Response constrained to: not(selected(${phc_b_21} , '9999') and count-selected(${phc_b_21}) > 1)*	1 Adequate table, chairs, bench and stool<br>2 Washbasin with Running Water Supply<br>3 Adequate area for sample collection area<br>4 Adequate areas for carrying out diagnostic activities<br>5 Adequate area for keeping equipment and storage of reagents and records<br>9999 None of the above
phc_b_22 *(required)*	ANC Room	1 Yes<br>2 No
PHC > ANC Room Infrastructure Status		

Field	Question	Answer
phc_b_23 *(required)*	ANC Room services - Available facilities<br>*Question relevant when: ${phc_b_22} =1*<br>*Response constrained to: not(selected(${phc_b_23} , '9999') and count-selected(${phc_b_23}) > 1)*	1 Adequate table, chairs, bench and stool<br>2 Examination table<br>5 Footstep<br>3 Washbasin with Running Water Supply<br>4 Waiting area with sitting facility<br>9999 None of the above
phc_b_24 *(required)*	Labour Room services	1 Yes<br>2 No
PHC > Labour Room Infrastructure Status		
phc_b_25 *(required)*	Labour Room services - Available facilities<br>*Question relevant when: ${phc_b_24} =1*<br>*Response constrained to: not(selected(${phc_b_25} , '9999') and count-selected(${phc_b_25}) > 1)*	1 Labour tables<br>2 Washbasin with Running Water Supply<br>3 Newborn care corner(NBCC)<br>4 Elbow tap<br>9999 None of the above
phc_b_27 *(required)*	Immunization Room services	1 Yes<br>2 No
PHC > Availability Status of Equipment at phc-SHC		
phc_d_1 *(required)*	24x7 Emergency Services with Triage, Resuscitation & Stabilization	1 Yes<br>2 No
PHC > Availability Status of Equipment at phc-SHC > phc_triage<br>*Group relevant when: ${phc_d_1} =1*		
phc_d_2 *(required)*	Emergency Drug Tray<br>*If No, specify missing medicines*	1 Yes<br>2 No
phc_d_3 *(required)*	Oxygen Cylinder<br>*If yes, please specify number of functional equipments*	1 Yes<br>2 No
phc_d_68 *(required)*	O2 concentrator<br>*If yes, please specify number of functional equipments*	1 Yes<br>2 No
phc_d_4 *(required)*	Suction Machine (Electrical)<br>*If yes, please specify number of functional equipments*	1 Yes<br>2 No
phc_d_4_1 *(required)*	Suction Machine (Foot-operated)<br>*If yes, please specify number of functional equipments*	1 Yes<br>2 No
phc_d_7 *(required)*	Open care/Radiant warmer<br>*If yes, please specify number of functional equipments*	1 Yes<br>2 No
phc_d_8 *(required)*	Pulse Oximeter Adult<br>*If yes, please specify number of functional equipments*	1 Yes<br>2 No
phc_d_69 *(required)*	Pulse Oximeter Pediatric<br>*If yes, please specify number of functional equipments*	1 Yes<br>2 No
phc_d_9 *(required)*	Self-Inflating Bag and Mask Adult<br>*If yes, please specify number of functional equipments*	1 Yes<br>2 No
phc_d_77 *(required)*	Self-Inflating Bag and Mask Child<br>*If yes, please specify number of functional equipments*	1 Yes<br>2 No
phc_d_82 *(required)*	Self-Inflating Bag and Mask Neonatal Size(0,1)<br>*If yes, please specify number of functional equipments*	1 Yes<br>2 No
phc_d_10 *(required)*	Laryngoscope<br>*If yes, please specify number of functional equipments*	1 Yes<br>2 No
phc_d_80 *(required)*	ET Intubation Tubes all Sizes<br>*If yes, please specify number of functional equipments*	1 Yes<br>2 No
phc_d_11 *(required)*	ANC Services	1 Yes<br>2 No
PHC > Availability Status of Equipment at phc-SHC > phc_anc_service<br>*Group relevant when: ${phc_d_11} =1*		
phc_d_12 *(required)*	Blood Pressure Monitor (Digital)<br>*If yes, please specify number of functional equipments*	1 Yes<br>2 No
phc_d_12_1 *(required)*	Blood Pressure Monitor (Manual)<br>*If yes, please specify number of functional equipments*	1 Yes<br>2 No

Field	Question	Answer
phc_d_13 *(required)*	Stethoscope<br>*If yes, please specify number of functional equipments*	1 Yes<br>2 No
phc_d_14 *(required)*	Thermometer (Digital)<br>*If yes, please specify number of functional equipments*	1 Yes<br>2 No
phc_d_70 *(required)*	Clinical Thermometer<br>*If yes, please specify number of functional equipments*	1 Yes<br>2 No
phc_d_15 *(required)*	Adult Weighing Scale (Digital)<br>*If yes, please specify number of functional equipments*	1 Yes<br>2 No
phc_d_15_1 *(required)*	Adult Weighing Scale (Analogue)<br>*If yes, please specify number of functional equipments*	1 Yes<br>2 No
phc_d_16 *(required)*	Stadiometer<br>*If yes, please specify number of functional equipments*	1 Yes<br>2 No
phc_d_17 *(required)*	Measuring Tape<br>*If yes, please specify number of functional equipments*	1 Yes<br>2 No
phc_d_18 *(required)*	Examination Lamp with White Light<br>*If yes, please specify number of functional equipments*	1 Yes<br>2 No
phc_d_19 *(required)*	Fetoscope<br>*If yes, please specify number of functional equipments*	1 Yes<br>2 No
phc_d_71 *(required)*	Fetal Doppler Monitor<br>*If yes, please specify number of functional equipments*	1 Yes<br>2 No
phc_d_20 *(required)*	Cusco's Speculum<br>*If yes, please specify number of functional equipments*	1 Yes<br>2 No
phc_d_72 *(required)*	Sims Speculum<br>*If yes, please specify number of functional equipments*	1 Yes<br>2 No
phc_d_21 *(required)*	Pulse oximeter (adult)<br>*If yes, please specify number of functional equipments*	1 Yes<br>2 No
phc_d_22 *(required)*	Pediatric Services	1 Yes<br>2 No
PHC > Availability Status of Equipment at phc-SHC > Pediatric Services<br>*Group relevant when: ${phc_d_22} =1*		
phc_d_23 *(required)*	Pediatric Stethoscope<br>*If yes, please specify number of functional equipments*	1 Yes<br>2 No
phc_d_79 *(required)*	Clinical Thermometer<br>*If yes, please specify number of functional equipments*	1 Yes<br>2 No
phc_d_24 *(required)*	Digital Thermometer<br>*If yes, please specify number of functional equipments*	1 Yes<br>2 No
phc_d_73 *(required)*	Infrared Thermometer<br>*If yes, please specify number of functional equipments*	1 Yes<br>2 No
phc_d_25 *(required)*	Infantometer<br>*If yes, please specify number of functional equipments*	1 Yes<br>2 No
phc_d_26 *(required)*	Baby Weighing Scale (Electronic)<br>*If yes, please specify number of functional equipments*	1 Yes<br>2 No
phc_d_26_1 *(required)*	Baby Weighing Scale (Analogue)<br>*If yes, please specify number of functional equipments*	1 Yes<br>2 No
phc_d_27 *(required)*	Pulse oximeter (Pediatric)<br>*If yes, please specify number of functional equipments*	1 Yes<br>2 No
phc_d_28 *(required)*	Phototherapy machine<br>*If yes, please specify number of functional equipments*	1 Yes<br>2 No
phc_d_29 *(required)*	Pediatric Resuscitation Kit<br>*If no, please specify missing equipments*	1 Yes<br>2 No
phc_d_30 *(required)*	Breast pump (Manual)<br>*If yes, please specify number of functional equipments*	1 Yes<br>2 No
phc_d_30_1 *(required)*	Breast pump (Electric)<br>*If yes, please specify number of functional equipments*	1 Yes<br>2 No
phc_d_31 *(required)*	Labour Room	1 Yes<br>2 No
PHC > Availability Status of Equipment at phc-SHC > Labour Room<br>*Group relevant when: ${phc_d_31} =1*		
phc_d_32 *(required)*	Foetal doppler	1 Yes

Field	Question	Answer
	If yes, please specify number of functional equipments	2 No
phc_d_34 *(required)*	ET tubes<br>*If yes, please specify number of functional equipments*	1 Yes<br>2 No
phc_d_36 *(required)*	Laryngoscopes (LED)<br>*If yes, please specify number of functional equipments*	1 Yes<br>2 No
phc_d_37 *(required)*	Suction Machine (Electrical)<br>*If yes, please specify number of functional equipments*	1 Yes<br>2 No
phc_d_74 *(required)*	Suction Machine (Foot-operated)<br>*If yes, please specify number of functional equipments*	1 Yes<br>2 No
phc_d_38 *(required)*	Examination light<br>*If yes, please specify number of functional equipments*	1 Yes<br>2 No
phc_d_78 *(required)*	Open care Radiant warmer<br>*If yes, please specify number of functional equipments*	1 Yes<br>2 No
phc_d_41 *(required)*	Vacuum extractors<br>*If yes, please specify number of functional equipments*	1 Yes<br>2 No
phc_d_42 *(required)*	Pulse Oximeter (Adult)<br>*If yes, please specify number of functional equipments*	1 Yes<br>2 No
phc_d_75 *(required)*	Pulse Oximeter (Pediatric Probes)<br>*If yes, please specify number of functional equipments*	1 Yes<br>2 No
phc_d_43 *(required)*	Self-Inflating Bag and Mask Neonatal Size(0,1)<br>*If yes, please specify number of functional equipments*	1 Yes<br>2 No
phc_d_44 *(required)*	NORMAL DELIVERY TRAY (equipments)<br>*Yes, with all functional equipments<br>No, specify missing equipments*	1 Yes<br>2 No
phc_d_45 *(required)*	Episiotomy kit<br>*Yes, with all functional equipments<br>No, specify missing equipments*	1 Yes<br>2 No
phc_d_76 *(required)*	Crash Cart with emergency medicines<br>*If No, specify missing medicines*	1 Yes<br>2 No
phc_d_49 *(required)*	Feeding tube<br>*If yes, please specify number of functional equipments*	1 Yes<br>2 No
phc_d_50 *(required)*	Suction catheter<br>*If yes, please specify number of functional equipments*	1 Yes<br>2 No
phc_d_60 *(required)*	Cold chain room services	1 Yes<br>2 No
PHC > Availability Status of Equipment at phc-SHC > Cold chain room services<br>*Group relevant when: ${phc_d_60} =1*		
phc_d_61 *(required)*	ILR with Voltage Stabilizer<br>*If yes, please specify number of functional equipments*	1 Yes<br>2 No
phc_d_62 *(required)*	DF Small with Voltage Stabilizer<br>*If yes, please specify number of functional equipments*	1 Yes<br>2 No
phc_d_64 *(required)*	Thermometer<br>*If yes, please specify number of functional equipments*	1 Yes<br>2 No
phc_d_65 *(required)*	Vaccine Carriers with Ice Packs<br>*If yes, please specify number of functional equipments*	1 Yes<br>2 No
phc_d_66 *(required)*	Cold Box<br>*If yes, please specify number of functional equipments*	1 Yes<br>2 No
lab_facility	Is lab service available ?	1 Yes<br>2 No
lab_services	Type of facility<br>*Question relevant when: ${lab_facility} =1*	1 Goverment<br>2 KRSNAA/Empanelled
PHC > Availability Status of Diagnostic tests/services at phc-SHC<br>*Group relevant when: selected(${lab_services} ,1) or selected(${lab_services} ,2)*		
phc_e_1 *(required)*	Haemoglobin - Required Equipment & Reagents are available in the Laboratory?<br>*If Yes, what method was used?*	1 Tested in facility<br>3 Only sample collection<br>2 Not Available
phc_e_2 *(required)*	Platelet Count<br>*If Yes, what method was used?*	1 Tested in facility<br>3 Only sample collection<br>2 Not Available
phc_e_3 *(required)*	Complete Blood Count with ESR<br>*If Yes, what method was used?*	1 Tested in facility<br>3 Only sample collection

Field	Question	Answer
		2 Not Available
phc_e_4 *(required)*	Blood Group And Rh Typing<br>*If Yes, what method was used?*	1 Tested in facility<br>3 Only sample collection<br>2 Not Available
phc_e_27 *(required)*	Blood Cross Matching<br>*If Yes, what method was used?*	1 Tested in facility<br>3 Only sample collection<br>2 Not Available
phc_e_6 *(required)*	Bleeding Time and Clotting Time<br>*If Yes, what method was used?*	1 Tested in facility<br>3 Only sample collection<br>2 Not Available
phc_e_7 *(required)*	MP slide method<br>*If Yes, what method was used?*	1 Tested in facility<br>3 Only sample collection<br>2 Not Available
phc_e_8 *(required)*	Malaria rapid test<br>*If Yes, what method was used?*	1 Tested in facility<br>3 Only sample collection<br>2 Not Available
phc_e_26 *(required)*	Urine test for pregnancy(UPT)<br>*If Yes, what method was used?*	1 Tested in facility<br>3 Only sample collection<br>2 Not Available
phc_e_10 *(required)*	Urine Microscopy<br>*If Yes, what method was used?*	1 Tested in facility<br>3 Only sample collection<br>2 Not Available
phc_e_11 *(required)*	Urine for microalbumin<br>*If Yes, what method was used?*	1 Tested in facility<br>3 Only sample collection<br>2 Not Available
phc_e_12 *(required)*	Stool for ova and cyst<br>*If Yes, what method was used?*	1 Tested in facility<br>3 Only sample collection<br>2 Not Available
phc_e_13 *(required)*	Stool for Occult Blood<br>*If Yes, what method was used?*	1 Tested in facility<br>3 Only sample collection<br>2 Not Available
phc_e_14 *(required)*	RPR/VDRL test for syphilis<br>*If Yes, what method was used?*	1 Tested in facility<br>3 Only sample collection<br>2 Not Available
phc_e_15 *(required)*	HIV test (Antibodies 1/2 and HIV 1/2)<br>*If Yes, what method was used?*	1 Tested in facility<br>3 Only sample collection<br>2 Not Available
phc_e_16 *(required)*	Hepatitis B surface antigen test<br>*If Yes, what method was used?*	1 Tested in facility<br>3 Only sample collection<br>2 Not Available
phc_e_17 *(required)*	HCV Antibody Test (Anti HCV)<br>*If Yes, what method was used?*	1 Tested in facility<br>3 Only sample collection<br>2 Not Available
phc_e_19 *(required)*	Sputum, pus etc. for AFB<br>*If Yes, what method was used?*	1 Tested in facility<br>3 Only sample collection<br>2 Not Available
phc_e_20 *(required)*	Typhoid test (IgM)<br>*If Yes, what method was used?*	1 Tested in facility<br>3 Only sample collection<br>2 Not Available
phc_e_21 *(required)*	Blood sugar -RBS (Random blood sugar)<br>*If Yes, what method was used?*	1 Tested in facility<br>3 Only sample collection<br>2 Not Available
phc_e_22 *(required)*	Oral Glucose Tolerance Test (OGTT)<br>*If Yes, what method was used?*	1 Tested in facility<br>3 Only sample collection<br>2 Not Available
phc_e_23 *(required)*	Wet mount and Gram stain for RTI/STD<br>*If Yes, what method was used?*	1 Tested in facility<br>3 Only sample collection<br>2 Not Available
phc_e_24 *(required)*	S. TSH (including for newborn screening) also include T3,T4 , TSH FOR PW<br>*If Yes, what method was used?*	1 Tested in facility

Field	Question	Answer
		3 Only sample collection<br>2 Not Available
phc_e_25 *(required)*	Urine Dipstick Test<br>*If Yes, what method was used?*	1 Tested in facility<br>3 Only sample collection<br>2 Not Available
phc_e_24_remarks *(required)*	Remarks (if any)	
PHC > phc_iec		
generated_table_list_label_459	Availability Status of IEC Material/algorithms and management charts related to the national/state programs in local script.	
reserved_name_for_field_list_labels_460		1 Program being implemented<br>2 IEC material Available<br>3 IEC material displayed<br>4 Program not being implemented
phc_f_1 *(required)*	Family Planning- Mission Parivar Vikas	1 Program being implemented<br>2 IEC material Available<br>3 IEC material displayed<br>4 Program not being implemented
phc_f_2 *(required)*	Anemia- AMB charts and algorithms	1 Program being implemented<br>2 IEC material Available<br>3 IEC material displayed<br>4 Program not being implemented
phc_f_3 *(required)*	Adolescent health programs	1 Program being implemented<br>2 IEC material Available<br>3 IEC material displayed<br>4 Program not being implemented
phc_f_4 *(required)*	Nutrition- POSHAN Abhiyan	1 Program being implemented<br>2 IEC material Available<br>3 IEC material displayed<br>4 Program not being implemented
phc_f_5 *(required)*	Sanitation and Hygiene: Swachh Bharat Mission Gramin, handwashing	1 Program being implemented<br>2 IEC material Available<br>3 IEC material displayed<br>4 Program not being implemented
phc_f_6 *(required)*	ANC	1 Program being implemented<br>2 IEC material Available<br>3 IEC material displayed<br>4 Program not being implemented
phc_f_7 *(required)*	PMSMA	1 Program being implemented<br>2 IEC material Available<br>3 IEC material displayed<br>4 Program not being implemented
phc_f_8 *(required)*	Management of PPH, Shock	1 Program being implemented<br>2 IEC material Available<br>3 IEC material displayed<br>4 Program not being implemented
phc_f_9 *(required)*	Newborn Resuscitation, NSSK	1 Program being implemented<br>2 IEC material Available<br>3 IEC material displayed<br>4 Program not being implemented
phc_f_10 *(required)*	HBNC	1 Program being implemented<br>2 IEC material Available

Field	Question	Answer
		3 IEC material displayed<br>4 Program not being implemented
phc_f_11 *(required)*	Childhood care- MAA, HBYC	1 Program being implemented<br>2 IEC material Available<br>3 IEC material displayed<br>4 Program not being implemented
phc_f_12 *(required)*	Immunization	1 Program being implemented<br>2 IEC material Available<br>3 IEC material displayed<br>4 Program not being implemented
phc_f_13 *(required)*	Breastfeeding	1 Program being implemented<br>2 IEC material Available<br>3 IEC material displayed<br>4 Program not being implemented
phc_f_14 *(required)*	Diarrhea management- Zinc, ORS	1 Program being implemented<br>2 IEC material Available<br>3 IEC material displayed<br>4 Program not being implemented
phc_f_15 *(required)*	Pneumonia Management	1 Program being implemented<br>2 IEC material Available<br>3 IEC material displayed<br>4 Program not being implemented
phc_f_16 *(required)*	JSY	1 Program being implemented<br>2 IEC material Available<br>3 IEC material displayed<br>4 Program not being implemented
phc_f_17 *(required)*	JSSK	1 Program being implemented<br>2 IEC material Available<br>3 IEC material displayed<br>4 Program not being implemented
phc_f_18 *(required)*	Ambulance (National Ambulance Services)	1 Program being implemented<br>2 IEC material Available<br>3 IEC material displayed<br>4 Program not being implemented
phc_f_19 *(required)*	Telemedicine	1 Program being implemented<br>2 IEC material Available<br>3 IEC material displayed<br>4 Program not being implemented
phc_f_20 *(required)*	Danger Signs (Maternal and Neonatal care)	1 Program being implemented<br>2 IEC material Available<br>3 IEC material displayed<br>4 Program not being implemented
PHC > phc_register_group		
phc_f_10_r *(required)*	General OPD Register	1 Yes<br>2 No
phc_f_11_r *(required)*	Pre-conception Women Register	1 Yes<br>2 No
phc_f_12_r *(required)*	ANC Register	1 Yes<br>2 No

Field	Question	Answer
phc_f_13_r *(required)*	Pediatric/0-24 Months Infants & Children Register	1 Yes<br>2 No
phc_f_14_r *(required)*	Labour Room Register	1 Yes<br>2 No
phc_f_15_r *(required)*	Laboratory Record	1 Yes<br>2 No
phc_f_16_r *(required)*	Birth/Death Register	1 Yes<br>2 No
phc_f_17_r *(required)*	Referral Register	1 Yes<br>2 No
PHC > phc_g_group		
PHC > phc_g_group > phc_maintenance_record_data<br>*Group relevant when: ${phc_f_10_r} =1*		
generated_table_list_label_492	General OPD Register	
reserved_name_for_field_list_labels_493		1 Indicator is filled<br>2 Indicator Not filled<br>3 No indicator available
phc_g_1_1 *(required)*	Date	1 Indicator is filled<br>2 Indicator Not filled<br>3 No indicator available
phc_g_1_2 *(required)*	Patient ID	1 Indicator is filled<br>2 Indicator Not filled<br>3 No indicator available
phc_g_1_3 *(required)*	Name	1 Indicator is filled<br>2 Indicator Not filled<br>3 No indicator available
phc_g_1_4 *(required)*	Age	1 Indicator is filled<br>2 Indicator Not filled<br>3 No indicator available
phc_g_1_5 *(required)*	Sex	1 Indicator is filled<br>2 Indicator Not filled<br>3 No indicator available
phc_g_1_6 *(required)*	Address	1 Indicator is filled<br>2 Indicator Not filled<br>3 No indicator available
phc_g_1_13 *(required)*	Contact details	1 Indicator is filled<br>2 Indicator Not filled<br>3 No indicator available
phc_g_1_7 *(required)*	Marital status	1 Indicator is filled<br>2 Indicator Not filled<br>3 No indicator available
phc_g_1_8 *(required)*	Condition for which visiting OPD	1 Indicator is filled<br>2 Indicator Not filled<br>3 No indicator available
phc_g_1_9 *(required)*	Treatment provided	1 Indicator is filled<br>2 Indicator Not filled<br>3 No indicator available
phc_g_1_10 *(required)*	Investigations done	1 Indicator is filled<br>2 Indicator Not filled<br>3 No indicator available
phc_g_1_11 *(required)*	Follow-up visit date	1 Indicator is filled<br>2 Indicator Not filled<br>3 No indicator available
phc_g_1_12 *(required)*	Referrals mode.	1 Indicator is filled<br>2 Indicator Not filled<br>3 No indicator available
PHC > phc_g_group > phc_preconception_record_data<br>*Group relevant when: ${phc_f_11_r} =1*		
generated_table_list_label_507	Pre-conception Women Register	

Field	Question	Answer
reserved_name_for_field_list_labels_508		1 Indicator is filled<br>2 Indicator Not filled<br>3 No indicator available
phc_g_2_1 *(required)*	Date of visit	1 Indicator is filled<br>2 Indicator Not filled<br>3 No indicator available
phc_g_2_2 *(required)*	Woman ID	1 Indicator is filled<br>2 Indicator Not filled<br>3 No indicator available
phc_g_2_3 *(required)*	Name	1 Indicator is filled<br>2 Indicator Not filled<br>3 No indicator available
phc_g_2_4 *(required)*	Age	1 Indicator is filled<br>2 Indicator Not filled<br>3 No indicator available
phc_g_2_5 *(required)*	Address	1 Indicator is filled<br>2 Indicator Not filled<br>3 No indicator available
phc_g_2_11 *(required)*	Contact details	1 Indicator is filled<br>2 Indicator Not filled<br>3 No indicator available
phc_g_2_6 *(required)*	Height	1 Indicator is filled<br>2 Indicator Not filled<br>3 No indicator available
phc_g_2_7 *(required)*	Weight	1 Indicator is filled<br>2 Indicator Not filled<br>3 No indicator available
phc_g_2_8 *(required)*	BMI	1 Indicator is filled<br>2 Indicator Not filled<br>3 No indicator available
phc_g_2_9 *(required)*	Medical history (e.g.Anemia,DM,HT,RTI,Thyroid) and screening results of the same conditions	1 Indicator is filled<br>2 Indicator Not filled<br>3 No indicator available
phc_g_2_10 *(required)*	Place/facility were the conditions tracked during follow up visits	1 Indicator is filled<br>2 Indicator Not filled<br>3 No indicator available
PHC > phc_g_group > phc_anc_record_data<br>*Group relevant when: ${phc_f_12_r} =1*		
generated_table_list_label_520	ANC Register	
reserved_name_for_field_list_labels_521		1 Indicator is filled<br>2 Indicator Not filled<br>3 No indicator available
phc_g_3_1 *(required)*	Date of visit	1 Indicator is filled<br>2 Indicator Not filled<br>3 No indicator available
phc_g_3_2 *(required)*	Woman ID	1 Indicator is filled<br>2 Indicator Not filled<br>3 No indicator available
phc_g_3_3 *(required)*	Name	1 Indicator is filled<br>2 Indicator Not filled<br>3 No indicator available
phc_g_3_4 *(required)*	Age	1 Indicator is filled<br>2 Indicator Not filled<br>3 No indicator available
phc_g_3_5 *(required)*	Address	1 Indicator is filled<br>2 Indicator Not filled<br>3 No indicator available
phc_g_3_16 *(required)*	Contact details	1 Indicator is filled<br>2 Indicator Not filled

Field	Question	Answer
		3 No indicator available
phc_g_3_6 *(required)*	Gestational age (GA) at first registration	1 Indicator is filled<br>2 Indicator Not filled<br>3 No indicator available
phc_g_3_7 *(required)*	BMI in first trimester	1 Indicator is filled<br>2 Indicator Not filled<br>3 No indicator available
phc_g_3_8 *(required)*	Records/columns of all ANC visits (no. of columns) along with provision to track GWG & Hb	1 Indicator is filled<br>2 Indicator Not filled<br>3 No indicator available
phc_g_3_9 *(required)*	Blood Pressure	1 Indicator is filled<br>2 Indicator Not filled<br>3 No indicator available
phc_g_3_10 *(required)*	Blood investigation- CBC at the time of registration	1 Indicator is filled<br>2 Indicator Not filled<br>3 No indicator available
phc_g_3_17 *(required)*	Blood investigation- TSH at the time of registration	1 Indicator is filled<br>2 Indicator Not filled<br>3 No indicator available
phc_g_3_11 *(required)*	OGTT- 1 time	1 Indicator is filled<br>2 Indicator Not filled<br>3 No indicator available
phc_g_3_12 *(required)*	Hb-4 times	1 Indicator is filled<br>2 Indicator Not filled<br>3 No indicator available
phc_g_3_13 *(required)*	Urine routine & microscopy- 4 times	1 Indicator is filled<br>2 Indicator Not filled<br>3 No indicator available
phc_g_3_14 *(required)*	Ultrasound	1 Indicator is filled<br>2 Indicator Not filled<br>3 No indicator available
phc_g_3_15 *(required)*	Td vaccination	1 Indicator is filled<br>2 Indicator Not filled<br>3 No indicator available
PHC > phc_g_group > phc_anc_record_ped<br>*Group relevant when: ${phc_f_13_r} =1*		
generated_table_list_label_539	Pediatric/0-24 Months Infants & Children Register	
reserved_name_for_field_list_labels_540		1 Indicator is filled<br>2 Indicator Not filled<br>3 No indicator available
phc_g_4_1 *(required)*	Date of visit	1 Indicator is filled<br>2 Indicator Not filled<br>3 No indicator available
phc_g_4_2 *(required)*	Child ID	1 Indicator is filled<br>2 Indicator Not filled<br>3 No indicator available
phc_g_4_3 *(required)*	Name	1 Indicator is filled<br>2 Indicator Not filled<br>3 No indicator available
phc_g_4_4 *(required)*	Age	1 Indicator is filled<br>2 Indicator Not filled<br>3 No indicator available
phc_g_4_5 *(required)*	Sex	1 Indicator is filled<br>2 Indicator Not filled<br>3 No indicator available
phc_g_4_6 *(required)*	Mother Name	1 Indicator is filled<br>2 Indicator Not filled<br>3 No indicator available
phc_g_4_7 *(required)*	Father Name	1 Indicator is filled

Field	Question	Answer
		2 Indicator Not filled<br>3 No indicator available
phc_g_4_8 *(required)*	Address	1 Indicator is filled<br>2 Indicator Not filled<br>3 No indicator available
phc_g_4_17 *(required)*	Contact details	1 Indicator is filled<br>2 Indicator Not filled<br>3 No indicator available
phc_g_4_9 *(required)*	Weight	1 Indicator is filled<br>2 Indicator Not filled<br>3 No indicator available
phc_g_4_10 *(required)*	Height	1 Indicator is filled<br>2 Indicator Not filled<br>3 No indicator available
phc_g_4_11 *(required)*	Tracking of IWG	1 Indicator is filled<br>2 Indicator Not filled<br>3 No indicator available
phc_g_4_12 *(required)*	Identification of danger signs	1 Indicator is filled<br>2 Indicator Not filled<br>3 No indicator available
phc_g_4_13 *(required)*	Morbidities Reported	1 Indicator is filled<br>2 Indicator Not filled<br>3 No indicator available
phc_g_4_14 *(required)*	Treatment Provided	1 Indicator is filled<br>2 Indicator Not filled<br>3 No indicator available
phc_g_4_15 *(required)*	Immunization Status	1 Indicator is filled<br>2 Indicator Not filled<br>3 No indicator available
phc_g_4_16 *(required)*	Follow-up visit Date	1 Indicator is filled<br>2 Indicator Not filled<br>3 No indicator available
PHC > phc_g_group > phc_labour_room_record_data<br>*Group relevant when: ${phc_f_14_r} =1*		
generated_table_list_label_558	Labour Room Register	
reserved_name_for_field_list_labels_559		1 Indicator is filled<br>2 Indicator Not filled<br>3 No indicator available
phc_g_5_1 *(required)*	Date of admission	1 Indicator is filled<br>2 Indicator Not filled<br>3 No indicator available
phc_g_5_2 *(required)*	Patient ID	1 Indicator is filled<br>2 Indicator Not filled<br>3 No indicator available
phc_g_5_3 *(required)*	Name	1 Indicator is filled<br>2 Indicator Not filled<br>3 No indicator available
phc_g_5_4 *(required)*	Age	1 Indicator is filled<br>2 Indicator Not filled<br>3 No indicator available
phc_g_5_5 *(required)*	Address	1 Indicator is filled<br>2 Indicator Not filled<br>3 No indicator available
phc_g_5_13 *(required)*	Contact details	1 Indicator is filled<br>2 Indicator Not filled<br>3 No indicator available
phc_g_5_6 *(required)*	Time of admission	1 Indicator is filled<br>2 Indicator Not filled<br>3 No indicator available

Field	Question	Answer
phc_g_5_7 *(required)*	Type of delivery (e.g. normal,cesarean)	1 Indicator is filled<br>2 Indicator Not filled<br>3 No indicator available
phc_g_5_8 *(required)*	Complications during delivery	1 Indicator is filled<br>2 Indicator Not filled<br>3 No indicator available
phc_g_5_9 *(required)*	Birth weight	1 Indicator is filled<br>2 Indicator Not filled<br>3 No indicator available
phc_g_5_10 *(required)*	APGAR score	1 Indicator is filled<br>2 Indicator Not filled<br>3 No indicator available
phc_g_5_11 *(required)*	Time of discharge	1 Indicator is filled<br>2 Indicator Not filled<br>3 No indicator available
phc_g_5_12 *(required)*	Referral information.	1 Indicator is filled<br>2 Indicator Not filled<br>3 No indicator available
PHC > phc_g_group > phc_lab_record<br>*Group relevant when: ${phc_f_15_r} =1*		
generated_table_list_label_573	Laboratory Record	
reserved_name_for_field_list_labels_574		1 Indicator is filled<br>2 Indicator Not filled<br>3 No indicator available
phc_g_6_1 *(required)*	Date	1 Indicator is filled<br>2 Indicator Not filled<br>3 No indicator available
phc_g_6_2 *(required)*	Sample ID	1 Indicator is filled<br>2 Indicator Not filled<br>3 No indicator available
phc_g_6_3 *(required)*	Patient ID	1 Indicator is filled<br>2 Indicator Not filled<br>3 No indicator available
phc_g_6_4 *(required)*	Name	1 Indicator is filled<br>2 Indicator Not filled<br>3 No indicator available
phc_g_6_5 *(required)*	Type of investigation	1 Indicator is filled<br>2 Indicator Not filled<br>3 No indicator available
phc_g_6_6 *(required)*	Investigation results	1 Indicator is filled<br>2 Indicator Not filled<br>3 No indicator available
phc_g_6_7 *(required)*	Remarks	1 Indicator is filled<br>2 Indicator Not filled<br>3 No indicator available
phc_g_6_8 *(required)*	Date of report	1 Indicator is filled<br>2 Indicator Not filled<br>3 No indicator available
PHC > phc_g_group > phc_birth_record<br>*Group relevant when: ${phc_f_16_r} =1*		
generated_table_list_label_583	Birth/Death Register	
reserved_name_for_field_list_labels_584		1 Indicator is filled<br>2 Indicator Not filled<br>3 No indicator available
phc_g_7_1 *(required)*	Date of event	1 Indicator is filled<br>2 Indicator Not filled<br>3 No indicator available
phc_g_7_2 *(required)*	Child ID	1 Indicator is filled<br>2 Indicator Not filled<br>3 No indicator available

Field	Question	Answer
phc_g_7_3 *(required)*	Name of child (if applicable)	1 Indicator is filled<br>2 Indicator Not filled<br>3 No indicator available
phc_g_7_4 *(required)*	Mother's Name	1 Indicator is filled<br>2 Indicator Not filled<br>3 No indicator available
phc_g_7_5 *(required)*	Father's Name	1 Indicator is filled<br>2 Indicator Not filled<br>3 No indicator available
phc_g_7_6 *(required)*	Date of birth/death	1 Indicator is filled<br>2 Indicator Not filled<br>3 No indicator available
phc_g_7_7 *(required)*	Place of birth/death	1 Indicator is filled<br>2 Indicator Not filled<br>3 No indicator available
phc_g_7_8 *(required)*	Cause of death	1 Indicator is filled<br>2 Indicator Not filled<br>3 No indicator available
phc_g_7_9 *(required)*	Attendant details (e.g., ASHA, AWW)	1 Indicator is filled<br>2 Indicator Not filled<br>3 No indicator available
phc_g_7_10 *(required)*	Remarks	1 Indicator is filled<br>2 Indicator Not filled<br>3 No indicator available
PHC > phc_g_group > phc_referral_record<br>*Group relevant when: ${phc_f_17_r} =1*		
generated_table_list_label_595	Referral Register	
reserved_name_for_field_list_labels_596		1 Indicator is filled<br>2 Indicator Not filled<br>3 No indicator available
phc_g_8_1 *(required)*	Date of referral	1 Indicator is filled<br>2 Indicator Not filled<br>3 No indicator available
phc_g_8_2 *(required)*	Patient ID	1 Indicator is filled<br>2 Indicator Not filled<br>3 No indicator available
phc_g_8_3 *(required)*	Name	1 Indicator is filled<br>2 Indicator Not filled<br>3 No indicator available
phc_g_8_4 *(required)*	Address	1 Indicator is filled<br>2 Indicator Not filled<br>3 No indicator available
phc_g_8_5 *(required)*	Reason for referral	1 Indicator is filled<br>2 Indicator Not filled<br>3 No indicator available
phc_g_8_6 *(required)*	Referring facility	1 Indicator is filled<br>2 Indicator Not filled<br>3 No indicator available
phc_g_8_7 *(required)*	Receiving facility	1 Indicator is filled<br>2 Indicator Not filled<br>3 No indicator available
phc_g_8_8 *(required)*	Date of receiving feedback	1 Indicator is filled<br>2 Indicator Not filled<br>3 No indicator available
phc_g_8_9 *(required)*	Follow-up visit date	1 Indicator is filled<br>2 Indicator Not filled<br>3 No indicator available
PHC > phc_i_group		
phc_i_1_1 *(required)*	Iron, Vitamin, and Nutritional Supplements<br>*Response constrained to: not(selected(${phc_i_1_1} , '9999') and count-selected(${phc_i_1_1}) > 1)*	1 Tab. Folic Acid (5 mg/Tab)

Field	Question	Answer
		2 Tab. Iron Folic Acid (IFA) (60 mg elemental Iron + 500 mcg Folic Acid, sugar coated)<br>3 Syp. Iron Folic Acid (IFA) (20 mg of elemental iron and 100 mcg of folic acid)<br>4 Inj. Iron Sucrose (50 mg/2.5 ml)<br>5 Tab. Calcium & Vitamin D (Calcium (500 mg) and Vit-D (250 IU))<br>6 Vitamin D Oral Solution (400 IU (10 mcg))<br>7 Multi-micronutrients (With higher dose of Vitamin B12)<br>8 WINGS MMN1<br>9 WINGS MMN2<br>10 Ferrous salt 100 mg<br>11 Cholecalciferol Tab<br>12 Pyridoxine tab<br>99 Other Specify<br>9999 None
PHC > phc_i_group > Drug Status (1)		(Repeated group)
PHC > phc_i_group > Drug Status (1) > [phc_i_1_cal1]<br>*Group relevant when: ${phc_i_1_1} !=9999*		
phc_i_1_t *(required)*	Target beneficiary	1 Preconception women (18-35 Years)<br>2 Pregnant women<br>3 Postnatal/ lactating women<br>4 0-6 Months Infants<br>5 6-24 Months Infants & Children
phc_i_1_2 *(required)*	Projected Monthly Requirements	
phc_i_1_3 *(required)*	Current Numbers in Stock	
phc_i_1_4 *(required)*	Stock Out in the last 3 month	1 Yes<br>2 No
phc_i_2_1 *(required)*	Antibiotics<br>*Response constrained to: not(selected(${phc_i_2_1} , '9999') and count-selected(${phc_i_2_1}) > 1)*	1 Inj. Ampicillin Sodium (500 mg or 1 gm/Vial)<br>2 Cap. Amoxicillin (500 mg/Cap)<br>3 Tab. Augmentin (625 mg/Tab)<br>4 Tab. Cefixime (200/400 mg Tab)<br>5 Inj. Gentamicin Sulphate (40 mg/ml)<br>6 Inj. Cefotaxime + Sulbactam (Cefotaxime 1 gm + Sulbactam 0.5 gm/Vial)<br>7 Tab. Cefadroxil (500 mg/Tab)<br>8 Tab. Metronidazole (Coated) (400 mg/Tab)<br>9 Tab. Nitrofurantoin (100 mg/Tab)<br>10 Azithromycin oral liquid (200mg)<br>11 Amoxicillin powder for suspension (125mg)

Field	Question	Answer
		12 Tab.Azithromycin (500mg)<br>13 Tab.Azithromycin (250mg)<br>14 Tab.Amoxicillin (250mg)<br>99 Other Specify<br>9999 None
PHC > phc_i_group > [phc_i_2_cal1] (1)		(Repeated group)
PHC > phc_i_group > [phc_i_2_cal1] (1) > phc_antibiotic_group<br>*Group relevant when: ${phc_i_2_1} !=9999*		
phc_i_2_t *(required)*	Target beneficiary	1 Preconception women (18-35 Years)<br>2 Pregnant women<br>3 Postnatal/ lactating women<br>4 0-6 Months Infants<br>5 6-24 Months Infants & Children
phc_i_2_2 *(required)*	Projected Monthly Requirements	
phc_i_2_3 *(required)*	Current Numbers in Stock	
phc_i_2_4 *(required)*	Stock Out in the last 3 month	1 Yes<br>2 No
phc_i_3_1 *(required)*	Antihypertensives and Seizure Management<br>*Response constrained to: not(selected(${phc_i_3_1} , '9999') and count-selected(${phc_i_3_1}) > 1)*	1 Tab. Methyldopa (250 mg/Tab)<br>2 Cap. Nifedipine (Soft gelatin) (10 mg/Cap)<br>3 Tab. Nifedipine SR (10 mg/Tab)<br>4 Tab. Labetalol (100 mg/Tab)<br>5 Inj. Labetalol (20 mg/4 ml Amp)<br>6 Inj. Magnesium Sulphate (500 mg/ml)<br>7 Tab. Phenytoin Sodium (100 mg/Tab)<br>8 Inj. Lorazepam (1 mg/ml)<br>9 Inj. Diazepam (5 mg/ml)<br>10 Amlodipine (5mg)<br>11 Telmisartan (40mg)<br>99 Other Specify<br>9999 None
PHC > phc_i_group > [phc_i_3_cal1] (1)		(Repeated group)
PHC > phc_i_group > [phc_i_3_cal1] (1) > phc_antihypertensives_group<br>*Group relevant when: ${phc_i_3_1} !=9999*		
phc_i_3_t *(required)*	Target beneficiary	1 Preconception women (18-35 Years)<br>2 Pregnant women<br>3 Postnatal/ lactating women<br>4 0-6 Months Infants<br>5 6-24 Months Infants & Children
phc_i_3_2 *(required)*	Projected Monthly Requirements	
phc_i_3_3 *(required)*	Current Numbers in Stock	
phc_i_3_4 *(required)*	Stock Out in the last 3 month	1 Yes<br>2 No
phc_i_4_1	Analgesics/Antipyretic and Antiemetic<br>*Response constrained to: not(selected(${phc_i_4_1} , '9999') and count-selected(${phc_i_4_1}) > 1)*	1 Tab. Paracetamol (500 mg/Tab)<br>2 Tab. Ibuprofen (400 mg/Tab)<br>3 Tab. Domperidone (10 mg/Tab)<br>4 Syrup. Paracetamol (250ml)

Field	Question	Answer
		5 Syrup. Paracetamol (150ml)<br>6 Syrup.Ibuprofen oral liquid (100mg)<br>7 Tab. Diclofenac 50mg<br>8 Aspirin tab 75mg<br>99 Other Specify<br>9999 None
PHC > phc_i_group > [phc_i_4_cal1] (1)		(Repeated group)
PHC > phc_i_group > [phc_i_4_cal1] (1) > phc_analgesics_antipyretics_group<br>*Group relevant when: ${phc_i_4_1} !=9999*		
phc_i_4_t *(required)*	Target beneficiary	1 Preconception women (18-35 Years)<br>2 Pregnant women<br>3 Postnatal/ lactating women<br>4 0-6 Months Infants<br>5 6-24 Months Infants & Children
phc_i_4_2 *(required)*	Projected Monthly Requirements	
phc_i_4_3 *(required)*	Current Numbers in Stock	
phc_i_4_4 *(required)*	Stock Out in the last 3 month	1 Yes<br>2 No
phc_i_5_1 *(required)*	Anthelmintics<br>*Response constrained to: not(selected(${phc_i_5_1} , '9999') and count-selected(${phc_i_5_1}) > 1)*	1 Tab. Albendazole (Chewable) 400 mg/Tab<br>2 Albendazole Oral Solution 200 mg/5 ml<br>3 Oral Antimalarial<br>99 Other Specify<br>9999 None
PHC > phc_i_group > [phc_i_5_cal1] (1)		(Repeated group)
PHC > phc_i_group > [phc_i_5_cal1] (1) > phc_anthelmintics_group<br>*Group relevant when: ${phc_i_5_1} !=9999*		
phc_i_5_t *(required)*	Target beneficiary	1 Preconception women (18-35 Years)<br>2 Pregnant women<br>3 Postnatal/ lactating women<br>4 0-6 Months Infants<br>5 6-24 Months Infants & Children
phc_i_5_2 *(required)*	Projected Monthly Requirements	
phc_i_5_3 *(required)*	Current Numbers in Stock	
phc_i_5_4 *(required)*	Stock Out in the last 3 month	1 Yes<br>2 No
phc_i_6_1 *(required)*	Diabetic & Thyroid Medications<br>*Response constrained to: not(selected(${phc_i_6_1} , '9999') and count-selected(${phc_i_6_1}) > 1)*	1 Inj. Human Soluble Insulin (40 IU/ml)<br>2 Tab. Metformin HCl (Coated) (500 mg/Tab)<br>3 Tab. Eltroxin / Thyroxine (100 mcg)<br>99 Other Specify<br>9999 None
PHC > phc_i_group > [phc_i_6_cal1] (1)		(Repeated group)
PHC > phc_i_group > [phc_i_6_cal1] (1) > phc_diabetic_thyroid_group<br>*Group relevant when: ${phc_i_6_1} !=9999*		
phc_i_6_t *(required)*	Target beneficiary	1 Preconception women (18-35 Years)<br>2 Pregnant women<br>3 Postnatal/ lactating women<br>4 0-6 Months Infants<br>5 6-24 Months Infants & Children

Field	Question	Answer
phc_i_6_2 *(required)*	Projected Monthly Requirements	
phc_i_6_3 *(required)*	Current Numbers in Stock	
phc_i_6_4 *(required)*	Stock Out in the last 3 month	1 Yes<br>2 No
phc_i_7_1 *(required)*	Other Miscellaneous<br>*Response constrained to: not(selected(${phc_i_7_1} , '9999') and count-selected(${phc_i_7_1}) > 1)*	1 Inj. Td (0.5 ml/Amp)<br>2 Tab. Digoxin (0.25 mg/Tab)<br>3 Tab. Frusemide (40 mg/Tab)<br>4 Inj. Frusemide (10 mg/1 ml)<br>5 Inj. Betamethasone Sod. Phosphate (4 mg/ml)<br>6 Inj. Human Anti-D Immunoglobulin (300 mcg/1.5 ml)<br>7 IV Fluids<br>8 ORS Sachet<br>9 Zinc Tablet<br>10 Povidone Iodine Ointment (5% w/v)<br>11 Sanitary Napkins<br>12 Vaccines (As per National Programme)<br>99 Other Medicine (Specify)<br>9999 None
PHC > phc_i_group > [phc_i_7_cal1] (1)		(Repeated group)
PHC > phc_i_group > [phc_i_7_cal1] (1) > phc_other_miscellaneous_group<br>*Group relevant when: ${phc_i_7_1} !=9999*		
phc_i_7_t *(required)*	Target beneficiary	1 Preconception women (18-35 Years)<br>2 Pregnant women<br>3 Postnatal/ lactating women<br>4 0-6 Months Infants<br>5 6-24 Months Infants & Children
phc_i_7_2 *(required)*	Projected Monthly Requirements	
phc_i_7_3 *(required)*	Current Numbers in Stock	
phc_i_7_4 *(required)*	Stock Out in the last 3 month	1 Yes<br>2 No
PHC > phc_j_group		
PHC > phc_j_group > Facility's Beneficiaries (Patient) Load		
phc_j_note	Please mention the Numbers (Last 12 Month Data) against each(Please enter 999 if data not available)	
phc_j_1 *(required)*	Eligible couples	
phc_j_2 *(required)*	Preconception women (18-35 years)	
phc_j_3 *(required)*	Pregnant women	
phc_j_4 *(required)*	Postnatal women	
phc_j_5 *(required)*	0 to 6 months infants	
phc_j_6 *(required)*	6 to 12 months infants	
phc_j_7 *(required)*	12 to 24 months children	
phc_j_8 *(required)*	Preconception women (18-35 years) screened for medical conditions	
phc_j_9 *(required)*	Preconception women (18-35 years) screened for depressive symptoms-	
PHC > phc_j_group > Preconception women (18-35 years) screened for medical conditions and depressive symptoms-<br>*Group relevant when: ${phc_j_8} > 0*		
phc_j_8_note	Please mention the Number of Preconception women (18-35 years) screened (Last 12 Month Data) against each	
phc_j_8_1 *(required)*	RTI/STI	
phc_j_8_2 *(required)*	TB	
phc_j_8_3 *(required)*	Epilepsy	
phc_j_8_4 *(required)*	Syphilis	
phc_j_8_5 *(required)*	Thyroid status	
phc_j_8_6 *(required)*	Blood pressure	

Field	Question	Answer
phc_j_8_7 *(required)*	Blood sugar	
phc_j_8_8 *(required)*	Height measured	
phc_j_8_9 *(required)*	Weight measured	
phc_j_8_10 *(required)*	Hemoglobin tested	
phc_j_8_11 *(required)*	PHQ 2	
PHC > phc_j_group > Preconception women (aged 18-35 years) identified with medical conditions & depressive symptoms following screening-<br>*Group relevant when: ${phc_j_9} > 0*		
phc_j_9_note	Please mention the Number of Preconception women (aged 18-35 years) identified with medical conditions & depressive symptoms following screening (Last 12 Month Data) against each	
phc_j_9_1 *(required)*	RTI/STI	
phc_j_9_2 *(required)*	TB	
phc_j_9_3 *(required)*	Epilepsy	
phc_j_9_4 *(required)*	Syphilis	
phc_j_9_5 *(required)*	Hypothyroidism (TSH > 5.5 IU/mL)	
phc_j_9_6 *(required)*	Hyperthyroidism (TSH <0.4 IU/mL)	
phc_j_9_7 *(required)*	Hypertension	
phc_j_9_7_1 *(required)*	Hypotension	
phc_j_9_8 *(required)*	Prediabetes (HbA1c 5.7% to 6.4%)	
phc_j_9_9 *(required)*	Diabetes mellitus (HbA1c ≥6.5%)	
phc_j_9_10 *(required)*	BMI<16 kg/m2	
phc_j_9_11 *(required)*	BMI 16-18.49 kg/m2	
phc_j_9_12 *(required)*	BMI 18.5- <21 kg/m2	
phc_j_9_13 *(required)*	Severe anemia (Hb <8 g/dL)	
phc_j_9_14 *(required)*	Moderate anemia (Hb 8 to 11.99 g/dL)	
phc_j_9_15 *(required)*	Depressive symptoms	
PHC > phc_j_group > Preconception women (aged 18-35 years) managed<br>*Group relevant when: ${phc_j_8} > 0*		
phc_j_10_note	Please mention the Number of Preconception women (aged 18-35 years) managed for (Last 12 Month Data) the following conditions	
phc_j_10_1 *(required)*	RTI/STI	
phc_j_10_2 *(required)*	TB	
phc_j_10_3 *(required)*	Epilepsy	
phc_j_10_4 *(required)*	Syphilis	
phc_j_10_5 *(required)*	Hypothyroidism (TSH > 5.5 IU/mL)	
phc_j_10_6 *(required)*	Hyperthyroidism (TSH <0.4 IU/mL)	
phc_j_10_7 *(required)*	Hypertension	
phc_j_10_7_1 *(required)*	Hypotension	
phc_j_10_8 *(required)*	Prediabetes (HbA1c 5.7% to 6.4%)	
phc_j_10_9 *(required)*	Diabetes mellitus (HbA1c ≥6.5%)	
phc_j_10_10 *(required)*	BMI<16 kg/m2	
phc_j_10_11 *(required)*	BMI 16-18.49 kg/m2	
phc_j_10_12 *(required)*	BMI 18.5- <21 kg/m2	
phc_j_10_13 *(required)*	Severe anemia (Hb <8 g/dL)	
phc_j_10_14 *(required)*	Moderate anemia (Hb 8 to 11.99 g/dL)	
phc_j_10_15 *(required)*	Depressive symptoms	
phc_j_10_16 *(required)*	No. of preconception women who were given IFA as per recommendation	
phc_j_10_17 *(required)*	No. of preconception women who counselled for Nutrition	
phc_j_10_18 *(required)*	No. of preconception women who counselled for WASH intervention	
PHC > phc_j_group > Pregnant women screened/tested for -<br>*Group relevant when: ${phc_j_3} > 0*		
phc_j_11 *(required)*	Number of Pregnant Women Registered for ANC in the last 12 months	
phc_j_11_note	Please mention the Number of Pregnant women screened/tested for (Last 12 Month Data)	
phc_j_11_1 *(required)*	Blood grouping	
phc_j_11_2 *(required)*	Complete Blood Count (CBC) at the time of registration	
phc_j_11_3 *(required)*	HIV	
phc_j_11_4 *(required)*	Syphilis	
phc_j_11_5 *(required)*	Urine routine & microscopy	
phc_j_11_6 *(required)*	Thyroid status / TSH	
phc_j_11_7 *(required)*	Gestational Diabetes Mellitus (GDM)	
phc_j_11_7_1	Oral Glucose Tolerance Test (OGTT)	
phc_j_11_8 *(required)*	Blood pressure	

Field	Question	Answer
phc_j_11_9 *(required)*	Height measured in First Trimester	
phc_j_11_10 *(required)*	Weight measured in First Trimester	
phc_j_11_11 *(required)*	Weight measured in all trimesters	
phc_j_11_12 *(required)*	Patients Health Questionnaire (PHQ-2) in each trimester	
phc_j_11_13 *(required)*	Tested for Hemoglobin (Hb) 4 time during pregnancy	
PHC > phc_j_group > Pregnant women identified with medical conditions & depressive symptoms following screening- *Group relevant when: ${phc_j_3} > 0*		
phc_j_12_note	Please mention the Number of Pregnant women identified with medical conditions & depressive symptoms following screening (Last 12 Month Data)	
phc_j_12_1 *(required)*	HIV	
phc_j_12_2 *(required)*	Syphilis	
phc_j_12_3 *(required)*	Hypothyroidism	
phc_j_12_4 *(required)*	Hyperthyroidism	
phc_j_12_5 *(required)*	Hypertension	
phc_j_12_5_1 *(required)*	Hypotension	
phc_j_12_6 *(required)*	Diabetes mellitus	
phc_j_12_7 *(required)*	BMI < 18.5 kg/m2	
phc_j_12_8 *(required)*	BMI < 25 kg/m2	
phc_j_12_9 *(required)*	Pregnant women with inadequate gestational weight gain	
phc_j_12_10 *(required)*	Severe anemia (Hb <8 g/dL)	
phc_j_12_11 *(required)*	Moderate anemia (Hb 8 to 11.99 g/dL)	
phc_j_12_12 *(required)*	Depressive symptoms	
PHC > phc_j_group > Pregnant women managed for treatment *Group relevant when: ${phc_j_3} > 0*		
phc_j_14_note	Please mention the Number of Pregnant women managed for following conditions(Last 12 Month Data)	
phc_j_14_1 *(required)*	HIV	
phc_j_14_2 *(required)*	Syphilis	
phc_j_14_3 *(required)*	Hypothyroidism	
phc_j_14_4 *(required)*	Hyperthyroidism	
phc_j_14_5 *(required)*	Hypertension	
phc_j_14_5_1 *(required)*	Hypotension	
phc_j_14_6 *(required)*	Diabetes mellitus	
phc_j_14_7 *(required)*	Inadequate gestational weight gain (IGWG)	
phc_j_14_8 *(required)*	Severe anemia (Hb <8 g/dL)	
phc_j_14_9 *(required)*	Moderate anemia (Hb 8 to 11.99 g/dL)	
phc_j_14_10 *(required)*	Depressive symptoms	
phc_j_14_11 *(required)*	Other medical conditions	
phc_j_14_12 *(required)*	No. of pregnant women who were given IFA as per recommendation	
phc_j_14_13 *(required)*	No. of pregnant women who counselled for Nutrition	
phc_j_14_14 *(required)*	No. of pregnant women who counselled for WASH intervention	
PHC > phc_j_group > phc_j_1_group *Group relevant when: ${phc_j_3} > 0*		
phc_j_15 *(required)*	Pregnant women referred during ANC	
phc_j_16 *(required)*	Pregnant women with atleast 4-ANC check-ups	
phc_j_17 *(required)*	Pregnant women with atleast 8-ANC check-ups	
phc_j_18 *(required)*	Pregnant women given corticosteroids for preterm labor	
phc_j_19 *(required)*	Registered antenatal mothers who came for delivery	
phc_j_20 *(required)*	Institutional deliveries	
phc_j_21 *(required)*	Home deliveries	
phc_j_22 *(required)*	Maternal deaths	
phc_j_23 *(required)*	Live births	
phc_j_24 *(required)*	Babies born with Very low birth weight (VLBW) < 1,500 grams	
phc_j_25 *(required)*	Babies born with low birth weight (LBW) < 2,500 grams	
phc_j_26 *(required)*	Preterm babies born (<37 weeks)	
phc_j_27 *(required)*	Early neonatal deaths (within 24 hours of birth)	
PHC > phc_j_group > Other high-risk pregnancies identified during ANC- *Group relevant when: ${phc_j_3} > 0*		
phc_j_13_note	Please mention the Number of Other high-risk pregnancies identified during ANC- (Last 12 Month Data)	
phc_j_13_1 *(required)*	Pregnant women with multiple pregnancy	
phc_j_13_2 *(required)*	Pregnant women with previous bad obstetric history (BOH)*	
phc_j_13_3 *(required)*	Pregnant women with other infections (Hepatitis, Malaria, etc.)	

Field	Question	Answer
phc_j_13_4 *(required)*	Pregnant women with other medical conditions (Asthma, COPD, heart disease, etc.)	
PHC > phc_j_group > Postnatal Women (0-6 Months) & 0-24 Months Infants & Child<br>*Group relevant when: ${phc_j_4} > 0*		
phc_j_28_note	Please mention the Number of Postnatal Women (0-6 Months) & 0-24 Months Infants & Child (Last 12 Month Data)	
phc_j_28_1 *(required)*	Newborns screened for birth defects (as per RBSK)	
phc_j_28_2 *(required)*	Women received postpartum checkup between 48 hours and 14 days after institutional delivery (Facility/Community)	
phc_j_28_3 *(required)*	HBNC visits performed as per guidelines	
phc_j_28_4 *(required)*	Postnatal mothers consumed nutritional supplements as per recommendation	
phc_j_28_5 *(required)*	Postnatal mothers screened for depressive symptoms	
phc_j_28_6 *(required)*	Postnatal mothers who received appropriate psychosocial support	
phc_j_28_7 *(required)*	Postnatal mothers who counselled for WASH intervention	
phc_j_28_8 *(required)*	Postnatal mothers who were given IFA as per recommendation	
phc_j_28_9 *(required)*	Postnatal mothers who counselled for Nutrition	
phc_j_28_10 *(required)*	0-6 months Infants received kangaroo mother care (KMC) as applicable (Facility/Home)	
phc_j_28_11 *(required)*	0-6 months Infants exclusively breastfed (EBF)	
phc_j_28_12 *(required)*	0-6 months Infants (VLBW and LBW) who consumed Iron, as per recommendation	
phc_j_28_13 *(required)*	0-6 months Infants who consumed Vitamin D 400 IU daily	
phc_j_28_14 *(required)*	0-6 months Infants (Pre-term) who consumed calcium and vitamin D, as per recommendation	
phc_j_28_15 *(required)*	6-24 months infants & children who consumed IFA, as per recommendation	
phc_j_28_16 *(required)*	Infants 6-12 months with inadequate weight gain	
phc_j_28_17 *(required)*	Children 12-24 months with inadequate weight gain	
phc_j_28_18 *(required)*	Children (0-24 months) with adequate weight gain from birth till 2 years of age	
phc_j_28_19 *(required)*	Family/Parents having 0-24 months infants & child counselled for WASH interventions	
phc_j_28_20 *(required)*	Children (12-23 months) fully immunized	
phc_j_28_21	Observational Remarks	
phc_j_28_22	Respondent Remarks	
PHC > photo_group		
phc_j_28_photo1	Document1	
phc_j_28_photo2	Document2	
phc_j_28_photo3	Document3	
phc_j_28_photo4	Document4	
phc_j_28_photo5	Document5	
phc_j_28_photo6	Document6	
phc_j_28_photo7	Document7	
phc_j_28_photo8	Document8	
document	Please upload pdf document	

CHC Assessment form

<table>
<tr><th>Field</th><th>Question</th><th>Answer</th></tr>
<tr><td>worker (required)</td><td>Worker Name</td><td>151 Abuhamza
152 Anmol Saini
153 Anshika Sahota
154 Ekta
155 Jyoti Devi
156 Kritika Thakur
157 Mehak Thakur
158 Poonam Devi
159 Riya Puri
160 Shivanshi
161 Varsha Kumari
162 Anchal Walia
163 Harshali
164 Kritika Puri</td></tr>
<tr><td>blocks (required)</td><td>Block Names</td><td>block_1 Amb
block_2 Basdehra
block_3 Gagret
block_4 Haroli
block_5 Thanakalan</td></tr>
<tr><td>civil_hospital (required)</td><td>CHC</td><td>ch_3 CHC Dussara
ch_4 CHC Basdehra
ch_5 CHC Santoshgarh
ch_7 CHC Daulatpur Chowk
ch_9 CHC Beeton
ch_10 CHC Bhadsali
ch_11 CHC Dulehar
ch_12 CHC Kungrath
ch_14 CHC Thanakalan
ch_999_1 None
ch_999_2 None
ch_999_3 None
ch_999_4 None
ch_999_5 None</td></tr>
<tr><td colspan="3">CHC</td></tr>
<tr><td colspan="3">CHC > CHC Identification</td></tr>
<tr><td>chc_a_1</td><td>Date of Assessment:</td><td></td></tr>
<tr><td>chc_a_6 (required)</td><td>Facility id:</td><td></td></tr>
<tr><td>chc_a_8 (required)</td><td>Facility Address with landmark</td><td></td></tr>
<tr><td>chc_a_10 (required)</td><td>Total Population covered by the facility</td><td></td></tr>
<tr><td>chc_a_11 (required)</td><td>Facility In-charge Name:
Response constrained to: not(regex(.,'^(.*)\d(.*)$'))</td><td></td></tr>
<tr><td>chc_a_12 (required)</td><td>Facility In-charge Designation:</td><td></td></tr>
<tr><td>chc_a_13 (required)</td><td>Facility In-charge Contact No.:
Response constrained to: regex(.,"^[6|7|8|9]\d{9}$")</td><td></td></tr>
<tr><td>chc_a_14</td><td>Facility In-charge Email Id.:</td><td></td></tr>
<tr><td>chc_a_15</td><td>Any Additional Respondent</td><td>1 Yes
2 No</td></tr>
<tr><td colspan="3">CHC > Additional Respondent
Group relevant when: ${chc_a_15} =1</td></tr>
<tr><td>chc_a_15_2 (required)</td><td>Additional Respondent Name
Response constrained to: not(regex(.,'^(.*)\d(.*)$'))</td><td></td></tr>
<tr><td>chc_a_15_3 (required)</td><td>Additional Respondent Designation</td><td></td></tr>
<tr><td colspan="3">CHC > chc_infra</td></tr>
<tr><td>chc_b_1 (required)</td><td>Located within Main Habitation (*where the majority of the population resides)
If No, Distance in Km</td><td>1 Yes
2 No</td></tr>
<tr><td>chc_b_2 (required)</td><td>Connected to Motorable Road</td><td>1 Yes
2 No</td></tr>
<tr><td>chc_b_3 (required)</td><td>Designated Government CHC Building</td><td>1 Yes
2 No</td></tr>
</table>

Field	Question	Answer
chc_b_4 *(required)*	General Structure of Building	1 Newly Built<br>2 Newly Renovated<br>3 Old Structure<br>4 Falling apart
chc_b_5 *(required)*	Maintenance of Building	1 No maintenance<br>2 Once a year<br>3 Once in 3 years<br>99 Other (specify)
chc_b_6 *(required)*	Is electrical supply available?	1 24/7<br>3 Average duration of electricity supply per day<br>2 No
chc_b_6_or	Is power backup available?	1 Generator<br>2 Inverter<br>3 Solar<br>99 Other (Specify)<br>9999 None
chc_b_7 *(required)*	Main source of water supply?	1 Borewell<br>2 Piped water<br>99 Other with duration
chc_b_8 *(required)*	Toilet Facility	1 Yes<br>2 No
chc_b_9 *(required)*	Littering	1 Yes<br>2 No
CHC > Toilet & Other facilities		
chc_b_8_1 *(required)*	Facility available inside the toilet<br>*Question relevant when: selected(${chc_b_8} , '1')*	1 Cleanliness<br>2 Toilet tap with running water<br>3 Flush working properly<br>4 Water bucket and mug inside the toilet<br>5 Washbasin with running water<br>6 Soap/liquid items handwash/hand rub<br>7 Disable friendly<br>8 Dustbin with yellow line (in female washroom)<br>9999 None
chc_b_8_2 *(required)*	Gender specific toilet facility<br>*Question relevant when: selected(${chc_b_8} , '1')*	1 Both (male & female separate) Available<br>2 Only Male Available<br>3 Only Female Available<br>4 Common Toilet Available<br>5 Availability of a disabled-friendly toilet
chc_b_10 *(required)*	Availability of general waste bins	1 Green<br>2 Blue<br>3 Both<br>99 Other
chc_b_11 *(required)*	Management (collection, segregation, storage, transportation, treatment and disposal) of BMW as per Bio-Medical Waste Management Rules, 2018	1 Yes<br>2 No
chc_b_11_bins *(required)*	Availability of colour coded BMW bins	1 Red<br>2 Yellow<br>3 Blue Puncture proof<br>4 White Puncture proof
CHC > Availability Status of Human Resource at CHC		
chc_c_2 *(required)*	Sanctioned (No.) MO MBBS	
chc_c_3 *(required)*	In position (No.) MO MBBS<br>*Question relevant when: ${chc_c_2} > 0*<br>*Response constrained to: . >= 0 and . <= ${chc_c_2}*	

Field	Question	Answer
CHC > Availability Status of Human Resource at CHC > MO MBBS (1)<br>*Group relevant when: ${chc_c_2} > 0 and ${chc_c_3} > 0*		(Repeated group)
chc_c_4 *(required)*	Status	1 Available<br>2 Long Leave<br>3 Deputed Somewhere Else
CHC > Availability Status of Human Resource at CHC > Training Status Of MO MBBS (1)		(Repeated group)
CHC > Availability Status of Human Resource at CHC > Training Status Of MO MBBS (1) > chc_mo_training		
generated_table_list_label_63	Training Status Of MO MBBS	
reserved_name_for_field_list_labels_64		1 Yes<br>2 No
chc_c_1_1 *(required)*	IUCD Training	1 Yes<br>2 No
chc_c_1_2 *(required)*	Breastfeeding Training	1 Yes<br>2 No
chc_c_1_3 *(required)*	Routine Immunization	1 Yes<br>2 No
chc_c_1_4 *(required)*	Emergency medicine	1 Yes<br>2 No
chc_c_1_5 *(required)*	NSSK	1 Yes<br>2 No
chc_c_1_6 *(required)*	Neonatal care Training	1 Yes<br>2 No
chc_c_1_7 *(required)*	SAANS Training (Under 5 Pneumonia management)	1 Yes<br>2 No
chc_c_1_8 *(required)*	National Health Program Training	1 Yes<br>2 No
chc_c_1_9 *(required)*	Anemia Mukt Bharat	1 Yes<br>2 No
chc_c_1_10 *(required)*	Infection Prevention and Control	1 Yes<br>2 No
chc_c_1_11 *(required)*	Other	1 Yes<br>2 No
CHC > Availability Status of Human Resource at CHC > Training Status Of MO MBBS (1) > chc_mo_training_others<br>*Group relevant when: ${chc_c_1_11} =1*		
chc_c_1_12 *(required)*	Please specify Training (others)	
chc_c_10 *(required)*	Sanctioned (No.) Nursing officer	
chc_c_11 *(required)*	In position (No.) Nursing officer<br>*Question relevant when: ${chc_c_10} > 0*<br>*Response constrained to: . >= 0 and . <= ${chc_c_10}*	
CHC > Availability Status of Human Resource at CHC > Nursing officer (1)<br>*Group relevant when: ${chc_c_10} > 0 and ${chc_c_11} > 0*		(Repeated group)
chc_c_12 *(required)*	Status	1 Available<br>2 Long Leave<br>3 Deputed Somewhere Else
CHC > Availability Status of Human Resource at CHC > Training Status Of Nursing officer (1)		(Repeated group)
CHC > Availability Status of Human Resource at CHC > Training Status Of Nursing officer (1) > chc_nurse_training		
generated_table_list_label_86	Training Status Of Nursing officer	
reserved_name_for_field_list_labels_87		1 Yes<br>2 No
chc_c_9_1 *(required)*	SBA	1 Yes<br>2 No
chc_c_9_2 *(required)*	NSSK	1 Yes<br>2 No
chc_c_9_3 *(required)*	IUCD	1 Yes<br>2 No
chc_c_9_4 *(required)*	Routine Immunization (Birth Dose)	1 Yes<br>2 No
chc_c_9_5 *(required)*	Cold chain management Training	1 Yes<br>2 No

Field	Question	Answer
chc_c_9_6 *(required)*	SAANS Training	1 Yes<br>2 No
chc_c_9_7 *(required)*	F-IMNCI	1 Yes<br>2 No
chc_c_9_8 *(required)*	Other	1 Yes<br>2 No
CHC > Availability Status of Human Resource at CHC > Training Status Of Nursing officer (1) > chc_nurse_training_others<br>*Group relevant when: ${chc_c_9_8} =1*		
chc_c_9_9 *(required)*	Please specify Training (others)	
chc_c_14 *(required)*	Sanctioned (No.) Pharmacist	
chc_c_15 *(required)*	In position (No.) Pharmacist<br>*Question relevant when: ${chc_c_14} > 0*<br>*Response constrained to: . >= 0 and . <= ${chc_c_14}*	
CHC > Availability Status of Human Resource at CHC > Pharmacist (1)<br>*Group relevant when: ${chc_c_14} > 0 and ${chc_c_15} > 0*		(Repeated group)
chc_c_16 *(required)*	Status	1 Available<br>2 Long Leave<br>3 Deputed Somewhere Else
CHC > Availability Status of Human Resource at CHC > Training Status Of Pharmacist (1)		(Repeated group)
CHC > Availability Status of Human Resource at CHC > Training Status Of Pharmacist (1) > chc_pharmacist_training		
generated_table_list_label_106	Training Status Of Pharmacist	
reserved_name_for_field_list_labels_107		1 Yes<br>2 No
chc_c_13_1 *(required)*	DVDMS Training	1 Yes<br>2 No
chc_c_13_2 *(required)*	Stock management	1 Yes<br>2 No
chc_c_13_3 *(required)*	Cold chain management	1 Yes<br>2 No
chc_c_13_4 *(required)*	Tuberculosis (TB) Training	1 Yes<br>2 No
chc_c_13_5 *(required)*	BMW Management	1 Yes<br>2 No
chc_c_13_6 *(required)*	Sanitation Training	1 Yes<br>2 No
chc_c_13_7 *(required)*	E-Sanjeevni	1 Yes<br>2 No
chc_c_13_8 *(required)*	Other	1 Yes<br>2 No
CHC > Availability Status of Human Resource at CHC > Training Status Of Pharmacist (1) > chc_pharmacist_training_others<br>*Group relevant when: ${chc_c_13_8} =1*		
chc_c_13_9 *(required)*	Please specify Training (others)	
chc_c_18 *(required)*	Sanctioned (No.) Storekeeper	
chc_c_19 *(required)*	In position (No.) Storekeeper<br>*Question relevant when: ${chc_c_18} > 0*<br>*Response constrained to: . >= 0 and . <= ${chc_c_18}*	
CHC > Availability Status of Human Resource at CHC > Storekeeper (1)<br>*Group relevant when: ${chc_c_18} > 0 and ${chc_c_19} > 0*		(Repeated group)
chc_c_20 *(required)*	Status	1 Available<br>2 Long Leave<br>3 Deputed Somewhere Else
CHC > Availability Status of Human Resource at CHC > Training Status Of Storekeeper (1)		(Repeated group)
CHC > Availability Status of Human Resource at CHC > Training Status Of Storekeeper (1) > chc_storekeep_training		
generated_table_list_label_126	Training Status Of Storekeeper	
reserved_name_for_field_list_labels_127		1 Yes<br>2 No
chc_c_17_1 *(required)*	DVDMS Training	1 Yes<br>2 No
chc_c_17_2 *(required)*	Others	1 Yes<br>2 No

Field	Question	Answer
CHC > Availability Status of Human Resource at CHC > Training Status Of Storekeeper (1) > chc_storekeep_training_others<br>*Group relevant when: ${chc_c_17_2} =1*		
chc_c_17_3 *(required)*	Please specify Training (others)	
chc_c_22 *(required)*	Sanctioned (No.) Lab Technician	
chc_c_23 *(required)*	In position (No.) Lab Technician<br>*Question relevant when: ${chc_c_22} > 0*<br>*Response constrained to: . >= 0 and . <= ${chc_c_22}*	
CHC > Availability Status of Human Resource at CHC > Lab Technician (1)<br>*Group relevant when: ${chc_c_22} > 0 and ${chc_c_23} > 0*		(Repeated group)
chc_c_24 *(required)*	Status	1 Available<br>2 Long Leave<br>3 Deputed Somewhere Else
CHC > Availability Status of Human Resource at CHC > Training Status Of Lab Technician (1)		(Repeated group)
CHC > Availability Status of Human Resource at CHC > Training Status Of Lab Technician (1) > chc_labtech_training		
generated_table_list_label_140	Training Status Of Lab Technician	
reserved_name_for_field_list_labels_141		1 Yes<br>2 No
chc_c_21_1 *(required)*	NTEP Training	1 Yes<br>2 No
chc_c_21_2 *(required)*	Malaria Training	1 Yes<br>2 No
chc_c_21_3 *(required)*	Use of Rapid Test Kits	1 Yes<br>2 No
chc_c_21_4 *(required)*	Others	1 Yes<br>2 No
CHC > Availability Status of Human Resource at CHC > Training Status Of Lab Technician (1) > chc_labtech_training_others<br>*Group relevant when: ${chc_c_21_4} =1*		
chc_c_21_5 *(required)*	Please specify Training (others)	
chc_c_26 *(required)*	Sanctioned (No.) Health Worker (Female)/ANM	
chc_c_27 *(required)*	In position (No.) Health Worker (Female)/ANM<br>*Question relevant when: ${chc_c_26} > 0*<br>*Response constrained to: . >= 0 and . <= ${chc_c_26}*	
CHC > Availability Status of Human Resource at CHC > Health Worker (Female)/ANM (1)<br>*Group relevant when: ${chc_c_26} > 0 and ${chc_c_27} > 0*		(Repeated group)
chc_c_28 *(required)*	Status	1 Available<br>2 Long Leave<br>3 Deputed Somewhere Else
CHC > Availability Status of Human Resource at CHC > Training Status Of Health worker (Female)/ANM (1)		(Repeated group)
CHC > Availability Status of Human Resource at CHC > Training Status Of Health worker (Female)/ANM (1) > chc_hwf_training		
generated_table_list_label_156	Training Status Of Health worker (Female)/ANM	
reserved_name_for_field_list_labels_157		1 Yes<br>2 No
chc_c_25_1 *(required)*	Routine immunization	1 Yes<br>2 No
chc_c_25_2 *(required)*	SAANS Training (Under 5 Pneumonia management)	1 Yes<br>2 No
chc_c_25_3 *(required)*	HBNC Training	1 Yes<br>2 No
chc_c_25_4 *(required)*	Diarrhoea management Training (IDCF)	1 Yes<br>2 No
chc_c_25_5 *(required)*	MAA program	1 Yes<br>2 No
chc_c_25_6 *(required)*	IUCD	1 Yes<br>2 No
chc_c_25_7 *(required)*	NCD	1 Yes<br>2 No
chc_c_25_8 *(required)*	VHND	1 Yes<br>2 No
chc_c_25_9 *(required)*	VHSNC	1 Yes<br>2 No

Field	Question	Answer
chc_c_25_10 *(required)*	Eat right tool kit	1 Yes<br>2 No
chc_c_25_11 *(required)*	Other	1 Yes<br>2 No
CHC > Availability Status of Human Resource at CHC > Training Status Of Health worker (Female)/ANM (1) > chc_hwf_training_others<br>*Group relevant when: ${chc_c_25_11} =1*		
chc_c_25_12 *(required)*	Please specify Training (others)	
chc_c_30 *(required)*	Sanctioned (No.) Health Worker (Male)/ Health Assistant (Male)	
chc_c_31 *(required)*	In position (No.) Health Worker (Male)/ Health Assistant (Male)<br>*Question relevant when: ${chc_c_30} > 0*<br>*Response constrained to: . >= 0 and . <= ${chc_c_30}*	
CHC > Availability Status of Human Resource at CHC > Health Worker (Male)/ Health Assistant (Male) (1)<br>*Group relevant when: ${chc_c_30} > 0 and ${chc_c_31} > 0*		(Repeated group)
chc_c_32 *(required)*	Status	1 Available<br>2 Long Leave<br>3 Deputed Somewhere Else
CHC > Availability Status of Human Resource at CHC > Training Status Of Health Worker (Male)/Health Assistant (Male) (1)		(Repeated group)
CHC > Availability Status of Human Resource at CHC > Training Status Of Health Worker (Male)/Health Assistant (Male) (1) > chc_hwm_training		
generated_table_list_label_179	Training Status Of Health Worker (Male)/Health Assistant (Male)	
reserved_name_for_field_list_labels_180		1 Yes<br>2 No
chc_c_29_1 *(required)*	TB	1 Yes<br>2 No
chc_c_29_2 *(required)*	Malaria	1 Yes<br>2 No
chc_c_29_3 *(required)*	Routine Immunization	1 Yes<br>2 No
chc_c_29_4 *(required)*	Water testing	1 Yes<br>2 No
chc_c_29_5 *(required)*	Other	1 Yes<br>2 No
CHC > Availability Status of Human Resource at CHC > Training Status Of Health Worker (Male)/Health Assistant (Male) (1) > chc_hwm_training_others<br>*Group relevant when: ${chc_c_29_5} =1*		
chc_c_29_6 *(required)*	Please specify Training (others)	
chc_c_34 *(required)*	Sanctioned (No.) Female health supervisor	
chc_c_35 *(required)*	In position (No.) Female health supervisor<br>*Question relevant when: ${chc_c_34} > 0*<br>*Response constrained to: . >= 0 and . <= ${chc_c_34}*	
CHC > Availability Status of Human Resource at CHC > Health Assistant Female health supervisor (1)<br>*Group relevant when: ${chc_c_34} > 0 and ${chc_c_35} > 0*		(Repeated group)
chc_c_36 *(required)*	Status	1 Available<br>2 Long Leave<br>3 Deputed Somewhere Else
CHC > Availability Status of Human Resource at CHC > Health Assistant Female health supervisor (1)		(Repeated group)
CHC > Availability Status of Human Resource at CHC > Health Assistant Female health supervisor (1) > chc_lhv_training		
generated_table_list_label_196	Health Assistant Female health supervisor	
reserved_name_for_field_list_labels_197		1 Yes<br>2 No
chc_c_33_1 *(required)*	Cold chain management	1 Yes<br>2 No
chc_c_33_2 *(required)*	Others	1 Yes<br>2 No
CHC > Availability Status of Human Resource at CHC > Health Assistant Female health supervisor (1) > chc_lhv_training_others<br>*Group relevant when: ${chc_c_33_2} =1*		
chc_c_33_3 *(required)*	Please specify Training (others)	
chc_c_38 *(required)*	Sanctioned (No.) Health Educator/ Counsellor	
chc_c_39 *(required)*	In position (No.) Health Educator/ Counsellor<br>*Question relevant when: ${chc_c_38} > 0*<br>*Response constrained to: . >= 0 and . <= ${chc_c_38}*	
CHC > Availability Status of Human Resource at CHC > Health Educator/ Counsellor (1)<br>*Group relevant when: ${chc_c_38} > 0 and ${chc_c_39} > 0*		(Repeated group)

Field	Question	Answer
chc_c_40 *(required)*	Status	1 Available<br>2 Long Leave<br>3 Deputed Somewhere Else
CHC > Availability Status of Human Resource at CHC > Health Educator/ Counsellor (1)		(Repeated group)
CHC > Availability Status of Human Resource at CHC > Health Educator/ Counsellor (1) > chc_hec_training		
generated_table_list_label_210	Health Educator/ Counsellor	
reserved_name_for_field_list_labels_211		1 Yes<br>2 No
chc_c_37_1 *(required)*	HBNC	1 Yes<br>2 No
chc_c_37_2 *(required)*	HBYC	1 Yes<br>2 No
chc_c_37_3 *(required)*	VHND	1 Yes<br>2 No
chc_c_37_4 *(required)*	HIV	1 Yes<br>2 No
chc_c_37_5 *(required)*	Routine Immunization	1 Yes<br>2 No
chc_c_37_6 *(required)*	Adolescent Health	1 Yes<br>2 No
chc_c_37_7 *(required)*	Maternal health	1 Yes<br>2 No
chc_c_37_8 *(required)*	Anemia Mukt Bharat	1 Yes<br>2 No
chc_c_37_9 *(required)*	Under 5 Pneumonia Management Program	1 Yes<br>2 No
chc_c_37_10 *(required)*	Intensive Diarrhoea Control Fortnight (IDCF)	1 Yes<br>2 No
chc_c_37_11 *(required)*	MAA program	1 Yes<br>2 No
chc_c_37_12 *(required)*	IEC Planning & Implementation Training	1 Yes<br>2 No
chc_c_37_13 *(required)*	Other	1 Yes<br>2 No
CHC > Availability Status of Human Resource at CHC > Health Educator/ Counsellor (1) > chc_hec_training_others<br>*Group relevant when: ${chc_c_37_13} =1*		
chc_c_37_14 *(required)*	Please specify Training (others)	
chc_c_42 *(required)*	Sanctioned (No.) Data Entry Operator	
chc_c_43 *(required)*	In position (No.) Data Entry Operator<br>*Question relevant when: ${chc_c_42} > 0*<br>*Response constrained to: . >= 0 and . <= ${chc_c_42}*	
CHC > Availability Status of Human Resource at CHC > Data Entry Operator (1)<br>*Group relevant when: ${chc_c_42} > 0 and ${chc_c_43} > 0*		(Repeated group)
chc_c_44 *(required)*	Status	1 Available<br>2 Long Leave<br>3 Deputed Somewhere Else
chc_c_46 *(required)*	Sanctioned (No.) Obstetrician	
chc_c_47 *(required)*	In position (No.) Obstetrician<br>*Response constrained to: . >= 0 and . <= ${chc_c_46}*	
CHC > Availability Status of Human Resource at CHC > Obstetrician (1)		(Repeated group)
chc_c_48 *(required)*	Status	1 Available<br>2 Long Leave<br>3 Deputed Somewhere Else
chc_c_50 *(required)*	Sanctioned (No.) Pediatrician	
chc_c_51 *(required)*	In position (No.) Pediatrician<br>*Response constrained to: . >= 0 and . <= ${chc_c_50}*	
CHC > Availability Status of Human Resource at CHC > Pediatrician (1)		(Repeated group)
chc_c_52 *(required)*	Status	1 Available<br>2 Long Leave<br>3 Deputed Somewhere Else

Field	Question	Answer
chc_c_54 *(required)*	Sanctioned (No.) Anesthetist	
chc_c_55 *(required)*	In position (No.) Anesthetist<br>*Response constrained to: . >= 0 and . <= ${chc_c_54}*	
CHC > Availability Status of Human Resource at CHC > Anesthetist (1)		(Repeated group)
chc_c_56 *(required)*	Status	1 Available<br>2 Long Leave<br>3 Deputed Somewhere Else
chc_c_58 *(required)*	Sanctioned (No.) Sonologist	
chc_c_59 *(required)*	In position (No.) Sonologist<br>*Response constrained to: . >= 0 and . <= ${chc_c_58}*	
CHC > Availability Status of Human Resource at CHC > Sonologist (1)		(Repeated group)
chc_c_60 *(required)*	Status	1 Available<br>2 Long Leave<br>3 Deputed Somewhere Else
chc_c_62 *(required)*	Sanctioned (No.) Psychiatrist	
chc_c_63 *(required)*	In position (No.) Psychiatrist<br>*Response constrained to: . >= 0 and . <= ${chc_c_62}*	
CHC > Availability Status of Human Resource at CHC > Psychiatrist (1)		(Repeated group)
chc_c_64 *(required)*	Status	1 Available<br>2 Long Leave<br>3 Deputed Somewhere Else
chc_c_66 *(required)*	Sanctioned (No.) Psychologist	
chc_c_67 *(required)*	In position (No.) Psychologist<br>*Response constrained to: . >= 0 and . <= ${chc_c_66}*	
CHC > Availability Status of Human Resource at CHC > Psychologist (1)		(Repeated group)
chc_c_68 *(required)*	Status	1 Available<br>2 Long Leave<br>3 Deputed Somewhere Else
chc_c_70 *(required)*	Sanctioned (No.) Lactation counsellor	
chc_c_71 *(required)*	In position (No.) Lactation counsellor<br>*Response constrained to: . >= 0 and . <= ${chc_c_70}*	
CHC > Availability Status of Human Resource at CHC > Lactation counsellor (1)		(Repeated group)
chc_c_69 *(required)*	Status	1 Available<br>2 Long Leave<br>3 Deputed Somewhere Else
chc_c_73 *(required)*	Any other staff?	1 Yes<br>2 No
CHC > Availability Status of Human Resource at CHC > anyother_group<br>*Group relevant when: ${chc_c_73} =1*		
any_other1	Name<br>*Response constrained to: not(regex(.,'^(.*)\d(.*)$'))*	
any_other2	Designation<br>*Response constrained to: not(regex(.,'^(.*)\d(.*)$'))*	
CHC > chc_infra2		
chc_b_12	Quality assurance assessment certificate/service	
reserved_name_for_field_list_labels_277		1 Yes<br>2 No
chc_b_13 *(required)*	Kayakalp	1 Yes<br>2 No
chc_b_14 *(required)*	NQAS	1 Yes<br>2 No
chc_b_16 *(required)*	SUMAN	1 Yes<br>2 No
CHC > chc_infra3		
chc_b_17 *(required)*	Emergency area and Triage services available	1 Yes<br>2 No
chc_b_18 *(required)*	OPD Room services	1 Yes<br>2 No
chc_b_20 *(required)*	Laboratory services	1 Yes<br>2 No

Field	Question	Answer
chc_b_22 *(required)*	ANC Room services	1 Yes<br>2 No
chc_b_24 *(required)*	Labour Room services	1 Yes<br>2 No
CHC > OPD room Infrastructure Status		
chc_b_19 *(required)*	OPD Room services - Available failities<br>*Question relevant when: ${chc_b_18} =1*<br>*Response constrained to: not(selected(${chc_b_19} , '9999') and count-selected(${chc_b_19}) > 1)*	1 Adequate table, chairs, bench and stool<br>2 Examination table<br>3 Footstep<br>4 Washbasin with Running Water Supply<br>5 Waiting area with sitting facility<br>9999 None of the above
CHC > Laboratory Infrastructure Status		
chc_b_21 *(required)*	Laboratory services - Available failities<br>*Question relevant when: ${chc_b_20} =1*<br>*Response constrained to: not(selected(${chc_b_21} , '9999') and count-selected(${chc_b_21}) > 1)*	1 Adequate table, chairs, bench and stool<br>2 Washbasin with Running Water Supply<br>3 Adequate area for sample collection area<br>4 Adequate areas for carrying out diagnostic activities<br>5 Adequate area for keeping equipment and storage of reagents and records<br>9999 None of the above
CHC > ANC Room Infrastructure Status		
chc_b_23 *(required)*	ANC Room services - Available failities<br>*Question relevant when: ${chc_b_22} =1*<br>*Response constrained to: not(selected(${chc_b_23} , '9999') and count-selected(${chc_b_23}) > 1)*	1 Adequate table, chairs, bench and stool<br>2 Examination table<br>5 Footstep<br>3 Washbasin with Running Water Supply<br>4 Waiting area with sitting facility<br>9999 None of the above
CHC > Labour Room Infrastructure Status		
chc_b_25 *(required)*	Labour Room services - Available failities<br>*Question relevant when: ${chc_b_24} =1*<br>*Response constrained to: not(selected(${chc_b_25} , '9999') and count-selected(${chc_b_25}) > 1)*	1 Adequate labour Tables<br>2 Washbasin with Running Water Supply<br>3 Area Newborn care corner<br>4 Elbow tap
chc_b_26 *(required)*	Post natal care area and beds<br>*If Yes, total number of beds available*	1 Yes<br>2 No
chc_b_27 *(required)*	Immunization Room	1 Yes<br>2 No
chc_b_28 *(required)*	Gynae/Obstetrics OPD	1 Yes<br>2 No
CHC > Gynae/Obstetrics OPD		
chc_b_29 *(required)*	Gynae/Obstetrics OPD - Available Facilities<br>*Question relevant when: ${chc_b_28} =1*<br>*Response constrained to: not(selected(${chc_b_29} , '9999') and count-selected(${chc_b_29}) > 1)*	1 Adequate table, chairs, bench and stool<br>2 Examination table<br>3 Footstep<br>4 Washbasin with Running Water Supply<br>5 Waiting area with sitting facility<br>9999 None of the above
chc_b_30 *(required)*	Pediatrics OPD	1 Yes<br>2 No

Field	Question	Answer
CHC > Pediatrics OPD<br>*Group relevant when: ${chc_b_30} =1*		
chc_b_31 *(required)*	Pediatrics OPD - Available Facilities<br>*Response constrained to: not(selected(${chc_b_29} , '9999') and count-selected(${chc_b_29}) > 1)*	1 Adequate table, chairs, bench and stool<br>2 Examination table<br>3 Footstep<br>4 Washbasin with Running Water Supply<br>5 Waiting area with sitting facility<br>9999 None of the above
CHC > Availability Status of Equipment at CHC		
chc_d_1 *(required)*	24x7 Emergency Services with Triage, Resuscitation & Stabilization	1 Yes<br>2 No
CHC > Availability Status of Equipment at CHC > chc_triage<br>*Group relevant when: ${chc_d_1} =1*		
chc_d_2 *(required)*	Emergency Drug Tray<br>*If No, specify missing medicines*	1 Yes<br>2 No
chc_d_3 *(required)*	Oxygen Cylinder<br>*If yes, please specify number of functional equipments*	1 Yes<br>2 No
chc_d_69 *(required)*	O2 concentrator<br>*If yes, please specify number of functional equipments*	1 Yes<br>2 No
chc_d_4 *(required)*	Suction Machine (Electrical)<br>*If yes, please specify number of functional equipments*	1 Yes<br>2 No
chc_d_70 *(required)*	Suction Machine (foot-operated)<br>*If yes, please specify number of functional equipments*	1 Yes<br>2 No
chc_d_5 *(required)*	Resuscitation equipment for adult<br>*If No, specify missing medicines*	1 Yes<br>2 No
chc_d_7 *(required)*	Open care/Radiant Warmer<br>*If yes, please specify number of functional equipments*	1 Yes<br>2 No
chc_d_8 *(required)*	Pulse Oximeter Adult<br>*If yes, please specify number of functional equipments*	1 Yes<br>2 No
chc_d_71 *(required)*	Pulse Oximeter Pediatric<br>*If yes, please specify number of functional equipments*	1 Yes<br>2 No
chc_d_9 *(required)*	Self-Inflating Bag and Mask Adult<br>*If yes, please specify number of functional equipments*	1 Yes<br>2 No
chc_d_72 *(required)*	Self-Inflating Bag and Mask Child<br>*If yes, please specify number of functional equipments*	1 Yes<br>2 No
chc_d_80 *(required)*	Self-Inflating Bag and Mask Neonatal Size(0,1)<br>*If yes, please specify number of functional equipments*	1 Yes<br>2 No
chc_d_10 *(required)*	Laryngoscope and ET Intubation Tubes all Sizes<br>*If yes, please specify number of functional equipments*	1 Yes<br>2 No
chc_d_11 *(required)*	ANC Services	1 Yes<br>2 No
CHC > Availability Status of Equipment at CHC > ANC Services<br>*Group relevant when: ${chc_d_11} =1*		
chc_d_12 *(required)*	Blood Pressure Monitor (Digital)<br>*If yes, please specify number of functional equipments*	1 Yes<br>2 No
chc_d_12_1 *(required)*	Blood Pressure Monitor (Manual)<br>*If yes, please specify number of functional equipments*	1 Yes<br>2 No
chc_d_13 *(required)*	Stethoscope<br>*If yes, please specify number of functional equipments*	1 Yes<br>2 No
chc_d_14 *(required)*	Thermometer (Digital)<br>*If yes, please specify number of functional equipments*	1 Yes<br>2 No
chc_d_73 *(required)*	Clinical Thermometer<br>*If yes, please specify number of functional equipments*	1 Yes<br>2 No
chc_d_15 *(required)*	Adult Weighing Scale (Digital)<br>*If yes, please specify number of functional equipments*	1 Yes<br>2 No
chc_d_15_1 *(required)*	Adult Weighing Scale (Analogue)<br>*If yes, please specify number of functional equipments*	1 Yes

Field	Question	Answer
		2 No
chc_d_16 *(required)*	Stadiometer<br>*If yes, please specify number of functional equipments*	1 Yes<br>2 No
chc_d_17 *(required)*	Measuring Tape<br>*If yes, please specify number of functional equipments*	1 Yes<br>2 No
chc_d_18 *(required)*	Examination Lamp with White Light<br>*If yes, please specify number of functional equipments*	1 Yes<br>2 No
chc_d_19 *(required)*	Fetoscope<br>*If yes, please specify number of functional equipments*	1 Yes<br>2 No
chc_d_74 *(required)*	Fetal Doppler Monitor<br>*If yes, please specify number of functional equipments*	1 Yes<br>2 No
chc_d_20 *(required)*	Cusco's Speculum<br>*If yes, please specify number of functional equipments*	1 Yes<br>2 No
chc_d_75 *(required)*	Sims Speculum<br>*If yes, please specify number of functional equipments*	1 Yes<br>2 No
chc_d_21 *(required)*	Pulse oximeter (adult)<br>*If yes, please specify number of functional equipments*	1 Yes<br>2 No
chc_d_22 *(required)*	Gynae/Obstetrics OPD Service	1 Yes<br>2 No
CHC > Availability Status of Equipment at CHC > Gynae/Obstetrics OPD Services<br>*Group relevant when: ${chc_d_22} =1*		
chc_d_23 *(required)*	Blood Pressure Monitor (Digital)<br>*If yes, please specify number of functional equipments*	1 Yes<br>2 No
chc_d_23_1 *(required)*	Blood Pressure Monitor (Manual)<br>*If yes, please specify number of functional equipments*	1 Yes<br>2 No
chc_d_24 *(required)*	Stethoscope<br>*If yes, please specify number of functional equipments*	1 Yes<br>2 No
chc_d_25 *(required)*	Pediatric OPD Service	1 Yes<br>2 No
CHC > Availability Status of Equipment at CHC > Pediatric OPD Services<br>*Group relevant when: ${chc_d_25} =1*		
chc_d_26 *(required)*	Pediatric Stethoscope<br>*If yes, please specify number of functional equipments*	1 Yes<br>2 No
chc_d_27 *(required)*	Clinical Thermometer<br>*If yes, please specify number of functional equipments*	1 Yes<br>2 No
chc_d_76 *(required)*	Digital Thermometer<br>*If yes, please specify number of functional equipments*	1 Yes<br>2 No
chc_d_77 *(required)*	Infrared Thermometer<br>*If yes, please specify number of functional equipments*	1 Yes<br>2 No
chc_d_28 *(required)*	Infantometer<br>*If yes, please specify number of functional equipments*	1 Yes<br>2 No
chc_d_29 *(required)*	Baby Weighing Scale (Electronic)<br>*If yes, please specify number of functional equipments*	1 Yes<br>2 No
chc_d_29_1 *(required)*	Baby Weighing Scale (Manual)<br>*If yes, please specify number of functional equipments*	1 Yes<br>2 No
chc_d_30 *(required)*	Pulse oximeter (Pediatric)<br>*If yes, please specify number of functional equipments*	1 Yes<br>2 No
chc_d_31 *(required)*	Phototherapy machine<br>*If yes, please specify number of functional equipments*	1 Yes<br>2 No
chc_d_32 *(required)*	Pediatric Resuscitation Kit<br>*If no, please specify missing equipments*	1 Yes<br>2 No
chc_d_33 *(required)*	Breast pump (Manual)<br>*If yes, please specify number of functional equipments*	1 Yes<br>2 No
chc_d_33_1 *(required)*	Breast pump (Electric)<br>*If yes, please specify number of functional equipments*	1 Yes<br>2 No
CHC > Availability Status of Equipment at CHC > Labour Room<br>*Group relevant when: ${chc_d_31} =1*		
chc_d_34 *(required)*	Foetal doppler<br>*If yes, please specify number of functional equipments*	1 Yes<br>2 No

Field	Question	Answer
chc_d_35 *(required)*	Cardiotocography (CTG) machine<br>*If yes, please specify number of functional equipments*	1 Yes<br>2 No
chc_d_36 *(required)*	Laryngoscopes (LED)<br>*If yes, please specify number of functional equipments*	1 Yes<br>2 No
chc_d_38 *(required)*	Suction Machine (Electrical)<br>*If yes, please specify number of functional equipments*	1 Yes<br>2 No
chc_d_78 *(required)*	Suction Machine (Foot-operated)<br>*If yes, please specify number of functional equipments*	1 Yes<br>2 No
chc_d_39 *(required)*	Examination light<br>*If yes, please specify number of functional equipments*	1 Yes<br>2 No
chc_d_40 *(required)*	Open care radiant warmer<br>*If yes, please specify number of functional equipments*	1 Yes<br>2 No
chc_d_41 *(required)*	Vacuum extractors<br>*If yes, please specify number of functional equipments*	1 Yes<br>2 No
chc_d_42 *(required)*	Pulse Oximeter (Adult and Pediatric Probes)<br>*If yes, please specify number of functional equipments*	1 Yes<br>2 No
chc_d_43 *(required)*	Self-Inflating Bag and Mask Neonatal Size(0,1)<br>*If yes, please specify number of functional equipments*	1 Yes<br>2 No
chc_d_44 *(required)*	NORMAL DELIVERY TRAY (equipments)<br>*If yes, please specify number of functional equipments*	1 Yes<br>2 No
chc_d_45 *(required)*	Episiotomy kit<br>*If yes, please specify number of functional equipments*	1 Yes<br>2 No
chc_d_46 *(required)*	Forceps delivery kit<br>*If yes, please specify number of functional equipments*	1 Yes<br>2 No
chc_d_47 *(required)*	Craniotomy kit<br>*If yes, please specify number of functional equipments*	1 Yes<br>2 No
chc_d_48 *(required)*	Crash Cart with emergency medicines<br>*If No, specify missing medicines*	1 Yes<br>2 No
chc_d_49 *(required)*	Feeding tube<br>*If yes, please specify number of functional equipments*	1 Yes<br>2 No
chc_d_50 *(required)*	Suction catheter<br>*If yes, please specify number of functional equipments*	1 Yes<br>2 No
chc_d_52 *(required)*	Cord clamps<br>*If yes, please specify number of functional equipments*	1 Yes<br>2 No
chc_d_53 *(required)*	Indoor Patient Service	1 Yes<br>2 No
CHC > Availability Status of Equipment at CHC > Indoor Patient Services<br>*Group relevant when: ${chc_d_53} =1*		
chc_d_54 *(required)*	Total Beds<br>*If yes, please specify number of functional equipments*	1 Yes<br>2 No
chc_d_55 *(required)*	Thermometer<br>*If yes, please specify number of functional equipments*	1 Yes<br>2 No
chc_d_56 *(required)*	BP Apparatus<br>*If yes, please specify number of functional equipments*	1 Yes<br>2 No
chc_d_57 *(required)*	Glucometer with strips<br>*If yes, please specify number of functional equipments*	1 Yes<br>2 No
chc_d_58 *(required)*	Adult Weighing Scale (Digital)<br>*If yes, please specify number of functional equipments*	1 Yes<br>2 No
chc_d_58_1 *(required)*	Adult Weighing Scale (Analog)<br>*If yes, please specify number of functional equipments*	1 Yes<br>2 No
chc_d_59 *(required)*	Oxygen Cylinder<br>*If yes, please specify number of functional equipments*	1 Yes<br>2 No
chc_d_79 *(required)*	O2 concentrator<br>*If yes, please specify number of functional equipments*	1 Yes<br>2 No
chc_d_60 *(required)*	Immunization Services	1 Yes<br>2 No
CHC > Availability Status of Equipment at CHC > Immunization Services<br>*Group relevant when: ${chc_d_60} =1*		
chc_d_61 *(required)*	ILR with Voltage Stabilizer	1 Yes

Field	Question	Answer
	If yes, please specify number of functional equipments	2 No
chc_d_62 *(required)*	DF Small with Voltage Stabilizer<br>*If yes, please specify number of functional equipments*	1 Yes<br>2 No
chc_d_63 *(required)*	Freeze Tag (2 no per ILR bimonthly)<br>*If yes, please specify number of functional equipments*	1 Yes<br>2 No
chc_d_64 *(required)*	Thermometer<br>*If yes, please specify number of functional equipments*	1 Yes<br>2 No
chc_d_65 *(required)*	Vaccine Carriers with Ice Packs<br>*If yes, please specify number of functional equipments*	1 Yes<br>2 No
chc_d_66 *(required)*	Cold Box<br>*If yes, please specify number of functional equipments*	1 Yes<br>2 No
chc_d_67 *(required)*	Waste Disposable Twin Buckets<br>*If yes, please specify number of functional equipments*	1 Yes<br>2 No
chc_d_68 *(required)*	NBSU Services Available	1 Yes<br>2 No
lab_facility	Is lab service available ?	1 Yes<br>2 No
lab_services	Type of facility<br>*Question relevant when: ${lab_facility} =1*	1 Goverment<br>2 KRSNAA/Empanelled
CHC > Availability Status of Diagnostic tests/services at CHC<br>*Group relevant when: selected(${lab_services} ,1) or selected(${lab_services} ,2)*		
chc_e_1 *(required)*	Haemoglobin - Required Equipment & Reagents are available in the Laboratory?<br>*If Yes, what method was used?*	1 Tested in facility<br>3 Only sample collection<br>2 Not Available
chc_e_2 *(required)*	Platelet Count<br>*If Yes, what method was used?*	1 Tested in facility<br>3 Only sample collection<br>2 Not Available
chc_e_3 *(required)*	Complete Blood Count with ESR<br>*If Yes, what method was used?*	1 Tested in facility<br>3 Only sample collection<br>2 Not Available
chc_e_4 *(required)*	Blood Group And Rh Typing<br>*If Yes, what method was used?*	1 Tested in facility<br>3 Only sample collection<br>2 Not Available
chc_e_5 *(required)*	Blood Cross Matching<br>*If Yes, what method was used?*	1 Tested in facility<br>3 Only sample collection<br>2 Not Available
chc_e_6 *(required)*	Bleeding Time and Clotting Time<br>*If Yes, what method was used?*	1 Tested in facility<br>3 Only sample collection<br>2 Not Available
chc_e_7 *(required)*	MP slide method<br>*If Yes, what other method was used?*	1 Tested in facility<br>3 Only sample collection<br>2 Not Available
chc_e_8 *(required)*	Malaria rapid test<br>*If Yes, what other method was used?*	1 Tested in facility<br>3 Only sample collection<br>2 Not Available
chc_e_9 *(required)*	Urine test for pregnancy(UPT)<br>*If Yes, what other method was used?*	1 Tested in facility<br>3 Only sample collection<br>2 Not Available
chc_e_10 *(required)*	Urine Microscopy<br>*If Yes, what other method was used?*	1 Tested in facility<br>3 Only sample collection<br>2 Not Available
chc_e_11 *(required)*	Urine for microalbumin<br>*If Yes, what other method was used?*	1 Tested in facility<br>3 Only sample collection<br>2 Not Available
chc_e_12 *(required)*	Stool for ova and cyst<br>*If Yes, what method was used?*	1 Tested in facility<br>3 Only sample collection<br>2 Not Available
chc_e_13 *(required)*	Stool for Occult Blood<br>*If Yes, what method was used?*	1 Tested in facility<br>3 Only sample collection

Field	Question	Answer
		2 Not Available
chc_e_14 *(required)*	RPR/VDRL test for syphilis<br>*If Yes, what method was used?*	1 Tested in facility<br>3 Only sample collection<br>2 Not Available
chc_e_15 *(required)*	HIV test (Antibodies 1/2 and HIV 1/2)<br>*If Yes, what method was used?*	1 Tested in facility<br>3 Only sample collection<br>2 Not Available
chc_e_16 *(required)*	Hepatitis B surface antigen test<br>*If Yes, what method was used?*	1 Tested in facility<br>3 Only sample collection<br>2 Not Available
chc_e_17 *(required)*	HCV Antibody Test (Anti HCV)<br>*If No, what method was used?*	1 Tested in facility<br>3 Only sample collection<br>2 Not Available
chc_e_19 *(required)*	Sputum, pus etc. for AFB<br>*If Yes, what method was used?*	1 Tested in facility<br>3 Only sample collection<br>2 Not Available
chc_e_20 *(required)*	Typhoid test (IgM)<br>*If Yes, what method was used?*	1 Tested in facility<br>3 Only sample collection<br>2 Not Available
chc_e_21 *(required)*	Blood sugar -RBS (Random blood sugar)<br>*If Yes, what method was used?*	1 Tested in facility<br>3 Only sample collection<br>2 Not Available
chc_e_22 *(required)*	Oral Glucose Tolerance Test (OGTT)<br>*If Yes, what method was used?*	1 Tested in facility<br>3 Only sample collection<br>2 Not Available
chc_e_23 *(required)*	Wet mount and Gram stain for RTI/STD<br>*If Yes, what method was used?*	1 Tested in facility<br>3 Only sample collection<br>2 Not Available
chc_e_24 *(required)*	S. TSH (including for newborn screening) also include T3,T4 , TSH FOR PW<br>*If Yes, what method was used?*	1 Tested in facility<br>3 Only sample collection<br>2 Not Available
chc_e_25 *(required)*	Urine Dipstick Test<br>*If Yes, what method was used?*	1 Tested in facility<br>3 Only sample collection<br>2 Not Available
chc_e_24_remarks *(required)*	Remarks (if any)	
CHC > chc_iec		
generated_table_list_label_539	Availability Status of IEC Material/algorithms and management charts related to the national/state programs in local script.	
reserved_name_for_field_list_labels_540		1 Program being implemented<br>2 IEC material Available<br>3 IEC material displayed<br>4 Program not being implemented
chc_f_1	Family Planning- Mission Parivar Vikas	1 Program being implemented<br>2 IEC material Available<br>3 IEC material displayed<br>4 Program not being implemented
chc_f_2	Anemia- AMB charts and algorithms	1 Program being implemented<br>2 IEC material Available<br>3 IEC material displayed<br>4 Program not being implemented
chc_f_3	Adolescent health programs	1 Program being implemented<br>2 IEC material Available<br>3 IEC material displayed<br>4 Program not being implemented
chc_f_4	Nutrition- POSHAN Abhiyan	1 Program being implemented<br>2 IEC material Available<br>3 IEC material displayed<br>4 Program not being implemented
chc_f_5	Sanitation and Hygiene: Swachh Bharat Mission Gramin, handwashing	1 Program being implemented<br>2 IEC material Available

Field	Question	Answer	
		3	IEC material displayed
		4	Program not being implemented
chc_f_6	ANC	1	Program being implemented
		2	IEC material Available
		3	IEC material displayed
		4	Program not being implemented
chc_f_7	PMSMA	1	Program being implemented
		2	IEC material Available
		3	IEC material displayed
		4	Program not being implemented
chc_f_8	Management of PPH, Shock	1	Program being implemented
		2	IEC material Available
		3	IEC material displayed
		4	Program not being implemented
chc_f_9	Newborn Resuscitation, NSSK	1	Program being implemented
		2	IEC material Available
		3	IEC material displayed
		4	Program not being implemented
chc_f_10	HBNC	1	Program being implemented
		2	IEC material Available
		3	IEC material displayed
		4	Program not being implemented
chc_f_11	Childhood care- MAA, HBYC	1	Program being implemented
		2	IEC material Available
		3	IEC material displayed
		4	Program not being implemented
chc_f_12	Immunization	1	Program being implemented
		2	IEC material Available
		3	IEC material displayed
		4	Program not being implemented
chc_f_13	Breastfeeding	1	Program being implemented
		2	IEC material Available
		3	IEC material displayed
		4	Program not being implemented
chc_f_14	Diarrhea management- Zinc, ORS	1	Program being implemented
		2	IEC material Available
		3	IEC material displayed
		4	Program not being implemented
chc_f_15	Pneumonia Management	1	Program being implemented
		2	IEC material Available
		3	IEC material displayed
		4	Program not being implemented
chc_f_16	JSY	1	Program being implemented
		2	IEC material Available
		3	IEC material displayed
		4	Program not being implemented
chc_f_17	JSSK	1	Program being implemented
		2	IEC material Available
		3	IEC material displayed
		4	Program not being implemented
chc_f_18	Ambulance (National Ambulance Services)	1	Program being implemented
		2	IEC material Available
		3	IEC material displayed
		4	Program not being implemented
chc_f_19	Telemedicine	1	Program being implemented
		2	IEC material Available
		3	IEC material displayed
		4	Program not being implemented
chc_f_20	Danger Signs (Maternal and Neonatal care)	1	Program being implemented
		2	IEC material Available

Field	Question	Answer
		3 IEC material displayed<br>4 Program not being implemented
CHC > chc_register_group		
chc_f_10_r *(required)*	General OPD Register	1 Yes<br>2 No
chc_f_11_r *(required)*	Pre-conception Women Register	1 Yes<br>2 No
chc_f_12_r *(required)*	ANC Register	1 Yes<br>2 No
chc_f_13_r *(required)*	Pediatric/0-24 Months Infants & Children Register	1 Yes<br>2 No
chc_f_14_r *(required)*	Labour Room Register	1 Yes<br>2 No
chc_f_15_r *(required)*	Laboratory Record	1 Yes<br>2 No
chc_f_16_r *(required)*	Birth Register	1 Yes<br>2 No
chc_f_16_1_r *(required)*	Death Register	1 Yes<br>2 No
chc_f_17_r *(required)*	Referral Register	1 Yes<br>2 No
CHC > chc_g_group		
CHC > chc_g_group > chc_maintenance_record_data<br>*Group relevant when: ${chc_f_10_r} =1*		
generated_table_list_label_573	General OPD Register	
reserved_name_for_field_list_labels_574		1 Indicator is filled<br>2 Indicator Not filled<br>3 No indicator available
chc_g_1_1 *(required)*	Date	1 Indicator is filled<br>2 Indicator Not filled<br>3 No indicator available
chc_g_1_2 *(required)*	Patient ID	1 Indicator is filled<br>2 Indicator Not filled<br>3 No indicator available
chc_g_1_3 *(required)*	Name	1 Indicator is filled<br>2 Indicator Not filled<br>3 No indicator available
chc_g_1_4 *(required)*	Age	1 Indicator is filled<br>2 Indicator Not filled<br>3 No indicator available
chc_g_1_5 *(required)*	Sex	1 Indicator is filled<br>2 Indicator Not filled<br>3 No indicator available
chc_g_1_6 *(required)*	Address	1 Indicator is filled<br>2 Indicator Not filled<br>3 No indicator available
chc_g_1_13 *(required)*	Contact details	1 Indicator is filled<br>2 Indicator Not filled<br>3 No indicator available
chc_g_1_7 *(required)*	Marital status	1 Indicator is filled<br>2 Indicator Not filled<br>3 No indicator available
chc_g_1_8 *(required)*	Condition for which visiting OPD	1 Indicator is filled<br>2 Indicator Not filled<br>3 No indicator available
chc_g_1_9 *(required)*	Treatment provided	1 Indicator is filled<br>2 Indicator Not filled<br>3 No indicator available
chc_g_1_10 *(required)*	Investigations done	1 Indicator is filled

Field	Question	Answer
		2 Indicator Not filled
		3 No indicator available
chc_g_1_11 *(required)*	Follow-up visit date	1 Indicator is filled
		2 Indicator Not filled
		3 No indicator available
chc_g_1_12 *(required)*	Referrals made.	1 Indicator is filled
		2 Indicator Not filled
		3 No indicator available
CHC > chc_g_group > chc_preconception_record_data<br>*Group relevant when: ${chc_f_11_r} =1*		
generated_table_list_label_588	Pre-conception Women Register	
reserved_name_for_field_list_labels_589		1 Indicator is filled
		2 Indicator Not filled
		3 No indicator available
chc_g_2_1 *(required)*	Date of visit	1 Indicator is filled
		2 Indicator Not filled
		3 No indicator available
chc_g_2_2 *(required)*	Woman ID	1 Indicator is filled
		2 Indicator Not filled
		3 No indicator available
chc_g_2_3 *(required)*	Name	1 Indicator is filled
		2 Indicator Not filled
		3 No indicator available
chc_g_2_4 *(required)*	Age	1 Indicator is filled
		2 Indicator Not filled
		3 No indicator available
chc_g_2_5 *(required)*	Address	1 Indicator is filled
		2 Indicator Not filled
		3 No indicator available
chc_g_2_11 *(required)*	Contact details	1 Indicator is filled
		2 Indicator Not filled
		3 No indicator available
chc_g_2_6 *(required)*	Height	1 Indicator is filled
		2 Indicator Not filled
		3 No indicator available
chc_g_2_7 *(required)*	Weight	1 Indicator is filled
		2 Indicator Not filled
		3 No indicator available
chc_g_2_8 *(required)*	BMI	1 Indicator is filled
		2 Indicator Not filled
		3 No indicator available
chc_g_2_9 *(required)*	Medical history (e.g.Anemia,DM,HT,RTI,Thyroid) and screening results of the same conditions	1 Indicator is filled
		2 Indicator Not filled
		3 No indicator available
chc_g_2_10 *(required)*	Place/facility were the conditions tracked during follow up visits	1 Indicator is filled
		2 Indicator Not filled
		3 No indicator available
CHC > chc_g_group > chc_anc_record_data<br>*Group relevant when: ${chc_f_12_r} =1*		
generated_table_list_label_601	ANC Register	
reserved_name_for_field_list_labels_602		1 Indicator is filled
		2 Indicator Not filled
		3 No indicator available
chc_g_3_1 *(required)*	Date of visit	1 Indicator is filled
		2 Indicator Not filled
		3 No indicator available
chc_g_3_2 *(required)*	Woman ID	1 Indicator is filled
		2 Indicator Not filled
		3 No indicator available
chc_g_3_3 *(required)*	Name	1 Indicator is filled

Field	Question	Answer
		2 Indicator Not filled<br>3 No indicator available
chc_g_3_4 *(required)*	Age	1 Indicator is filled<br>2 Indicator Not filled<br>3 No indicator available
chc_g_3_5 *(required)*	Address	1 Indicator is filled<br>2 Indicator Not filled<br>3 No indicator available
chc_g_3_16 *(required)*	Contact details	1 Indicator is filled<br>2 Indicator Not filled<br>3 No indicator available
chc_g_3_6 *(required)*	Gestational age (GA) at first registration	1 Indicator is filled<br>2 Indicator Not filled<br>3 No indicator available
chc_g_3_7 *(required)*	BMI in first trimester	1 Indicator is filled<br>2 Indicator Not filled<br>3 No indicator available
chc_g_3_18	Height	1 Indicator is filled<br>2 Indicator Not filled<br>3 No indicator available
chc_g_3_19	Weight	1 Indicator is filled<br>2 Indicator Not filled<br>3 No indicator available
chc_g_3_8 *(required)*	Records/columns of all ANC visits (no. of columns) along with provision to track GWG & Hb	1 Indicator is filled<br>2 Indicator Not filled<br>3 No indicator available
chc_g_3_9 *(required)*	Blood Pressure	1 Indicator is filled<br>2 Indicator Not filled<br>3 No indicator available
chc_g_3_10 *(required)*	Blood investigation- CBC at the time of registration	1 Indicator is filled<br>2 Indicator Not filled<br>3 No indicator available
chc_g_3_17 *(required)*	Blood investigation-TSH at the time of registration	1 Indicator is filled<br>2 Indicator Not filled<br>3 No indicator available
chc_g_3_11 *(required)*	OGTT- 1 time	1 Indicator is filled<br>2 Indicator Not filled<br>3 No indicator available
chc_g_3_12 *(required)*	Hb-4 times	1 Indicator is filled<br>2 Indicator Not filled<br>3 No indicator available
chc_g_3_13 *(required)*	Urine routine & microscopy- 4 times	1 Indicator is filled<br>2 Indicator Not filled<br>3 No indicator available
chc_g_3_14 *(required)*	Ultrasound	1 Indicator is filled<br>2 Indicator Not filled<br>3 No indicator available
chc_g_3_15 *(required)*	Td vaccination	1 Indicator is filled<br>2 Indicator Not filled<br>3 No indicator available
CHC > chc_g_group > chc_anc_record_ped<br>*Group relevant when: ${chc_f_13_r} =1*		
generated_table_list_label_622	Pediatric/0-24 Months Infants & Children Register	
reserved_name_for_field_list_labels_623		1 Indicator is filled<br>2 Indicator Not filled<br>3 No indicator available
chc_g_4_1 *(required)*	Date of visit	1 Indicator is filled<br>2 Indicator Not filled<br>3 No indicator available

Field	Question	Answer
chc_g_4_2 *(required)*	Child ID	1 Indicator is filled<br>2 Indicator Not filled<br>3 No indicator available
chc_g_4_3 *(required)*	Name	1 Indicator is filled<br>2 Indicator Not filled<br>3 No indicator available
chc_g_4_4 *(required)*	Age	1 Indicator is filled<br>2 Indicator Not filled<br>3 No indicator available
chc_g_4_5 *(required)*	Sex	1 Indicator is filled<br>2 Indicator Not filled<br>3 No indicator available
chc_g_4_6 *(required)*	Mother Name	1 Indicator is filled<br>2 Indicator Not filled<br>3 No indicator available
chc_g_4_7 *(required)*	Father Name	1 Indicator is filled<br>2 Indicator Not filled<br>3 No indicator available
chc_g_4_8 *(required)*	Address	1 Indicator is filled<br>2 Indicator Not filled<br>3 No indicator available
chc_g_4_17 *(required)*	Contact details	1 Indicator is filled<br>2 Indicator Not filled<br>3 No indicator available
chc_g_4_9 *(required)*	Weight	1 Indicator is filled<br>2 Indicator Not filled<br>3 No indicator available
chc_g_4_10 *(required)*	Height	1 Indicator is filled<br>2 Indicator Not filled<br>3 No indicator available
chc_g_4_11 *(required)*	Tracking of IWG	1 Indicator is filled<br>2 Indicator Not filled<br>3 No indicator available
chc_g_4_12 *(required)*	Identification of danger signs	1 Indicator is filled<br>2 Indicator Not filled<br>3 No indicator available
chc_g_4_13 *(required)*	Morbidities Reported	1 Indicator is filled<br>2 Indicator Not filled<br>3 No indicator available
chc_g_4_14 *(required)*	Treatment Provided	1 Indicator is filled<br>2 Indicator Not filled<br>3 No indicator available
chc_g_4_15 *(required)*	Immunisation Status	1 Indicator is filled<br>2 Indicator Not filled<br>3 No indicator available
chc_g_4_16 *(required)*	Follow-up visit Date	1 Indicator is filled<br>2 Indicator Not filled<br>3 No indicator available
CHC > chc_g_group > chc_labour_room_record_data<br>*Group relevant when: ${chc_f_14_r} =1*		
generated_table_list_label_641	Labour Room Register	
reserved_name_for_field_list_labels_642		1 Indicator is filled<br>2 Indicator Not filled<br>3 No indicator available
chc_g_5_1 *(required)*	Date of admission	1 Indicator is filled<br>2 Indicator Not filled<br>3 No indicator available
chc_g_5_2 *(required)*	Patient ID	1 Indicator is filled<br>2 Indicator Not filled

Field	Question	Answer
		3 No indicator available
chc_g_5_3 *(required)*	Name	1 Indicator is filled<br>2 Indicator Not filled<br>3 No indicator available
chc_g_5_4 *(required)*	Age	1 Indicator is filled<br>2 Indicator Not filled<br>3 No indicator available
chc_g_5_5 *(required)*	Address	1 Indicator is filled<br>2 Indicator Not filled<br>3 No indicator available
chc_g_5_13 *(required)*	Contact details	1 Indicator is filled<br>2 Indicator Not filled<br>3 No indicator available
chc_g_5_6 *(required)*	Time of admission	1 Indicator is filled<br>2 Indicator Not filled<br>3 No indicator available
chc_g_5_7 *(required)*	Type of delivery (e.g. normal,cesarean)	1 Indicator is filled<br>2 Indicator Not filled<br>3 No indicator available
chc_g_5_8 *(required)*	Complications during delivery	1 Indicator is filled<br>2 Indicator Not filled<br>3 No indicator available
chc_g_5_9 *(required)*	Birth weight	1 Indicator is filled<br>2 Indicator Not filled<br>3 No indicator available
chc_g_5_10 *(required)*	APGAR score	1 Indicator is filled<br>2 Indicator Not filled<br>3 No indicator available
chc_g_5_11 *(required)*	Time of discharge	1 Indicator is filled<br>2 Indicator Not filled<br>3 No indicator available
chc_g_5_12 *(required)*	Referral information.	1 Indicator is filled<br>2 Indicator Not filled<br>3 No indicator available
CHC > chc_g_group > chc_lab_record<br>*Group relevant when: ${chc_f_15_r} =1*		
generated_table_list_label_656	Laboratory Record	
reserved_name_for_field_list_labels_657		1 Indicator is filled<br>2 Indicator Not filled<br>3 No indicator available
chc_g_6_1 *(required)*	Date	1 Indicator is filled<br>2 Indicator Not filled<br>3 No indicator available
chc_g_6_2 *(required)*	Sample ID	1 Indicator is filled<br>2 Indicator Not filled<br>3 No indicator available
chc_g_6_3 *(required)*	Patient ID	1 Indicator is filled<br>2 Indicator Not filled<br>3 No indicator available
chc_g_6_4 *(required)*	Name	1 Indicator is filled<br>2 Indicator Not filled<br>3 No indicator available
chc_g_6_5 *(required)*	Type of investigation	1 Indicator is filled<br>2 Indicator Not filled<br>3 No indicator available
chc_g_6_6 *(required)*	Investigation results	1 Indicator is filled<br>2 Indicator Not filled<br>3 No indicator available
chc_g_6_7 *(required)*	Remarks	1 Indicator is filled

Field	Question	Answer
		2 Indicator Not filled<br>3 No indicator available
chc_g_6_8 *(required)*	Date of report	1 Indicator is filled<br>2 Indicator Not filled<br>3 No indicator available
CHC > chc_g_group > chc_birth_record<br>*Group relevant when: ${chc_f_16_r} =1*		
generated_table_list_label_666	Birth Register	
reserved_name_for_field_list_labels_667		1 Indicator is filled<br>2 Indicator Not filled<br>3 No indicator available
chc_g_7_1 *(required)*	Date of event	1 Indicator is filled<br>2 Indicator Not filled<br>3 No indicator available
chc_g_7_2 *(required)*	Child ID	1 Indicator is filled<br>2 Indicator Not filled<br>3 No indicator available
chc_g_7_3 *(required)*	Name of child (if applicable)	1 Indicator is filled<br>2 Indicator Not filled<br>3 No indicator available
chc_g_7_4 *(required)*	Mother's Name	1 Indicator is filled<br>2 Indicator Not filled<br>3 No indicator available
chc_g_7_5 *(required)*	Father's Name	1 Indicator is filled<br>2 Indicator Not filled<br>3 No indicator available
chc_g_7_6 *(required)*	Date of birth	1 Indicator is filled<br>2 Indicator Not filled<br>3 No indicator available
chc_g_7_7 *(required)*	Place of birth	1 Indicator is filled<br>2 Indicator Not filled<br>3 No indicator available
chc_g_7_9 *(required)*	Attendant details (e.g., ASHA, AWW)	1 Indicator is filled<br>2 Indicator Not filled<br>3 No indicator available
chc_g_7_10 *(required)*	Remarks	1 Indicator is filled<br>2 Indicator Not filled<br>3 No indicator available
CHC > chc_g_group > chc_death_record<br>*Group relevant when: ${chc_f_16_1_r} =1*		
generated_table_list_label_677	Death Register	
reserved_name_for_field_list_labels_678		1 Indicator is filled<br>2 Indicator Not filled<br>3 No indicator available
chc_d_7_1 *(required)*	Date of event	1 Indicator is filled<br>2 Indicator Not filled<br>3 No indicator available
chc_d_7_2 *(required)*	Child ID	1 Indicator is filled<br>2 Indicator Not filled<br>3 No indicator available
chc_d_7_3 *(required)*	Name of child (if applicable)	1 Indicator is filled<br>2 Indicator Not filled<br>3 No indicator available
chc_d_7_4 *(required)*	Mother's Name	1 Indicator is filled<br>2 Indicator Not filled<br>3 No indicator available
chc_d_7_5 *(required)*	Father's Name	1 Indicator is filled<br>2 Indicator Not filled<br>3 No indicator available
chc_d_7_6 *(required)*	Date of death	1 Indicator is filled

Field	Question	Answer
		2 Indicator Not filled<br>3 No indicator available
chc_d_7_7 *(required)*	Place of death	1 Indicator is filled<br>2 Indicator Not filled<br>3 No indicator available
chc_d_7_8 *(required)*	Cause of death	1 Indicator is filled<br>2 Indicator Not filled<br>3 No indicator available
chc_d_7_9 *(required)*	Attendant details (e.g., ASHA, AWW)	1 Indicator is filled<br>2 Indicator Not filled<br>3 No indicator available
chc_d_7_10 *(required)*	Remarks	1 Indicator is filled<br>2 Indicator Not filled<br>3 No indicator available
CHC > chc_g_group > chc_referral_record<br>*Group relevant when: ${chc_f_17_r} =1*		
generated_table_list_label_689	Referral Register	
reserved_name_for_field_list_labels_690		1 Indicator is filled<br>2 Indicator Not filled<br>3 No indicator available
chc_g_8_1 *(required)*	Date of referral	1 Indicator is filled<br>2 Indicator Not filled<br>3 No indicator available
chc_g_8_2 *(required)*	Patient ID	1 Indicator is filled<br>2 Indicator Not filled<br>3 No indicator available
chc_g_8_3 *(required)*	Name	1 Indicator is filled<br>2 Indicator Not filled<br>3 No indicator available
chc_g_8_4 *(required)*	Address	1 Indicator is filled<br>2 Indicator Not filled<br>3 No indicator available
chc_g_8_5 *(required)*	Reason for referral	1 Indicator is filled<br>2 Indicator Not filled<br>3 No indicator available
chc_g_8_6 *(required)*	Referring facility	1 Indicator is filled<br>2 Indicator Not filled<br>3 No indicator available
chc_g_8_7 *(required)*	Receiving facility	1 Indicator is filled<br>2 Indicator Not filled<br>3 No indicator available
chc_g_8_8 *(required)*	Date of receiving feedback	1 Indicator is filled<br>2 Indicator Not filled<br>3 No indicator available
chc_g_8_9 *(required)*	Follow-up visit date	1 Indicator is filled<br>2 Indicator Not filled<br>3 No indicator available
CHC > chc_i_group		
chc_i_1_1 *(required)*	Iron, Vitamin, and Nutritional Supplements<br>*Response constrained to: not(selected(${chc_i_1_1} , '9999') and count-selected(${chc_i_1_1}) > 1)*	1 Ferrous Salts equivalent to 60 mg of Elemental Iron<br>2 Ferrous Salts Oral Liquid equivalent to 25 mg of Elemental Iron/ml<br>3 Ferrous Salt (A) + Folic Acid (B) Tablet 45 mg Elemental Iron (A) + 400 mcg Folic Acid (B)<br>4 Ferrous Salt (A) + Folic Acid (B) Tablet 60 mg Elemental

Field	Question	Answer
		Iron (A)+ 500 mcg Folic Acid (B)
		5 Ferrous Salt (A) + Folic Acid (B) Oral Liqiud 20 mg Elemental Iron(A) + 100 mcg (B)/ml
		6 Folic Acid Tablet 5 mg
		7 Folic Acid Tablet 400 mcg
		8 Folic Acid 5 mg Injection 1 mg/ml
		9 Iron Sucrose Injection 20 mg/ml
		10 Phytomenadione (Vitamin K1) Tablet 10 mg
		11 Phytomenadione (Vitamin K1) Inj 10 mg/ml
		12 Calcium with Vitamin D Tablets USP
		13 Syrup Calcium with Phosphate in the ratio 2:1 with Vitamin D
		14 Tablet Calcium Carbonate 650 mg equivalent to Elemental Calcium 250 mg and Cholecalciferol USP 125 mg
		15 Cholecalciferol Tablet 1000 IU
		16 Cholecalciferol Tablet 60,000 IU
		17 Cholecalciferol Oral Liquid 400 IU/ml
		18 Cholecalciferol Granules 60,000 IU in Sachet
		19 Cholecalciferol Injection of 6,00,000 IU
		20 Vitamin D3 60,000 Granules in 1 gm Sachet
		21 Vitamin A Oral liquid 100000 IU/ml
		22 Zinc Sulphate tablets
		23 Multiple Micronutrients (MMN) (WINGS)
		99 Other Medicine (Specify)
		9999 None
CHC > chc_i_group > [chc_i_1_cal1] (1)		(Repeated group)
CHC > chc_i_group > [chc_i_1_cal1] (1) > Availability Status of Essential Medicines Required at chc-SHC for MCH Care [chc_i_1_cal1]} *Group relevant when: ${chc_i_1_1} !=9999*		
chc_i_1_t *(required)*	Target beneficiary	1 Preconception women (18-35 Years)<br>2 Pregnant women<br>3 Postnatal/ lactating women<br>4 0-6 Months Infants<br>5 6-24 Months Infants & Children
chc_i_1_2 *(required)*	Projected Monthly Requirements	
chc_i_1_3 *(required)*	Current Numbers in Stock	
chc_i_1_4 *(required)*	Stock Out in the last 3 month	1 Yes<br>2 No
chc_i_2_1 *(required)*	Antibiotics *Response constrained to: not(selected(${chc_i_2_1} , '9999') and count-selected(${chc_i_2_1}) > 1)*	1 Inj. Gentamicin 80 mg (40 mg/ml)<br>2 Injection Ampicillin<br>3 Injection Amikacin

Field	Question	Answer
		4 Albendazole Tablet 400 mg<br>5 Albendazole Oral Liquid 200 mg/5 ml<br>6 Amoxicillin Capsule 250 mg<br>7 Amoxicillin Tablet 500 mg<br>8 Ampicillin Capsule 500 mg<br>9 Ampicillin Injection 500 mg/ml<br>10 Amoxicillin (500 mg) + Clavulanic Acid (125 mg) Tablet<br>11 Amoxicillin (875 mg) + Clavulanic Acid (125 mg) Tablet<br>12 Benzylpenicillin Injection 10 lakh unit<br>13 Cefixime Tablet 200 mg<br>14 Cefotaxime Injection 1 g/vial<br>15 Piperacillin (A) + Tazobactam (B) Powder for Injection 1 g (A) + 125 mg (B)/2 g (A) + 250 mg (B)/4 g (A) + 500 mg (B)<br>16 Azithromycin Tablet 250 mg/500 mg/Oral Liquid 200 mg/5 ml<br>17 Norfloxacin Tablet 400 mg<br>18 Ciprofloxacin Tablet 250 mg/500 mg/Oral Liquid 250 mg/5 ml<br>19 Ciprofloxacin I.V 200 mg/100 ml<br>20 Co-trimoxazole [Sulphamethoxazol e (A) +Trimethoprim (B)] Tablet 400 mg (A) + 80 mg (B)/Tablet 800 mg (A) + 160 mg (B) /Oral liquid 200 mg (A) + 40 mg (B)/5 ml<br>21 Doxycycline Capsule 100 mg<br>22 Metronidazole Tablet 200 mg, 400 mg<br>23 Metronidazole Injection 5 mg/ml, 500 mg/100 ml<br>24 Levofloxacin Tablet 250 mg/500 mg/750 mg<br>25 Tetracycline/Chloramphenicol eye drops<br>26 Atropine eye drops<br>27 Chloramphenicol<br>99 Other Medicine(Specify)<br>9999 None
CHC > chc_i_group > [chc_i_2_cal1] (1)		(Repeated group)
CHC > chc_i_group > [chc_i_2_cal1] (1) > Availability Status of Essential Medicines Required at chc-SHC for MCH Care [chc_i_2_cal1]}<br>*Group relevant when: ${chc_i_2_1} !=9999*		
chc_i_2_t *(required)*	Target beneficiary	1 Preconception women (18-35 Years)<br>2 Pregnant women<br>3 Postnatal/ lactating women<br>4 0-6 Months Infants<br>5 6-24 Months Infants & Children
chc_i_2_2 *(required)*	Projected Monthly Requirements	

Field	Question	Answer
chc_i_2_3 *(required)*	Current Numbers in Stock	
chc_i_2_4 *(required)*	Stock Out in the last 3 month	1 Yes<br>2 No
chc_i_3_1 *(required)*	Antihypertensives and Seizure Management<br>*Response constrained to: not(selected(${chc_i_3_1} , '9999') and count-selected(${chc_i_3_1}) > 1)*	1 Labetalol Injection 5 mg/ml/20 mg/2ml<br>2 Labetalol Tablet 100 mg<br>3 Methyldopa Tablet 250 mg/500 mg<br>4 Enalapril Tablet 2.5 mg/5 mg/10 mg<br>5 Tab Digoxin - Digoxin IP 250 ug/tab<br>6 Inj. Lorazepam 1mg/ml<br>7 Carbamazepine Tablet 100 mg, 200 mg<br>8 Lorazepam Tablet 1 mg<br>9 Lorazepam Injection 1 or 2 mg/ml<br>10 Magnesium Sulphate Injection 500 mg/ml<br>11 Magnesium Sulphate Injection IP 50% w/v<br>12 Phenytoin Tablet 50 mg/100 mg, 300 mg<br>13 Phenytoin Injection 25 mg/ml, 50 mg/ml<br>14 Sodium Valproate Tablet 100 mg/500 mg<br>15 Inj. Calcium Gluconate 1gm<br>99 Other Medicine(Specify)<br>9999 None
CHC > chc_i_group > [chc_i_3_cal1] (1)		(Repeated group)
CHC > chc_i_group > [chc_i_3_cal1] (1) > Availability Status of Essential Medicines Required at chc-SHC for MCH Care [chc_i_3_cal1]}<br>*Group relevant when: ${chc_i_3_1} !=9999*		
chc_i_3_t *(required)*	Target beneficiary	1 Preconception women (18-35 Years)<br>2 Pregnant women<br>3 Postnatal/ lactating women<br>4 0-6 Months Infants<br>5 6-24 Months Infants & Children
chc_i_3_2 *(required)*	Projected Monthly Requirements	
chc_i_3_3 *(required)*	Current Numbers in Stock	
chc_i_3_4 *(required)*	Stock Out in the last 3 month	1 Yes<br>2 No
chc_i_4_1 *(required)*	Analgesics and Antipyretics<br>*Response constrained to: not(selected(${chc_i_4_1} , '9999') and count-selected(${chc_i_4_1}) > 1)*	1 Diclofenac Tablet 50 mg<br>2 Diclofenac Injection 25 mg/ml<br>3 Diclofenac Injection 25 mg/3 ml<br>4 Ibuprofen Tablet 200 mg<br>5 Ibuprofen Tablet 400 mg<br>6 Ibuprofen Syrup 100 mg/5 ml<br>7 Paracetamol Tablet 500 mg/650 mg<br>8 Paracetamol Injection 150 mg/ml<br>9 Paracetamol Syrup 125 mg/5 ml<br>10 Hydroxychloroquine Tablet 200 mg<br>11 Hydroxychloroquine Tablet 400 mg<br>99 Other Medicine (Specify)

Field	Question	Answer
		9999 None
CHC > chc_i_group > [chc_i_4_cal1] (1)		(Repeated group)
CHC > chc_i_group > [chc_i_4_cal1] (1) > Availability Status of Essential Medicines Required at chc-SHC for MCH Care [chc_i_4_cal1]} *Group relevant when: ${chc_i_4_1} !=9999*		
chc_i_4_t *(required)*	Target beneficiary	1 Preconception women (18-35 Years)<br>2 Pregnant women<br>3 Postnatal/ lactating women<br>4 0-6 Months Infants<br>5 6-24 Months Infants & Children
chc_i_4_2 *(required)*	Projected Monthly Requirements	
chc_i_4_3 *(required)*	Current Numbers in Stock	
chc_i_4_4 *(required)*	Stock Out in the last 3 month	1 Yes<br>2 No
chc_i_5_1	Anthelmintics *Response constrained to: not(selected(${chc_i_5_1} , '9999') and count-selected(${chc_i_5_1}) > 1)*	1 Tab. Albendazole (Chewable) 400 mg/Tab<br>2 Albendazole Oral Solution 200 mg/5 ml<br>3 Oral Antimalarial<br>99 Other Medicine (Specify)<br>9999 None
CHC > chc_i_group > [chc_i_5_cal1] (1)		(Repeated group)
CHC > chc_i_group > [chc_i_5_cal1] (1) > Availability Status of Essential Medicines Required at chc-SHC for MCH Care [chc_i_5_cal1]} *Group relevant when: ${chc_i_5_1} !=9999*		
chc_i_5_t *(required)*	Target beneficiary	1 Preconception women (18-35 Years)<br>2 Pregnant women<br>3 Postnatal/ lactating women<br>4 0-6 Months Infants<br>5 6-24 Months Infants & Children
chc_i_5_2 *(required)*	Projected Monthly Requirements	
chc_i_5_3 *(required)*	Current Numbers in Stock	
chc_i_5_4 *(required)*	Stock Out in the last 3 month	1 Yes<br>2 No
chc_i_6_1 *(required)*	Diabetic & Thyroid Medications *Response constrained to: not(selected(${chc_i_6_1} , '9999') and count-selected(${chc_i_6_1}) > 1)*	1 Inj. Human Soluble Insulin (40 IU/ml)<br>2 Tab. Metformin HCl (Coated) (500 mg/Tab)<br>3 Tab. Eltroxin / Thyroxine (100 mcg)<br>99 Other Medicine (Specify)<br>9999 None
CHC > chc_i_group > [chc_i_6_cal1] (1)		(Repeated group)
CHC > chc_i_group > [chc_i_6_cal1] (1) > Availability Status of Essential Medicines Required at chc-SHC for MCH Care [chc_i_6_cal1]} *Group relevant when: ${chc_i_6_1} !=9999*		
chc_i_6_t *(required)*	Target beneficiary	1 Preconception women (18-35 Years)<br>2 Pregnant women<br>3 Postnatal/ lactating women<br>4 0-6 Months Infants<br>5 6-24 Months Infants & Children
chc_i_6_2 *(required)*	Projected Monthly Requirements	
chc_i_6_3 *(required)*	Current Numbers in Stock	
chc_i_6_4 *(required)*	Stock Out in the last 3 month	1 Yes<br>2 No
chc_i_7_1	Other Miscellaneous *Response constrained to: not(selected(${chc_i_7_1} , '9999') and count-selected(${chc_i_7_1}) > 1)*	1 Inj. Td (0.5 ml/Amp)<br>2 Tab. Digoxin (0.25 mg/Tab)<br>3 Tab. Frusemide (40 mg/Tab)<br>4 Inj. Frusemide (10 mg/1 ml)<br>5 Inj. Betamethasone Sod. Phosphate (4 mg/ml)

Field	Question	Answer
		6 Inj. Human Anti-D Immunoglobulin (300 mcg/1.5 ml)<br>7 IV Fluids<br>8 ORS Sachet<br>9 Zinc Tablet<br>10 Povidone Iodine Ointment (5% w/v)<br>11 Sanitary Napkins<br>12 Vaccines (As per National Programme)<br>99 Other Medicine (Specify)<br>9999 None
CHC > chc_i_group > [chc_i_7_cal1] (1)		(Repeated group)
CHC > chc_i_group > [chc_i_7_cal1] (1) > Availability Status of Essential Medicines Required at chc-SHC for MCH Care [chc_i_7_cal1]}<br>*Group relevant when: ${chc_i_7_1} !=9999*		
chc_i_7_t *(required)*	Target beneficiary	1 Preconception women (18-35 Years)<br>2 Pregnant women<br>3 Postnatal/ lactating women<br>4 0-6 Months Infants<br>5 6-24 Months Infants & Children
chc_i_7_2 *(required)*	Projected Monthly Requirements	
chc_i_7_3 *(required)*	Current Numbers in Stock	
chc_i_7_4 *(required)*	Stock Out in the last 3 month	1 Yes<br>2 No
chc_7_1_other	Other Medicine (Specify)<br>*Question relevant when: ${chc_i_7_1} =99*	
CHC > chc_j_group		
CHC > chc_j_group > Facility's Beneficiaries (Patient) Load		
chc_j_note	Please mention the Numbers (Last 12 Month Data) against each(Please enter 999 if data not available)<br>*Please fill 999 if no record available*	
chc_j_1 *(required)*	Eligible couples<br>*Response constrained to: (.>=0 and .<=1000) or (.=999)*	
chc_j_2 *(required)*	Preconception women (18-35 years)<br>*Response constrained to: (.>=0 and .<=1000) or (.=999)*	
chc_j_3 *(required)*	Pregnant women<br>*Response constrained to: (.>=0 and .<=1000) or (.=999)*	
chc_j_4 *(required)*	Postnatal women<br>*Response constrained to: (.>=0 and .<=1000) or (.=999)*	
chc_j_5 *(required)*	0 to 6 months infants<br>*Response constrained to: (.>=0 and .<=1000) or (.=999)*	
chc_j_6 *(required)*	6 to 12 months infants<br>*Response constrained to: (.>=0 and .<=1000) or (.=999)*	
chc_j_7 *(required)*	12 to 24 months children<br>*Response constrained to: (.>=0 and .<=1000) or (.=999)*	
chc_j_8 *(required)*	Preconception women (18-35 years) screened for medical conditions<br>*Response constrained to: (.>=0 and .<=1000) or (.=999)*	
chc_j_9 *(required)*	Preconception women (18-35 years) screened for depressive symptoms-<br>*Response constrained to: (.>=0 and .<=1000) or (.=999)*	
CHC > chc_j_group > Preconception women (18-35 years) screened for medical conditions and depressive symptoms-<br>*Group relevant when: ${chc_j_2} != 0 and ${chc_j_2} != 999*		
chc_j_8_note	Please mention the Number of Preconception women (18-35 years) screened (Last 12 Month Data) against each	
chc_j_8_1 *(required)*	RTI/STI<br>*Response constrained to: (.>=0 and .<=1000) or (.=999)*	
chc_j_8_2 *(required)*	TB<br>*Response constrained to: (.>=0 and .<=1000) or (.=999)*	
chc_j_8_3 *(required)*	Epilepsy<br>*Response constrained to: (.>=0 and .<=1000) or (.=999)*	
chc_j_8_4 *(required)*	Syphilis<br>*Response constrained to: (.>=0 and .<=1000) or (.=999)*	

Field	Question	Answer
chc_j_8_5 *(required)*	Thyroid status<br>*Response constrained to: (.>=0 and .<=1000) or (.=999)*	
chc_j_8_6 *(required)*	Blood pressure<br>*Response constrained to: (.>=0 and .<=1000) or (.=999)*	
chc_j_8_7 *(required)*	Blood sugar<br>*Response constrained to: (.>=0 and .<=1000) or (.=999)*	
chc_j_8_8 *(required)*	Height measured<br>*Response constrained to: (.>=0 and .<=1000) or (.=999)*	
chc_j_8_9 *(required)*	Weight measured<br>*Response constrained to: (.>=0 and .<=1000) or (.=999)*	
chc_j_8_10 *(required)*	Hemoglobin tested<br>*Response constrained to: (.>=0 and .<=1000) or (.=999)*	
chc_j_8_11 *(required)*	PHQ 2<br>*Response constrained to: (.>=0 and .<=1000) or (.=999)*	
CHC > chc_j_group > Preconception women (aged 18-35 years) identified with medical conditions & depressive symptoms following screening-<br>*Group relevant when: ${chc_j_2} != 0 and ${chc_j_2} != 999*		
chc_j_9_note	Please mention the Number of Preconception women (aged 18-35 years) identified with medical conditions & depressive symptoms following screening (Last 12 Month Data) against each	
chc_j_9_1 *(required)*	RTI/STI<br>*Response constrained to: (.>=0 and .<=1000) or (.=999)*	
chc_j_9_2 *(required)*	TB<br>*Response constrained to: (.>=0 and .<=1000) or (.=999)*	
chc_j_9_3 *(required)*	Epilepsy<br>*Response constrained to: (.>=0 and .<=1000) or (.=999)*	
chc_j_9_4 *(required)*	Syphilis<br>*Response constrained to: (.>=0 and .<=1000) or (.=999)*	
chc_j_9_5 *(required)*	Hypothyroidism (TSH > 5.5 IU/mL)<br>*Response constrained to: (.>=0 and .<=1000) or (.=999)*	
chc_j_9_6 *(required)*	Hyperthyroidism (TSH <0.4 IU/mL)<br>*Response constrained to: (.>=0 and .<=1000) or (.=999)*	
chc_j_9_7 *(required)*	Hypertension<br>*Response constrained to: (.>=0 and .<=1000) or (.=999)*	
chc_j_9_7_1 *(required)*	Hypotension<br>*Response constrained to: (.>=0 and .<=1000) or (.=999)*	
chc_j_9_8 *(required)*	Prediabetes (HbA1c 5.7% to 6.4%)<br>*Response constrained to: (.>=0 and .<=1000) or (.=999)*	
chc_j_9_9 *(required)*	Diabetes mellitus (HbA1c ≥6.5%)<br>*Response constrained to: (.>=0 and .<=1000) or (.=999)*	
chc_j_9_10 *(required)*	BMI<16 kg/m2<br>*Response constrained to: (.>=0 and .<=1000) or (.=999)*	
chc_j_9_11 *(required)*	BMI 16-18.49 kg/m2<br>*Response constrained to: (.>=0 and .<=1000) or (.=999)*	
chc_j_9_12 *(required)*	BMI 18.5- <21 kg/m2<br>*Response constrained to: (.>=0 and .<=1000) or (.=999)*	
chc_j_9_13 *(required)*	Severe anemia (Hb < 8 g/dL)<br>*Response constrained to: (.>=0 and .<=1000) or (.=999)*	
chc_j_9_14 *(required)*	Moderate anemia (Hb 7 to 11.99 g/dL)<br>*Response constrained to: (.>=0 and .<=1000) or (.=999)*	
chc_j_9_15 *(required)*	Depressive symptoms<br>*Response constrained to: (.>=0 and .<=1000) or (.=999)*	
CHC > chc_j_group > Preconception women (aged 18-35 years) managed<br>*Group relevant when: ${chc_j_2} != 0 and ${chc_j_2} != 999*		
chc_j_10_note	Please mention the Number of Preconception women (aged 18-35 years) managed for (Last 12 Month Data) the following conditions	
chc_j_10_1 *(required)*	RTI/STI<br>*Response constrained to: (.>=0 and .<=1000) or (.=999)*	
chc_j_10_2 *(required)*	TB<br>*Response constrained to: (.>=0 and .<=1000) or (.=999)*	
chc_j_10_3 *(required)*	Epilepsy<br>*Response constrained to: (.>=0 and .<=1000) or (.=999)*	
chc_j_10_4 *(required)*	Syphilis<br>*Response constrained to: (.>=0 and .<=1000) or (.=999)*	
chc_j_10_5 *(required)*	Hypothyroidism (TSH > 5.5 IU/mL)	

Field	Question	Answer
	Response constrained to: (.>=0 and .<=1000) or (.=999)	
chc_j_10_6 *(required)*	Hyperthyroidism (TSH <0.4 IU/mL)<br>*Response constrained to: (.>=0 and .<=1000) or (.=999)*	
chc_j_10_7 *(required)*	Hypertension<br>*Response constrained to: (.>=0 and .<=1000) or (.=999)*	
chc_j_10_7_1 *(required)*	Hypotension<br>*Response constrained to: (.>=0 and .<=1000) or (.=999)*	
chc_j_10_8 *(required)*	Prediabetes (HbA1c 5.7% to 6.4%)<br>*Response constrained to: (.>=0 and .<=1000) or (.=999)*	
chc_j_10_9 *(required)*	Diabetes mellitus (HbA1c ≥6.5%)<br>*Response constrained to: (.>=0 and .<=1000) or (.=999)*	
chc_j_10_10 *(required)*	BMI<16 kg/m2<br>*Response constrained to: (.>=0 and .<=1000) or (.=999)*	
chc_j_10_11 *(required)*	BMI 16-18.49 kg/m2<br>*Response constrained to: (.>=0 and .<=1000) or (.=999)*	
chc_j_10_12 *(required)*	BMI 18.5- <21 kg/m2<br>*Response constrained to: (.>=0 and .<=1000) or (.=999)*	
chc_j_10_13 *(required)*	Severe anemia (Hb <8 g/dL)<br>*Response constrained to: (.>=0 and .<=1000) or (.=999)*	
chc_j_10_14 *(required)*	Moderate anemia (Hb 8 to 11.99 g/dL)<br>*Response constrained to: (.>=0 and .<=1000) or (.=999)*	
chc_j_10_15 *(required)*	Depressive symptoms<br>*Response constrained to: (.>=0 and .<=1000) or (.=999)*	
chc_j_10_16 *(required)*	No. of preconception women who were given IFA as per recommendation<br>*Response constrained to: (.>=0 and .<=1000) or (.=999)*	
chc_j_10_17 *(required)*	No. of preconception women who counselled for Nutrition<br>*Response constrained to: (.>=0 and .<=1000) or (.=999)*	
chc_j_10_18 *(required)*	No. of preconception women who counselled for WASH intervention<br>*Response constrained to: (.>=0 and .<=1000) or (.=999)*	
CHC > chc_j_group > Pregnant women screened/tested for -<br>*Group relevant when: ${chc_j_2} != 0 and ${chc_j_2} != 999*		
chc_j_11 *(required)*	Number of Pregnant Women Registered for ANC Care in the last 12 months<br>*Question relevant when: ${chc_j_3} != 0 and ${chc_j_3} != 999*<br>*Response constrained to: (.>=0 and .<=1000) or (.=999)*	
chc_j_11_note	Please mention the Number of Pregnant women screened/tested for (Last 12 Month Data)	
chc_j_11_1 *(required)*	Blood grouping<br>*Response constrained to: (.>=0 and .<=1000) or (.=999)*	
chc_j_11_2 *(required)*	Complete Blood Count (CBC) at the time of registration<br>*Response constrained to: (.>=0 and .<=1000) or (.=999)*	
chc_j_11_3 *(required)*	HIV<br>*Response constrained to: (.>=0 and .<=1000) or (.=999)*	
chc_j_11_4 *(required)*	Syphilis<br>*Response constrained to: (.>=0 and .<=1000) or (.=999)*	
chc_j_11_5 *(required)*	Urine routine & microscopy<br>*Response constrained to: (.>=0 and .<=1000) or (.=999)*	
chc_j_11_6 *(required)*	Thyroid status / TSH<br>*Response constrained to: (.>=0 and .<=1000) or (.=999)*	
chc_j_11_7 *(required)*	Gestational Diabetes Mellitus (GDM) / OGTT<br>*Response constrained to: (.>=0 and .<=1000) or (.=999)*	
chc_j_11_8 *(required)*	Blood pressure<br>*Response constrained to: (.>=0 and .<=1000) or (.=999)*	
chc_j_11_9 *(required)*	Height measured in First Trimester<br>*Response constrained to: (.>=0 and .<=1000) or (.=999)*	
chc_j_11_10 *(required)*	Weight measured in First Trimester<br>*Response constrained to: (.>=0 and .<=1000) or (.=999)*	
chc_j_11_11 *(required)*	Weight measured in all trimesters<br>*Response constrained to: (.>=0 and .<=1000) or (.=999)*	
chc_j_11_12 *(required)*	Patients Health Questionnaire (PHQ-2) in each trimester<br>*Response constrained to: (.>=0 and .<=1000) or (.=999)*	
chc_j_11_13 *(required)*	Tested for Hemoglobin (Hb) 4 time during pregnancy<br>*Response constrained to: (.>=0 and .<=1000) or (.=999)*	
CHC > chc_j_group > Pregnant women identified with medical conditions & depressive symptoms following screening-<br>*Group relevant when: ${chc_j_3} != 0 and ${chc_j_3} != 999*		

Field	Question	Answer
chc_j_12_note	Please mention the Number of Pregnant women identified with medical conditions & depressive symptoms following screening (Last 12 Month Data)	
chc_j_12_1 *(required)*	HIV<br>*Response constrained to: (.>=0 and .<=1000) or (.=999)*	
chc_j_12_2 *(required)*	Syphilis<br>*Response constrained to: (.>=0 and .<=1000) or (.=999)*	
chc_j_12_3 *(required)*	Hypothyroidism<br>*Response constrained to: (.>=0 and .<=1000) or (.=999)*	
chc_j_12_4 *(required)*	Hyperthyroidism<br>*Response constrained to: (.>=0 and .<=1000) or (.=999)*	
chc_j_12_5 *(required)*	Hypertension<br>*Response constrained to: (.>=0 and .<=1000) or (.=999)*	
chc_j_12_5_1 *(required)*	Hypotension<br>*Response constrained to: (.>=0 and .<=1000) or (.=999)*	
chc_j_12_6 *(required)*	Diabetes mellitus<br>*Response constrained to: (.>=0 and .<=1000) or (.=999)*	
chc_j_12_7 *(required)*	BMI < 18.5 kg/m2<br>*Response constrained to: (.>=0 and .<=1000) or (.=999)*	
chc_j_12_8 *(required)*	BMI < 25 kg/m2<br>*Response constrained to: (.>=0 and .<=1000) or (.=999)*	
chc_j_12_9 *(required)*	Pregnant women with inadequate gestational weight gain<br>*Response constrained to: (.>=0 and .<=1000) or (.=999)*	
chc_j_12_10 *(required)*	Severe anemia (Hb <8 g/dL)<br>*Response constrained to: (.>=0 and .<=1000) or (.=999)*	
chc_j_12_11 *(required)*	Moderate anemia (Hb 8 to 11.99 g/dL)<br>*Response constrained to: (.>=0 and .<=1000) or (.=999)*	
chc_j_12_12 *(required)*	Depressive symptoms<br>*Response constrained to: (.>=0 and .<=1000) or (.=999)*	
CHC > chc_j_group > Pregnant women managed for treatment<br>*Group relevant when: ${chc_j_3} != 0 and ${chc_j_3} != 999*		
chc_j_14_note	Please mention the Number of Pregnant women managed for following conditions(Last 12 Month Data)	
chc_j_14_1 *(required)*	HIV<br>*Response constrained to: (.>=0 and .<=1000) or (.=999)*	
chc_j_14_2 *(required)*	Syphilis<br>*Response constrained to: (.>=0 and .<=1000) or (.=999)*	
chc_j_14_3 *(required)*	Hypothyroidism<br>*Response constrained to: (.>=0 and .<=1000) or (.=999)*	
chc_j_14_4 *(required)*	Hyperthyroidism<br>*Response constrained to: (.>=0 and .<=1000) or (.=999)*	
chc_j_14_5 *(required)*	Hypertension<br>*Response constrained to: (.>=0 and .<=1000) or (.=999)*	
chc_j_14_5_1 *(required)*	Hypotension<br>*Response constrained to: (.>=0 and .<=1000) or (.=999)*	
chc_j_14_6 *(required)*	Diabetes mellitus<br>*Response constrained to: (.>=0 and .<=1000) or (.=999)*	
chc_j_14_7 *(required)*	Inadequate gestational weight gain (IGWG)<br>*Response constrained to: (.>=0 and .<=1000) or (.=999)*	
chc_j_14_8 *(required)*	Severe anemia (Hb < 7 g/dL)<br>*Response constrained to: (.>=0 and .<=1000) or (.=999)*	
chc_j_14_9 *(required)*	Moderate anemia (Hb 7 to 9.9 g/dL)<br>*Response constrained to: (.>=0 and .<=1000) or (.=999)*	
chc_j_14_10 *(required)*	Depressive symptoms<br>*Response constrained to: (.>=0 and .<=1000) or (.=999)*	
chc_j_14_11 *(required)*	Other medical conditions<br>*Response constrained to: (.>=0 and .<=1000) or (.=999)*	
chc_j_14_12 *(required)*	No. of pregnant women who were given IFA as per recommendation<br>*Response constrained to: (.>=0 and .<=1000) or (.=999)*	
chc_j_14_13 *(required)*	No. of pregnant women who counselled for Nutrition<br>*Response constrained to: (.>=0 and .<=1000) or (.=999)*	
chc_j_14_14 *(required)*	No. of pregnant women who counselled for WASH intervention<br>*Response constrained to: (.>=0 and .<=1000) or (.=999)*	
CHC > chc_j_group > chc_j_1_group<br>*Group relevant when: ${chc_j_3} != 0 and ${chc_j_3} != 999*		

Field	Question	Answer
chc_j_15 *(required)*	Pregnant women referred during ANC<br>*Response constrained to: (.>=0 and .<=1000) or (.=999)*	
chc_j_16 *(required)*	Pregnant women with atleast 4-ANC check-ups<br>*Response constrained to: (.>=0 and .<=1000) or (.=999)*	
chc_j_17 *(required)*	Pregnant women with atleast 8-ANC check-ups<br>*Response constrained to: (.>=0 and .<=1000) or (.=999)*	
chc_j_18 *(required)*	Pregnant women given corticosteroids for preterm labor<br>*Response constrained to: (.>=0 and .<=1000) or (.=999)*	
chc_j_19 *(required)*	Registered antenatal mothers who came for delivery<br>*Response constrained to: (.>=0 and .<=1000) or (.=999)*	
chc_j_20 *(required)*	Institutional deliveries in facility<br>*Response constrained to: (.>=0 and .<=1000) or (.=999)*	
chc_j_20_1	Institutional deliveries other facility<br>*Response constrained to: (.>=0 and .<=1000) or (.=999)*	
chc_j_21 *(required)*	Home deliveries<br>*Response constrained to: (.>=0 and .<=1000) or (.=999)*	
chc_j_22 *(required)*	Maternal deaths<br>*Response constrained to: (.>=0 and .<=1000) or (.=999)*	
chc_j_23 *(required)*	Live births<br>*Response constrained to: (.>=0 and .<=1000) or (.=999)*	
chc_j_24 *(required)*	Babies born with Very low birth weight (VLBW) < 1,500 grams<br>*Response constrained to: (.>=0 and .<=1000) or (.=999)*	
chc_j_25 *(required)*	Babies born with low birth weight (LBW) < 2,500 grams<br>*Response constrained to: (.>=0 and .<=1000) or (.=999)*	
chc_j_26 *(required)*	Preterm babies born (<37 weeks)<br>*Response constrained to: (.>=0 and .<=1000) or (.=999)*	
chc_j_27 *(required)*	Early neonatal deaths (within 24 hours of birth)<br>*Response constrained to: (.>=0 and .<=1000) or (.=999)*	
CHC > chc_j_group > Other high-risk pregnancies identified during ANC-<br>*Group relevant when: ${chc_j_3} != 0 and ${chc_j_3} != 999*		
chc_j_13_note	Please mention the Number of Other high-risk pregnancies identified during ANC- (Last 12 Month Data)	
chc_j_13_1 *(required)*	Pregnant women with multiple pregnancy<br>*Response constrained to: (.>=0 and .<=1000) or (.=999)*	
chc_j_13_2 *(required)*	Pregnant women with previous bad obstetric history (BOH)*<br>*Response constrained to: (.>=0 and .<=1000) or (.=999)*	
chc_j_13_3 *(required)*	Pregnant women with other infections (Hepatitis, Malaria, etc.)<br>*Response constrained to: (.>=0 and .<=1000) or (.=999)*	
chc_j_13_4 *(required)*	Pregnant women with other medical conditions (Asthma, COPD, heart disease, etc.)<br>*Response constrained to: (.>=0 and .<=1000) or (.=999)*	
CHC > chc_j_group > Postnatal Women (0-6 Months) & 0-24 Months Infants & Child<br>*Group relevant when: ${chc_j_4} != 0 and ${chc_j_4} != 999*		
chc_j_28_note	Please mention the Number of Postnatal Women (0-6 Months) & 0-24 Months Infants & Child (Last 12 Month Data)	
chc_j_28_1 *(required)*	Newborns screened for birth defects (as per RBSK)<br>*Response constrained to: (.>=0 and .<=1000) or (.=999)*	
chc_j_28_2 *(required)*	Women received postpartum checkup between 48 hours and 14 days after institutional delivery (Facility/Community)<br>*Response constrained to: (.>=0 and .<=1000) or (.=999)*	
chc_j_28_3 *(required)*	HBNC visits performed as per guidelines<br>*Response constrained to: (.>=0 and .<=1000) or (.=999)*	
chc_j_28_4 *(required)*	Postnatal mothers consumed nutritional supplements as per recommendation<br>*Response constrained to: (.>=0 and .<=1000) or (.=999)*	
chc_j_28_5 *(required)*	Postnatal mothers screened for depressive symptoms<br>*Response constrained to: (.>=0 and .<=1000) or (.=999)*	
chc_j_28_6 *(required)*	Postnatal mothers who received appropriate psychosocial support<br>*Response constrained to: (.>=0 and .<=1000) or (.=999)*	
chc_j_28_7 *(required)*	Postnatal mothers who counselled for WASH intervention<br>*Response constrained to: (.>=0 and .<=1000) or (.=999)*	
chc_j_28_8 *(required)*	Postnatal mothers who were given IFA as per recommendation<br>*Response constrained to: (.>=0 and .<=1000) or (.=999)*	
chc_j_28_9 *(required)*	Postnatal mothers who counselled for Nutrition<br>*Response constrained to: (.>=0 and .<=1000) or (.=999)*	
chc_j_28_10 *(required)*	0-6 months Infants received kangaroo mother care (KMC) as applicable (Facility/Home)	

Field	Question	Answer
	Response constrained to: (.>=0 and .<=1000) or (.=999)	
chc_j_28_11 *(required)*	0-6 months Infants exclusively breastfed (EBF)<br>*Response constrained to: (.>=0 and .<=1000) or (.=999)*	
chc_j_28_12 *(required)*	0-6 months Infants (VLBW and LBW) who consumed Iron, as per recommendation<br>*Response constrained to: (.>=0 and .<=1000) or (.=999)*	
chc_j_28_13 *(required)*	0-6 months Infants who consumed Vitamin D 400 IU daily<br>*Response constrained to: (.>=0 and .<=1000) or (.=999)*	
chc_j_28_14 *(required)*	0-6 months Infants (Pre-term) who consumed calcium and vitamin D, as per recommendation<br>*Response constrained to: (.>=0 and .<=1000) or (.=999)*	
chc_j_28_15 *(required)*	6-24 months infants & children who consumed IFA, as per recommendation<br>*Response constrained to: (.>=0 and .<=1000) or (.=999)*	
chc_j_28_16 *(required)*	Infants 6-12 months with inadequate weight gain (<25th centile according to WHO weight velocity/month)<br>*Response constrained to: (.>=0 and .<=1000) or (.=999)*	
chc_j_28_17 *(required)*	Children 12-24 months with inadequate weight gain (<25th centile according to WHO weight velocity/month)<br>*Response constrained to: (.>=0 and .<=1000) or (.=999)*	
chc_j_28_18 *(required)*	Children (0-24 months) with adequate weight gain from birth till 2 years of age<br>*Response constrained to: (.>=0 and .<=1000) or (.=999)*	
chc_j_28_19 *(required)*	Family/Parents having 0-24 months infants & child counselled for WASH interventions<br>*Response constrained to: (.>=0 and .<=1000) or (.=999)*	
chc_j_28_20 *(required)*	Children (12-23 months) fully immunized<br>*Response constrained to: (.>=0 and .<=1000) or (.=999)*	
chc_j_28_21	Observational Remarks	
chc_j_28_22	Respondent Remarks	
CHC > photo_group		
image1	Document 1	
image2	Document 2	
image3	Document 3	
image4	Document 4	
image5	Document 5	
image6	Document 6	
image7	Document 7	
image8	Document 8	
file_pdf	Please upload PDF Documents	

Hospital Assessment form

<table>
<tr><th>Field</th><th>Question</th><th>Answer</th></tr>
<tr><td>worker</td><td>Worker Name</td><td>151 Abuhamza
152 Anmol Saini
153 Anshika Sahota
154 Ekta
155 Jyoti Devi
156 Kritika Thakur
157 Mehak Thakur
158 Poonam Devi
159 Riya Puri
160 Shivanshi
161 Varsha Kumari
162 Anchal Walia
163 Harshali
164 Kritika Puri</td></tr>
<tr><td>blocks (required)</td><td>Block Names</td><td>block_1 Amb
block_2 Basdehra
block_3 Gagret
block_4 Haroli
block_5 Thanakalan</td></tr>
<tr><td>civil_hospital (required)</td><td>Civil hospital</td><td>ch_1 CH Amb
ch_2 CH Chintpurni
ch_3 CH Cum ESI Gagret
ch_4 CH Haroli
ch_5 CH Bangana
ch_999_1 None
ch_999_2 None
ch_999_3 None
ch_999_4 None
ch_999_5 None</td></tr>
<tr><td colspan="3">Hospital</td></tr>
<tr><td colspan="3">Hospital > Hospital Identification [hosp_label]</td></tr>
<tr><td>hosp_a_1</td><td>Date of Assessment:</td><td></td></tr>
<tr><td>hosp_a_6 (required)</td><td>Facility id:</td><td></td></tr>
<tr><td>hosp_a_8 (required)</td><td>Facility Address with landmark</td><td></td></tr>
<tr><td>hosp_a_10 (required)</td><td>Total Population covered by the facility</td><td></td></tr>
<tr><td>hosp_a_11 (required)</td><td>Facility In-charge Name:
Response constrained to: not(regex(.,'^(.*)\d(.*)$'))</td><td></td></tr>
<tr><td>hosp_a_12 (required)</td><td>Facility In-charge Designation:</td><td></td></tr>
<tr><td>hosp_a_13 (required)</td><td>Facility In-charge Contact No.:
Response constrained to: regex(.,"^[6|7|8|9]\d{9}$")</td><td></td></tr>
<tr><td>hosp_a_14</td><td>Facility In-charge Email Id.:</td><td></td></tr>
<tr><td>hosp_a_15 (required)</td><td>Any Additional Respondent?</td><td>1 Yes
2 No</td></tr>
<tr><td colspan="3">Hospital > Additional Respondent
Group relevant when: ${hosp_a_15} =1</td></tr>
<tr><td>hosp_a_15_2 (required)</td><td>Additional Respondent Name
Response constrained to: not(regex(.,'^(.*)\d(.*)$'))</td><td></td></tr>
<tr><td>hosp_a_15_3 (required)</td><td>Additional Respondent Designation</td><td></td></tr>
<tr><td colspan="3">Hospital > Hospital Infrastructure</td></tr>
<tr><td>hosp_b_1 (required)</td><td>Located within Main Habitation (*where the majority of the population resides)
If No, Distance in Km</td><td>1 Yes
2 No</td></tr>
<tr><td>hosp_b_2 (required)</td><td>Connected to Motorable Road</td><td>1 Yes
2 No</td></tr>
<tr><td>hosp_b_3 (required)</td><td>Designated Government Hospital Building</td><td>1 Yes
2 No</td></tr>
<tr><td>hosp_b_4 (required)</td><td>General Structure of Building</td><td>1 Newly Built
2 Newly Renovated
3 Old Structure
4 Falling apart</td></tr>
</table>

Field	Question	Answer
hosp_b_5 *(required)*	Maintenance of Building	1 No maintenance<br>2 Once a year<br>3 Once in 3 years<br>99 Other (specify)
hosp_b_6 *(required)*	Is electrical supply available?	1 24/7<br>3 Average duration of electricity supply per day<br>2 No
hosp_b_6_or	Is power backup available?	1 Generator<br>2 Inverter<br>3 Solar<br>99 Other (Specify)<br>9999 None
hosp_b_7 *(required)*	Main source of water supply?	1 Borewell<br>2 Piped water<br>99 Other with duration
hosp_b_8 *(required)*	Toilet Facility	1 Yes<br>2 No
hosp_b_9 *(required)*	Littering	1 Yes<br>2 No
Hospital > Toilet & Other facilities		
hosp_b_8_1 *(required)*	Facility available inside the toilet<br>*Question relevant when: selected(${hosp_b_8} , '1')*	1 Cleanliness<br>2 Toilet tap with running water<br>3 Flush working properly<br>4 Water bucket and mug inside the toilet<br>5 Washbasin with running water<br>6 Soap/liquid items handwash/hand rub<br>7 Disable friendly<br>9999 None
hosp_b_8_2 *(required)*	Gender specific toilet facility<br>*Question relevant when: selected(${hosp_b_8} , '1')*	1 Both (male & female separate) Available<br>2 Only Male Available<br>3 Only Female Available<br>4 Common Toilet Available<br>5 Availability of a disabled-friendly toilet
hosp_b_10 *(required)*	Availability of general waste bins	1 Green<br>2 Blue<br>3 Both<br>99 Other
hosp_b_11 *(required)*	Management (collection, segregation, storage, transportation, treatment and disposal) of BMW as per Bio-Medical Waste Management Rules, 2018	1 Yes<br>2 No
hosp_b_11_bins *(required)*	Availability of colour coded BMW bins	1 Red<br>2 Yellow<br>3 Blue Puncture proof<br>4 White Puncture proof
Hospital > Availability Status of Human Resource		
hosp_c_2 *(required)*	Sanctioned (No.) MO MBBS	
hosp_c_3 *(required)*	In position (No.) MO MBBS<br>*Question relevant when: ${hosp_c_2} > 0*<br>*Response constrained to: . >= 0 and . <= ${hosp_c_2}*	
Hospital > Availability Status of Human Resource > MO MBBS (1)<br>*Group relevant when: ${hosp_c_2} > 0 and ${hosp_c_3} > 0*		(Repeated group)
hosp_c_4 *(required)*	Status	1 Available<br>2 Long Leave<br>3 Deputed Somewhere Else
Hospital > Availability Status of Human Resource > Training Status Of MO MBBS (1)		(Repeated group)

Field	Question	Answer
Hospital > Availability Status of Human Resource > Training Status Of MO MBBS (1) > hosp_mo_training		
generated_table_list_label_64	Training Status Of MO MBBS	
reserved_name_for_field_list_labels_65		1 Yes<br>2 No
hosp_c_1_1 *(required)*	IUCD Training	1 Yes<br>2 No
hosp_c_1_2 *(required)*	Breastfeeding Training	1 Yes<br>2 No
hosp_c_1_3 *(required)*	Routine Immunization	1 Yes<br>2 No
hosp_c_1_4 *(required)*	Emergency medicine	1 Yes<br>2 No
hosp_c_1_5 *(required)*	NSSK	1 Yes<br>2 No
hosp_c_1_6 *(required)*	Neonatal care Training	1 Yes<br>2 No
hosp_c_1_7 *(required)*	SAANS Training (Under 5 Pneumonia management)	1 Yes<br>2 No
hosp_c_1_8 *(required)*	National Health Program Training	1 Yes<br>2 No
hosp_c_1_9 *(required)*	Anemia Mukt Bharat	1 Yes<br>2 No
hosp_c_1_10 *(required)*	Infection Prevention and Control	1 Yes<br>2 No
hosp_c_1_11 *(required)*	Other	1 Yes<br>2 No
Hospital > Availability Status of Human Resource > Training Status Of MO MBBS (1) > hosp_mo_training_others<br>*Group relevant when: ${hosp_c_1_11} =1*		
hosp_c_1_12 *(required)*	Please specify Training (others)	
hosp_c_10 *(required)*	Sanctioned (No.) Nursing Officer	
hosp_c_11 *(required)*	In position (No.) Nursing Officer<br>*Question relevant when: ${hosp_c_10} > 0*	
Hospital > Availability Status of Human Resource > Nursing Officer (1)<br>*Group relevant when: ${hosp_c_10} > 0 and ${hosp_c_11} > 0*		(Repeated group)
hosp_c_12 *(required)*	Status	1 Available<br>2 Long Leave<br>3 Deputed Somewhere Else
Hospital > Availability Status of Human Resource > Training Status Of Nursing Officer (1)		(Repeated group)
Hospital > Availability Status of Human Resource > Training Status Of Nursing Officer (1) > hosp_nurse_training		
generated_table_list_label_87	Training Status Of Nursing Officer	
reserved_name_for_field_list_labels_88		1 Yes<br>2 No
hosp_c_9_1 *(required)*	SBA	1 Yes<br>2 No
hosp_c_9_2 *(required)*	NSSK	1 Yes<br>2 No
hosp_c_9_3 *(required)*	IUCD	1 Yes<br>2 No
hosp_c_9_4 *(required)*	Routine Immunization (Birth Dose)	1 Yes<br>2 No
hosp_c_9_5 *(required)*	Cold chain management Training	1 Yes<br>2 No
hosp_c_9_6 *(required)*	SAANS Training	1 Yes<br>2 No
hosp_c_9_7 *(required)*	F-IMNCI	1 Yes<br>2 No
hosp_c_9_8 *(required)*	Other	1 Yes<br>2 No
Hospital > Availability Status of Human Resource > Training Status Of Nursing Officer (1) > hosp_nurse_training_others		

Field	Question	Answer
Group relevant when: ${hosp_c_9_9} =1<br>hosp_c_9_9 *(required)*	Please specify Training (others)	
hosp_c_14 *(required)*	Sanctioned (No.) Pharmacist	
hosp_c_15 *(required)*	In position (No.) Pharmacist<br>*Question relevant when: ${hosp_c_14} > 0*<br>*Response constrained to: . >= 0 and . <= ${hosp_c_14}*	
Hospital > Availability Status of Human Resource > Pharmacist (1)<br>*Group relevant when: ${hosp_c_14} > 0 and ${hosp_c_15} > 0*		(Repeated group)
hosp_c_16 *(required)*	Status	1 Available<br>2 Long Leave<br>3 Deputed Somewhere Else
Hospital > Availability Status of Human Resource > Training Status Of Pharmacist (1)		(Repeated group)
Hospital > Availability Status of Human Resource > Training Status Of Pharmacist (1) > hosp_pharmacist_training		
generated_table_list_label_107	Training Status Of Pharmacist	
reserved_name_for_field_list_labels_108		1 Yes<br>2 No
hosp_c_13_1 *(required)*	DVDMS Training	1 Yes<br>2 No
hosp_c_13_2 *(required)*	Stock management	1 Yes<br>2 No
hosp_c_13_3 *(required)*	Cold chain management	1 Yes<br>2 No
hosp_c_13_4 *(required)*	Tuberculosis (TB) Training	1 Yes<br>2 No
hosp_c_13_5 *(required)*	BMW Management	1 Yes<br>2 No
hosp_c_13_6 *(required)*	Sanitation Training	1 Yes<br>2 No
hosp_c_13_7 *(required)*	E-Sanjeevni	1 Yes<br>2 No
hosp_c_13_8 *(required)*	Other	1 Yes<br>2 No
Hospital > Availability Status of Human Resource > Training Status Of Pharmacist (1) > hosp_pharmacist_training_others<br>*Group relevant when: ${hosp_c_13_8} =1*		
hosp_c_13_9 *(required)*	Please specify Training (others)	
hosp_c_18 *(required)*	Sanctioned (No.) Storekeeper	
hosp_c_19 *(required)*	In position (No.) Storekeeper<br>*Question relevant when: ${hosp_c_18} > 0*<br>*Response constrained to: . >= 0 and . <= ${hosp_c_18}*	
Hospital > Availability Status of Human Resource > Storekeeper (1)<br>*Group relevant when: ${hosp_c_18} > 0 and ${hosp_c_19} > 0*		(Repeated group)
hosp_c_20 *(required)*	Status	1 Available<br>2 Long Leave<br>3 Deputed Somewhere Else
Hospital > Availability Status of Human Resource > Training Status Of Storekeeper (1)		(Repeated group)
Hospital > Availability Status of Human Resource > Training Status Of Storekeeper (1) > hosp_storekeep_training		
generated_table_list_label_127	Training Status Of Storekeeper	
reserved_name_for_field_list_labels_128		1 Yes<br>2 No
hosp_c_17_1 *(required)*	DVDMS Training	1 Yes<br>2 No
hosp_c_17_2 *(required)*	Others	1 Yes<br>2 No
Hospital > Availability Status of Human Resource > Training Status Of Storekeeper (1) > hosp_storekeep_training_others<br>*Group relevant when: ${hosp_c_17_2} =1*		
hosp_c_17_8 *(required)*	Please specify Training (others)	
hosp_c_22 *(required)*	Sanctioned (No.) Lab Technician	
hosp_c_23 *(required)*	In position (No.) Lab Technician<br>*Question relevant when: ${hosp_c_22} > 0*<br>*Response constrained to: . >= 0 and . <= ${hosp_c_22}*	
Hospital > Availability Status of Human Resource > Lab Technician (1)		(Repeated group)

Field	Question	Answer
Group relevant when: ${hosp_c_22} > 0 and ${hosp_c_23} > 0		
hosp_c_24 *(required)*	Status	1 Available<br>2 Long Leave<br>3 Deputed Somewhere Else
Hospital > Availability Status of Human Resource > Training Status Of Lab Technician (1)		(Repeated group)
Hospital > Availability Status of Human Resource > Training Status Of Lab Technician (1) > hosp_labtech_training		
generated_table_list_label_141	Training Status Of Lab Technician	
reserved_name_for_field_list_labels_142		1 Yes<br>2 No
hosp_c_21_1 *(required)*	NTEP Training	1 Yes<br>2 No
hosp_c_21_2 *(required)*	Malaria Training	1 Yes<br>2 No
hosp_c_21_3 *(required)*	Use of Rapid Test Kits	1 Yes<br>2 No
hosp_c_21_4 *(required)*	Others	1 Yes<br>2 No
Hospital > Availability Status of Human Resource > Training Status Of Lab Technician (1) > hosp_labtech_training_others<br>*Group relevant when: ${hosp_c_21_4} =1*		
hosp_c_21_5 *(required)*	Please specify Training (others)	
hosp_c_26 *(required)*	Sanctioned (No.) Health Worker (Female)/ANM	
hosp_c_27 *(required)*	In position (No.) Health Worker (Female)/ANM<br>*Question relevant when: ${hosp_c_26} > 0*<br>*Response constrained to: . >= 0 and . <= ${hosp_c_26}*	
Hospital > Availability Status of Human Resource > Health Worker (Female)/ANM (1)<br>*Group relevant when: ${hosp_c_26} > 0 and ${hosp_c_27} > 0*		(Repeated group)
hosp_c_28 *(required)*	Status	1 Available<br>2 Long Leave<br>3 Deputed Somewhere Else
Hospital > Availability Status of Human Resource > Training Status Of Health worker (Female)/ANM (1)		(Repeated group)
Hospital > Availability Status of Human Resource > Training Status Of Health worker (Female)/ANM (1) > hosp_hwf_training		
generated_table_list_label_157	Training Status Of Health worker (Female)/ANM	
reserved_name_for_field_list_labels_158		1 Yes<br>2 No
hosp_c_25_1 *(required)*	Routine immunization	1 Yes<br>2 No
hosp_c_25_2 *(required)*	SAANS Training (Under 5 Pneumonia management)	1 Yes<br>2 No
hosp_c_25_3 *(required)*	HBNC Training	1 Yes<br>2 No
hosp_c_25_4 *(required)*	Diarrhoea management Training (IDCF)	1 Yes<br>2 No
hosp_c_25_5 *(required)*	MAA program	1 Yes<br>2 No
hosp_c_25_6 *(required)*	IUCD	1 Yes<br>2 No
hosp_c_25_7 *(required)*	NCD	1 Yes<br>2 No
hosp_c_25_8 *(required)*	VHND	1 Yes<br>2 No
hosp_c_25_9 *(required)*	VHSNC	1 Yes<br>2 No
hosp_c_25_10 *(required)*	Eat right tool kit	1 Yes<br>2 No
hosp_c_25_11 *(required)*	Other	1 Yes<br>2 No
Hospital > Availability Status of Human Resource > Training Status Of Health worker (Female)/ANM (1) > hosp_hwf_training_others<br>*Group relevant when: ${hosp_c_25_11} =1*		
hosp_c_25_12 *(required)*	Please specify Training (others)	
hosp_c_30 *(required)*	Sanctioned (No.) Health Worker (Male)/ Health Assistant (Male)	

Field	Question	Answer
hosp_c_31 *(required)*	In position (No.) Health Worker (Male)/ Health Assistant (Male)<br>*Question relevant when: ${hosp_c_30} > 0*<br>*Response constrained to: . >= 0 and . <= ${hosp_c_30}*	
Hospital > Availability Status of Human Resource > Health Worker (Male)/ Health Assistant (Male) (1)<br>*Group relevant when: ${hosp_c_30} > 0 and ${hosp_c_31} > 0*		(Repeated group)
hosp_c_32 *(required)*	Status	1 Available<br>2 Long Leave<br>3 Deputed Somewhere Else
Hospital > Availability Status of Human Resource > Training Status Of Health Worker (Male)/Health Assistant (Male) (1)		(Repeated group)
Hospital > Availability Status of Human Resource > Training Status Of Health Worker (Male)/Health Assistant (Male) (1) > hosp_hwm_training		
generated_table_list_label_180	Training Status Of Health Worker (Male)/Health Assistant (Male)	
reserved_name_for_field_list_labels_181		1 Yes<br>2 No
hosp_c_29_1 *(required)*	TB	1 Yes<br>2 No
hosp_c_29_2 *(required)*	Malaria	1 Yes<br>2 No
hosp_c_29_3 *(required)*	Routine Immunization	1 Yes<br>2 No
hosp_c_29_4 *(required)*	Water testing	1 Yes<br>2 No
hosp_c_29_5 *(required)*	Other	1 Yes<br>2 No
Hospital > Availability Status of Human Resource > Training Status Of Health Worker (Male)/Health Assistant (Male) (1) > hosp_hwm_training_others<br>*Group relevant when: ${hosp_c_29_5} =1*		
hosp_c_29_6 *(required)*	Please specify Training (others)	
hosp_c_34 *(required)*	Sanctioned (No.) Female health supervisor	
hosp_c_35 *(required)*	In position (No.) Female health supervisor<br>*Question relevant when: ${hosp_c_34} > 0*<br>*Response constrained to: . >= 0 and . <= ${hosp_c_34}*	
Hospital > Availability Status of Human Resource > Health Assistant Female health supervisor (1)<br>*Group relevant when: ${hosp_c_34} > 0 and ${hosp_c_35} > 0*		(Repeated group)
hosp_c_36 *(required)*	Status	1 Available<br>2 Long Leave<br>3 Deputed Somewhere Else
Hospital > Availability Status of Human Resource > Health Assistant Female health supervisor (1)		(Repeated group)
Hospital > Availability Status of Human Resource > Health Assistant Female health supervisor (1) > hosp_lhv_training		
generated_table_list_label_197	Health Assistant Female health supervisor	
reserved_name_for_field_list_labels_198		1 Yes<br>2 No
hosp_c_33_1 *(required)*	Cold chain management	1 Yes<br>2 No
hosp_c_33_2 *(required)*	Others	1 Yes<br>2 No
Hospital > Availability Status of Human Resource > Health Assistant Female health supervisor (1) > hosp_lhv_training_others<br>*Group relevant when: ${hosp_c_33_3} =1*		
hosp_c_33_3 *(required)*	Please specify Training (others)	
hosp_c_38 *(required)*	Sanctioned (No.) Health Educator/ Counsellor	
hosp_c_39 *(required)*	In position (No.) Health Educator/ Counsellor<br>*Question relevant when: ${hosp_c_38} > 0*<br>*Response constrained to: . >= 0 and . <= ${hosp_c_38}*	
Hospital > Availability Status of Human Resource > Health Educator/ Counsellor (1)<br>*Group relevant when: ${hosp_c_38} > 0 and ${hosp_c_39} > 0*		(Repeated group)
hosp_c_40 *(required)*	Status	1 Available<br>2 Long Leave<br>3 Deputed Somewhere Else
Hospital > Availability Status of Human Resource > Health Educator/ Counsellor (1)		(Repeated group)
Hospital > Availability Status of Human Resource > Health Educator/ Counsellor (1) > hosp_hec_training		
generated_table_list_label_211	Health Educator/ Counsellor	

Field	Question	Answer
reserved_name_for_field_list_labels_212		1 Yes<br>2 No
hosp_c_37_1 *(required)*	HBNC	1 Yes<br>2 No
hosp_c_37_2 *(required)*	HBYC	1 Yes<br>2 No
hosp_c_37_3 *(required)*	VHND	1 Yes<br>2 No
hosp_c_37_4 *(required)*	HIV	1 Yes<br>2 No
hosp_c_37_5 *(required)*	Routine Immunization	1 Yes<br>2 No
hosp_c_37_6 *(required)*	Adolescent Health	1 Yes<br>2 No
hosp_c_37_7 *(required)*	Maternal health	1 Yes<br>2 No
hosp_c_37_8 *(required)*	Anemia Mukt Bharat	1 Yes<br>2 No
hosp_c_37_9 *(required)*	Under 5 Pneumonia Management Program	1 Yes<br>2 No
hosp_c_37_10 *(required)*	Intensive Diarrhoea Control Fortnight (IDCF)	1 Yes<br>2 No
hosp_c_37_11 *(required)*	MAA program	1 Yes<br>2 No
hosp_c_37_12 *(required)*	IEC Planning & Implementation Training	1 Yes<br>2 No
hosp_c_37_13 *(required)*	Other	1 Yes<br>2 No
Hospital > Availability Status of Human Resource > Health Educator/ Counsellor (1) > hosp_hec_training_others<br>*Group relevant when: ${hosp_c_37_13} =1*		
hosp_c_37_14 *(required)*	Please specify Training (others)	
hosp_c_42 *(required)*	Sanctioned (No.) Data Entry Operator	
hosp_c_43 *(required)*	In position (No.) Data Entry Operator<br>*Question relevant when: ${hosp_c_42} > 0*<br>*Response constrained to: . >= 0 and . <= ${hosp_c_42}*	
Hospital > Availability Status of Human Resource > Data Entry Operator (1)<br>*Group relevant when: ${hosp_c_42} > 0 and ${hosp_c_43} > 0*		(Repeated group)
hosp_c_44 *(required)*	Status	1 Available<br>2 Long Leave<br>3 Deputed Somewhere Else
hosp_c_46 *(required)*	Sanctioned (No.) Obstetrician	
hosp_c_47 *(required)*	In position (No.) Obstetrician<br>*Question relevant when: ${hosp_c_46} > 0*<br>*Response constrained to: . >= 0 and . <= ${hosp_c_46}*	
Hospital > Availability Status of Human Resource > Obstetrician (1)<br>*Group relevant when: ${hosp_c_46} > 0 and ${hosp_c_47} > 0*		(Repeated group)
hosp_c_48 *(required)*	Status	1 Available<br>2 Long Leave<br>3 Deputed Somewhere Else
hosp_c_50 *(required)*	Sanctioned (No.) Pediatrician	
hosp_c_51 *(required)*	In position (No.) Pediatrician<br>*Question relevant when: ${hosp_c_50} > 0*<br>*Response constrained to: . >= 0 and . <= ${hosp_c_50}*	
Hospital > Availability Status of Human Resource > Pediatrician (1)<br>*Group relevant when: ${hosp_c_50} > 0 and ${hosp_c_51} > 0*		(Repeated group)
hosp_c_52 *(required)*	Status	1 Available<br>2 Long Leave<br>3 Deputed Somewhere Else
hosp_c_54 *(required)*	Sanctioned (No.) Anesthetist	
hosp_c_55 *(required)*	In position (No.) Anesthetist	

Field	Question	Answer
	Question relevant when: ${hosp_c_54} > 0<br>*Response constrained to: . >= 0 and . <= ${hosp_c_54}*	
Hospital > Availability Status of Human Resource > Anesthetist (1)<br>*Group relevant when: ${hosp_c_54} > 0 and ${hosp_c_55} > 0*		(Repeated group)
hosp_c_56 *(required)*	Status	1 Available<br>2 Long Leave<br>3 Deputed Somewhere Else
hosp_c_58 *(required)*	Sanctioned (No.) Radiologist	
hosp_c_59 *(required)*	In position (No.) Radiologist<br>*Question relevant when: ${hosp_c_58} > 0*<br>*Response constrained to: . >= 0 and . <= ${hosp_c_58}*	
Hospital > Availability Status of Human Resource > Radiologist (1)<br>*Group relevant when: ${hosp_c_58} > 0 and ${hosp_c_59} > 0*		(Repeated group)
hosp_c_60 *(required)*	Status	1 Available<br>2 Long Leave<br>3 Deputed Somewhere Else
hosp_c_62 *(required)*	Sanctioned (No.) Psychiatrist	
hosp_c_63 *(required)*	In position (No.) Psychiatrist<br>*Question relevant when: ${hosp_c_62} > 0*<br>*Response constrained to: . >= 0 and . <= ${hosp_c_62}*	
Hospital > Availability Status of Human Resource > Psychiatrist (1)<br>*Group relevant when: ${hosp_c_62} > 0 and ${hosp_c_63} > 0*		(Repeated group)
hosp_c_64 *(required)*	Status	1 Available<br>2 Long Leave<br>3 Deputed Somewhere Else
hosp_c_66 *(required)*	Sanctioned (No.) Psychologist	
hosp_c_67 *(required)*	In position (No.) Psychologist<br>*Question relevant when: ${hosp_c_66} > 0*<br>*Response constrained to: . >= 0 and . <= ${hosp_c_66}*	
Hospital > Availability Status of Human Resource > Psychologist (1)<br>*Group relevant when: ${hosp_c_66} > 0 and ${hosp_c_67} > 0*		(Repeated group)
hosp_c_68 *(required)*	Status	1 Available<br>2 Long Leave<br>3 Deputed Somewhere Else
hosp_c_70 *(required)*	Sanctioned (No.) Lactation counsellor	
hosp_c_71 *(required)*	In position (No.) Lactation counsellor<br>*Question relevant when: ${hosp_c_70} > 0*<br>*Response constrained to: . >= 0 and . <= ${hosp_c_70}*	
Hospital > Availability Status of Human Resource > Lactation counsellor (1)<br>*Group relevant when: ${hosp_c_70} > 0 and ${hosp_c_71} > 0*		(Repeated group)
hosp_c_72 *(required)*	Status	1 Available<br>2 Long Leave<br>3 Deputed Somewhere Else
hosp_c_73 *(required)*	Any other staff?	1 Yes<br>2 No
Hospital > Availability Status of Human Resource > anyother_group<br>*Group relevant when: ${hosp_c_73} =1*		
any_other1	Name<br>*Response constrained to: not(regex(.,'^(.*)\d(.*)$'))*	
any_other2	Designation<br>*Response constrained to: not(regex(.,'^(.*)\d(.*)$'))*	
Hospital > hosp_infra2		
generated_table_list_label_276	Quality Assurance & Others	
hosp_b_12	Quality assurance certification/services	
reserved_name_for_field_list_labels_278		1 Yes<br>2 No
hosp_b_13 *(required)*	Kayakalp	1 Yes<br>2 No
hosp_b_14 *(required)*	NQAS	1 Yes<br>2 No
hosp_b_16 *(required)*	SUMAN	1 Yes

Field	Question	Answer
		2 No
Hospital > hosp_infra3		
hosp_b_17 *(required)*	Emergency area and Triage services available	1 Yes<br>2 No<br>9999 Not Applicable
hosp_b_17_or	Available 24x7?	1 Yes<br>2 No
hosp_b_18 *(required)*	Gynec/Obstetrics OPD Services	1 Yes<br>2 No
hosp_b_20 *(required)*	Laboratory Services	1 Yes<br>2 No
hosp_b_21 *(required)*	Pediatrics OPD Room Services	1 Yes<br>2 No
hosp_b_23 *(required)*	Psychologist OPD Room Services	1 Yes<br>2 No
hosp_b_25 *(required)*	Lactation Counsellor Room Services	1 Yes<br>2 No
hosp_b_27 *(required)*	Dietitian/ Nutrition Counsellor Room Services	1 Yes<br>2 No
hosp_b_30 *(required)*	ANC Room Services	1 Yes<br>2 No
hosp_b_32 *(required)*	Labour Room Services	1 Yes<br>2 No
Hospital > hosp_infra_room		
hosp_b_19 *(required)*	Gynec/Obstetrics OPD services OPD services - Available failities<br>*Question relevant when: ${hosp_b_18} =1*<br>*Response constrained to: not(selected(${hosp_b_19} , '9999') and count-selected(${hosp_b_19}) > 1)*	1 Adequate table, chairs, bench and stool<br>5 Footstep<br>3 Handwashing Station with Running Water Supply<br>4 Waiting area with sitting facility<br>9999 None
hosp_b_22 *(required)*	Pediatrics OPD Room Services - Available facilities<br>*Question relevant when: ${hosp_b_21} =1*<br>*Response constrained to: not(selected(${hosp_b_22} , '9999') and count-selected(${hosp_b_22}) > 1)*	1 Adequate table, chairs, bench and stool<br>5 Footstep<br>3 Handwashing Station with Running Water Supply<br>4 Waiting area with sitting facility<br>9999 None
hosp_b_24 *(required)*	Psychologist OPD Room Services - Available Facilities<br>*Question relevant when: ${hosp_b_23} =1*<br>*Response constrained to: not(selected(${hosp_b_24} , '9999') and count-selected(${hosp_b_24}) > 1)*	1 Adequate table, chairs, bench and stool<br>2 Examination table<br>3 Footstep<br>4 Handwashing Station with Running Water Supply<br>5 Waiting area with sitting facility<br>9999 None
hosp_b_26 *(required)*	Lactation Counsellor Room Services - Available Facilities<br>*Question relevant when: ${hosp_b_25} =1*<br>*Response constrained to: not(selected(${hosp_b_26} , '9999') and count-selected(${hosp_b_26}) > 1)*	1 Adequate table, chairs, bench and stool<br>2 Examination table<br>3 Footstep<br>4 Handwashing Station with Running Water Supply<br>5 Waiting area with sitting facility<br>9999 None

Field	Question	Answer
hosp_b_28 *(required)*	Dietitian/ Nutrition Counsellor Room Services - Available Facilities<br>*Question relevant when: ${hosp_b_27} =1*<br>*Response constrained to: not(selected(${hosp_b_28} , '9999') and count-selected(${hosp_b_28}) > 1)*	1 Adequate table, chairs, bench and stool<br>2 Examination table<br>3 Footstep<br>4 Handwashing Station with Running Water Supply<br>5 Waiting area with sitting facility<br>9999 None
hosp_b_29 *(required)*	Laboratory Services - Available failities<br>*Question relevant when: ${hosp_b_20} =1*<br>*Response constrained to: not(selected(${hosp_b_29} , '9999') and count-selected(${hosp_b_29}) > 1)*	1 Adequate table, chairs, bench and stool<br>2 Washbasin with Running Water Supply<br>3 Adequate area for sample collection area<br>4 Adequate areas for carrying out diagnostic activities<br>5 Adequate area for keeping equipment and storage of reagents and records<br>9999 None of the above
hosp_b_31 *(required)*	ANC Room Services - Available failities<br>*Question relevant when: ${hosp_b_30} =1*<br>*Response constrained to: not(selected(${hosp_b_31} , '9999') and count-selected(${hosp_b_31}) > 1)*	1 Adequate table, chairs, bench and stool<br>2 Examination table<br>3 Footstep<br>4 Handwashing Station with Running Water Supply<br>5 Waiting area with sitting facility<br>9999 None
hosp_b_33 *(required)*	Labour Room Services - Available failities<br>*Question relevant when: ${hosp_b_32} =1*<br>*Response constrained to: not(selected(${hosp_b_33} , '9999') and count-selected(${hosp_b_33}) > 1)*	1 Adequate labour table<br>2 Washbasin with Running Water Supply<br>3 Area embarked for Newborn care corner<br>4 Elbow tap<br>9999 None
Hospital > Labour Room Infrastructure Status		
hosp_b_34 *(required)*	Post natal care area and beds<br>*If Yes, total number of beds available*	1 Yes<br>2 No
hosp_b_35 *(required)*	Pediatric ward<br>*If Yes, total number of beds available*	1 Yes<br>2 No
hosp_b_36 *(required)*	Special Newborn Care Unit (SNCU)	1 Yes<br>2 No
hosp_b_37 *(required)*	Neonatal Intensive Care Unit (NICU)	1 Yes<br>2 No
hosp_b_38 *(required)*	Gynae/Obstetrics ward<br>*If Yes, total number of beds available*	1 Yes<br>2 No
Hospital > Nutrition Rehabilitation Center (NRC)		
hosp_b_44 *(required)*	Nutrition Rehabilitation Center (NRC) : Available Facilities<br>*Response constrained to: not(selected(${hosp_b_44} , '9999') and count-selected(${hosp_b_44}) > 1)*	1 Play Area<br>2 Couselling Area<br>3 Nursing Station<br>4 Kitchen with Cooking Demonstration Aids<br>9999 None
hosp_b_45 *(required)*	Blood Bank / Blood Storage Units	1 Yes<br>2 No
Hospital > Availability Status of Equipment at Hospital		
hosp_d_1 *(required)*	24x7 Emergency Services with Triage, Resuscitation & Stabilization	1 Yes<br>2 No
Hospital > Availability Status of Equipment at Hospital > hosp_triage		

Field	Question	Answer
Group relevant when: ${hosp_d_1} =1		
hosp_d_2 *(required)*	Emergency Drug Tray<br>*If No, specify missing medicines*	1 Yes<br>2 No
hosp_d_3 *(required)*	Oxygen Cylinder<br>*If yes, please specify number of functional equipments*	1 Yes<br>2 No
hosp_d_171 *(required)*	O2 concentrator<br>*If yes, please specify number of functional equipments*	1 Yes<br>2 No
hosp_d_4 *(required)*	Suction Machine (Electrical)<br>*If yes, please specify number of functional equipments*	1 Yes<br>2 No
hosp_d_4_1 *(required)*	Suction Machine (Foot-operated)<br>*If yes, please specify number of functional equipments*	1 Yes<br>2 No
hosp_d_5 *(required)*	Resuscitation equipment for adult<br>*If No, specify missing medicines*	1 Yes<br>2 No
hosp_d_7 *(required)*	Open care/Radiant Warmer<br>*If yes, please specify number of functional equipments*	1 Yes<br>2 No
hosp_d_8 *(required)*	Pulse Oximeter Adult<br>*If yes, please specify number of functional equipments*	1 Yes<br>2 No
hosp_d_172 *(required)*	Pulse Oximeter Pediatric<br>*If yes, please specify number of functional equipments*	1 Yes<br>2 No
hosp_d_9 *(required)*	Self-Inflating Bag and Mask Adult<br>*If yes, please specify number of functional equipments*	1 Yes<br>2 No
hosp_d_173 *(required)*	Self-Inflating Bag and Mask Child<br>*If yes, please specify number of functional equipments*	1 Yes<br>2 No
hosp_d_174 *(required)*	Self-Inflating Bag and Mask Neonatal Size(0,1)<br>*If yes, please specify number of functional equipments*	1 Yes<br>2 No
hosp_d_10 *(required)*	Laryngoscope and ET Intubation Tubes all Sizes<br>*If yes, please specify number of functional equipments*	1 Yes<br>2 No
hosp_d_11 *(required)*	Defibrillators<br>*If yes, please specify number of functional equipments*	1 Yes<br>2 No
hosp_d_12 *(required)*	ECG Machine<br>*If yes, please specify number of functional equipments*	1 Yes<br>2 No
hosp_d_13 *(required)*	ANC Services	1 Yes<br>2 No
Hospital > Availability Status of Equipment at Hospital > ANC Services<br>*Group relevant when: ${hosp_d_13} =1*		
hosp_d_14 *(required)*	Blood Pressure Monitor (Digital)<br>*If yes, please specify number of functional equipments*	1 Yes<br>2 No
hosp_d_14_1 *(required)*	Blood Pressure Monitor (Manual)<br>*If yes, please specify number of functional equipments*	1 Yes<br>2 No
hosp_d_15 *(required)*	Stethoscope<br>*If yes, please specify number of functional equipments*	1 Yes<br>2 No
hosp_d_175 *(required)*	Thermometer (Digital)<br>*If yes, please specify number of functional equipments*	1 Yes<br>2 No
hosp_d_16 *(required)*	Clinical Thermometer<br>*If yes, please specify number of functional equipments*	1 Yes<br>2 No
hosp_d_17 *(required)*	Adult Weighing Scale (Digital)<br>*If yes, please specify number of functional equipments*	1 Yes<br>2 No
hosp_d_17_1 *(required)*	Adult Weighing Scale (Analogue)<br>*If yes, please specify number of functional equipments*	1 Yes<br>2 No
hosp_d_18 *(required)*	Stadiometer<br>*If yes, please specify number of functional equipments*	1 Yes<br>2 No
hosp_d_19 *(required)*	Measuring Tape<br>*If yes, please specify number of functional equipments*	1 Yes<br>2 No
hosp_d_20 *(required)*	Examination Lamp with White Light<br>*If yes, please specify number of functional equipments*	1 Yes<br>2 No
hosp_d_20_1 *(required)*	Fetoscope<br>*If yes, please specify number of functional equipments*	1 Yes<br>2 No
hosp_d_176 *(required)*	Fetal Doppler Monitor<br>*If yes, please specify number of functional equipments*	1 Yes<br>2 No

Field	Question	Answer
hosp_d_20_2 *(required)*	Cusco's Speculum<br>*If yes, please specify number of functional equipments*	1 Yes<br>2 No
hosp_d_177 *(required)*	Sims Speculum<br>*If yes, please specify number of functional equipments*	1 Yes<br>2 No
hosp_d_21 *(required)*	Pulse oximeter (adult)<br>*If yes, please specify number of functional equipments*	1 Yes<br>2 No
hosp_d_22 *(required)*	Gynae/Obstetrics OPD Service	1 Yes<br>2 No
Hospital > Availability Status of Equipment at Hospital > Gynae/Obstetrics OPD Services<br>*Group relevant when: ${hosp_d_22} =1*		
hosp_d_23 *(required)*	Blood Pressure Monitor (Digital)<br>*If yes, please specify number of functional equipments*	1 Yes<br>2 No
hosp_d_23_1 *(required)*	Blood Pressure Monitor (Manual)<br>*If yes, please specify number of functional equipments*	1 Yes<br>2 No
hosp_d_24 *(required)*	Stethoscope<br>*If yes, please specify number of functional equipments*	1 Yes<br>2 No
hosp_d_25 *(required)*	Thermometer<br>*If yes, please specify number of functional equipments*	1 Yes<br>2 No
hosp_d_26 *(required)*	Adult Weighing Scale (Digital)<br>*If yes, please specify number of functional equipments*	1 Yes<br>2 No
hosp_d_26_1 *(required)*	Adult Weighing Scale (Analog)<br>*If yes, please specify number of functional equipments*	1 Yes<br>2 No
hosp_d_27 *(required)*	Stadiometer<br>*If yes, please specify number of functional equipments*	1 Yes<br>2 No
hosp_d_28 *(required)*	Measuring Tape<br>*If yes, please specify number of functional equipments*	1 Yes<br>2 No
hosp_d_29 *(required)*	Examination Lamp with White Light<br>*If yes, please specify number of functional equipments*	1 Yes<br>2 No
hosp_d_30 *(required)*	Fetoscope<br>*If yes, please specify number of functional equipments*	1 Yes<br>2 No
hosp_d_181 *(required)*	Fetal Doppler Monitor<br>*If yes, please specify number of functional equipments*	1 Yes<br>2 No
hosp_d_31 *(required)*	Cusco's Speculum<br>*If yes, please specify number of functional equipments*	1 Yes<br>2 No
hosp_d_182 *(required)*	Sims Speculum<br>*If yes, please specify number of functional equipments*	1 Yes<br>2 No
hosp_d_32 *(required)*	Pulse oximeter (adult)<br>*If yes, please specify number of functional equipments*	1 Yes<br>2 No
hosp_d_33 *(required)*	Pediatric OPD Service	1 Yes<br>2 No
Hospital > Availability Status of Equipment at Hospital > Pediatric OPD Services<br>*Group relevant when: ${hosp_d_33} =1*		
hosp_d_34 *(required)*	Pediatric Stethoscope<br>*If yes, please specify number of functional equipments*	1 Yes<br>2 No
hosp_d_35 *(required)*	Clinical Thermometer<br>*If yes, please specify number of functional equipments*	1 Yes<br>2 No
hosp_d_178 *(required)*	Digital Thermometer<br>*If yes, please specify number of functional equipments*	1 Yes<br>2 No
hosp_d_36 *(required)*	Infantometer<br>*If yes, please specify number of functional equipments*	1 Yes<br>2 No
hosp_d_37 *(required)*	Baby Weighing Scale (Electronic)<br>*If yes, please specify number of functional equipments*	1 Yes<br>2 No
hosp_d_37_1 *(required)*	Baby Weighing Scale (Manual)<br>*If yes, please specify number of functional equipments*	1 Yes<br>2 No
hosp_d_38 *(required)*	Pulse oximeter (Pediatric)<br>*If yes, please specify number of functional equipments*	1 Yes<br>2 No
hosp_d_39 *(required)*	Phototherapy machine<br>*If yes, please specify number of functional equipments*	1 Yes<br>2 No
hosp_d_40 *(required)*	Pediatric Resuscitation Kit	1 Yes

Field	Question	Answer
	If yes, please specify number of functional equipments	2 No
hosp_d_41 *(required)*	Breast pump (Manual)<br>*If yes, please specify number of functional equipments*	1 Yes<br>2 No
hosp_d_41_1 *(required)*	Breast pump (Electric)<br>*If yes, please specify number of functional equipments*	1 Yes<br>2 No
hosp_d_190 *(required)*	Labour Room	1 Yes<br>2 No
Hospital > Availability Status of Equipment at Hospital > Labour Room<br>*Group relevant when: ${hosp_d_190} =1*		
hosp_d_42 *(required)*	Foetal doppler<br>*If yes, please specify number of functional equipments*	1 Yes<br>2 No
hosp_d_43 *(required)*	Cardiotocography (CTG) machine<br>*If yes, please specify number of functional equipments*	1 Yes<br>2 No
hosp_d_44 *(required)*	Laryngoscopes (Led)<br>*If yes, please specify number of functional equipments*	1 Yes<br>2 No
hosp_d_45 *(required)*	Laryngoscopes (Led) Both Straight And Curved for Pediatric and ET tubes<br>*If yes, please specify number of functional equipments*	1 Yes<br>2 No
hosp_d_46 *(required)*	Suction Machine (Electrical)<br>*If yes, please specify number of functional equipments*	1 Yes<br>2 No
hosp_d_179 *(required)*	Suction Machine (foot-operated)<br>*If yes, please specify number of functional equipments*	1 Yes<br>2 No
hosp_d_47 *(required)*	Examination light<br>*If yes, please specify number of functional equipments*	1 Yes<br>2 No
hosp_d_48 *(required)*	Open care radiant warmer<br>*If yes, please specify number of functional equipments*	1 Yes<br>2 No
hosp_d_49 *(required)*	Vacuum extractors<br>*If yes, please specify number of functional equipments*	1 Yes<br>2 No
hosp_d_50 *(required)*	Pulse Oximeter (Adult and Pediatric Probes)<br>*If yes, please specify number of functional equipments*	1 Yes<br>2 No
hosp_d_51 *(required)*	Self-Inflating Bag and Mask Neonatal Size(0,1)<br>*If yes, please specify number of functional equipments*	1 Yes<br>2 No
hosp_d_53 *(required)*	NORMAL DELIVERY TRAY (equipments)<br>*If yes, please specify number of functional equipments*	1 Yes<br>2 No
hosp_d_54 *(required)*	Episiotomy kit<br>*If yes, please specify number of functional equipments*	1 Yes<br>2 No
hosp_d_55 *(required)*	Forceps delivery kit<br>*If yes, please specify number of functional equipments*	1 Yes<br>2 No
hosp_d_56 *(required)*	Craniotomy kit<br>*If yes, please specify number of functional equipments*	1 Yes<br>2 No
hosp_d_57 *(required)*	Crash Cart with emergency medicines<br>*If No, specify missing medicines*	1 Yes<br>2 No
hosp_d_58 *(required)*	Feeding tube<br>*If yes, please specify number of functional equipments*	1 Yes<br>2 No
hosp_d_60 *(required)*	Suction catheter<br>*If yes, please specify number of functional equipments*	1 Yes<br>2 No
hosp_d_62 *(required)*	Cord clamps<br>*If yes, please specify number of functional equipments*	1 Yes<br>2 No
hosp_d_77_1 *(required)*	Special Newborn Care Unit (SNCU)	1 Yes<br>2 No
Hospital > Availability Status of Equipment at Hospital > Special Newborn Care Unit (SNCU)<br>*Group relevant when: ${hosp_d_77_1} =1*		
hosp_d_78 *(required)*	Open care Radiant warmer<br>*If yes, please specify number of functional equipments*	1 Yes<br>2 No
hosp_d_79 *(required)*	Neonatal Resuscitation Equipment<br>*If yes, please specify number of functional equipments*	1 Yes<br>2 No
hosp_d_80 *(required)*	Pulse oximeter with Neonatal Wrap up<br>*If yes, please specify number of functional equipments*	1 Yes<br>2 No
hosp_d_81 *(required)*	Multi para Monitor with Neonatal leads<br>*If yes, please specify number of functional equipments*	1 Yes<br>2 No

Field	Question	Answer
hosp_d_82 *(required)*	Syringe Pump<br>*If yes, please specify number of functional equipments*	1 Yes<br>2 No
hosp_d_83 *(required)*	Phototherapy Single Surface LED<br>*If yes, please specify number of functional equipments*	1 Yes<br>2 No
hosp_d_84 *(required)*	Nebulizer<br>*If yes, please specify number of functional equipments*	1 Yes<br>2 No
hosp_d_85 *(required)*	Portable LED Standing light<br>*If yes, please specify number of functional equipments*	1 Yes<br>2 No
hosp_d_86 *(required)*	Foot Operating Suction machine<br>*If yes, please specify number of functional equipments*	1 Yes<br>2 No
hosp_d_87 *(required)*	BP Apparatus - Neonatal Cuff<br>*If yes, please specify number of functional equipments*	1 Yes<br>2 No
hosp_d_88 *(required)*	Baby weighing scale –Digital<br>*If yes, please specify number of functional equipments*	1 Yes<br>2 No
hosp_d_89 *(required)*	Infantometer<br>*If yes, please specify number of functional equipments*	1 Yes<br>2 No
hosp_d_90 *(required)*	Transcutaneous Bilirubinometer<br>*If yes, please specify number of functional equipments*	1 Yes<br>2 No
hosp_d_91 *(required)*	Bubble CPAP with compressor<br>*If yes, please specify number of functional equipments*	1 Yes<br>2 No
hosp_d_92 *(required)*	Transport Incubator<br>*If yes, please specify number of functional equipments*	1 Yes<br>2 No
hosp_d_93 *(required)*	Irradiance Meter<br>*If yes, please specify number of functional equipments*	1 Yes<br>2 No
hosp_d_94 *(required)*	Vein Finder<br>*If yes, please specify number of functional equipments*	1 Yes<br>2 No
hosp_d_95 *(required)*	Oxygen Hood<br>*If yes, please specify number of functional equipments*	1 Yes<br>2 No
hosp_d_96 *(required)*	Ambu bags (neonatal & paediatrics)<br>*If yes, please specify number of functional equipments*	1 Yes<br>2 No
hosp_d_97 *(required)*	Oxygen Manifold<br>*If yes, please specify number of functional equipments*	1 Yes<br>2 No
hosp_d_97_1 *(required)*	Neonatal Intensive Care Unit (NICU)	1 Yes<br>2 No
Hospital > Availability Status of Equipment at Hospital > Neonatal Intensive Care Unit (NICU)<br>*Group relevant when: ${hosp_d_97_1} =1*		
hosp_d_98 *(required)*	Radiant warmer<br>*If yes, please specify number of functional equipments*	1 Yes<br>2 No
hosp_d_100 *(required)*	Neonatal Resuscitation Equipment<br>*If yes, please specify number of functional equipments*	1 Yes<br>2 No
hosp_d_101 *(required)*	BP Apparatus - Neonatal Cuff<br>*If yes, please specify number of functional equipments*	1 Yes<br>2 No
hosp_d_102 *(required)*	Neonatal Ventilator<br>*If yes, please specify number of functional equipments*	1 Yes<br>2 No
hosp_d_103 *(required)*	Pulse Oximeter With Neonatal Wrap Up<br>*If yes, please specify number of functional equipments*	1 Yes<br>2 No
hosp_d_104 *(required)*	Multi Para Monitor With Leads<br>*If yes, please specify number of functional equipments*	1 Yes<br>2 No
hosp_d_106 *(required)*	Syringe Pump<br>*If yes, please specify number of functional equipments*	1 Yes<br>2 No
hosp_d_107 *(required)*	Phototherapy Single Surface Led<br>*If yes, please specify number of functional equipments*	1 Yes<br>2 No
hosp_d_108 *(required)*	Nebulizer<br>*If yes, please specify number of functional equipments*	1 Yes<br>2 No
hosp_d_109 *(required)*	Portable Led Standing Light<br>*If yes, please specify number of functional equipments*	1 Yes<br>2 No
hosp_d_110 *(required)*	Foot Operating Suction Machine<br>*If yes, please specify number of functional equipments*	1 Yes<br>2 No
hosp_d_111 *(required)*	Infantometer	1 Yes

Field	Question	Answer
	If yes, please specify number of functional equipments	2 No
hosp_d_112 *(required)*	Transcutaneous Bilirubinometer<br>*If yes, please specify number of functional equipments*	1 Yes<br>2 No
hosp_d_113 *(required)*	Bubble Cpap With Compressor<br>*If yes, please specify number of functional equipments*	1 Yes<br>2 No
hosp_d_114 *(required)*	Transport Incubator<br>*If yes, please specify number of functional equipments*	1 Yes<br>2 No
hosp_d_115 *(required)*	Irradiance Meter<br>*If yes, please specify number of functional equipments*	1 Yes<br>2 No
hosp_d_116 *(required)*	Vein Finder<br>*If yes, please specify number of functional equipments*	1 Yes<br>2 No
hosp_d_118 *(required)*	Oxygen Hood<br>*If yes, please specify number of functional equipments*	1 Yes<br>2 No
hosp_d_119 *(required)*	Mechanical Ventilator for paediatric use<br>*If yes, please specify number of functional equipments*	1 Yes<br>2 No
hosp_d_120 *(required)*	Disaster Trolley/Crash Cart with emergency medicines<br>*If yes, please specify number of functional equipments*	1 Yes<br>2 No
hosp_d_121 *(required)*	Ambu Bags (Paediatrics)<br>*If yes, please specify number of functional equipments*	1 Yes<br>2 No
hosp_d_122 *(required)*	Pediatric ward	1 Yes<br>2 No
Hospital > Availability Status of Equipment at Hospital > Pediatric ward<br>*Group relevant when: ${hosp_d_122} =1*		
hosp_d_123 *(required)*	Pediatric Stethoscope<br>*If yes, please specify number of functional equipments*	1 Yes<br>2 No
hosp_d_124 *(required)*	Digital/Infrared Thermometer<br>*If yes, please specify number of functional equipments*	1 Yes<br>2 No
hosp_d_125 *(required)*	Infantometer<br>*If yes, please specify number of functional equipments*	1 Yes<br>2 No
hosp_d_126 *(required)*	Baby Weighing Scale (Electronic)<br>*If yes, please specify number of functional equipments*	1 Yes<br>2 No
hosp_d_126_1 *(required)*	Baby Weighing Scale (Manual)<br>*If yes, please specify number of functional equipments*	1 Yes<br>2 No
hosp_d_127 *(required)*	Pulse oximeter (Pediatric)<br>*If yes, please specify number of functional equipments*	1 Yes<br>2 No
hosp_d_128 *(required)*	Phototherapy machine<br>*If yes, please specify number of functional equipments*	1 Yes<br>2 No
hosp_d_129 *(required)*	Pediatric Resuscitation Kit<br>*If yes, please specify number of functional equipments*	1 Yes<br>2 No
hosp_d_130 *(required)*	Breast pump (Manual)<br>*If yes, please specify number of functional equipments*	1 Yes<br>2 No
hosp_d_130_1 *(required)*	Breast pump (Electric)<br>*If yes, please specify number of functional equipments*	1 Yes<br>2 No
hosp_d_131 *(required)*	Gynae/Obstetrics Ward	1 Yes<br>2 No
Hospital > Availability Status of Equipment at Hospital > Gynae/Obstetrics Ward<br>*Group relevant when: ${hosp_d_131} =1*		
hosp_d_132 *(required)*	Thermometer<br>*If yes, please specify number of functional equipments*	1 Yes<br>2 No
hosp_d_133 *(required)*	BP Apparatus<br>*If yes, please specify number of functional equipments*	1 Yes<br>2 No
hosp_d_134 *(required)*	Glucometer with sticks<br>*If yes, please specify number of functional equipments*	1 Yes<br>2 No
hosp_d_135 *(required)*	Adult Weighing Scale (Digital)<br>*If yes, please specify number of functional equipments*	1 Yes<br>2 No
hosp_d_135_1 *(required)*	Adult Weighing Scale (Analog)<br>*If yes, please specify number of functional equipments*	1 Yes<br>2 No
hosp_d_136 *(required)*	Oxygen Cylinder<br>*If yes, please specify number of functional equipments*	1 Yes<br>2 No

Field	Question	Answer
hosp_d_180 *(required)*	O2 concentrator<br>*If yes, please specify number of functional equipments*	1 Yes<br>2 No
hosp_d_191 *(required)*	Immunization Services	1 Yes<br>2 No
Hospital > Availability Status of Equipment at Hospital > Immunization Services<br>*Group relevant when: ${hosp_d_191} =1*		
hosp_d_157 *(required)*	ILR with Voltage Stabilizer<br>*If yes, please specify number of functional equipments*	1 Yes<br>2 No
hosp_d_158 *(required)*	DF Small with Voltage Stabilizer<br>*If yes, please specify number of functional equipments*	1 Yes<br>2 No
hosp_d_159 *(required)*	Freeze Tag (2 no per ILR bimonthly)<br>*If yes, please specify number of functional equipments*	1 Yes<br>2 No
hosp_d_160 *(required)*	Thermometer<br>*If yes, please specify number of functional equipments*	1 Yes<br>2 No
hosp_d_161 *(required)*	Vaccine Carriers with Ice Packs<br>*If yes, please specify number of functional equipments*	1 Yes<br>2 No
hosp_d_162 *(required)*	Cold Box<br>*If yes, please specify number of functional equipments*	1 Yes<br>2 No
hosp_d_163 *(required)*	Waste Disposable Twin Buckets<br>*If yes, please specify number of functional equipments*	1 Yes<br>2 No
Hospital > Availability Status of Equipment at Hospital > Blood Bank / Blood Storage Units		
hosp_d_170 *(required)*	Blood bag refrigerators with real-time temperature monitoring devices	1 Yes<br>2 No
lab_facility *(required)*	Is lab service available ?	1 Yes<br>2 No
lab_services *(required)*	Type of facility<br>*Question relevant when: ${lab_facility} =1*	1 Goverment<br>2 KRSNAA/Empanelled
Hospital > Availability Status of Diagnostic tests/services at hosp<br>*Group relevant when: selected(${lab_services} ,1) or selected(${lab_services} ,2)*		
hosp_e_1 *(required)*	Hemoglobin - Required Equipment & Reagents are available in the Laboratory?<br>*If Yes, what method was used?*	1 Tested in facility<br>3 Only sample collection<br>2 Not Available
hosp_e_2 *(required)*	Platelet Count<br>*If Yes, what method was used?*	1 Tested in facility<br>3 Only sample collection<br>2 Not Available
hosp_e_3 *(required)*	Complete Blood Count with ESR<br>*If Yes, what method was used?*	1 Tested in facility<br>3 Only sample collection<br>2 Not Available
hosp_e_4 *(required)*	Blood Group And Rh Typing<br>*If No, what other method was used?*	1 Tested in facility<br>3 Only sample collection<br>2 Not Available
hosp_e_5 *(required)*	Blood Cross Matching<br>*If Yes, what method was used?*	1 Tested in facility<br>3 Only sample collection<br>2 Not Available
hosp_e_6 *(required)*	Bleeding Time and Clotting Time<br>*If Yes, what method was used?*	1 Tested in facility<br>3 Only sample collection<br>2 Not Available
hosp_e_7 *(required)*	MP slide method<br>*If Yes, what other method was used?*	1 Tested in facility<br>3 Only sample collection<br>2 Not Available
hosp_e_8 *(required)*	Malaria rapid test<br>*If Yes, what other method was used?*	1 Tested in facility<br>3 Only sample collection<br>2 Not Available
hosp_e_9 *(required)*	Human chorionic gonadotropin (HCG) (Urine test for pregnancy)<br>*If Yes, what other method was used?*	1 Yes<br>2 No
hosp_e_10 *(required)*	Urine Microscopy<br>*If Yes, what other method was used?*	1 Tested in facility<br>3 Only sample collection<br>2 Not Available
hosp_e_11 *(required)*	Urine for microalbumin<br>*If Yes, what other method was used?*	1 Tested in facility

Field	Question	Answer
		3 Only sample collection<br>2 Not Available
hosp_e_28 *(required)*	Urine for Culture<br>*If Yes, what other method was used?*	1 Tested in facility<br>3 Only sample collection<br>2 Not Available
hosp_e_12 *(required)*	Stool for ova and cyst<br>*If Yes, what method was used?*	1 Tested in facility<br>3 Only sample collection<br>2 Not Available
hosp_e_13 *(required)*	Stool for Occult Blood<br>*If Yes, what method was used?*	1 Tested in facility<br>3 Only sample collection<br>2 Not Available
hosp_e_14 *(required)*	RPR/VDRL test for syphilis<br>*If Yes, what method was used?*	1 Tested in facility<br>3 Only sample collection<br>2 Not Available
hosp_e_15 *(required)*	HIV test (Antibodies 1/2 and HIV 1/2)<br>*If Yes, what method was used?*	1 Tested in facility<br>3 Only sample collection<br>2 Not Available
hosp_e_16 *(required)*	Hepatitis B surface antigen test<br>*If Yes, what method was used?*	1 Tested in facility<br>3 Only sample collection<br>2 Not Available
hosp_e_17 *(required)*	HCV Antibody Test (Anti HCV)<br>*If Yes, what method was used?*	1 Tested in facility<br>3 Only sample collection<br>2 Not Available
hosp_e_19 *(required)*	Sputum, pus etc. for AFB<br>*If Yes, what method was used?*	1 Tested in facility<br>3 Only sample collection<br>2 Not Available
hosp_e_20 *(required)*	Typhoid test (IgM)<br>*If Yes, what method was used?*	1 Tested in facility<br>3 Only sample collection<br>2 Not Available
hosp_e_21 *(required)*	Blood sugar<br>*If Yes, what method was used?*	1 Tested in facility<br>3 Only sample collection<br>2 Not Available
hosp_e_22 *(required)*	Oral Glucose Tolerance Test (OGTT)<br>*If Yes, what method was used?*	1 Tested in facility<br>3 Only sample collection<br>2 Not Available
hosp_e_23 *(required)*	Wet mount and Gram stain for RTI/STD<br>*If Yes, what other method was used?*	1 Tested in facility<br>3 Only sample collection<br>2 Not Available
hosp_e_24 *(required)*	S. TSH (including for newborn screening) also include T3,T4 , TSH FOR PW<br>*If Yes, what method was used?*	1 Tested in facility<br>3 Only sample collection<br>2 Not Available
hosp_e_25 *(required)*	STI/RTI<br>*If Yes, what method was used?*	1 Tested in facility<br>3 Only sample collection<br>2 Not Available
hosp_e_26 *(required)*	TB - NAAT<br>*If Yes, what method was used?*	1 Tested in facility<br>3 Only sample collection<br>2 Not Available
hosp_e_27 *(required)*	Ferritin<br>*If Yes, what method was used?*	1 Tested in facility<br>3 Only sample collection<br>2 Not Available
hosp_e_29 *(required)*	HPLC<br>*If Yes, what method was used?*	1 Tested in facility<br>3 Only sample collection<br>2 Not Available
hosp_e_30 *(required)*	HbA1c<br>*If Yes, what method was used?*	1 Tested in facility<br>3 Only sample collection<br>2 Not Available
hosp_e_31 *(required)*	Dengue Test<br>*If Yes, what method was used?*	1 Tested in facility<br>3 Only sample collection<br>2 Not Available

Field	Question	Answer
hosp_e_32 *(required)*	Urine Dipstick Test<br>*If Yes, what method was used?*	1 Tested in facility<br>3 Only sample collection<br>2 Not Available
hosp_e_24_remarks	Remarks (if any)	
Hospital > hosp_iec		
generated_table_list_label_699	Availability Status of IEC Material/algorithms and management charts related to the national/state programs in local script.	
reserved_name_for_field_list_labels_700		1 Program being implemented<br>2 IEC material Available<br>3 IEC material displayed<br>4 Program not being implemented
hosp_f_1 *(required)*	Family Planning- Mission Parivar Vikas	1 Program being implemented<br>2 IEC material Available<br>3 IEC material displayed<br>4 Program not being implemented
hosp_f_2 *(required)*	Anemia- AMB charts and algorithms	1 Program being implemented<br>2 IEC material Available<br>3 IEC material displayed<br>4 Program not being implemented
hosp_f_3 *(required)*	Adolescent health programs	1 Program being implemented<br>2 IEC material Available<br>3 IEC material displayed<br>4 Program not being implemented
hosp_f_4 *(required)*	Nutrition- POSHAN Abhiyan	1 Program being implemented<br>2 IEC material Available<br>3 IEC material displayed<br>4 Program not being implemented
hosp_f_5 *(required)*	Sanitation and Hygiene: Swachh Bharat Mission Gramin, handwashing	1 Program being implemented<br>2 IEC material Available<br>3 IEC material displayed<br>4 Program not being implemented
hosp_f_6 *(required)*	ANC	1 Program being implemented<br>2 IEC material Available<br>3 IEC material displayed<br>4 Program not being implemented
hosp_f_7 *(required)*	PMSMA	1 Program being implemented<br>2 IEC material Available<br>3 IEC material displayed<br>4 Program not being implemented
hosp_f_8 *(required)*	Management of PPH, Shock	1 Program being implemented<br>2 IEC material Available<br>3 IEC material displayed<br>4 Program not being implemented
hosp_f_9 *(required)*	Newborn Resuscitation, NSSK	1 Program being implemented<br>2 IEC material Available<br>3 IEC material displayed<br>4 Program not being implemented
hosp_f_10 *(required)*	HBNC	1 Program being implemented<br>2 IEC material Available<br>3 IEC material displayed<br>4 Program not being implemented
hosp_f_11 *(required)*	Childhood care- MAA, HBYC	1 Program being implemented<br>2 IEC material Available<br>3 IEC material displayed<br>4 Program not being implemented
hosp_f_12 *(required)*	Immunization	1 Program being implemented<br>2 IEC material Available<br>3 IEC material displayed<br>4 Program not being implemented
hosp_f_13 *(required)*	Breastfeeding	1 Program being implemented

Field	Question	Answer
		2 IEC material Available<br>3 IEC material displayed<br>4 Program not being implemented
hosp_f_14 *(required)*	Diarrhea management- Zinc, ORS	1 Program being implemented<br>2 IEC material Available<br>3 IEC material displayed<br>4 Program not being implemented
hosp_f_15 *(required)*	Pneumonia Management	1 Program being implemented<br>2 IEC material Available<br>3 IEC material displayed<br>4 Program not being implemented
hosp_f_16 *(required)*	JSY	1 Program being implemented<br>2 IEC material Available<br>3 IEC material displayed<br>4 Program not being implemented
hosp_f_17 *(required)*	JSSK	1 Program being implemented<br>2 IEC material Available<br>3 IEC material displayed<br>4 Program not being implemented
hosp_f_18 *(required)*	Ambulance (National Ambulance Services)	1 Program being implemented<br>2 IEC material Available<br>3 IEC material displayed<br>4 Program not being implemented
hosp_f_19 *(required)*	Telemedicine	1 Program being implemented<br>2 IEC material Available<br>3 IEC material displayed<br>4 Program not being implemented
hosp_f_20 *(required)*	Danger Signs (Maternal and Neonatal care)	1 Program being implemented<br>2 IEC material Available<br>3 IEC material displayed<br>4 Program not being implemented
Hospital > hosp_register_group		
hosp_f_10_r *(required)*	General OPD Register	1 Yes<br>2 No
hosp_f_11_r *(required)*	Pre-conception Women Register	1 Yes<br>2 No
hosp_f_12_r *(required)*	ANC Register	1 Yes<br>2 No
hosp_f_13_r *(required)*	Pediatric/0-24 Months Infants & Children Register	1 Yes<br>2 No
hosp_f_14_r *(required)*	Labour Room Register	1 Yes<br>2 No
hosp_f_15_r *(required)*	Laboratory Record	1 Yes<br>2 No
hosp_f_16_r *(required)*	Birth Register	1 Yes<br>2 No
hosp_f_18_r *(required)*	Death Register	1 Yes<br>2 No
hosp_f_17_r *(required)*	Referral Register	1 Yes<br>2 No
Hospital > hosp_g_group		
Hospital > hosp_g_group > hosp_maintenance_record_data<br>*Group relevant when: ${hosp_f_10_r} =1*		
generated_table_list_label_733	General OPD Register	
reserved_name_for_field_list_labels_734		1 Indicator is filled<br>2 Indicator Not filled<br>3 No indicator available

Field	Question	Answer
hosp_g_1_1 *(required)*	Date	1 Indicator is filled<br>2 Indicator Not filled<br>3 No indicator available
hosp_g_1_2 *(required)*	Patient ID	1 Indicator is filled<br>2 Indicator Not filled<br>3 No indicator available
hosp_g_1_3 *(required)*	Name	1 Indicator is filled<br>2 Indicator Not filled<br>3 No indicator available
hosp_g_1_4 *(required)*	Age	1 Indicator is filled<br>2 Indicator Not filled<br>3 No indicator available
hosp_g_1_5 *(required)*	Sex	1 Indicator is filled<br>2 Indicator Not filled<br>3 No indicator available
hosp_g_1_6 *(required)*	Address	1 Indicator is filled<br>2 Indicator Not filled<br>3 No indicator available
hosp_g_1_13 *(required)*	Contact details	1 Indicator is filled<br>2 Indicator Not filled<br>3 No indicator available
hosp_g_1_7 *(required)*	Marital status	1 Indicator is filled<br>2 Indicator Not filled<br>3 No indicator available
hosp_g_1_8 *(required)*	Condition for which visiting OPD	1 Indicator is filled<br>2 Indicator Not filled<br>3 No indicator available
hosp_g_1_9 *(required)*	Treatment provided	1 Indicator is filled<br>2 Indicator Not filled<br>3 No indicator available
hosp_g_1_10 *(required)*	Investigations done	1 Indicator is filled<br>2 Indicator Not filled<br>3 No indicator available
hosp_g_1_11 *(required)*	Follow-up visit date	1 Indicator is filled<br>2 Indicator Not filled<br>3 No indicator available
hosp_g_1_12 *(required)*	Referrals made.	1 Indicator is filled<br>2 Indicator Not filled<br>3 No indicator available
Hospital > hosp_g_group > hosp_preconception_record_data<br>*Group relevant when: ${hosp_f_11_r} =1*		
generated_table_list_label_748	Pre-conception Women Register	
reserved_name_for_field_list_labels_749		1 Indicator is filled<br>2 Indicator Not filled<br>3 No indicator available
hosp_g_2_1 *(required)*	Date of visit	1 Indicator is filled<br>2 Indicator Not filled<br>3 No indicator available
hosp_g_2_2 *(required)*	Woman ID	1 Indicator is filled<br>2 Indicator Not filled<br>3 No indicator available
hosp_g_2_3 *(required)*	Name	1 Indicator is filled<br>2 Indicator Not filled<br>3 No indicator available
hosp_g_2_4 *(required)*	Age	1 Indicator is filled<br>2 Indicator Not filled<br>3 No indicator available
hosp_g_2_5 *(required)*	Address	1 Indicator is filled<br>2 Indicator Not filled

Field	Question	Answer
		3 No indicator available
hosp_g_2_11 *(required)*	Contact details	1 Indicator is filled<br>2 Indicator Not filled<br>3 No indicator available
hosp_g_2_6 *(required)*	Height	1 Indicator is filled<br>2 Indicator Not filled<br>3 No indicator available
hosp_g_2_7 *(required)*	Weight	1 Indicator is filled<br>2 Indicator Not filled<br>3 No indicator available
hosp_g_2_8 *(required)*	BMI	1 Indicator is filled<br>2 Indicator Not filled<br>3 No indicator available
hosp_g_2_9 *(required)*	Medical history (e.g.Anemia,DM,HT,RTI,Thyroid) and screening results of the same conditions	1 Indicator is filled<br>2 Indicator Not filled<br>3 No indicator available
hosp_g_2_10 *(required)*	Place/facility were the conditions tracked during follow up visits	1 Indicator is filled<br>2 Indicator Not filled<br>3 No indicator available
Hospital > hosp_g_group > hosp_anc_record_data<br>*Group relevant when: ${hosp_f_12_r} =1*		
generated_table_list_label_761	ANC Register	
reserved_name_for_field_list_labels_762		1 Indicator is filled<br>2 Indicator Not filled<br>3 No indicator available
hosp_g_3_1 *(required)*	Date of visit	1 Indicator is filled<br>2 Indicator Not filled<br>3 No indicator available
hosp_g_3_2 *(required)*	Woman ID	1 Indicator is filled<br>2 Indicator Not filled<br>3 No indicator available
hosp_g_3_3 *(required)*	Name	1 Indicator is filled<br>2 Indicator Not filled<br>3 No indicator available
hosp_g_3_4 *(required)*	Age	1 Indicator is filled<br>2 Indicator Not filled<br>3 No indicator available
hosp_g_3_5 *(required)*	Address	1 Indicator is filled<br>2 Indicator Not filled<br>3 No indicator available
hosp_g_3_18 *(required)*	Contact details	1 Indicator is filled<br>2 Indicator Not filled<br>3 No indicator available
hosp_g_3_6 *(required)*	Gestational age (GA) at first registration	1 Indicator is filled<br>2 Indicator Not filled<br>3 No indicator available
hosp_g_3_7 *(required)*	BMI in first trimester	1 Indicator is filled<br>2 Indicator Not filled<br>3 No indicator available
hosp_g_3_16 *(required)*	Height	1 Indicator is filled<br>2 Indicator Not filled<br>3 No indicator available
hosp_g_3_17 *(required)*	Weight	1 Indicator is filled<br>2 Indicator Not filled<br>3 No indicator available
hosp_g_3_8 *(required)*	Records/columns of all ANC visits (no. of columns) along with provision to track GWG & Hb	1 Indicator is filled<br>2 Indicator Not filled<br>3 No indicator available
hosp_g_3_9 *(required)*	Blood Pressure	1 Indicator is filled

Field	Question	Answer
		2 Indicator Not filled<br>3 No indicator available
hosp_g_3_10 *(required)*	Blood investigation- CBC at the time of registration	1 Indicator is filled<br>2 Indicator Not filled<br>3 No indicator available
hosp_g_3_19 *(required)*	Blood investigation- TSH at the time of registration	1 Indicator is filled<br>2 Indicator Not filled<br>3 No indicator available
hosp_g_3_11 *(required)*	OGTT- 1 time	1 Indicator is filled<br>2 Indicator Not filled<br>3 No indicator available
hosp_g_3_12 *(required)*	Hb-4 times	1 Indicator is filled<br>2 Indicator Not filled<br>3 No indicator available
hosp_g_3_13 *(required)*	Urine routine & microscopy- 4 times	1 Indicator is filled<br>2 Indicator Not filled<br>3 No indicator available
hosp_g_3_14 *(required)*	Ultrasound	1 Indicator is filled<br>2 Indicator Not filled<br>3 No indicator available
hosp_g_3_15 *(required)*	Td vaccination	1 Indicator is filled<br>2 Indicator Not filled<br>3 No indicator available
Hospital > hosp_g_group > hosp_anc_record_ped<br>*Group relevant when: ${hosp_f_13_r} =1*		
generated_table_list_label_782	Pediatric/0-24 Months Infants & Children Register	
reserved_name_for_field_list_labels_783		1 Indicator is filled<br>2 Indicator Not filled<br>3 No indicator available
hosp_g_4_1 *(required)*	Date of visit	1 Indicator is filled<br>2 Indicator Not filled<br>3 No indicator available
hosp_g_4_2 *(required)*	Child ID	1 Indicator is filled<br>2 Indicator Not filled<br>3 No indicator available
hosp_g_4_3 *(required)*	Name	1 Indicator is filled<br>2 Indicator Not filled<br>3 No indicator available
hosp_g_4_4 *(required)*	Age	1 Indicator is filled<br>2 Indicator Not filled<br>3 No indicator available
hosp_g_4_5 *(required)*	Sex	1 Indicator is filled<br>2 Indicator Not filled<br>3 No indicator available
hosp_g_4_6 *(required)*	Mother Name	1 Indicator is filled<br>2 Indicator Not filled<br>3 No indicator available
hosp_g_4_7 *(required)*	Father Name	1 Indicator is filled<br>2 Indicator Not filled<br>3 No indicator available
hosp_g_4_8 *(required)*	Address	1 Indicator is filled<br>2 Indicator Not filled<br>3 No indicator available
hosp_g_4_17 *(required)*	Contact details	1 Indicator is filled<br>2 Indicator Not filled<br>3 No indicator available
hosp_g_4_9 *(required)*	Weight	1 Indicator is filled<br>2 Indicator Not filled<br>3 No indicator available

Field	Question	Answer
hosp_g_4_10 *(required)*	Height	1 Indicator is filled<br>2 Indicator Not filled<br>3 No indicator available
hosp_g_4_11 *(required)*	Tracking of IWG	1 Indicator is filled<br>2 Indicator Not filled<br>3 No indicator available
hosp_g_4_12 *(required)*	Identification of danger signs	1 Indicator is filled<br>2 Indicator Not filled<br>3 No indicator available
hosp_g_4_13 *(required)*	Morbidities Reported	1 Indicator is filled<br>2 Indicator Not filled<br>3 No indicator available
hosp_g_4_14 *(required)*	Treatment Provided	1 Indicator is filled<br>2 Indicator Not filled<br>3 No indicator available
hosp_g_4_15 *(required)*	Immunisation Status	1 Indicator is filled<br>2 Indicator Not filled<br>3 No indicator available
hosp_g_4_16 *(required)*	Follow-up visit Date	1 Indicator is filled<br>2 Indicator Not filled<br>3 No indicator available
Hospital > hosp_g_group > hosp_labour_room_record_data<br>*Group relevant when: ${hosp_f_14_r} =1*		
generated_table_list_label_801	Labour Room Register	
reserved_name_for_field_list_labels_802		1 Indicator is filled<br>2 Indicator Not filled<br>3 No indicator available
hosp_g_5_1 *(required)*	Date of admission	1 Indicator is filled<br>2 Indicator Not filled<br>3 No indicator available
hosp_g_5_2 *(required)*	Patient ID	1 Indicator is filled<br>2 Indicator Not filled<br>3 No indicator available
hosp_g_5_3 *(required)*	Name	1 Indicator is filled<br>2 Indicator Not filled<br>3 No indicator available
hosp_g_5_4 *(required)*	Age	1 Indicator is filled<br>2 Indicator Not filled<br>3 No indicator available
hosp_g_5_5 *(required)*	Address	1 Indicator is filled<br>2 Indicator Not filled<br>3 No indicator available
hosp_g_5_13 *(required)*	Contact details	1 Indicator is filled<br>2 Indicator Not filled<br>3 No indicator available
hosp_g_5_6 *(required)*	Time of admission	1 Indicator is filled<br>2 Indicator Not filled<br>3 No indicator available
hosp_g_5_7 *(required)*	Type of delivery (e.g. normal,cesarean)	1 Indicator is filled<br>2 Indicator Not filled<br>3 No indicator available
hosp_g_5_8 *(required)*	Complications during delivery	1 Indicator is filled<br>2 Indicator Not filled<br>3 No indicator available
hosp_g_5_9 *(required)*	Birth weight	1 Indicator is filled<br>2 Indicator Not filled<br>3 No indicator available
hosp_g_5_10 *(required)*	APGAR score	1 Indicator is filled<br>2 Indicator Not filled

Field	Question	Answer
		3 No indicator available
hosp_g_5_11 *(required)*	Time of discharge	1 Indicator is filled<br>2 Indicator Not filled<br>3 No indicator available
hosp_g_5_12 *(required)*	Referral information.	1 Indicator is filled<br>2 Indicator Not filled<br>3 No indicator available
Hospital > hosp_g_group > hosp_lab_record<br>*Group relevant when: ${hosp_f_15_r} =1*		
generated_table_list_label_816	Laboratory Record	
reserved_name_for_field_list_labels_817		1 Indicator is filled<br>2 Indicator Not filled<br>3 No indicator available
hosp_g_6_1 *(required)*	Date	1 Indicator is filled<br>2 Indicator Not filled<br>3 No indicator available
hosp_g_6_2 *(required)*	Sample ID	1 Indicator is filled<br>2 Indicator Not filled<br>3 No indicator available
hosp_g_6_3 *(required)*	Patient ID	1 Indicator is filled<br>2 Indicator Not filled<br>3 No indicator available
hosp_g_6_4 *(required)*	Name	1 Indicator is filled<br>2 Indicator Not filled<br>3 No indicator available
hosp_g_6_5 *(required)*	Type of investigation	1 Indicator is filled<br>2 Indicator Not filled<br>3 No indicator available
hosp_g_6_6 *(required)*	Investigation results	1 Indicator is filled<br>2 Indicator Not filled<br>3 No indicator available
hosp_g_6_7 *(required)*	Remarks	1 Indicator is filled<br>2 Indicator Not filled<br>3 No indicator available
hosp_g_6_8 *(required)*	Date of report	1 Indicator is filled<br>2 Indicator Not filled<br>3 No indicator available
Hospital > hosp_g_group > hosp_birth_record<br>*Group relevant when: ${hosp_f_16_r} =1*		
generated_table_list_label_826	Birth Register	
reserved_name_for_field_list_labels_827		1 Indicator is filled<br>2 Indicator Not filled<br>3 No indicator available
hosp_g_7_1 *(required)*	Date of event	1 Indicator is filled<br>2 Indicator Not filled<br>3 No indicator available
hosp_g_7_2 *(required)*	Child ID	1 Indicator is filled<br>2 Indicator Not filled<br>3 No indicator available
hosp_g_7_3 *(required)*	Name of child (if applicable)	1 Indicator is filled<br>2 Indicator Not filled<br>3 No indicator available
hosp_g_7_4 *(required)*	Mother's Name	1 Indicator is filled<br>2 Indicator Not filled<br>3 No indicator available
hosp_g_7_5 *(required)*	Father's Name	1 Indicator is filled<br>2 Indicator Not filled<br>3 No indicator available
hosp_g_7_6 *(required)*	Date of birth	1 Indicator is filled<br>2 Indicator Not filled

Field	Question	Answer
		3 No indicator available
hosp_g_7_7 *(required)*	Place of birth	1 Indicator is filled<br>2 Indicator Not filled<br>3 No indicator available
hosp_g_7_9 *(required)*	Attendant details (e.g., ASHA, AWW)	1 Indicator is filled<br>2 Indicator Not filled<br>3 No indicator available
hosp_g_7_10 *(required)*	Remarks	1 Indicator is filled<br>2 Indicator Not filled<br>3 No indicator available
Hospital > hosp_g_group > hosp_death_record<br>*Group relevant when: ${hosp_f_18_r} =1*		
generated_table_list_label_837	Death Register	
reserved_name_for_field_list_labels_838		1 Indicator is filled<br>2 Indicator Not filled<br>3 No indicator available
hosp_g_7_1_1 *(required)*	Date of event	1 Indicator is filled<br>2 Indicator Not filled<br>3 No indicator available
hosp_g_7_2_1 *(required)*	Child ID	1 Indicator is filled<br>2 Indicator Not filled<br>3 No indicator available
hosp_g_7_3_1 *(required)*	Name of child (if applicable)	1 Indicator is filled<br>2 Indicator Not filled<br>3 No indicator available
hosp_g_7_4_1 *(required)*	Mother's Name	1 Indicator is filled<br>2 Indicator Not filled<br>3 No indicator available
hosp_g_7_5_1 *(required)*	Father's Name	1 Indicator is filled<br>2 Indicator Not filled<br>3 No indicator available
hosp_g_7_6_1 *(required)*	Date of death	1 Indicator is filled<br>2 Indicator Not filled<br>3 No indicator available
hosp_g_7_7_1 *(required)*	Place of death	1 Indicator is filled<br>2 Indicator Not filled<br>3 No indicator available
hosp_g_7_8_1 *(required)*	Cause of death	1 Indicator is filled<br>2 Indicator Not filled<br>3 No indicator available
hosp_g_7_9_1 *(required)*	Attendant details (e.g., ASHA, AWW)	1 Indicator is filled<br>2 Indicator Not filled<br>3 No indicator available
hosp_g_7_10_1 *(required)*	Remarks	1 Indicator is filled<br>2 Indicator Not filled<br>3 No indicator available
Hospital > hosp_g_group > hosp_referral_record<br>*Group relevant when: ${hosp_f_17_r} =1*		
generated_table_list_label_849	Referral Register	
reserved_name_for_field_list_labels_850		1 Indicator is filled<br>2 Indicator Not filled<br>3 No indicator available
hosp_g_8_1 *(required)*	Date of referral	1 Indicator is filled<br>2 Indicator Not filled<br>3 No indicator available
hosp_g_8_2 *(required)*	Patient ID	1 Indicator is filled<br>2 Indicator Not filled<br>3 No indicator available
hosp_g_8_3 *(required)*	Name	1 Indicator is filled<br>2 Indicator Not filled

Field	Question	Answer
		3 No indicator available
hosp_g_8_4 *(required)*	Address	1 Indicator is filled<br>2 Indicator Not filled<br>3 No indicator available
hosp_g_8_5 *(required)*	Reason for referral	1 Indicator is filled<br>2 Indicator Not filled<br>3 No indicator available
hosp_g_8_6 *(required)*	Referring facility	1 Indicator is filled<br>2 Indicator Not filled<br>3 No indicator available
hosp_g_8_7 *(required)*	Receiving facility	1 Indicator is filled<br>2 Indicator Not filled<br>3 No indicator available
hosp_g_8_8 *(required)*	Date of receiving feedback	1 Indicator is filled<br>2 Indicator Not filled<br>3 No indicator available
hosp_g_8_9 *(required)*	Follow-up visit date	1 Indicator is filled<br>2 Indicator Not filled<br>3 No indicator available
Hospital > hosp_i_group		
hosp_i_1_1 *(required)*	Iron, Vitamin, and Nutritional Supplements<br>*Response constrained to: not(selected(${hosp_i_1_1} , '9999') and count-selected(${hosp_i_1_1}) > 1)*	1 Ferrous Salts equivalent to 60 mg of Elemental Iron<br>2 Ferrous Salts Oral Liquid equivalent to 25 mg of Elemental Iron/ml<br>3 Ferrous Salt (A) + Folic Acid (B) Tablet 45 mg Elemental Iron (A) + 400 mcg Folic Acid (B)<br>4 Ferrous Salt (A) + Folic Acid (B) Tablet 60 mg Elemental Iron (A)+ 500 mcg Folic Acid (B)<br>5 Ferrous Salt (A) + Folic Acid (B) Oral Liqiud 20 mg Elemental Iron(A) + 100 mcg (B)/ml<br>6 Folic Acid Tablet 5 mg<br>7 Folic Acid Tablet 400 mcg<br>8 Folic Acid 5 mg Injection 1 mg/ml<br>9 Iron Sucrose Injection 20 mg/ml<br>10 Phytomenadione (Vitamin K1) Tablet 10 mg<br>11 Phytomenadione (Vitamin K1) Inj 10 mg/ml<br>12 Calcium with Vitamin D Tablets USP<br>13 Syrup Calcium with Phosphate in the ratio 2:1 with Vitamin D<br>14 Tablet Calcium Carbonate 650 mg equivalent to Elemental Calcium 250 mg and Cholecalciferol USP 125 mg<br>15 Cholecalciferol Tablet 1000 IU<br>16 Cholecalciferol Tablet 60,000 IU

Field	Question	Answer
		17 Cholecalciferol Oral Liquid 400 IU/ml<br>18 Cholecalciferol Granules 60,000 IU in Sachet<br>19 Cholecalciferol Injection of 6,00,000 IU<br>20 Vitamin D3 60,000 Granules in 1 gm Sachet<br>21 Vitamin A Oral liquid 100000 IU/ml<br>22 Zinc Sulphate tablets<br>23 Multiple Micronutrients (MMN) (WINGS)<br>99 Other Medicine (Specify)<br>9999 None
Hospital > hosp_i_group > [hosp_i_1_cal1] (1)		(Repeated group)
Hospital > hosp_i_group > [hosp_i_1_cal1] (1) > Availability Status of Essential Medicines Required at hosp-SHC for MCH Care [hosp_i_1_cal1]}<br>*Group relevant when: ${hosp_i_1_1} !=9999*		
hosp_i_1_t *(required)*	Target beneficiary	1 Preconception women (18-35 Years)<br>2 Pregnant women<br>3 Postnatal/ lactating women<br>4 0-6 Months Infants<br>5 6-24 Months Infants & Children
hosp_i_1_2 *(required)*	Projected Monthly Requirements	
hosp_i_1_3 *(required)*	Current Numbers in Stock	
hosp_i_1_4 *(required)*	Stock Out in the last 3 month	1 Yes<br>2 No
hosp_i_2_1 *(required)*	Antibiotics<br>*Response constrained to: not(selected(${hosp_i_2_1} , '9999') and count-selected(${hosp_i_2_1}) > 1)*	1 Inj. Gentamicin 80 mg (40 mg/ml)<br>2 Injection Ampicillin<br>3 Injection Amikacin<br>4 Albendazole Tablet 400 mg<br>5 Albendazole Oral Liquid 200 mg/5 ml<br>6 Amoxicillin Capsule 250 mg<br>7 Amoxicillin Tablet 500 mg<br>8 Ampicillin Capsule 500 mg<br>9 Ampicillin Injection 500 mg/ml<br>10 Amoxicillin (500 mg) + Clavulanic Acid (125 mg) Tablet<br>11 Amoxicillin (875 mg) + Clavulanic Acid (125 mg) Tablet<br>12 Benzylpenicillin Injection 10 lakh unit<br>13 Cefixime Tablet 200 mg<br>14 Cefotaxime Injection 1 g/vial<br>15 Piperacillin (A) + Tazobactam (B) Powder for Injection 1 g (A) + 125 mg (B)/2 g (A) + 250 mg (B)/4 g (A) + 500 mg (B)<br>16 Azithromycin Tablet 250 mg/500 mg/Oral Liquid 200 mg/5 ml<br>17 Norfloxacin Tablet 400 mg<br>18 Ciprofloxacin Tablet 250 mg/500 mg/Oral Liquid 250 mg/5 ml

Field	Question	Answer
		19 Ciprofloxacin I.V 200 mg/100 ml<br>20 Co-trimoxazole [Sulphamethoxazol e (A) +Trimethoprim (B)] Tablet 400 mg (A) + 80 mg (B)/Tablet 800 mg (A) + 160 mg (B) /Oral liquid 200 mg (A) + 40 mg (B)/5 ml<br>21 Doxycycline Capsule 100 mg<br>22 Metronidazole Tablet 200 mg, 400 mg<br>23 Metronidazole Injection 5 mg/ml, 500 mg/100 ml<br>24 Levofloxacin Tablet 250 mg/500 mg/750 mg<br>25 Tetracycline/Chloramphenicol eye drops<br>26 Atropine eye drops<br>27 Chloramphenicol<br>99 Other Medicine(Specify)<br>9999 None
Hospital > hosp_i_group > [hosp_i_2_cal1] (1)		(Repeated group)
Hospital > hosp_i_group > [hosp_i_2_cal1] (1) > Availability Status of Essential Medicines Required at hosp-SHC for MCH Care [hosp_i_2_cal1]}<br>*Group relevant when: ${hosp_i_2_1} !=9999*		
hosp_i_2_t *(required)*	Target beneficiary	1 Preconception women (18-35 Years)<br>2 Pregnant women<br>3 Postnatal/ lactating women<br>4 0-6 Months Infants<br>5 6-24 Months Infants & Children
hosp_i_2_2 *(required)*	Projected Monthly Requirements	
hosp_i_2_3 *(required)*	Current Numbers in Stock	
hosp_i_2_4 *(required)*	Stock Out in the last 3 month	1 Yes<br>2 No
hosp_i_3_1 *(required)*	Antihypertensives and Seizure Management<br>*Response constrained to: not(selected(${hosp_i_3_1} , '9999') and count-selected(${hosp_i_3_1}) > 1)*	1 Labetalol Injection 5 mg/ml/20 mg/2ml<br>2 Labetalol Tablet 100 mg<br>3 Methyldopa Tablet 250 mg/500 mg<br>4 Enalapril Tablet 2.5 mg/5 mg/10 mg<br>5 Tab Digoxin - Digoxin IP 250 ug/tab<br>6 Inj. Lorazepam 1mg/ml<br>7 Carbamazepine Tablet 100 mg, 200 mg<br>8 Lorazepam Tablet 1 mg<br>9 Lorazepam Injection 1 or 2 mg/ml<br>10 Magnesium Sulphate Injection 500 mg/ml<br>11 Magnesium Sulphate Injection IP 50% w/v<br>12 Phenytoin Tablet 50 mg/100 mg, 300 mg<br>13 Phenytoin Injection 25 mg/ml, 50 mg/ml<br>14 Sodium Valproate Tablet 100 mg/500 mg<br>15 Inj. Calcium Gluconate 1gm<br>99 Other Medicine(Specify)

Field	Question	Answer
		9999 None
Hospital > hosp_i_group > [hosp_i_3_cal1] (1)		(Repeated group)
Hospital > hosp_i_group > [hosp_i_3_cal1] (1) > Availability Status of Essential Medicines Required at hosp-SHC for MCH Care [hosp_i_3_cal1]}<br>*Group relevant when: ${hosp_i_3_1} !=9999*		
hosp_i_3_t *(required)*	Target beneficiary	1 Preconception women (18-35 Years)<br>2 Pregnant women<br>3 Postnatal/ lactating women<br>4 0-6 Months Infants<br>5 6-24 Months Infants & Children
hosp_i_3_2 *(required)*	Projected Monthly Requirements	
hosp_i_3_3 *(required)*	Current Numbers in Stock	
hosp_i_3_4 *(required)*	Stock Out in the last 3 month	1 Yes<br>2 No
hosp_i_4_1 *(required)*	Analgesics and Antipyretics<br>*Response constrained to: not(selected(${hosp_i_4_1} , '9999') and count-selected(${hosp_i_4_1}) > 1)*	1 Diclofenac Tablet 50 mg<br>2 Diclofenac Injection 25 mg/ml<br>3 Diclofenac Injection 25 mg/3 ml<br>4 Ibuprofen Tablet 200 mg<br>5 Ibuprofen Tablet 400 mg<br>6 Ibuprofen Syrup 100 mg/5 ml<br>7 Paracetamol Tablet 500 mg/650 mg<br>8 Paracetamol Injection 150 mg/ml<br>9 Paracetamol Syrup 125 mg/5 ml<br>10 Hydroxychloroquine Tablet 200 mg<br>11 Hydroxychloroquine Tablet 400 mg<br>99 Other Medicine (Specify)<br>9999 None
Hospital > hosp_i_group > [hosp_i_4_cal1] (1)		(Repeated group)
Hospital > hosp_i_group > [hosp_i_4_cal1] (1) > Availability Status of Essential Medicines Required at hosp-SHC for MCH Care [hosp_i_4_cal1]}<br>*Group relevant when: ${hosp_i_4_1} !=9999*		
hosp_i_4_t *(required)*	Target beneficiary	1 Preconception women (18-35 Years)<br>2 Pregnant women<br>3 Postnatal/ lactating women<br>4 0-6 Months Infants<br>5 6-24 Months Infants & Children
hosp_i_4_2 *(required)*	Projected Monthly Requirements	
hosp_i_4_3 *(required)*	Current Numbers in Stock	
hosp_i_4_4 *(required)*	Stock Out in the last 3 month	1 Yes<br>2 No
hosp_i_5_1 *(required)*	Anthelmintics<br>*Response constrained to: not(selected(${hosp_i_5_1} , '9999') and count-selected(${hosp_i_5_1}) > 1)*	1 Tab. Albendazole (Chewable) 400 mg/Tab<br>2 Albendazole Oral Solution 200 mg/5 ml<br>3 Oral Antimalarial<br>99 Other Medicine (Specify)<br>9999 None
Hospital > hosp_i_group > [hosp_i_5_cal1] (1)		(Repeated group)
Hospital > hosp_i_group > [hosp_i_5_cal1] (1) > Availability Status of Essential Medicines Required at hosp-SHC for MCH Care [hosp_i_5_cal1]}<br>*Group relevant when: ${hosp_i_5_1} !=9999*		
hosp_i_5_t *(required)*	Target beneficiary	1 Preconception women (18-35 Years)<br>2 Pregnant women<br>3 Postnatal/ lactating women<br>4 0-6 Months Infants

Field	Question	Answer
		5 6-24 Months Infants & Children
hosp_i_5_2 *(required)*	Projected Monthly Requirements	
hosp_i_5_3 *(required)*	Current Numbers in Stock	
hosp_i_5_4 *(required)*	Stock Out in the last 3 month	1 Yes<br>2 No
hosp_i_6_1 *(required)*	Diabetic & Thyroid Medications<br>*Response constrained to: not(selected(${hosp_i_6_1} , '9999') and count-selected(${hosp_i_6_1}) > 1)*	1 Inj. Human Soluble Insulin (40 IU/ml)<br>2 Tab. Metformin HCl (Coated) (500 mg/Tab)<br>3 Tab. Eltroxin / Thyroxine (100 mcg)<br>99 Other Medicine (Specify)<br>9999 None
Hospital > hosp_i_group > [hosp_i_6_cal1] (1)		(Repeated group)
Hospital > hosp_i_group > [hosp_i_6_cal1] (1) > Availability Status of Essential Medicines Required at hosp-SHC for MCH Care [hosp_i_6_cal1]}<br>*Group relevant when: ${hosp_i_6_1} !=9999*		
hosp_i_6_t *(required)*	Target beneficiary	1 Preconception women (18-35 Years)<br>2 Pregnant women<br>3 Postnatal/ lactating women<br>4 0-6 Months Infants<br>5 6-24 Months Infants & Children
hosp_i_6_2 *(required)*	Projected Monthly Requirements	
hosp_i_6_3 *(required)*	Current Numbers in Stock	
hosp_i_6_4 *(required)*	Stock Out in the last 3 month	1 Yes<br>2 No
hosp_i_7_1 *(required)*	Other Miscellaneous<br>*Response constrained to: not(selected(${hosp_i_7_1} , '9999') and count-selected(${hosp_i_7_1}) > 1)*	1 Inj. Td (0.5 ml/Amp)<br>2 Tab. Digoxin (0.25 mg/Tab)<br>3 Tab. Frusemide (40 mg/Tab)<br>4 Inj. Frusemide (10 mg/1 ml)<br>5 Inj. Betamethasone Sod. Phosphate (4 mg/ml)<br>6 Inj. Human Anti-D Immunoglobulin (300 mcg/1.5 ml)<br>7 IV Fluids<br>8 ORS Sachet<br>9 Zinc Tablet<br>10 Povidone Iodine Ointment (5% w/v)<br>11 Sanitary Napkins<br>12 Vaccines (As per National Programme)<br>99 Other Medicine (Specify)<br>9999 None
Hospital > hosp_i_group > [hosp_i_7_cal1] (1)		(Repeated group)
Hospital > hosp_i_group > [hosp_i_7_cal1] (1) > Availability Status of Essential Medicines Required at hosp-SHC for MCH Care [hosp_i_7_cal1]}<br>*Group relevant when: ${hosp_i_7_1} !=9999*		
hosp_i_7_t *(required)*	Target beneficiary	1 Preconception women (18-35 Years)<br>2 Pregnant women<br>3 Postnatal/ lactating women<br>4 0-6 Months Infants<br>5 6-24 Months Infants & Children
hosp_i_7_2 *(required)*	Projected Monthly Requirements	
hosp_i_7_3 *(required)*	Current Numbers in Stock	
hosp_i_7_4 *(required)*	Stock Out in the last 3 month	1 Yes<br>2 No
Hospital > hosp_j_group		
Hospital > hosp_j_group > Facility's Beneficiaries (Patient) Load		
hosp_j_note	Please mention the Numbers (Last 12 Month Data) against each	

Field	Question	Answer
hosp_j_1 *(required)*	Eligible couples	
hosp_j_2 *(required)*	Preconception women (18-35 years)	
hosp_j_3 *(required)*	Pregnant women	
hosp_j_4 *(required)*	Postnatal women	
hosp_j_5 *(required)*	0 to 6 months infants	
hosp_j_6 *(required)*	6 to 12 months infants	
hosp_j_7 *(required)*	12 to 24 months children	
hosp_j_8 *(required)*	Preconception women (18-35 years) screened for medical conditions	
hosp_j_9 *(required)*	Preconception women (18-35 years) screened for depressive symptoms-	
Hospital > hosp_j_group > Preconception women (18-35 years) screened for medical conditions and depressive symptoms- *Group relevant when: ${hosp_j_2} != 0 and ${hosp_j_2} != 999*		
hosp_j_8_note	Please mention the Number of Preconception women (18-35 years) screened (Last 12 Month Data) against each	
hosp_j_8_1 *(required)*	RTI/STI	
hosp_j_8_2 *(required)*	TB	
hosp_j_8_3 *(required)*	Epilepsy	
hosp_j_8_4 *(required)*	Syphilis	
hosp_j_8_5 *(required)*	Thyroid status	
hosp_j_8_6 *(required)*	Blood pressure	
hosp_j_8_7 *(required)*	Blood sugar	
hosp_j_8_8 *(required)*	Height measured	
hosp_j_8_9 *(required)*	Weight measured	
hosp_j_8_10 *(required)*	Hemoglobin tested	
hosp_j_8_11 *(required)*	PHQ 2	
Hospital > hosp_j_group > Preconception women (aged 18-35 years) identified with medical conditions & depressive symptoms following screening- *Group relevant when: ${hosp_j_2} != 0 and ${hosp_j_2} != 999*		
hosp_j_9_note	Please mention the Number of Preconception women (aged 18-35 years) identified with medical conditions & depressive symptoms following screening (Last 12 Month Data) against each	
hosp_j_9_1 *(required)*	RTI	
hosp_j_9_1_1 *(required)*	STI	
hosp_j_9_2 *(required)*	TB	
hosp_j_9_3 *(required)*	Epilepsy	
hosp_j_9_4 *(required)*	Syphilis	
hosp_j_9_5 *(required)*	Hypothyroidism (TSH > 5.5 IU/mL)	
hosp_j_9_6 *(required)*	Hyperthyroidism (TSH <0.4 IU/mL)	
hosp_j_9_7 *(required)*	Hypertension	
hosp_j_9_7_1 *(required)*	Hypotension	
hosp_j_9_8 *(required)*	Prediabetes (HbA1c 5.7% to 6.4%)	
hosp_j_9_9 *(required)*	Diabetes mellitus (HbA1c ≥6.5%)	
hosp_j_9_10 *(required)*	BMI<16 kg/m2	
hosp_j_9_11 *(required)*	BMI 16-18.49 kg/m2	
hosp_j_9_12 *(required)*	BMI 18.5- <21 kg/m2	
hosp_j_9_13 *(required)*	Severe anemia (Hb <7 g/dL)	
hosp_j_9_14 *(required)*	Moderate anemia (Hb 7 to 11.99 g/dL)	
hosp_j_9_15 *(required)*	Depressive symptoms	
Hospital > hosp_j_group > Preconception women (aged 18-35 years) managed *Group relevant when: ${hosp_j_2} != 0 and ${hosp_j_2} != 999*		
hosp_j_10_note	Please mention the Number of Preconception women (aged 18-35 years) managed for (Last 12 Month Data) the following conditions	
hosp_j_10_1 *(required)*	RTI	
hosp_j_10_1_1 *(required)*	STI	
hosp_j_10_2 *(required)*	TB	
hosp_j_10_3 *(required)*	Epilepsy	
hosp_j_10_4 *(required)*	Syphilis	
hosp_j_10_5 *(required)*	Hypothyroidism (TSH > 5.5 IU/mL)	
hosp_j_10_6 *(required)*	Hyperthyroidism (TSH <0.4 IU/mL)	
hosp_j_10_7 *(required)*	Hypertension	
hosp_j_10_7_1 *(required)*	Hypotension	
hosp_j_10_8 *(required)*	Prediabetes (HbA1c 5.7% to 6.4%)	
hosp_j_10_9 *(required)*	Diabetes mellitus (HbA1c ≥6.5%)	
hosp_j_10_10 *(required)*	BMI<16 kg/m2	

Field	Question	Answer
hosp_j_10_11 *(required)*	BMI 16-18.49 kg/m2	
hosp_j_10_12 *(required)*	BMI 18.5- <21 kg/m2	
hosp_j_10_13 *(required)*	Severe anemia (Hb <7 g/dL)	
hosp_j_10_14 *(required)*	Moderate anemia (Hb 7 to 11.99 g/dL)	
hosp_j_10_15 *(required)*	Depressive symptoms	
hosp_j_10_16 *(required)*	No. of preconception women who were given IFA as per recommendation	
hosp_j_10_17 *(required)*	No. of preconception women who counselled for Nutrition	
hosp_j_10_18 *(required)*	No. of preconception women who counselled for WASH intervention	
hosp_j_11 *(required)*	Number of Pregnant Women Registered for ANC Care in the last 12 months	
Hospital > hosp_j_group > Pregnant women screened/tested for -<br>*Group relevant when: ${hosp_j_3} != 0 and ${hosp_j_3} != 999*		
hosp_j_11_note	Please mention the Number of Pregnant women screened/tested for (Last 12 Month Data)	
hosp_j_11_1 *(required)*	Blood grouping	
hosp_j_11_2 *(required)*	Complete Blood Count (CBC) at the time of registration	
hosp_j_11_3 *(required)*	HIV	
hosp_j_11_4 *(required)*	Syphilis	
hosp_j_11_5 *(required)*	Urine routine & microscopy	
hosp_j_11_6 *(required)*	Thyroid status / TSH	
hosp_j_11_7 *(required)*	Gestational Diabetes Mellitus (GDM) / OGTT	
hosp_j_11_8 *(required)*	Blood pressure	
hosp_j_11_9 *(required)*	Height measured in First Trimester	
hosp_j_11_10 *(required)*	Weight measured in First Trimester	
hosp_j_11_11 *(required)*	Weight measured in all trimesters	
hosp_j_11_12 *(required)*	Patients Health Questionnaire (PHQ-2) in each trimester	
hosp_j_11_13 *(required)*	Tested for Hemoglobin (Hb) 4 time during pregnancy	
Hospital > hosp_j_group > Pregnant women identified with medical conditions & depressive symptoms following screening-<br>*Group relevant when: ${hosp_j_3} != 0 and ${hosp_j_3} != 999*		
hosp_j_12_note	Please mention the Number of Pregnant women identified with medical conditions & depressive symptoms following screening (Last 12 Month Data)	
hosp_j_12_1 *(required)*	HIV	
hosp_j_12_2 *(required)*	Syphilis	
hosp_j_12_3 *(required)*	Hypothyroidism	
hosp_j_12_4 *(required)*	Hyperthyroidism	
hosp_j_12_5 *(required)*	Hypertension	
hosp_j_12_5_1 *(required)*	Hypotension	
hosp_j_12_6 *(required)*	Diabetes mellitus	
hosp_j_12_7 *(required)*	BMI < 18.5 kg/m2	
hosp_j_12_8 *(required)*	BMI < 25 kg/m2	
hosp_j_12_9 *(required)*	Pregnant women with inadequate gestational weight gain	
hosp_j_12_10 *(required)*	Severe anemia (Hb < 7 g/dL)	
hosp_j_12_11 *(required)*	Moderate anemia (Hb 7 to 9.9 g/dL)	
hosp_j_12_12 *(required)*	Depressive symptoms	
Hospital > hosp_j_group > Pregnant women managed for-<br>*Group relevant when: ${hosp_j_3} != 0 and ${hosp_j_3} != 999*		
hosp_j_14_note	Please mention the Number of Pregnant women managed for following conditions(Last 12 Month Data)	
hosp_j_14_1 *(required)*	HIV	
hosp_j_14_2 *(required)*	Syphilis	
hosp_j_14_3 *(required)*	Hypothyroidism	
hosp_j_14_4 *(required)*	Hyperthyroidism	
hosp_j_14_5 *(required)*	Hypertension	
hosp_j_14_5_1 *(required)*	Hypotension	
hosp_j_14_6 *(required)*	Diabetes mellitus	
hosp_j_14_7 *(required)*	Inadequate gestational weight gain (IGWG)	
hosp_j_14_8 *(required)*	Severe anemia (Hb < 7 g/dL)	
hosp_j_14_9 *(required)*	Moderate anemia (Hb 7 to 9.9 g/dL)	
hosp_j_14_10 *(required)*	Depressive symptoms	
hosp_j_14_11 *(required)*	Other medical conditions	
hosp_j_14_12 *(required)*	No. of pregnant women who were given IFA as per recommendation	
hosp_j_14_13 *(required)*	No. of pregnant women who counselled for Nutrition	
hosp_j_14_14 *(required)*	No. of pregnant women who counselled for WASH intervention	
Hospital > hosp_j_group > hosp_j_1_group		

Field	Question	Answer
Group relevant when: ${hosp_j_3} != 0 and ${hosp_j_3} != 999		
hosp_j_15 *(required)*	Pregnant women referred during ANC	
hosp_j_16 *(required)*	Pregnant women with atleast 4-ANC check-ups	
hosp_j_17 *(required)*	Pregnant women with atleast 8-ANC check-ups	
hosp_j_18 *(required)*	Pregnant women given corticosteroids for preterm labor	
hosp_j_19 *(required)*	Registered antenatal mothers who came for delivery	
hosp_j_20 *(required)*	Institutional deliveries in facility	
hosp_j_20_1 *(required)*	Institutional deliveries other facility	
hosp_j_21 *(required)*	Home deliveries	
hosp_j_22 *(required)*	Maternal deaths	
hosp_j_23 *(required)*	Live births	
hosp_j_24 *(required)*	Babies born with Very low birth weight (VLBW) < 1,500 grams	
hosp_j_25 *(required)*	Babies born with low birth weight (LBW) < 2,500 grams	
hosp_j_26 *(required)*	Preterm babies born (<37 weeks)	
hosp_j_27 *(required)*	Early neonatal deaths (within 24 hours of birth)	
Hospital > hosp_j_group > Other high-risk pregnancies identified during ANC- *Group relevant when: ${hosp_j_3} != 0 and ${hosp_j_3} != 999*		
hosp_j_13_note	Please mention the Number of Other high-risk pregnancies identified during ANC- (Last 12 Month Data)	
hosp_j_13_1 *(required)*	Pregnant women with multiple pregnancy	
hosp_j_13_2 *(required)*	Pregnant women with previous bad obstetric history (BOH)*	
hosp_j_13_3 *(required)*	Pregnant women with other infections (Hepatitis, Malaria, etc.)	
hosp_j_13_4 *(required)*	Pregnant women with other medical conditions (Asthma, COPD, heart disease, etc.)	
Hospital > hosp_j_group > Postnatal Women (0-6 Months) & 0-24 Months Infants & Child *Group relevant when: ${hosp_j_4} != 0 and ${hosp_j_4} != 999*		
hosp_j_28_note	Please mention the Number of Postnatal Women (0-6 Months) & 0-24 Months Infants & Child (Last 12 Month Data)	
hosp_j_28_1 *(required)*	Newborns screened for birth defects (as per RBSK)	
hosp_j_28_2 *(required)*	Women received postpartum checkup between 48 hours and 14 days after institutional delivery (Facility/Community)	
hosp_j_28_3 *(required)*	HBNC visits performed as per guidelines	
hosp_j_28_4 *(required)*	Postnatal mothers consumed nutritional supplements as per recommendation	
hosp_j_28_5 *(required)*	Postnatal mothers screened for depressive symptoms	
hosp_j_28_6 *(required)*	Postnatal mothers who received appropriate psychosocial support	
hosp_j_28_7 *(required)*	Postnatal mothers who counselled for WASH intervention	
hosp_j_28_8 *(required)*	Postnatal mothers who were given IFA as per recommendation	
hosp_j_28_9 *(required)*	Postnatal mothers who counselled for Nutrition	
hosp_j_28_10 *(required)*	0-6 months Infants received kangaroo mother care (KMC) as applicable (Facility/Home)	
hosp_j_28_11 *(required)*	0-6 months Infants exclusively breastfed (EBF)	
hosp_j_28_12 *(required)*	0-6 months Infants (VLBW and LBW) who consumed Iron, as per recommendation	
hosp_j_28_13 *(required)*	0-6 months Infants who consumed Vitamin D 400 IU daily	
hosp_j_28_14 *(required)*	0-6 months Infants (Pre-term) who consumed calcium and vitamin D, as per recommendation	
hosp_j_28_15 *(required)*	6-24 months infants & children who consumed IFA, as per recommendation	
hosp_j_28_16 *(required)*	Infants 6-12 months with inadequate weight gain (<25th centile according to WHO weight velocity/month)	
hosp_j_28_17 *(required)*	Children 12-24 months with inadequate weight gain (<25th centile according to WHO weight velocity/month)	
hosp_j_28_18 *(required)*	Children (0-24 months) with adequate weight gain from birth till 2 years of age	
hosp_j_28_19 *(required)*	Family/Parents having 0-24 months infants & child counselled for WASH interventions	
hosp_j_28_20 *(required)*	Children (12-23 months) fully immunized	
hosp_j_28_21 *(required)*	Observational Remarks	
hosp_j_28_22 *(required)*	Respondent Remarks	
Hospital > Document		
image1	Document 1	
image2	Document 2	
image3	Document 3	
image4	Document 4	
image5	Document 5	
image6	Document 6	
image7	Document 7	
image8	Document 8	
file_pdf	Please upload PDF Documents	

RH Assessment form

Field	Question	Answer
worker	Worker Name	151 Abuhamza<br>152 Anmol Saini<br>153 Anshika Sahota<br>154 Ekta<br>155 Jyoti Devi<br>156 Kritika Thakur<br>157 Mehak Thakur<br>158 Poonam Devi<br>159 Riya Puri<br>160 Shivanshi<br>161 Varsha Kumari<br>162 Anchal Walia<br>163 Harshali<br>164 Kritika Puri
RH		
RH > RH Identification		
rh_a_1	Date of Assessment:	
rh_a_6 *(required)*	Facility id:	
rh_a_8 *(required)*	Facility Address with landmark	
rh_a_10 *(required)*	Total Population covered by the facility	
rh_a_11 *(required)*	Facility In-charge Name:<br>*Response constrained to: not(regex(.,'^(.*)\d(.*)$'))*	
rh_a_12 *(required)*	Facility In-charge Designation:	
rh_a_13 *(required)*	Facility In-charge Contact No.:<br>*Response constrained to: regex(.,"^[6|7|8|9]\d{9}$")*	
rh_a_14	Facility In-charge Email Id.:	
rh_a_15 *(required)*	Any Additional Respondent?	1 Yes<br>2 No
RH > Additional Respondent<br>*Group relevant when: ${rh_a_15} =1*		
rh_a_15_2 *(required)*	Additional Respondent Name<br>*Response constrained to: not(regex(.,'^(.*)\d(.*)$'))*	
rh_a_15_3 *(required)*	Additional Respondent Designation	
RH > RH Infrastructure		
rh_b_1 *(required)*	Located within Main Habitation (*where the majority of the population resides)<br>*If No, Distance in Km*	1 Yes<br>2 No
rh_b_2 *(required)*	Connected to Motorable Road	1 Yes<br>2 No
rh_b_3 *(required)*	Designated Government rhital Building	1 Yes<br>2 No
rh_b_4 *(required)*	General Structure of Building	1 Newly Built<br>2 Newly Renovated<br>3 Old Structure<br>4 Falling apart
rh_b_5 *(required)*	Maintenance of Building	1 No maintenance<br>2 Once a year<br>3 Once in 3 years<br>99 Other (specify)
rh_b_6 *(required)*	Is electrical supply available?	1 24/7<br>3 Average duration of electricity supply per day<br>2 No
rh_b_6_or	Is power backup available?	1 Generator<br>2 Inverter<br>3 Solar<br>99 Other (Specify)<br>9999 None
rh_b_7 *(required)*	Main source of water supply?	1 Borewell<br>2 Piped water

Field	Question	Answer
		99 Other with duration
rh_b_8 *(required)*	Toilet Facility	1 Yes<br>2 No
rh_b_9 *(required)*	Littering	1 Yes<br>2 No
RH > Toilet & Other facilities		
rh_b_8_1 *(required)*	Facility available inside the toilet<br>*Question relevant when: selected(${rh_b_8} , '1')*	1 Cleanliness<br>2 Toilet tap with running water<br>3 Flush working properly<br>4 Water bucket and mug inside the toilet<br>5 Washbasin with running water<br>6 Soap/liquid items handwash/hand rub<br>7 Disable friendly<br>9999 None
rh_b_8_2 *(required)*	Gender specific toilet facility<br>*Question relevant when: selected(${rh_b_8} , '1')*	1 Both (male & female separate) Available<br>2 Only Male Available<br>3 Only Female Available<br>4 Common Toilet Available<br>5 Availability of a disabled-friendly toilet
rh_b_10 *(required)*	Availability of general waste bins	1 Green<br>2 Blue<br>3 Both<br>99 Other
rh_b_11 *(required)*	Management (collection, segregation, storage, transportation, treatment and disposal) of BMW as per Bio-Medical Waste Management Rules, 2018	1 Yes<br>2 No
rh_b_11_bins *(required)*	Availability of colour coded BMW bins	1 Red<br>2 Yellow<br>3 Blue Puncture proof<br>4 White Puncture proof
RH > Availability Status of Human Resource		
rh_c_2 *(required)*	Sanctioned (No.) MO MBBS	
rh_c_3 *(required)*	In position (No.) MO MBBS<br>*Question relevant when: ${rh_c_2} > 0*<br>*Response constrained to: . >= 0 and . <= ${rh_c_2}*	
RH > Availability Status of Human Resource > MO MBBS (1)<br>*Group relevant when: ${rh_c_2} > 0 and ${rh_c_3} > 0*		(Repeated group)
rh_c_4 *(required)*	Status	1 Available<br>2 Long Leave<br>3 Deputed Somewhere Else
RH > Availability Status of Human Resource > Training Status Of MO MBBS (1)		(Repeated group)
RH > Availability Status of Human Resource > Training Status Of MO MBBS (1) > rh_mo_training		
generated_table_list_label_61	Training Status Of MO MBBS	
reserved_name_for_field_list_labels_62		1 Yes<br>2 No
rh_c_1_1 *(required)*	IUCD Training	1 Yes<br>2 No
rh_c_1_2 *(required)*	Breastfeeding Training	1 Yes<br>2 No
rh_c_1_3 *(required)*	Routine Immunization	1 Yes<br>2 No
rh_c_1_4 *(required)*	Emergency medicine	1 Yes<br>2 No
rh_c_1_5 *(required)*	NSSK	1 Yes<br>2 No
rh_c_1_6 *(required)*	Neonatal care Training	1 Yes

Field	Question	Answer
		2 No
rh_c_1_7 *(required)*	SAANS Training (Under 5 Pneumonia management)	1 Yes<br>2 No
rh_c_1_8 *(required)*	National Health Program Training	1 Yes<br>2 No
rh_c_1_9 *(required)*	Anemia Mukt Bharat	1 Yes<br>2 No
rh_c_1_10 *(required)*	Infection Prevention and Control	1 Yes<br>2 No
rh_c_1_11 *(required)*	Other	1 Yes<br>2 No
RH > Availability Status of Human Resource > Training Status Of MO MBBS (1) > rh_mo_training_others<br>*Group relevant when: ${rh_c_1_11} =1*		
rh_c_1_12 *(required)*	Please specify Training (others)	
rh_c_10 *(required)*	Sanctioned (No.) Nursing Officer	
rh_c_11 *(required)*	In position (No.) Nursing Officer<br>*Question relevant when: ${rh_c_10} > 0*	
RH > Availability Status of Human Resource > Nursing Officer (1)<br>*Group relevant when: ${rh_c_10} > 0 and ${rh_c_11} > 0*		(Repeated group)
rh_c_12 *(required)*	Status	1 Available<br>2 Long Leave<br>3 Deputed Somewhere Else
RH > Availability Status of Human Resource > Training Status Of Nursing Officer (1)		(Repeated group)
RH > Availability Status of Human Resource > Training Status Of Nursing Officer (1) > rh_nurse_training		
generated_table_list_label_84	Training Status Of Nursing Officer	
reserved_name_for_field_list_labels_85		1 Yes<br>2 No
rh_c_9_1 *(required)*	SBA	1 Yes<br>2 No
rh_c_9_2 *(required)*	NSSK	1 Yes<br>2 No
rh_c_9_3 *(required)*	IUCD	1 Yes<br>2 No
rh_c_9_4 *(required)*	Routine Immunization (Birth Dose)	1 Yes<br>2 No
rh_c_9_5 *(required)*	Cold chain management Training	1 Yes<br>2 No
rh_c_9_6 *(required)*	SAANS Training	1 Yes<br>2 No
rh_c_9_7 *(required)*	F-IMNCI	1 Yes<br>2 No
rh_c_9_8 *(required)*	Other	1 Yes<br>2 No
RH > Availability Status of Human Resource > Training Status Of Nursing Officer (1) > rh_nurse_training_others<br>*Group relevant when: ${rh_c_9_9} =1*		
rh_c_9_9 *(required)*	Please specify Training (others)	
rh_c_14 *(required)*	Sanctioned (No.) Pharmacist	
rh_c_15 *(required)*	In position (No.) Pharmacist<br>*Question relevant when: ${rh_c_14} > 0*<br>*Response constrained to: . >= 0 and . <= ${rh_c_14}*	
RH > Availability Status of Human Resource > Pharmacist (1)<br>*Group relevant when: ${rh_c_14} > 0 and ${rh_c_15} > 0*		(Repeated group)
rh_c_16 *(required)*	Status	1 Available<br>2 Long Leave<br>3 Deputed Somewhere Else
RH > Availability Status of Human Resource > Training Status Of Pharmacist (1)		(Repeated group)
RH > Availability Status of Human Resource > Training Status Of Pharmacist (1) > rh_pharmacist_training		
generated_table_list_label_104	Training Status Of Pharmacist	
reserved_name_for_field_list_labels_105		1 Yes<br>2 No

Field	Question	Answer
rh_c_13_1 *(required)*	DVDMS Training	1 Yes<br>2 No
rh_c_13_2 *(required)*	Stock management	1 Yes<br>2 No
rh_c_13_3 *(required)*	Cold chain management	1 Yes<br>2 No
rh_c_13_4 *(required)*	Tuberculosis (TB) Training	1 Yes<br>2 No
rh_c_13_5 *(required)*	BMW Management	1 Yes<br>2 No
rh_c_13_6 *(required)*	Sanitation Training	1 Yes<br>2 No
rh_c_13_7 *(required)*	E-Sanjeevni	1 Yes<br>2 No
rh_c_13_8 *(required)*	Other	1 Yes<br>2 No
RH > Availability Status of Human Resource > Training Status Of Pharmacist (1) > rh_pharmacist_training_others<br>*Group relevant when: ${rh_c_13_8} =1*		
rh_c_13_9 *(required)*	Please specify Training (others)	
rh_c_18 *(required)*	Sanctioned (No.) Storekeeper	
rh_c_19 *(required)*	In position (No.) Storekeeper<br>*Question relevant when: ${rh_c_18} > 0*<br>*Response constrained to: . >= 0 and . <= ${rh_c_18}*	
RH > Availability Status of Human Resource > Storekeeper (1)<br>*Group relevant when: ${rh_c_18} > 0 and ${rh_c_19} > 0*		(Repeated group)
rh_c_20 *(required)*	Status	1 Available<br>2 Long Leave<br>3 Deputed Somewhere Else
RH > Availability Status of Human Resource > Training Status Of Storekeeper (1)		(Repeated group)
RH > Availability Status of Human Resource > Training Status Of Storekeeper (1) > rh_storekeep_training		
generated_table_list_label_124	Training Status Of Storekeeper	
reserved_name_for_field_list_labels_125		1 Yes<br>2 No
rh_c_17_1 *(required)*	DVDMS Training	1 Yes<br>2 No
rh_c_17_2 *(required)*	Others	1 Yes<br>2 No
RH > Availability Status of Human Resource > Training Status Of Storekeeper (1) > rh_storekeep_training_others<br>*Group relevant when: ${rh_c_17_2} =1*		
rh_c_17_8 *(required)*	Please specify Training (others)	
rh_c_22 *(required)*	Sanctioned (No.) Lab Technician	
rh_c_23 *(required)*	In position (No.) Lab Technician<br>*Question relevant when: ${rh_c_22} > 0*<br>*Response constrained to: . >= 0 and . <= ${rh_c_22}*	
RH > Availability Status of Human Resource > Lab Technician (1)<br>*Group relevant when: ${rh_c_22} > 0 and ${rh_c_23} > 0*		(Repeated group)
rh_c_24 *(required)*	Status	1 Available<br>2 Long Leave<br>3 Deputed Somewhere Else
RH > Availability Status of Human Resource > Training Status Of Lab Technician (1)		(Repeated group)
RH > Availability Status of Human Resource > Training Status Of Lab Technician (1) > rh_labtech_training		
generated_table_list_label_138	Training Status Of Lab Technician	
reserved_name_for_field_list_labels_139		1 Yes<br>2 No
rh_c_21_1 *(required)*	NTEP Training	1 Yes<br>2 No
rh_c_21_2 *(required)*	Malaria Training	1 Yes<br>2 No
rh_c_21_3 *(required)*	Use of Rapid Test Kits	1 Yes<br>2 No

Field	Question	Answer
rh_c_21_4 *(required)*	Others	1 Yes<br>2 No
RH > Availability Status of Human Resource > Training Status Of Lab Technician (1) > rh_labtech_training_others<br>*Group relevant when: ${rh_c_21_4} =1*		
rh_c_21_5 *(required)*	Please specify Training (others)	
rh_c_26 *(required)*	Sanctioned (No.) Health Worker (Female)/ANM	
rh_c_27 *(required)*	In position (No.) Health Worker (Female)/ANM<br>*Question relevant when: ${rh_c_26} > 0*<br>*Response constrained to: . >= 0 and . <= ${rh_c_26}*	
RH > Availability Status of Human Resource > Health Worker (Female)/ANM (1)<br>*Group relevant when: ${rh_c_26} > 0 and ${rh_c_27} > 0*		(Repeated group)
rh_c_28 *(required)*	Status	1 Available<br>2 Long Leave<br>3 Deputed Somewhere Else
RH > Availability Status of Human Resource > Training Status Of Health worker (Female)/ANM (1)		(Repeated group)
RH > Availability Status of Human Resource > Training Status Of Health worker (Female)/ANM (1) > rh_hwf_training		
generated_table_list_label_154	Training Status Of Health worker (Female)/ANM	
reserved_name_for_field_list_labels_155		1 Yes<br>2 No
rh_c_25_1 *(required)*	Routine immunization	1 Yes<br>2 No
rh_c_25_2 *(required)*	SAANS Training (Under 5 Pneumonia management)	1 Yes<br>2 No
rh_c_25_3 *(required)*	HBNC Training	1 Yes<br>2 No
rh_c_25_4 *(required)*	Diarrhoea management Training (IDCF)	1 Yes<br>2 No
rh_c_25_5 *(required)*	MAA program	1 Yes<br>2 No
rh_c_25_6 *(required)*	IUCD	1 Yes<br>2 No
rh_c_25_7 *(required)*	NCD	1 Yes<br>2 No
rh_c_25_8 *(required)*	VHND	1 Yes<br>2 No
rh_c_25_9 *(required)*	VHSNC	1 Yes<br>2 No
rh_c_25_10 *(required)*	Eat right tool kit	1 Yes<br>2 No
rh_c_25_11 *(required)*	Other	1 Yes<br>2 No
RH > Availability Status of Human Resource > Training Status Of Health worker (Female)/ANM (1) > rh_hwf_training_others<br>*Group relevant when: ${rh_c_25_11} =1*		
rh_c_25_12 *(required)*	Please specify Training (others)	
rh_c_30 *(required)*	Sanctioned (No.) Health Worker (Male)/ Health Assistant (Male)	
rh_c_31 *(required)*	In position (No.) Health Worker (Male)/ Health Assistant (Male)<br>*Question relevant when: ${rh_c_30} > 0*<br>*Response constrained to: . >= 0 and . <= ${rh_c_30}*	
RH > Availability Status of Human Resource > Health Worker (Male)/ Health Assistant (Male) (1)<br>*Group relevant when: ${rh_c_30} > 0 and ${rh_c_31} > 0*		(Repeated group)
rh_c_32 *(required)*	Status	1 Available<br>2 Long Leave<br>3 Deputed Somewhere Else
RH > Availability Status of Human Resource > Training Status Of Health Worker (Male)/Health Assistant (Male) (1)		(Repeated group)
RH > Availability Status of Human Resource > Training Status Of Health Worker (Male)/Health Assistant (Male) (1) > rh_hwm_training		
generated_table_list_label_177	Training Status Of Health Worker (Male)/Health Assistant (Male)	
reserved_name_for_field_list_labels_178		1 Yes<br>2 No
rh_c_29_1 *(required)*	TB	1 Yes<br>2 No

Field	Question	Answer
rh_c_29_2 *(required)*	Malaria	1 Yes<br>2 No
rh_c_29_3 *(required)*	Routine Immunization	1 Yes<br>2 No
rh_c_29_4 *(required)*	Water testing	1 Yes<br>2 No
rh_c_29_5 *(required)*	Other	1 Yes<br>2 No
RH > Availability Status of Human Resource > Training Status Of Health Worker (Male)/Health Assistant (Male) (1) > rh_hwm_training_others<br>*Group relevant when: ${rh_c_29_5} =1*		
rh_c_29_6 *(required)*	Please specify Training (others)	
rh_c_34 *(required)*	Sanctioned (No.) Female health supervisor	
rh_c_35 *(required)*	In position (No.) Female health supervisor<br>*Question relevant when: ${rh_c_34} > 0*<br>*Response constrained to: . >= 0 and . <= ${rh_c_34}*	
RH > Availability Status of Human Resource > Health Assistant Female health supervisor (1)<br>*Group relevant when: ${rh_c_34} > 0 and ${rh_c_35} > 0*		(Repeated group)
rh_c_36 *(required)*	Status	1 Available<br>2 Long Leave<br>3 Deputed Somewhere Else
RH > Availability Status of Human Resource > Health Assistant Female health supervisor (1)		(Repeated group)
RH > Availability Status of Human Resource > Health Assistant Female health supervisor (1) > rh_lhv_training		
generated_table_list_label_194	Health Assistant Female health supervisor	
reserved_name_for_field_list_labels_195		1 Yes<br>2 No
rh_c_33_1 *(required)*	Cold chain management	1 Yes<br>2 No
rh_c_33_2 *(required)*	Others	1 Yes<br>2 No
RH > Availability Status of Human Resource > Health Assistant Female health supervisor (1) > rh_lhv_training_others<br>*Group relevant when: ${rh_c_33_3} =1*		
rh_c_33_3 *(required)*	Please specify Training (others)	
rh_c_38 *(required)*	Sanctioned (No.) Health Educator/ Counsellor	
rh_c_39 *(required)*	In position (No.) Health Educator/ Counsellor<br>*Question relevant when: ${rh_c_38} > 0*<br>*Response constrained to: . >= 0 and . <= ${rh_c_38}*	
RH > Availability Status of Human Resource > Health Educator/ Counsellor (1)<br>*Group relevant when: ${rh_c_38} > 0 and ${rh_c_39} > 0*		(Repeated group)
rh_c_40 *(required)*	Status	1 Available<br>2 Long Leave<br>3 Deputed Somewhere Else
RH > Availability Status of Human Resource > Health Educator/ Counsellor (1)		(Repeated group)
RH > Availability Status of Human Resource > Health Educator/ Counsellor (1) > rh_hec_training		
generated_table_list_label_208	Health Educator/ Counsellor	
reserved_name_for_field_list_labels_209		1 Yes<br>2 No
rh_c_37_1 *(required)*	HBNC	1 Yes<br>2 No
rh_c_37_2 *(required)*	HBYC	1 Yes<br>2 No
rh_c_37_3 *(required)*	VHND	1 Yes<br>2 No
rh_c_37_4 *(required)*	HIV	1 Yes<br>2 No
rh_c_37_5 *(required)*	Routine Immunization	1 Yes<br>2 No
rh_c_37_6 *(required)*	Adolescent Health	1 Yes<br>2 No
rh_c_37_7 *(required)*	Maternal health	1 Yes<br>2 No

Field	Question	Answer
rh_c_37_8 *(required)*	Anemia Mukt Bharat	1 Yes<br>2 No
rh_c_37_9 *(required)*	Under 5 Pneumonia Management Program	1 Yes<br>2 No
rh_c_37_10 *(required)*	Intensive Diarrhoea Control Fortnight (IDCF)	1 Yes<br>2 No
rh_c_37_11 *(required)*	MAA program	1 Yes<br>2 No
rh_c_37_12 *(required)*	IEC Planning & Implementation Training	1 Yes<br>2 No
rh_c_37_13 *(required)*	Other	1 Yes<br>2 No
RH > Availability Status of Human Resource > Health Educator/ Counsellor (1) > rh_hec_training_others<br>*Group relevant when: ${rh_c_37_13} =1*		
rh_c_37_14 *(required)*	Please specify Training (others)	
rh_c_42 *(required)*	Sanctioned (No.) Data Entry Operator	
rh_c_43 *(required)*	In position (No.) Data Entry Operator<br>*Question relevant when: ${rh_c_42} > 0*<br>*Response constrained to: . >= 0 and . <= ${rh_c_42}*	
RH > Availability Status of Human Resource > Data Entry Operator (1)<br>*Group relevant when: ${rh_c_42} > 0 and ${rh_c_43} > 0*		(Repeated group)
rh_c_44 *(required)*	Status	1 Available<br>2 Long Leave<br>3 Deputed Somewhere Else
rh_c_46 *(required)*	Sanctioned (No.) Obstetrician	
rh_c_47 *(required)*	In position (No.) Obstetrician<br>*Question relevant when: ${rh_c_46} > 0*<br>*Response constrained to: . >= 0 and . <= ${rh_c_46}*	
RH > Availability Status of Human Resource > Obstetrician (1)<br>*Group relevant when: ${rh_c_46} > 0 and ${rh_c_47} > 0*		(Repeated group)
rh_c_48 *(required)*	Status	1 Available<br>2 Long Leave<br>3 Deputed Somewhere Else
rh_c_50 *(required)*	Sanctioned (No.) Pediatrician	
rh_c_51 *(required)*	In position (No.) Pediatrician<br>*Question relevant when: ${rh_c_50} > 0*<br>*Response constrained to: . >= 0 and . <= ${rh_c_50}*	
RH > Availability Status of Human Resource > Pediatrician (1)<br>*Group relevant when: ${rh_c_50} > 0 and ${rh_c_51} > 0*		(Repeated group)
rh_c_52 *(required)*	Status	1 Available<br>2 Long Leave<br>3 Deputed Somewhere Else
rh_c_54 *(required)*	Sanctioned (No.) Anesthetist	
rh_c_55 *(required)*	In position (No.) Anesthetist<br>*Question relevant when: ${rh_c_54} > 0*<br>*Response constrained to: . >= 0 and . <= ${rh_c_54}*	
RH > Availability Status of Human Resource > Anesthetist (1)<br>*Group relevant when: ${rh_c_54} > 0 and ${rh_c_55} > 0*		(Repeated group)
rh_c_56 *(required)*	Status	1 Available<br>2 Long Leave<br>3 Deputed Somewhere Else
rh_c_58 *(required)*	Sanctioned (No.) Radiologist	
rh_c_59 *(required)*	In position (No.) Radiologist<br>*Question relevant when: ${rh_c_58} > 0*<br>*Response constrained to: . >= 0 and . <= ${rh_c_58}*	
RH > Availability Status of Human Resource > Radiologist (1)<br>*Group relevant when: ${rh_c_58} > 0 and ${rh_c_59} > 0*		(Repeated group)
rh_c_60 *(required)*	Status	1 Available<br>2 Long Leave<br>3 Deputed Somewhere Else
rh_c_62 *(required)*	Sanctioned (No.) Psychiatrist	

Field	Question	Answer
rh_c_63 *(required)*	In position (No.) Psychiatrist<br>*Question relevant when: ${rh_c_62} > 0*<br>*Response constrained to: . >= 0 and . <= ${rh_c_62}*	
RH > Availability Status of Human Resource > Psychiatrist (1)<br>*Group relevant when: ${rh_c_62} > 0 and ${rh_c_63} > 0*		(Repeated group)
rh_c_64 *(required)*	Status	1 Available<br>2 Long Leave<br>3 Deputed Somewhere Else
rh_c_66 *(required)*	Sanctioned (No.) Psychologist	
rh_c_67 *(required)*	In position (No.) Psychologist<br>*Question relevant when: ${rh_c_66} > 0*<br>*Response constrained to: . >= 0 and . <= ${rh_c_66}*	
RH > Availability Status of Human Resource > Psychologist (1)<br>*Group relevant when: ${rh_c_66} > 0 and ${rh_c_67} > 0*		(Repeated group)
rh_c_68 *(required)*	Status	1 Available<br>2 Long Leave<br>3 Deputed Somewhere Else
rh_c_70 *(required)*	Sanctioned (No.) Lactation counsellor	
rh_c_71 *(required)*	In position (No.) Lactation counsellor<br>*Question relevant when: ${rh_c_70} > 0*<br>*Response constrained to: . >= 0 and . <= ${rh_c_70}*	
RH > Availability Status of Human Resource > Lactation counsellor (1)<br>*Group relevant when: ${rh_c_70} > 0 and ${rh_c_71} > 0*		(Repeated group)
rh_c_72 *(required)*	Status	1 Available<br>2 Long Leave<br>3 Deputed Somewhere Else
rh_c_73 *(required)*	Any other staff?	1 Yes<br>2 No
RH > Availability Status of Human Resource > anyother_group<br>*Group relevant when: ${rh_c_73} =1*		
any_other1	Name<br>*Response constrained to: not(regex(.,'^(.*)\d(.*)$'))*	
any_other2	Designation<br>*Response constrained to: not(regex(.,'^(.*)\d(.*)$'))*	
RH > rh_infra2		
generated_table_list_label_273	Quality Assurance & Others	
rh_b_12	Quality assurance certification/services	
reserved_name_for_field_list_labels_275		1 Yes<br>2 No
rh_b_13 *(required)*	Kayakalp	1 Yes<br>2 No
rh_b_14 *(required)*	NQAS	1 Yes<br>2 No
rh_b_16 *(required)*	SUMAN	1 Yes<br>2 No
RH > rh_infra3		
rh_b_17 *(required)*	Emergency area and Triage services available	1 Yes<br>2 No<br>9999 Not Applicable
rh_b_17_or	Available 24x7?	1 Yes<br>2 No
rh_b_18 *(required)*	Gynec/Obstetrics OPD Services	1 Yes<br>2 No
rh_b_20 *(required)*	Laboratory Services	1 Yes<br>2 No
rh_b_21 *(required)*	Pediatrics OPD Room Services	1 Yes<br>2 No
rh_b_23 *(required)*	Psychologist OPD Room Services	1 Yes<br>2 No
rh_b_25 *(required)*	Lactation Counsellor Room Services	1 Yes

Field	Question	Answer	
		2	No
rh_b_27 *(required)*	Dietitian/ Nutrition Counsellor Room Services	1	Yes
		2	No
rh_b_30 *(required)*	ANC Room Services	1	Yes
		2	No
rh_b_32 *(required)*	Labour Room Services	1	Yes
		2	No
RH > rh_infra_room			
rh_b_19 *(required)*	Gynec/Obstetrics OPD services OPD services - Available failities<br>*Question relevant when: ${rh_b_18} =1*<br>*Response constrained to: not(selected(${rh_b_19} , '9999') and count-selected(${rh_b_19}) > 1)*	1	Adequate table, chairs, bench and stool
		5	Footstep
		3	Handwashing Station with Running Water Supply
		4	Waiting area with sitting facility
		9999	None
rh_b_22 *(required)*	Pediatrics OPD Room Services - Available facilities<br>*Question relevant when: ${rh_b_21} =1*<br>*Response constrained to: not(selected(${rh_b_22} , '9999') and count-selected(${rh_b_22}) > 1)*	1	Adequate table, chairs, bench and stool
		5	Footstep
		3	Handwashing Station with Running Water Supply
		4	Waiting area with sitting facility
		9999	None
rh_b_24 *(required)*	Psychologist OPD Room Services - Available Facilities<br>*Question relevant when: ${rh_b_23} =1*<br>*Response constrained to: not(selected(${rh_b_24} , '9999') and count-selected(${rh_b_24}) > 1)*	1	Adequate table, chairs, bench and stool
		2	Examination table
		3	Footstep
		4	Handwashing Station with Running Water Supply
		5	Waiting area with sitting facility
		9999	None
rh_b_26 *(required)*	Lactation Counsellor Room Services - Available Facilities<br>*Question relevant when: ${rh_b_25} =1*<br>*Response constrained to: not(selected(${rh_b_26} , '9999') and count-selected(${rh_b_26}) > 1)*	1	Adequate table, chairs, bench and stool
		2	Examination table
		3	Footstep
		4	Handwashing Station with Running Water Supply
		5	Waiting area with sitting facility
		9999	None
rh_b_28 *(required)*	Dietitian/ Nutrition Counsellor Room Services - Available Facilities<br>*Question relevant when: ${rh_b_27} =1*<br>*Response constrained to: not(selected(${rh_b_28} , '9999') and count-selected(${rh_b_28}) > 1)*	1	Adequate table, chairs, bench and stool
		2	Examination table
		3	Footstep
		4	Handwashing Station with Running Water Supply
		5	Waiting area with sitting facility
		9999	None
rh_b_29 *(required)*	Laboratory Services - Available failities<br>*Question relevant when: ${rh_b_20} =1*<br>*Response constrained to: not(selected(${rh_b_29} , '9999') and count-selected(${rh_b_29}) > 1)*	1	Adequate table, chairs, bench and stool
		2	Washbasin with Running Water Supply
		3	Adequate area for sample collection area
		4	Adequate areas for carrying out diagnostic activities
		5	Adequate area for keeping equipment and storage of

Field	Question	Answer
		reagents and records
		9999 None of the above
rh_b_31 *(required)*	ANC Room Services - Available failities<br>*Question relevant when: ${rh_b_30} =1*<br>*Response constrained to: not(selected(${rh_b_31} , '9999') and count-selected(${rh_b_31}) > 1)*	1 Adequate table, chairs, bench and stool<br>2 Examination table<br>3 Footstep<br>4 Handwashing Station with Running Water Supply<br>5 Waiting area with sitting facility<br>9999 None
rh_b_33 *(required)*	Labour Room Services - Available failities<br>*Question relevant when: ${rh_b_32} =1*<br>*Response constrained to: not(selected(${rh_b_33} , '9999') and count-selected(${rh_b_33}) > 1)*	1 Adequate labour table<br>2 Washbasin with Running Water Supply<br>3 Area embarked for Newborn care corner<br>4 Elbow tap<br>9999 None
RH > Labour Room Infrastructure Status		
rh_b_34 *(required)*	Post natal care area and beds<br>*If Yes, total number of beds available*	1 Yes<br>2 No
rh_b_35 *(required)*	Pediatric ward<br>*If Yes, total number of beds available*	1 Yes<br>2 No
rh_b_36 *(required)*	Special Newborn Care Unit (SNCU)	1 Yes<br>2 No
rh_b_37 *(required)*	Neonatal Intensive Care Unit (NICU)	1 Yes<br>2 No
rh_b_38 *(required)*	Gynae/Obstetrics ward<br>*If Yes, total number of beds available*	1 Yes<br>2 No
RH > Nutrition Rehabilitation Center (NRC)		
rh_b_44 *(required)*	Nutrition Rehabilitation Center (NRC) : Available Facilities<br>*Response constrained to: not(selected(${rh_b_44} , '9999') and count-selected(${rh_b_44}) > 1)*	1 Play Area<br>2 Couselling Area<br>3 Nursing Station<br>4 Kitchen with Cooking Demonstration Aids<br>9999 None
rh_b_45 *(required)*	Blood Bank / Blood Storage Units	1 Yes<br>2 No
RH > Availability Status of Equipment at rhital		
rh_d_1 *(required)*	24x7 Emergency Services with Triage, Resuscitation & Stabilization	1 Yes<br>2 No
RH > Availability Status of Equipment at rhital > rh_triage<br>*Group relevant when: ${rh_d_1} =1*		
rh_d_2 *(required)*	Emergency Drug Tray<br>*If No, specify missing medicines*	1 Yes<br>2 No
rh_d_3 *(required)*	Oxygen Cylinder<br>*If yes, please specify number of functional equipments*	1 Yes<br>2 No
rh_d_171 *(required)*	O2 concentrator<br>*If yes, please specify number of functional equipments*	1 Yes<br>2 No
rh_d_4 *(required)*	Suction Machine (Electrical)<br>*If yes, please specify number of functional equipments*	1 Yes<br>2 No
rh_d_4_1 *(required)*	Suction Machine (Foot-operated)<br>*If yes, please specify number of functional equipments*	1 Yes<br>2 No
rh_d_5 *(required)*	Resuscitation equipment for adult<br>*If No, specify missing medicines*	1 Yes<br>2 No
rh_d_7 *(required)*	Open care/Radiant Warmer<br>*If yes, please specify number of functional equipments*	1 Yes<br>2 No
rh_d_8 *(required)*	Pulse Oximeter Adult<br>*If yes, please specify number of functional equipments*	1 Yes<br>2 No
rh_d_172 *(required)*	Pulse Oximeter Pediatric	1 Yes

Field	Question	Answer
	If yes, please specify number of functional equipments	2 No
rh_d_9 *(required)*	Self-Inflating Bag and Mask Adult<br>*If yes, please specify number of functional equipments*	1 Yes<br>2 No
rh_d_173 *(required)*	Self-Inflating Bag and Mask Child<br>*If yes, please specify number of functional equipments*	1 Yes<br>2 No
rh_d_174 *(required)*	Self-Inflating Bag and Mask Neonatal Size(0,1)<br>*If yes, please specify number of functional equipments*	1 Yes<br>2 No
rh_d_10 *(required)*	Laryngoscope and ET Intubation Tubes all Sizes<br>*If yes, please specify number of functional equipments*	1 Yes<br>2 No
rh_d_11 *(required)*	Defibrillators<br>*If yes, please specify number of functional equipments*	1 Yes<br>2 No
rh_d_12 *(required)*	ECG Machine<br>*If yes, please specify number of functional equipments*	1 Yes<br>2 No
rh_d_13 *(required)*	ANC Services	1 Yes<br>2 No
RH > Availability Status of Equipment at rhital > ANC Services<br>*Group relevant when: ${rh_d_13} =1*		
rh_d_14 *(required)*	Blood Pressure Monitor (Digital)<br>*If yes, please specify number of functional equipments*	1 Yes<br>2 No
rh_d_14_1 *(required)*	Blood Pressure Monitor (Manual)<br>*If yes, please specify number of functional equipments*	1 Yes<br>2 No
rh_d_15 *(required)*	Stethoscope<br>*If yes, please specify number of functional equipments*	1 Yes<br>2 No
rh_d_175 *(required)*	Thermometer (Digital)<br>*If yes, please specify number of functional equipments*	1 Yes<br>2 No
rh_d_16 *(required)*	Clinical Thermometer<br>*If yes, please specify number of functional equipments*	1 Yes<br>2 No
rh_d_17 *(required)*	Adult Weighing Scale (Digital)<br>*If yes, please specify number of functional equipments*	1 Yes<br>2 No
rh_d_17_1 *(required)*	Adult Weighing Scale (Analogue)<br>*If yes, please specify number of functional equipments*	1 Yes<br>2 No
rh_d_18 *(required)*	Stadiometer<br>*If yes, please specify number of functional equipments*	1 Yes<br>2 No
rh_d_19 *(required)*	Measuring Tape<br>*If yes, please specify number of functional equipments*	1 Yes<br>2 No
rh_d_20 *(required)*	Examination Lamp with White Light<br>*If yes, please specify number of functional equipments*	1 Yes<br>2 No
rh_d_20_1 *(required)*	Fetoscope<br>*If yes, please specify number of functional equipments*	1 Yes<br>2 No
rh_d_176 *(required)*	Fetal Doppler Monitor<br>*If yes, please specify number of functional equipments*	1 Yes<br>2 No
rh_d_20_2 *(required)*	Cusco's Speculum<br>*If yes, please specify number of functional equipments*	1 Yes<br>2 No
rh_d_177 *(required)*	Sims Speculum<br>*If yes, please specify number of functional equipments*	1 Yes<br>2 No
rh_d_21 *(required)*	Pulse oximeter (adult)<br>*If yes, please specify number of functional equipments*	1 Yes<br>2 No
rh_d_22 *(required)*	Gynae/Obstetrics OPD Service	1 Yes<br>2 No
RH > Availability Status of Equipment at rhital > Gynae/Obstetrics OPD Services<br>*Group relevant when: ${rh_d_22} =1*		
rh_d_23 *(required)*	Blood Pressure Monitor (Digital)<br>*If yes, please specify number of functional equipments*	1 Yes<br>2 No
rh_d_23_1 *(required)*	Blood Pressure Monitor (Manual)<br>*If yes, please specify number of functional equipments*	1 Yes<br>2 No
rh_d_24 *(required)*	Stethoscope<br>*If yes, please specify number of functional equipments*	1 Yes<br>2 No
rh_d_25 *(required)*	Thermometer<br>*If yes, please specify number of functional equipments*	1 Yes<br>2 No

Field	Question	Answer
rh_d_26 *(required)*	Adult Weighing Scale (Digital)<br>*If yes, please specify number of functional equipments*	1 Yes<br>2 No
rh_d_26_1 *(required)*	Adult Weighing Scale (Analog)<br>*If yes, please specify number of functional equipments*	1 Yes<br>2 No
rh_d_27 *(required)*	Stadiometer<br>*If yes, please specify number of functional equipments*	1 Yes<br>2 No
rh_d_28 *(required)*	Measuring Tape<br>*If yes, please specify number of functional equipments*	1 Yes<br>2 No
rh_d_29 *(required)*	Examination Lamp with White Light<br>*If yes, please specify number of functional equipments*	1 Yes<br>2 No
rh_d_30 *(required)*	Fetoscope<br>*If yes, please specify number of functional equipments*	1 Yes<br>2 No
rh_d_181 *(required)*	Fetal Doppler Monitor<br>*If yes, please specify number of functional equipments*	1 Yes<br>2 No
rh_d_31 *(required)*	Cusco's Speculum<br>*If yes, please specify number of functional equipments*	1 Yes<br>2 No
rh_d_182 *(required)*	Sims Speculum<br>*If yes, please specify number of functional equipments*	1 Yes<br>2 No
rh_d_32 *(required)*	Pulse oximeter (adult)<br>*If yes, please specify number of functional equipments*	1 Yes<br>2 No
rh_d_33 *(required)*	Pediatric OPD Service	1 Yes<br>2 No
RH > Availability Status of Equipment at rhital > Pediatric OPD Services<br>*Group relevant when: ${rh_d_33} =1*		
rh_d_34 *(required)*	Pediatric Stethoscope<br>*If yes, please specify number of functional equipments*	1 Yes<br>2 No
rh_d_35 *(required)*	Clinical Thermometer<br>*If yes, please specify number of functional equipments*	1 Yes<br>2 No
rh_d_178 *(required)*	Digital Thermometer<br>*If yes, please specify number of functional equipments*	1 Yes<br>2 No
rh_d_36 *(required)*	Infantometer<br>*If yes, please specify number of functional equipments*	1 Yes<br>2 No
rh_d_37 *(required)*	Baby Weighing Scale (Electronic)<br>*If yes, please specify number of functional equipments*	1 Yes<br>2 No
rh_d_37_1 *(required)*	Baby Weighing Scale (Manual)<br>*If yes, please specify number of functional equipments*	1 Yes<br>2 No
rh_d_38 *(required)*	Pulse oximeter (Pediatric)<br>*If yes, please specify number of functional equipments*	1 Yes<br>2 No
rh_d_39 *(required)*	Phototherapy machine<br>*If yes, please specify number of functional equipments*	1 Yes<br>2 No
rh_d_40 *(required)*	Pediatric Resuscitation Kit<br>*If yes, please specify number of functional equipments*	1 Yes<br>2 No
rh_d_41 *(required)*	Breast pump (Manual)<br>*If yes, please specify number of functional equipments*	1 Yes<br>2 No
rh_d_41_1 *(required)*	Breast pump (Electric)<br>*If yes, please specify number of functional equipments*	1 Yes<br>2 No
rh_d_190 *(required)*	Labour Room	1 Yes<br>2 No
RH > Availability Status of Equipment at rhital > Labour Room<br>*Group relevant when: ${rh_d_190} =1*		
rh_d_42 *(required)*	Foetal doppler<br>*If yes, please specify number of functional equipments*	1 Yes<br>2 No
rh_d_43 *(required)*	Cardiotocography (CTG) machine<br>*If yes, please specify number of functional equipments*	1 Yes<br>2 No
rh_d_44 *(required)*	Laryngoscopes (Led)<br>*If yes, please specify number of functional equipments*	1 Yes<br>2 No
rh_d_45 *(required)*	Laryngoscopes (Led) Both Straight And Curved for Pediatric and ET tubes<br>*If yes, please specify number of functional equipments*	1 Yes<br>2 No
rh_d_46 *(required)*	Suction Machine (Electrical)	1 Yes

Field	Question	Answer
	If yes, please specify number of functional equipments	2 No
rh_d_179 *(required)*	Suction Machine (foot-operated)<br>*If yes, please specify number of functional equipments*	1 Yes<br>2 No
rh_d_47 *(required)*	Examination light<br>*If yes, please specify number of functional equipments*	1 Yes<br>2 No
rh_d_48 *(required)*	Open care radiant warmer<br>*If yes, please specify number of functional equipments*	1 Yes<br>2 No
rh_d_49 *(required)*	Vacuum extractors<br>*If yes, please specify number of functional equipments*	1 Yes<br>2 No
rh_d_50 *(required)*	Pulse Oximeter (Adult and Pediatric Probes)<br>*If yes, please specify number of functional equipments*	1 Yes<br>2 No
rh_d_51 *(required)*	Self-Inflating Bag and Mask Neonatal Size(0,1)<br>*If yes, please specify number of functional equipments*	1 Yes<br>2 No
rh_d_53 *(required)*	NORMAL DELIVERY TRAY (equipments)<br>*If yes, please specify number of functional equipments*	1 Yes<br>2 No
rh_d_54 *(required)*	Episiotomy kit<br>*If yes, please specify number of functional equipments*	1 Yes<br>2 No
rh_d_55 *(required)*	Forceps delivery kit<br>*If yes, please specify number of functional equipments*	1 Yes<br>2 No
rh_d_56 *(required)*	Craniotomy kit<br>*If yes, please specify number of functional equipments*	1 Yes<br>2 No
rh_d_57 *(required)*	Crash Cart with emergency medicines<br>*If No, specify missing medicines*	1 Yes<br>2 No
rh_d_58 *(required)*	Feeding tube<br>*If yes, please specify number of functional equipments*	1 Yes<br>2 No
rh_d_60 *(required)*	Suction catheter<br>*If yes, please specify number of functional equipments*	1 Yes<br>2 No
rh_d_62 *(required)*	Cord clamps<br>*If yes, please specify number of functional equipments*	1 Yes<br>2 No
rh_d_77_1 *(required)*	Special Newborn Care Unit (SNCU)	1 Yes<br>2 No
RH > Availability Status of Equipment at rhital > Special Newborn Care Unit (SNCU)<br>*Group relevant when: ${rh_d_77_1} =1*		
rh_d_78 *(required)*	Open care Radiant warmer<br>*If yes, please specify number of functional equipments*	1 Yes<br>2 No
rh_d_79 *(required)*	Neonatal Resuscitation Equipment<br>*If yes, please specify number of functional equipments*	1 Yes<br>2 No
rh_d_80 *(required)*	Pulse oximeter with Neonatal Wrap up<br>*If yes, please specify number of functional equipments*	1 Yes<br>2 No
rh_d_81 *(required)*	Multi para Monitor with Neonatal leads<br>*If yes, please specify number of functional equipments*	1 Yes<br>2 No
rh_d_82 *(required)*	Syringe Pump<br>*If yes, please specify number of functional equipments*	1 Yes<br>2 No
rh_d_83 *(required)*	Phototherapy Single Surface LED<br>*If yes, please specify number of functional equipments*	1 Yes<br>2 No
rh_d_84 *(required)*	Nebulizer<br>*If yes, please specify number of functional equipments*	1 Yes<br>2 No
rh_d_85 *(required)*	Portable LED Standing light<br>*If yes, please specify number of functional equipments*	1 Yes<br>2 No
rh_d_86 *(required)*	Foot Operating Suction machine<br>*If yes, please specify number of functional equipments*	1 Yes<br>2 No
rh_d_87 *(required)*	BP Apparatus - Neonatal Cuff<br>*If yes, please specify number of functional equipments*	1 Yes<br>2 No
rh_d_88 *(required)*	Baby weighing scale –Digital<br>*If yes, please specify number of functional equipments*	1 Yes<br>2 No
rh_d_89 *(required)*	Infantometer<br>*If yes, please specify number of functional equipments*	1 Yes<br>2 No
rh_d_90 *(required)*	Transcutaneous Bilirubinometer<br>*If yes, please specify number of functional equipments*	1 Yes<br>2 No

Field	Question	Answer
rh_d_91 *(required)*	Bubble CPAP with compressor<br>*If yes, please specify number of functional equipments*	1 Yes<br>2 No
rh_d_92 *(required)*	Transport Incubator<br>*If yes, please specify number of functional equipments*	1 Yes<br>2 No
rh_d_93 *(required)*	Irradiance Meter<br>*If yes, please specify number of functional equipments*	1 Yes<br>2 No
rh_d_94 *(required)*	Vein Finder<br>*If yes, please specify number of functional equipments*	1 Yes<br>2 No
rh_d_95 *(required)*	Oxygen Hood<br>*If yes, please specify number of functional equipments*	1 Yes<br>2 No
rh_d_96 *(required)*	Ambu bags (neonatal & paediatrics)<br>*If yes, please specify number of functional equipments*	1 Yes<br>2 No
rh_d_97 *(required)*	Oxygen Manifold<br>*If yes, please specify number of functional equipments*	1 Yes<br>2 No
rh_d_97_1 *(required)*	Neonatal Intensive Care Unit (NICU)	1 Yes<br>2 No
RH > Availability Status of Equipment at rhital > Neonatal Intensive Care Unit (NICU)<br>*Group relevant when: ${rh_d_97_1} =1*		
rh_d_98 *(required)*	Radiant warmer<br>*If yes, please specify number of functional equipments*	1 Yes<br>2 No
rh_d_100 *(required)*	Neonatal Resuscitation Equipment<br>*If yes, please specify number of functional equipments*	1 Yes<br>2 No
rh_d_101 *(required)*	BP Apparatus - Neonatal Cuff<br>*If yes, please specify number of functional equipments*	1 Yes<br>2 No
rh_d_102 *(required)*	Neonatal Ventilator<br>*If yes, please specify number of functional equipments*	1 Yes<br>2 No
rh_d_103 *(required)*	Pulse Oximeter With Neonatal Wrap Up<br>*If yes, please specify number of functional equipments*	1 Yes<br>2 No
rh_d_104 *(required)*	Multi Para Monitor With Leads<br>*If yes, please specify number of functional equipments*	1 Yes<br>2 No
rh_d_106 *(required)*	Syringe Pump<br>*If yes, please specify number of functional equipments*	1 Yes<br>2 No
rh_d_107 *(required)*	Phototherapy Single Surface Led<br>*If yes, please specify number of functional equipments*	1 Yes<br>2 No
rh_d_108 *(required)*	Nebulizer<br>*If yes, please specify number of functional equipments*	1 Yes<br>2 No
rh_d_109 *(required)*	Portable Led Standing Light<br>*If yes, please specify number of functional equipments*	1 Yes<br>2 No
rh_d_110 *(required)*	Foot Operating Suction Machine<br>*If yes, please specify number of functional equipments*	1 Yes<br>2 No
rh_d_111 *(required)*	Infantometer<br>*If yes, please specify number of functional equipments*	1 Yes<br>2 No
rh_d_112 *(required)*	Transcutaneous Bilirubinometer<br>*If yes, please specify number of functional equipments*	1 Yes<br>2 No
rh_d_113 *(required)*	Bubble Cpap With Compressor<br>*If yes, please specify number of functional equipments*	1 Yes<br>2 No
rh_d_114 *(required)*	Transport Incubator<br>*If yes, please specify number of functional equipments*	1 Yes<br>2 No
rh_d_115 *(required)*	Irradiance Meter<br>*If yes, please specify number of functional equipments*	1 Yes<br>2 No
rh_d_116 *(required)*	Vein Finder<br>*If yes, please specify number of functional equipments*	1 Yes<br>2 No
rh_d_118 *(required)*	Oxygen Hood<br>*If yes, please specify number of functional equipments*	1 Yes<br>2 No
rh_d_119 *(required)*	Mechanical Ventilator for paediatric use<br>*If yes, please specify number of functional equipments*	1 Yes<br>2 No
rh_d_120 *(required)*	Disaster Trolley/Crash Cart with emergency medicines<br>*If yes, please specify number of functional equipments*	1 Yes<br>2 No
rh_d_121 *(required)*	Ambu Bags (Paediatrics)	1 Yes

Field	Question	Answer
	If yes, please specify number of functional equipments	2 No
rh_d_122 *(required)*	Pediatric ward	1 Yes<br>2 No
RH > Availability Status of Equipment at rhital > Pediatric ward<br>*Group relevant when: ${rh_d_122} =1*		
rh_d_123 *(required)*	Pediatric Stethoscope<br>*If yes, please specify number of functional equipments*	1 Yes<br>2 No
rh_d_124 *(required)*	Digital/Infrared Thermometer<br>*If yes, please specify number of functional equipments*	1 Yes<br>2 No
rh_d_125 *(required)*	Infantometer<br>*If yes, please specify number of functional equipments*	1 Yes<br>2 No
rh_d_126 *(required)*	Baby Weighing Scale (Electronic)<br>*If yes, please specify number of functional equipments*	1 Yes<br>2 No
rh_d_126_1 *(required)*	Baby Weighing Scale (Manual)<br>*If yes, please specify number of functional equipments*	1 Yes<br>2 No
rh_d_127 *(required)*	Pulse oximeter (Pediatric)<br>*If yes, please specify number of functional equipments*	1 Yes<br>2 No
rh_d_128 *(required)*	Phototherapy machine<br>*If yes, please specify number of functional equipments*	1 Yes<br>2 No
rh_d_129 *(required)*	Pediatric Resuscitation Kit<br>*If yes, please specify number of functional equipments*	1 Yes<br>2 No
rh_d_130 *(required)*	Breast pump (Manual)<br>*If yes, please specify number of functional equipments*	1 Yes<br>2 No
rh_d_130_1 *(required)*	Breast pump (Electric)<br>*If yes, please specify number of functional equipments*	1 Yes<br>2 No
rh_d_131 *(required)*	Gynae/Obstetrics Ward	1 Yes<br>2 No
RH > Availability Status of Equipment at rhital > Gynae/Obstetrics Ward<br>*Group relevant when: ${rh_d_131} =1*		
rh_d_132 *(required)*	Thermometer<br>*If yes, please specify number of functional equipments*	1 Yes<br>2 No
rh_d_133 *(required)*	BP Apparatus<br>*If yes, please specify number of functional equipments*	1 Yes<br>2 No
rh_d_134 *(required)*	Glucometer with sticks<br>*If yes, please specify number of functional equipments*	1 Yes<br>2 No
rh_d_135 *(required)*	Adult Weighing Scale (Digital)<br>*If yes, please specify number of functional equipments*	1 Yes<br>2 No
rh_d_135_1 *(required)*	Adult Weighing Scale (Analog)<br>*If yes, please specify number of functional equipments*	1 Yes<br>2 No
rh_d_136 *(required)*	Oxygen Cylinder<br>*If yes, please specify number of functional equipments*	1 Yes<br>2 No
rh_d_180 *(required)*	O2 concentrator<br>*If yes, please specify number of functional equipments*	1 Yes<br>2 No
rh_d_191 *(required)*	Immunization Services	1 Yes<br>2 No
RH > Availability Status of Equipment at rhital > Immunization Services<br>*Group relevant when: ${rh_d_191} =1*		
rh_d_157 *(required)*	ILR with Voltage Stabilizer<br>*If yes, please specify number of functional equipments*	1 Yes<br>2 No
rh_d_158 *(required)*	DF Small with Voltage Stabilizer<br>*If yes, please specify number of functional equipments*	1 Yes<br>2 No
rh_d_159 *(required)*	Freeze Tag (2 no per ILR bimonthly)<br>*If yes, please specify number of functional equipments*	1 Yes<br>2 No
rh_d_160 *(required)*	Thermometer<br>*If yes, please specify number of functional equipments*	1 Yes<br>2 No
rh_d_161 *(required)*	Vaccine Carriers with Ice Packs<br>*If yes, please specify number of functional equipments*	1 Yes<br>2 No
rh_d_162 *(required)*	Cold Box<br>*If yes, please specify number of functional equipments*	1 Yes<br>2 No

Field	Question	Answer
rh_d_163 *(required)*	Waste Disposable Twin Buckets<br>*If yes, please specify number of functional equipments*	1 Yes<br>2 No
RH > Availability Status of Equipment at rhital > Blood Bank / Blood Storage Units		
rh_d_170 *(required)*	Blood bag refrigerators with real-time temperature monitoring devices	1 Yes<br>2 No
lab_facility *(required)*	Is lab service available ?	1 Yes<br>2 No
lab_services *(required)*	Type of facility<br>*Question relevant when: ${lab_facility} =1*	1 Goverment<br>2 KRSNAA/Empanelled
RH > Availability Status of Diagnostic tests/services at rh<br>*Group relevant when: selected(${lab_services} ,1) or selected(${lab_services} ,2)*		
rh_e_1 *(required)*	Hemoglobin - Required Equipment & Reagents are available in the Laboratory?<br>*If Yes, what method was used?*	1 Tested in facility<br>3 Only sample collection<br>2 Not Available
rh_e_2 *(required)*	Platelet Count<br>*If Yes, what method was used?*	1 Tested in facility<br>3 Only sample collection<br>2 Not Available
rh_e_3 *(required)*	Complete Blood Count with ESR<br>*If Yes, what method was used?*	1 Tested in facility<br>3 Only sample collection<br>2 Not Available
rh_e_4 *(required)*	Blood Group And Rh Typing<br>*If No, what other method was used?*	1 Tested in facility<br>3 Only sample collection<br>2 Not Available
rh_e_5 *(required)*	Blood Cross Matching<br>*If Yes, what method was used?*	1 Tested in facility<br>3 Only sample collection<br>2 Not Available
rh_e_6 *(required)*	Bleeding Time and Clotting Time<br>*If Yes, what method was used?*	1 Tested in facility<br>3 Only sample collection<br>2 Not Available
rh_e_7 *(required)*	MP slide method<br>*If Yes, what other method was used?*	1 Tested in facility<br>3 Only sample collection<br>2 Not Available
rh_e_8 *(required)*	Malaria rapid test<br>*If Yes, what other method was used?*	1 Tested in facility<br>3 Only sample collection<br>2 Not Available
rh_e_9 *(required)*	Human chorionic gonadotropin (HCG) (Urine test for pregnancy)<br>*If Yes, what other method was used?*	1 Yes<br>2 No
rh_e_10 *(required)*	Urine Microscopy<br>*If Yes, what other method was used?*	1 Tested in facility<br>3 Only sample collection<br>2 Not Available
rh_e_11 *(required)*	Urine for microalbumin<br>*If Yes, what other method was used?*	1 Tested in facility<br>3 Only sample collection<br>2 Not Available
rh_e_28 *(required)*	Urine for Culture<br>*If Yes, what other method was used?*	1 Tested in facility<br>3 Only sample collection<br>2 Not Available
rh_e_12 *(required)*	Stool for ova and cyst<br>*If Yes, what method was used?*	1 Tested in facility<br>3 Only sample collection<br>2 Not Available
rh_e_13 *(required)*	Stool for Occult Blood<br>*If Yes, what method was used?*	1 Tested in facility<br>3 Only sample collection<br>2 Not Available
rh_e_14 *(required)*	RPR/VDRL test for syphilis<br>*If Yes, what method was used?*	1 Tested in facility<br>3 Only sample collection<br>2 Not Available
rh_e_15 *(required)*	HIV test (Antibodies 1/2 and HIV 1/2)<br>*If Yes, what method was used?*	1 Tested in facility<br>3 Only sample collection<br>2 Not Available
rh_e_16 *(required)*	Hepatitis B surface antigen test<br>*If Yes, what method was used?*	1 Tested in facility

Field	Question	Answer
		3 Only sample collection<br>2 Not Available
rh_e_17 *(required)*	HCV Antibody Test (Anti HCV)<br>*If Yes, what method was used?*	1 Tested in facility<br>3 Only sample collection<br>2 Not Available
rh_e_19 *(required)*	Sputum, pus etc. for AFB<br>*If Yes, what method was used?*	1 Tested in facility<br>3 Only sample collection<br>2 Not Available
rh_e_20 *(required)*	Typhoid test (IgM)<br>*If Yes, what method was used?*	1 Tested in facility<br>3 Only sample collection<br>2 Not Available
rh_e_21 *(required)*	Blood sugar<br>*If Yes, what method was used?*	1 Tested in facility<br>3 Only sample collection<br>2 Not Available
rh_e_22 *(required)*	Oral Glucose Tolerance Test (OGTT)<br>*If Yes, what method was used?*	1 Tested in facility<br>3 Only sample collection<br>2 Not Available
rh_e_23 *(required)*	Wet mount and Gram stain for RTI/STD<br>*If Yes, what other method was used?*	1 Tested in facility<br>3 Only sample collection<br>2 Not Available
rh_e_24 *(required)*	S. TSH (including for newborn screening) also include T3,T4 , TSH FOR PW<br>*If Yes, what method was used?*	1 Tested in facility<br>3 Only sample collection<br>2 Not Available
rh_e_25 *(required)*	STI/RTI<br>*If Yes, what method was used?*	1 Tested in facility<br>3 Only sample collection<br>2 Not Available
rh_e_26 *(required)*	TB - NAAT<br>*If Yes, what method was used?*	1 Tested in facility<br>3 Only sample collection<br>2 Not Available
rh_e_27 *(required)*	Ferritin<br>*If Yes, what method was used?*	1 Tested in facility<br>3 Only sample collection<br>2 Not Available
rh_e_29 *(required)*	HPLC<br>*If Yes, what method was used?*	1 Tested in facility<br>3 Only sample collection<br>2 Not Available
rh_e_30 *(required)*	HbA1c<br>*If Yes, what method was used?*	1 Tested in facility<br>3 Only sample collection<br>2 Not Available
rh_e_31 *(required)*	Dengue Test<br>*If Yes, what method was used?*	1 Tested in facility<br>3 Only sample collection<br>2 Not Available
rh_e_32 *(required)*	Urine Dipstick Test<br>*If Yes, what method was used?*	1 Tested in facility<br>3 Only sample collection<br>2 Not Available
rh_e_24_remarks	Remarks (if any)	
RH > rh_iec		
generated_table_list_label_696	Availability Status of IEC Material/algorithms and management charts related to the national/state programs in local script.	
reserved_name_for_field_list_labels_697		1 Program being implemented<br>2 IEC material Available<br>3 IEC material displayed<br>4 Program not being implemented
rh_f_1 *(required)*	Family Planning- Mission Parivar Vikas	1 Program being implemented<br>2 IEC material Available<br>3 IEC material displayed<br>4 Program not being implemented
rh_f_2 *(required)*	Anemia- AMB charts and algorithms	1 Program being implemented<br>2 IEC material Available<br>3 IEC material displayed<br>4 Program not being implemented

**Field**	**Question**	**Answer**
rh_f_3 *(required)*	Adolescent health programs	1 Program being implemented<br>2 IEC material Available<br>3 IEC material displayed<br>4 Program not being implemented
rh_f_4 *(required)*	Nutrition- POSHAN Abhiyan	1 Program being implemented<br>2 IEC material Available<br>3 IEC material displayed<br>4 Program not being implemented
rh_f_5 *(required)*	Sanitation and Hygiene: Swachh Bharat Mission Gramin, handwashing	1 Program being implemented<br>2 IEC material Available<br>3 IEC material displayed<br>4 Program not being implemented
rh_f_6 *(required)*	ANC	1 Program being implemented<br>2 IEC material Available<br>3 IEC material displayed<br>4 Program not being implemented
rh_f_7 *(required)*	PMSMA	1 Program being implemented<br>2 IEC material Available<br>3 IEC material displayed<br>4 Program not being implemented
rh_f_8 *(required)*	Management of PPH, Shock	1 Program being implemented<br>2 IEC material Available<br>3 IEC material displayed<br>4 Program not being implemented
rh_f_9 *(required)*	Newborn Resuscitation, NSSK	1 Program being implemented<br>2 IEC material Available<br>3 IEC material displayed<br>4 Program not being implemented
rh_f_10 *(required)*	HBNC	1 Program being implemented<br>2 IEC material Available<br>3 IEC material displayed<br>4 Program not being implemented
rh_f_11 *(required)*	Childhood care- MAA, HBYC	1 Program being implemented<br>2 IEC material Available<br>3 IEC material displayed<br>4 Program not being implemented
rh_f_12 *(required)*	Immunization	1 Program being implemented<br>2 IEC material Available<br>3 IEC material displayed<br>4 Program not being implemented
rh_f_13 *(required)*	Breastfeeding	1 Program being implemented<br>2 IEC material Available<br>3 IEC material displayed<br>4 Program not being implemented
rh_f_14 *(required)*	Diarrhea management- Zinc, ORS	1 Program being implemented<br>2 IEC material Available<br>3 IEC material displayed<br>4 Program not being implemented
rh_f_15 *(required)*	Pneumonia Management	1 Program being implemented<br>2 IEC material Available<br>3 IEC material displayed<br>4 Program not being implemented
rh_f_16 *(required)*	JSY	1 Program being implemented<br>2 IEC material Available<br>3 IEC material displayed<br>4 Program not being implemented
rh_f_17 *(required)*	JSSK	1 Program being implemented<br>2 IEC material Available<br>3 IEC material displayed<br>4 Program not being implemented

Field	Question	Answer
rh_f_18 *(required)*	Ambulance (National Ambulance Services)	1 Program being implemented<br>2 IEC material Available<br>3 IEC material displayed<br>4 Program not being implemented
rh_f_19 *(required)*	Telemedicine	1 Program being implemented<br>2 IEC material Available<br>3 IEC material displayed<br>4 Program not being implemented
rh_f_20 *(required)*	Danger Signs (Maternal and Neonatal care)	1 Program being implemented<br>2 IEC material Available<br>3 IEC material displayed<br>4 Program not being implemented
RH > rh_register_group		
rh_f_10_r *(required)*	General OPD Register	1 Yes<br>2 No
rh_f_11_r *(required)*	Pre-conception Women Register	1 Yes<br>2 No
rh_f_12_r *(required)*	ANC Register	1 Yes<br>2 No
rh_f_13_r *(required)*	Pediatric/0-24 Months Infants & Children Register	1 Yes<br>2 No
rh_f_14_r *(required)*	Labour Room Register	1 Yes<br>2 No
rh_f_15_r *(required)*	Laboratory Record	1 Yes<br>2 No
rh_f_16_r *(required)*	Birth Register	1 Yes<br>2 No
rh_f_18_r *(required)*	Death Register	1 Yes<br>2 No
rh_f_17_r *(required)*	Referral Register	1 Yes<br>2 No
RH > rh_g_group		
RH > rh_g_group > rh_maintenance_record_data<br>*Group relevant when: ${rh_f_10_r} =1*		
generated_table_list_label_730	General OPD Register	
reserved_name_for_field_list_labels_731		1 Indicator is filled<br>2 Indicator Not filled<br>3 No indicator available
rh_g_1_1 *(required)*	Date	1 Indicator is filled<br>2 Indicator Not filled<br>3 No indicator available
rh_g_1_2 *(required)*	Patient ID	1 Indicator is filled<br>2 Indicator Not filled<br>3 No indicator available
rh_g_1_3 *(required)*	Name	1 Indicator is filled<br>2 Indicator Not filled<br>3 No indicator available
rh_g_1_4 *(required)*	Age	1 Indicator is filled<br>2 Indicator Not filled<br>3 No indicator available
rh_g_1_5 *(required)*	Sex	1 Indicator is filled<br>2 Indicator Not filled<br>3 No indicator available
rh_g_1_6 *(required)*	Address	1 Indicator is filled<br>2 Indicator Not filled<br>3 No indicator available
rh_g_1_13 *(required)*	Contact details	1 Indicator is filled<br>2 Indicator Not filled<br>3 No indicator available

Field	Question	Answer
rh_g_1_7 *(required)*	Marital status	1 Indicator is filled<br>2 Indicator Not filled<br>3 No indicator available
rh_g_1_8 *(required)*	Condition for which visiting OPD	1 Indicator is filled<br>2 Indicator Not filled<br>3 No indicator available
rh_g_1_9 *(required)*	Treatment provided	1 Indicator is filled<br>2 Indicator Not filled<br>3 No indicator available
rh_g_1_10 *(required)*	Investigations done	1 Indicator is filled<br>2 Indicator Not filled<br>3 No indicator available
rh_g_1_11 *(required)*	Follow-up visit date	1 Indicator is filled<br>2 Indicator Not filled<br>3 No indicator available
rh_g_1_12 *(required)*	Referrals made.	1 Indicator is filled<br>2 Indicator Not filled<br>3 No indicator available
RH > rh_g_group > rh_preconception_record_data<br>*Group relevant when: ${rh_f_11_r} =1*		
generated_table_list_label_745	Pre-conception Women Register	
reserved_name_for_field_list_labels_746		1 Indicator is filled<br>2 Indicator Not filled<br>3 No indicator available
rh_g_2_1 *(required)*	Date of visit	1 Indicator is filled<br>2 Indicator Not filled<br>3 No indicator available
rh_g_2_2 *(required)*	Woman ID	1 Indicator is filled<br>2 Indicator Not filled<br>3 No indicator available
rh_g_2_3 *(required)*	Name	1 Indicator is filled<br>2 Indicator Not filled<br>3 No indicator available
rh_g_2_4 *(required)*	Age	1 Indicator is filled<br>2 Indicator Not filled<br>3 No indicator available
rh_g_2_5 *(required)*	Address	1 Indicator is filled<br>2 Indicator Not filled<br>3 No indicator available
rh_g_2_11 *(required)*	Contact details	1 Indicator is filled<br>2 Indicator Not filled<br>3 No indicator available
rh_g_2_6 *(required)*	Height	1 Indicator is filled<br>2 Indicator Not filled<br>3 No indicator available
rh_g_2_7 *(required)*	Weight	1 Indicator is filled<br>2 Indicator Not filled<br>3 No indicator available
rh_g_2_8 *(required)*	BMI	1 Indicator is filled<br>2 Indicator Not filled<br>3 No indicator available
rh_g_2_9 *(required)*	Medical history (e.g.Anemia,DM,HT,RTI,Thyroid) and screening results of the same conditions	1 Indicator is filled<br>2 Indicator Not filled<br>3 No indicator available
rh_g_2_10 *(required)*	Place/facility were the conditions tracked during follow up visits	1 Indicator is filled<br>2 Indicator Not filled<br>3 No indicator available
RH > rh_g_group > rh_anc_record_data<br>*Group relevant when: ${rh_f_12_r} =1*		
generated_table_list_label_758	ANC Register	

Field	Question	Answer
reserved_name_for_field_list_labels_759		1 Indicator is filled<br>2 Indicator Not filled<br>3 No indicator available
rh_g_3_1 *(required)*	Date of visit	1 Indicator is filled<br>2 Indicator Not filled<br>3 No indicator available
rh_g_3_2 *(required)*	Woman ID	1 Indicator is filled<br>2 Indicator Not filled<br>3 No indicator available
rh_g_3_3 *(required)*	Name	1 Indicator is filled<br>2 Indicator Not filled<br>3 No indicator available
rh_g_3_4 *(required)*	Age	1 Indicator is filled<br>2 Indicator Not filled<br>3 No indicator available
rh_g_3_5 *(required)*	Address	1 Indicator is filled<br>2 Indicator Not filled<br>3 No indicator available
rh_g_3_18 *(required)*	Contact details	1 Indicator is filled<br>2 Indicator Not filled<br>3 No indicator available
rh_g_3_6 *(required)*	Gestational age (GA) at first registration	1 Indicator is filled<br>2 Indicator Not filled<br>3 No indicator available
rh_g_3_7 *(required)*	BMI in first trimester	1 Indicator is filled<br>2 Indicator Not filled<br>3 No indicator available
rh_g_3_16 *(required)*	Height	1 Indicator is filled<br>2 Indicator Not filled<br>3 No indicator available
rh_g_3_17 *(required)*	Weight	1 Indicator is filled<br>2 Indicator Not filled<br>3 No indicator available
rh_g_3_8 *(required)*	Records/columns of all ANC visits (no. of columns) along with provision to track GWG & Hb	1 Indicator is filled<br>2 Indicator Not filled<br>3 No indicator available
rh_g_3_9 *(required)*	Blood Pressure	1 Indicator is filled<br>2 Indicator Not filled<br>3 No indicator available
rh_g_3_10 *(required)*	Blood investigation- CBC at the time of registration	1 Indicator is filled<br>2 Indicator Not filled<br>3 No indicator available
rh_g_3_19 *(required)*	Blood investigation- TSH at the time of registration	1 Indicator is filled<br>2 Indicator Not filled<br>3 No indicator available
rh_g_3_11 *(required)*	OGTT- 1 time	1 Indicator is filled<br>2 Indicator Not filled<br>3 No indicator available
rh_g_3_12 *(required)*	Hb-4 times	1 Indicator is filled<br>2 Indicator Not filled<br>3 No indicator available
rh_g_3_13 *(required)*	Urine routine & microscopy- 4 times	1 Indicator is filled<br>2 Indicator Not filled<br>3 No indicator available
rh_g_3_14 *(required)*	Ultrasound	1 Indicator is filled<br>2 Indicator Not filled<br>3 No indicator available
rh_g_3_15 *(required)*	Td vaccination	1 Indicator is filled<br>2 Indicator Not filled

Field	Question	Answer
		3 No indicator available
RH > rh_g_group > rh_anc_record_ped<br>*Group relevant when: ${rh_f_13_r} =1*		
generated_table_list_label_779	Pediatric/0-24 Months Infants & Children Register	
reserved_name_for_field_list_labels_780		1 Indicator is filled<br>2 Indicator Not filled<br>3 No indicator available
rh_g_4_1 *(required)*	Date of visit	1 Indicator is filled<br>2 Indicator Not filled<br>3 No indicator available
rh_g_4_2 *(required)*	Child ID	1 Indicator is filled<br>2 Indicator Not filled<br>3 No indicator available
rh_g_4_3 *(required)*	Name	1 Indicator is filled<br>2 Indicator Not filled<br>3 No indicator available
rh_g_4_4 *(required)*	Age	1 Indicator is filled<br>2 Indicator Not filled<br>3 No indicator available
rh_g_4_5 *(required)*	Sex	1 Indicator is filled<br>2 Indicator Not filled<br>3 No indicator available
rh_g_4_6 *(required)*	Mother Name	1 Indicator is filled<br>2 Indicator Not filled<br>3 No indicator available
rh_g_4_7 *(required)*	Father Name	1 Indicator is filled<br>2 Indicator Not filled<br>3 No indicator available
rh_g_4_8 *(required)*	Address	1 Indicator is filled<br>2 Indicator Not filled<br>3 No indicator available
rh_g_4_17 *(required)*	Contact details	1 Indicator is filled<br>2 Indicator Not filled<br>3 No indicator available
rh_g_4_9 *(required)*	Weight	1 Indicator is filled<br>2 Indicator Not filled<br>3 No indicator available
rh_g_4_10 *(required)*	Height	1 Indicator is filled<br>2 Indicator Not filled<br>3 No indicator available
rh_g_4_11 *(required)*	Tracking of IWG	1 Indicator is filled<br>2 Indicator Not filled<br>3 No indicator available
rh_g_4_12 *(required)*	Identification of danger signs	1 Indicator is filled<br>2 Indicator Not filled<br>3 No indicator available
rh_g_4_13 *(required)*	Morbidities Reported	1 Indicator is filled<br>2 Indicator Not filled<br>3 No indicator available
rh_g_4_14 *(required)*	Treatment Provided	1 Indicator is filled<br>2 Indicator Not filled<br>3 No indicator available
rh_g_4_15 *(required)*	Immunisation Status	1 Indicator is filled<br>2 Indicator Not filled<br>3 No indicator available
rh_g_4_16 *(required)*	Follow-up visit Date	1 Indicator is filled<br>2 Indicator Not filled<br>3 No indicator available
RH > rh_g_group > rh_labour_room_record_data<br>*Group relevant when: ${rh_f_14_r} =1*		

Field	Question	Answer
generated_table_list_label_798	Labour Room Register	
reserved_name_for_field_list_labels_799		1 Indicator is filled<br>2 Indicator Not filled<br>3 No indicator available
rh_g_5_1 *(required)*	Date of admission	1 Indicator is filled<br>2 Indicator Not filled<br>3 No indicator available
rh_g_5_2 *(required)*	Patient ID	1 Indicator is filled<br>2 Indicator Not filled<br>3 No indicator available
rh_g_5_3 *(required)*	Name	1 Indicator is filled<br>2 Indicator Not filled<br>3 No indicator available
rh_g_5_4 *(required)*	Age	1 Indicator is filled<br>2 Indicator Not filled<br>3 No indicator available
rh_g_5_5 *(required)*	Address	1 Indicator is filled<br>2 Indicator Not filled<br>3 No indicator available
rh_g_5_13 *(required)*	Contact details	1 Indicator is filled<br>2 Indicator Not filled<br>3 No indicator available
rh_g_5_6 *(required)*	Time of admission	1 Indicator is filled<br>2 Indicator Not filled<br>3 No indicator available
rh_g_5_7 *(required)*	Type of delivery (e.g. normal,cesarean)	1 Indicator is filled<br>2 Indicator Not filled<br>3 No indicator available
rh_g_5_8 *(required)*	Complications during delivery	1 Indicator is filled<br>2 Indicator Not filled<br>3 No indicator available
rh_g_5_9 *(required)*	Birth weight	1 Indicator is filled<br>2 Indicator Not filled<br>3 No indicator available
rh_g_5_10 *(required)*	APGAR score	1 Indicator is filled<br>2 Indicator Not filled<br>3 No indicator available
rh_g_5_11 *(required)*	Time of discharge	1 Indicator is filled<br>2 Indicator Not filled<br>3 No indicator available
rh_g_5_12 *(required)*	Referral information.	1 Indicator is filled<br>2 Indicator Not filled<br>3 No indicator available
RH > rh_g_group > rh_lab_record<br>*Group relevant when: ${rh_f_15_r} =1*		
generated_table_list_label_813	Laboratory Record	
reserved_name_for_field_list_labels_814		1 Indicator is filled<br>2 Indicator Not filled<br>3 No indicator available
rh_g_6_1 *(required)*	Date	1 Indicator is filled<br>2 Indicator Not filled<br>3 No indicator available
rh_g_6_2 *(required)*	Sample ID	1 Indicator is filled<br>2 Indicator Not filled<br>3 No indicator available
rh_g_6_3 *(required)*	Patient ID	1 Indicator is filled<br>2 Indicator Not filled<br>3 No indicator available
rh_g_6_4 *(required)*	Name	1 Indicator is filled

Field	Question	Answer
		2 Indicator Not filled<br>3 No indicator available
rh_g_6_5 *(required)*	Type of investigation	1 Indicator is filled<br>2 Indicator Not filled<br>3 No indicator available
rh_g_6_6 *(required)*	Investigation results	1 Indicator is filled<br>2 Indicator Not filled<br>3 No indicator available
rh_g_6_7 *(required)*	Remarks	1 Indicator is filled<br>2 Indicator Not filled<br>3 No indicator available
rh_g_6_8 *(required)*	Date of report	1 Indicator is filled<br>2 Indicator Not filled<br>3 No indicator available
RH > rh_g_group > rh_birth_record<br>*Group relevant when: ${rh_f_16_r} =1*		
generated_table_list_label_823	Birth Register	
reserved_name_for_field_list_labels_824		1 Indicator is filled<br>2 Indicator Not filled<br>3 No indicator available
rh_g_7_1 *(required)*	Date of event	1 Indicator is filled<br>2 Indicator Not filled<br>3 No indicator available
rh_g_7_2 *(required)*	Child ID	1 Indicator is filled<br>2 Indicator Not filled<br>3 No indicator available
rh_g_7_3 *(required)*	Name of child (if applicable)	1 Indicator is filled<br>2 Indicator Not filled<br>3 No indicator available
rh_g_7_4 *(required)*	Mother's Name	1 Indicator is filled<br>2 Indicator Not filled<br>3 No indicator available
rh_g_7_5 *(required)*	Father's Name	1 Indicator is filled<br>2 Indicator Not filled<br>3 No indicator available
rh_g_7_6 *(required)*	Date of birth	1 Indicator is filled<br>2 Indicator Not filled<br>3 No indicator available
rh_g_7_7 *(required)*	Place of birth	1 Indicator is filled<br>2 Indicator Not filled<br>3 No indicator available
rh_g_7_9 *(required)*	Attendant details (e.g., ASHA, AWW)	1 Indicator is filled<br>2 Indicator Not filled<br>3 No indicator available
rh_g_7_10 *(required)*	Remarks	1 Indicator is filled<br>2 Indicator Not filled<br>3 No indicator available
RH > rh_g_group > rh_death_record<br>*Group relevant when: ${rh_f_18_r} =1*		
generated_table_list_label_834	Death Register	
reserved_name_for_field_list_labels_835		1 Indicator is filled<br>2 Indicator Not filled<br>3 No indicator available
rh_g_7_1_1 *(required)*	Date of event	1 Indicator is filled<br>2 Indicator Not filled<br>3 No indicator available
rh_g_7_2_1 *(required)*	Child ID	1 Indicator is filled<br>2 Indicator Not filled<br>3 No indicator available
rh_g_7_3_1 *(required)*	Name of child (if applicable)	1 Indicator is filled

Field	Question	Answer
		2 Indicator Not filled<br>3 No indicator available
rh_g_7_4_1 *(required)*	Mother's Name	1 Indicator is filled<br>2 Indicator Not filled<br>3 No indicator available
rh_g_7_5_1 *(required)*	Father's Name	1 Indicator is filled<br>2 Indicator Not filled<br>3 No indicator available
rh_g_7_6_1 *(required)*	Date of death	1 Indicator is filled<br>2 Indicator Not filled<br>3 No indicator available
rh_g_7_7_1 *(required)*	Place of death	1 Indicator is filled<br>2 Indicator Not filled<br>3 No indicator available
rh_g_7_8_1 *(required)*	Cause of death	1 Indicator is filled<br>2 Indicator Not filled<br>3 No indicator available
rh_g_7_9_1 *(required)*	Attendant details (e.g., ASHA, AWW)	1 Indicator is filled<br>2 Indicator Not filled<br>3 No indicator available
rh_g_7_10_1 *(required)*	Remarks	1 Indicator is filled<br>2 Indicator Not filled<br>3 No indicator available
RH > rh_g_group > rh_referral_record<br>*Group relevant when: ${rh_f_17_r} =1*		
generated_table_list_label_846	Referral Register	
reserved_name_for_field_list_labels_847		1 Indicator is filled<br>2 Indicator Not filled<br>3 No indicator available
rh_g_8_1 *(required)*	Date of referral	1 Indicator is filled<br>2 Indicator Not filled<br>3 No indicator available
rh_g_8_2 *(required)*	Patient ID	1 Indicator is filled<br>2 Indicator Not filled<br>3 No indicator available
rh_g_8_3 *(required)*	Name	1 Indicator is filled<br>2 Indicator Not filled<br>3 No indicator available
rh_g_8_4 *(required)*	Address	1 Indicator is filled<br>2 Indicator Not filled<br>3 No indicator available
rh_g_8_5 *(required)*	Reason for referral	1 Indicator is filled<br>2 Indicator Not filled<br>3 No indicator available
rh_g_8_6 *(required)*	Referring facility	1 Indicator is filled<br>2 Indicator Not filled<br>3 No indicator available
rh_g_8_7 *(required)*	Receiving facility	1 Indicator is filled<br>2 Indicator Not filled<br>3 No indicator available
rh_g_8_8 *(required)*	Date of receiving feedback	1 Indicator is filled<br>2 Indicator Not filled<br>3 No indicator available
rh_g_8_9 *(required)*	Follow-up visit date	1 Indicator is filled<br>2 Indicator Not filled<br>3 No indicator available
RH > rh_i_group		
rh_i_1_1 *(required)*	Iron, Vitamin, and Nutritional Supplements<br>*Response constrained to: not(selected(${rh_i_1_1} , '9999') and count-selected(${rh_i_1_1}) > 1)*	1 Ferrous Salts equivalent to 60 mg of Elemental Iron

Field	Question	Answer
		2 Ferrous Salts Oral Liquid equivalent to 25 mg of Elemental Iron/ml<br>3 Ferrous Salt (A) + Folic Acid (B) Tablet 45 mg Elemental Iron (A) + 400 mcg Folic Acid (B)<br>4 Ferrous Salt (A) + Folic Acid (B) Tablet 60 mg Elemental Iron (A)+ 500 mcg Folic Acid (B)<br>5 Ferrous Salt (A) + Folic Acid (B) Oral Liqiud 20 mg Elemental Iron(A) + 100 mcg (B)/ml<br>6 Folic Acid Tablet 5 mg<br>7 Folic Acid Tablet 400 mcg<br>8 Folic Acid 5 mg Injection 1 mg/ml<br>9 Iron Sucrose Injection 20 mg/ml<br>10 Phytomenadione (Vitamin K1) Tablet 10 mg<br>11 Phytomenadione (Vitamin K1) Inj 10 mg/ml<br>12 Calcium with Vitamin D Tablets USP<br>13 Syrup Calcium with Phosphate in the ratio 2:1 with Vitamin D<br>14 Tablet Calcium Carbonate 650 mg equivalent to Elemental Calcium 250 mg and Cholecalciferol USP 125 mg<br>15 Cholecalciferol Tablet 1000 IU<br>16 Cholecalciferol Tablet 60,000 IU<br>17 Cholecalciferol Oral Liquid 400 IU/ml<br>18 Cholecalciferol Granules 60,000 IU in Sachet<br>19 Cholecalciferol Injection of 6,00,000 IU<br>20 Vitamin D3 60,000 Granules in 1 gm Sachet<br>21 Vitamin A Oral liquid 100000 IU/ml<br>22 Zinc Sulphate tablets<br>23 Multiple Micronutrients (MMN) (WINGS)<br>99 Other Medicine (Specify)<br>9999 None
RH > rh_i_group > [rh_i_1_cal1] (1)		(Repeated group)
RH > rh_i_group > [rh_i_1_cal1] (1) > Availability Status of Essential Medicines Required at rh-SHC for MCH Care [rh_i_1_cal1]}<br>*Group relevant when: ${rh_i_1_1} !=9999*		
rh_i_1_t *(required)*	Target beneficiary	1 Preconception women (18-35 Years)<br>2 Pregnant women<br>3 Postnatal/ lactating women<br>4 0-6 Months Infants<br>5 6-24 Months Infants & Children

Field	Question	Answer
rh_i_1_2 *(required)*	Projected Monthly Requirements	
rh_i_1_3 *(required)*	Current Numbers in Stock	
rh_i_1_4 *(required)*	Stock Out in the last 3 month	1 Yes<br>2 No
rh_i_2_1 *(required)*	Antibiotics<br>*Response constrained to: not(selected(${rh_i_2_1} , '9999') and count-selected(${rh_i_2_1}) > 1)*	1 Inj. Gentamicin 80 mg (40 mg/ml)<br>2 Injection Ampicillin<br>3 Injection Amikacin<br>4 Albendazole Tablet 400 mg<br>5 Albendazole Oral Liquid 200 mg/5 ml<br>6 Amoxicillin Capsule 250 mg<br>7 Amoxicillin Tablet 500 mg<br>8 Ampicillin Capsule 500 mg<br>9 Ampicillin Injection 500 mg/ml<br>10 Amoxicillin (500 mg) + Clavulanic Acid (125 mg) Tablet<br>11 Amoxicillin (875 mg) + Clavulanic Acid (125 mg) Tablet<br>12 Benzylpenicillin Injection 10 lakh unit<br>13 Cefixime Tablet 200 mg<br>14 Cefotaxime Injection 1 g/vial<br>15 Piperacillin (A) + Tazobactam (B) Powder for Injection 1 g (A) + 125 mg (B)/2 g (A) + 250 mg (B)/4 g (A) + 500 mg (B)<br>16 Azithromycin Tablet 250 mg/500 mg/Oral Liquid 200 mg/5 ml<br>17 Norfloxacin Tablet 400 mg<br>18 Ciprofloxacin Tablet 250 mg/500 mg/Oral Liquid 250 mg/5 ml<br>19 Ciprofloxacin I.V 200 mg/100 ml<br>20 Co-trimoxazole [Sulphamethoxazol e (A) +Trimethoprim (B)] Tablet 400 mg (A) + 80 mg (B)/Tablet 800 mg (A) + 160 mg (B) /Oral liquid 200 mg (A) + 40 mg (B)/5 ml<br>21 Doxycycline Capsule 100 mg<br>22 Metronidazole Tablet 200 mg, 400 mg<br>23 Metronidazole Injection 5 mg/ml, 500 mg/100 ml<br>24 Levofloxacin Tablet 250 mg/500 mg/750 mg<br>25 Tetracycline/Chloramphenicol eye drops<br>26 Atropine eye drops<br>27 Chloramphenicol<br>99 Other Medicine(Specify)<br>9999 None
RH > rh_i_group > [rh_i_2_cal1] (1)		(Repeated group)
RH > rh_i_group > [rh_i_2_cal1] (1) > Availability Status of Essential Medicines Required at rh-SHC for MCH Care [rh_i_2_cal1]}		

Field	Question	Answer
Group relevant when: ${rh_i_2_1} !=9999<br>rh_i_2_t *(required)*	Target beneficiary	1 Preconception women (18-35 Years)<br>2 Pregnant women<br>3 Postnatal/ lactating women<br>4 0-6 Months Infants<br>5 6-24 Months Infants & Children
rh_i_2_2 *(required)*	Projected Monthly Requirements	
rh_i_2_3 *(required)*	Current Numbers in Stock	
rh_i_2_4 *(required)*	Stock Out in the last 3 month	1 Yes<br>2 No
rh_i_3_1 *(required)*	Antihypertensives and Seizure Management<br>*Response constrained to: not(selected(${rh_i_3_1} , '9999') and count-selected(${rh_i_3_1}) > 1)*	1 Labetalol Injection 5 mg/ml/20 mg/2ml<br>2 Labetalol Tablet 100 mg<br>3 Methyldopa Tablet 250 mg/500 mg<br>4 Enalapril Tablet 2.5 mg/5 mg/10 mg<br>5 Tab Digoxin - Digoxin IP 250 ug/tab<br>6 Inj. Lorazepam 1mg/ml<br>7 Carbamazepine Tablet 100 mg, 200 mg<br>8 Lorazepam Tablet 1 mg<br>9 Lorazepam Injection 1 or 2 mg/ml<br>10 Magnesium Sulphate Injection 500 mg/ml<br>11 Magnesium Sulphate Injection IP 50% w/v<br>12 Phenytoin Tablet 50 mg/100 mg, 300 mg<br>13 Phenytoin Injection 25 mg/ml, 50 mg/ml<br>14 Sodium Valproate Tablet 100 mg/500 mg<br>15 Inj. Calcium Gluconate 1gm<br>99 Other Medicine(Specify)<br>9999 None
RH > rh_i_group > [rh_i_3_cal1] (1)		(Repeated group)
RH > rh_i_group > [rh_i_3_cal1] (1) > Availability Status of Essential Medicines Required at rh-SHC for MCH Care [rh_i_3_cal1]]<br>*Group relevant when: ${rh_i_3_1} !=9999*		
rh_i_3_t *(required)*	Target beneficiary	1 Preconception women (18-35 Years)<br>2 Pregnant women<br>3 Postnatal/ lactating women<br>4 0-6 Months Infants<br>5 6-24 Months Infants & Children
rh_i_3_2 *(required)*	Projected Monthly Requirements	
rh_i_3_3 *(required)*	Current Numbers in Stock	
rh_i_3_4 *(required)*	Stock Out in the last 3 month	1 Yes<br>2 No
rh_i_4_1 *(required)*	Analgesics and Antipyretics<br>*Response constrained to: not(selected(${rh_i_4_1} , '9999') and count-selected(${rh_i_4_1}) > 1)*	1 Diclofenac Tablet 50 mg<br>2 Diclofenac Injection 25 mg/ml<br>3 Diclofenac Injection 25 mg/3 ml<br>4 Ibuprofen Tablet 200 mg<br>5 Ibuprofen Tablet 400 mg<br>6 Ibuprofen Syrup 100 mg/5 ml<br>7 Paracetamol Tablet 500 mg/650 mg

Field	Question	Answer
		8 Paracetamol Injection 150 mg/ml<br>9 Paracetamol Syrup 125 mg/5 ml<br>10 Hydroxychloroquine Tablet 200 mg<br>11 Hydroxychloroquine Tablet 400 mg<br>99 Other Medicine (Specify)<br>9999 None
RH > rh_i_group > [rh_i_4_cal1] (1)		(Repeated group)
RH > rh_i_group > [rh_i_4_cal1] (1) > Availability Status of Essential Medicines Required at rh-SHC for MCH Care [rh_i_4_cal1]}<br>*Group relevant when: ${rh_i_4_1} !=9999*		
rh_i_4_t *(required)*	Target beneficiary	1 Preconception women (18-35 Years)<br>2 Pregnant women<br>3 Postnatal/ lactating women<br>4 0-6 Months Infants<br>5 6-24 Months Infants & Children
rh_i_4_2 *(required)*	Projected Monthly Requirements	
rh_i_4_3 *(required)*	Current Numbers in Stock	
rh_i_4_4 *(required)*	Stock Out in the last 3 month	1 Yes<br>2 No
rh_i_5_1 *(required)*	Anthelmintics<br>*Response constrained to: not(selected(${rh_i_5_1} , '9999') and count-selected(${rh_i_5_1}) > 1)*	1 Tab. Albendazole (Chewable) 400 mg/Tab<br>2 Albendazole Oral Solution 200 mg/5 ml<br>3 Oral Antimalarial<br>99 Other Medicine (Specify)<br>9999 None
RH > rh_i_group > [rh_i_5_cal1] (1)		(Repeated group)
RH > rh_i_group > [rh_i_5_cal1] (1) > Availability Status of Essential Medicines Required at rh-SHC for MCH Care [rh_i_5_cal1]}<br>*Group relevant when: ${rh_i_5_1} !=9999*		
rh_i_5_t *(required)*	Target beneficiary	1 Preconception women (18-35 Years)<br>2 Pregnant women<br>3 Postnatal/ lactating women<br>4 0-6 Months Infants<br>5 6-24 Months Infants & Children
rh_i_5_2 *(required)*	Projected Monthly Requirements	
rh_i_5_3 *(required)*	Current Numbers in Stock	
rh_i_5_4 *(required)*	Stock Out in the last 3 month	1 Yes<br>2 No
rh_i_6_1 *(required)*	Diabetic & Thyroid Medications<br>*Response constrained to: not(selected(${rh_i_6_1} , '9999') and count-selected(${rh_i_6_1}) > 1)*	1 Inj. Human Soluble Insulin (40 IU/ml)<br>2 Tab. Metformin HCl (Coated) (500 mg/Tab)<br>3 Tab. Eltroxin / Thyroxine (100 mcg)<br>99 Other Medicine (Specify)<br>9999 None
RH > rh_i_group > [rh_i_6_cal1] (1)		(Repeated group)
RH > rh_i_group > [rh_i_6_cal1] (1) > Availability Status of Essential Medicines Required at rh-SHC for MCH Care [rh_i_6_cal1]}<br>*Group relevant when: ${rh_i_6_1} !=9999*		
rh_i_6_t *(required)*	Target beneficiary	1 Preconception women (18-35 Years)<br>2 Pregnant women<br>3 Postnatal/ lactating women<br>4 0-6 Months Infants<br>5 6-24 Months Infants & Children
rh_i_6_2 *(required)*	Projected Monthly Requirements	

Field	Question	Answer
rh_i_6_3 *(required)*	Current Numbers in Stock	
rh_i_6_4 *(required)*	Stock Out in the last 3 month	1 Yes<br>2 No
rh_i_7_1 *(required)*	Other Miscellaneous<br>*Response constrained to: not(selected(${rh_i_7_1} , '9999') and count-selected(${rh_i_7_1}) > 1)*	1 Inj. Td (0.5 ml/Amp)<br>2 Tab. Digoxin (0.25 mg/Tab)<br>3 Tab. Frusemide (40 mg/Tab)<br>4 Inj. Frusemide (10 mg/1 ml)<br>5 Inj. Betamethasone Sod. Phosphate (4 mg/ml)<br>6 Inj. Human Anti-D Immunoglobulin (300 mcg/1.5 ml)<br>7 IV Fluids<br>8 ORS Sachet<br>9 Zinc Tablet<br>10 Povidone Iodine Ointment (5% w/v)<br>11 Sanitary Napkins<br>12 Vaccines (As per National Programme)<br>99 Other Medicine (Specify)<br>9999 None
RH > rh_i_group > [rh_i_7_cal1] (1)		(Repeated group)
RH > rh_i_group > [rh_i_7_cal1] (1) > Availability Status of Essential Medicines Required at rh-SHC for MCH Care [rh_i_7_cal1]}<br>*Group relevant when: ${rh_i_7_1} !=9999*		
rh_i_7_t *(required)*	Target beneficiary	1 Preconception women (18-35 Years)<br>2 Pregnant women<br>3 Postnatal/ lactating women<br>4 0-6 Months Infants<br>5 6-24 Months Infants & Children
rh_i_7_2 *(required)*	Projected Monthly Requirements	
rh_i_7_3 *(required)*	Current Numbers in Stock	
rh_i_7_4 *(required)*	Stock Out in the last 3 month	1 Yes<br>2 No
RH > rh_j_group		
RH > rh_j_group > Facility's Beneficiaries (Patient) Load		
rh_j_note	Please mention the Numbers (Last 12 Month Data) against each	
rh_j_1 *(required)*	Eligible couples	
rh_j_2 *(required)*	Preconception women (18-35 years)	
rh_j_3 *(required)*	Pregnant women	
rh_j_4 *(required)*	Postnatal women	
rh_j_5 *(required)*	0 to 6 months infants	
rh_j_6 *(required)*	6 to 12 months infants	
rh_j_7 *(required)*	12 to 24 months children	
rh_j_8 *(required)*	Preconception women (18-35 years) screened for medical conditions	
rh_j_9 *(required)*	Preconception women (18-35 years) screened for depressive symptoms-	
RH > rh_j_group > Preconception women (18-35 years) screened for medical conditions and depressive symptoms-<br>*Group relevant when: ${rh_j_2} != 0 and ${rh_j_2} != 999*		
rh_j_8_note	Please mention the Number of Preconception women (18-35 years) screened (Last 12 Month Data) against each	
rh_j_8_1 *(required)*	RTI/STI	
rh_j_8_2 *(required)*	TB	
rh_j_8_3 *(required)*	Epilepsy	
rh_j_8_4 *(required)*	Syphilis	
rh_j_8_5 *(required)*	Thyroid status	
rh_j_8_6 *(required)*	Blood pressure	
rh_j_8_7 *(required)*	Blood sugar	
rh_j_8_8 *(required)*	Height measured	
rh_j_8_9 *(required)*	Weight measured	
rh_j_8_10 *(required)*	Hemoglobin tested	

Field	Question	Answer
rh_j_8_11 *(required)*	PHQ 2	
RH > rh_j_group > Preconception women (aged 18-35 years) identified with medical conditions & depressive symptoms following screening-<br>*Group relevant when: ${rh_j_2} != 0 and ${rh_j_2} != 999*		
rh_j_9_note	Please mention the Number of Preconception women (aged 18-35 years) identified with medical conditions & depressive symptoms following screening (Last 12 Month Data) against each	
rh_j_9_1 *(required)*	RTI	
rh_j_9_1_1 *(required)*	STI	
rh_j_9_2 *(required)*	TB	
rh_j_9_3 *(required)*	Epilepsy	
rh_j_9_4 *(required)*	Syphilis	
rh_j_9_5 *(required)*	Hypothyroidism (TSH > 5.5 IU/mL)	
rh_j_9_6 *(required)*	Hyperthyroidism (TSH <0.4 IU/mL)	
rh_j_9_7 *(required)*	Hypertension	
rh_j_9_7_1 *(required)*	Hypotension	
rh_j_9_8 *(required)*	Prediabetes (HbA1c 5.7% to 6.4%)	
rh_j_9_9 *(required)*	Diabetes mellitus (HbA1c ≥6.5%)	
rh_j_9_10 *(required)*	BMI<16 kg/m2	
rh_j_9_11 *(required)*	BMI 16-18.49 kg/m2	
rh_j_9_12 *(required)*	BMI 18.5- <21 kg/m2	
rh_j_9_13 *(required)*	Severe anemia (Hb <7 g/dL)	
rh_j_9_14 *(required)*	Moderate anemia (Hb 7 to 11.99 g/dL)	
rh_j_9_15 *(required)*	Depressive symptoms	
RH > rh_j_group > Preconception women (aged 18-35 years) managed<br>*Group relevant when: ${rh_j_2} != 0 and ${rh_j_2} != 999*		
rh_j_10_note	Please mention the Number of Preconception women (aged 18-35 years) managed for (Last 12 Month Data) the following conditions	
rh_j_10_1 *(required)*	RTI	
rh_j_10_1_1 *(required)*	STI	
rh_j_10_2 *(required)*	TB	
rh_j_10_3 *(required)*	Epilepsy	
rh_j_10_4 *(required)*	Syphilis	
rh_j_10_5 *(required)*	Hypothyroidism (TSH > 5.5 IU/mL)	
rh_j_10_6 *(required)*	Hyperthyroidism (TSH <0.4 IU/mL)	
rh_j_10_7 *(required)*	Hypertension	
rh_j_10_7_1 *(required)*	Hypotension	
rh_j_10_8 *(required)*	Prediabetes (HbA1c 5.7% to 6.4%)	
rh_j_10_9 *(required)*	Diabetes mellitus (HbA1c ≥6.5%)	
rh_j_10_10 *(required)*	BMI<16 kg/m2	
rh_j_10_11 *(required)*	BMI 16-18.49 kg/m2	
rh_j_10_12 *(required)*	BMI 18.5- <21 kg/m2	
rh_j_10_13 *(required)*	Severe anemia (Hb <7 g/dL)	
rh_j_10_14 *(required)*	Moderate anemia (Hb 7 to 11.99 g/dL)	
rh_j_10_15 *(required)*	Depressive symptoms	
rh_j_10_16 *(required)*	No. of preconception women who were given IFA as per recommendation	
rh_j_10_17 *(required)*	No. of preconception women who counselled for Nutrition	
rh_j_10_18 *(required)*	No. of preconception women who counselled for WASH intervention	
rh_j_11 *(required)*	Number of Pregnant Women Registered for ANC Care in the last 12 months	
RH > rh_j_group > Pregnant women screened/tested for -<br>*Group relevant when: ${rh_j_3} != 0 and ${rh_j_3} != 999*		
rh_j_11_note	Please mention the Number of Pregnant women screened/tested for (Last 12 Month Data)	
rh_j_11_1 *(required)*	Blood grouping	
rh_j_11_2 *(required)*	Complete Blood Count (CBC) at the time of registration	
rh_j_11_3 *(required)*	HIV	
rh_j_11_4 *(required)*	Syphilis	
rh_j_11_5 *(required)*	Urine routine & microscopy	
rh_j_11_6 *(required)*	Thyroid status / TSH	
rh_j_11_7 *(required)*	Gestational Diabetes Mellitus (GDM) / OGTT	
rh_j_11_8 *(required)*	Blood pressure	
rh_j_11_9 *(required)*	Height measured in First Trimester	
rh_j_11_10 *(required)*	Weight measured in First Trimester	
rh_j_11_11 *(required)*	Weight measured in all trimesters	

Field	Question	Answer
rh_j_11_12 *(required)*	Patients Health Questionnaire (PHQ-2) in each trimester	
rh_j_11_13 *(required)*	Tested for Hemoglobin (Hb) 4 time during pregnancy	
RH > rh_j_group > Pregnant women identified with medical conditions & depressive symptoms following screening- *Group relevant when: ${rh_j_3} != 0 and ${rh_j_3} != 999*		
rh_j_12_note	Please mention the Number of Pregnant women identified with medical conditions & depressive symptoms following screening (Last 12 Month Data)	
rh_j_12_1 *(required)*	HIV	
rh_j_12_2 *(required)*	Syphilis	
rh_j_12_3 *(required)*	Hypothyroidism	
rh_j_12_4 *(required)*	Hyperthyroidism	
rh_j_12_5 *(required)*	Hypertension	
rh_j_12_5_1 *(required)*	Hypotension	
rh_j_12_6 *(required)*	Diabetes mellitus	
rh_j_12_7 *(required)*	BMI < 18.5 kg/m2	
rh_j_12_8 *(required)*	BMI < 25 kg/m2	
rh_j_12_9 *(required)*	Pregnant women with inadequate gestational weight gain	
rh_j_12_10 *(required)*	Severe anemia (Hb < 7 g/dL)	
rh_j_12_11 *(required)*	Moderate anemia (Hb 7 to 9.9 g/dL)	
rh_j_12_12 *(required)*	Depressive symptoms	
RH > rh_j_group > Pregnant women managed for- *Group relevant when: ${rh_j_3} != 0 and ${rh_j_3} != 999*		
rh_j_14_note	Please mention the Number of Pregnant women managed for following conditions(Last 12 Month Data)	
rh_j_14_1 *(required)*	HIV	
rh_j_14_2 *(required)*	Syphilis	
rh_j_14_3 *(required)*	Hypothyroidism	
rh_j_14_4 *(required)*	Hyperthyroidism	
rh_j_14_5 *(required)*	Hypertension	
rh_j_14_5_1 *(required)*	Hypotension	
rh_j_14_6 *(required)*	Diabetes mellitus	
rh_j_14_7 *(required)*	Inadequate gestational weight gain (IGWG)	
rh_j_14_8 *(required)*	Severe anemia (Hb < 7 g/dL)	
rh_j_14_9 *(required)*	Moderate anemia (Hb 7 to 9.9 g/dL)	
rh_j_14_10 *(required)*	Depressive symptoms	
rh_j_14_11 *(required)*	Other medical conditions	
rh_j_14_12 *(required)*	No. of pregnant women who were given IFA as per recommendation	
rh_j_14_13 *(required)*	No. of pregnant women who counselled for Nutrition	
rh_j_14_14 *(required)*	No. of pregnant women who counselled for WASH intervention	
RH > rh_j_group > rh_j_1_group *Group relevant when: ${rh_j_3} != 0 and ${rh_j_3} != 999*		
rh_j_15 *(required)*	Pregnant women referred during ANC	
rh_j_16 *(required)*	Pregnant women with atleast 4-ANC check-ups	
rh_j_17 *(required)*	Pregnant women with atleast 8-ANC check-ups	
rh_j_18 *(required)*	Pregnant women given corticosteroids for preterm labor	
rh_j_19 *(required)*	Registered antenatal mothers who came for delivery	
rh_j_20 *(required)*	Institutional deliveries in facility	
rh_j_20_1 *(required)*	Institutional deliveries other facility	
rh_j_21 *(required)*	Home deliveries	
rh_j_22 *(required)*	Maternal deaths	
rh_j_23 *(required)*	Live births	
rh_j_24 *(required)*	Babies born with Very low birth weight (VLBW) < 1,500 grams	
rh_j_25 *(required)*	Babies born with low birth weight (LBW) < 2,500 grams	
rh_j_26 *(required)*	Preterm babies born (<37 weeks)	
rh_j_27 *(required)*	Early neonatal deaths (within 24 hours of birth)	
RH > rh_j_group > Other high-risk pregnancies identified during ANC- *Group relevant when: ${rh_j_3} != 0 and ${rh_j_3} != 999*		
rh_j_13_note	Please mention the Number of Other high-risk pregnancies identified during ANC- (Last 12 Month Data)	
rh_j_13_1 *(required)*	Pregnant women with multiple pregnancy	
rh_j_13_2 *(required)*	Pregnant women with previous bad obstetric history (BOH)*	
rh_j_13_3 *(required)*	Pregnant women with other infections (Hepatitis, Malaria, etc.)	
rh_j_13_4 *(required)*	Pregnant women with other medical conditions (Asthma, COPD, heart disease, etc.)	

Field	Question	Answer
RH > rh_j_group > Postnatal Women (0-6 Months) & 0-24 Months Infants & Child<br>*Group relevant when: ${rh_j_4} != 0 and ${rh_j_4} != 999*		
rh_j_28_note	Please mention the Number of Postnatal Women (0-6 Months) & 0-24 Months Infants & Child (Last 12 Month Data)	
rh_j_28_1 *(required)*	Newborns screened for birth defects (as per RBSK)	
rh_j_28_2 *(required)*	Women received postpartum checkup between 48 hours and 14 days after institutional delivery (Facility/Community)	
rh_j_28_3 *(required)*	HBNC visits performed as per guidelines	
rh_j_28_4 *(required)*	Postnatal mothers consumed nutritional supplements as per recommendation	
rh_j_28_5 *(required)*	Postnatal mothers screened for depressive symptoms	
rh_j_28_6 *(required)*	Postnatal mothers who received appropriate psychosocial support	
rh_j_28_7 *(required)*	Postnatal mothers who counselled for WASH intervention	
rh_j_28_8 *(required)*	Postnatal mothers who were given IFA as per recommendation	
rh_j_28_9 *(required)*	Postnatal mothers who counselled for Nutrition	
rh_j_28_10 *(required)*	0-6 months Infants received kangaroo mother care (KMC) as applicable (Facility/Home)	
rh_j_28_11 *(required)*	0-6 months Infants exclusively breastfed (EBF)	
rh_j_28_12 *(required)*	0-6 months Infants (VLBW and LBW) who consumed Iron, as per recommendation	
rh_j_28_13 *(required)*	0-6 months Infants who consumed Vitamin D 400 IU daily	
rh_j_28_14 *(required)*	0-6 months Infants (Pre-term) who consumed calcium and vitamin D, as per recommendation	
rh_j_28_15 *(required)*	6-24 months infants & children who consumed IFA, as per recommendation	
rh_j_28_16 *(required)*	Infants 6-12 months with inadequate weight gain (<25th centile according to WHO weight velocity/month)	
rh_j_28_17 *(required)*	Children 12-24 months with inadequate weight gain (<25th centile according to WHO weight velocity/month)	
rh_j_28_18 *(required)*	Children (0-24 months) with adequate weight gain from birth till 2 years of age	
rh_j_28_19 *(required)*	Family/Parents having 0-24 months infants & child counselled for WASH interventions	
rh_j_28_20 *(required)*	Children (12-23 months) fully immunized	
rh_j_28_21 *(required)*	Observational Remarks	
rh_j_28_22 *(required)*	Respondent Remarks	
RH > Document		
image1	Document 1	
image2	Document 2	
image3	Document 3	
image4	Document 4	
image5	Document 5	
image6	Document 6	
image7	Document 7	
image8	Document 8	
file_pdf	Please upload PDF Documents	
