# Supplementary material for: Implementation research to scale up the women and infants integrated interventions for growth study (WINGS) in Himachal Pradesh: Protocol for a quasi-experimental, mixed-methods study
Source: PLoS One. 2026 Feb 17;21(2):e0341048. doi: 10.1371/journal.pone.0341048 (PMC12912596; doi:10.1371/journal.pone.0341048)
Supplement: S6 File — (PDF) [file pone.0341048.s007.pdf]

Wings Scale Up

Field	Question	Answer	
household_identification			
a_1	Section-A. Household Identification:<br>District- Una		
worker *(required)*	Worker Name<br>कार्यकर्ता का नाम	101	Sonia Rani
		102	Juhi Sharma
		103	Sushant kumar
		104	Ishita
		105	Jatinder Singh
		106	Parveen Lata
		107	Rekha Devi
		108	Harjeet kaur
		109	Seema Rani
		110	Indu Bala
		111	Manpreet kaur
		112	Akshay 2
		113	Rajan
		114	Upasna
		115	Raman
		116	Bhanu
		117	Kirti
		118	Neha
		119	Romil Singh
		120	Devanshu
		121	Sujata
		122	Rajneesh
		123	Shivam
		124	Akshay 1
		125	Ruksar
		126	Pankaj
		127	Himanshu
		128	Rajat
		129	Ajay
		130	Jyoti
		131	Suruchi
		132	Poorvansh
		133	Monika
		134	Anu Bala
blocks *(required)*	Block Names<br>ब्लॉक नाम	block_1	Amb
		block_2	Basdehra
		block_3	Gagret
		block_4	Haroli
		block_5	Thanakalan
civil_hospital	Civil Hospital	ch_1	CH Amb
		ch_2	CH Chintpurni
		ch_3	CH Cum ESI Gagret
		ch_4	CH Haroli
		ch_5	CH Bangana
		ch_999_1	None
		ch_999_2	None
		ch_999_3	None
		ch_999_4	None
		ch_999_5	None
chc *(required)*	CHC	chc_1	CHC Dussara
		chc_2	CHC Basdehra
		chc_3	CHC Santoshgarh
		chc_4	CHC Daulatpur Chowk
		chc_5	CHC Beeton
		chc_6	CHC Bhadsali

Field	Question	Answer		
			chc_7	CHC Dulehar
			chc_8	CHC Kungrath
			chc_9	CHC Thanakalan
			chc_999_1	None
			chc_999_2	None
			chc_999_3	None
			chc_999_4	None
			chc_999_5	None
phc_selected *(required)*	PHC<br>पीएचसी		phc_1	PHC Akrot
			phc_2	PHC Chaksrai
			phc_3	PHC Chururu
			phc_4	PHC Dharamshala Mahanta
			phc_5	PHC Lohara
			phc_6	PHC Shivpur
			phc_7	PHC Basal
			phc_8	PHC Basoli
			phc_9	PHC Chalola
			phc_10	PHC Dehlan
			phc_11	PHC Amlehar
			phc_12	PHC Badehra Rajputan
			phc_13	PHC Marwari
			phc_14	PHC Badehra
			phc_15	PHC Bathri
			phc_16	PHC Khad
			phc_17	PHC Kuthar Beet
			phc_18	PHC Palkwah
			phc_19	PHC Panjawar
			phc_20	PHC Saloh
			phc_21	PHC Chamiari
			phc_22	PHC Lathiani
			phc_23	PHC Raipur Maidan
			phc_24	PHC Sohari Takoli
			phc_9999_1	None
			phc_9999_2	None
			phc_9999_3	None
			phc_9999_4	None
			phc_9999_5	None
hwc_selected *(required)*	Health wellness centre (HWC)		hwc_1	HWC-HSC KatoharKalan
			hwc_2	HWC-HSC KatoharKhurd
			hwc_3	HWC-HSC Kuthiari
			hwc_4	HWC-HSC Neharian
			hwc_5	HWC-HSC Panjoa
			hwc_6	HWC-HSC Jagannath Mandir
			hwc_7	HWC-HSC Naloh
			hwc_8	HWC-HSC PolianProhitan
			hwc_9	HWC-HSC Ripooh Misran
			hwc_10	HWC-HSC Behra
			hwc_11	HWC-HSC Diara
			hwc_12	HWC-HSC Hamboli
			hwc_13	HWC-HSC Takarala
			hwc_14	HWC-HSC Thathal
			hwc_15	HWC-HSC Badwana

Field	Question	Answer		
			hwc_16	HWC-HSC BaherBatehar
			hwc_17	HWC-HSC Dilwari
			hwc_18	HWC-HSC Ghangret
			hwc_19	HWC-HSC GindpurMaloun
			hwc_20	HWC-HSC Kharoh
			hwc_21	HWC-HSC Thanikpura
			hwc_22	HWC-HSC Chahbag
			hwc_23	HWC-HSC Chowar
			hwc_24	HWC-HSC Daloh
			hwc_25	HWC-HSC Gangoti (sapouri)
			hwc_26	HWC-HSC Lohara
			hwc_27	HWC-HSC Andora
			hwc_28	HWC-HSC Saloi
			hwc_29	HWC-HSC Sidhchalher
			hwc_30	HWC-HSC Suin
			hwc_138	HWC Mubarikpur
			hwc_31	HWC-HSC Basal
			hwc_32	HWC-HSC KotlaKhurd
			hwc_33	HWC-HSC Rainsary
			hwc_34	HWC-HSC Takka
			hwc_35	HWC-HSC Basoli
			hwc_36	HWC-HSC Dangoli
			hwc_37	HWC-HSC Kotla Kalan
			hwc_38	HWC-HSC Lamlehri
			hwc_39	HWC-HSC SamoorKalan
			hwc_40	HWC-HSC Badoli
			hwc_41	HWC-HSC Badsala
			hwc_42	HWC-HSC Ghandwal
			hwc_43	HWC-HSC Kuriala
			hwc_44	HWC-HSC NangalSalangri
			hwc_45	HWC-HSC Panoh
			hwc_46	HWC-HSC Teuri
			hwc_47	HWC-HSC Bedehar
			hwc_48	HWC-HSC Behdala
			hwc_49	HWC-HSC BhadolianKalan
			hwc_50	HWC-HSC Charatgarh
			hwc_51	HWC-HSC Chattara
			hwc_52	HWC-HSC Chattarpur
			hwc_53	HWC-HSC Fatehwal
			hwc_54	HWC-HSC Jalgran
			hwc_55	HWC-HSC Jankaur
			hwc_56	HWC-HSC Jakhera
			hwc_57	HWC-HSC Jhudowal
			hwc_58	HWC-HSC Malahat

Field	Question	Answer		
			hwc_59	HWC-HSC Nangran
			hwc_60	HWC-HSC Rakkar
			hwc_61	HWC-HSC Rampur
			hwc_62	HWC-HSC Sanoli
			hwc_63	HWC-HSC Sassan
			hwc_64	HWC-HSC Gondpur Banera
			hwc_65	HWC-HSC Kuneran
			hwc_66	HWC-HSC Nakroh
			hwc_67	HWC-HSC Ambota
			hwc_68	HWC-HSC Guglehar
			hwc_69	HWC-HSC Keori
			hwc_70	HWC-HSC KutheraJaswalan
			hwc_71	HWC-HSC Loharli
			hwc_72	HWC-HSC MawaSindhian
			hwc_73	HWC-HSC Oel
			hwc_74	HWC-HSC Pambra
			hwc_75	HWC-HSC Saghnai
			hwc_76	HWC-HSC Amboa
			hwc_77	HWC-HSC Babehar
			hwc_78	HWC-HSC Bhaderkali
			hwc_79	HWC-HSC Chalet
			hwc_80	HWC-HSC Dangoh
			hwc_81	HWC-HSC Deoli
			hwc_82	HWC-HSC Ghanari
			hwc_83	HWC-HSC Mandwara
			hwc_84	HWC-HSC MawaKohlan
			hwc_85	HWC-HSC Nangal Jariyala
			hwc_86	HWC-HSC Pirthipur
			hwc_87	HWC-HSC Salohberri
			hwc_88	HWC-HSC Baliwal
			hwc_89	HWC-HSC Dharampur
			hwc_90	HWC-HSC Sainsowal
			hwc_91	HWC-HSC Bathri
			hwc_92	HWC-HSC Bathu
			hwc_93	HWC-HSC Beetan
			hwc_94	HWC-HSC Nangal Kalan
			hwc_95	HWC-HSC Singan
			hwc_96	HWC-HSC Ispur
			hwc_97	HWC-HSC Chhetran
			hwc_98	HWC-HSC Gondpur Bulla
			hwc_99	HWC-HSC Janani
			hwc_100	HWC-HSC Kuthar Beet
			hwc_101	HWC-HSC Polian Beet
			hwc_102	HWC-HSC Pubowal
			hwc_103	HWC-HSC Bhadauri

Field	Question	Answer		
			hwc_104	HWC-HSC Lalehri
			hwc_105	HWC-HSC Nangal Khurd
			hwc_106	HWC-HSC Palakwah
			hwc_107	HWC-HSC Pandoga
			hwc_108	HWC-HSC Nangnoli
			hwc_109	HWC-HSC Panjawar
			hwc_110	HWC-HSC Kangar
			hwc_111	HWC-HSC Chamiari
			hwc_112	HWC-HSC Jarola
			hwc_113	HWC-HSC Piploo
			hwc_114	HWC-HSC Bharmout
			hwc_115	HWC-HSC Charoli
			hwc_116	HWC-HSC Charara
			hwc_117	HWC-HSC Deehar
			hwc_118	HWC-HSC Dhanet
			hwc_119	HWC-HSC Dhundla
			hwc_120	HWC-HSC Jassana
			hwc_121	HWC-HSC Kodra
			hwc_122	HWC-HSC Tanoh
			hwc_123	HWC-HSC Balh
			hwc_124	HWC-HSC Bihroo
			hwc_125	HWC-HSC Boul
			hwc_126	HWC-HSC Budhwar
			hwc_127	HWC-HSC Chugath
			hwc_128	HWC-HSC Chulari
			hwc_129	HWC-HSC Harot
			hwc_130	HWC-HSC Karian
			hwc_131	HWC-HSC Paroin
			hwc_132	HWC-HSC Saili
			hwc_133	HWC-HSC Talai
			hwc_134	HWC-HSC Ambehra
			hwc_135	HWC-HSC Baduhi
			hwc_136	HWC-HSC Bhindla
			hwc_137	HWC-HSC Jol
			hwc_9999_1	None
			hwc_9999_2	None
			hwc_9999_3	None
			hwc_9999_4	None
			hwc_9999_5	None
circle_selected *(required)*	Circle of area		circle_1	Amb
			circle_2	Bhaira
			circle_3	Chaksarai
			circle_4	Chintpurni
			circle_5	Chururu
			circle_6	Jubehar
			circle_7	Kalruhi
			circle_8	Kharoh
			circle_9	Nandpur
			circle_10	Nehrian
			circle_11	Sapouri
			circle_12	Sidhchalehar
			circle_13	Abada Barana
			circle_14	Babarudru

Field	Question	Answer
		circle_15 Bahdala<br>circle_16 Barnoh<br>circle_17 Basal<br>circle_18 Bhatoli<br>circle_19 Dehlan<br>circle_20 Fatehpur<br>circle_21 Ghandawal<br>circle_22 Jhalera<br>circle_23 Lower Arniala<br>circle_24 Raipur<br>circle_25 Rakkad<br>circle_26 Sanoli<br>circle_27 Una<br>circle_28 Ambota<br>circle_29 Badeda Rajputan<br>circle_30 Bhaderkali<br>circle_31 Bhanjal<br>circle_32 Gagret<br>circle_33 Ganu Madwada<br>circle_34 Ghanari<br>circle_35 Mawa Kaholan<br>circle_36 Mawa Sindhia<br>circle_37 Pirtipur<br>circle_38 Bathri<br>circle_39 Dulehar<br>circle_40 Ghaluwal<br>circle_41 Haroli<br>circle_42 Ispur<br>circle_43 Kanger<br>circle_44 Kungrat<br>circle_45 Lalri<br>circle_46 Palakwah<br>circle_47 Panjawar<br>circle_48 Pubowal<br>circle_49 Santoshgarh<br>circle_50 Tahliwal<br>circle_51 Chowki<br>circle_52 Dhundla<br>circle_53 Jasana<br>circle_54 Jol<br>circle_55 Khurwain<br>circle_56 Lathiani<br>circle_57 Piploo<br>circle_58 Raipur<br>circle_59 Thanaklan
awc_center *(required)*	Anganwadi Center<br>आंगनबाड़ी केंद्र	1 Amb-I<br>2 Amb-II<br>3 Amb-III<br>4 Amb-IV<br>5 Amb-V<br>6 Sham Nagar-I<br>7 Sham Nagar-II<br>8 Partap Nagar-I<br>9 Partap Nagar-II<br>10 Hira Nagar-I<br>11 Hira Nagar-II<br>12 Adarash Nagar<br>13 Andoura Upper-I<br>14 Andoura Upper-II<br>15 Andoura Upper-III<br>16 Andoura Upper Middle

Field	Question	Answer

	17	Andora Gujjar Basti
	18	Andora Lower-II
	19	Andora Lower-III
	20	Kuthera Kherla
	21	Kuthera Kherla-IV
	22	Bhaira
	23	Bhaira-II
	24	Bhaira Upper
	25	Bhaira Middle
	26	Bhaira Lower
	27	Dhusara
	28	Dhusara Abble
	29	Dhusara Doam-I
	30	Dhusara Doam-II
	31	Saluri
	32	Diara
	33	Diara-II
	34	Diara-III
	35	Dilwan
	36	Dilwan-II
	37	Chhambah
	38	Satother
	39	Satother-II
	40	Satother Teli Muhalla
	41	Gijjar Cho
	42	Chaksrai
	43	Gathroon
	44	Jhager
	45	Ripoh Misran
	46	Ripoh Muchlian-II
	47	Danguhi
	48	Jhamber
	49	Karap Kotla
	50	Tiai
	51	Baroh
	52	Polian Parohitan
	53	Kuthera Kherla-II
	54	Kuthera Kherla-III
	55	Kherla-I
	56	Kherla-II
	57	Paloh
	58	Bharobadsar
	59	Lander Tikkari
	60	Majhar
	61	Dhar Gujaran
	62	Nari
	63	Nari 2
	64	Nari Harijan Basti
	65	Nari Chobe Basti
	66	Badhmana
	67	Badhmana-II
	68	Badhmana Harijan Basti
	69	Jawal
	70	Jawal-II
	71	Jawal Chang Basti
	72	Chhaproh
	73	Chhaproh 2
	74	Rehi
	75	Duhal Bhatwala
	76	Mirgu (Amokla Pritam)
	77	Dhalwari

Field	Question	Answer

	78	Dhalwari Dehlwan
	79	Chalol Behar
	80	Duhal Bangwala
	81	Paplehra
	82	Chururu-I
	83	Chururu-II
	84	Chururu-III
	85	Chururu-IV
	86	Hamboli
	87	Upper Hamboli
	88	Bandukian Da Behra
	89	Baheri
	90	Singhan Da Behra
	91	Seri-I
	92	Seri-ii
	93	Dhandri-I
	94	Dhandri-II
	95	Shiv Nagar-I
	96	Shiv Nagar-II
	97	Thathal-III
	98	Thathal-IV
	99	Thathal-V
	100	Thathal Ram nagar
	101	Kathiari Ward No. 3
	102	Jubehar
	103	Saroi
	104	Jandoh
	105	Lander Landian
	106	Suri-I
	107	Suri-II
	108	Suri-III
	109	Behar Jaswan
	110	Ladial Chuk
	111	Bagru
	112	Akrot
	113	Chak
	114	Chak Bella
	115	Takarla Lower
	116	Takarla-I
	117	Takarla-II
	118	Takarla-III
	119	Gondpur
	120	Thathal Nakki
	121	Lohara Lower
	122	Bhagra
	123	Tikkari-I
	124	Tikkari-II
	125	Mubarikpur
	126	Ghebat behar
	127	Shivpur
	128	Kashipur-I
	129	Saloi
	130	Pramb
	131	Alehar
	132	Karluhi-I
	133	Karluhi-II
	134	Athwan-I
	135	Athwan-II
	136	Jhangoli
	137	Mandholi
	138	Bringal

Field	Question	Answer		
			139	Mather
			140	Channi Devi
			141	Takoli
			142	Chowar-I
			143	Chowar-2
			144	Kwah
			145	Ghangret-I
			146	Ghangret-II
			147	Ghangret-III
			148	Gindpur
			149	Maloun-I
			150	Maloun-II
			151	Kharoh-I
			152	Kharoh-II
			153	Behar Bhater
			154	Bhater
			155	Behar-I
			156	Behar-II
			157	Baret
			158	Chanourian
			159	Harijan Basti
			160	Dharamshala Mahanta-I
			161	Dharamshala Mahanta-II
			162	Bharar Bar
			163	Badsla Basti
			164	Baba Nakodar Dass
			165	Nandpur-I
			166	Nandpur-II
			167	Gadiale
			168	Muhalla Lambran
			169	Thathal-I
			170	Thathal-II
			171	Kathiari
			172	Kathiari Harijan Basti
			173	Thakur Dwara
			174	Kathiari par Bela
			175	Katohar Kalan-I
			176	Katohar Kalan-II
			177	Talwal
			178	Bijapur
			179	Katohar Khurd-I
			180	Katohar Khurd-II
			181	Pucca Paroh
			182	Andora Lower-IV
			183	Badaun-I
			184	Badaun-II
			185	Bajigar Muhalla
			186	Andora lower
			187	Nehari Nauranga
			188	Nehari Khas
			189	Santo Tilla
			190	Bagga Brota
			191	Karar Behar
			192	Duhki
			193	Januhi
			194	Mairi Khas
			195	Mairi-II
			196	Mairi-III
			197	Gawalsar
			198	Panjoa Khurd
			199	Panjoa kalan

Field	Question	Answer		
			200	Ladoli-I
			201	Thara-I
			202	Thara-II
			203	Thara-III
			204	Kangruhi
			205	Naloh Miyor
			206	Lower Poliyan Purohitan
			207	Spouri
			208	Gangoti
			209	Mughal
			210	Nouhan
			211	Amb Tilla
			212	Band Bakhshi
			213	Kaniari
			214	Ardoh
			215	Rajpur Jaswan
			216	Jaman Kuwali
			217	Maslana
			218	Guliar
			219	Kohar Chhan
			220	Basantpur
			221	Lamba sail
			222	Lahar
			223	Jawar-I
			224	Jawar-II
			225	Jawar Harijan Basti
			226	Patehar
			227	Lohara Upper
			228	Kotli
			229	Guret
			230	Aranwal Chahbag
			231	Thanikpur
			232	Sarda
			233	Aloh
			234	Mawa
			235	Jholan Bhatolan
			236	Chhaprohian
			237	Kashipur-II
			238	Behar
			239	Sidh Chaler
			240	Suhin
			241	Khariali
			242	Jandour-I
			243	Jandour-II
			244	Rampur-I
			245	Rampur-II
			246	Bane Di Hatti
			247	Pinjore-I
			248	Pinjore-II
			249	Abada Barana
			250	Abada Barana-Lohar Basti
			251	Abada Barana-Brahman Khatri Mohalla
			252	Jankaur
			253	Barsada
			254	Barsada-Bahti Jat Mohalla
			255	Jankaur Saini Mohalla
			256	Jankaur Tarkhan Mohalla
			257	Sunehara
			258	Sunehara-Kabir Panthi Moh.

Field	Question	Answer		
			259	Nangran
			260	Nangran-Harijan Moh.
			261	Nangran-Bahati Moh.
			262	Nangran-Bahati Moh.-2
			263	Nangran-Bahati Moh.-3
			264	Nangran-Bahati Moh.-4
			265	Nangran-Bahati Moh.-5
			266	Nangran-Bahati Moh.-6
			267	Badehar
			268	Badehar-Swar Nai-1
			269	Badehar-Swar Nai-2
			270	Badehar-Chilawala
			271	Jhurowal-1
			272	Jhurowal-2
			273	Khui Pekhu Bela
			274	Jhurowal Bahati Moh.
			275	Jhurowal Bahati Moh.-2
			276	Nangal Slangri-1
			277	Lehad
			278	Parla Sanjhot
			279	Nangal Salangri-2
			280	Sanjhot
			281	Nari
			282	Nari- Lower -1
			283	Nari- 2
			284	Dhadhial-2
			285	Dhadhial-
			286	Nari-3
			287	Chalola
			288	Chalola-2
			289	Chalola-3
			290	Dhamandri-1
			291	Dhamandri-2
			292	Dhamandri-Satleta
			293	Dhamandri-Mansoh
			294	Dhamandri-3
			295	Dhamandri-4
			296	Dathwada
			297	Dathwara-2
			298	Barera
			299	Barera-2
			300	Behdala-1
			301	Behdala-2
			302	Vasdev Khidri Mohalla
			303	Behdala-Harijan Mohalla-1
			304	Behdala-Harijan Mohalla-2
			305	Behdala-Khidri Mohalla
			306	Vasdev Harijan Mohalla
			307	Behdala-Rajput Mohalla-1
			308	Behdala-Rajput Mohalla-2
			309	Behdala-Rajput Mohalla-3
			310	Behdala-Valmiki Mohalla-1
			311	Behdala-Valmiki Mohalla-2
			312	Chatara
			313	Chatara Mahadev
			314	Chatara Brahaman Mohalla
			315	Chatara Harijan Mohalla
			316	Chatara Lohar Mohalla
			317	Chatara Khatri Mohalla
			318	Chatara Brahaman Mohalla-2

Field	Question	Answer		
			319	Chatara Labana Mohalla
			320	Bharolian Kalan
			321	Bharolian Kalan Jhingla Behda
			322	Bharolian Kalan Bade Wala Mohalla
			323	Bharolian Kalan Harijan Mohalla
			324	Barnoh
			325	Barnoh Jat Saini Mohalla
			326	Dangera-1
			327	Dangera-2
			328	Dangoli
			329	Dangoli Tarkhan Mohalla
			330	Dangoli Harijan Mohalla
			331	Dangoli Brahaman Mohalla
			332	Dangoli Jatt Mohalla
			333	Samoor
			334	Bhaur
			335	Samoor Bhaur Brahaman Mohalla
			336	Samoor Bhaur Saur Mohalla
			337	Samoor Bhaur Chhalwad Mohalla
			338	Kuriala-1
			339	Kuriala Bhaur
			340	Kuriala Haled
			341	Kuriala-2
			342	Jhambar-1
			343	Jhambar Chilliyan Harijan
			344	Sarjehra
			345	Laam
			346	Jhambar Lower
			347	Basal Upper
			348	Basal Harijan Basti
			349	Basal Brahaman Basti
			350	Basal Khwaja Basti-1
			351	Basal Bada Behda
			352	Basal Bugde Bablu
			353	Basal Khwaja Basti-2
			354	Lower Basal
			355	Lower Basal Harijan Basti
			356	Lower Basal Dhiman Bahati Mohalla
			357	Lower Basalbahati Mohalla-2
			358	Lower Basalbahati Mohalla-3
			359	Lower Basalbahati Mohalla-4
			360	Takka Bishna
			361	Takka Ramsahay
			362	Takka Bishna Saini Mohalla
			363	Takka Bahati Mohalla
			364	Takka Harijan Mohalla
			365	Takka Ramsahay Saini Moh.
			366	Takka Ramsahay Brahaman Harijan Moh.
			367	Kotla Khurd
			368	Kotla Khurd Khatri Mohalla

Field	Question	Answer		
			369	Kotla Khurd Lohar Mohalla
			370	Bhatoli -1
			371	Bhatoli -2
			372	Bhatoli Khrtikre Mohlla
			373	Bhatoli Chirbe Julahe Mohlla
			374	Morbar-1
			375	Morbar-2
			376	Bhatoli Jatt Moh.-1
			377	Bhatoli Jatt Moh.-2
			378	Bhatoli Harijan Basti-1
			379	Bhatoli Harijan Basti-2
			380	Jakhera -1
			381	Jakhera -2
			382	Jakhera Hari Basti
			383	Jakhera Basdev Moh.
			384	Jakhera Brahman Moh.-1
			385	Jakhera Brahman Moh.-2
			386	Jakhera Balmiki Moh.
			387	Bangrah Purana Kander
			388	Fatewal
			389	Bangrah -1
			390	Bangrah -2
			391	Bangrah Pukhru Moh.
			392	Dehlan-Upper-1
			393	Dehlan-Upper-2
			394	Uppar Dehlan Mahldarji-1
			395	Uppar Dehlan Mahldarji-2
			396	Uppar Dehlan Negi Budu Mohlla
			397	Uppar Dehlan Harjin Basti
			398	Uppar Dehlan Grewal Mohlla
			399	Uppar Dehlan Kavir Panthi Nai Mohlla
			400	Upper Dehlan Bans Bansre Moh.
			401	Uppar Dehlan Bade Wale Mohalla
			402	Lower Dehla-1
			403	Lower Dehla-2
			404	Lower Dehla-3
			405	Lower Dehlan Tarkhan Moh.
			406	Lower Dehlan Bahti Moh.
			407	Lower Dehlan Harijan Basti
			408	Lower Dehlan Brahman Moh.
			409	Lower Dehlan Bahti Moh.-1
			410	Lower Dehlan Bahti Moh.-11
			411	Lower Dehlan Bahti Moh.-111
			412	Lower Dehlan Bats Tikre
			413	Mehtpur
			414	Mehatpur Dwedi Mohalla
			415	Shri Lanka Mohlla Mehtpur
			416	Fateh Pur-1
			417	Fateh Pur Harijan Basti-1
			418	Fateh Pur Harijan Basti-2
			419	Fateh Pur Bahati Mohalla
			420	Fateh Pur -2

Field	Question	Answer		
			421	Khanpur-1
			422	Khanpur Bahati Saini Mohalla
			423	Khanpur Bahati Mohalla
			424	Khanpur Harijan Basti
			425	Khanpur -2
			426	Uday Pur
			427	Uday Pur Dhiman/ Rajput Mohalla
			428	Sasan
			429	Sasan Harijan Basti
			430	Sasan Bahati Mohalla
			431	Sasan Jatt Mohalla
			432	Charat Garh-1
			433	Charat Garh-2
			434	Charat Garh-Lahar Mohalla
			435	Charat Garh-Aeri Mohalla
			436	Charat Garh-Harijan Basti
			437	Charat Garh-Bajit Pur
			438	Kuthar Kalan
			439	Kuthar Kalan Bahati Mohalla
			440	Kuthar Kalan Tarkhan/Rajput Mohalla
			441	Tyuri-1
			442	Tyuri-2
			443	Tyuri-3
			444	Panoh-1
			445	Panoh-2
			446	Panoh-3
			447	Bhalola
			448	Baduhi
			449	Baduhi-1
			450	Bhaloh
			451	Ghandawal-1
			452	Ghandawal-2
			453	Badoli-1
			454	Badoli Hari Basti
			455	Badoli-2
			456	Badsala
			457	Badsala Khambuya Da Mohlla
			458	Badsala Bankeya Da Mohlla
			459	Jhalera Upper
			460	Jhalera Lower
			461	Jhalera Banga Bala Mohlla
			462	Jhlera Partap Mohlla
			463	Jhlera New Beli Kloni
			464	Rampur-1
			465	Rampur-2
			466	Rampur Jatt Mohlla
			467	Rampur Harijan Mohlla
			468	Rampur Bahti Brahman Mohlla
			469	Kuthar Harijan Ghabre Mohlla
			470	Kuthar Khurd Brahman Bati Mohlla
			471	Kuthar Khurd
			472	Lal Singi

Field	Question	Answer		
			473	Lal Singi Rajput Brahman Mohlla
			474	Lal Singi Rajputsaini Mohlla
			475	Rainsari Mdhey
			476	Rainsari Purb
			477	Rainsari Brahman Mohlla
			478	Rainsari Harijan Moh.
			479	Rainsari Bahti Moh.-1
			480	Rainsari Brahman Jatt Moh.
			481	Rainsari Bahti Mohlla-2
			482	Lower Arniala
			483	Lower Arniala Sharma Saini Mohalla
			484	Lower Arniala Bahati Saini Mohalla
			485	Upper Arniala
			486	Upper Arniala Saini Harijan Mohalla
			487	Upper Arniala Adarsh Nagar
			488	Upper Arniala Rajput Lohar Mohalla
			489	Upper Arniala Jhangri Mohalla
			490	Upper Arniala Tarkhan Rajput Mohalla
			491	Lower Kotla Kalan
			492	Lower Kotla Kalan Saini Mohalla
			493	Lower Kotla Kalan Brahaman Mohalla
			494	Lower Kotla Kalan Tarkhan Mohalla
			495	Upper Kotla Kalan
			496	Upper Kotla Kalan Brahaman Mohalla
			497	Upper Kotla Kalan Lohar Mohalla
			498	Upper Kotla Kalan Tarkhan Mohalla
			499	Upper Kotla Kalan Harijan Mohalla
			500	Ajnoli
			501	Ajnoli Saini Mohalla
			502	Ajnoli Brahman Sood Mohalla
			503	Ajnoli Upper
			504	Lamlahri
			505	Lamlahri Brahman Mohalla
			506	Lamlahri Upper
			507	Lamlahri Badla Mohalla
			508	Raypur-1
			509	Raypur-2
			510	Raypur-3
			511	Raypur-4
			512	Raypur Buje Bahti Mohlla
			513	Raypur Hatti Bale Mohlla
			514	Raypur Tarkhan Mohlla
			515	Raypur Gabla Mohlla
			516	Raypur Mehar Mohlla
			517	Raypur Braman Mohlla
			518	Raypur Jatt Behra

Field	Question	Answer

	519	Raypur Bhatha Lekhraj
	520	Basdera Kendr No.13
	521	Basdera Kendr No.14
	522	Basdera Kendr No.15
	523	Basdera Kendr No.16
	524	Basdera Kendr No.17
	525	Basdera Kendr No.18
	526	Basdera Bard No.3+5
	527	Basdera Bard No.6
	528	Basdera Bard No.9+8
	529	Basdera Bard No.1
	530	Lamlehra Purana
	531	Lamlehra Brahman Mohlla
	532	Lamlehra -2
	533	Madan Pur
	534	Madan Pur-Saini Mohalla
	535	Madan Pur-2
	536	Basoli-1
	537	Basoli-2
	538	Basoli-Dhesi Jat Mohalla
	539	Basoli-Bhat Jat Mohalla
	540	Basoli-Brahaman Mohalla
	541	Basoli-Dhiman Mohalla
	542	Basoli-Nala Mohalla
	543	Malahat
	544	Bharolian Khurd
	545	Parli Patti Malahat
	546	Brahaman Patti Malahat
	547	Harijan Patti Malahat
	548	Rajput Patti Malahat
	549	Bharolian Khurd Brahanana-1
	550	Bharolian Khurd Brahanana-2
	551	Tabba-2
	552	Rakkar
	553	Tabba Rajput Mohalla-2
	554	Tabba Rajput Mohalla-3
	555	Tabba Harijan Mohalla
	556	Tabba Lohar Mohalla
	557	Tabba -1
	558	Sanoli-1
	559	Sanoli-2
	560	Sanoli-Rajput Jat Mohalla-1
	561	Sanoli-Rajput Jat Mohalla-2
	562	Sanoli-Rajput Mohalla
	563	Sanoli-Harijan Mohalla-1
	564	Sanoli-Harijan Mohalla-2
	565	Sanoli-Brahaman Mohalla
	566	Majara
	567	Majara Jat Mohalla-1
	568	Majara Jat Mohalla-2
	569	Majara Jat Mohalla-3
	570	Malukpur
	571	Malukpur Jat Mohalla
	572	Binewal
	573	Puhna-1
	574	Puhna-2
	575	Puhna-3
	576	Ajoli
	577	Ajauli Brahman Mohalla-1

Field	Question	Answer

	578	Ajauli Brahman Mohalla-2
	579	Ajauli Harijan Basti
	580	Ajauli Bahati Jat Mohalla-1
	581	Ajauli Bahati Jat Mohalla-2
	582	Prem Nagar
	583	Gursar Mohalla
	584	Vikas Nagar
	585	Vivek Nagar
	586	Pulwala Bazar
	587	Purana Dakkhana
	588	Shiv Nagar
	589	Nagraj Mohalla
	590	Dc Colony
	591	Behli Mohalla-
	592	Neela Ghat
	593	Sabji Mandi W.No-1
	594	W.No-1 Centre-2
	595	Galua-1
	596	Galua-2
	597	Ward (2+7)
	598	Ward (4+8)
	599	Behli Mohalla-2
	600	Ward 7&11 Centre-2
	601	Chanderlok Colony
	602	Neelaghat Colony
	603	Friends Colony
	604	Lower Chatehar
	605	Upper Chatehar
	606	Polytechnical Ambota
	607	Dawali Ambota
	608	Jogdehi
	609	Jhalowali
	610	Handiyala
	611	Parla Behra
	612	Mahila Mandal
	613	Kharasi Mohalla
	614	Lambardara Mohalla
	615	Panchyat Ghar
	616	Jindwad Mahila Mandal
	617	Ambota Gumma
	618	Nagnath Ambota
	619	Chatehar Sant Mohalla
	620	Matyalika Saghnai
	621	Gujjar Khad
	622	Nangal Panga
	623	Panchyat Ghar
	624	Torewala Saghnai
	625	Chang Basti
	626	Mahila Mandal
	627	Kala Panga
	628	Guglehar
	629	Upper Guglehar
	630	Mahila Mandal
	631	Thakur Dwara
	632	Lath Muhalla
	633	Upper Sarai
	634	Teli Mohalla
	635	Harizan Basti
	636	Jat Sarai
	637	Obc Mohalla
	638	Sarai Jadla Keori

Field	Question	Answer		
			639	Jaswal Mohalla
			640	Bazar-11
			641	Dehra Mohalla
			642	Swan Mohalla
			643	Mahila Mandal
			644	Shiv Dwala Upper Nagar Wala
			645	Pathani Mohalla
			646	Dharamshala Mohalla
			647	Kolar Mohalla
			648	Kuan Bala Mohalla
			649	Ara Mohalla
			650	Sadak Mohalla
			651	Piplu-1
			652	Bandu-11
			653	Ambi-111
			654	Mahila Mandal
			655	Bhat Basti
			656	Fatehpur
			657	Brahampur-1
			658	Brahampur-11
			659	Lower Baneda-1
			660	Lower Baneda -11
			661	Dharma-111
			662	Harwal
			663	Gondpur Baneda Upper -1
			664	Baneda -11
			665	Baneda -111
			666	Upper Baneda Roda-4
			667	Kuneran-1
			668	Sanoli
			669	Kuneran-4
			670	Kuneran-11
			671	Harizan Basti
			672	Bazigar Basti
			673	Bagan Mohalla
			674	Julaha Basti
			675	Bilu Di Talai
			676	Kailash Nagar -1
			677	Kailashnagar-11 Tundkhuri
			678	Hariyala Kuteda
			679	Kamali Ram Nagar-1
			680	Ram Nagar -2 Haled
			681	Amlehar-1 Rampur Kuteda
			682	Amlehar-11 Harizan Basti
			683	Amlehar-111 Sunkali
			684	Amlehar Khas
			685	Gokal Nagar
			686	Upper Bhanjal Shankar Nagar
			687	Bhanjal Upper Guga Basti
			688	Bhanjal Upper Harizan Basti
			689	Lower Bhanjal -1 Sarai
			690	Lower Bhanjal -11 Bhatwal
			691	Bada Talab
			692	Lower Bhanjal -4
			693	Lower Bhanjal-5 Kadd
			694	Jit Pur Behari-1
			695	Jitpur Behri-11
			696	Thaplan

Field	Question	Answer		
			697	Dakha Bala Kuan
			698	Braham Sarai
			699	Lower Bhathia Wala
			700	Panwada
			701	Nai Mohalla
			702	Badoh Depot
			703	Upper Bhathia Bala
			704	Shiv Mandir Kaloh
			705	Harizan Basti
			706	Bumbaloo
			707	Kaloh Behli
			708	Chang Basti
			709	Kaloh Beli
			710	Harizan Basti
			711	Beli Gagret
			712	Brahmin Basti Gagret
			713	Panchyat Ghar
			714	Gujjar Khad
			715	Dev Nagar
			716	Purana Amb Road Gagret
			717	Bharwai Road Gagret
			718	Hanuman Mandir
			719	Purana Amb Road Chakki
			720	Pakka Paroh
			721	Shiv Mandir
			722	Raipur Mohalla
			723	Rania Talab
			724	Miidle Depot
			725	Harizan Basti
			726	Kumhar Basti
			727	Bukhaipuir
			728	Brahaman Basti
			729	Chang Pukhar
			730	Obc Mohalla
			731	Marwadi Behal
			732	Marwadi Lower
			733	Lower Madwada
			734	Middle Madwada
			735	Upper Madwada
			736	Tilla Takka
			737	Salohberi Samadi
			738	Lohar Muhalla
			739	Harizan Basti
			740	Salohberi Kothi
			741	Kurialai
			742	Joh Khas
			743	Panchyat Ghar
			744	Tillu Chaunta
			745	Joh Beh
			746	Joh Beh Khad
			747	Deoli Wadi
			748	Cylinder Factory
			749	Shiv Mandir Deoli
			750	Harizan Basti Deoli
			751	High School Deoli
			752	Deoli Chada Basti
			753	Tubewell Deoli
			754	Ghanari Moni Baba
			755	Ayrvedic Dispensary Ghanari
			756	Harizan Basti Ghanari

Field	Question	Answer

	757	Upper Ghanari
	758	Ghanri Chang Basti
	759	Upper Chang Basti
	760	Nangal Jarialan Talab
	761	Nangal Jarialan Middle School
	762	Nangal Jarialan Harizan Basti
	763	Nangal Jarialan Moru Panga
	764	Nangal Jariaan Theda
	765	Nangal Jarialan Kandi Bhavan
	766	Nangaj Jarialan Andwad
	767	Nangal Jariaalan Shivali
	768	Nangal Jarialan Jhalera
	769	Amboa Sub Centre
	770	Amboa Upper
	771	Amboa Primary School
	772	Amboa Harizan Basti
	773	Swan Par Harwall
	774	Bus Stand Mawa Kaholan
	775	Health Centre
	776	Mawa Kaholan Middle
	777	Tarali
	778	Bedha Mohalla
	779	Harizan Basti
	780	Shiv Badi
	781	Badhiakha
	782	Mahila Mandal
	783	Bagan Bala Kuan Chalet
	784	Simli Bala Kuan
	785	Pukhari Pur
	786	Mande Chalet
	787	Harizan Basti
	788	Girl School
	789	Tapriala
	790	Daulatpur Chowk
	791	Dav Daulatpur Chowk
	792	Dholwaha Road
	793	Kua Devi
	794	Tuta Bala Kuan
	795	Bai Bhatha
	796	Babehad Mahila Mandal
	797	Middle School
	798	Lawana Mohalla
	799	Panchyat Ghar
	800	Maralu Mohalla
	801	Khad Gujaran
	802	Lambar Dar Mohalla
	803	Obc Mohalla
	804	Upper Gram
	805	Sc Basti
	806	Upper Society
	807	Seth Coloney
	808	Upper Khad Gujaran
	809	Bus Stand
	810	Upper Sarai Tateda
	811	Panchyat Ghar
	812	Factory Coloney
	813	Sc Basti

Field	Question	Answer

	814	Obc Mohalla
	815	Nepali Coloney
	816	Mahila Mandal
	817	Oel Ashram
	818	Upper Sarai
	819	Harizan Basti
	820	Haroti Mohalla
	821	Pirthipur Faquti
	822	Mahila Mandal
	823	Maidangarh
	824	Harizan Basti
	825	Lohar Basti
	826	Bank Ghar
	827	Pirthipur Chaua
	828	Pirthipur Khas
	829	Dangoh Khurd
	830	Upper Pirthipur
	831	Gujjar Khad Dangohkhas
	832	Setha Mohalla
	833	Mahila Mandal
	834	Dangoh Pirthipur Road
	835	Harizan Basti
	836	Gumma Dangohkhas
	837	Mohalla Kavirpanthi
	838	Dodua Dangoh
	839	Abhaypur Chang Basti
	840	Abhaypur Upper
	841	Abhaypur Middle
	842	Bathri (Present)
	843	Bathri I (Present)
	844	Lohar Muhalla
	845	Bahti Muhalla
	846	Rajput Muhalla
	847	Changare Muhalla
	848	Brahmin Muhalla
	849	Beetan (Present)
	850	Jakhewal (Present)
	851	Jakhewal I
	852	Gujjar Basti I
	853	Gujjar Basti Iii
	854	Gujjar Basti Ii
	855	Harijan Muhalla
	856	Lohar Muhalla
	857	Singan Present
	858	Singan I
	859	Havelli Vala
	860	Brahmin Muhalla
	861	Rajput Muhalla
	862	Harijan Basti
	863	Kotli Vala
	864	Pangyan Vala
	865	Heera Nagar
	866	Sahoowal 1
	867	Sahowal Ii
	868	Rajputan Polician
	869	Brahman Muhalla
	870	Lohar Muhalla
	871	Dulehar
	872	Gaua
	873	Muslim Muhalla
	874	Mishra Muhalla

Field	Question	Answer		
			875	Beebar Muhalla
			876	Bhagtan Muhalla
			877	Harijan Muhalla
			878	Brahman Muhalla
			879	Gondpur Jai Chand 1
			880	Gondpur Jai Chand Ii
			881	Gondpurjaichand Iii
			882	Kabir Panthi Muhalla
			883	Beldar Basati
			884	Brahman Muhalla
			885	Rajput Tarkhan Muhalla
			886	Gondpur Bullan 1
			887	Gandpur Bullan Ii
			888	Brahman Muhalla I
			889	Brahman Muhalla Ii
			890	Gongpur
			891	Pathak Muhalla
			892	Bank Ghar
			893	Harijan Basti
			894	Saini Basti
			895	Panchayat Ghar
			896	Bhadsali Harijan Basti
			897	Bagru Muhalla
			898	Bhuliyan Muhalla
			899	Manguwal Muhalla
			900	Shah Muhalla
			901	Bhadsali Varatmaan
			902	Bhadsali Haar
			903	Kalla Muhalla
			904	Bada Haar
			905	Chota Haar
			906	Jejon Moad
			907	Saloh Bhatta
			908	Jejon Moad Ii
			909	Ghaluwal Bazar
			910	Kyar Muhalla
			911	Saloh Haar
			912	Harijan Basti
			913	Saloh Mahadev I
			914	Saloh Mahadev Ii
			915	Shiv Mandir Saloh
			916	Dharampur Lower
			917	Gurduara Basti
			918	Sidh Channo Mandir
			919	Bada Beda
			920	Shiv Mandir
			921	Sansowal (Present)
			922	Samnal (Present)
			923	Kuruwala Samnal
			924	Bati Muhalla
			925	Harijan Basti
			926	Rahar Muhalla
			927	Main Bazar Samnal
			928	Rora (Present)
			929	Nichla Mazra
			930	Chande Muhalla
			931	Palaki Vala
			932	Haroli I(Present)
			933	Haroli Ii
			934	Roleyan Muhalla
			935	Harijan Basti

Field	Question	Answer		
			936	Lohar Basti
			937	Bati Muhalla Dhol
			938	Haar Khera
			939	Kali Badi
			940	Saini Bati Muhalla
			941	Haijan Basti Khad I
			942	Harijan Basti Ii
			943	Jangle Panehra
			944	Pandoga Barrier
			945	Upper Pandoga
			946	Pandoga Lower
			947	Pandoga Kharoian Muhalla
			948	Pandoga Barrier Ii
			949	Chakk Muhalla Pandoga
			950	Tahlia Muhalla
			951	Atava Muhalla
			952	Pandoga Pathak Muhalla
			953	Saini Muhalla
			954	Jole Harijan Basti
			955	Pandoga Kyarian Muhalla
			956	Bag Bharwal Muhalla
			957	Brahman Basti
			958	Ispur Hoshiarpur Road
			959	Lawana Majra I
			960	Ispur 'Gagret Road
			961	Labana Majra -Ii
			962	Pathak Muhalla
			963	Ravidas Mandir
			964	Saruain Bag
			965	Tippar Muhalla
			966	Saloh Harijan Basti I
			967	Purian Muhalla
			968	Sr Sec. School Saloh
			969	Bholian Muhalla
			970	Jaat Muhalla
			971	Upper Badehra Phc
			972	Middle School Badehra
			973	Upper Harijan Basti
			974	Upper Lohar Muhalla
			975	Katwal Muhalla
			976	Thakaran Muhalla
			977	Lavana Muhalla
			978	Gujjar Pahari Muhalla
			979	Lower Badera
			980	Shiv Mandir Badhera
			981	Jatt Muhalla
			982	Bhai Da Moad
			983	Jaswal Muhalla
			984	Upper Kanger
			985	Lower Kanger
			986	Bakralu Muhalla
			987	Kanger Haar
			988	Bharwal Muhalla
			989	Harijan Basti
			990	Dharampur (Present Upper)
			991	Harijan Basti
			992	Brahmin Satta Muhalla
			993	Malluwal Purana
			994	Polia Beet I
			995	Janni I
			996	Majra Jhole

Field	Question	Answer

	997	Polian Beet Ii
	998	Polian Iii
	999	Janni Ii
	1000	Chhetran I
	1001	Biderwal
	1002	Harijan Dita
	1003	Tarkhan Muhalla
	1004	Khatri Muhalla
	1005	Chhetran Ii
	1006	Kungrat Vartman
	1007	Rana Muhalla
	1008	Ambi Muhalla
	1009	Chowki Muhalla
	1010	Bhandiara Ii
	1011	Brahmin Kash Muhalla
	1012	Bhandiara I
	1013	Lalri I(Present)
	1014	Lalri Ii (Present)
	1015	Lalri Iii (Present)
	1016	Lalri Iv (Present)
	1017	Kelian Muhalla
	1018	Brahmin Muhalla
	1019	Dhanaru Muhalla
	1020	Bati Muhalla
	1021	Jhave Muhalla
	1022	Bakralu Muhalla
	1023	Tarkhan Muhalla
	1024	Bharwal Muhalla
	1025	Brahmin, Bati Muhalla
	1026	Bati, Saini Muhalla
	1027	Lohar Bati Muhalla
	1028	Kalehra (Present)
	1029	Saini Chuhowal Muhalla
	1030	Kalehra Ii
	1031	Khatta Muhalla
	1032	Heera I (Present)
	1033	Heera Ii (Present)
	1034	Loothre Muhalla
	1035	Dhugge Muhalla
	1036	Subboana
	1037	Bhadhori (Present)
	1038	Lohar Muhalla
	1039	Brahmin Harijan Muhalla
	1040	Bati Muhalla
	1041	Nichli Jatt Basti
	1042	Upper Palakwah
	1043	Kante Varatman
	1044	Jatt Muhalla
	1045	Brahmin Muhalla
	1046	Pandit Tarkhan, Naee, Lavana Muhalla
	1047	Harijan Muhalla
	1048	Tarkhan Muhalla
	1049	Kumhaar Muhalla
	1050	Bati Muhalla
	1051	Gill Behra Thakra
	1052	Thakaran Muhalla
	1053	Gangtho (Present)
	1054	Sutre Saini Muhalla
	1055	Pulvari Muhalla
	1056	Karampur

Field	Question	Answer

	1057	Tholle Muhalla
	1058	Kharwal Muhalla
	1059	Karampur Lower
	1060	Nangnoli (Present)
	1061	Nangnoli (Haar)
	1062	Lavana Majra
	1063	Harijan Basti
	1064	Panjawar I (Present)
	1065	Panjawar Ii
	1066	Jole I
	1067	Panjawar Iii
	1068	Jangle Muhalla
	1069	Khatri Basti
	1070	Jole Ii
	1071	Tarkhana Mohalla
	1072	Thhakki I
	1073	Daulatpur Haar
	1074	Thhakki Ii
	1075	Upper Daulatpur
	1076	Sain Basti
	1077	Main Baazar Panjawar
	1078	Master Muhalla
	1079	Khad Present
	1080	Bhaini Muhalla
	1081	Hoshiarpur Road Khad
	1082	Swanpar Malagarh
	1083	Khad Centre
	1084	Dangewala Muhalla
	1085	Pubowal (Present)
	1086	Pubowal (Center)
	1087	Harijan Basti I
	1088	Gurudwara Basti
	1089	Harijan Basti Ii
	1090	Padyan Muhalla
	1091	Ramsar Muhalla
	1092	Baliwal
	1093	Jatt Muhalla
	1094	Brahmin Muhalla
	1095	Harijan Muhalla
	1096	Rajput Muhalla
	1097	Panjuana I
	1098	Panjuana Ii
	1099	Pubowal Ii
	1100	Badewala
	1101	Gurudwara Muhalla Bilna
	1102	Halera
	1103	Kuthar I
	1104	Makkorgarh
	1105	Aperlipali
	1106	Mishra Brahmin Muhala
	1107	Kutharbeet Ii
	1108	Kumhar Kabir Panthi Muhalla
	1109	Ward No.I Santoshgarh
	1110	Ward No.Ii Santoshgarh
	1111	Ward No.Iii Santoshgarh
	1112	Ward No.Iv Santoshgarh
	1113	Ward No.V Santoshgarh
	1114	Ward No.Vi Santoshgarh
	1115	Ward No.Vii Santoshgarh
	1116	Ward No.Ix Santoshgarh

Field	Question	Answer

	1117	Ward No Viii Santoshgarh
	1118	Ward No I+Ii Santoshgarh
	1119	Ward No Vii, Viii, Ix
	1120	Chhaterpur(Present)
	1121	Bahti Muhalla
	1122	Dada
	1123	Bathu (Present)
	1124	Gurplah (Present)
	1125	Gurplah Ii
	1126	Harijan Basti I
	1127	Harijan Basti Ii
	1128	Kelluan Muhalla
	1129	Lamber Luvana Muhalla
	1130	Upperla Muhalla
	1131	Morvadi I
	1132	Morvadi Ii
	1133	Bathu Khas
	1134	Nangal Kalan (Present)
	1135	Jattpura (Present)
	1136	Harijan Basti I
	1137	Bharare Mohalla
	1138	Brahmin Mohalla
	1139	Jattpura Ii
	1140	Nangal Kalan Ii
	1141	Rajput Muhalla (Nangal Kalan)
	1142	Tahliwal
	1143	Nangal Khurd(Present)
	1144	Manuwal
	1145	Upper Manuwal
	1146	Tibba Muhalla
	1147	Bahti Muhalla I
	1148	Bahti Muhalla Ii
	1149	Upper Rajput Muhalla
	1150	Lower Rajput Muhalla
	1151	Kiduan Muhalla
	1152	Batkalan (Present)
	1153	Upper Basti I
	1154	Upper Basti Ii
	1155	Nichli Basti
	1156	Saini Basti
	1157	Chowki -1
	1158	Chowki -2
	1159	Chowki-4
	1160	Chowki -5
	1161	Beerian-1
	1162	Beerian -2
	1163	Beerian -3
	1164	Ladila
	1165	Bhaloun
	1166	Bharmar
	1167	Baduha -1
	1168	Baduha -2
	1169	Baldoh
	1170	Kuder
	1171	Sohari
	1172	Chauli
	1173	Baderah
	1174	Basapatti
	1175	Bhindla
	1176	Baduhi-I

Field	Question	Answer		
			1177	Baduhi-Ii
			1178	Baduhi-Iii
			1179	Pallian
			1180	Panjoda
			1181	Chowki-Iii
			1182	Kachhyari
			1183	Amrera
			1184	Sasoli
			1185	Malanger
			1186	Naroonh
			1187	Nanawin-I
			1188	Nanawin-Ii
			1189	Dhundla-1
			1190	Dhundla-Ii
			1191	Dhundla-Iii
			1192	Dhatol-I
			1193	Dhatol-Ii
			1194	Kusan Ranauta
			1195	Beri Hatli
			1196	Beri-Ii
			1197	Kotla
			1198	Dohgi
			1199	Upper Dohgi
			1200	Bhugdiyan
			1201	Baut
			1202	Bangana
			1203	Upper Nayali
			1204	Bhaleti
			1205	Muchhali
			1206	Chilli
			1207	Jakhola
			1208	Jandoor
			1209	Hatli
			1210	Rivar
			1211	Danoh
			1212	Hathloun
			1213	Aisan
			1214	Samlara
			1215	Lakhroon
			1216	Majhiani
			1217	Arloo
			1218	Arloo Gurmukh
			1219	Karor
			1220	Arloo Khas
			1221	Bharmot
			1222	Karmali
			1223	Nahri
			1224	Baggi
			1225	Dagru
			1226	Khadol
			1227	Sai
			1228	Charara
			1229	Badoa
			1230	Raonkhar
			1231	Vahi
			1232	Talmera
			1233	Deehar-1
			1234	Deehar-2
			1235	Rajpura
			1236	Chadoli
			1237	Buhana

Field	Question	Answer		
			1238	Chaplah
			1239	Thathoon
			1240	Talmet
			1241	Nalwari
			1242	Dumkhar
			1243	Takoli-1
			1244	Takoli-2
			1245	Behla
			1246	Jol
			1247	Baslehar
			1248	Harsa Jandora
			1249	Bagnal
			1250	Amroh
			1251	Dhroon
			1252	Ghaneti Mini Awc
			1253	Khurwin
			1254	Samoor Khurd
			1255	Boul
			1256	Boul Har
			1257	Jogi Panga
			1258	Moh Khass
			1259	Tyar-1
			1260	Tyar-2
			1261	Kubadi
			1262	Ambehera Ramkishan
			1263	Ambeheradeeraj
			1264	Kukhera
			1265	Harot
			1266	Chakdoa
			1267	Kud
			1268	Gehra Kothi
			1269	Gughan Kalan
			1270	Kakrna
			1271	Kyara
			1272	Jagatkhana
			1273	Handola-1
			1274	Handola-2
			1275	Kamoon
			1276	Sanhal
			1277	Tanda
			1278	Tanoh
			1279	Kaihwin
			1280	Karsai
			1281	Dughar
			1282	Tureta
			1283	Dadiyar
			1284	Lathiani
			1285	U.Rajli
			1286	Rajli Baniyala
			1287	Tyasar
			1288	Bilgran
			1289	Aliyana
			1290	Naloot
			1291	Budhan-1
			1292	Budhan-2
			1293	Turkal
			1294	Kughal
			1295	Neri
			1296	Padyola
			1297	Kohdra
			1298	Dhret Dam

Field	Question	Answer

	1299	Daihan
	1300	Rachhoh
	1301	Kheri
	1302	Saroh
	1303	Chamyari
	1304	Basaatar
	1305	Kot
	1306	Jandana
	1307	Dolu
	1308	Jarola
	1309	Hatwana
	1310	Piploo
	1311	Nichla Thana
	1312	Ghaloon
	1313	Alsaan
	1314	Bhyambhi
	1315	Chataihar
	1316	Tehi
	1317	Hatli Patiyalan
	1318	Marot
	1319	Nargru
	1320	Chamukha Mini Awc
	1321	Raipur-I
	1322	Raipur-Ii
	1323	Raipur-Iii
	1324	Androli
	1325	Dobar-I
	1326	Dobar-Ii
	1327	Proian-I
	1328	Proian-Ii
	1329	Kusiala
	1330	Chaugath
	1331	Lidkot
	1332	Chulhari
	1333	Muslim Kheri
	1334	Gharwasra
	1335	Kyor
	1336	Makrair
	1337	Makrair Sidh
	1338	Balh Saili
	1339	Changer
	1340	Anokha Tanda
	1341	Bihru Kalan
	1342	Nughrari
	1343	Changreri
	1344	Mandli
	1345	Thanakalan
	1346	Majher
	1347	Jhorkhar
	1348	Rachhol
	1349	Chhaproh
	1350	Boosal
	1351	Narghota
	1352	Balh
	1353	Kholi
	1354	Budwar
	1355	Braal
	1356	Doh
	1357	Tihra-1
	1358	Tihra-Ii
	1359	Aghlaur

Field	Question	Answer
		1360 Sakaun<br>1361 New Sakaun<br>1362 Dhwala<br>1363 Dohak<br>1364 Kolka
household_identification > household_identification1		
a_6 *(required)*	Ward/Village<br>वार्ड/गांव	
a_7 *(required)*	City/Town/Tehsil<br>शहर/कस्बा/तहसील	
a_8 *(required)*	Rural/Urban<br>ग्रामीण/शहरी	1 Rural<br>2 Urban
a_9 *(required)*	Address of Household<br>घर का पता	
a_9_1 *(required)*	Location<br>स्थान<br>*GPS coordinates can only be collected when outside.*	
a_10 *(required)*	Contact Number<br>संपर्क<br>*Response constrained to: regex(.,"^[6|7|8|9]\d{9}$")*	
a_11 *(required)*	Alternate contact number<br>वैकल्पिक संपर्क<br>*Response constrained to: regex(.,"^[6|7|8|9]\d{9}$")*	
hhno *(required)*	Household number<br>*Response constrained to: regex(., "^(1[0-9]{2}|7[0-9]{2})$")*	
Respondent		
b	Section-B. Respondent Profile<br>खण्ड-बी. उत्तरदाता प्रोफ़ाइल	
b_1 *(required)*	Name of Respondent<br>उत्तरदाता का नाम<br>*Response constrained to: not(regex(.,'^(.*)\d(.*)$'))*	
b_2 *(required)*	Age of the Respondent<br>उत्तरदाता की आयु<br>*Response constrained to: .>= 18 and .<=100*	
b_3 *(required)*	Gender of Respondent<br>उत्तरदाता का लिंग	1 Male<br>2 Female<br>99 Others
b_4 *(required)*	Occupation	1 Student<br>2 Unemployed<br>3 Homemaker<br>4 Agriculture<br>5 Business/Entrepreneur<br>6 Govt Job<br>7 Pvt Job<br>8 Daily Wage<br>99 Others (specify)
hh_charactersitics		
c_1	Household Charactersitics	
c_2 *(required)*	Type of House<br>घर का प्रकार	1 Yes (Own House)<br>2 No (Rented)
c_3 *(required)*	What is the main source of drinking water for members of your household?<br>आपके घर के सदस्यों के लिए पीने के पानी का मुख्य स्रोत क्या है?	1 Piped Water<br>2 Public Tap<br>3 Tube Well/Borehole/Hand Pump<br>4 Open Well<br>5 Close Well<br>6 Spring<br>7 Rain Water<br>8 Tanker Truck<br>9 Small cart with tank<br>10 Surface Water(River,Lakes,etc)

Field	Question	Answer
		11 Botteld<br>99 Others (specify)
c_4 *(required)*	What type of toilet facility does your household primarily use?<br>आपके घर में मुख्य रूप से किस प्रकार की शौचालय सुविधा का उपयोग किया जाता है?	1 Flush or pour flush toilet<br>2 Pit latrine<br>3 Dry Toilet<br>4 Twin Pit<br>5 No Facility<br>99 Others (specify)
c_5 *(required)*	What type of fuel does the household mainly use for cooking?<br>खाना पकाने के लिए घर मुख्य रूप से किस प्रकार के ईंधन का उपयोग करता है?	1 Electricity<br>2 LPG<br>3 Kerosene<br>4 Coal<br>5 Characoal<br>6 Wood<br>7 Dung Cakes<br>8 Straw<br>9 Biogas<br>10 Solar<br>99 Others (specify)
c_6 *(required)*	Main material of the floor<br>फर्श की मुख्य सामग्री	1 Mud/clay/earth<br>2 Sand<br>3 Dung<br>4 Raw wood planks<br>5 Palm/bamboo<br>6 Brick<br>7 Stone<br>8 Parquet or polished wood<br>9 Vinyl or asphalt<br>10 Ceramic tiles<br>11 Cement<br>12 Polished stone/marble/granite<br>99 Others(specify)
c_7 *(required)*	Main material of the roof<br>छत की मुख्य सामग्री	1 Thatch/palm leaf/reed/grass<br>2 Mud<br>3 Sod/mud and grass mixture<br>4 Plastic/polythene sheeting<br>5 Rustic mat<br>6 Palm/bamboo<br>7 Raw wood planks/timber<br>8 Unburnt brick<br>9 Loosely packed stone<br>10 Metal<br>11 Wood<br>12 Calamine/cement fiber<br>13 Asbestos sheets<br>14 Reinforced cement concrete (RCC)/Reinforced brick concrete/cement/concrete<br>15 Roofing shingles<br>16 Tiles<br>17 Slate<br>18 Burnt brick<br>99 Others(specify)
c_8 *(required)*	Main material of the exterior walls<br>बाहरी दीवारों की मुख्य सामग्री	1 Cane/palm/trunks/bamboo<br>2 Mud<br>3 Grass/reeds/thatch<br>4 Bamboo with mud<br>5 Stone with mud<br>6 Plywood<br>7 Cardboard<br>8 Unburnt brick

Field	Question	Answer
		9 Raw wood/reused wood<br>10 Cement/concrete<br>11 Stone with lime/cement<br>12 Burnt bricks<br>13 Cement blocks<br>14 Wood planks/shingles<br>15 Galvanized iron/metal/asbestos sheets<br>99 Others(specify)
amenities		
intro_ame	Amenities Details<br>सुविधाओं का विवरण	
reserved_name_for_field_list_labels_60		1 Yes<br>2 No
ame1 *(required)*	Electricity<br>बिजली	1 Yes<br>2 No
ame2 *(required)*	Mattress<br>गद्दा	1 Yes<br>2 No
ame3 *(required)*	Pressure cooker<br>प्रेशर-कुकर	1 Yes<br>2 No
ame4 *(required)*	Chair<br>कुर्सी	1 Yes<br>2 No
ame5 *(required)*	Cot or bed<br>खाट या बेड	1 Yes<br>2 No
ame6 *(required)*	Table<br>मेज़	1 Yes<br>2 No
ame7 *(required)*	Electric fan (even not working one)<br>बिजली का पंखा (काम नहीं भी कर रहा हो)	1 Yes<br>2 No
ame8 *(required)*	Radio or transistor (even not working one)<br>रेडियो या ट्रांजिस्टर (काम नहीं भी कर रहा हो)	1 Yes<br>2 No
ame9 *(required)*	Black and white television (even not working one)<br>ब्लैक एण्ड व्हाइट टेलीविजन (काम नहीं भी कर रहा हो)	1 Yes<br>2 No
ame10 *(required)*	Color television (even not working one)<br>रंगीन टेलीविजन (काम नहीं भी कर रहा हो)	1 Yes<br>2 No
ame11 *(required)*	Sewing machine (even not working one)<br>सिलाई मशीन (काम नहीं भी कर रहा हो)	1 Yes<br>2 No
ame12 *(required)*	Working mobile telephone<br>काम कर रहे मोबाइल टेलीफोन	1 Yes<br>2 No
ame13 *(required)*	Smart phone<br>स्मार्ट फोन	1 Yes<br>2 No
ame14 *(required)*	Working landline telephone<br>लैंडलाइन टेलीफोन	1 Yes<br>2 No
ame15 *(required)*	Internet<br>इंटरनेट	1 Yes<br>2 No
ame16 *(required)*	Working computer<br>कंप्यूटर	1 Yes<br>2 No
ame17 *(required)*	Working refrigerator<br>रेफ्रिजरेटर	1 Yes<br>2 No
ame18 *(required)*	Air conditioner/ cooler<br>एयर कंडीशनर/कूलर	1 Yes<br>2 No
ame19 *(required)*	Washing machine<br>वॉशिंग मशीन	1 Yes<br>2 No
ame20 *(required)*	Watch or clock<br>घड़ी	1 Yes<br>2 No
ame21 *(required)*	Bicycle<br>साइकिल	1 Yes<br>2 No
ame22 *(required)*	Motorcycle or scooter<br>मोटरसाइकिल या स्कूटर	1 Yes<br>2 No

Field	Question	Answer
ame23 *(required)*	Animal-drawn cart<br>जानवरों द्वारा खींची जाने वाली गाड़ी	1 Yes<br>2 No
ame24 *(required)*	Car<br>कार	1 Yes<br>2 No
ame25 *(required)*	Water pump<br>पानी का पंप	1 Yes<br>2 No
ame26 *(required)*	Thresher<br>थ्रेशर	1 Yes<br>2 No
ame27 *(required)*	Tractor<br>ट्रैक्टर	1 Yes<br>2 No
fam_charactersitics		
intro_family	Family Characteristics	
d_1 *(required)*	Type of Family<br>परिवार का प्रकार<br>*Nuclear(माता, पिता, बच्चे),Extended(माता, पिता, बच्चे, सास, ससुर),Joint(एक परिवार की कई पीढ़ियाँ, जिनमें दादा-दादी, माता-पिता, बच्चे और कभी-कभी चाचा, चाची और चचेरे भाई-बहन शामिल होते हैं)*	1 Nuclear<br>2 Extended<br>3 Joint
d_2 *(required)*	Number of Family Members<br>परिवार के सदस्यों की संख्या<br>*Those who eat food with same kitchen*<br>*Response constrained to: .>= 1 and .<=20*	
d_3 *(required)*	Religion of the household head<br>घर के मुखिया का धर्म	1 Hindu<br>2 Sikh<br>3 Buddhist<br>4 Christian<br>5 Muslim<br>6 No Religion<br>99 Others (specify)
d_4 *(required)*	What is the caste/tribe of the head of the household?<br>घर के मुखिया की जाति/जनजाति क्या है?	1 SC<br>2 ST<br>3 OBC<br>4 General<br>99 Others(specify)
d_5 *(required)*	Which type of card does household have<br>किस प्रकार की कार्ड परिवार में है<br>*APL-White,BPL-Yellow,AAY*	1 APL<br>2 BPL<br>3 AAY<br>5 PHH(Priority Household)<br>4 No card
d_7 *(required)*	Total Family Income per Year<br>प्रति वर्ष कुल पारिवारिक आय<br>*Income of each family member + Annual Agricultural product if sold + Rent from property + Animal Husbandry and Others*<br>*Response constrained to: .>= 0 and .<=10000000*	
d_6 *(required)*	Is the beneficiary coverd under health insurance<br>क्या लाभार्थी के पास स्वास्थ्य बीमा है	1 Yes<br>2 No
d_6_1 *(required)*	Insurance Type<br>बीमा का प्रकार<br>*Question relevant when: ${d_6} =1*	1 Goverment(AYUSHMAN Bharat/ESI)<br>2 Private
eligible_cirteria		
d_8 *(required)*	Married Preconception Women with no or one child(18-35)<br>*Married Woman with in age group 18-35 with no or one child and planning to get pregnant*	1 Yes<br>2 No
d_9 *(required)*	Pregnant Women<br>गर्भवती महिला	1 Yes<br>2 No
d_10 *(required)*	Postnatal Mother and child (0-6 months)<br>प्रसवोत्तर माँ और बच्चा (0-6 महीने)<br>*Woman who delivered in last 6 months*	1 Yes<br>2 No
d_11 *(required)*	Children between 6-24 months<br>6-24 महीने का बच्चा	1 Yes<br>2 No
preconception_group<br>*Group relevant when: ${d_8} =1*		
preconception_group > prec_group_info		
preconception_care_note	Preconception Care Status of Reproductive Married Females(18-35)	
e_2 *(required)*	ID of the Woman<br>महिला की पहचान	

Field	Question	Answer
	Please verify with the given list of IDs<br>*Response constrained to: regex(., '^(?!200$)[2][0-9]{2}$')*	
e_1 *(required)*	Name of the Woman<br>महिला का नाम<br>*Response constrained to: not(regex(.,'^(.*)\d(.*)$'))*	
e_3 *(required)*	Age of the Woman<br>महिला की उम्र<br>*Response constrained to: .>= 18 and .<=35*	
e_6 *(required)*	How many years of schooling has the woman completed?<br>महिला ने कितने साल की स्कूली शिक्षा पूरी की है?<br>*Response constrained to: .>= 0 and .<=25*	
e_7 *(required)*	What is the current occupation of the woman?<br>महिला का वर्तमान व्यवसाय क्या है?	1 Student<br>2 Unemployed<br>3 Homemaker<br>4 Agriculture<br>5 Business/Entrepreneur<br>6 Govt Job<br>7 Pvt Job<br>8 Daily Wage<br>99 Others (specify)
e_4 *(required)*	Name of the Husband<br>पति का नाम<br>*Response constrained to: not(regex(.,'^(.*)\d(.*)$'))*	
e_5 *(required)*	Age of the Husband<br>पति की उम्र<br>*Response constrained to: .>= 18 and .<=55*	
e_8 *(required)*	How many years of schooling has the husband completed?<br>पति ने कितने साल की स्कूली शिक्षा पूरी की है?<br>*Response constrained to: .>= 0 and .<=25*	
e_9 *(required)*	What is the current occupation of the husband?<br>पति का वर्तमान व्यवसाय क्या है?	1 Student<br>2 Unemployed<br>3 Homemaker<br>4 Agriculture<br>5 Business/Entrepreneur<br>6 Govt Job<br>7 Pvt Job<br>8 Daily Wage<br>99 Others (specify)
e_17_1 *(required)*	Do you have access of the phone through out the day?<br>क्या आप के पास व्यक्तिगत फोन है या दिन भर फ़ोन आप के पास रहता हैं	1 Yes (own phone)<br>3 Yes (Family member phone)<br>2 No
e_17_2 *(required)*	Type of phone<br>*Question relevant when: ${e_17_1} =1 or ${e_17_1} =3*	1 Yes (Normal Button Phone)<br>2 Yes (Android)<br>3 Yes (Apple)
e_17_3 *(required)*	Timing of phone access in a day<br>*Fill in the hours*<br>*Question relevant when: ${e_17_1} =3*<br>*Response constrained to: .>= 0 and .<=24*	
preconception_group > Preconception		
e_12 *(required)*	How many times woman become pregnant(G)<br>महिला कितनी बार गर्भवती हुई है(G)<br>*Response constrained to: .>= 0 and .<=12*	
e_13 *(required)*	How many times pregnancy crossed 28 weeks of Gestation(P)<br>कितनी बार गर्भावस्था ने गर्भधारण के 28 सप्ताह को पार किया(P)<br>*Response constrained to: ${e_13} <= ${e_12}*	
e_14 *(required)*	Pregnancy loss before 28 weeks of Gestation (A)<br>गर्भधारण के 28 सप्ताह से पहले गर्भावस्था समाप्त हुई (A)<br>*Response constrained to: ${e_14} <= ${e_12}*	
e_15 *(required)*	Number of living children (L)<br>जीवित बच्चों की संख्या (L)<br>*Response constrained to: ${e_15} <= 1*	
e_15_note	Health Screening and Anthropometric Measurements of Women<br>महिलाओं की स्वास्थ्य जांच और एंथ्रोपोमेट्री माप	

Field	Question	Answer
e_16_1 *(required)*	Height 1<br>ऊंचाई 1<br>*Please enter a value between 110.0 – 198.0 cm, NA = No*<br>*Response constrained to: ((${e_16_1} != 'NA' and regex(${e_16_1} , '^\d{1,3}\.\d{1}$') and number(${e_16_1}) >= 110 and number(${e_16_1}) <= 198) or ${e_16_1} = 'NA')*	
e_16_1_1 *(required)*	Height 2<br>ऊंचाई 2<br>*Response constrained to: ((${e_16_1_1} != 'NA' and regex(${e_16_1_1} , '^\d{1,3}\.\d{1}$') and number(${e_16_1_1}) >= 110 and number(${e_16_1_1}) <= 198) or ${e_16_1_1} = 'NA')*	
e_16_2 *(required)*	Weight 1<br>वजन 1<br>*Please enter a value between 25.0 – 120.0 kg, NA = No*<br>*Response constrained to: ((${e_16_2} != 'NA' and regex(${e_16_2} , '^\d{1,3}\.\d{1}$') and number(${e_16_2}) >= 25.0 and number(${e_16_2}) <= 120) or ${e_16_2} = 'NA')*	
e_16_2_1 *(required)*	Weight 2<br>वजन 2<br>*Response constrained to: ((${e_16_2_1} != 'NA' and regex(${e_16_2_1} , '^\d{1,3}\.\d{1}$') and number(${e_16_2_1}) >= 25.0 and number(${e_16_2_1}) <= 120) or ${e_16_2_1} = 'NA')*	
e_16_6 *(required)*	Hemoglobin (Hb) Level<br>हीमोग्लोबिन (Hb) स्तर<br>*Please enter a value between 2.5 – 20.0, NA = No*<br>*Response constrained to: ((${e_16_6} != 'NA' and regex(${e_16_6} , '^\d{1,2}\.\d{1}$') and number(${e_16_6}) >= 2.5 and number(${e_16_6}) <= 20) or ${e_16_6} = 'NA')*	
e_17 *(required)*	Does the woman consume the following food?<br>क्या महिला निम्नलिखित भोजन का सेवन करती है?	1 Eggs<br>2 Milk<br>3 Mutton<br>4 Fish<br>5 Chicken<br>6 Vegetable/Vegetarian food<br>9999 Other animal food<br>99 Other specify
e_37_1 *(required)*	In the last one month, have you been diagnosed or suffered from any of the following condition/Illness?<br>पिछले एक महीने में, क्या आपका निम्नलिखित में से किसी भी स्थिति / बीमारी का निदान किया गया है या आप इनसे ग्रासित हुई हैं?<br>*पेशाब वाली जगह से दुर्गंध, बदबूदार सफेद पानी आना, पेट के निचले हिस्से में दर्द, पेशाब करते समय जलन होना,गुप्तांग पर घाव या छाले!*<br>*Response constrained to: not(selected(${e_37_1} , '9999') and count-selected(${e_37_1}) > 1) or not(selected(${e_37_1} , '9999'))*	1 Hypothyroidism<br>2 Reproductive tract infection(RTI)/Sexually Transmitted Infection(STI)<br>3 Diabetes<br>4 Hypertension<br>5 Anaemia<br>7 UTI(Urinary Tract Infection)<br>8 TB(Tuberculosis)<br>99 Others(specify)<br>9999 No Disease/Illness
e_37_1_source *(required)*	Source document of the problem<br>*Question relevant when: not(selected(${e_37_1} , '9999'))*	1 Reported by Beneficiary<br>2 Verified from document
e_37_1_source1 *(required)*	Type of document<br>*custom-specify-other(other=99)*<br>*Question relevant when: ${e_37_1_source} =2*	1 Prescription<br>2 Lab report<br>3 Government card(MCP)<br>99 Other
e_38_d *(required)*	Did you seek treatment outside of home<br>क्या आपने घर के बाहर इलाज कराया<br>*Question relevant when: ${e_37_1} !=9999*	1 Yes<br>2 No
e_39_d1 *(required)*	From whom?<br>किससे?<br>*Question relevant when: ${e_38_d} =1*	1 HWC<br>2 CHC<br>3 PHC<br>4 Civil Hospital(CH)<br>5 Regional Hospital<br>6 ASHA<br>7 ANM<br>8 Medical college<br>9 Private provider clinic(Without in-patient bed)<br>10 Private hospital(With in-patient bed)

Field	Question	Answer
		11 Faith healers/traditional health care providers<br>99 Others(Specify)
e_40_d2 *(required)*	Do you have any treatment related documents(investigation reports, prescriptions, if admitted discharge summary, tablet strips, syrup bottles) क्या आपके पास उपचार से संबंधित कोई दस्तावेज हैं<br>*Question relevant when: ${e_38_d} =1*<br>*Response constrained to: not(selected(${e_40_d2} , '3') and count-selected(${e_40_d2}) > 1) or not(selected(${e_40_d2} , '3'))*	1 Documents<br>2 Medicine(Tablets strips/Syrup)<br>3 None of the above
e_40_d2_1 *(required)*	Upload Document<br>दस्तावेज़ अपलोड करें<br>*Question relevant when: selected(${e_40_d2} , '1')*	
e_40_d2_2 *(required)*	Mention the name of medicine<br>दवा के नाम का उल्लेख करें<br>*Question relevant when: selected(${e_40_d2} , '2')*	
e_18 *(required)*	Were you examined by any government worker in the last 6 months (to be asked at baseline) and in the last 3 months (to be asked at 3 monthly surveys). If yes, ask and check from available records.<br>क्या आपने पिछले 6 महीनों में (बेसलाइन पर पूछे जाने के लिए) और पिछले 3 महीनों में (3 मासिक सर्वेक्षणों में पूछे जाने के लिए) किसी सरकारी स्वास्थ्य कार्यकर्ता द्वारा जांच की थी। यदि हां, तो उपलब्ध रिकॉर्ड से पूछें और जांचें।	1 Reported by Beneficiary<br>2 Verified from document<br>3 No
e_18_1_source1 *(required)*	Type of document<br>*Question relevant when: ${e_18} =2*	1 Prescription<br>2 Lab report<br>3 Government card(MCP)<br>99 Other
preconception_group > health_group<br>*Group relevant when: selected(${e_18} , '2')*		
intro_note_e19	Fill the value from the Health records available(Document)<br>उपलब्ध स्वास्थ्य रिकॉर्ड से भरें	
e_19 *(required)*	Height<br>ऊंचाई<br>*Please enter a value between 110.0 – 198.0 cm, NA = No*<br>*Response constrained to: ((${e_19} != 'NA' and regex(${e_19} , '^\d{1,3}\.\d{1}$') and number(${e_19}) >= 110 and number(${e_19}) <= 198) or ${e_19} = 'NA')*	
e_20 *(required)*	Weight<br>वजन<br>*Please enter a value between 25.0 – 120.0 kg, NA = No*<br>*Response constrained to: ((${e_20} != 'NA' and regex(${e_20} , '^\d{1,3}\.\d{1}$') and number(${e_20}) >= 25 and number(${e_20}) <= 120) or ${e_20} = 'NA')*	
e_21 *(required)*	Blood test for Hb<br>एचबी के लिए रक्त परीक्षण<br>*Please enter a value between 2.5 – 20.0, NA = No*<br>*Response constrained to: ((${e_21} != 'NA' and regex(${e_21} , '^\d{1,2}\.\d{1}$') and number(${e_21}) >= 2.5 and number(${e_21}) <= 20) or ${e_21} = 'NA')*	
e_22	Blood test for diabetes(Fasting,Random,OGTT,HbA1c)<br>मधुमेह के लिए रक्त परीक्षण	
e_22_1 *(required)*	Fasting Blood sugar<br>*Please enter a value between 20.0 to 500.0, NA = No*<br>*Response constrained to: ((${e_22_1} != 'NA' and regex(${e_22_1} , '^\d{1,3}\.\d{1}$') and number(${e_22_1}) >= 20 and number(${e_22_1}) <= 500) or ${e_22_1} = 'NA')*	
e_22_2 *(required)*	Random blood sugar<br>*Please enter a value between 20.0 to 500.0, NA = No*<br>*Response constrained to: ((${e_22_2} != 'NA' and regex(${e_22_2} , '^\d{1,3}\.\d{1}$') and number(${e_22_2}) >= 20 and number(${e_22_2}) <= 500) or ${e_22_2} = 'NA')*	
e_22_3 *(required)*	Oral glucose tolerance test(OGTT)<br>*Please enter a value between 20.0 to 500.0, NA = No*<br>*Response constrained to: ((${e_22_3} != 'NA' and regex(${e_22_3} , '^\d{1,3}\.\d{1}$') and number(${e_22_3}) >= 20 and number(${e_22_3}) <= 500) or ${e_22_3} = 'NA')*	
e_22_4 *(required)*	HbA1c (%)<br>*Please enter a value between 1.0 to 12.0, NA = No*<br>*Response constrained to: ((${e_22_4} != 'NA' and regex(${e_22_4} , '^\d{1,2}\.\d{1}$') and number(${e_22_4}) >= 1 and number(${e_22_4}) <= 12) or ${e_22_4} = 'NA')*	
e_23	Blood test for thyroid<br>थायराइड के लिए रक्त परीक्षण	
e_23_1 *(required)*	TSH(Thyroid-stimulating hormone)<br>टीएसएच	1 µg/dl<br>2 µIU/mL
e_23_dl	µg/dl<br>*Please enter a value between 0.1 to 20.0, NA = No*	

Field	Question	Answer
	Response constrained to: ((regex(., '^[0-9]+(\.[0-9]+)?$') and number(.) >= 0 and number(.) <= 20) or . = 'NA') and (${e_23_1} = 1 or . = 'NA')	
e_23_ml	μIU/mL<br>*Please enter a value between 0.1 to 20.0, NA = No*<br>*Response constrained to: ((regex(., '^[0-9]+(\.[0-9]+)?$') and number(.) >= 0 and number(.) <= 20) or . = 'NA') and (${e_23_1} = 2 or . = 'NA')*	
e_23_2 *(required)*	T3(ng/dl)<br>टी3(ng/dl)<br>*Please enter a value between 0 to 300.0, NA = No*<br>*Response constrained to: ((${e_23_2} != 'NA' and regex(${e_23_2} , '^\d{1,3}(\.\d+)?$') and number(${e_23_2}) >= 0 and number(${e_23_2}) <= 300) or ${e_23_2} = 'NA')*	
e_23_3 *(required)*	T4(μg/dl)<br>टी4(μg/dl)<br>*Please enter a value between 0.1 to 20.0, NA = No*<br>*Response constrained to: ((${e_23_3} != 'NA' and regex(${e_23_3} , '^\d{1,1}\.\d{1}$') and number(${e_23_3}) >= 0.1 and number(${e_23_3}) <= 20) or ${e_23_3} = 'NA')*	
preconception_group > e_group<br>*Group relevant when: selected(${e_18} , '1')*		
e_26_1	Were you told of your health condition?<br>क्या आपको आपकी स्वास्थ्य स्थिति के बारे में बताया गया?	
reserved_name_for_field_list_labels_175		1 Yes<br>2 No
e_25 *(required)*	Asked about RTI/STI symptoms?<br>आरटीआई/एसटीआई के लक्षणों के बारे में पूछा गया?<br>*पेशाब वाली जगह से दुर्गंध, बदबूदार सफेद पानी आना, पेट के निचले हिस्से में दर्द, पेशाब करते समय जलन होना,गुप्तांग पर घाव या छाले*	1 Yes<br>2 No
e_26 *(required)*	Asked about UTI symptoms?<br>यूटीआई के लक्षणों के बारे में पूछा गया?	1 Yes<br>2 No
e_27 *(required)*	Low BMI<br>कम बीएमआई	1 Yes<br>2 No
e_28 *(required)*	Anemia<br>रक्ताल्पता<br>*Anemia < 12 gm/dl*	1 Yes<br>2 No
e_28_1 *(required)*	Abnormal thyroid status (Hyperthyroidism/Hypothyroidism)<br>असामान्य थायराइड स्थिति (हाइपरथायरायडिज्म/हाइपोथायरायडिज्म)	1 Yes<br>2 No
e_29 *(required)*	Hypertension<br>उच्च रक्तचाप	1 Yes<br>2 No
e_30 *(required)*	Diabetes<br>डायबिटीज़	1 Yes<br>2 No
e_33 *(required)*	Inadequate weight gain (IWG)<br>अपर्याप्त वजन बढ़ना (IWG)<br>*If weight gain is < 500 gram per month for those having BMI < 18.5*	1 Yes<br>2 No
e_34 *(required)*	Other Specify<br>*If no please fill NA*<br>*Question relevant when: selected(${e_18} , '1')*	
e_41_d3 *(required)*	Were you admitted to hospital in the last 3 months?<br>क्या आप पिछले 3 महीनों में अस्पताल में भर्ती हुये थे?	1 Yes<br>2 No
e_42_d4 *(required)*	For what condition have you been admited<br>आपको किस स्थिति के लिए भर्ती कराया गया है<br>*Question relevant when: ${e_41_d3} =1*<br>*Response constrained to: not(regex(.,'^(.*)\d(.*)$'))*	
e_38 *(required)*	Were you ever referred for any health/nutrition related condition?<br>क्या आपको कभी किसी स्वास्थ्य / पोषण संबंधी स्थिति के लिए रेफर किया गया था?<br>*बीमारी या कमजोरी के कारण रेफरल*	1 Yes<br>2 No
preconception_group > Health/nutrition condition for referral<br>स्वास्थ्य/पोषण स्थिति जिसके लिए रेफर किया गया<br>*Group relevant when: selected(${e_38} ,1)*		
e_38_1 *(required)*	Where were you referred?<br>आपको कहां रेफर किया गया था?	1 HWC<br>2 CHC<br>3 PHC<br>4 Civil Hospital(CH)<br>5 Regional Hospital<br>6 ASHA<br>7 ANM<br>8 Medical college

Field	Question	Answer
		9 Private provider clinic(Without in-patient bed)<br>10 Private hospital(With in-patient bed)<br>11 Faith healers/traditional health care providers<br>99 Others(Specify)
e_38_reason *(required)*	Reason for referral<br>*Question relevant when: ${e_38} =1*	
e_38_3 *(required)*	Did you comply with the referral.<br>क्या आपने रेफरल का अनुपालन किया?	1 Yes<br>2 No
preconception_group > referral_comply<br>*Group relevant when: selected(${e_38_3} ,1)*		
e_38_4 *(required)*	Where did you go?<br>आप कहाँ गए थे?	1 HWC<br>2 CHC<br>3 PHC<br>4 Civil Hospital(CH)<br>5 Regional Hospital<br>6 ASHA<br>7 ANM<br>8 Medical college<br>9 Private provider clinic(Without in-patient bed)<br>10 Private hospital(With in-patient bed)<br>11 Faith healers/traditional health care providers<br>99 Others(Specify)
e_38_5 *(required)*	Did you receive any treatment?<br>क्या आपको कोई इलाज मिला?	1 Yes<br>2 No
e_38_5_1 *(required)*	Was the condition better/cured?<br>क्या स्थिति बेहतर / ठीक हो गई थी?	1 Yes<br>2 No
e_38_6 *(required)*	Did you face any challenges to comply with referral or at the referral facility?<br>क्या आपको रेफरल के दौरान या रेफरल केन्द्र पर सुविधा लेने में किसी भी चुनौती का सामना करना पड़ा?	1 Yes<br>2 No
e_38_7 *(required)*	What challenges you did you face?<br>आपको किन चुनौतियों का सामना करना पड़ा?<br>*Question relevant when: selected(${e_38_6} ,1)*	1 Lack of transportation<br>2 Too far, long difficult road<br>3 Could not afford<br>4 No one to accompany<br>5 Referral facility not good<br>6 Don't think I need referral<br>99 Others (specify)
e_36 *(required)*	Which of the following health facilities do you visit or use for health or nutrition-related care?<br>आप निम्नलिखित में से किस स्वास्थ्य केन्द्र पर जाते हैं या स्वास्थ्य या पोषण संबंधी देखभाल के लिए उपयोग करते हैं?	1 HWC<br>2 CHC<br>3 PHC<br>4 Civil Hospital(CH)<br>5 Regional Hospital<br>6 ASHA<br>7 ANM<br>8 Medical college<br>9 Private provider clinic(Without in-patient bed)<br>10 Private hospital(With in-patient bed)<br>11 Faith healers/traditional health care providers<br>99 Others(Specify)
preconception_group > health_center_group		
e_31_0	What are the services that are available or services that you utilize at the government health centers?<br>सरकारी स्वास्थ्य केंद्रों पर आप कौन-कौन सी सेवाएं हैं या आप किन सेवाओ उपयोग करते हैं?<br>*Last six months*	
reserved_name_for_field_list_labels_207		1 Available

Field	Question	Answer
		2 Available, utilized<br>3 Not Available<br>4 No knowledge
e_31_1_1 *(required)*	Family Planning<br>परिवार-नियोजन	1 Available<br>2 Available, utilized<br>3 Not Available<br>4 No knowledge
e_31_1_2 *(required)*	Treatment for health condition<br>स्वास्थ्य ख़राब होने की स्थिति के लिए उपचार	1 Available<br>2 Available, utilized<br>3 Not Available<br>4 No knowledge
e_31_1_4 *(required)*	Provision IFA tablets<br>आईएफए टैबलेट<br>*If Utilized Please Check prescription/Strips/Bottle or ask woman*	1 Available<br>2 Available, utilized<br>3 Not Available<br>4 No knowledge
e_31_1_5 *(required)*	Provision multiple micronutrients<br>*If Utilized Please Check prescription/Strips/Bottle or ask woman*	1 Available<br>2 Available, utilized<br>3 Not Available<br>4 No knowledge
e_31_1_6 *(required)*	Provision Albendazole/deworming medicines<br>एल्बेंडाजोल/कृमि मुक्ति दवा	1 Available<br>2 Available, utilized<br>3 Not Available<br>4 No knowledge
e_31_1_8 *(required)*	Counselling (WaSH, Menstrual hygiene)<br>परामर्श (WaSH, Menstrual hygiene)	1 Available<br>2 Available, utilized<br>3 Not Available<br>4 No knowledge
e_31_1_9 *(required)*	Mental health counseling<br>मानसिक स्वास्थ्य के लिये परामर्श/सलाह	1 Available<br>2 Available, utilized<br>3 Not Available<br>4 No knowledge
e_31_1_10 *(required)*	Diet nutrition counselling<br>आहार और पोषण के लिये परामर्श	1 Available<br>2 Available, utilized<br>3 Not Available<br>4 No knowledge
preconception_group > health_screening_group		
health_screening	Health screening in goverment health facilities	
reserved_name_for_field_list_labels_218		1 Available<br>2 Available,Utilize<br>3 Not Available<br>4 No Knowledge
health_wings_1 *(required)*	Diabetes	1 Available<br>2 Available,Utilize<br>3 Not Available<br>4 No Knowledge
health_wings_2 *(required)*	Hypertension	1 Available<br>2 Available,Utilize<br>3 Not Available<br>4 No Knowledge
health_wings_3 *(required)*	Height	1 Available<br>2 Available,Utilize<br>3 Not Available<br>4 No Knowledge
health_wings_4 *(required)*	Weight	1 Available<br>2 Available,Utilize<br>3 Not Available<br>4 No Knowledge
health_wings_5 *(required)*	Thyroid	1 Available<br>2 Available,Utilize<br>3 Not Available

Field	Question	Answer
		4 No Knowledge
health_wings_6 *(required)*	Anemia	1 Available<br>2 Available,Utilize<br>3 Not Available<br>4 No Knowledge
health_wings_7 *(required)*	RTI/STI	1 Available<br>2 Available,Utilize<br>3 Not Available<br>4 No Knowledge
health_wings_8 *(required)*	Oral Health	1 Available<br>2 Available,Utilize<br>3 Not Available<br>4 No Knowledge
e_d_p_19 *(required)*	Do you get any food from the AWC for yourself?<br>क्या आप अपने लिए आंगनवाड़ी केंद्र से कोई भोजन/ राशन प्राप्त करते हैं?	1 Yes<br>2 No
e_d_p_20 *(required)*	Was the food provided by the AWC entirely consumed by the beneficiary<br>क्या आंगनवाड़ी केंद्र द्वारा उपलब्ध कराया गया भोजन पूरी तरह से लाभार्थी द्वारा खाया गया था?<br>*Question relevant when: ${e_d_p_19} =1*	1 Yes<br>2 No
preconception_group > Supplementary Nutrition<br>*Group relevant when: ${e_d_p_19} =1*		
preconception_group > Supplementary Nutrition > food_practices2_1_1_awc_a		
j_1_3_1_awc_a *(required)*	Type of Food Received<br>प्रदान किए गए भोजन का प्रकार	1 Hot Cooked Meal<br>2 THR
preconception_group > Supplementary Nutrition > food_practices2_1_1_awc_a > hcm_1_awc_a<br>*Group relevant when: selected(${j_1_3_1_awc_a} , '1')*		
hcm_1_note_awc_a	Hot Cooked Meal<br>गर्म पका हुआ भोजन	
hot_cooke_name_a *(required)*	Specify Hot cooked meal name	
j_1_13_1_awc_a *(required)*	Quantity consumed per day(in gram)<br>प्रति दिन खायी गयी मात्रा (ग्राम में)<br>*Response constrained to: .>= 50 and .<=200*	
j_1_14_1_awc_a *(required)*	Variation in Quantity/Type Based on Beneficiary Condition<br>लाभार्थी की स्थिति के आधार पर मात्रा/प्रकार में भिन्नता<br>*If yes, specify*	1 Yes<br>2 No
preconception_group > Supplementary Nutrition > food_practices2_1_1_awc_a > thr_1_awc_a<br>*Group relevant when: selected(${j_1_3_1_awc_a} , '2')*		
thr_text1_awc_a *(required)*	THR (Take home ration) THR	1 Channa<br>2 Dalia<br>3 Jaggary<br>4 Oil<br>5 Panjiri<br>6 Rajma<br>7 Rice<br>8 Salt<br>9 Sevia<br>10 Soya<br>11 Salt Biscuit<br>12 Sweet Biscuit<br>13 WMP(Milk)<br>14 Milk<br>15 Egg<br>26 Black Chana<br>27 Chane Ki Daal<br>16 Wings1<br>17 Wings2<br>18 Wings3<br>19 Wings4<br>20 Wings5<br>21 Wings6<br>22 Wings7<br>23 Wings8

Field	Question	Answer
		24 Wings9<br>25 Wings10<br>99 Other
preconception_group > Supplementary Nutrition > food_practices2_1_1_awc_a > thr_1_awc_a > THR [thr_cal_repeat_a] (1)		(Repeated group)
hot_total_a *(required)*	Total Quantity receive in a month(in gram)<br>*Response constrained to: .>= 0*	
week_a *(required)*	Receive for how many days in a week<br>*Response constrained to: .>= 0 and .<=7*	
quantity_p3_a *(required)*	Quantity receive per day(in gram)<br>प्रति दिन कितना मिला (ग्राम में)<br>*Response constrained to: .>= 0 and .<=200*	
j_1_17_1_awc_a *(required)*	Quantity Consumed in gram (Per Day)<br>प्रति दिन खायी गयी मात्रा (ग्राम में)<br>*Response constrained to: .>= 0 and .<=200*	
j_1_18_1_awc_a *(required)*	Variation in Quantity/Type Based on Beneficiary Condition<br>लाभार्थी की स्थिति के आधार पर मात्रा/प्रकार में भिन्नता<br>*If yes, specify*	1 Yes<br>2 No
thr_month *(required)*	Did AWW tell the beneficiary about consumption of THR/ how to consume it in a month?	1 Yes<br>2 No
thr_remarks *(required)*	Remark for THR	
e_d_p_19_no *(required)*	Reason for not getting food from the aganwawdi<br>अगनबाड़ी से भोजन/राशन न मिलने का कारण<br>*Question relevant when: ${e_d_p_19} =2*	1 Preconception women not registered at AWCs<br>99 Other(Specify)
e_d_p_21 *(required)*	Did they share it with family members?<br>*Question relevant when: ${e_d_p_20} =1*	1 Yes<br>2 No
f_32_1 *(required)*	Do you face any challenges in receiving food from the AWC?<br>क्या आपको आंगनवाड़ी केंद्र से भोजन/राशन प्राप्त करने में किसी चुनौती का सामना करना पड़ता है?<br>*Question relevant when: selected(${e_d_p_19} ,1)*	1 Yes<br>2 No
f_33 *(required)*	What were the challenges?<br>चुनौतियां क्या हैं?<br>*Question relevant when: selected(${f_32_1} ,1)*	1 Food not available<br>2 Difficult to get it every day<br>3 Milk/egg not given<br>4 Quantity less than what was told to<br>5 Poor quality food<br>99 Others(specify)
f_34_a *(required)*	Do you face any challenges in consuming of AWC food?<br>*Question relevant when: selected(${e_d_p_19} ,1)*	1 Yes<br>2 No
f_1_34_challenges_a *(required)*	What were the challenges?<br>चुनौतियां क्या थीं?<br>*Question relevant when: ${f_33_1} =1*	
preconception_group > supplements_consumed		
f_31_a *(required)*	Have you consumed or are you presently consuming any of the following?<br>क्या आप निम्नलिखित में से किसी का सेवन किया है या कर रहे हैं?<br>*In the last 6 month*<br>*Response constrained to: not(selected(${f_31_a} , '9999') and count-selected(${f_31_a}) > 1) or not(selected(${f_31_a} , '9999'))*	1 Iron and Folic Acid (IFA) tablets<br>8 Iron and Folic Acid (IFA) syrup<br>7 Folic Acid<br>2 Combination of Calcium & Vitamin-D<br>3 Calcium Tablet<br>4 Vitamin-D<br>5 Multiple Micronutrient Supplements (MMS)<br>6 Multi Vitamin<br>99 Others(medicinal suppliment)<br>9999 None
preconception_group > supplements_consumed > f_med_status_g_a<br>*Group relevant when: not(selected(${f_31_a} , '9999'))*		
preconception_group > supplements_consumed > f_med_status_g_a > [med_names_a] (1)		(Repeated group)
med_status_a *(required)*	When did you consume [med_names_a]?<br>आपने [med_names_a] की गोली कब ली है?	1 Consumed in the past पहले ली थी<br>2 Currently consuming अभी ले रही

Field	Question	Answer
f_33_4_4_a *(required)*	Since you started, for how many total days have you consumed [med_names_a]?<br>(Approximate number of days)<br>[med_names_a] या गोली लेना शुरू करने के बाद से अब तक आपने कुल कितने दिनों तक गोली ली है?<br>(कृपया अनुमानित दिनों की संख्या बताएं)<br>*Response constrained to: .>= 1 and .<=180*	
f_33_4_5_a *(required)*	How many days in a week do/did you consume [med_names_a]?<br>(Enter number of days: 0 to 7)<br>आप एक सप्ताह में कितने दिन [med_names_a] या गोली लेती हैं/थीं?<br>(दिनों की संख्या दर्ज करें: 0 से 7)<br>*Response constrained to: .>= 1 and .<=7*	
f_33_4_1_a *(required)*	How many [med_names_a] tablets do/did you consume in a day?<br>आप एक दिन में कितनी [med_names_a] या गोलियाँ लेती हैं/थीं?<br>*Response constrained to: .>= 1 and .<=3*	
f_33_4_2_a *(required)*	What is your [med_names_a] consumption pattern?	1 Regularly<br>2 Irregularly
f_text_a *(required)*	If you consume or consumed [med_names_a] irregularly, what are the reasons for missing the tablets?<br>अगर आप अनियमित रूप से [med_names_a] लेती हैं/थीं, तो Syrup या गोली न लेने के क्या कारण हैं	
source_of_supp *(required)*	[med_names_a] Received from	1 Received from Goverment<br>2 Purchase from Private
albenda1 *(required)*	Do you consume albendazole tablet	1 Yes<br>2 No
date_albanda_a *(required)*	When was the last time albendazole tablet was consumed?<br>आखिरी बार एल्बेंडाजोल टैबलेट का सेवन कब किया गया था?<br>*Question relevant when: ${albenda1} =1*	
preconception_group > diet_group_pre		
e_d_p	What do you usually have in your diet and how many times in a week? आप आमतौर पर अपने आहार में क्या लेते हैं और सप्ताह में कितनी बार?	
e_d_p_1 *(required)*	Cereal (rice/wheat)<br>अनाज (चावल/गेहूं)<br>*No of times*<br>*Response constrained to: .>= 0 and .<=21*	
e_d_p_2 *(required)*	Legumes /Lentils(beans, peas)<br>फलियां / दाल (बीन्स, मटर)<br>*No of times*<br>*Response constrained to: .>= 0 and .<=21*	
e_d_p_3 *(required)*	Green leafy vegetables<br>हरी पत्तेदार सब्जियां<br>*No of times*<br>*Response constrained to: .>= 0 and .<=21*	
e_d_p_4 *(required)*	Other vegetables<br>अन्य सब्जियां<br>*No of times*<br>*Response constrained to: .>= 0 and .<=21*	
e_d_p_5 *(required)*	Fruits (guava, orange, apple, pomegranate)<br>फल (अमरूद, संतरा, सेब, अनार)<br>*No of times*<br>*Response constrained to: .>= 0 and .<=21*	
e_d_p_6 *(required)*	Meat<br>मांस<br>*No of times*<br>*Response constrained to: .>= 0 and .<=21*	
e_d_p_7 *(required)*	Fish<br>मछली<br>*No of times*<br>*Response constrained to: .>= 0 and .<=21*	
e_d_p_8 *(required)*	Eggs<br>अंडे<br>*No of times*<br>*Response constrained to: .>= 0 and .<=21*	
e_d_p_9 *(required)*	Milk<br>दूध<br>*No of times*<br>*Response constrained to: .>= 0 and .<=21*	
e_d_p_10 *(required)*	Milk products (yogurt, paneer)<br>दूध उत्पाद (दही, पनीर)<br>*No of times*	

Field	Question	Answer
	Response constrained to: .>= 0 and .<=21	
e_d_p_11 *(required)*	Jaggery (gudd/Shakkar)<br>गुड़ (गुड़)<br>*No of times*<br>*Response constrained to: .>= 0 and .<=21*	
e_d_p_12 *(required)*	Red meat<br>लाल मांस (मटन)<br>*No of times*<br>*Response constrained to: .>= 0 and .<=21*	
e_d_p_13 *(required)*	Millets (ragi, bajra, jawar)<br>बाजरा (रागी, बाजरा, ज्वार)<br>*No of times*<br>*Response constrained to: .>= 0 and .<=21*	
e_d_p_14 *(required)*	Soy product<br>सोया उत्पाद<br>*No of times*<br>*Response constrained to: .>= 0 and .<=21*	
e_d_p_15 *(required)*	Lemon/amla<br>नींबू/आंवला<br>*No of times*<br>*Response constrained to: .>= 0 and .<=21*	
e_d_p_22 *(required)*	Nuts and oil seeds<br>मेवे और तेल के बीज<br>*No of times<br>(Eg: Almond, Walnuts, Peanut, Sesame, Sunflower seeds etc)*<br>*Response constrained to: .>= 0 and .<=42*	
e_d_p_23 *(required)*	Fats and oils<br>वसा और तेल<br>*No of times<br>(Butter, Ghee, Mustard oil, Coconut oil etc.)*<br>*Response constrained to: .>= 0 and .<=42*	
e_d_p_16 *(required)*	Any fortified rice/wheat<br>कोई भी फोर्टिफाइड चावल/गेहूं<br>*No of times*<br>*Response constrained to: .>= 0 and .<=21*	
e_d_p_18 *(required)*	Other fortified foods<br>अन्य फोर्टिफाइड खाद्य पदार्थ<br>*No of times*<br>*Response constrained to: .>= 0 and .<=21*	
prec_remarks *(required)*	Respondent remarks<br>*Question relevant when: ${d_8} =1*	
prec_interviewer_remark *(required)*	Interviewer remarks<br>*Question relevant when: ${d_8} =1*	
Pregnant Women<br>गर्भवती महिला<br>*Group relevant when: ${d_9} =1*		
Pregnant Women<br>गर्भवती महिला > preg_women_group		
f_2 *(required)*	ID of the woman<br>महिला की आईडी<br>*Please verify with the given list of IDs*<br>*Response constrained to: regex(., '^(?!300$)[3][0-9]{2}$')*	
f_1 *(required)*	Name of the woman<br>महिला का नाम<br>*Response constrained to: not(regex(.,'^(.*)\d(.*)$'))*	
f_3 *(required)*	Age of the woman<br>महिला की उम्र<br>*Response constrained to: .>= 18 and .<=45*	
f_3_1 *(required)*	Name of husband<br>पति का नाम<br>*Response constrained to: not(regex(.,'^(.*)\d(.*)$'))*	
f_4 *(required)*	Age of the husband<br>पति की उम्र<br>*Response constrained to: .>= 18 and .<=55*	
f_5 *(required)*	How many years of schooling has the woman completed?<br>महिला ने कितने साल की स्कूली शिक्षा पूरी की है?<br>*Response constrained to: .>= 0 and .<=25*	
f_6 *(required)*	Occupation of the woman<br>महिला का व्यवसाय	1 Student<br>2 Unemployed<br>3 Homemaker

Field	Question	Answer
		4 Agriculture<br>5 Business/Entrepreneur<br>6 Govt Job<br>7 Pvt Job<br>8 Daily Wage<br>99 Others (specify)
f_7 *(required)*	How many years of schooling has the husband completed?<br>पति ने कितने साल की स्कूली शिक्षा पूरी की है?<br>*Response constrained to: .>= 0 and .<=25*	
f_8 *(required)*	Occupation of the husband<br>पति का व्यवसाय	1 Student<br>2 Unemployed<br>3 Homemaker<br>4 Agriculture<br>5 Business/Entrepreneur<br>6 Govt Job<br>7 Pvt Job<br>8 Daily Wage<br>99 Others (specify)
f_9 *(required)*	Does the woman consume the following food?<br>क्या महिला निम्नलिखित भोजन का सेवन करती है?	1 Eggs<br>2 Milk<br>3 Mutton<br>4 Fish<br>5 Chicken<br>6 Vegetable/Vegetarian food<br>9999 Other animal food<br>99 Other specify
f_11_lmp *(required)*	LMP Date<br>एलएमपी तिथि<br>*Response constrained to: . >= today() - (10 * 30.4375) and . <= today()*	
f_13 *(required)*	Data Source<br>डेटा स्रोत	1 Reported<br>2 USG<br>3 MCP card/other records
f_10 *(required)*	Do you have access of the phone through out the day?<br>क्या आप के पास व्यक्तिगत फोन है या दिन भर फ़ोन आप के पास रहता हैं	1 Yes (own phone)<br>3 Yes (Family member phone)<br>2 No
f_10_1 *(required)*	Type of phone<br>*Question relevant when: ${f_10} =1 or ${f_10} =3*	1 Yes (Normal Button Phone)<br>2 Yes (Android)<br>3 Yes (Apple)
f_10_2 *(required)*	Timing of phone access in a day<br>*Fill in the hours*<br>*Question relevant when: ${f_10} =3*<br>*Response constrained to: .>= 0 and .<=24*	
Pregnant Women<br>गर्भवती महिला > f_12_ga		
f_11	Gestational Age in weeks	
f_11_1	Gestational Age in days	
Pregnant Women<br>गर्भवती महिला > GPLA details for pregnant woman<br>गर्भवती महिला के लिए GPLA विवरण		
f_15 *(required)*	How many times she woman become pregnant(G)<br>महिला कितनी बार गर्भवती हुई है(G)<br>*Response constrained to: . >= 0*	
f_16 *(required)*	How many times pregnancy crossed 28 weeks of Gestation(P)<br>कितनी बार गर्भावस्था ने गर्भधारण के 28 सप्ताह को पार किया(P)<br>*Response constrained to: . >= 0 and . <= ${f_15}*	
f_17 *(required)*	Pregnancy loss before 28 weeks of Gestation (A)<br>गर्भधारण के 28 सप्ताह से पहले गर्भपात (ए)<br>*Response constrained to: . >= 0 and . <= ${f_15}*	
f_18 *(required)*	Number of living children (L)<br>जीवित बच्चों की संख्या (L)<br>*Response constrained to: . >= 0 and . <= ${f_15}*	
f_14_2	Type of outcome last pregnancy<br>*Question relevant when: ${f_15} >1*	1 Live Birth<br>2 Still Birth<br>3 Miscarriage

Field	Question	Answer
f_14_1 *(required)*	Date of last delivery/ Outcome ?<br>अंतिम प्रसव की तारीख/परिणाम ?<br>*Question relevant when: ${f_15} >1*<br>*Response constrained to: int(${f_18}) < int(today())*	
f_18_1 *(required)*	Age of the youngest child in months<br>महीनों में सबसे छोटे बच्चे की उम्र<br>*Question relevant when: ${f_18} >=1*	
Pregnant Women<br>गर्भवती महिला > preg_anthro		
f_19	Health Screening and Anthropometric Measurements of the woman<br>महिला की स्वास्थ्य जांच और माप	
f_20 *(required)*	Height 1<br>ऊंचाई 1<br>*Please enter a value between 110.0 – 198.0 cm, NA = No*<br>*Response constrained to: ((${f_20} != 'NA' and regex(${f_20} , '^\d{1,3}\.\d{1}$') and number(${f_20}) >= 110 and number(${f_20}) <= 198) or ${f_20} = 'NA')*	
f_20_1 *(required)*	Height 2<br>ऊंचाई 2<br>*Response constrained to: ((${f_20_1} != 'NA' and regex(${f_20_1} , '^\d{1,3}\.\d{1}$') and number(${f_20_1}) >= 110 and number(${f_20_1}) <= 198) or ${f_20_1} = 'NA')*	
f_21 *(required)*	Weight 1<br>वजन 1<br>*Please enter a value between 25.0 – 120.0 kg, NA = No*<br>*Response constrained to: ((${f_21} != 'NA' and regex(${f_21} , '^\d{1,3}\.\d{1}$') and number(${f_21}) >= 25.0 and number(${f_21}) <= 120) or ${f_21} = 'NA')*	
f_21_1 *(required)*	Weight 2<br>वजन 2<br>*Response constrained to: ((${f_21_1} != 'NA' and regex(${f_21_1} , '^\d{1,3}\.\d{1}$') and number(${f_21_1}) >= 25.0 and number(${f_21_1}) <= 120) or ${f_21_1} = 'NA')*	
f_26 *(required)*	Hemoglobin (Hb) Level<br>हीमोग्लोबिन (Hb) स्तर<br>*Please enter a value between 2.5 – 20.0 , NA = No*<br>*Response constrained to: ((${f_26} != 'NA' and regex(${f_26} , '^\d{1,2}\.\d{1}$') and number(${f_26}) >= 2.5 and number(${f_26}) <= 20.0) or ${f_26} = 'NA')*	
f_30_1_1 *(required)*	Did you receive the MCP Card<br>क्या आपको MCP कार्ड प्राप्त हुआ है	1 Yes<br>2 No
f_30_1_5 *(required)*	Are all sections updated (Check card)<br>क्या सभी फ़ील्ड अपडेट किए गए हैं (चेक कार्ड)<br>*Question relevant when: ${f_30_1_1} =1*	1 Yes<br>2 No
f_30_1_5_remark *(required)*	Please specify the blank fields<br>कृपया रिक्त फ़ील्ड निर्दिष्ट करें<br>*Question relevant when: ${f_30_1_5} =2*	1 Basic Indentications<br>2 TD medicinal supplements (page 6)<br>3 ANC specifc information (page 7/8)
f_30_1_5_remark1 *(required)*	Please specify about the blank field in Basic Indentications<br>कृपया बेसिक पहचान में रिक्त फ़ील्ड के बारे में निर्दिष्ट करें<br>*Question relevant when: selected(${f_30_1_5_remark} ,'1')*	
f_30_1_5_remark2 *(required)*	Please specify about the blank field in ANC specific information<br>कृपया ANC जानकारी में रिक्त फ़ील्ड के बारे में निर्दिष्ट करें<br>*Question relevant when: selected(${f_30_1_5_remark} ,'3')*	
f_30_1_5_remark3 *(required)*	Please specify about the blank field in ANC Profile/ Screening Test<br>कृपया एएनसी प्रोफाइल/स्क्रीनिंग टेस्ट में रिक्त क्षेत्र के बारे में निर्दिष्ट करें<br>*Question relevant when: selected(${f_30_1_5_remark} ,'3')*	
f_30_1_5_remark7 *(required)*	Please specify about the blank field for missing TD Vaccination/Medicine Supplement<br>कृपया अनुपलब्ध टीडी टीकाकरण के लिए रिक्त फ़ील्ड के बारे में निर्दिष्ट करें<br>*Question relevant when: selected(${f_30_1_5_remark} ,'2')*	
f_anc_trimester *(required)*	Is the First trimester's ANC record of this pregnancy available?<br>क्या इस गर्भावस्था का पहला तिमाही का एएनसी रिकॉर्ड उपलब्ध है?	1 Yes<br>2 No
Pregnant Women<br>गर्भवती महिला > Additionally check records for first trimester BMI, adequate gestational weight gain, Hb and thyroid status<br>इसके अतिरिक्त पहली तिमाही बीएमआई, पर्याप्त गर्भावधि वजन, एचबी और थायरॉयड स्थिति के लिए रिकॉर्ड की जांच करें<br>*Group relevant when: ${f_anc_trimester} =1*		
f_20_a *(required)*	Height<br>ऊंचाई<br>*Please enter a value between 110.0 – 198.0 cm, NA = No*	

Field	Question	Answer
	Response constrained to: ((${f_20_a} != 'NA' and regex(${f_20_a} , '^\d{1,3}\.\d{1}$') and number(${f_20_a}) >= 110 and number(${f_20_a}) <= 198) or ${f_20_a} = 'NA')	
f_21_a *(required)*	Weight<br>वजन<br>*Please enter a value between 25.0 – 120.0 kg, NA = No*<br>*Response constrained to: ((${f_21_a} != 'NA' and regex(${f_21_a} , '^\d{1,3}\.\d{1}$') and number(${f_21_a}) >= 25.0 and number(${f_21_a}) <= 120) or ${f_21_a} = 'NA')*	
f_21_a_date *(required)*	Date of the weight measurement<br>वजन माप की तिथि<br>*Response constrained to: (${f_21_a} = 'NA') or (${f_21_a} > 0)*	
f_26_a *(required)*	Hemoglobin (Hb) Level<br>हीमोग्लोबिन (Hb) स्तर<br>*Please enter a value between 2.5 – 20.0 , NA = No*<br>*Response constrained to: ((${f_26_a} != 'NA' and regex(${f_26_a} , '^\d{1,2}\.\d{1}$') and number(${f_26_a}) >= 2.5 and number(${f_26_a}) <= 20) or ${f_26_a} = 'NA')*	
f_28_a *(required)*	Fasting Blood sugar<br>*Please enter a value between 20 – 500.0 , NA = No*<br>*Response constrained to: ((${f_28_a} != 'NA' and regex(${f_28_a} , '^\d{1,3}\.\d{1}$') and number(${f_28_a}) >= 20 and number(${f_28_a}) <= 500) or ${f_28_a} = 'NA')*	
f_28_a_1 *(required)*	RBS(Random Blood Sugar)<br>आरबीएस (रेंडम ब्लड शुगर)<br>*Please enter a value between 20 – 500.0 , NA = No*<br>*Response constrained to: ((${f_28_a_1} != 'NA' and regex(${f_28_a_1} , '^\d{1,3}\.\d{1}$') and number(${f_28_a_1}) >= 20 and number(${f_28_a_1}) <= 500) or ${f_28_a_1} = 'NA')*	
f_28_a_2 *(required)*	OGTT(Oral glucose tolerance test) OGTT<br>*Please enter a value between 20 – 200.0 , NA = No*<br>*Response constrained to: ((${f_28_a_2} != 'NA' and regex(${f_28_a_2} , '^\d{1,3}\.\d{1}$') and number(${f_28_a_2}) >= 20 and number(${f_28_a_2}) <= 200) or ${f_28_a_2} = 'NA')*	
f_28_a_2_1 *(required)*	PP(Postprandial)<br>पीपी (खाने के बाद)<br>*Please enter a value between 20 – 200.0 , NA = No*<br>*Response constrained to: ((${f_28_a_2_1} != 'NA' and regex(${f_28_a_2_1} , '^\d{1,3}\. \d{1}$') and number(${f_28_a_2_1}) >= 20 and number(${f_28_a_2_1}) <= 200) or ${f_28_a_2_1} = 'NA')*	
f_28_a_3 *(required)*	HbA1c (%)<br>एचबीए1सी (%)<br>*Please enter a value between 1 – 12.0 , NA = No*<br>*Response constrained to: ((${f_28_a_3} != 'NA' and regex(${f_28_a_3} , '^\d{1,3}\.\d{1}$') and number(${f_28_a_3}) >= 1 and number(${f_28_a_3}) <= 12) or ${f_28_a_3} = 'NA')*	
f_29_a *(required)*	TSH(Thyroid-stimulating hormone)(µg/dl)<br>टीएसएच (µg/dl)	1 µg/dl<br>2 µIU/mL
f_29_dl	µg/dl<br>*Please enter a value between 0.1 to 20.0, NA = No*<br>*Response constrained to: ((regex(., '^[0-9]+(\.[0-9]+)?$') and number(.) >= 0 and number(.) <= 20) or . = 'NA') and (${f_29_a} = 1 or . = 'NA')*	
f_29_ml	µIU/mL<br>*Please enter a value between 0.1 to 20.0, NA = No*<br>*Response constrained to: ((regex(., '^[0-9]+(\.[0-9]+)?$') and number(.) >= 0 and number(.) <= 20) or . = 'NA') and (${f_29_a} = 2 or . = 'NA')*	
f_29_a_t3 *(required)*	T3(ng/dl)<br>टी3(ng/dl)<br>*Please enter a value between 0 to 300.0, NA = No*<br>*Question relevant when: selected(${e_18} ,1)*<br>*Response constrained to: ((${f_29_a_t3} != 'NA' and regex(${f_29_a_t3} , '^\d{1,3}(\.\d+)?$') and number(${f_29_a_t3}) >= 0 and number(${f_29_a_t3}) <= 300) or ${f_29_a_t3} = 'NA')*	
f_29_a_t4 *(required)*	T4(µg/dl)<br>टी4(µg/dl)<br>*Please enter a value between 0.1 to 20.0, NA = No*<br>*Question relevant when: selected(${e_18} ,1)*<br>*Response constrained to: ((${f_29_a_t4} != 'NA' and regex(${f_29_a_t4} , '^\d{1,1}\.\d{1}$') and number(${f_29_a_t4}) >= 0.1 and number(${f_29_a_t4}) <= 20) or ${f_29_a_t4} = 'NA')*	
f_29_a_t4_other *(required)*	If any other investigation available<br>यदि कोई अन्य जांच उपलब्ध है	
f_29_1_a	Gestational weight gain<br>[f_29_1_a_cal]<br>गर्भावधि में वजन बढ़ना<br>[f_29_1_a_cal]	

Field	Question	Answer
f_30 *(required)*	Did you receive antenatal care during your current pregnancy?<br>क्या आपने अपनी वर्तमान गर्भावस्था के दौरान प्रसवपूर्व देखभाल प्राप्त की थी?	1 Yes<br>2 No
Pregnant Women<br>गर्भवती महिला > anc_group_pre		
f_30_1_2 *(required)*	How many ANC checkups did you have?<br>आपने कितनी बार एएनसी चेकअप करवाये थे?<br>*Question relevant when: selected(${f_30} ,1)*<br>*Response constrained to: .>= 1 and .<=10*	
Pregnant Women<br>गर्भवती महिला > anc_group_pre > ANC visit<br>एएनसी का दौरा (1)<br>*Group relevant when: ${f_30} =1*		(Repeated group)
f_30_1_2_anc *(required)*	Date of ANC<br>एएनसी की तिथि<br>*Question relevant when: selected(${f_30} ,1)*<br>*Response constrained to: . <= today()*	
Pregnant Women<br>गर्भवती महिला > anc_group_pre > ANC visit<br>एएनसी का दौरा (1) > anc_visits_group		
f_30_1_3	Gestational Age (GA) in weeks at which ANC Visit done (fill not applicable if not eligible for the particular ANC)	
f_30_1_3_1	Gestational Age (GA) in days at which ANC Visit done (fill not applicable if not eligible for the particular ANC)	
f_30_1_4 *(required)*	Where did you get your ANC checkup done?<br>आपने अपना एएनसी चेकअप कहां करवाया?	1 PHC<br>2 HWC/Sub centre/ANM<br>3 CHC<br>4 Regional Hospital<br>8 Civil Hospital(CH)<br>5 GMC<br>6 Private Hospital<br>7 TBA/Dai<br>99 Others(specify)
f_30_1_6 *(required)*	Were you examined per abdomen at all visits?<br>क्या आपकी सभी एएनसी चेकअप में पेट की जांच की गई थी?	1 Yes<br>2 No
f_30_1_7_1 *(required)*	Weight<br>वजन<br>*Please enter a value between 25.0 – 120.0 kg, NA = No*<br>*Response constrained to: ((${f_30_1_7_1} != 'NA' and regex(${f_30_1_7_1} , '^\d{1,3}\.\d{1}$') and number(${f_30_1_7_1}) >= 25.0 and number(${f_30_1_7_1}) <= 120) or ${f_30_1_7_1} = 'NA')*	
f_30_1_8 *(required)*	Height<br>ऊंचाई<br>*Please enter a value between 110.0 – 198.0 cm, NA = No*<br>*Response constrained to: ((${f_30_1_8} != 'NA' and regex(${f_30_1_8} , '^\d{1,3}\.\d{1}$') and number(${f_30_1_8}) >= 110 and number(${f_30_1_8}) <= 198) or ${f_30_1_8} = 'NA')*	
f_30_1_9 *(required)*	Systolic(Blood pressure)<br>सिस्टोलिक (रक्तचाप)<br>*Please enter a value between 40 to 200, NA=No*<br>*Response constrained to: (${f_30_1_9} != 'NA' and regex(${f_30_1_9} , '^\d{1,3}$') and number(${f_30_1_9}) >= 40 and number(${f_30_1_9}) <= 200) or ${f_30_1_9} = 'NA'*	
f_30_1_9_1 *(required)*	Diastolic(Blood pressure)<br>डायस्टोलिक (रक्तचाप)<br>*Please enter a value between 40 to 200, NA=No*<br>*Response constrained to: (${f_30_1_9_1} != 'NA' and regex(${f_30_1_9_1} , '^\d{1,3}$') and number(${f_30_1_9_1}) >= 40 and number(${f_30_1_9_1}) <= 200) or ${f_30_1_9_1} = 'NA'*	
f_30_1_10_1 *(required)*	Hemoglobin (Hb) Level<br>हीमोग्लोबिन (Hb) स्तर<br>*Please enter a value between 2.5 – 20.0 , NA = No*<br>*Response constrained to: ((${f_30_1_10_1} != 'NA' and regex(${f_30_1_10_1} , '^\d{1,2}\.\d{1}$') and number(${f_30_1_10_1}) >= 2.5 and number(${f_30_1_10_1}) <= 20) or ${f_30_1_10_1} = 'NA')*	
f_30_1_10_2_4 *(required)*	FBS(Fasting Blood Sugar) FBS<br>*Please enter a value between 20 – 200.0 , NA = No*<br>*Response constrained to: ((${f_30_1_10_2_4} != 'NA' and regex(${f_30_1_10_2_4} , '^\d{1,3}\.\d{1}$') and number(${f_30_1_10_2_4}) >= 20 and number(${f_30_1_10_2_4}) <= 200) or ${f_30_1_10_2_4} = 'NA')*	
f_30_1_10_2_2 *(required)*	RBS(Random Blood Sugar)<br>आरबीएस (रेंडम ब्लड शुगर)<br>*Please enter a value between 20 – 500.0 , NA = No*<br>*Response constrained to: ((${f_30_1_10_2_2} != 'NA' and regex(${f_30_1_10_2_2} , '^\d{1,3}\.\d{1}$') and number(${f_30_1_10_2_2}) >= 20 and number(${f_30_1_10_2_2}) <= 500) or ${f_30_1_10_2_2} = 'NA')*	
f_30_1_10_2_3 *(required)*	OGTT(Oral glucose tolerance test) OGTT<br>*Please enter a value between 20 – 200.0 , NA = No*<br>*Response constrained to: ((${f_30_1_10_2_3} != 'NA' and regex(${f_30_1_10_2_3} , '^\d{1,3}\.\d{1}$') and number(${f_30_1_10_2_3}) >= 20 and number(${f_30_1_10_2_3}) <= 200) or ${f_30_1_10_2_3} = 'NA')*	

<table>
<tr><th>Field</th><th>Question</th><th>Answer</th></tr>
<tr><td>f_30_1_10_2_5 (required)</td><td>PP(Postprandial)
पीपी (खाने के बाद)
Please enter a value between 20 – 200.0 , NA = No
Response constrained to: ((${f_30_1_10_2_5} != 'NA' and regex(${f_30_1_10_2_5} , '^\d{1,3}\.\d{1}$') and number(${f_30_1_10_2_5}) >= 20 and number(${f_30_1_10_2_5}) <= 200) or ${f_30_1_10_2_5} = 'NA')</td><td></td></tr>
<tr><td>f_30_1_10_2_5_1 (required)</td><td>HbA1c (%)
एचबीए1सी (%)
Please enter a value between 1 – 12.0 , NA = No
Response constrained to: ((${f_30_1_10_2_5_1} != 'NA' and regex(${f_30_1_10_2_5_1} , '^\d{1,3}\.\d{1}$') and number(${f_30_1_10_2_5_1}) >= 1 and number(${f_30_1_10_2_5_1}) <= 12) or ${f_30_1_10_2_5_1} = 'NA')</td><td></td></tr>
<tr><td>f_30_1_10_3 (required)</td><td>TSH(Thyroid-stimulating hormone)(µg/dl)
टीएसएच (µg/dl)
Please enter a value between 0.1 to 20.0, NA = No</td><td>1 µg/dl
2 µIU/mL</td></tr>
<tr><td>f_30_dl</td><td>µg/dl
Please enter a value between 0.1 to 20.0, NA = No
Response constrained to: ((regex(., '^[0-9]+(\.[0-9]+)?$') and number(.) >= 0 and number(.) <= 20) or . = 'NA') and (${f_30_1_10_3} = 1 or . = 'NA')</td><td></td></tr>
<tr><td>f_30_ml</td><td>µIU/mL
Please enter a value between 0.1 to 20.0, NA = No
Response constrained to: ((regex(., '^[0-9]+(\.[0-9]+)?$') and number(.) >= 0 and number(.) <= 20) or . = 'NA') and (${f_30_1_10_3} = 2 or . = 'NA')</td><td></td></tr>
<tr><td>f_30_1_10_6 (required)</td><td>T3(ng/dl)
टी3(ng/dl)
Please enter a value between 0. to 300.0, NA = No
Response constrained to: ((${f_30_1_10_6} != 'NA' and regex(${f_30_1_10_6} , '^\d{1,3}(\.\d+)?$') and number(${f_30_1_10_6}) >= 0 and number(${f_30_1_10_6}) <= 300) or ${f_30_1_10_6} = 'NA')</td><td></td></tr>
<tr><td>f_30_1_10_7 (required)</td><td>T4(µg/dl)
टी4(µg/dl)
Please enter a value between 0.1 to 20.0, NA = No
Response constrained to: ((${f_30_1_10_7} != 'NA' and regex(${f_30_1_10_7} , '^\d{1,2}\.\d{1}$') and number(${f_30_1_10_7}) >= 0.1 and number(${f_30_1_10_7}) <= 20) or ${f_30_1_10_7} = 'NA')</td><td></td></tr>
<tr><td>f_30_1_10_4 (required)</td><td>VDRL
वीडीआरएल</td><td>1 Positive
3 Negative
2 Not Recorded</td></tr>
<tr><td>f_30_1_10_5 (required)</td><td>HIV</td><td>1 Positive
3 Negative
2 Not Recorded</td></tr>
<tr><td>f_30_1_10_6_1 (required)</td><td>Hep B
हेपेटाइटिस बी</td><td>1 Positive
3 Negative
2 Not Recorded</td></tr>
<tr><td>f_30_1_10_7_1 (required)</td><td>Blood group</td><td>1 Positive
3 Negative
2 Not Recorded</td></tr>
<tr><td>f_30_1_10_9 (required)</td><td>Urine Albumin test</td><td>1 Yes
2 No</td></tr>
<tr><td>f_30_1_10_10 (required)</td><td>Screening/counselling for following
निम्नलिखित के लिए स्क्रीनिंग/परामर्श
पेशाब वाली जगह से दुर्गंध, बदबूदार सफेद पानी आना, पेट के निचले हिस्से में दर्द, पेशाब करते समय जलन होना,गुप्तांग पर घाव या छाले</td><td>1 USG
2 Screened for Reproductive Tract Infections (RTIs)/Sexually transmitted infection (STIs)
4 Screened for depressive symptoms
5 Mental health counselling
6 Nutrition Counselling
7 Counselling on WASH
8 Treated for existing disease
99 Other(Specify)
9999 None</td></tr>
<tr><td>f_30_1_10_12 (required)</td><td>Please specify blood group
Response constrained to: regex(., '^(A|B|AB|O)[+-]$|NA')</td><td></td></tr>
<tr><td>f_34 (required)</td><td>Did the health worker at your ANC (Antenatal Care) visit identify your pregnancy as high-risk?
क्या आपके एएनसी (प्रसवपूर्व देखभाल) दौरे पर स्वास्थ्य कार्यकर्ता ने आपकी गर्भावस्था को उच्च जोखिम के रूप में पहचाना?
Question relevant when: ${f_30} =1</td><td>1 Yes
2 No</td></tr>
</table>

Field	Question	Answer
f_34_1 *(required)*	What was the reason for classifying your pregnancy as high-risk? (Please indicate all that apply and provide details on the gestational age when diagnosed, management, and follow-up. Note from documents available) आपकी गर्भावस्था को उच्च जोखिम के रूप में वर्गीकृत करने का कारण क्या था? (कृपया उन सभी को इंगित करें जो लागू होते हैं । उपलब्ध दस्तावेजों से नोट करें)<br>*Question relevant when: selected(${f_34} ,1)*<br>*Response constrained to: not(selected(${f_34_1} , '9999') and count-selected(${f_34_1}) > 1) or not(selected(${f_34_1} , '9999'))*	1 Severe anemia (<7 gm%)<br>2 PIH/High blood pressure<br>3 GDM/Diabetes<br>4 Syphilis/HIV positive<br>5 Hypothyroidism<br>6 Young primigravida (<20 years) or elderly gravida (>35 years)<br>7 Twin/multiple pregnancies<br>8 Malpresentation<br>9 Previous LSCS (Lower Segment Caesarean Section)<br>10 Low lying placenta/placenta previa<br>11 BOH (Bad Obstetric History: History of stillbirth/abortion/congenital malformation/obstructed labor/preterm delivery)<br>12 Rh negative<br>13 Convulsions<br>14 Oedema<br>15 Bleeding<br>99 Others(specify)
Pregnant Women<br>गर्भवती महिला > [f_34_1_1_1] (1)		(Repeated group)
Pregnant Women<br>गर्भवती महिला > [f_34_1_1_1] (1) > HRP<br>एचआरपी<br>*Group relevant when: selected(${f_34} ,1)*		
f_35 *(required)*	Gestational age when diagnosed with [f_34_1_1_1] (Weeks)<br>गर्भकालीन आयु जब [f_34_1_1_1] (सप्ताह) का निदान किया गया<br>*Response constrained to: .>= 0 and .<=42*	
f_35_1 *(required)*	Gestational age when diagnosed with [f_34_1_1_1] (Days)<br>गर्भकालीन आयु जब [f_34_1_1_1] (दिन) का निदान किया गया<br>*Response constrained to: .>= 0 and .<=6*	
f_36 *(required)*	Management for [f_34_1_1_1][f_34_1_1_1] के लिए प्रबंधन	1 SC/HWC<br>2 PHC<br>3 CHC<br>4 Regional Hospital<br>8 Civil Hospital(CH)<br>5 Private provider clinic(Without in-patient bed)<br>6 Private hospital(With in-patient bed)<br>99 Others(specify)<br>9 Do not use any health facility
f_36_1_1 *(required)*	Were you refered<br>क्या आपको रेफर किया गया था	1 Yes<br>2 No
f_37 *(required)*	Called for follow up visits or followed up at home?<br>फॉलोअप के लिए बुलाया गया या घर पर विजिट किया गया?<br>*Response constrained to: not(selected(${f_37} , '2') and count-selected(${f_37}) > 1) or not(selected(${f_37} , '2'))*	1 Follow up visits<br>3 Home visit<br>2 No
f_38_reason *(required)*	Reason for referral<br>*Question relevant when: ${f_36_1_1} =1*	
f_d_p_19 *(required)*	Do you get any food from the AWC for yourself?<br>क्या आप अपने लिए आंगनवाड़ी केंद्र से कोई भोजन/राशन प्राप्त करते हैं?	1 Yes<br>2 No
f_d_p_19_reason *(required)*	Please specify reason<br>*Question relevant when: ${f_d_p_19} =2*	
Pregnant Women<br>गर्भवती महिला > Supplementary Nutrition<br>*Group relevant when: selected(${f_d_p_19} ,1)*		
Pregnant Women<br>गर्भवती महिला > Supplementary Nutrition > food_practices2_1_1_awc		
j_1_3_1_awc *(required)*	Type of Food Received<br>प्रदान किए गए भोजन का प्रकार	1 Hot Cooked Meal<br>2 THR
Pregnant Women<br>गर्भवती महिला > Supplementary Nutrition > food_practices2_1_1_awc > hcm_1_awc		

Field	Question	Answer
Group relevant when: selected(${j_1_3_1_awc} , '1')		
hcm_1_note_awc	Hot Cooked Meal<br>गर्म पका हुआ भोजन	
hot_cooke_name *(required)*	Specify Hot cooked meal name	
j_1_13_1_awc *(required)*	Quantity consumed per day(in gram)<br>प्रति दिन खायी गयी मात्रा (ग्राम में)<br>*Response constrained to: .>= 0 and .<=200*	
j_1_14_1_awc *(required)*	Variation in Quantity/Type Based on Beneficiary Condition<br>लाभार्थी की स्थिति के आधार पर मात्रा/प्रकार में भिन्नता<br>*If yes, specify*	1 Yes<br>2 No
Pregnant Women<br>गर्भवती महिला > Supplementary Nutrition > food_practices2_1_1_awc > thr_1_awc<br>*Group relevant when: selected(${j_1_3_1_awc} , '2')*		
thr_text1_awc *(required)*	THR (Take home ration) THR	1 Channa<br>2 Dalia<br>3 Jaggary<br>4 Oil<br>5 Panjiri<br>6 Rajma<br>7 Rice<br>8 Salt<br>9 Sevia<br>10 Soya<br>11 Salt Biscuit<br>12 Sweet Biscuit<br>13 WMP(Milk)<br>14 Milk<br>15 Egg<br>26 Black Chana<br>27 Chane Ki Daal<br>16 Wings1<br>17 Wings2<br>18 Wings3<br>19 Wings4<br>20 Wings5<br>21 Wings6<br>22 Wings7<br>23 Wings8<br>24 Wings9<br>25 Wings10<br>99 Other
Pregnant Women<br>गर्भवती महिला > Supplementary Nutrition > food_practices2_1_1_awc > thr_1_awc > THR [thr_cal_repeat] (1)		(Repeated group)
hot_total_awc *(required)*	Total Quantity receive in a month(in gram)<br>*Response constrained to: .>= 0*	
week_awc	Receive for how many days in a week<br>*Response constrained to: .>= 0 and .<=7*	
quantity_p3	Quantity receive per day(in gram)<br>प्रति दिन कितना मिला (ग्राम में)<br>*Response constrained to: .>= 0 and .<=300*	
j_1_17_1_awc *(required)*	Quantity Consumed in gram (Per Day)<br>प्रति दिन खायी गयी मात्रा (ग्राम में)<br>*Response constrained to: .>= 0 and .<=200*	
j_1_18_1_awc *(required)*	Variation in Quantity/Type Based on Beneficiary Condition<br>लाभार्थी की स्थिति के आधार पर मात्रा/प्रकार में भिन्नता<br>*If yes, specify*	1 Yes<br>2 No
f_1_32_1 *(required)*	Do you face any challenges in receiving food from the AWC?<br>क्या आपको आंगनवाड़ी केंद्र से भोजन/राशन प्राप्त करने में किसी चुनौती का सामना करना पड़ता है?<br>*Question relevant when: selected(${f_d_p_19} ,1)*	1 Yes<br>2 No
f_1_33 *(required)*	What were the challenges?<br>चुनौतियां क्या थीं?<br>*Question relevant when: selected(${f_1_32_1} ,1)*	1 Food not available<br>2 Difficult to get it every day<br>3 Milk/egg not given<br>4 Quantity less than what was told to<br>5 Poor quality food

Field	Question	Answer
		99 Others(specify)
f_1_34 *(required)*	Do you face any challenges in consuming of AWC food?<br>*Question relevant when: selected(${f_d_p_19} ,1)*	1 Yes<br>2 No
f_1_34_challenges *(required)*	What were the challenges?<br>चुनौतियां क्या थीं?<br>*Question relevant when: ${f_1_34} =1*	
Pregnant Women<br>गर्भवती महिला > supplements_consumed1		
f_31 *(required)*	Have you consumed or are you presently consuming any of the following during your pregnancy period? If yes, please furnish details regarding the duration of consumption, quantity.<br>क्या आपने गर्भावस्था की अवधि के दौरान निम्नलिखित में से किसी का सेवन किया है या कर रहे हैं? यदि हाँ, तो कृपया उपभोग की अवधि, मात्रा के बारे में विवरण प्रस्तुत करें।<br>*In the last 6 months*<br>*Response constrained to: not(selected(., '9999') and count-selected(.) > 1)*	1 Iron and Folic Acid (IFA) tablets<br>8 Iron and Folic Acid (IFA) syrup<br>7 Folic Acid<br>2 Combination of Calcium & Vitamin-D<br>3 Calcium Tablet<br>4 Vitamin-D<br>5 Multiple Micronutrient Supplements (MMS)<br>6 Multi Vitamin<br>99 Others(medicinal suppliment)<br>9999 None
Pregnant Women<br>गर्भवती महिला > supplements_consumed1 > f_med_status_g<br>*Group relevant when: not(selected(${f_31} , '9999'))*		
Pregnant Women<br>गर्भवती महिला > supplements_consumed1 > f_med_status_g > [med_names] (1)		(Repeated group)
med_status *(required)*	When did you consume [med_names]?<br>आपने [med_names] की गोली कब ली है?	1 Consumed in the past पहले ली थी<br>2 Currently consuming अभी ले रही
f_33_4_4 *(required)*	Since you started, for how many total days have you consumed [med_names]?<br>(Approximate number of days)<br>[med_names] या गोली लेना शुरू करने के बाद से अब तक आपने कुल कितने दिनों तक गोली ली है?<br>(कृपया अनुमानित दिनों की संख्या बताएं)<br>*Response constrained to: .>= 1 and .<=180*	
f_33_4_5 *(required)*	How many days in a week do/did you consume [med_names]?<br>(Enter number of days: 0 to 7)<br>आप एक सप्ताह में कितने दिन [med_names] या गोली लेती हैं/थीं?<br>(दिनों की संख्या दर्ज करें: 0 से 7)<br>*Response constrained to: .>= 1 and .<=7*	
f_33_4_1 *(required)*	How many [med_names] tablets do/did you consume in a day?<br>आप एक दिन में कितनी [med_names] या गोलियाँ लेती हैं/थीं?<br>*Response constrained to: .>= 1 and .<=3*	
f_33_4_2 *(required)*	What is your [med_names] consumption pattern?	1 Regularly<br>2 Irregularly
f_text *(required)*	If you consume or consumed [med_names] irregularly, what are the reasons for missing the tablets?<br>अगर आप अनियमित रूप से [med_names] लेती हैं/थीं, तो Syrup या गोली न लेने के क्या कारण हैं	
source_of_supp1 *(required)*	[med_names_a] Received from	1 Received from Goverment<br>2 Purchase from Private
albenda *(required)*	Do you consume albendazole tablet	1 Yes<br>2 No
date_albanda *(required)*	When was the last time albendazole tablet was consumed?<br>आखिरी बार एल्बेंडाजोल टैबलेट का सेवन कब किया गया था?<br>*Question relevant when: ${albenda} =1*	
f_37_1_d2 *(required)*	In the last one month, have you been diagnosed or suffered from any of the following condition/Illness?<br>पिछले एक महीने में, क्या आपका निम्नलिखित में से किसी भी स्थिति / बीमारी का निदान किया गया है या आप इनसे ग्रासित हुई हैं?<br>*पेशाब वाली जगह से दुर्गंध, बदबूदार सफेद पानी आना, पेट के निचले हिस्से में दर्द, पेशाब करते समय जलन होना,गुप्तांग पर घाव या छाले*<br>*Response constrained to: not(selected(${f_37_1_d2} , '9999') and count-selected(${f_37_1_d2}) > 1) or not(selected(${f_37_1_d2} , '9999'))*	1 Hypothyroidism<br>2 Reproductive tract infection(RTI)/Sexually Transmitted Infection(STI)<br>10 UTI(Urinary Tract Infection)<br>3 Gestational Diabetes<br>4 Pre-eclampsia<br>5 Eclampsia<br>6 Hypertension<br>7 Anaemia

Field	Question	Answer
		8 Bleeding (APH)<br>9 Multiple pregnancy<br>99 Other(Specify)<br>9999 No Disease/Illness
f_37_1_d2_source *(required)*	Source document of the problem<br>*Question relevant when: not(selected(${f_37_1_d2} , '9999'))*	1 Reported by Beneficiary<br>2 Verified from document
f_37_1_d2_source1 *(required)*	Type of document<br>*custom-specify-other(other=99)*<br>*Question relevant when: ${f_37_1_d2_source} =2*	1 Prescription<br>2 Lab report<br>3 Government card(MCP)<br>99 Other
f_38_d2 *(required)*	Did you seek treatment outside of home<br>क्या आपने घर के बाहर इलाज कराया<br>*Question relevant when: ${f_37_1_d2} !=9999*	1 Yes<br>2 No
f_39_d2 *(required)*	From whom?<br>किससे?<br>*Question relevant when: ${f_38_d2} =1*	1 HWC<br>2 CHC<br>3 PHC<br>4 Civil Hospital(CH)<br>5 Regional Hospital<br>6 ASHA<br>7 ANM<br>8 Medical college<br>9 Private provider clinic(Without in-patient bed)<br>10 Private hospital(With in-patient bed)<br>11 Faith healers/traditional health care providers<br>99 Others(Specify)
f_40_d2 *(required)*	Do you have any treatment related documents(investigation reports, prescriptions, if admitted discharge summary, tablet strips, syrup bottles) क्या आपके पास उपचार से संबंधित कोई दस्तावेज हैं (जांच रिपोर्ट, नुस्खे, यदि भर्ती कराया गया है तो डिस्चार्ज सारांश, टैबलेट स्ट्रिप्स, सिरप की बोतलें)<br>*Question relevant when: ${f_38_d2} =1*<br>*Response constrained to: not(selected(${f_40_d2} , '3') and count-selected(${f_40_d2}) > 1) or not(selected(${f_40_d2} , '3'))*	1 Documents<br>2 Medicine(Tablets strips/Syrup)<br>3 None of the above
f_40_d2_1 *(required)*	Upload Document<br>दस्तावेज़ अपलोड करें<br>*Question relevant when: selected(${f_40_d2} ,'1')*	
f_40_d2_2 *(required)*	Mention the name of medicine<br>दवा के नाम का उल्लेख करें<br>*Question relevant when: selected(${f_40_d2} ,'2')*	
f_41_d2 *(required)*	Were you admitted to hospital in the last 3 months?<br>क्या आप पिछले 3 महीनों में अस्पताल में भर्ती थे?	1 Yes<br>2 No
f_42_d2 *(required)*	For what condition have you been admited<br>आपको किस स्थिति के लिए भर्ती कराया गया<br>*Question relevant when: ${f_41_d2} =1*	
Pregnant Women<br>गर्भवती महिला > diet_group_preg		
f_d_p_1_pre	What do you usually have in your diet and how many times in a week? आप आमतौर पर अपने आहार में क्या लेते हैं और सप्ताह में कितनी बार?	
f_d_p_1_1 *(required)*	Cereal (rice/wheat)<br>अनाज (चावल/गेहूं)<br>*No of times*<br>*Response constrained to: .>= 0 and .<=21*	
f_d_p_1_2 *(required)*	Legumes / Lantils (beans, peas)<br>फलियां / दाल (बीन्स, मटर)<br>*No of times*<br>*Response constrained to: .>= 0 and .<=21*	
f_d_p_1_3 *(required)*	Green leafy vegetables<br>हरी पत्तेदार सब्जियां<br>*No of times*<br>*Response constrained to: .>= 0 and .<=21*	
f_d_p_1_4 *(required)*	Other vegetables<br>अन्य सब्जियां<br>*No of times*	

Field	Question	Answer
	Response constrained to: .>= 0 and .<=21	
f_d_p_1_5 *(required)*	Fruits (guava, orange, apple, pomegranate)<br>फल (अमरूद, संतरा, सेब, अनार)<br>*No of times*<br>*Response constrained to: .>= 0 and .<=21*	
f_d_p_1_6 *(required)*	Meat<br>मांस<br>*No of times*<br>*Response constrained to: .>= 0 and .<=21*	
f_d_p_1_7 *(required)*	Fish<br>मछली<br>*No of times*<br>*Response constrained to: .>= 0 and .<=21*	
f_d_p_1_8 *(required)*	Eggs<br>अंडे<br>*No of times*<br>*Response constrained to: .>= 0 and .<=21*	
f_d_p_1_9 *(required)*	Milk<br>दूध<br>*No of times*<br>*Response constrained to: .>= 0 and .<=21*	
f_d_p_1_10 *(required)*	Milk products (yogurt, paneer)<br>दूध उत्पाद (दही, पनीर)<br>*No of times*<br>*Response constrained to: .>= 0 and .<=21*	
f_d_p_1_11 *(required)*	Jaggery (gudd)<br>गुड़ (गुड़)<br>*No of times*<br>*Response constrained to: .>= 0 and .<=21*	
f_d_p_1_12 *(required)*	Red meat<br>लाल मांस (मटन)<br>*No of times*<br>*Response constrained to: .>= 0 and .<=21*	
f_d_p_1_13 *(required)*	Millets (ragi, bajra, jawar)<br>बाजरा (रागी, बाजरा, ज्वार)<br>*No of times*<br>*Response constrained to: .>= 0 and .<=21*	
f_d_p_1_14 *(required)*	Soy product<br>सोया उत्पाद<br>*No of times*<br>*Response constrained to: .>= 0 and .<=21*	
f_d_p_1_15 *(required)*	Lemon/amla<br>नींबू/आंवला<br>*No of times*<br>*Response constrained to: .>= 0 and .<=21*	
f_d_p_1_18 *(required)*	Nuts and oil seeds<br>मेवे और तेल के बीज<br>*No of times<br>(Eg: Almond, Walnuts, Peanut, Sesame, Sunflower seeds etc)*<br>*Response constrained to: .>= 0 and .<=42*	
f_d_p_1_19 *(required)*	Fats and oils<br>वसा और तेल<br>*No of times<br>(Butter, Ghee, Mustard oil, Coconut oil etc.)*<br>*Response constrained to: .>= 0 and .<=42*	
f_d_p_1_16 *(required)*	Any fortified rice/wheat<br>कोई भी फोर्टिफाइड चावल/गेहूं<br>*No of times*<br>*Response constrained to: .>= 0 and .<=21*	
f_d_p_1_17 *(required)*	Other fortified foods<br>अन्य फोर्टिफाइड खाद्य पदार्थ<br>*No of times*<br>*Response constrained to: .>= 0 and .<=21*	
Pregnant Women<br>गर्भवती महिला > Pregnant woman healthcare seeking		
f_38 *(required)*	Was the pregnant woman ever referred for any health/nutrition related condition during this pregnacy?<br>क्या गर्भवती महिला को कभी किसी स्वास्थ्य/पोषण संबंधी स्थिति के लिए भेजा गया था (इस गर्भावस्था के दौरान)?	1 Yes<br>2 No
f_38_1_1 *(required)*	Where was the pregnant woman referred?<br>गर्भवती महिला को कहां रेफर किया गया?<br>*Question relevant when: selected(${f_38} ,1)*	1 HWC<br>2 CHC<br>3 PHC<br>4 Civil Hospital(CH)

Field	Question	Answer
		5 Regional Hospital<br>6 ASHA<br>7 ANM<br>8 Medical college<br>9 Private provider clinic(Without in-patient bed)<br>10 Private hospital(With in-patient bed)<br>11 Faith healers/traditional health care providers<br>99 Others(Specify)
f_38_1_1_reason *(required)*	Reason for referral<br>*Question relevant when: ${f_38} =1*	
f_38_1_2 *(required)*	Did the pregnant woman comply with referral<br>क्या गर्भवती महिला ने रेफरल का पालन किया<br>*Question relevant when: selected(${f_38} ,1)*	1 Yes<br>2 No
f_38_1_3 *(required)*	Where did the pregnant woman go?<br>गर्भवती महिला कहां गई?<br>*Question relevant when: selected(${f_38_1_2} ,1)*	1 HWC<br>2 CHC<br>3 PHC<br>4 Civil Hospital(CH)<br>5 Regional Hospital<br>6 ASHA<br>7 ANM<br>8 Medical college<br>9 Private provider clinic(Without in-patient bed)<br>10 Private hospital(With in-patient bed)<br>11 Faith healers/traditional health care providers<br>99 Others(Specify)
f_38_1_4 *(required)*	Did the pregnant woman receive any treatment?<br>क्या गर्भवती महिला को कोई इलाज मिला?<br>*Question relevant when: ${f_38_1_2} =1*	1 Yes<br>2 No
f_38_1_4_1 *(required)*	Condition resolved?<br>क्या समस्या हल हो गई?<br>*Question relevant when: ${f_38_1_4} =1*	1 Yes<br>2 No
f_39 *(required)*	Did the pregnant woman face any challenges to comply with referral or at the referral facility?<br>क्या गर्भवती महिला को रेफरल के दौरन या रेफरल केन्द्र पर सुविधा लेने में किसी भी चुनौती का सामना करना पड़ा?<br>*Question relevant when: ${f_38_1_2} =1*	1 Yes<br>2 No
f_39_1 *(required)*	What challenges did the pregnant woman face?<br>गर्भवती महिला को किन चुनौतियों का सामना करना पड़ा?<br>*Question relevant when: ${f_39} =1*	1 Overcrowded<br>2 Doctors not available<br>3 Long waiting hours<br>4 Medicines not available<br>5 No laboratory facilities<br>6 Long distance<br>7 Lack of transportation<br>8 Timing unpredictable<br>9 Rude behavior of health staff<br>10 Casual attitude of staff<br>99 Others(specify)
f_41 *(required)*	Was the pregnant woman happy/satisfied with the services provided at the ANC clinic, including referral?<br>क्या गर्भवती महिला रेफरल सहित एएनसी क्लिनिक में प्रदान की जाने वाली सेवाओं से खुश/संतुष्ट थी?	1 Yes<br>2 No
preg_remarks	Respondent remarks<br>*Question relevant when: ${d_9} =1*	
preg_interviewer_remark *(required)*	Interviewer remarks<br>*Question relevant when: ${d_9} =1*	
Postnatal Mother(0 - 6)<br>*Group relevant when: ${d_10} =1*		
Postnatal Mother(0 - 6) > intrapartum_group		
f_2_2 *(required)*	ID of the Mother<br>माँ की आईडी	

Field	Question	Answer
	Please verify with the given list of IDs<br>Enter the value between 401 - 499<br>*Response constrained to: regex(., '^(?!400$)[4][0-9]{2}$')*	
f_2_1 *(required)*	Name of the mother/caregiver<br>माता/देखभाल करने वाले का नाम<br>*Response constrained to: not(regex(.,'^(.*)\d(.*)$'))*	
f_2_3 *(required)*	Age of the Mother<br>माँ की उम्र<br>*Response constrained to: .>= 18 and .<=45*	
f_2_3_1 *(required)*	Name of the husband<br>पति का नाम<br>*Response constrained to: not(regex(.,'^(.*)\d(.*)$'))*	
f_2_4 *(required)*	Age of the husband<br>पति की उम्र<br>*Response constrained to: .>= 18 and .<=55*	
f_2_5 *(required)*	How many years of schooling has the mother completed?<br>माँ ने कितने साल की स्कूली शिक्षा पूरी की है?<br>*Response constrained to: .>= 0 and .<=25*	
f_2_6 *(required)*	Occupation of the Mother<br>माँ का व्यवसाय	1 Student<br>2 Unemployed<br>3 Homemaker<br>4 Agriculture<br>5 Business/Entrepreneur<br>6 Govt Job<br>7 Pvt Job<br>8 Daily Wage<br>99 Others (specify)
f_2_7 *(required)*	How many years of schooling has the husband completed?<br>पति ने कितने साल की स्कूली शिक्षा पूरी की है?<br>*Response constrained to: .>= 0 and .<=25*	
f_2_8 *(required)*	Occupation of Husband<br>पति का व्यवसाय	1 Student<br>2 Unemployed<br>3 Homemaker<br>4 Agriculture<br>5 Business/Entrepreneur<br>6 Govt Job<br>7 Pvt Job<br>8 Daily Wage<br>99 Others (specify)
f_2_9 *(required)*	Does the mother consume the following food<br>क्या माँ निम्नलिखित भोजन का सेवन करती है	1 Eggs<br>2 Milk<br>3 Mutton<br>4 Fish<br>5 Chicken<br>6 Vegetable/Vegetarian food<br>9999 Other animal food<br>99 Other specify
f_2_10 *(required)*	Do you have access of the phone through out the day?<br>क्या आप के पास व्यक्तिगत फोन है या दिन भर फ़ोन आप के पास रहता हैं	1 Yes (own phone)<br>3 Yes (Family member phone)<br>2 No
f_2_10a *(required)*	Type of phone<br>*Question relevant when: ${f_2_10} =1 or ${f_2_10} =3*	1 Yes (Normal Button Phone)<br>2 Yes (Android)<br>3 Yes (Apple)
f_2_10b *(required)*	Timing of phone access in a day<br>*Fill in the hours*<br>*Question relevant when: ${f_2_10} =3*<br>*Response constrained to: .>= 0 and .<=24*	
f_2_26 *(required)*	Hemoglobin (Hb) Level<br>हीमोग्लोबिन (Hb) स्तर<br>*Please enter a value between 2.5 – 20.0 , NA = No<br>Measured by study team*<br>*Response constrained to: ((${f_2_26} != 'NA' and regex(${f_2_26} , '^\d{1,2}\.\d{1}$') and number(${f_2_26}) >= 2.5 and number(${f_2_26}) <= 20.0) or ${f_2_26} = 'NA')*	
Postnatal Mother(0 - 6) > Delivery Details डिलिवरी विवरण		

Field	Question	Answer
f_2_27 *(required)*	Date of delivery<br>डिलीवरी की तारीख<br>*Response constrained to: . >= today() - (6 * 30.4375) and . <= today()*	
f_2_28 *(required)*	Where did you deliver the baby?<br>आपने बच्चे को कहां जन्म दिया?	1 Home<br>2 Institution (Government- type of facility)<br>3 Institution (Private- type of facility)<br>4 Delivery during transit
f_2_28_govt *(required)*	Please specify name and type of goverment institution<br>कृपया सरकारी संस्थान का नाम और प्रकार निर्दिष्ट करें<br>*Question relevant when: ${f_2_28} =2*	
f_2_28_pvt *(required)*	Please specify name and type of private institution<br>कृपया निजी संस्थान का नाम और प्रकार निर्दिष्ट करें<br>*Question relevant when: ${f_2_28} =3*	
f_2_28_trn *(required)*	Please specify (e.g Ambulance)<br>कृपया निर्दिष्ट करें (जैसे एम्बुलेंस)<br>*Question relevant when: ${f_2_28} =4*	
f_2_29 *(required)*	Who conducted the delivery?<br>डिलीवरी किसने करवाइ?	1 Doctor<br>2 Nurse<br>3 Midwife<br>4 Dai<br>5 Relatives<br>6 ASHA<br>99 Other
f_2_30_inc *(required)*	Did you receive any incentives for Institutional Delivery?<br>क्या आपको संस्थागत प्रसव के लिए कोई राशी मिली?<br>*Question relevant when: selected(${f_2_28} ,2) or selected(${f_2_28} ,3)*	1 Yes Received<br>3 Yes applied for<br>2 No
f_2_32 *(required)*	How long after birth did you initiate breastfeeding in hours?<br>जन्म के कितने समय बाद आपने स्तनपान शुरू किया?<br>*If half an hr enter .3 If 1 hr, enter 1.*	
f_2_31 *(required)*	If home delivery, reasons?<br>अगर होम डिलीवरी हो, तो कारण?<br>*Question relevant when: selected(${f_2_28} ,1)*	1 Lack of knowledge<br>2 Feel safe to deliver at home<br>3 Hospital & Transport cost<br>4 Distance to health facilities<br>5 Poor qulaity of care in health facilties<br>99 Others(specify)
f_2_33 *(required)*	Which type of delivery did you have?<br>डिलीवरी का प्रकार<br>*Question relevant when: selected(${f_2_28} ,2) or selected(${f_2_28} ,3)*	1 Normal Vaginal delivery<br>2 Cesarean section (C-section)<br>3 Instrumental delivery (forceps or vacuum extraction)
f_2_34 *(required)*	How many babies did you give birth?<br>आपने कितने बच्चों को जन्म दिया?	1 Singleton<br>2 Twins<br>3 Triplets
f_2_35_g *(required)*	Gender of the baby<br>बच्चे का लिंग	1 Male<br>2 Female
f_2_35_g_two *(required)*	Gender of the baby 2<br>बच्चे का लिंग<br>*Question relevant when: ${f_2_34} =2*	1 Male<br>2 Female
f_2_35_g_out	Birth Outcome<br>*Question relevant when: ${f_2_34} =2*	1 Live birth<br>2 Stillbirth
f_2_35_status *(required)*	Current Vital status of the baby<br>बच्चे की वर्तमान स्थिति	1 Live<br>2 Dead
child_dob_pn *(required)*	Date of Birth of the child<br>बच्चे की जन्म तिथि<br>*Response constrained to: . >= today() - (24 * 30.4375) and . <= today()*	
Postnatal Mother(0 - 6) > Delivery Details डिलिवरी विवरण > pn_delivery1		
f_kmc *(required)*	Weight of child at the time birth<br>जन्म के समय बच्चे का वजन<br>*Please enter a value between 1.00 – 10.00 kg, NA = No*	

Field	Question	Answer
	Response constrained to: ((${f_kmc} != 'NA' and regex(${f_kmc} , '^\d{1,2}\.\d{2}$') and number(${f_kmc}) >= 1 and number(${f_kmc}) <= 10) or ${f_kmc} = 'NA')	
birth_source_doc *(required)*	Source documents birth weight	1 Reported by Beneficiary<br>2 Verified from document
f_2_37 *(required)*	New born gestational age at the time of birth (in Weeks)<br>जन्म के समय नवजात की गर्भकालीन आयु (सप्ताह में)<br>*Response constrained to: .>= 0 and .<=42*	
f_2_37_1 *(required)*	New born gestational age at the time of birth (in Days)<br>जन्म के समय नवजात की गर्भकालीन आयु (दिनों में)<br>*Response constrained to: .>= 0 and .<=6*	
f_2_55 *(required)*	Were you provided with the following postnatal services? (Just after delivery)<br>क्या आपको निम्नलिखित प्रसव के बाद की सेवाएं प्रदान की गई थीं?	1 Assessment of P/V bleeding<br>2 Examination of breasts & nipples<br>3 Assessment of breastfeeding<br>4 Initiation of Breastfeeding within 1 Hour of Birth<br>5 Counselling on exclusive breastfeeding (Birth to 6 months)<br>6 Cord care<br>7 Keeping baby warm, delayed bathing<br>8 For LBW/preterm, KMC<br>10 For LBW/preterm, feeding of expressed breastmilk<br>11 Information on identification of danger signs in newborns and immediate care seeking<br>9999 None
Postnatal Mother(0 - 6) > Delivery Details डिलिवरी विवरण > [f_2_55_cal] (1)<br>*Group relevant when: ${f_2_55} !=9999*		(Repeated group)
f_2_55_1 *(required)*	Timings of postnatal services(in hours after birth)<br>प्रसवोत्तर सेवाओं का समय (जन्म के बाद घंटों में)	
f_2_56 *(required)*	Who provided post-natal services before discharge from the facility<br>*Question relevant when: not(selected(${f_2_55} , '9999'))*	1 ASHA<br>2 ANM<br>3 AWW<br>4 Health Supervisor<br>5 MO<br>6 ICDS Supervisor<br>7 Staff Nurse<br>99 Others (specify)
Postnatal Mother(0 - 6) > post_deli_group		
f_2_50 *(required)*	Lactation Support Received, What type of lactation support did you recived स्तनपान में सहायता मिली,आपको स्तनपान में किस प्रकार की सहायता मिली	1 Counselling and demonstration on appropriate positioning during feeding<br>2 Counselling and demonstration on appropriate attachment<br>3 Counselling and demonstration on appropriate sucking<br>4 Technique of expressing breast milk<br>5 Feeding of (Expressed Breast Milk)<br>6 Management of complications (engorged breasts, inverted nipple,

Field	Question	Answer
		cracked nipples, lack of milk flow et)<br>9999 None
f_2_38_vlbw	Is the baby very low birth weight at time of birth?	
f_2_38_lbw	Was the baby low birth weight at time of birth?	
f_2_38_pre	Was the baby preterm?	
f_2_39 *(required)*	Were you provided post-natal check-ups in the facility or at home?<br>क्या आपको स्वास्थ्य केन्द्र पर या घर पर प्रसवोत्तर चेक-अप की सुविधा प्रदान की गई थी?<br>*Question relevant when: selected(${f_2_28} ,2) or selected(${f_2_28} ,3)*	1 Facility<br>2 Home<br>3 Both<br>9999 None
f_2_41 *(required)*	How long after birth did you initiate breastfeeding in hours?<br>जन्म के कितने समय बाद आपने स्तनपान शुरू किया? (घंटों में)	
g_31 *(required)*	If baby is less than 6 months old, is the baby exclusively breastfed?	1 Yes<br>2 No
f_2_42 *(required)*	Are you exclusive breastfeeding?<br>*Feeding of mothers milk either directly breast fed or expressed breast milk feeding.Not giving anything other then mothers milk*	1 Yes<br>2 No
Postnatal Mother(0 - 6) > pn_delivery2		
f_2_43 *(required)*	Did you give Kangaroo Mother Care(KMC)?<br>क्या आपने कंगारू मदर केयर (KMC) दिया?<br>*Question relevant when: ${f_2_38_lbw} =1 or ${f_2_38_pre} =1*	1 Yes<br>2 No
f_2_44 *(required)*	When was KMC initiated after birth (in hours)<br>जन्म के बाद केएमसी कब शुरू किया गया था (घंटों में)<br>*Question relevant when: ${f_2_43} =1*	
f_2_44_1 *(required)*	How many days KMC was given<br>केएमसी कितने दिन दिया गया था<br>*Question relevant when: ${f_2_43} =1*	
f_2_45 *(required)*	Average duration of Skin to skin contact (SSC)per day(In minutes)<br>त्वचा से त्वचा संपर्क (एसएससी) की औसत अवधि प्रति दिन (मिनटों में)<br>*Question relevant when: ${f_2_43} =1*<br>*Response constrained to: .>= 0 and .<=1440*	
f_2_46 *(required)*	At the time of discharge, was discharge card and advice given?<br>डिस्चार्ज के समय डिस्चार्ज कार्ड और सलाह दी गई थी?<br>*Question relevant when: selected(${f_2_28} ,2) or selected(${f_2_28} ,3)*	1 Yes<br>2 No
f_2_47_1 *(required)*	Did any worker visit your home after birth to provide HBNC?<br>क्या कोई कार्यकर्ता जन्म के बाद एचबीएनसी प्रदान करने के लिए आपके घर आया था<br>*Question relevant when: ${f_2_43} =1*	1 ASHA<br>2 ANM<br>3 AWW<br>4 Health Supervisor<br>5 MO<br>6 ICDS Supervisor<br>7 Staff Nurse<br>99 Others (specify)
f_2_48 *(required)*	Did you receive Home based neonatal care (HBNC)?<br>क्या आपने एचबीएनसी प्राप्त की?	1 Yes Reported<br>3 Yes Documented<br>2 No
f_2_49 *(required)*	Who visited your home during most of the HBNC visits<br>एचबीएनसी की अधिकांश विजिट के दौरान आपके घर कौन आया<br>*Question relevant when: ${f_2_48} =1 or ${f_2_48} =3*	1 ASHA<br>2 ANM<br>3 AWW<br>4 Health Supervisor<br>5 MO<br>6 ICDS Supervisor<br>7 Staff Nurse<br>99 Others (specify)
f_2_48_1 *(required)*	Which are the days when you receive HBNC?<br>वे कौन से दिन हैं जब आप एचबीएनसी प्राप्त करते हैं?<br>*Question relevant when: ${f_2_48} =1 or ${f_2_48} =3*	1 Day 1<br>2 Day 3<br>3 Day 7<br>4 Day 14<br>5 Day 21<br>6 Day 28<br>7 Day 42<br>99 Other specify

Field	Question	Answer
f_2_d_p_19 *(required)*	Do you get any food from the AWC for yourself?<br>क्या आप अपने लिए आंगनवाड़ी केंद्र से कोई भोजन/राशन प्राप्त करते हैं?	1 Yes<br>2 No
Postnatal Mother(0 - 6) > Supplementary Nutrition<br>*Group relevant when: ${f_2_d_p_19} =1*		
Postnatal Mother(0 - 6) > Supplementary Nutrition > food_practices2_1_1_awc_b		
j_1_3_1_awc_b *(required)*	Type of Food Received<br>प्रदान किए गए भोजन का प्रकार	1 Hot Cooked Meal<br>2 THR
Postnatal Mother(0 - 6) > Supplementary Nutrition > food_practices2_1_1_awc_b > hcm_1_awc_b<br>*Group relevant when: selected(${j_1_3_1_awc_b} , '1')*		
hcm_1_note_awc_b	Hot Cooked Meal<br>गर्म पका हुआ भोजन	
hot_cooke_name_b *(required)*	Specify Hot cooked meal name	
j_1_13_1_awc_b *(required)*	Quantity consumed per day(in gram)<br>प्रति दिन खायी गयी मात्रा (ग्राम में)<br>*Response constrained to: .>= 0 and .<=200*	
j_1_14_1_awc_b *(required)*	Variation in Quantity/Type Based on Beneficiary Condition<br>लाभार्थी की स्थिति के आधार पर मात्रा/प्रकार में भिन्नता<br>*If yes, specify*	1 Yes<br>2 No
Postnatal Mother(0 - 6) > Supplementary Nutrition > food_practices2_1_1_awc_b > thr_1_awc_b<br>*Group relevant when: selected(${j_1_3_1_awc_b} , '2')*		
thr_text1_awc_b *(required)*	THR (Take home ration) THR	1 Channa<br>2 Dalia<br>3 Jaggary<br>4 Oil<br>5 Panjiri<br>6 Rajma<br>7 Rice<br>8 Salt<br>9 Sevia<br>10 Soya<br>11 Salt Biscuit<br>12 Sweet Biscuit<br>13 WMP(Milk)<br>14 Milk<br>15 Egg<br>26 Black Chana<br>27 Chane Ki Daal<br>16 Wings1<br>17 Wings2<br>18 Wings3<br>19 Wings4<br>20 Wings5<br>21 Wings6<br>22 Wings7<br>23 Wings8<br>24 Wings9<br>25 Wings10<br>99 Other
Postnatal Mother(0 - 6) > Supplementary Nutrition > food_practices2_1_1_awc_b > thr_1_awc_b > THR [thr_cal_repeat_b] (1)		(Repeated group)
hot_total_awc_b *(required)*	Total Quantity receive in a month(in gram)<br>*Response constrained to: .>= 0*	
week_awc_b *(required)*	Receive for how many days in a week<br>*Response constrained to: .>= 0 and .<=7*	
quantity_p3_b *(required)*	Quantity receive per day(in gram)<br>प्रति दिन कितना मिला (ग्राम में)<br>*Response constrained to: .>= 0 and .<=200*	
j_1_12_1_awc_b *(required)*	Quantity consumed per day(in gram)<br>प्रति दिन खायी गयी मात्रा (ग्राम में)<br>*Response constrained to: .>= 0 and .<=200*	
j_1_18_1_awc_b *(required)*	Variation in Quantity/Type Based on Beneficiary Condition<br>लाभार्थी की स्थिति के आधार पर मात्रा/प्रकार में भिन्नता<br>*If yes, specify*	1 Yes<br>2 No

Field	Question	Answer
f_2_1_32_1 *(required)*	Do you face any challenges in receiving food from the AWC?<br>क्या आपको आंगनवाड़ी केंद्र से भोजन/राशन प्राप्त करने में किसी चुनौती का सामना करना पड़ता है?<br>*Question relevant when: ${f_2_d_p_19} =1*	1 Yes<br>2 No
f_2_1_33 *(required)*	What were the challenges?<br>चुनौतियां क्या थीं?<br>*Question relevant when: selected(${f_2_1_32_1} ,1)*	1 Food not available<br>2 Difficult to get it every day<br>3 Milk/egg not given<br>4 Quantity less than what was told to<br>5 Poor quality food<br>99 Others(specify)
f_2_34_a *(required)*	Do you face any challenges in consuming of AWC food?<br>*Question relevant when: ${f_2_d_p_19} =1*	1 Yes<br>2 No
f_2_34_challenges *(required)*	What were the challenges?<br>चुनौतियां क्या थीं?<br>*Question relevant when: ${f_2_34_a} =1*	
Postnatal Mother(0 - 6) > awc_counselling		
generated_table_list_label_657	Counselling and AWC<br>परामर्श और एडब्ल्यूसी	
f_2_51	Did you receive any counselling on<br>क्या आपको कोई परामर्श मिला	
reserved_name_for_field_list_labels_659		1 ASHA<br>2 AWW<br>3 MO<br>4 CHO<br>9999 None
couns_1 *(required)*	Health<br>*Response constrained to: not(selected(${couns_1} , '9999') and count-selected(${couns_1}) > 1) or not(selected(${couns_1} , '9999'))*	1 ASHA<br>2 AWW<br>3 MO<br>4 CHO<br>9999 None
couns_2 *(required)*	Nutrition<br>*Response constrained to: not(selected(${couns_2} , '9999') and count-selected(${couns_2}) > 1) or not(selected(${couns_2} , '9999'))*	1 ASHA<br>2 AWW<br>3 MO<br>4 CHO<br>9999 None
couns_3 *(required)*	Mental health<br>*Response constrained to: not(selected(${couns_3} , '9999') and count-selected(${couns_3}) > 1) or not(selected(${couns_3} , '9999'))*	1 ASHA<br>2 AWW<br>3 MO<br>4 CHO<br>9999 None
couns_4 *(required)*	Personal hygiene<br>*Response constrained to: not(selected(${couns_4} , '9999') and count-selected(${couns_4}) > 1) or not(selected(${couns_4} , '9999'))*	1 ASHA<br>2 AWW<br>3 MO<br>4 CHO<br>9999 None
couns_5 *(required)*	Safe drinking water<br>*Response constrained to: not(selected(${couns_5} , '9999') and count-selected(${couns_5}) > 1) or not(selected(${couns_5} , '9999'))*	1 ASHA<br>2 AWW<br>3 MO<br>4 CHO<br>9999 None
couns_6 *(required)*	Sanitation<br>*Response constrained to: not(selected(${couns_6} , '9999') and count-selected(${couns_6}) > 1) or not(selected(${couns_6} , '9999'))*	1 ASHA<br>2 AWW<br>3 MO<br>4 CHO<br>9999 None
couns_7 *(required)*	Routine check up in facilities<br>*Response constrained to: not(selected(${couns_7} , '9999') and count-selected(${couns_7}) > 1) or not(selected(${couns_7} , '9999'))*	1 ASHA<br>2 AWW<br>3 MO<br>4 CHO<br>9999 None
couns_8 *(required)*	Postpartum family planning till health condition and nutritional status improved	1 ASHA

**Field**	**Question**	**Answer**
	Response constrained to: not(selected(${couns_8} , '9999') and count-selected(${couns_8}) > 1) or not(selected(${couns_8} , '9999'))	2 AWW<br>3 MO<br>4 CHO<br>9999 None
f_31_b *(required)*	Have you consumed or are you presently consuming any of the following?<br>क्या आप निम्नलिखित में से किसी का सेवन किया है या कर रहे हैं?<br>*After Delivery*<br>*Response constrained to: not(selected(${f_31_b} , '9999') and count-selected(${f_31_b}) > 1) or not(selected(${f_31_b} , '9999'))*	1 Iron and Folic Acid (IFA) tablets<br>8 Iron and Folic Acid (IFA) syrup<br>7 Folic Acid<br>2 Combination of Calcium & Vitamin-D<br>3 Calcium Tablet<br>4 Vitamin-D<br>5 Multiple Micronutrient Supplements (MMS)<br>6 Multi Vitamin<br>99 Others(medicinal suppliment)<br>9999 None
Postnatal Mother(0 - 6) > f_med_status_g_b<br>*Group relevant when: not(selected(${f_31_b} , '9999'))*		
Postnatal Mother(0 - 6) > f_med_status_g_b > [med_names_b] (1)		(Repeated group)
med_status_b *(required)*	When did you consume [med_names_b]?<br>आपने [med_names_b] की गोली कब ली है?	1 Consumed in the past पहले ली थी<br>2 Currently consuming अभी ले रही
f_33_4_4_b *(required)*	Since you started, for how many total days have you consumed [med_names_b]?<br>(Approximate number of days)<br>[med_names_b] या गोली लेना शुरू करने के बाद से अब तक आपने कुल कितने दिनों तक गोली ली है?<br>(कृपया अनुमानित दिनों की संख्या बताएं)<br>*Response constrained to: .>= 1 and .<=180*	
f_33_4_5_b *(required)*	How many days in a week do/did you consume [med_names_b]?<br>(Enter number of days: 0 to 7)<br>आप एक सप्ताह में कितने दिन [med_names_b] या गोली लेती हैं/थीं?<br>(दिनों की संख्या दर्ज करें: 0 से 7)<br>*Response constrained to: .>= 1 and .<=7*	
f_33_4_1_b *(required)*	How many [med_names_b] tablets do/did you consume in a day?<br>आप एक दिन में कितनी [med_names_b] या गोलियाँ लेती हैं/थीं?<br>*Response constrained to: .>= 1 and .<=3*	
f_33_4_2_b *(required)*	What is your [med_names_b] consumption pattern?	1 Regularly<br>2 Irregularly
f_text_b *(required)*	If you consume or consumed [med_names_b] irregularly, what are the reasons for missing the tablets?<br>अगर आप अनियमित रूप से [med_names_b] लेती हैं/थीं, तो Syrup या गोली न लेने के क्या कारण हैं	
source_of_supp3 *(required)*	[med_names_a] Received from	1 Received from Goverment<br>2 Purchase from Private
albenda2 *(required)*	Do you consume albendazole tablet	1 Yes<br>2 No
date_albanda_b *(required)*	When was the last time albendazole tablet was consumed?<br>आखिरी बार एल्बेंडाजोल टैबलेट का सेवन कब किया गया था?<br>*Question relevant when: ${albenda2} =1*	
mental_health_as *(required)*	Were any question asked to you to understand how do you feel<br>क्या आपसे यह समझने के लिए कोई प्रश्न पूछा गया था कि आप कैसा महसूस करते हैं	1 Yes<br>2 No
f_2_60 *(required)*	Are you happy/satisfied with the services provided at the post-natal services, including referral?<br>क्या आप रेफरल सहित प्रसवोत्तर सेवाओं में प्रदान की जाने वाली सेवाओं से खुश/संतुष्ट हैं?	1 Yes<br>2 No
f_2_61 *(required)*	How would you rate your experience?<br>आप अपने अनुभव का मूल्यांकन कैसे करेंगे?<br>*Please enter value between 1 to 5 (1 is lowest 5 is highest)*<br>*Question relevant when: ${f_2_60} =1*<br>*Response constrained to: .>= 0 and .<=5*	
post_remarks *(required)*	Respondent remarks<br>*Question relevant when: ${d_10} =1*	
post_interviewer_remark *(required)*	Interviewer remarks<br>*Question relevant when: ${d_10} =1*	

**Field**	**Question**	**Answer**
Early childhood Care Status (0 - 24 month)<br>*Group relevant when: ${d_11} =1*		
g_3 *(required)*	ID of the Child<br>बच्चे की पहचान<br>*Enter the value between 501 - 599*<br>*Question relevant when: (${child_dob_pn1} < 6)*<br>*Response constrained to: regex(., '^(?!500$)[5][0-9]{2}$')*	
Early childhood Care Status (0 - 24 month) > child_group624		
Early childhood Care Status (0 - 24 month) > child_group624 > child_group		
g_1 *(required)*	Name of the Child<br>बच्चे का नाम<br>*Response constrained to: not(regex(.,'^(.*)\d(.*)$'))*	
g_3_id *(required)*	ID of the Child<br>बच्चे की पहचान<br>*Enter the value between 601 - 699*<br>*Question relevant when: (${child_dob_pn1} >= 6 and not(selected(${child_dob_pn} , ''))) or (${child_dob_pn1} = '')*<br>*Response constrained to: regex(., '^(?!600$)[6][0-9]{2}$')*	
g_10 *(required)*	Name of the Mother<br>माता का नाम<br>*Question relevant when: (${child_dob_pn1} >= 6 and not(selected(${child_dob_pn} , ''))) or (${child_dob_pn1} = '')*<br>*Response constrained to: not(regex(.,'^(.*)\d(.*)$'))*	
g_11 *(required)*	Age of the Mother<br>माँ की उम्र<br>*Question relevant when: (${child_dob_pn1} >= 6 and not(selected(${child_dob_pn} , ''))) or (${child_dob_pn1} = '')*<br>*Response constrained to: .>= 18 and .<=45*	
g_12 *(required)*	Name of the Father<br>पिता का नाम<br>*Question relevant when: (${child_dob_pn1} >= 6 and not(selected(${child_dob_pn} , ''))) or (${child_dob_pn1} = '')*<br>*Response constrained to: not(regex(.,'^(.*)\d(.*)$'))*	
g_13 *(required)*	Age of Father<br>पिता की आयु<br>*Question relevant when: (${child_dob_pn1} >= 6 and not(selected(${child_dob_pn} , ''))) or (${child_dob_pn1} = '')*<br>*Response constrained to: .>= 18 and .<=55*	
g_14 *(required)*	How many years of schooling has the woman completed?<br>महिला ने कितने साल की स्कूली शिक्षा पूरी की है?<br>*Question relevant when: (${child_dob_pn1} >= 6 and not(selected(${child_dob_pn} , ''))) or (${child_dob_pn1} = '')*<br>*Response constrained to: .>= 0 and .<=25*	
g_15 *(required)*	Occupation of the Mother<br>माता का व्यवसाय<br>*Question relevant when: (${child_dob_pn1} >= 6 and not(selected(${child_dob_pn} , ''))) or (${child_dob_pn1} = '')*	1 Student<br>2 Unemployed<br>3 Homemaker<br>4 Agriculture<br>5 Business/Entrepreneur<br>6 Govt Job<br>7 Pvt Job<br>8 Daily Wage<br>99 Others (specify)
g_16 *(required)*	How many years of schooling has the Father completed?<br>पिता ने कितने वर्ष की स्कूली शिक्षा पूरी की है?<br>*Question relevant when: (${child_dob_pn1} >= 6 and not(selected(${child_dob_pn} , ''))) or (${child_dob_pn1} = '')*<br>*Response constrained to: .>= 0 and .<=25*	
g_17 *(required)*	Occupation of Father<br>पिता का व्यवसाय<br>*Question relevant when: (${child_dob_pn1} >= 6 and not(selected(${child_dob_pn} , ''))) or (${child_dob_pn1} = '')*	1 Student<br>2 Unemployed<br>3 Homemaker<br>4 Agriculture<br>5 Business/Entrepreneur<br>6 Govt Job

Field	Question	Answer
		7 Pvt Job<br>8 Daily Wage<br>99 Others (specify)
g_17_1_1 *(required)*	Gender of the baby<br>बच्चे का लिंग	1 Male<br>2 Female
g_17_2 *(required)*	Date of Birth of the child<br>बच्चे की जन्म तिथि<br>*Response constrained to: . >= today() - (24 * 30.4375) and . <= today()*	
g_17_3_1	Age of child(in Month)<br>[g_17_3]<br>बच्चे की आयु (महीने में)<br>[g_17_3]	
g_2_3 *(required)*	Place of Birth<br>जन्म स्थान	1 Home<br>2 Institution (Government- type of facility)<br>3 Institution (Private- type of facility)<br>4 Delivery during transit
g_2_3_1 *(required)*	Who conducted the delivery?<br>डिलीवरी किसने करवायी?<br>*Question relevant when: ${g_2_3} =1*	1 Doctor<br>2 Nurse<br>3 Midwife<br>4 Dai<br>5 Relatives<br>6 ASHA<br>99 Other
g_2_3_1_govt *(required)*	Please specify name and type of goverment institution<br>कृपया सरकारी संस्थान का नाम और प्रकार निर्दिष्ट करें<br>*Question relevant when: ${g_2_3} =2*	
g_2_3_1_pvt *(required)*	Please specify name and type of private institution<br>कृपया निजी संस्थान का नाम और प्रकार निर्दिष्ट करें<br>*Question relevant when: ${g_2_3} =3*	
f_2_3_1_trn *(required)*	Please specify (e.g Ambulance)<br>कृपया निर्दिष्ट करें (जैसे एम्बुलेंस)<br>*Question relevant when: ${g_2_3} =4*	
Early childhood Care Status (0 - 24 month) > child_group624 > child_anthro		
g_2_4 *(required)*	Weight at the time of Birth (in kg)<br>जन्म के समय वजन (किलो में)<br>*Please enter a value between 1.00 – 10.00 kg, NA = No*<br>*Response constrained to: ((${g_2_4} != 'NA' and regex(${g_2_4} , '^\d{1,2}\.\d{2}$') and number(${g_2_4}) >= 1 and number(${g_2_4}) <= 10) or ${g_2_4} = 'NA')*	
g_2_4_1 *(required)*	Source document of birth weight	1 Reported by Beneficiary<br>2 Verified from document
g_2_5 *(required)*	Gestational age at birth (in weeks)<br>जन्म के समय गर्भकालीन आयु (हफ्तों में)<br>*Response constrained to: .>= 0 and .<=42*	
g_2_5_1 *(required)*	Gestational age at birth (in days)<br>जन्म के समय गर्भकालीन आयु (दिनों में)<br>*Response constrained to: .>= 0 and .<=6*	
Early childhood Care Status (0 - 24 month) > Child 0 - 24 months बच्चा 0 - 24 महीने		
Early childhood Care Status (0 - 24 month) > Child 0 - 24 months बच्चा 0 - 24 महीने > Child 0 - 24 months - details बच्चा 0 - 24 महीने - विवरण		
g_anthro	Anthropometric Measurements of the child<br>बच्चे के एंथ्रोपोमेट्रिक माप	
g_20_1 *(required)*	Weight of the child in kg (Reading 1)<br>किलो में बच्चे का वजन (1 पढ़ना)<br>*Please enter a value between 0.00 – 25.00 kg, NA = No*<br>*Response constrained to: ((${g_20_1} != 'NA' and regex(${g_20_1} , '^\d{1,2}\.\d{2}$') and number(${g_20_1}) >= 0 and number(${g_20_1}) <= 25) or ${g_20_1} = 'NA')*	
g_20_1_1 *(required)*	Weight of the child in kg (Reading 2)<br>किलो में बच्चे का वजन (2 पढ़ना)<br>*Response constrained to: ((${g_20_1_1} != 'NA' and regex(${g_20_1_1} , '^\d{1,2}\.\d{2}$') and number(${g_20_1_1}) >= 0 and number(${g_20_1_1}) <= 25) or ${g_20_1_1} = 'NA')*	
g_20_2 *(required)*	Length of child in cms (Reading 1)<br>सेंटीमीटर में बच्चे की लंबाई (1 पढ़ना)	

Field	Question	Answer
	Please enter a value between 40.0 – 110.0 cm, NA = No<br>*Response constrained to: ((${g_20_2} != 'NA' and regex(${g_20_2} , '^\d{1,3}\.\d{1}$') and number(${g_20_2}) >= 40 and number(${g_20_2}) <= 110) or ${g_20_2} = 'NA')*	
g_20_2_1 *(required)*	Length of child in cms (Reading 2)<br>सेंटीमीटर में बच्चे की लंबाई (पढ़ना 2)<br>*Response constrained to: ((${g_20_2_1} != 'NA' and regex(${g_20_2_1} , '^\d{1,3}\.\d{1}$') and number(${g_20_2_1}) >= 40 and number(${g_20_2_1}) <= 110) or ${g_20_2_1} = 'NA')*	
Early childhood Care Status (0 - 24 month) > Child 0 - 24 months बच्चा 0 - 24 महीने > wsu_group		
g_29	Is the baby very low birth weight at time of birth?	
g_28	Was the baby low birth weight at time of birth?	
g_28_0	Was the baby preterm?	
g_20_3	Wasting	
g_20_4	Stunting	
g_20_5	Underweight	
g_30 *(required)*	How soon after birth was breastfeeding initiated (In hours)?<br>जन्म के बाद स्तनपान कितने समय में शुरू किया गया (घंटों में)?	
g_2_18	Does the mother/caregiver have MCP card<br>क्या मां/देखभाल करने वाले के पास एमसीपी कार्ड है	1 Yes<br>2 No
g_19 *(required)*	Are all relevant sections filled completely (check card)?<br>क्या सभी फ़ील्ड अपडेट किए गए हैं (चेक कार्ड)?<br>*Question relevant when: ${g_2_18} =1*	1 Yes<br>2 No
g_19_remark *(required)*	Please specify the blank fields<br>कृपया रिक्त फ़ील्ड निर्दिष्ट करें<br>*Question relevant when: ${g_19} =2*	1 Basic Indentications<br>2 TD medicinal supplements (page 6)<br>3 ANC specifc information (page 7/8)<br>4 PNC (page 13/14/15)<br>5 Danger Signs (page 19/30)<br>6 Growth monitoring (page 36/43)<br>7 Immunization record (page 44/45)
g_19_remark1	Please specify about the blank field in Basic Indentications<br>कृपया बेसिक पहचान में रिक्त फ़ील्ड के बारे में निर्दिष्ट करें<br>*Question relevant when: selected(${g_19_remark} ,1)*	
g_19_remark2	Please specify about the blank field in TD medicinal supplements (page 6)<br>कृपया टीडी औषधीय अनुपूरक (पृष्ठ 6) में रिक्त स्थान के बारे में बताएं<br>*Question relevant when: selected(${g_19_remark} ,2)*	
g_20_remark3	Please specify about the blank field in ANC specifc information (page 7/8)<br>कृपया ANC विशिष्ट जानकारी में रिक्त फ़ील्ड के बारे में बताएं (पृष्ठ 7/8)<br>*Question relevant when: selected(${g_19_remark} ,3)*	
g_21_remark4	Please specify about the blank field in PNC (page 13/14/15)<br>कृपया PNC में रिक्त फ़ील्ड के बारे में बताएं (पृष्ठ 13/14/15)<br>*Question relevant when: selected(${g_19_remark} ,4)*	
g_22_remark5	Please specify about the blank field in ECD (page 19/30)<br>कृपया ECD में रिक्त फ़ील्ड के बारे में बताएं (पृष्ठ 19/30)<br>*Question relevant when: selected(${g_19_remark} ,5)*	
g_23_remark6	Please specify about the blank field in Growth monitoring (page 36/43) कृपया ग्रोथ मॉनिटरिंग में रिक्त फ़ील्ड के बारे में बताएं (पृष्ठ 36/43)<br>*Question relevant when: selected(${g_19_remark} ,6)*	
g_23_remark7	Please specify about the blank field in Immunization record (page 44/45) कृपया टीकाकरण रिकॉर्ड में रिक्त स्थान के बारे में बताएं (पृष्ठ 44/45)<br>*Question relevant when: selected(${g_19_remark} ,7)*	
g_22 *(required)*	Any problem identified by the health worker (in addition to mother's report, check MCP card if available)?<br>स्वास्थ्य कार्यकर्ता द्वारा पहचानी गई कोई समस्या (मां की रिपोर्ट के अलावा, यदि उपलब्ध हो तो एमसीपी कार्ड की जांच करें)?<br>*Response constrained to: not(selected(${g_22} , '9') and count-selected(${g_22}) > 1) or not(selected(${g_22} , '9'))*	1 Inadequate weight gain<br>2 Congenital Malformation<br>3 Any danger signs<br>4 Delayed development<br>5 Stunted<br>6 Wasted<br>7 Underweight<br>8 Anaemic<br>99 Others(specify)<br>9 No problem Identified

Field	Question	Answer
		10 No record available
g_22_source *(required)*	Source documents of problems<br>*Question relevant when: not(selected(${g_22} , '9'))*	1 Prescription<br>2 MCP Card<br>3 Reported by Mother<br>4 Lab Report<br>99 Others(specify)
g_23 *(required)*	If the child had any of the problems mentioned above, what was done ?<br>यदि बच्चे को ऊपर वर्णित समस्याओं में से कोई भी समस्या थी, तो क्या किया गया था?<br>*Question relevant when: not(selected(${g_22} , '9'))*	1 Counselling on feeding by ASHAs<br>2 Counselling on feeding by ANMs<br>3 Counselling on feeding by AWWs<br>4 EBF till 6 months<br>5 Complementary feeding from 6 months of age<br>6 CF type of food, consistency<br>9 Responsive feeding(बच्चे को खेल खेल में खिलाना)<br>10 Feeding during illness<br>11 Referred to health facilities<br>99 Others(specify)
g_24_1 *(required)*	Consistancy of complementry food<br>*Question relevant when: selected(${g_23} ,6)*	1 Solid<br>2 Semi Solid<br>3 Liquid
g_43 *(required)*	Has the child received all recommended vaccinations for their age? (Please ask the mother and verify using the MCP card, if available).<br>क्या बच्चे को उसकी उम्र के अनुसार सभी अनुशंसित टीके मिले हैं?<br>(कृपया मां से पूछें और यदि उपलब्ध हो तो एमसीपी कार्ड का उपयोग करके सत्यापित करें)।	1 Yes Reported<br>3 Yes Documented<br>2 No
g_37_1_d2 *(required)*	In the last one month, have your child have been diagnosed or suffered from any of the following condition/Illness?<br>पिछले एक महीने में, क्या आपके बच्चे का निम्नलिखित में से किसी भी स्थिति / बीमारी का निदान किया गया है या इनसे ग्रासित हुआ है?<br>*Response constrained to: not(selected(${g_37_1_d2} , '9999') and count-selected(${g_37_1_d2}) > 1) or not(selected(${g_37_1_d2} , '9999'))*	1 Pneumonia<br>2 Fast breathing (tej saans)<br>3 Convulsions<br>4 Diarrhea<br>5 Dehydration<br>6 Fever<br>7 Jaundice<br>8 Hypothermia<br>9 Feeding issues<br>10 Sepsis<br>11 PSBI(fast breathing, severe chest indrawing, fever or hypothermia, poor feeding, and altered level of consciousness)<br>99 Others(Specify)<br>9999 No Disease/Illness
g_37_1_d2_source *(required)*	Source document of the problem<br>*Question relevant when: not(selected(${g_37_1_d2} , '9999'))*	1 Reported by Beneficiary<br>2 Verified from document
g_37_1_d2_source1 *(required)*	Type of document<br>*custom-specify-other(other=99)*<br>*Question relevant when: ${g_37_1_d2_source} =2*	1 Prescription<br>2 Lab report<br>3 Government card(MCP)<br>99 Other
g_38_d2 *(required)*	Did you seek treatment outside of home<br>क्या आपने घर के बाहर इलाज कराया<br>*Question relevant when: not(selected(${g_37_1_d2} , '9999'))*	1 Yes<br>2 No
g_39_d2 *(required)*	From whom?<br>किससे?<br>*Question relevant when: ${g_38_d2} =1*	1 HWC<br>2 CHC<br>3 PHC<br>4 Civil Hospital(CH)<br>5 Regional Hospital<br>6 ASHA<br>7 ANM

Field	Question	Answer
		8 Medical college<br>9 Private provider clinic(Without in-patient bed)<br>10 Private hospital(With in-patient bed)<br>11 Faith healers/traditional health care providers<br>99 Others(Specify)
g_40_d2 *(required)*	Do you have any treatment related documents(investigation reports, prescriptions, if admitted discharge summary, tablet strips, syrup bottles)<br>क्या आपके पास उपचार से संबंधित कोई दस्तावेज हैं (जांच रिपोर्ट, नुस्खे, यदि भर्ती कराया गया है तो डिस्चार्ज summary, टैबलेट स्ट्रिप्स, सिरप की बोतलें)<br>*Question relevant when: ${g_38_d2} =1*<br>*Response constrained to: not(selected(${g_40_d2} , '3') and count-selected(${g_40_d2}) > 1) or not(selected(${g_40_d2} , '3'))*	1 Documents<br>2 Medicine(Tablets strips/Syrup)<br>3 None of the above
g_40_d2_1 *(required)*	Upload Document<br>दस्तावेज़ अपलोड करें<br>*Question relevant when: selected(${g_40_d2} ,'1')*	
g_40_d2_2 *(required)*	Mention the name of medicine<br>दवा के नाम का उल्लेख करें<br>*Question relevant when: selected(${g_40_d2} ,'2')*	
g_41_d2 *(required)*	Was your child admitted to hospital in the last 3 months?<br>क्या आपका बच्चा पिछले 3 महीनों में अस्पताल में भर्ती था?	1 Yes<br>2 No
g_41_d2_1 *(required)*	Place of admission<br>*Question relevant when: ${g_41_d2} =1*	1 Goverment Facility<br>2 Private Facility
g_42_d2 *(required)*	For what condition<br>किस स्थिति के लिए भर्ती कराया गया है<br>*Question relevant when: ${g_41_d2} =1*	
g_23_2 *(required)*	Was the child referred to a facility?<br>क्या बच्चे को स्वास्थ्य केंद्र के लिए रेफर किया गया था?<br>*Question relevant when: ${g_41_d2} =1*	1 Yes<br>2 No
g_23_2_reason *(required)*	Reason for referral<br>*Question relevant when: ${g_23_2} =1*	
g_24 *(required)*	If referred, was the child taken to the facility<br>यदि रेफर किया गया था, तो क्या बच्चे को स्वास्थ्य केंद्र में ले जाया गया था<br>*Question relevant when: ${g_23_2} =1*	1 Yes<br>2 No
g_25 *(required)*	Where was the child taken?<br>बच्चे को कहां ले जाया गया?<br>*Question relevant when: ${g_24} =1*	1 HWC<br>2 CHC<br>3 PHC<br>4 Civil Hospital(CH)<br>5 Regional Hospital<br>6 ASHA<br>7 ANM<br>8 Medical college<br>9 Private provider clinic(Without in-patient bed)<br>10 Private hospital(With in-patient bed)<br>11 Faith healers/traditional health care providers<br>99 Others(Specify)
g_26 *(required)*	Did you face any challenges in accessing healthcare for your child?<br>क्या आपको अपने बच्चे के लिए स्वास्थ्य केंद्र से सुविधा लेने में किसी चुनौती का सामना करना पड़ा?	1 Yes<br>2 No
g_26_1 *(required)*	What were the challenges that you faced?<br>आपको किन चुनौतियों का सामना करना पड़ा?<br>*Question relevant when: ${g_26} =1*	1 Doctors not available<br>2 Long waiting hours<br>3 Medicines not available<br>4 No laboratory facilities<br>5 Long distances<br>6 Lack of transportation<br>7 Timing unpredictable<br>8 Rude behavior of health staff<br>99 Others(specify)

Field	Question	Answer
g_27 *(required)*	Did your baby receive Vitamin D supplementation before 6 month age ? क्या आपके बच्चे को 6 महीने की उम्र से पहले विटामिन डी की खुराक मिली है?	1 Yes<br>2 No
g_27_1_1 *(required)*	Did your baby consume Vitamin D supplementation before 6 month age ? क्या आप के बच्चे ने 6 महीने की उम्र से पहले विटामिन डी अनुपूरण लिया था?<br>*Question relevant when: ${g_27} =1*	1 Yes<br>2 No
g_27_1 *(required)*	Is your baby receiving Vitamin D supplementation currently?<br>क्या आप के बच्चे को वर्तमान में विटामिन डी की खुराक मिल रही हैं?<br>*Question relevant when: ${g_27} =1*	1 Yes<br>2 No
g_27_2 *(required)*	Is your baby currently consuming Vitamin D supplementation currently? क्या आपका बच्चा वर्तमान में विटामिन डी अनुपूरक ले रहा है?<br>*Question relevant when: ${g_27_1} =1*	1 Yes<br>2 No
g_28_1 *(required)*	Is the baby receiving vitamin syrup/micronutrient supplementation?<br>क्या बच्चे को विटामिन सिरप/माइक्रोन्यूट्रिएंट सप्लीमेंट मिल रहा है?	1 Yes<br>2 No
g_28_1_1 *(required)*	Is the baby consuming vitamin syrup/micronutrient supplementation?<br>क्या बच्चा विटामिन सिरप/माइक्रोन्यूट्रिएंट सप्लीमेंट का सेवन कर रहा है?<br>*Question relevant when: ${g_28_1} =1*	1 Yes<br>2 No
g_29_1 *(required)*	Is the baby receiving Iron & Folic Acid?<br>क्या बच्चा आयरन और फोलिक एसिड प्राप्त कर रहा है?	1 Yes<br>2 No
g_29_1_1 *(required)*	Is the baby consuming Iron & Folic Acid?<br>क्या बच्चा आयरन और फोलिक एसिड का सेवन कर रहा है?<br>*Question relevant when: selected(${g_29_1} ,1)*	1 Yes<br>2 No
Early childhood Care Status (0 - 24 month) > Child 0 - 24 months बच्चा 0 - 24 महीने > month_care6<br>*Group relevant when: if(${g_17_3} <6,1,2)*		
g_32_1 *(required)*	At what age (in completed months) was complementary feeding initiated? (Specify age in months)<br>किस उम्र में (पूरा महीना) पूरक आहार शुरू किया गया था? (महीनों में आयु निर्दिष्ट करें)<br>*If No fill 999*	
g_34 *(required)*	Do you get any food from the AWC for infant/children?<br>क्या शिशु/बच्चों लिए आंगनवाड़ी केंद्र से कोई भोजन/राशन प्राप्त करते हैं?	1 Yes<br>2 No
diet_nutri_624_29 *(required)*	If not receiving supplement feed , give reasons<br>यदि पूरक फ़ीड प्राप्त नहीं हो रहा है, तो कारण बताएं<br>*Question relevant when: ${g_34} =2*	1 AWC not accessible<br>2 Not Offered at AWCs<br>3 Supplement food is not of good quality<br>4 Causing ill health<br>5 Time/place not convenient<br>6 Not free of cost<br>7 Does not need<br>99 Others(Specify)<br>8 NA
g_35 *(required)*	Was the food provided by the AWC entirely consumed by the infant/children?<br>*Question relevant when: ${g_34} =1*	1 Yes<br>2 No
g_36	Did they share it with family members?<br>*Question relevant when: ${g_35} =2*	1 Yes<br>2 No
Early childhood Care Status (0 - 24 month) > Child 0 - 24 months बच्चा 0 - 24 महीने > month_care6 > Supplementary Feeding Program<br>एसएनपी<br>*Group relevant when: ${g_34} =1*		
Early childhood Care Status (0 - 24 month) > Child 0 - 24 months बच्चा 0 - 24 महीने > month_care6 > Supplementary Feeding Program<br>एसएनपी > Supplementary Nutrition		
Early childhood Care Status (0 - 24 month) > Child 0 - 24 months बच्चा 0 - 24 महीने > month_care6 > Supplementary Feeding Program<br>एसएनपी > Supplementary Nutrition > food_practices2_1_1_awc_c		
j_1_3_1_awc_c *(required)*	Type of Food Received<br>प्रदान किए गए भोजन का प्रकार	1 Hot Cooked Meal<br>2 THR
Early childhood Care Status (0 - 24 month) > Child 0 - 24 months बच्चा 0 - 24 महीने > month_care6 > Supplementary Feeding Program<br>एसएनपी > Supplementary Nutrition > food_practices2_1_1_awc_c > hcm_1_awc_c<br>*Group relevant when: selected(${j_1_3_1_awc_c} , '1')*		
hcm_1_note_awc_c	Hot Cooked Meal<br>गर्म पका हुआ भोजन	
hot_cooke_name_c *(required)*	Specify Hot cooked meal name	
j_1_13_1_awc_c *(required)*	Quantity consumed per day(in gram)<br>प्रति दिन खायी गयी मात्रा (ग्राम में)<br>*Response constrained to: .>= 0 and .<=200*	
j_1_14_1_awc_c *(required)*	Variation in Quantity/Type Based on Beneficiary Condition<br>लाभार्थी की स्थिति के आधार पर मात्रा/प्रकार में भिन्नता<br>*If yes, specify*	1 Yes<br>2 No

Field	Question	Answer
Early childhood Care Status (0 - 24 month) > Child 0 - 24 months बच्चा 0 - 24 महीने > month_care6 > Supplementary Feeding Program<br>एसएनपी > Supplementary Nutrition > food_practices2_1_1_awc_c > thr_1_awc_c<br>*Group relevant when: selected(${j_1_3_1_awc_c} , '2')*		
thr_text1_awc_c *(required)*	THR (Take home ration) THR	1 Channa<br>2 Dalia<br>3 Jaggary<br>4 Oil<br>5 Panjiri<br>6 Rajma<br>7 Rice<br>8 Salt<br>9 Sevia<br>10 Soya<br>11 Salt Biscuit<br>12 Sweet Biscuit<br>13 WMP(Milk)<br>14 Milk<br>15 Egg<br>26 Black Chana<br>27 Chane Ki Daal<br>16 Wings1<br>17 Wings2<br>18 Wings3<br>19 Wings4<br>20 Wings5<br>21 Wings6<br>22 Wings7<br>23 Wings8<br>24 Wings9<br>25 Wings10<br>99 Other
Early childhood Care Status (0 - 24 month) > Child 0 - 24 months बच्चा 0 - 24 महीने > month_care6 > Supplementary Feeding Program<br>एसएनपी > Supplementary Nutrition > food_practices2_1_1_awc_c > thr_1_awc_c > THR [thr_cal_repeat_c] (1)		(Repeated group)
hot_total_awc_c *(required)*	Total Quantity receive in a month(in gram)<br>*Response constrained to: .>= 0*	
week_c	Receive for how many days in a week<br>*Response constrained to: .>= 0 and .<=7*	
quantity_p3_c *(required)*	Quantity receive per day(in gram)<br>प्रति दिन कितना मिला (ग्राम में)<br>*Response constrained to: .>= 0 and .<=200*	
j_1_17_1_awc_c *(required)*	Quantity Consumed in gram (Per Day)<br>प्रति दिन खायी गयी मात्रा (ग्राम में)<br>*Response constrained to: .>= 0 and .<=200*	
j_1_18_1_awc_c *(required)*	Variation in Quantity/Type Based on Beneficiary Condition<br>लाभार्थी की स्थिति के आधार पर मात्रा/प्रकार में भिन्नता<br>*If yes, specify*	1 Yes<br>2 No
g_40 *(required)*	WASH (Water, Sanitation, and Hygiene) WASH<br>(जल, स्वच्छता और स्वच्छता)<br>*Response constrained to: not(selected(${g_40} , '9999') and count-selected(${g_40}) > 1) or not(selected(${g_40} , '9999'))*	1 Clean play area<br>2 Safe disposal of feces:<br>3 Hand washing:<br>4 Safe drinking water:<br>99 Others(specify)<br>9999 None
Early childhood Care Status (0 - 24 month) > Child 0 - 24 months बच्चा 0 - 24 महीने > month_care6 > Children aged 6 -24 months(Nutrition) 6 -24 महीने की आयु के बच्चे (पोषण)<br>*Group relevant when: ${g_17_3} >= 6*		
diet_nutri_624_1 *(required)*	What type of feeding is currently being given to the child?	1 Water<br>2 EBM (Expressed Breast Milk)<br>3 Formula milk<br>5 Snacks<br>6 Direct breastfeeding<br>7 Cow /goat/ buffalo milk diluted with water

Field	Question	Answer
		8 Cow /goat/ buffalo milk undiluted<br>9 ICDS Supplement<br>10 Commercial baby foods<br>11 Processed Foods (Biscuits)<br>12 Homemade semi-solids (Khichadi, Dalia, churma etc.)<br>13 Homemade solids<br>14 Energy-dense CF (with added oil)<br>15 Iron-rich CF
diet_nutri_624_2 *(required)*	Till what age your child fed only breast-milk (Not even water)?<br>Age (in completed months)<br>किस उम्र तक आपके बच्चे को केवल स्तन का दूध (बिना पानी के) पिलाया जाता है?<br>आयु (पूर्ण महीनों में)<br>*Fill 77 if the child is being EBF currently*	
diet_nutri_624_3 *(required)*	When did you start giving complementary food to your child?<br>Age (in completed Months)<br>आपने अपने बच्चे को पूरक भोजन देना कब शुरू किया? आयु (पूर्ण महीनों में)<br>*Enter '77' if not started*<br>*Question relevant when: ${diet_nutri_624_2} != 77*	
Early childhood Care Status (0 - 24 month) > Child 0 - 24 months बच्चा 0 - 24 महीने > month_care6 > Children aged 6 -24 months(Nutrition) 6 -24 महीने की आयु के बच्चे (पोषण) > diet_group_nutri		
diet_nutri_624_5	What are the foods generally included in complementary foods and how many times in a week?<br>पूरक खाद्य पदार्थों में आम तौर पर कौन से खाद्य पदार्थ शामिल होते हैं और सप्ताह में कितनी बार?	
diet_nutri_624_6 *(required)*	Cereal (rice/wheat)<br>अनाज (चावल/गेहूं)<br>*No of times*<br>*Response constrained to: .>= 0 and .<=42*	
diet_nutri_624_7 *(required)*	Legumes/Lentils (beans, peas)<br>फलियां / दाल (बीन्स, मटर)<br>*No of times*<br>*Response constrained to: .>= 0 and .<=42*	
diet_nutri_624_8 *(required)*	Green leafy vegetables<br>हरी पत्तेदार सब्जियां<br>*No of times*<br>*Response constrained to: .>= 0 and .<=42*	
diet_nutri_624_9 *(required)*	Other vegetables<br>अन्य सब्जियां<br>*No of times*<br>*Response constrained to: .>= 0 and .<=42*	
diet_nutri_624_10 *(required)*	Fruits (guava, orange, apple, pomegranate)<br>फल (अमरूद, संतरा, सेब, अनार)<br>*No of times*<br>*Response constrained to: .>= 0 and .<=42*	
diet_nutri_624_11 *(required)*	Meat<br>मांस<br>*No of times*<br>*Response constrained to: .>= 0 and .<=42*	
diet_nutri_624_12 *(required)*	Fish<br>मछली<br>*No of times*<br>*Response constrained to: .>= 0 and .<=42*	
diet_nutri_624_13 *(required)*	Eggs<br>अंडे<br>*No of times*<br>*Response constrained to: .>= 0 and .<=42*	
diet_nutri_624_14 *(required)*	Milk<br>दूध<br>*No of times*<br>*Response constrained to: .>= 0 and .<=42*	
diet_nutri_624_15 *(required)*	Milk products (yogurt, paneer)<br>दूध उत्पाद (दही, पनीर)<br>*No of times*<br>*Response constrained to: .>= 0 and .<=42*	
diet_nutri_624_16 *(required)*	Jaggery (gudd)<br>गुड़ (गुड़)<br>*No of times*	

Field	Question	Answer
	Response constrained to: .>= 0 and .<=42	
diet_nutri_624_17 *(required)*	Red meat<br>लाल मांस<br>*No of times*<br>*Response constrained to: .>= 0 and .<=42*	
diet_nutri_624_18 *(required)*	Millets (ragi, bajra, jawar)<br>बाजरा (रागी, बाजरा, ज्वार)<br>*No of times*<br>*Response constrained to: .>= 0 and .<=42*	
diet_nutri_624_19 *(required)*	Soy product<br>सोया उत्पाद<br>*No of times*<br>*Response constrained to: .>= 0 and .<=42*	
diet_nutri_624_20 *(required)*	Lemon/amla<br>नींबू/आंवला<br>*No of times*<br>*Response constrained to: .>= 0 and .<=42*	
diet_nutri_624_28 *(required)*	Nuts and oil seeds<br>मेवे और तेल के बीज<br>*No of times<br>(Eg: Almond, Walnuts, Peanut, Sesame, Sunflower seeds etc)*<br>*Response constrained to: .>= 0 and .<=42*	
diet_nutri_624_30 *(required)*	Fats and oils<br>वसा और तेल<br>*No of times<br>(Butter, Ghee, Mustard oil, Coconut oil etc.)*<br>*Response constrained to: .>= 0 and .<=42*	
diet_nutri_624_21 *(required)*	Any fortified rice/wheat<br>कोई भी फोर्टिफाइड चावल/गेहूं<br>*No of times*<br>*Response constrained to: .>= 0 and .<=42*	
diet_nutri_624_23 *(required)*	Other fortified foods<br>अन्य फोर्टिफाइड खाद्य पदार्थ<br>*No of times*<br>*Response constrained to: .>= 0 and .<=42*	
diet_nutri_624_24 *(required)*	If complementary food started, on an average how many times is complementary food given to a child in typical day?<br>No of times<br>यदि पूरक भोजन शुरू किया जाता है, तो एक बच्चे को एक दिन में औसतन कितनी बार पूरक भोजन दिया जाता है? समय की संख्या<br>*Response constrained to: .>= 0 and .<=10*	
diet_nutri_624_25 *(required)*	What is average quantity of food that your child has in each complementary feed?<br>प्रत्येक पूरक आहार में आपके बच्चे के भोजन की औसत मात्रा क्या है?<br>*Note in terms of bowl (Katori size- 150 ml / 1 tablespoon=0.03 Katori)*<br>*Response constrained to: .>= 0 and .<=8*	
diet_nutri_624_26 *(required)*	Does your child get Take home ration/hot cooked meal under SNP Supplementary Feeding Programme<br>क्या आपके बच्चे को एसएनपी पूरक आहार कार्यक्रम के तहत घर ले जाने का राशन/गर्म पका हुआ भोजन/राशन मिलता है	1 Yes<br>2 No
diet_nutri_624_27 *(required)*	In general, how many days in a month does your child get supplementary food from AWWs?<br>सामान्य तौर पर, आपके बच्चे को महीने में कितने दिन आंगनवाड़ी कार्यकत्रयों से पूरक आहार मिलता है?<br>*Question relevant when: ${diet_nutri_624_26} =1*	1 < 10 days/month<br>2 10-20 days/month<br>3 > 20 days/month
Early childhood Care Status (0 - 24 month) > Child 0 - 24 months बच्चा 0 - 24 महीने > month_care6 > ecd_group		
ecd_1 *(required)*	Do you take out time especially to play and communicate with your child?<br>क्या आप विशेष रूप से अपने बच्चे के साथ खेलने और संवाद करने के लिए समय निकालते हैं?	1 Yes<br>2 No
ecd_2 *(required)*	How much time did you spend playing and communicating with your child yesterday (in minutes)?<br>आपने कल (मिनटों में) अपने बच्चे के साथ खेलने और संवाद करने में कितना समय बिताया?<br>*Question relevant when: ${ecd_1} =1*<br>*Response constrained to: .>= 0 and .<=1440*	
ecd_3 *(required)*	Does the father play /communicate with the child?<br>क्या पिता बच्चे के साथ खेलता / संवाद करता है?	1 Yes<br>2 No
ecd_4 *(required)*	How much time did the father spend playing and communicating with the child yesterday (in minutes)?<br>पिता ने कल बच्चे के साथ खेलने और संवाद करने में कितना समय बिताया (मिनटों में)?<br>*Question relevant when: ${ecd_3} =1*<br>*Response constrained to: .>= 0 and .<=1440*	
addiction_1 *(required)*	Is there any addiction or substance abuse by the woman	1 Yes<br>2 No
substance_1	What type of substance is consumed<br>*Question relevant when: ${addiction_1} =1*	1 Tobacco smoking<br>2 Alcohol<br>3 Tobacco chewing

Field	Question	Answer
		99 Any other substance consumed, specify
addiction_2 *(required)*	Is there any addiction or substance abuse by the husband	1 Yes<br>2 No
substance_2	What type of substance is consumed<br>*Question relevant when: ${addiction_2} =1*	1 Tobacco smoking<br>2 Alcohol<br>3 Tobacco chewing<br>99 Any other substance consumed, specify
child_remarks	Respondent remarks<br>*Question relevant when: ${d_11} =1*	
child_interviewer_remark *(required)*	Interviewer remarks<br>*Question relevant when: ${d_11} =1*	
Healthservice Utilisation स्वास्थ्य सेवा उपयोग		
h_13 *(required)*	Have you been exposed to any Social Behaviour Change Communication (SBCC) activity in your area in the past 6 months?<br>पिछले 6 महीनों में क्या आपके क्षेत्र में ऐसी कोई गतिविधि हुई थी, जिसकी जानकारी से आपमें कुछ बदलाव आया हो या आप उससे जागरूक हुए हों	1 Interpersonal communication (home visit by ASHA, AWW, ANM) व्यक्तिगत संपर्क (आशा, आंगनवाड़ी या एएनएम द्वारा घर पर विज़िट)<br>2 Group meetings समूह बैठकें<br>3 Community events (like Poshan Diwas) सामुदायिक कार्यक्रम (जैसे पोषण दिवस)<br>4 Printed materials (posters, wall paintings, leaflets) मुद्रित सामग्री (पोस्टर, वॉल पेंटिंग, पर्चे)<br>5 Folk shows, puppet shows, street plays nukkad natak लोकनाट्य, कठपुतली शो, नुक्कड़ नाटक<br>6 Mobile messages or WhatsApp communication मोबाइल संदेश या व्हाट्सएप के माध्यम से जानकारी<br>7 Social media (YouTube, Facebook, Instagram, etc.) सोशल मीडिया (यूट्यूब, फेसबुक, इंस्टाग्राम आदि)<br>8 Radio programs रेडियो कार्यक्रम<br>9 Television programs टेलीविजन कार्यक्रम<br>99 Other (please specify): अन्य (कृपया बताएं):<br>10 Not exposed to any activity
h_15 *(required)*	Where did you see or hear this SBCC activity?<br>आपने यह SBCC गतिविधि कहाँ देखी या सुनी?<br>*Question relevant when: not(selected(${h_13} ,10))*	1 At Anganwadi Centre आंगनवाड़ी केंद्र में<br>2 During home visit by health worker घर पर स्वास्थ्यकर्मी के दौरे के दौरान<br>3 At VHND (Village Health and Nutrition Day) वीएचएनडी (ग्राम स्वास्थ्य एवं पोषण दिवस) पर<br>4 At Health Sub-centre or PHC उप-स्वास्थ्य केंद्र या प्राथमिक स्वास्थ्य केंद्र में<br>5 In school or community hall/panchayat ghar स्कूल या पंचायत भवन / सामुदायिक केंद्र में<br>6 In haat, bazaar or other public events हाट, बाजार या अन्य सार्वजनिक कार्यक्रम में<br>7 Through mobile (SMS, WhatsApp) मोबाइल (एसएमएस,

Field	Question	Answer
		व्हाट्सएप) के माध्यम से<br>8 On social media (YouTube, Facebook, Instagram) सोशल मीडिया (यूट्यूब, फेसबुक, इंस्टाग्राम) पर<br>9 On radio रेडियो पर<br>10 On television टेलीविजन पर<br>99 Other (please specify): अन्य (कृपया बताएं):
h_13_1 *(required)*	What was the topic or focus of the SBCC activity?<br>SBCC गतिविधि का विषय या फोकस क्या था?<br>*Question relevant when: not(selected(${h_13} ,10))*	1 Nutrition पोषण<br>2 WASH<br>3 Health screening and treatment स्वास्थ्य जांच और उपचार<br>4 Mental health or emotional well-being मानसिक स्वास्थ्य या भावनात्मक स्थिति<br>99 Other (please specify): अन्य (कृपया बताएं):
h_16 *(required)*	What could be the possible solutions for better health services utilization /mitigation strategies?<br>बेहतर स्वास्थ्य सेवाओं के उपयोग/न्यूनीकरण रणनीतियों के लिए संभावित समाधान क्या हो सकते हैं?	1 Care close to home घर के नजदीक देखभाल करें<br>2 Availability of doctors डॉक्टरों की उपलब्धता<br>3 Availability of transport परिवहन की उपलब्धता<br>4 Extended hours of service availability सेवा उपलब्धता के विस्तारित घंटे<br>5 Medicines should be provided दवाइयां उपलब्ध कराई जानी चाहिए<br>6 Provision for investigations should be available जांच के लिए प्रावधान उपलब्ध होना चाहिए<br>99 Others(specify)
picture_number *(required)*	Please enter the number of Picture/Document	
Picture/Document (1)<br>*Group relevant when: if(${picture_number} >0,1,2)*		(Repeated group)
picture *(required)*	Health/Nutrition record document<br>स्वास्थ्य/पोषण रिकॉर्ड दस्तावेज़	
file_photo	Please upload PDF Documents<br>कृपया पीडीएफ दस्तावेज अपलोड करें	
