# Supplementary material for: Implementation research to scale up the women and infants integrated interventions for growth study (WINGS) in Himachal Pradesh: Protocol for a quasi-experimental, mixed-methods study
Source: PLoS One. 2026 Feb 17;21(2):e0341048. doi: 10.1371/journal.pone.0341048 (PMC12912596; doi:10.1371/journal.pone.0341048)
Supplement: S7 File — (PDF) [file pone.0341048.s008.pdf]

WINGS Scale-UP– Proposal Narrative

Basic Information

Title Of The Proposal

Pilot Scale up Programme of WINGS interventions in Five Blocks of District Una (Amb, Dhundla/Thanakalan, Gagret, Haroli & Una/Basdhera) of Himachal Pradesh

Duration (In months)

36 months

Name of Organisation

Society for Applied Studies

Total Budget (Rs. in lakhs):

No. of Collaborators

Collaborator(s) Details

Collaborator(s)	Name of Contact Person	Email	Collaborator Type	MoU File
State Implementation Unit (H.P)	Ms M. Sudha Devi (IAS)<br>Secretary Health<br>Govt. of Himachal Pradesh<br>Phone: 0177-2621902	healthsecy-hp@nic.in		
	Sh Ashish Singhmar (IAS)<br>Social Justice & Empowerment<br>Govt. of Himachal Pradesh<br>Phone: 0177- 2620625	Socialjesecy-hp@nic.in		
	Ms Priyanka Verma (IAS)<br>Mission Director, National Health Mission Himachal Pradesh<br>Phone: 0177 2624505	md-hp-nrhm@nic.in		
	Dr Gopal Beri<br>Director Health Services<br>Himachal Pradesh<br>Phone: 0177-2621424	dirhealthdhs@gmail.com		
	Ms Rupali Thakur (IAS)<br>Director Women & Child Development<br>Himachal Pradesh<br>Phone: 0177-2622033, 2623113	wcd-hp@nic.in		
	Dr H R Thakur<br>Principal State Institute of Health & Family Welfare, Himachal Pradesh<br>Phone: 0177-2620226,2970136	primahalshimla@gmail.com		

Authorisation Letter for Submission of Proposal in Prescribed Format for each partner

Particular of the Applicant(s)

Name of the Principal Applicant

Dr. Sarmila Mazumder

Designation

Senior Scientist

Mobile

+91 9811681530

Applicant Team Members

S. No.	Name	Designation	Email	Landline	Mobile	Resume	Edit	
1.	Dr. Sunita Taneja	Senior Scientist	sunita.taneja@sas.org.in	011 46043751-55	+91-9811206456			
2.	Dr. Ranadip Chowdhury	Scientist	ranadip.chowdhury@sas.org.in	011 46043751-55	+91-7838870359			
3.	Dr. Neeta Dhabhai	Senior Clinical Researcher	neeta.dhabhai@sas.org.in	011 46043751-55	+91-8470068495			
4.	Dr. Nita Bhandari	Senior Scientist	nita.bhandari@sas.org.in	011 46043751-55	+91-			
5.	Dr. Barsha Gadapani Pathak	Research Scientist	Barsha.pathak@sas.org.in	011 46043751-55	+91-9920391586			
6.								
7.								
8.								
9.								

Contact Details:

Address:

Street/Village:

**City/Town:** 45, Kalu Sarai,	**Zip Pin code:** 110016
**State:** New Delhi	**Country:** India
**Fax:** +91 11 46043756	**Landline/Office Phone:** +91 11-46043751-55
**Email Address:**	**Website:** https://sas.org.in/

Brief Background of the Lead Organisation

**For Institution/ Universities/ Public Research Organization**	NA
Year of Establishment of the Institution	
Recognition or Accreditation Status	Attach File
R&D Activities (Area)	
Source of Core Funding:	

Annual Report for Previous Three Financial Year	Attach the file in PDF Format
**For Society/ Trust/ Ngo/ Foundation/ Association**	NA
Year of Establishment	1990
Registration Certificate;	Attach file
Recognition or Accreditation Status	
Scientific and Industrial Research Organization (SIRO) recognition by Government of India<br>WHO Collaborating Centre for Research, Community Based Action and Programme Development in Child Health	
Area of activities	

The Society of Applied Studies (SAS), New Delhi focuses on prevention of diseases, deficiencies and disabilities of public health significance with special emphasis on newborns, children and mothers through large intervention trials, impact assessment of interventions, explanatory research around proof of concept and policy research; in community-based settings.
The future priority of the group is to use an interdisciplinary and intersectoral approach through strategic collaborations and partnerships to achieve its vision of 'Improved newborn, child and maternal health, and nutrition in India through high quality, population-based research leading to development of effective interventions and design of novel delivery strategies.

The priorities and core programs of SAS are placed in the following domains:

WOMEN AND CHILDREN

- **Health and nutrition:** Research in this domain is dedicated to understand the determinants of impaired growth and development in children, the cross talk between nutrition and infection and reasons for slow progress in reducing rates of stunting in India. SAS has developed and evaluated multiple interventions to elucidate their clinical, biological and biochemical effects in the continuum from pre-conception, different phases of pregnancy and childhood. The drivers of impairment are common as well as unique at different time points in the continuum. Additionally, we aim to elucidate determinants of survival and morbidity, with major emphasis on development of interventions for reducing child mortality and promoting growth and development.
- **Mental health and cognitive science:** The research conducted primarily aims at understanding ways to accelerate development in children as well as addresses mental health issues in mothers, young children, and adolescents. In this domain newer tools to measure neurodevelopment in children such as eye tracking assessment and global scale for early development (GSED) are also being used.

INFECTIOUS DISEASES

The focus of research is to generate population-based disease burden of major infectious diseases through surveillance, nutrition-infection cross talk and clinical evaluation of vaccines (phase II to phase IV trials).

- **Evaluation of vaccines:** Conducted phase I to III clinical trials of the indigenously manufactured ROTAVAC® vaccine which is licensed and introduced into national program by Government of India in March 2016. We also examined the risk of intussusception after ROTAVAC® vaccination among Indian infants during pilot rollout in the public health system in three states: Himachal Pradesh, Maharashtra and Tamil Nadu. The other studies include predictors of poor immune response to Rotavirus Vaccine in Infant, clinical development of ROTASIIL®, **e**valuating the immunogenicity, safety and tolerability of a 10-valent Pneumococcal Conjugate Vaccine in healthy Indian infants in a phase III, multicentre, randomized, double-blind controlled trial, study on the Serum Institute of India's Meningococcal ACYWX Conjugate Vaccine (NmCV-5) and Serum Institute of India's HEXASIIL (DTwP–HepB-IPV-Hib) Vaccine
- **Epidemiology and surveillance:** Studies **i**n this domain include disease burden of Rotavirus diarrhea, he effects of human intestinal microbiota on immune responses, national surveillance system for enteric fever in India; establishment of a new demographic and health surveillance site in an urban resettlement colony in Delhi to study disease-specific epidemiology and conduct future vaccine trials, optimizing place of treatment and antibiotic regimens for young infants presenting with signs of Possible Serious Bacterial Infection

KNOWLEDGE INTEGRATION AND ECONOMIC ANALYSIS:
Knowledge integration uses one or more of the following methods: review of available evidence, program evaluation reports, implementation research outputs, secondary analysis of data from large nationally representative surveys, intervention trials or cohorts, and reports of consultative groups.
Economic evaluation is the comparative analysis of alternative courses of action in terms of both their costs and consequences. It involves two main areas, first, the costs and consequences of programmes or interventions and, second, choices which have to be made in allocation of resources. The purpose is to identify the best course of action, based on the evidence available.
The Knowledge Integration and Translational Platform (KnIT) was launched in 2016 with support from Biotechnology Industry Research Assistance Council, Dept. of Biotechnology and the Bill and Melinda Gates Foundation. It was conceptualized for providing evidence and experience-based guidance on how to accelerate progress, equity, impact in maternal and child health and nutrition. The platform aims to collate and analyzing available evidence within India, to inform policymakers and health authorities and aid in the development of evidence-based policy.
The overall interest is in the domain of maternal and child nutrition with specific interest in thriving of children for attainment of their full potential for growth and development. The priority areas have been chosen with the first 1000 days of life in mind, starting with antenatal period and till 2 years of age. The current priority areas include antenatal care, growth and nutrition in early life, low birth weight, anemia.

ONGOING RESEARCH PROJECTS INCLUDE:
MATERNAL AND CHILD NUTRITION
- Prevention and management of anemia: Effect of daily supplementation with multiple micronutrients (erythropoiesis-relevant) and IFA compared to IFA alone in children aged 6 to 59 months on haemoglobin concentration and treatment of mild to moderate anemia
- Evaluation of the impact of nutritional supplementation plus intensive breastfeeding support compared with intensive breastfeeding support alone on mortality, morbidity and growth in infants aged 0-6 months with growth faltering in low resource settings

(**Br**e**a**stfeedi**n**g **C**ounselling and Management of Growt**h** in early infancy – BRANCH)
- Impact of an integrated intervention package during preconception, pregnancy, and early childhood on biomarkers of infant growth in the first 6 months of life: A sub study in WINGS
- Impact of an integrated health, nutrition and early child stimulation and responsive care intervention package delivered to preterm or term small for gestational age babies during infancy on growth and neurodevelopment (Small babies Trial).

MENTAL HEALTH AND COGNITIVE SCIENCES
- Effect of improved dietary quality and quality of proteins in complementary food on cognition at 24 months of age
- Neurodevelopmental outcomes at 4-6 years in children with low birth weight who received kangaroo mother care during neonatal period: Early Career Fellowship application (Wellcome Trust/DBT India Alliance); starting in September 2020
- Impact of an integrated health, nutrition and early child stimulation and responsive care intervention package delivered to preterm or term small for gestational age babies during infancy on growth and neurodevelopment
- Use the Global Scale for Early development (GSED) within the women and infants integrated growth study (Wings) for Impact Evaluation on Neurodevelopment.
- Use of automated eye tracking assessment in children to predict their neurodevelopment outcomes at 24 months

REPRODUCTIVE, MATERNAL, NEWBORN AND CHILD HEALTH
- Antenatal, Intrapartum and Postnatal Care: A Prospective, Longitudinal Study of Maternal and Newborn Health: The Antenatal and Postnatal Care Research Collaborative (ARC) using harmonized methods across study sites to estimate the burden of maternal, fetal and neonatal deaths and select maternal and newborn morbidities in the catchment areas to understand the primary factors influencing pregnancy risk in LMICs.
- Reproductive health research
  Laboratory - confirmed reproductive tract infections in symptomatic and asymptomatic women
  Infertility - experiences and actions taken by women
  Postpartum contraceptive practices among couples
- Implementation research to promote treatment of pneumonia in under-five children to achieve high population-based coverage.

INFECTIOUS DISEASES, EPIDEMIOLOGY AND VACCINE TRIALS
- Microbiota-directed complementary foods to treat children with moderate acute malnutrition in the first two years of life – Open label Randomized controlled Pre-proof of Concept (MDCF prePOC) study
- Effect of community initiated Kangaroo mother care in low birth weight infants on infant breast milk intake and gut microbiome
- Establishment of a new Demographic and Health Surveillance Site in an urban resettlement colony in Delhi with the aim to study disease-specific epidemiology and conduct future vaccine trials
- Acute Diarrheal Surveillance and Molecular Testing for Shigella in India
- Optimizing place of treatment and antibiotic regimens for young infants presenting with signs of possible serious bacterial infection

SAS has been awarded a four year grant by the Bill and Melinda Gates Foundation for setting up a platform of institutions under the guidance of a strong technical Advisory Committee for promoting improved, innovative, impactful research on maternal and child health and development. The platform enables the high quality sites in India to come together as a network, harmonize their protocols, and collaborate both internally and globally to advance the Healthy Birth Growth and Development agenda.	
**Collaborators Particulars and Details of the Project Implementation Site**	
**Name of The Organization: Government of Himachal Pradesh**	
**Name of Project Coordinator: Ms M. Sudha Devi (IAS) - Secretary Heath**	
**Address1: Armsdale Building, HP Secretariat**	
**Street/Village: Chota Shimla**	
**City/Town: Shimla**	**Zip Pin code: 171002**
**State: Himachal Pradesh**	**Country: India**
**Fax:**	**Landline/Office Phone/ Mobile: 0177 2621902**
**Email Address: healthsecy-hp@nic.in**	**Website:**
**Brief Background of the Collaborators Organisation**	
**For Institution/ Universities/ Public Research Organization**	
**Year of Establishment of the Institution**	
**Recognition or Accreditation Status**	
**R&D Activities (Area)**	
**Name of The Organization: Government of Himachal Pradesh**	
**Name of Project Coordinator: Sh Ashish Singhmar (IAS) - Social Justice & Empowerment**	
**Address1: HP Secretariat**	
**Street/Village: Chotta Shimla**	
**City/Town: Shimla**	**Zip Pin code: 171002**
**State: Himachal Pradesh**	**Country: India**
**Fax:**	**Landline/Office Phone/ Mobile: 0177 2620625**
**Email Address: socialjesecy-hp@nic.in**	**Website:**
**Brief Background of the Collaborators Organisation**	
**For Institution/ Universities/ Public Research Organization**	
**Year of Establishment of the Institution**	
**Recognition or Accreditation Status**	
**R&D Activities (Area)**	
**Name of The Organization: National Health Mission**	
**Name of Project Coordinator: Ms Priyanka Verma (IAS)-Mission Director**	
**Address1: SDA Complex**	
**Street/Village: Kasumapti**	
**City/Town: Shimla**	**Zip Pin code: 171009**
**State: Himachal Pradesh**	**Country: India**

**Fax:**	**Landline/Office Phone/ Mobile:** 0177 2624505
**Email Address: md-hp-nrhm@nic.in**	**Website: https://nhm.hp.gov.in/**
**Brief Background of the Collaborators Organisation**	
**For Institution/ Universities/ Public Research Organization**	**Not applicable**
**Year of Establishment of the Institution**	**NA**
Recognition or Accreditation Status	**NA**
R&D Activities (Area)	**NA**
Source of Core Funding	**NA**
**Name of The Organization: Directorate of Health Services**	
**Name of Project Coordinator: Dr Gopal Beri- Director Health Services**	
**Address1: SDA Complex**	
**Street/Village: Kasumapti**	
**City/Town: Shimla**	**Zip Pin code: 171009**
**State: Himachal Pradesh**	**Country: India**
**Fax:**	**Landline/Office Phone/ Mobile:** 0177 2624505
**Email Address: dirhealthdhs@gmail.com**	**Website:**
**Brief Background of the Collaborators Organisation**	
**For Institution/ Universities/ Public Research Organization**	**Not applicable**
**Year of Establishment of the Institution**	**NA**
Recognition or Accreditation Status	**NA**
R&D Activities (Area)	**NA**
Source of Core Funding	**NA**
**Name of The Organization: Directorate of Women & Child Development**	
**Name of Project Coordinator: Ms Rupali Thakur (IAS) - Director of Women & Child Development**	
**Address1: Tutikandi, Shimla, Himachal Pradesh 171004**	
**Street/Village: Shimla**	
**City/Town: Shimla**	**Zip Pin code: 171001**
**State: Himachal Pradesh**	**Country: India**
**Fax:**	**Landline/Office Phone/ Mobile:** 0177-2622033
**Email Address: dirhealthdhs@gmail.com**	**Website:**
**Brief Background of the Collaborators Organisation**	
**For Institution/ Universities/ Public Research Organization**	**Not applicable**
**Year of Establishment of the Institution**	**NA**
Recognition or Accreditation Status	**NA**
R&D Activities (Area)	**NA**

Source of Core Funding	**NA**
**Name of The Organization:** ***State Implementation Unit*** **(State Institute of Health & Family Welfare)**	
**Name of Project Coordinator: Dr H R Thakur- Principal State Institute of Health & Family Welfare**	
**Address1: Parimahal**	
**Street/Village: Kasumapti**	
**City/Town: Shimla**	**Zip Pin code: 171009**
**State: Himachal Pradesh**	**Country: India**
**Fax:**	**Landline/Office Phone/ Mobile:** 0177-2620226
**Email Address: dirhealthdhs@gmail.com**	**Website:**
**Brief Background of the Collaborators Organisation**	
**For Institution/ Universities/ Public Research Organization**	**Not applicable**
**Year of Establishment of the Institution**	**NA**
Recognition or Accreditation Status	**NA**
R&D Activities (Area)	**NA**
Source of Core Funding	**NA**

PROPOSAL TECHNICAL DETAILS

Executive summary:

Provide a **concise** statement about the primary objective of the proposal, including the research question(s) the program will address, the impact the project will achieve, and the specific innovation(s) being proposed.

Adequate growth and development of the child lays the foundation of adult health and productivity and is an integral step towards achieving the Sustainable Development Goals (SDGs). Birth weight, gestational age and size at birth are key parameters influencing growth and development in early life. Low birth weight (LBW) is a predictor of linear growth in early childhood and an important risk factor for stunting, poor cognitive development and mortality. Most of the existing evidence reveals that standalone interventions within the first 1000 days window in the domains of health, nutrition, WaSH and psychosocial health, had modest effects on linear growth.

The Lancet, 2013 nutrition series has identified maternal undernutrition during pregnancy as a major determinant of poor fetal growth and child stunting. Women with a height <145 cm or BMI <18.5 kg/m^2 during early pregnancy are at greater risk for delivering a small for gestational age (SGA) infant. Furthermore, SGA, a marker for fetal growth restriction, is associated with an increased risk of child morbidity and mortality. Stunting is in turn associated with increased risk of child morbidity and mortality, poor cognition, lower school performance and human capital measures such as decreased earning potential, adult stature and increased risk of chronic disease later in life. Given the short and long term consequences of linear growth retardation during the first 1000 days of life, the prevention of stunting is a key global priority.

The Society for Applied Studies (SAS) recently conducted an individual randomized controlled trial (WINGS – The Women and Infants Integrated Interventions for Growth Study) (WINGS protocol paper; Trials. 2020 Jan 31;21(1):127 and WINGS main study paper; BMJ. 2022 Oct 26;379:e072046). The study provides evidence of impact on birth outcomes (preterm birth, LBW and SGA), growth (stunting at 24 months of age) and development in children, by intervening before women become pregnant, during pregnancy and postnatal life. through concurrent delivery of an integrated package of evidence-based interventions covering the continuum from the pre- and periconception period to early childhood. The study showed that LBW and stunting at 24 months were substantially reduced. Pregnancy and early childhood interventions alone had lower but important effects on birth outcomes and 24-month outcomes. Preconception interventions alone had an important effect on birth outcomes but not on 24-month outcomes.

BACKGROUND

Key achievement from the earlier phase

WINGS Study summary:

The study was conducted in low- to lower-middle income neighborhoods of Delhi, India. Married women aged 18-30 years, with no or one child were identified through a survey. Those living in temporary housing and moving away were excluded. Written informed consent was obtained for participation. Enrolled women were randomized to receive either preconception interventions or to routine care (first randomization). Women were followed up until pregnant, or up to 18 months post-enrolment. After ultrasonographic confirmation of pregnancy, women were randomized (second randomization) to either pregnancy and early childhood interventions or to routine care.

The primary outcomes were low birth weight, small for gestational age, preterm, and mean birth weight. At 24 months, the outcomes were mean length-for-age z scores and proportion stunted. Three prespecified comparisons were made: preconception intervention groups (A+B) versus no preconception intervention groups (C+D); pregnancy and early childhood intervention groups (A+C) versus routine care during pregnancy and early childhood (B+D) and preconception, pregnancy, and early childhood interventions groups (A) versus control group (D).

The proportion with low birth weight was lower in the preconception intervention groups (506/2235) than in the no preconception intervention groups (502/1889; incidence rate ratio 0.85, 98.3% confidence interval 0.75 to 0.97; absolute risk reduction −3.80%, 98.3% confidence interval −6.99% to −0.60%). The proportion with low birth weight was lower in the pregnancy intervention groups (502/2096) than in the no pregnancy intervention groups (506/2028) but the upper limit of the confidence interval crossed null effect (0.87, 0.76 to 1.01; −1.71%, −4.96% to 1.54%). There was a larger effect on proportion with low birth weight in the group that received interventions in the preconception and pregnancy periods (267/1141) compared with the control group (267/934; 0.76, 0.62 to 0.91; −5.59%, −10.32% to −0.85%). The proportion stunted at 24 months of age was substantially lower in the pregnancy and early childhood intervention groups (79/746) compared with the groups that did not receive these interventions (136/710; 0.51, 0.38 to 0.70; −8.32%, −12.31% to −4.32%), and in the group that received preconception, pregnancy, and early childhood interventions (47/453) compared with the

control group (51/271; 0.49, 0.32 to 0.75; −7.98%, −14.24% to −1.71%). No effect on stunting at 24 months was observed in the preconception intervention groups (132/892) compared with the no preconception intervention groups (83/564).
The study provides evidence of the impact of an intervention package delivered during preconception, pregnancy, and early childhood, on substantial reduction in low birth weight and stunting at 24 months. Pregnancy and early childhood interventions alone had lower but important effects on birth outcomes and 24 month outcomes. Preconception interventions alone had an important effect on birth outcomes but not on 24 month outcomes.

Based on the consensus of the government representatives, NITI Aayog team and the research team at SAS, Una district has been selected as the study district to implement the scale up WINGS integrated interventions. Una is an average performing district and accessible to the research team at SAS who will need to provide support and closely monitor the implementation progress.
The study will be conducted in 3 phases,
1. Initial preparatory phase (formative research)- 6 months
2. Model optimization and implementation phase and monitoring – 2 years
3. Data analysis and preparation of final report- 6 months

Una district has 5 blocks. One of the blocks will be selected as the learning block or the first block where the activities will be initiated. The model of implementation strategies will be optimized through iterative cycles of concurrent implementation, close monitoring and refinement based on reflections from the qualitative and quantitative data that will be collected every 3 months. During this phase of model optimization in the first block, the research team will build the capacity of the government personnel in the block so that the activities can be independently implemented and sustained in the absence of the research teams. This will also facilitate the government personnel to implement in other blocks and districts of the state. Once the optimized model is developed in the first block, the model will be implemented concurrently in the remaining 4 blocks with support from the research team at SAS and further adapted as per the context and requirements of each block. The research teams supporting implementation will exit from the blocks, based on predefined coverage and process indicators that will be finalized with the government, NITI Aayog and the TAG.
It is anticipated that the period of model optimization of implementation strategies and having the implementation of the interventions in place, will take around 9 months.
Prior to initiation of the implementation phase in each block, 3 rounds of baseline surveys will be conducted. After the intervention implementation is in place, 3 rounds of post-intervention surveys will be conducted in each block. During the implementation phase in each block, 3 monthly monitoring will be conducted. The detailed methodology is described later.

2. Project Plan:

1. Describe the proposed approach, including design and methods, for the project.
2. Clearly articulate the plan to achieve both short- and long-term outcomes, including a path to scale.
3. Highlight areas, scientific basis and potential of innovation in the proposed approach.

STRATEGIC GOAL OF THE INVESTMENT

Healthy women and healthy children through integrated delivery of health, nutrition, psychosocial care and WASH interventions during preconception period, pregnancy, postnatal and early childhood.

The overall aim of this investment is to develop a framework and guide the government partners with a clean action plan to scale up the WINGS integrated interventions in a selected district of Himachal Pradesh, that will be sustainable, scalable, and achieve high population-based coverage with quality.
The study will be conducted with deep government engagement and in close collaboration with NITI Aayog and ICMR. The government will be responsible for implementing the interventions. SAS will provide technical and implementation support to the government using the principles of implementation research.
Primary results that the project aims to achieve:
Improve population level predefined outcomes in Una district of Himachal Pradesh, which is the district selected for the study, through implementation of the WINGS integrated interventions in the four different domains, health, nutrition, WaSH and psychosocial support, delivered concurrently. Close periodic monitoring of the implementation strategies will be done within the existing health systems.

Primary Outcomes:

1. Health systems strengthening and streamlining of processes, by identifying existing gaps and implementing solutions for improved health of mothers and infants through Innovative and Sustainable approaches.
2. Enhanced commitment and resources for improving health of mothers and infants through evidence-informed advocacy.

Secondary outcome:

To estimate the incremental cost of implementing WINGS interventions within the government health systems

Outcomes

Outcomes measured through 3 monthly monitoring during the implementation phase and 3 rounds of post intervention surveys.

1. **Preconception wo**men defined as married women in reproductive age group who have not completed their families.
    - Proportion of women who are non-anemic.
    - Proportion of women with BMI between 18.5 to 24.99 kg/m2
2. **Pregnant women**
    - Proportion of women who are non-anemic.
    - Proportion of women with adequate gestational weight gain
    - Proportion of women with normal thyroid levels in pregnancy
3. **Infants**
    - Proportion of infants with adequate weight gain from birth till 2 years of age

Additionally, we will monitor **coverage indicators** and **process indicators**, through 3 monthly monitoring during the implementation phase and 3 rounds of surveys post intervention.

Preconception period
- anemic women who consumed Iron Folic Acid (IFA), multiple micronutrients (MMS)
- women with low BMI and inadequate weight gain (IWG) who received snacks, egg/milk as recommended.
- women who were screened and managed for health conditions (hypertension, RTI, diabetes, hypothyroidism)

Pregnancy
- women registering for ANC in the 1st trimester.
- women with at least 4 ANC visits and women with 8 ANC visits
- women with high risk pregnancies identified
- women who consumed IFA, albendazole and MMS as recommended
- women who were screened and managed for health conditions (hypertension, RTI, diabetes, hypothyroidism
- women with low BMI in the first trimester and inadequate GWG who received snacks (egg/milk) as recommended.

Postnatal
- mothers receiving and consuming nutritional supplements (medicines and food)
- babies who received at least 6 HBNC visits by ASHAs

Infants
- Infants 0-6 months exclusively breastfed (EBF), received kangaroo mother care (KMC) as applicable
- Infants received IFA, as recommended

Activities to create awareness and generate demand
- Social Behavioral Change Communication (SBCC) activities conducted as planned
- Families exposed to SBCC activities

Psychosocial and WaSH interventions
- Eligible women who received appropriate psychosocial support
- Eligible women and infants who received WaSH interventions as recommended

Definitions:

1. Preconception women are defined as married women in reproductive age group who have not completed their families.
2. Inadequate weight gain in preconception women is defined as less than 500gms per month in women with low BMI.
3. Inadequate weight gain in pregnancy at each trimester of pregnancy according to the BMI categories as defined by the Institute of Medicine Guidelines [Pregnancy weight gain of 12.5 to 18kgs in women with BMI <18.5 kg/m2 ; 11.5 to 16kgs in women with BMI of 18.5 to 24.9 kg/m2; 7 to 11.5kgs in women with BMI of 25.0 to 29.9 kg/m2 and weight gain of 5 to 9kgs in women with BMI ≥ 30.0 kg/m2.

UNA AT GLANCE

District Una lies in the South-Western part of Himachal Pradesh bounded by Kangra in the North and north-eastern, Hamirpur district in East, Bilaspur district in South East and Punjab state in West and South.

District Una has a population of **5,21,173** of which males and females are **2,63,692** and **2,57,481** respectively as per Census, 2011. There are five Health and Administrative Blocks in District Una (Una/Basdehra, Amb, Gagret, Dhundla/ Thanakalan & Haroli).

There are a total **1364** Anganwadi centres, **138** Heath & Wellness sub-centres **24** Primary Health centres, **9** Community Health Centres, **5** Civil Hospitals and **1** Regional Hospital

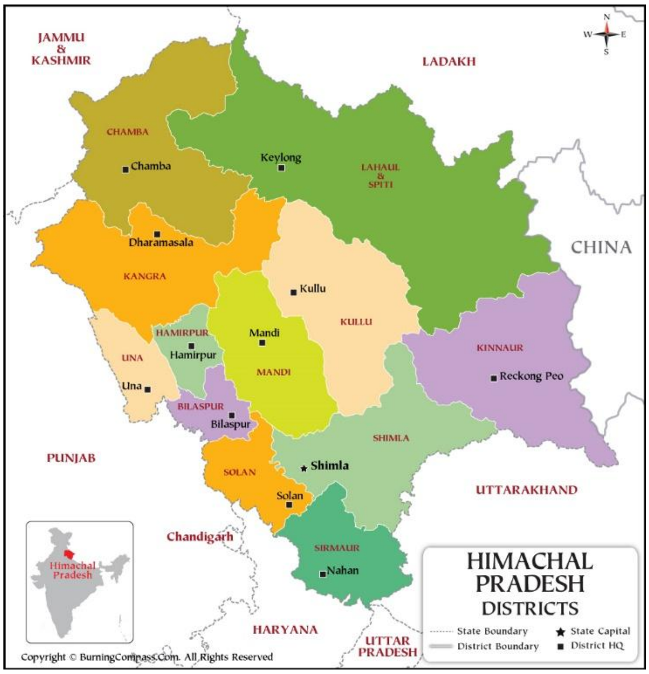

Map of Himachal Pradesh with districts

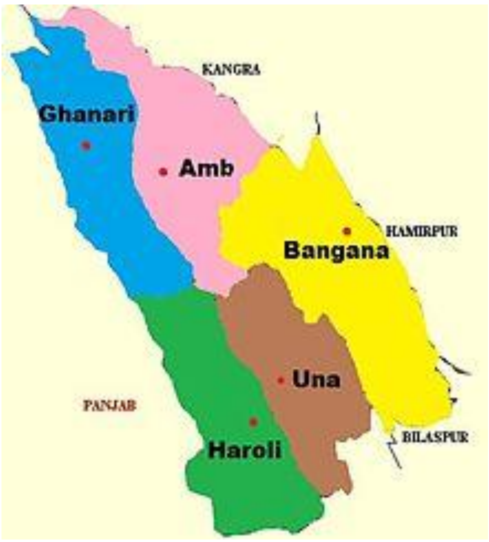

Map of district Una with 5

Present scenario/situation of health and nutritional indicators of women & children in Himachal Pradesh and Una district

The finding of the National Family Health Survey-5 (NFHS-5) reveals alarming stagnation or decline in critical health indicators, especially those pertaining to women and child health. This underscores the urgent need for focused interventions and increased resources to address these issues and improve the health outcomes of women and children. Some of the indicators of the NFHS-5 of Himachal Pradesh and District Una are depicted below:

S.N	Indicators	Himachal Data NFHS -5	District Una Data NFHS -5

1	**Population and Household Profile**		
	Sex Ratio at birth for children born in the last five years (females per 1000 males)	843	873
	Population living in households with an improved drinking water source	98.4%	98.8%
2	**Marriage and Fertility**		
	Percentage of women age 20-24 years got married before attaining the legal minimum age of 18 year as per NFHS -5	5.4%	1.6%
3	**Infant and Child Mortality Rates (per 1000 live births)**		
	The infant mortality rate before the age of one year per 1,000 live births	26	11 (District ICDS yearly report)
4	**Maternity and Child Health**		
	Mothers who had an antenatal Check-up in the first trimester	72.4%	70.7%
	Mothers who had at least four antenatal care visits.	70.3%	71.7%
	Mothers who consumed iron folic acid for 100 days or more when they were pregnant	67.2%	46.5%
	Mothers who consumed iron folic acid for 180 days or more when they were pregnant	43.0%	36%
	Mothers who received postnatal care from a doctor /nurse/LHV/ANM/Midwife/Others within two days of the birth.	86.3%	84.9%
5	**Delivery care**		
	Institutional Birth	88.2%	89%
	Institutional Birth at public facility	71.7%	52.2%
6	**Child Vaccinations and Vitamin A Supplementation**		
	Children aged 12-23 Months fully vaccinated based on information from either vaccination card or mother recall	89.3%	75.3%
	Children aged 12-23 months who have received BCG	98.2%	100%
	Children aged 12-23 months who have received 3 doses of Polio vaccine.	90.1%	77.6%
	Children aged 12-23 months who have received 3 doses of Penta or DPT vaccine.	96.1%	88.2%
	Children aged 9-59 months who have received a Vitamin- A dose In the last 6 months.	77.1%	79.1%
7	**Child feeding Practices and Nutritional Status of Children**		
	Children under age 6 months exclusively breastfed.	69.9%	91 %(HMIS)
	Breastfeeding Children age 6-23 months receiving an adequate diet.	18.00%	9.1%
	Total Children age 6-23 months receiving an adequate diet.	19.00%	8.8%
	Percentage of children under 5 years who are stunted is	30.8%	24.7%
	Percentage of children under 5 years who are underweight	25.5%	25.8%
	Percentage of children under 5 years who are wasted	17.4%	13.1%
	Percentage of children under 5 years who are severely wasted	6.9%	5.9%
	Percentage of children under 5 years who are overweight	5.7%	2.8%
8	**Nutritional status of Women age 15-49 years**		
	Women whose body Mass index (BMI) is below normal	13.9%	16.3%
	Women who are overweight or obese	30.4%	41.2%
	Women who have high risk waist to hip ratio	61.9%	58.2%

Gap Analysis

More than 30 per cent children under the age of five years in Himachal Pradesh are stunted and more than 1/4th are underweight according to the fifth National Family Health Survey-5. The health of children in the State has taken a turn for the worse in terms of all three crucial indicators of nutrition stunting, wasting and underweight. As per NFHS-5 data, the prevalence of anemia among children aged 5 to 59 months has increased from 53.7% to 55.4% compared to the previous NFHS survey.

The State of Himachal Pradesh needs to continue its investments in improving the coverage of interventions targeting pre/peri conception and the first 1000 days of life. It is also important to achieve progress in child birth interventions (institutional delivery and skilled birth attendance during delivery) to combat undernutrition and anemia effectively. Significant efforts are needed to strengthen the coverage of several pre and postnatal interventions, especially on reversing the declining trends in early initiation of breastfeeding, timely introduction of complementary feeding, and full immunization.

Project reach (as per the Baseline Survey of WCD and NHM Data)

S.N.	Category	Number
1.	Preconception period<br>(18-35 years of married women having no child and having one child)	19722
2.	Pregnant women	8253
3.	Postnatal (infants and children)<br>0-6 months<br>6 months to 2 years	 5105<br>12371
4.	Postnatal: mother till 6 months after delivery	8000

EXISTING INTERVENTION IN GOVERNMENT PROGRAMS AND WINGS INTERVENTIONS

S.N	Domain
1	Health
2	Nutrition
3	Psychosocial care and support
4	WaSH

The interventions are in four domains during the preconception, pregnancy, and early childhood (0-24 months) periods are as under. The detail interventions and strategies are described below

Preconception (18-35 years women) Target: 19722

a) Screening

Domain	Screening	Existing	Intervention through WINGS
Health	Medical Conditions known to affect foetal/infant growth; RTI/STI; TB, hypothyroidism, hypertension, diabetes	On-demand in an OPD basis	Women will be called for the Group meetings which will be organized at the nearest facility with the purpose of collection of Samples, and clinical examination. The Screening will be done annually except for ascertain signs/symptoms of RTI which will be done every 3 months. (2 meetings at AWC and 2 will be at HWC-SC, In all the meetings ASHA, AWW and other concern staff from WCD and Health should be present)
Nutrition	Screening of BMI	On-demand in an OPD basis	Women will be called for the Group meetings which will be organized at the nearest facility with the purpose of collection of Samples, and clinical

			examination. Collection of samples will be done annually, only if clinically indicated, samples will be collected every 3 months. The Screening will be done every 3 months. (2 meetings at AWC and 2 will be at HWC-SC)
Psychosocial Care	Screening of depressive symptoms	Not done	Screening will be conducted at the nearest facility using the Patient Health Questionnaire (PHQ-2), which is currently being used in facilities. This will be done every 3 months when conducting RTI screening.
WaSH		Swachh Bharat Program; Menstrual Health Management; hand washing	Counselling will be done in Group Meetings and during home visits to improve awareness regarding personal hygiene, hand washing, and safe drinking water and appropriate sanitation practices.

b) Prophylaxis and Treatment

Domain	Treatment	Existing	Intervention through WINGS
Health	Clinically identified with medical conditions like- Reproductive Tract Infection /Sexually Transmitted Disease, TB, diabetes, hypothyroidism, and hypertension.	Treated as per standard treatment guidelines at the nearest health facility.	• Ensuring **treatment and individual follow-up of cases.** For STI, the husband will also be treated. One dose can be observed in facilities.<br>• Providing contraceptive measures to delay pregnancy until women are infection-free, nutritionally-replete and in a positive state of mental health when they conceive.
	If clinically identified with anaemia	Treatment with IFA	Prophylactic doses will be given to non-anemic women once a week.<br>For women who are anemic, treatment with IFA (current preparation in the health systems of iron and folic acid) delivered by AWW & ASHA<br>- Prophylactic doses will be given for 15 days (Fortnightly Home visit).<br>- Observed intake will be done on the day of home visit.<br>- The rest of the day's intake photos will be uploaded on the digital app which will be verified by ASHA.<br>Vitamin B12 will be provided through multiple micronutrients having a higher dose of vitamin B12
Nutrition	Micronutrient supplementations	Not given	Multi micronutrients to be given for 15 days by AWW & ASHA. This is for prophylaxis and will be given thrice a week.<br>The rest of the day's intake photos will be uploaded on the digital app which will be verified by ASHA.

Nutrition WCD	BMI<18.5 kg/m2	Women in the age group of 18-35 years are not being covered.	Supplementary nutrition/food supplements will be provided to married women in the age group of 18-35 years having BMI less than 18.5 kg/m2.<br>- **BMI<16** : 1000kcal + 11.5-16gm protein as locally prepared snacks and egg/milk (70 kcal +6gm protein). Referred to hospital for screening and management.<br>- **BMI 16-18.49** :500 Kcal + 4.5 -7gm protein as locally prepared snacks and egg/milk (70 kcal +6gm protein). egg/milk<br>- Supplementary Calorie Nutrition and Protein Nutrition will be provided weekly by AWC with one compulsory observed intake during visit.<br>- Women will be weighed 3 monthly till recovery<br>- The rest of the day's intake photos will be uploaded on the digital app which will be verified by ASHA.
			Procurement of Dietary measuring scale at Anganwadi centres. (Supplementary nutrition/Food item measuring equipment)
			Procurement of storage containers for food material.
Psychosocial Care		Mental Health helpline: Tele MANAS along with 104 call centres	Counselling services on psychosocial care through trained healthcare providers (ASHA/CHO, Teleconsultation by Medical officers and Nai Disha Kendra (NDK) Counsellor)

Pregnant women screening target: 8253

a) Screening

Domain	Screening	Existing	Intervention through WINGS
Health	Complete blood count, Oral Glucose tolerance test, Blood Grouping, HIV, TSH, VDRL, Urine routine examination and blood pressure monitoring	ANC: 4 ANC	ANC: 8 ANC
		OGTT: 1 time	OGTT: 1 time
		Urine routine & microscopy: 2-3 times	Urine routine & microscopy: 4 times For asymptomatic bacteriuria, culture sensitivity will be done
		Hb: 1 time	Hb: 4 time during pregnancy i.e. at the time of registration, 20, 28, 34-35 weeks sufficient.

		CBC: none	At registration
Health	Screening of Thyroid disorder	Thyroid Stimulating Hormone test (only if required)	Only once at registration
Nutrition	For BMI, GWG	ANC	ANC: BMI in the first trimester; tracking of GWG at all ANC visits
Psychosocial Care	Screening of depressive symptoms	Not done	Screening using the Patients Health Questionnaire (PHQ-2 or local instrument currently in use) in each trimester health facility at ANC contact

b) Prophylaxis and Treatment

Domain	Treatment	Existing	Intervention through WINGS
Health	Clinically identified with medical conditions like-diabetes, hypothyroidism, hypertension.	Treated as per standard treatment guidelines at the nearest health facility	Ensuring treatment and individual follow-up of cases by CHO, AWW & ASHA (The rest of the day's intake photos will be uploaded on the digital app which will be verified by ASHA.)
Health	Prophylactic supplementation with Calcium (1000 mg) and vit D (400 IU)	During pregnancy and lactation	Strengthening, treatment by CHO, AWW & ASHA<br>- Prophylactic doses will be given for 15 days (Fortnightly Home visit).
Health	Antihelminth at 20 weeks.	During pregnancy after 1st trimester	To be given at 20th week of antenatal contact<br>The rest of the day's intake photos will be uploaded on the digital app which will be verified by ASHA.
Nutrition	Micronutrient supplements<br>IFA	Not given	Multi-micronutrient to all PW for 9 months daily for prevention of Nutrition deficiency<br>IFA/FA will be given daily based on the anaemia status<br>- The rest of the day's intake photos will be uploaded on the digital app which will be verified by ASHA.
Nutrition<br>WCD	Pregnant Women	Supplementary nutrition is provided to the pregnant women under the Supplementary Nutrition Programme viz 600 Kcal energy and protein 18-20 gms enrolled in the Anganwadi Centres	Supplementary nutrition/food supplements to all pregnant women in the District customised based on the gestational age and gestational weight gain.<br>- The rest of the day's intake photos will be uploaded on the digital app which will be verified by ASHA.

	PW with Inadequate Weight gain		Extra Provision of supplementary nutrition to pregnant women having inadequate gestational weight gain<br>- The rest of the day's intake photos will be uploaded on the digital app which will be verified by ASHA.
			AWWs to encourage the intake of food supplements.
Psychosocial Care		Mental Health Helpline: Tele MANAS along with 104 call centre	Counselling services through trained healthcare providers
WaSH		Swachh Bharat Program; hand washing	Counselling to improve awareness regarding personal hygiene, hand washing, safe drinking water practices, appropriate sanitation practices

Postnatal Mothers till 6 months: Screening Target: 8000

a) Screening

Domain	Screening	Existing	Intervention through WINGS
Health	Identification of Danger Signs in postnatal period till 42 days	Under HBNC:6 visits (Institutional Delivery) and 7 visits (home deliveries) within 42 days of delivery (3, 7, 14, 21, 28 and 42)	Empower family to identify danger signs and seek early care by ASHA & AWW.
			ASHA will be mapped to the medical officers/ specialist for home-based consultation
Psychosocial Care	Screening of depressive symptoms	Not done	Screening using Patients Health Questionnaire (PHQ-2) by AWW & ASHA during the HBNC on day 7, 28 42 and may be at 2 and 6 months for late onset

b) Treatment

Domain	Treatment	Existing	Intervention through WINGS
Nutrition (Health)	Nutritional supplements	Daily, 1 Iron and Folic Acid tablet for 180 days, post-partum	Provision of Multi micronutrient & Calcium/ IFA and Vitamin D<br>- Prophylactic doses will be given for 15 days (Fortnightly Home visit).<br>- The rest of the day's intake photos will be uploaded on the digital app which will be verified by ASHA.
		360 tablets of calcium in the first six months of the postnatal period (@ 2 tablets per day for 6 months).	
Nutrition (WCD)		Supplementary nutrition is provided to the lactating mothers under Supplementary Nutrition Programme viz 600 Kcal energy and protein 18-20 gms enrolled in the Anganwadi Centres.	Supplementary nutrition/food supplement to all lactating mothers in the District.<br>- The rest of the day's intake photos will be uploaded on the digital app which will be verified by ASHA.

WaSH		Swachh Bharat Program; hand washing	Counselling to improve awareness regarding personal hygiene, hand washing, safe drinking water practices, appropriate sanitation practices.<br>- The rest of the day's intake photos will be uploaded on the digital app which will be verified by ASHA.

Early Childhood (0-24 months): (0-6m Screening Target: 5105, 6-24m Screening Target:12371)

a) Screening

Domain	Screening	Existing	Intervention through WINGS
Health	Identification of Danger Signs of 0-24 months infant and child	**Under HBNC: 6 visits** (Institutional Delivery) and 7 visits (home deliveries) within 42 days of child-birth by ASHA.<br>Additional visits will be made for Low Birth Weight (LBW) or sick babies being discharged from SNCUs	Empower family to identify danger signs and seek early health care by AWW & ASHA.
		**Under HBYC: 5 visits** within 3 to 15 months of child-birth by ASHA	ASHA will be mapped to the specialist for home-based consultation.
		**Under ICDS: 9 visits** from 7months - 22 months after child-birth by AWW Weights will be measured at each visit to identify IWG	
Nutrition WCD	Child development	Measurement of height and weight of 0-2 years children in AWCs.	Measurement of height & weight of 0-2 years children every month as per the ICDS schedule
			Counselling on clean play area for he children in age group of 1-2 years.

b) Prophylaxis and Treatment

Domain	Treatment	Existing	Intervention through WINGS
Nutrition	For Nutrition support	Early initiation and exclusive breast feeding for 6 months	**0-6 months:** support exclusive breast feeding by rewarding mothers for their good practices that will achieve doubling of birth weight.
			**6-24 months**: support complementary feeding and continuous breast feeding by rewarding mothers for their good practices that will achieve tripling of birth weight.
Nutrition	For all 0-6 months & Low Birth Weight	Not given	**For all infants:**
			Vitamin D 400 IU daily up to 6 months
			Iron Supplementation: IFA (10 mg iron and 100 mcg folic acid) supplementation

			daily up to 24 months for VLBW from 2 weeks and LBW from 6 weeks as prescribe<br>- The rest of the day's intake photos will be uploaded on the digital app which will be verified by ASHA.
Nutrition	For preterm and Low Birth Weight	Family participatory care and KMC at health facility level, MCP card, ECD call center to focus on first 1000 days	Support kangaroo mother care **at home**
		Growth Monitoring through HBNC and HBYC visit	
Nutrition<br>WCD	Inadequate weight gain	Supplementary nutrition is provided to children in the age group of 6 months to 3 years & 3-6 years under Supplementary Nutrition Programme viz 500 Kcal energy and protein 12-15 gms	Supplementary nutrition/food supplement to be provided to all children ages 6 months to 1 year (2 years) and additional food to those with In-adequate Wight Gain.<br>- The rest of the day's intake photos will be uploaded on the digital app which will be verified by ASHA.
			1-2 years: Management of inadequate weight gain.
			Responsive feeding (By AWW)
Psychosocial care:<br>Promote early child play and responsive care<br>Identification of delayed development and timely referral.	Observation of the child development.	-Saksham Anganwadi & Poshan 2.0 **HBYC**<br><br>-MCP Card with ECD component<br>-Guiding Note for Early Childhood -Development **(ECD)** Call Center<br>-Operational Guidelines on ECD	As per the HBNC , HBYC visits and ICDS visits the delay in any development will be identified and timely referrals will be done by the ASHA and AWW.
WaSH:<br>Provide play mat and potty.<br>Continuation of wash interventions provided in pregnancy.	-	-	Counselling on clean play area for children in age group of 6months-2 years.<br>Hand hygiene, safe disposal of faeces, safe drinking water and personal hygiene during the contacts and home visits will be done by AWW and ASHA.

DETAIL STRATEGIES OF IMPLEMENTATION
PRECONCEPTION WOMEN: HEALTH
Screening and management in WINGS

Women were screened and treated for medical conditions known to affect foetal/infant growth; RTI/STI; TB, hypothyroidism, hypertension, diabetes. Women with severe anemia (Hb < 8 g/dl), prediabetes (HbA1c 5.7% to 6.4%), diabetes (HbA1c ≥6.5%), high blood pressure (≥140/90 mmHg; at least two measurements 48 h apart), hypo- (TSH > 5.5 IU/mL) or hyperthyroidism (TSH < 0.4 IU/mL), presence of symptoms of suspected TB, rapid plasma reagin positivity, reporting of symptoms of RTI or sexually transmitted diseases (STI), epilepsy and severe undernutrition (BMI < 16 kg/m2) were referred to the collaborating tertiary care

hospital. At enrolment and end of preconception, micronutrient assays were done- vitamins A, D, B12, Zn, Fe, Folate concentration and proportion deficient.

The intervention delivery team conducted the first visit post enrolment and three-monthly follow up visits thereafter for a period of 18m or till the women became pregnant. Weight (Salter 9509 weighing scale) and height (Seca-213 stadiometer) of women were measured to ascertain BMI. At the first visit, symptoms of RTI and tuberculosis (TB) were ascertained, and a history of epilepsy was taken. Blood pressure was measured (Omron 1300 digital blood pressure device). A blood specimen of 10 ml taken to check for anemia (hemoglobin - Hb), diabetes (Glycated hemoglobin - HbA1c), thyroid disorder (Thyroid-stimulating hormones - TSH), syphilis (Rapid plasma regain; RPR) and micronutrient assays. An outreach clinic manned by study physicians, nutritionists, psychologists and laboratory staff was set up in the study area. Women with mild to moderate anemia (Hb 8 to < 12 g/dL) were treated with iron-folic acid (100 mg elemental Fe, 1500mcg folic acid and 15mcg Vit B12) for three months or till they were nonanemic. Women with RTI or STI were managed in the outreach clinic or hospital using algorithms of the syndromic approach.

Family planning advice was offered to recently (< 1 year) married women, those with a young (< 1 year) child and if women had moderate to severe anemia, symptoms and signs of RTI/STI, undernutrition, hypertension, hypo- or hyperthyroidism and diabetes requiring treatment.

PRECONCEPTION WOMEN: NUTRITION

Screening of BMI, anemia: Prophylaxis, weekly Autrin (100mg elemental Fe, 1500mcg folic acid, 15mcg Vit B12) was administered and observed.

Mild/ moderate anemia: daily Autrin for 3 months, all not observed; Severe anemia referred.

Albendazole 400mg orally was given 6 mthly as a part of anemia prophylaxis to all.

Multiple micronutrients:Riconia Silver to all women thrice weekly.

Weight gain monitored in preconception women. Weight gain <500gm/month in women with low BMI was defined as IWG.

Intervention in WINGS based on BMI:- BMI<16 - 1000kcal + 12-15gm protein as locally prepared snacks and egg/milk (70 kcal +6gm protein). Referred to hospital for screening and management.

BMI 16-18.49- 500 Kcal + 6 -8gm protein as locally prepared snacks and egg/milk (70 kcal +6gm protein).

BMI 18.5- <21- egg/milk only

BMI and Hb were ascertained at enrolment. Study community workers (Sangini, similar to ASHAs) visited enrolled women at least once a week throughout the follow up period of 18 months or till women become pregnant. They counseled on study interventions, observed intake of supplements, when possible, replenished supplies and organized referrals to hospital and the outreach clinic, when necessary. “High risk” women i.e. those with moderate to severe anemia, hypo- and hyper-thyroidism, symptoms and signs of RTI/STI and undernutrition, were visited more often. These visits also provided women an opportunity to discuss their personal problems with the Sangini. Multiple micronutrients: All doses were not observed in WINGS. Riconia Silver to all women thrice weekly. Sanginis replenished the food to women at home. Eggs or milk were delivered 6 days a week through neighborhood depots managed by women residing in the study communities. These women visited enrolled participants and attempted to observe the intake of egg or milk. If the woman was not available, repeat visits were made. The intake by each woman was documented every day.

PRECONCEPTION WOMEN: PSYCHOSOCIAL CARE

Screening of depressive symptoms, use of tobacco, exposure to second hand smoking and alcohol use by spouse. Women were counselled using the adapted version of the module which emphasized the basic five principles: empathetic listening, guided discovery using pictures, family engagement, problem solving and behavioural activation, and was aligned to the local context which made it easy to comprehend by the participant. All women in the intervention group were counselled to promote generic problem-solving skills, inculcate positive thinking and empowered them in a way that they learnt to devise strategies, within their prevailing circumstances, to overcome day-to-day stressors.

Women were managed according to the severity of depressive symptoms. All women were counselled on positive thinking and problem-solving skills and against tobacco use (smoke and smokeless form) and on ways to reduce exposure to second-hand smoke. All women in the intervention group were counselled at home, using the module described earlier. Those with moderate depressive symptoms (PHQ-9 score 10–14) were counselled in the outreach clinic by a trained psychologist. For women with a score of 15 or more or those reporting suicidal thoughts, urgent referral to a psychiatrist was facilitated. A study team member approached the spouse at a convenient time and administered the AUDIT tool to assess severity of alcohol use. Referral was facilitated to a tertiary care hospital for those with a score of ≥20; in those with a score of < 20, counselling was done at home on ways to quit or reduce alcohol use.

PRECONCEPTION WOMEN: WaSH

Women were counselled on personal, menstrual and hand hygiene; safe drinking water and sanitation; done by the Sanginis during their home visits

STRATEGIES TO BE ADOPTED BY THE STATE IN IMPLEMENTATION RESEARCH FOR SCREENING AND MANAGEMENT

PRECONCEPTION WOMEN: 19722

Information to women, husbands and families:
At the outset women and their family members will be told about the project, the details of the program will be explained in the community meetings. SBCC activities will be conducted to create awareness about project using the existing platforms. Family support and support from husbands will be crucial.
Inclusion into the program:
Women who are registered as eligible couples in the RCH portal, aged 18-35 years are assumed to have fertility intention, will receive the preconception interventions. Couples who have undergone permanent sterilisation will not be included into the program.
Identification of preconception women:
ASHA eligible couple registers will be updated every month to strengthen identification of preconception women. Additionally, community awareness will be created to generate demand, so that women and families come forward to be a part of the program. All initial screenings to be completed in 1 month at each SC/AWC area.
Contact points with preconception women and coordination across health and ICDS:
Women will be told to visit the nearest facility (HWC/SC/PHC/CHC/DH/AWCs) where blood samples will be collected, history on symptoms will be taken, all measurements including anthropometry and blood pressure will be done, BMI will be calculated using an Android based Application installed into the mobile of ASHAs and AWWs, The MO or CHO will have desktop with the Application to calculate BMI. PHQ 2 to assess depressive symptoms (currently in use at health facilities) will be administered. Additionally, a 3 monthly drive will be conducted, 2 meetings at AWCs and 2 meetings at HWCs, where preconception women will be called to screen for BMI, RTI/STI, and other conditions.
Coordination between BMI identification at health facility and AWC to give the required supplement: For this, a day will be fixed (could be once a month) at the facility, the AWW, ASHAs will be available when women visit the facility, the information will be communicated.
Interventions to be delivered: Based on the BMI, the nutritional interventions will be given by the AWWs, Preventive management for all other conditions will be initiated, till the investigation reports are available. For non-anemic women prophylactic IFA and for all women, Multiple micronutrients will be started.
Women who are diagnosed to have any health problems based on their blood reports, will be called after 2 to 3 days, when reports will be available. For those who have normal reports, will be informed about the reports at home.
Nutrition supplement
For those between 16 to <18.5, 500 Kcal + 6 -8gm protein as locally prepared snacks and egg/milk (70 kcal +6gm protein.
For women with BMI <16: 1000kcal + 12-15gm protein as locally prepared snacks and egg/milk (70 kcal +6gm protein.
This will be panjeeri, premix, and at least 3 to 4 options, similar to WINGS. For women with BMI <16, double packets of the supplement will be provided, therefore separate packaging will not be required.
Women with BMI <16, will need to be referred to PHC/CHC/DH for assessment of morbidities.
MMN will be given thrice weekly and IFA as per the anemia status of the women.
Morbidity management: As per guidelines for adult management of cases
Depressive symptoms: by teleconsultation at HWCs; counselors at NDK (Nai Disha Kendra) clinics
WASH counseling and all other counseling**:** at the nearest facility when they visit every 3 months. AWWs and ASHAs through Jan Bhagidari will counsels the beneficiaries about menstrual and hand hygiene; safe drinking water and sanitation. Every month CBEs and VHSNDs are conducted at AWCs in which they counsel their beneficiaries about these practices. Further through Home Visits, POSHAN Maah, POSHAN Pakhwada and Breastfeeding week which are organized at AWC level, these platforms can be used for counselling of the beneficiaries and their family members.
The department of Jal Shakti will be told to keep a periodic check on the quality of drinking water, to prevent any diarrhea outbreak.
Strategies for replenishment and observation:
All medicine/IFA/MMN will be given every 15 days by ASHAs at home. On the day of visit, the ASHAs can observe intake of IFA and MMN.
The AWW will deliver supplementary food packets and egg/milk (tetra pack flavoured), once a week to the womens' home and observe intake of egg/milk on the day of visit. Rest of the days, observed intake of supplements and medicines, will be based on geotagging system with pictures of beneficiary while taking the Supplements with date and time. Photos of egg/milk intake will be uploaded on the application through phones of the women. These will be also uploaded by AWWs/ASHAs to supervisors; they need to have access to all links.
Follow up
All screening tests will be done annually. However, if the woman is anaemic or has abnormal thyroid level, treatment will be provided and repeat tests (Hb, TSH) will be done every 3 months. Women will be asked about symptoms of RTI/STI and screened if there are symptoms. If woman has high blood pressure, she will be treated and followed up every 3 months at the facility. Women will be called to facility every 3 months or even otherwise if they have any problems at any time point.

For those with BMI <18.5, repeat weight will be taken after 3 months to ascertain BMI.
Referral: All women with TB, severe anemia, BMI<16; epilepsy, suicidal ideation will be referred to the district hospital or medical college for further management.

Tracking of all parameters

An unique ID number will be given to women across preconception, pregnancy, birth and of the child till 2 years

Procurement plan by state: As per the DPO Una: Eggs or milk will be distributed among preconception women based on their preference. These items will be purchased at Circle level by the ICDS Supervisors and to minimize the transit costs and to maintain the shelf life & quality of these items, it is decided that these will be purchased by the local vendors by the ICDS Supervisors and can be distributed once in week or fortnightly.

Across all groups the IFA preparations by the government will continue, composition varies in the content of iron, folic acid and vitamin B12. The government supply of IFA has lower dose of iron and folic acid, but no vitamin B12. Therefore a multiple micronutrients with higher dose of vitamin B12 will be selected to compensate for the lack of the same in government supply of IFA.

SCREENING AND MANAGEMENT IN WINGS

PREGNANT WOMEN HEALTH

When a pregnancy was reported, trans-abdominal ultrasound (GE ultrasound Voluson S8, PI Healthcare Inc.,23865 Via Del Rio, Yorba Linda CA 92887, USA) was done between 9 and 13 weeks of gestation. Repeat ultrasounds were done at 26–28 weeks and 35–36 weeks of gestation for fetal growth. Around 10 ml blood was taken from women in when they reported pregnancy Complete blood count, Oral Glucose tolerance test, Blood Grouping, HIV, TSH, VDRL, Urine routine examination and culture, and blood pressure monitoring were done. CBC, OGTT and urine r/e& culture were repeated. Women were treated for morbidities. Women were screened & treated GDM, PIH, RTI/STI (VDRL, HIV), thyroid disorder and antihelminth was administered at 20 weeks. At 35 weeks gestation, micronutrient assays were done- vitamins A, D, B12, Zn, Fe, Folate concentration and proportion deficient.

Each woman was allocated to a worker in the intervention delivery team for pregnancy. This worker visited the women monthly and ensured registration in the collaborating hospital (if the woman did not want to go, she was encouraged to register in a hospital of her choice) for delivery, counselled on the importance of regular antenatal care, danger signs during pregnancy, on the benefits of an adequate diet during pregnancy and preparation for breastfeeding and infant care and promoted institutional delivery.

The workers encouraged the woman for timely antenatal care(1 visit in the first trimester, 2 in the second and 5 in the third trimester). They also ensured uninterrupted supplies of iron folic acid, calcium, vitamin D and multiple micronutrient supplements to the woman throughout pregnancy.

PREGNANT WOMEN NUTRITION

Screened for BMI, GWG. As micronutrient deficiency was high in this setting; pregnant women were advised ~ 1 RDA of daily micronutrient supplementation everyday throughout pregnancy. Daily oral IFA -Autrin and twice daily Calcium (500mg) +Vit D (400IU) supplements were given throughout pregnancy. To meet the additional energy andprotein requirements, food supplements were given to all women with BMI < 25 kg/m2. The consensus by the TAG was to estimate additional requirements assuming a 12 kg weight gain during pregnancy. The supplements were provided through a choice of locally-prepared snacks (containing cereal, pulses, soya, oil, sugar, salt, milk powder); 210 kcal, 2 g protein in second trimester;
400 kcal, 21 g protein in third trimester.

All women were also given milk (180 ml, 70 Kcal, 6 g protein)6 days a week throughout pregnancy.

Additionally, women with BMI < 18.5 kg/m2 were given 500 Kcal, 20 g protein in the form of a hot-cooked meal as the first meal in the morning. Weight gain was monitored monthly till 32 weeks and more frequently thereafter. Those with inadequate weight gain defined based on Institute of Medicine Guidelines got a hot cooked meal (500 Kcal, 20 g protein), 6 days a week. Milk was delivered to pregnant women through neighbourhood depots; an attempt was made to observe the intake and compliance was documented at each visit. Women were encouraged to consume snacks given to them. Monthly weight measurements were taken at home or at the outreach clinic.

PREGNANT WOMEN PSYCHOSOCIAL CARE

Women are screened for depressive symptoms using the PHQ-9 questionnaire four times during the pregnancy (once in the first trimester, twice in the second and once in the third trimester).

An adaptation of the WHO Thinking Healthy Module was used for counselling during pregnancy. The content covered three broad domains: personal health of the mother, her relationship with other family members and her relationship with the child.

Sanginis counselled pregnant women on positive thinking and problem solving skills using the adapted module. The use of tobacco by woman, exposure to second-hand smoke and alcohol use in the spouse was ascertained. The ensuing counselling was similar to that in the pre-conception period.

PHQ-9 score >= 10 referral was made to outreach clinic for a psychologist review and psychiatrist referral if needed. For women with a score of 15 or more or those reporting suicidal thoughts, urgent referral to a psychiatrist was facilitated.

PREGNANT WOMEN WaSH

The WASH interventions during pregnancy (and continued during postnatal period) included improvement of drinking water quality through provision of water filters and storage bottles; reducing fecal load in the environment by providing disinfectants for cleaning toilets (if not available at home), and promotion of handwashing to reduce fecal transmission by placing a handwashing station in households where these were not available, counselling on correct handwashing technique and timing and provision of soap for handwashing.

The study team delivered the disinfectants, soaps, installed handwashing stations, distributed water bottles to store water, and counselled on hygienic practices.

STRATEGIES TO BE ADOPTED BY THE STATE IN IMPLEMENTATION RESEARCH FOR SCREENING AND MANAGEMENT

PREGNANT WOMEN SCREENING TARGET: 8253

Identification of pregnant women:

ASHAs will maintain updated pregnancy surveillance RCH registers.

Screening and intervention delivery

The government will encourage all women for 8 ANCs (1 in first trimester, 2 in 2nd trimester and 5 in 3rd trimester).

USG will be done at 18 weeks at least once; if possible one more USG will be done in the second trimester if required or advised by the doctor. The government will strengthen USG facility. USG machines will be shifted to the SDH, 4 doctors are trained in 6 month course PCPNDT USG training, who will do the USGs.

There is provision of all blood investigations at all levels of facilities including the HWC**.** Blood samples will be collected at ANC visits and prophylaxis will be initiated. In case of any abnormal report, women will be called after 2 to 3 days.

Tracking of Hb levels and gestational weight gain will be done across ANC visits. The data on Hb, gestational weight and other parameters will be entered into the hardcopies of MCP card as well as the digital platform/RCH portal. Information on the parameters can be retrieved using the unique identification number at each ANC visit. MCP card will be maintained along with digital platform.

Food supplement: 210 kcal, 2 g protein in second trimester and 400 kcal, 21 g protein in third trimester will be provided. Different packaging will be done for the 2nd and 3rd trimester as was done in WINGS. The HP State Co-operative Milk Producer's Federation or any other agency will be involved in manufacture these products.

All women will also be given milk (180 ml, 70 Kcal, 6 g protein) 6 days a week throughout pregnancy.

Additionally, Pregnant women with inadequate weight gain will be given supplementary nutrition.

PHQ 2 or the local instrument being used, will be administered once at each ANC visit by facility-in-charge.

ASHAs will counsel on WaSH during the home visits

All AWCs conduct counselling sessions every month for handwashing practices, during CBEs and VHSNDs.

Coordination between health ANC clinic and ICDS: each woman will be mapped with AWW and ASHAs right from the preconception and will continue till child is 2 years old. WhatsApp group can be made with the ANMs, ASHAs and MO/CHO at facilities and the AWWs and their supervisors. Communications on the group will increase accountability. Dedicated group ANC days in the PHC/CHC/ SC/HWC/AWC, where ASHAs and AWWs will be available and review data of women and their requirement of nutritional supplement. The data will be entered in the newly designed App as well as RCH portal

Packaging of food supplements: separate for 2nd and 3rd trimester as well as for women with BMI <18.5.

Strategies for replenishment and observation:

All medicine/IFA/MMN will be given every 15 days by ASHAs at home. On the day of visit, the ASHAs can observe intake of IFA and MMN.

The AWW will deliver supplementary food packets and egg/milk (tetra pack flavoured), once a week to the women's home and observe intake of egg/milk on the day of visit. Rest of the days, observed intake of supplements and medicines, will be based on geotagging system with pictures of beneficiary while taking the Supplements with date and time. Photos of egg/milk intake will be uploaded on the application through phones of the women. These will be also uploaded by AWWs/ASHAs to supervisors; they need to have access to all links.

MANAGEMENT IN WINGS

POSTNATAL WOMEN HEALTH AND NUTRITION

Danger Signs were identified in postnatal period till 42 days. Mothers were encouraged to visit the delivery facility according to the follow up schedule advised by the facility. Post-birth, the Prerna workers conducted follow-up visits to the intervention group households. The first visit was made within 24 h of hospital discharge or birth in case of home delivery. Mothers were

encouraged to comply with postnatal visits advised by the hospital for themselves, encouraged to consume milk, iron-folic acid, calcium, vitamin D and multiple micronutrients for the first 6 months post-delivery. Women were provided locally-prepared snacks (500 kcal, 15 g protein) and milk (180 ml, 70 Kcal, 6 g protein) 6 days a week to meet the additional requirements during lactation. Home visits for all births were made by Prerna on days 3, 7, 10, 14, 28, and monthly from 2 to 24 months

POSTNATAL WOMEN PSYCHOSOCIAL SUPPORT

Screening of depressive symptoms was done. The intent was to decrease the risk of postpartum depression which had shown to negatively affect breastfeeding performance and long-term child growth and development. Counselling was done using a module developed through adaptation of the WHO Thinking Healthy Module

POSTNATAL WOMEN WaSH

Same interventions during pregnancy to continue

STRATEGIES TO BE ADOPTED BY THE STATE IN IMPLEMENTATION RESEARCH FOR SCREENING AND MANAGEMENT

POSTNATAL MOTHERS TILL 6 MONTHS: 6239

0-6 months: support exclusive breastfeeding by rewarding mothers for doubling of birth weight and 6-24 months, support complementary feeding and continuous breast feeding by rewarding mothers for tripling of birth weight. It should however be kept in mind that doubling of birth weight may not be appropriate for SGA babies (who constitute at least 25-30% of all births. The other option is to use the growth trends of the infants to show how breast feeding is being beneficial and also link with morbidities.

Food supplement, eggs/milk will be provided as for other beneficiaries as preconception and pregnant women.

All interventions will be delivered at home by ASHAs during the HBPNC visits till 42 days of child age, thereafter fortnightly by ASHAs.

AWWs will deliver the food supplements and egg/milk once a week.

During the home visits, both AWWs and ASHAs will observe intakes of medicines, egg/milk.

PHQ 2 or the local instrument being used currently, will be administered during the HBPNC visits by ASHAs on days 7, 28 42 and may be at 2 and 6 months for late onset ASHAs will counsel on WaSH during the home visits.

All AWCs conduct counselling sessions every month for handwashing practices, during CBEs and VHSNDs.

MANAGEMENT IN WINGS

EARLY CHILDHOOD HEALTH

Interventions included identification of danger Signs of 0-24 months infant and child; Empower family to identify danger signs and seek care early; counselling on timely immunisation and Albendazole starting 12 months 6 monthly. 5 ml blood was collected from children at 24 months of age in both groups for micronutrient assays.

Post-birth, the Prerna workers conducted follow-up visits to the intervention group households. The first visit was made within 24 hours of hospital discharge or birth in case of home delivery. Home visits for all births were made by Prerna on days 3, 7, 10, 14, 28, and monthly from 2 to 24 months. Additional visits were made for those born preterm, LBW and for mothers with breastfeeding problems. Exclusive breastfeeding was promoted for the first 6 months. If the mothers reported breastfeeding problems, visits by the study lactation counsellors were arranged.

Mothers were counselled to initiate complementary feeding at 6 months while continuing to breastfeed till 24 months of age. Recipes were shared on foods that could be prepared at home, along with ways to encourage the infant to eat.

Packets of milk-cereal mix were provided to all children. Intake of daily iron supplementation was encouraged

EARLY CHILDHOOD NUTRITION

For Nutrition support, from birth to 6 months of age, mothers were counselled on early initiation of and exclusive breastfeeding. Monthly growth monitoring was done for early identification of growth faltering. Those with inadequate weight gain (<15th centile as per WHO weight velocity/month) were referred to the outreach clinic for a physician examination for morbidity and for lactation counselling.

Caregivers of children aged 6 to 24 months were counselled on timely introduction of complementary foods at 6 months, on the frequency of feeding and types of food to be fed and their amounts, recipes of energy and nutrient-dense meals made from locally-available, culturally-acceptable foods were shared. Additionally, a daily milk cereal mix packet was given.

6 to 12 months; 125 Kcal per day, 2.5 g protein; **12 to 24 months**; 250 Kcal per day, 5 g protein that included 80% to 100% RDA of micronutrients) was provided. This covered 40% to 60% of the energy requirement between6 to 24 months of age assuming the child was breastfed. Monthly weighing was continued to detect growth Faltering. Those with inadequate weight gain (<25th centile according to WHO weight velocity/month) were referred to the physicians in the outreach clinic for assessment of morbidity. Additional food supplements in the form of snacks providing (~ 125 Kcal per day, ~ 2.5 g protein during 6 to 12 months; ~ 250 Kcal per day, ~ 5 g protein during 12 to 24 months) were offered

For all 0-6 months & Low Birth Weight: Micronutrient supplementation (calcium, iron, phosphorus, vitamin D) for LBW and very LBW infants was done according to WHO guidelines

For preterm and Low Birth Weight 0-6 month: lactation support for early and exclusive BF and expressed breast milk for preterm were provide

Management of Inadequate weight gain was done and kangaroo mother care supported at home.

Post-birth, the Prerna workers conducted follow up visits to the intervention group households. The first visit was made within 24 h of hospital discharge or birth in case of home delivery. Home visits for all births were made by Prerna on days 3, 7, 10, 14, 28, and monthly from 2 to 24 months. Additional visits were made for those born preterm, LBW and for mothers with breastfeeding problems. Exclusive breastfeeding was promoted for the first 6 months.

If the mothers reported breastfeeding problems, visits by the study lactation counsellors were arranged.

Mothers were counselled to initiate complementary feeding at 6 months while continuing to breastfeed till 24 months of age. Recipes were shared on foods that could be prepared at home, along with ways to encourage the infant to eat. Packets of milk-cereal mix were provided to all children. Intake of daily iron supplementation was encouraged.

Monthly weight and length measurements were taken by the Prerna. Infants (0 to 6months) with inadequate weight gain were visited by a lactation counsellor and referred to the physician at the outreach clinic if morbidity was present.

Children between 6 to 24months of age with inadequate weight gain (<25th centile according to WHO weight velocity/month) were referred to outreach clinic for examination by physicians. Additional locally-procured snacks or extra packets of foods were provided. Children with severe acute malnutrition (weight-for-length Z-score, WLZ < -3 SD) were managed at facilities.

Six-monthly deworming was given to all children from12 months of age. Families were counselled about timely immunization, taught to recognize danger signs and to seek care promptly when the infant was ill.

Vitamin D 400 IU daily for all infants up to 6 months; Iron Supplementation: VLBW from 2 weeks and LBW from 6 weeks

EARLY CHILDHOOD PSYCHOSOCIAL SUPPORT

Promotion of early child play and responsive care; Identification of delayed development and timely referral. Observation of the child development. Age-specific child play and stimulation activities, soon after birth, along with early identification of developmental deviations and their prompt management were the core components of the child-care package as brain growth is highly dynamic in the first two years of life and structured stimulation provided soon after birth by the mother and family members would accelerate development.

The interventions for early child development was adapted using Care for Child Development manual developed by WHO and UNICEF

The Prerna'scounselled mothers on positive thinking and problem solving skills. During these visits, the team also assessed the use of tobacco by woman, exposure to second-hand smoke and alcohol use in spouse. A brief PHQ 9 questionnaire was administered to women within weeks of delivery to assess her mood and screen for depression.

The Prerna demonstrated to the families ways to interact and communicate with their babies. They also assessed key developmental milestones at specified ages. Infants who do not attain age-specific milestones were referred to the paediatricians and psychologists.

An electronic monitoring system was developed to track women and children with problems and those who required additional support to achieve high compliance to interventions delivered during all the periods. All participants (women and children) in both the groups were free to access the usual care pathways including free services from the government health system.

EARLY CHILDHOOD WaSH

The pregnancy WASH interventions were continued in the postnatal period. Additional interventions were promotion of safe disposal of child faeces and providing a potty at ~1 year of age. To promote a clean area for children, a play mat was provided ~ 6 months of age.

The Prerna's counselled on WASH interventions as during pregnancy. Additionally, counselling was done on hand hygiene for child feeding, safe disposal of feces, use of diapers and their disposal, and use of clean play area

STRATEGIES TO BE ADOPTED BY THE STATE IN IMPLEMENTATION RESEARCH FOR SCREENING AND MANAGEMENT

EARLY CHILDHOOD (0-24 MONTHS): (0-6M SCREENING TARGET: 6239, 6-24M SCREENING TARGET:11735)

ASHAs will be trained in appropriate identification of danger signs and timely referral. They will empower family members to recognise danger signs and seek care. ASHAs will counsel and ensure timely immunization during HBPNC visits. ASHAs will also be trained in lactation and KMC. Additional visits will be made to support breastfeeding and KMC for preterm/LBW babies. For problems in lactation, mothers will be referred to the HWC or PHC for lactation support. The ANMs and CHOs will be trained in lactation support.

At present under SNP same nutritional norms are for 6 months to 3 years children, whereas, in WINGS scale up, supplementary nutrition for 6 to 12 months will be separate and will be milk or cereal based mix as done in WINGS study. Milk cereal based mix samples, composition of the premix supplements and recipes will be shared with the State by SAS. The state will procure these products through HP Milk Federation or any other agency.

Monthly weight monitoring will be done at AWCs from 6 months onwards. Weights will be plotted in the growth chart. From 0 to 6 months, the ASHAs will weigh the babies at home during the HBPNC visits and monthly thereafter. Infants with inadequate weight gain will be referred to the HWC/PHC/CHC for examination and rule out any morbidities. Heights of the children will be measured every 6 months.

Children with severe acute malnutrition (weight-for-length Z-score, WLZ < -3 SD) will be referred to NRC.

AWWs will support and counsel mothers and families on appropriate complementary feeding, in terms of quantity, quality, frequency, consistency and responsive feeding. The current ICDS SNP will be replaced with food supplements similar to that of WINGS, which is milk cereal mix in different flavours; providing 125 Kcal per day, 2.5 g protein for 6 to 12 months old infants and 250 Kcal per day, 5 g protein for 12 to 24 months old children. Those with inadequate weight gain (<25th centile according to WHO weight velocity/month) will be given additional food supplements (~ 125 Kcal per day, ~ 2.5 g protein during 6 to 12 months; ~ 250 Kcal per day, ~ 5 g protein during 12 to 24 months); i.e., double the milk cereal mix that is given regularly. The supplement needs to be fortified with multiple micronutrients.

6 monthly deworming will be done.

IFA will be given to all children above 6 months to 2 years (10mg Fe and 100 mcg folic acid). For LBW and preterm, it will be started at 6-8 weeks after birth and VLBW, from 2 weeks. Only iron will be given till 6 months for VLBW and LBW, following which all children were given IFA post 6 months

The cereal mix will be fortified with MMN for children >6 months old.

Vitamin D 400 IU daily for all infants up to 6 months of age. Preterm babies will be given calcium and vitamin D till 6 months of age, although this was not given in WINGS.

ASHAs will deliver the ECD component during home visit, for any developmental delay, the children will be referred to the nearest HWC/PHC/CHC/DH. ASHAs will be trained in early child development.

ASHAs and AWWs will counsel families on WaSH, hand hygiene for child feeding, safe disposal of feces, use of diapers and their disposal, and use of clean play area to reduce fecal load in the environment and fecal contamination. There is no need of play mats.

PROJECT IMPLEMENTATION PROCESS:

The interventions will be aligned with the existing government programs that are already being implemented in the state. However, some of the WINGS interventions are not in the government programs, particularly those in the preconception period. Prior to rolling these out in the Una district, a policy decision at the state government level has been made through initial discussions, for adopting additional interventions and innovative delivery strategies across all domains.

SAS will be responsible for supporting implementation in one district, Una, through close monitoring using the Consolidated framework of Implementation Research (CFIR) which is composed of five key domains, with multiple constructs related to each domain. The five key domains are characteristics of individuals involved; definition of each Intervention and characteristics; outer setting or broader context that refers to the economic, political, social, and cultural context which would influence implementation of the interventions; inner setting or health system context and the process of implementation**,** which is considered one of the most important domains of the framework, and focuses on engaging the relevant stakeholders, executing, reflecting, and evaluating with the aim of optimizing the implementation strategies. We will use the non-linear, recursive implementation process in which there will be multiple concurrent cycles of implementation, process learning (quantitative and qualitative feedback) and coverage/quality evaluation (quantitative feedback) with periodic meetings led by the government authorities to reflect on the implementation process, learnings gained, assess the model performance, and revisions to be made to the implementation model to continuously improve it. REAIM (reach, effectiveness, adoption, implementation and maintenance) framework for monitoring implementation and the COM-B model (capability, opportunity and motivation) for behavior change will be used. This model will be used to design the Information, Education and Communication (IEC) materials and counselling guides for intervention delivery. We will use the interrupted time series design to implement the interventions in all the 5 blocks of the study district to monitor the outcomes of the interventions.

Research teams

Three research teams will be involved- the Implementation Support Team (IST), Program Learning Team (PLT) and outcome monitoring team (OMT). The IST will handhold the government personnel in the various departments, supporting implementation, working closely with all stakeholders, in the initial phase. Later the support will be gradually withdrawn, when the government no longer needs the same. The IST will subsequently take up an advisory role and their expertise may be used by the government for scaling up in other districts. The PLT, which is primarily a qualitative team, will conduct activities aimed to assess the fidelity and adherence to program implementation, whether the interventions

are being implemented and utilized as desired, will do a root cause analysis to identify the barriers and facilitators to effective implementation and inform the IST. The IST will take prompt and corrective action with deep government engagement, encouraging the government to come up with feasible pragmatic solutions, which will be implemented subsequently. The PLT will additionally monitor process indicators. The third team is the outcome monitoring team that will monitor the predefined outcomes and coverage indicators through 3 monthly monitoring during the implementation phase and 3 rounds of surveys each in the pre-intervention and post intervention periods.
The data on outcome indicators will be collected at population level.
The most relevant coverage and process indicators that are likely to impact the outcome indicators, will be selected in discussion with the government and the TAG, to take a decision of the time point of exit of the IST and PLT, from the blocks in the district.

Preparatory phase
High-level meetings with the state authorities, NHM (maternal, child and mental health, and community processes divisions), Department of Women and Child Development (WCD), Psychology department of HP university, Department of Jal Shakti, will be conducted. The meetings will be to discuss the status of implementation of the interventions that are already in the government programs. The discussions will additionally focus on the proposed plan and way forward with implementing the interventions that are not a part of the government programs. A series of meetings with the local government, at the district and block levels will follow; a list issues and anticipated implementation challenges will be prepared. Solutions will be discussed for the mandatory prerequisites to initiate implementation of WINGS interventions. These will include the human resources, infrastructure, transportation, equipment, supplies and supervision within the government health systems. It may not be feasible to resolve all issues at the outset. However, a concrete plan with timelines on the way forward will be developed and followed up.

During these meetings, the following specific issues will be discussed.

- Review existing information and processes, identify gaps.
- Staff recruitment plan to fill vacancies, retention of human resources.
- Streamlining of procurement systems.
- Plan to implement WINGS interventions not in the national programs.
    - the food supplement for preconception women through the WCD
    - additional food supplement for pregnant women with low BMI and inadequate weight gain
    - Provision of animal protein in the supplementary food- feasibility
    - screening and management of health conditions of preconception women
    - additional screening of pregnant women
    - procurement of supplies such as multiple micronutrients
    - provision of laboratory and ultrasound facilities within the government system or private outsourcing
    - referral facilities and processes, etc.
- Availability of funds: Scope of mobilizing existing government resources through reallocation of government funds or use of untied funds, whether there is a need for additional external financial support and the time needed to arrange the funds through Project Implementation Plan (PIP) at the state level.
- Capacity building for sustainability: Identify the preferred local technical institutions to support sustain capacity building.
- IT support to strengthen HMIS, for data driven actions, development of trackers and integrating coverage and quality indicators with the HMIS. (Development of IT application for the purpose of study intervention)
- Reallocation of responsibilities of existing staff to lead and actively participate in coordinating WINGS implementation.

Additionally, during the preparatory phase, research staff for all the 5 blocks will be recruited locally and trained. The data collection tools and strategies will be finalized.
The preparatory phase will require around 6 months.

Recruitment of the research teams and training
The research teams will be recruited locally from each block. Considering the difficult terrain and distance across the blocks, we propose to engage separate sets of teams for each block. Following model optimization in the first block the

implementation of the optimized model will be initiated concurrently in all the remaining 4 blocks. All team members for the 5 blocks will be recruited during the preparatory phase.
Training of the teams will be conducted during the preparatory phase. The IST and PLT of the remaining 5 blocks will receive hands-on training while the model is being optimized in the first block.

Overall implementation plan and timelines in the 5 blocks of Una district

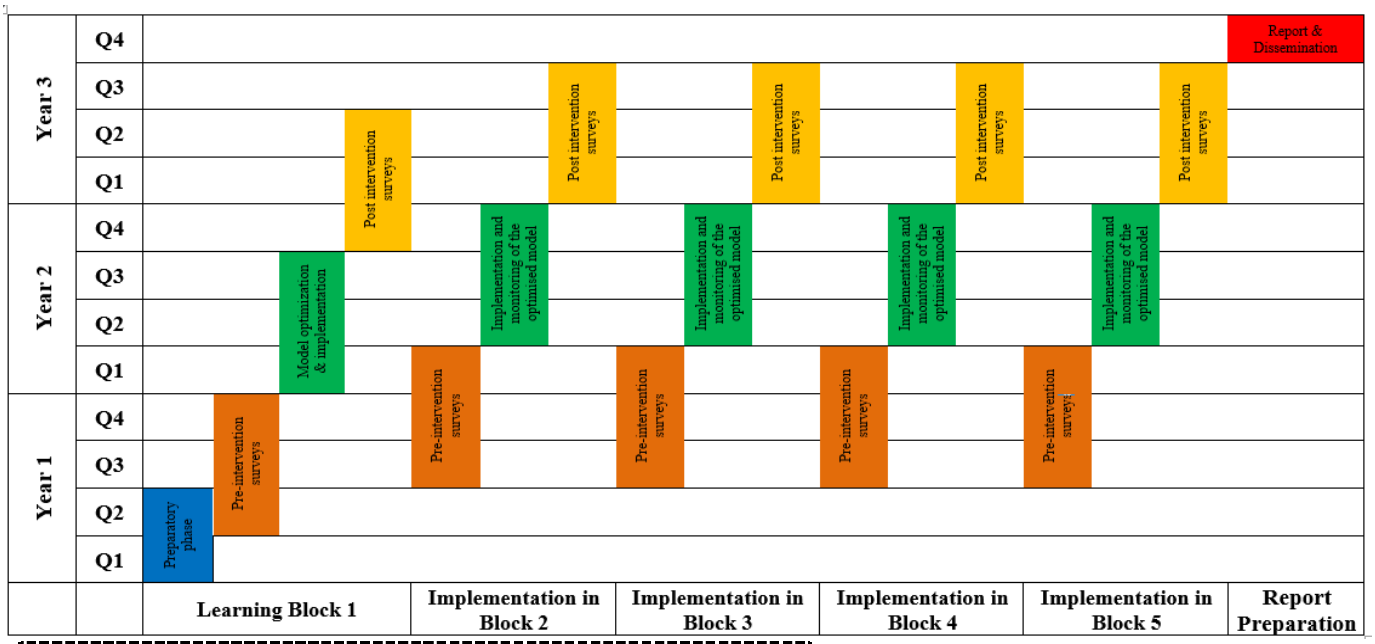

	Preparatory phase		Model optimization and implementation
	Pre intervention surveys		Post intervention surveys
	Report and dissemination		

Note: There will be 3 rounds of pre and post intervention survey

Formative research and baseline surveys

Formative research will be conducted by the PLT in the first block using mixed methods (qualitative and quantitative) to do a systems level diagnosis and identify the barriers and facilitators to implementation of the WINGS intervention.
The formative research methods will include facility assessment/survey and community-based activities (quantitative) and in-depth interviews, focus group discussions and observations (qualitative).
The following areas will be explored during the baseline survey and formative research.

- ***Population and population distribution*** in each of the 5 blocks in Una; married women in reproductive age group, eligible couples, pregnant women and under-two children, to obtain an idea about the potential beneficiaries.
- ***Information on pre-conception women:*** married women in reproductive age group with no or 1 child; number in each district; routine health and ICDS services they are entitled to in the state and service utilization including family planning services; the common sources for care seeking; points of contact of these women with the health and ICDS systems, and at facility and community levels.
- ***Key baseline health indicators*** of women in the reproductive age group including preconception women, pregnant and postnatal women and of children less than 2 years of age.
- ***Health and ICDS facilities in the area***, categories (CHC, PHC, HWC/SC, Anganwadi centers); number fully functional; infrastructure; human resources- sanctioned and vacant posts at each health facility, current workload of existing staff, average foot fall per month and private facilities in the area catering to maternal and child health.
- ***Available services in health facilities and community; service quality***:

- ANC and immunization
- Intra-partum services
- Postnatal care and child health services; home based postnatal care (HBPNC),
- Integrated Child Development Schemes (ICDS) supplementary nutrition- role of Self-Help Groups (SHGs); beneficiaries, description of food, availability, quality, amount, etc.
- WaSH and psychosocial services available in the study areas.
- Laboratory facilities and provision for the various investigations, any private laboratories empaneled under the JSSK scheme.
- Ultrasound facilities
- Equipment and supplies at the health facilities and anganwadi centers- functional anthropometry equipment, essential medicines, frequency of stock out and replenishment; procurement systems.
- Training and skill set of the personnel in health and ICDS, initial and periodic training they underwent; training content; any central pool of trainers, documentation of training quality assessment.
- Supervision and monitoring; periodicity of supervision, content.

- ***Referral facilities and availability of transport in the area*.** Referral facilities- type, distance from the study site. Transportation: government ambulances, public transport, buses, others.
- ***Current practices and utilization of services*** with reference to the 4 WINGS domains and across the periods of preconception, pregnancy and early childhood; common sources for ANC and coverage of various ANC indicators; delivery and postnatal care including lactation support and KMC, esp. for LBW/preterm babies; HBPNC visits; acceptability and utilization of the ICDS services; if low utilization of government health and ICDS services, the reasons for the same. Sources of drinking water, place of defecation; care seeking for psychosocial support; Care seeking for MCH services from the private sector, both informal and formal.
- ***Implementation status of national programs in maternal and child health*** that are relevant for WINGS- coverage, quality and utilization by beneficiaries and reasons for low utilization, if so.
- ***Use of digital media and mHealth*;** whether Auxiliary Nurse Midwife (ANMs) using Auxiliary Nurse Midwife Online (ANMOL) app; Closed Unit Group (CUG) in place; use of POSHAN tracker; MCTS/RCH and ICDS CAS whether operational.
- ***Health management information system (HMIS)*;** system of documentation, maintaining records; manual registers/electronic; system of periodic review of data; current practices of tracking preconception women (if any), pregnant women till delivery and postnatal period.
- Existing ***Social Behavioral Change Communication (SBCC) activities*,** commonly used government platforms (VHNDs, others- to create awareness, generate demand)
- ***Awareness and utilization of various government schemes:*** public distribution system (PDS), insurance schemes, PMJAY, JSY, JSSK etc.
- ***Any local non-government organizations*:** providing services in relevant intervention domains.

During the same period, the OMT will initiate the baseline surveys to collect information on the outcome indicators as defined above. We propose to conduct 3 rounds of surveys. This will be following the interrupted time series design.
The duration of the formative research and baseline surveys will take around 9 months.

Implementation model optimization in the first block.
CFIR framework will be followed for a comprehensive approach during the iterative cycles for model optimization. At the outset, the interventions in the government programs for pregnancy and childhood will guide the implementation strategies. Additionally, based on discussions with the government, the WINGS interventions which are not in the current programs for preconception women, pregnant women and children, will be included. This will constitute the 'Model 0'.
The formative research findings will be incorporated into Model 0 to develop Model 1. Following this, repeated rapid cycles of concurrent implementation, monitoring and refining strategies will be carried out. All iterations in the strategies will be done in multiple co-design workshops with deep government engagement. These iterative cycles will continue till optimized strategies are designed that have the potential of high and effective coverage.

Iterative cycles of model optimization.

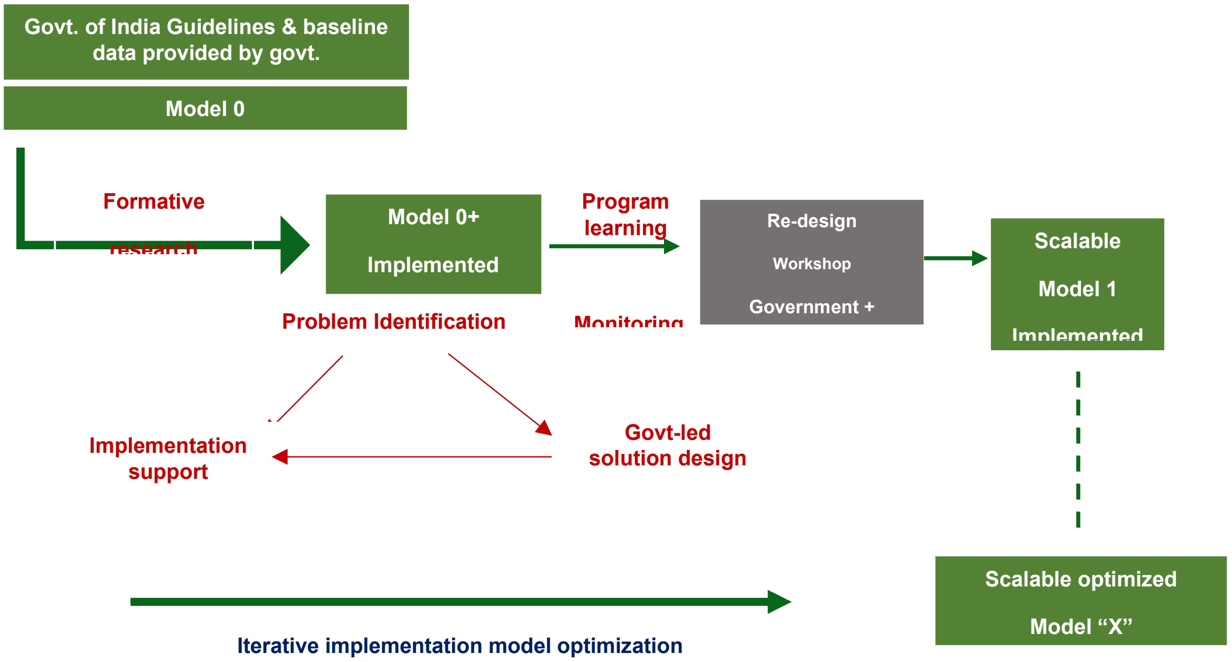

High coverage will be defined along with the government partners and will depend on the baseline coverage data. We consider a 20% increase in coverage of the indicators compared to the baseline coverage data. The target coverage may be differential for different outcomes and will be finalized after discussions with the government partners. Considering the complexity of the intervention package and delivery of these interventions across 4 domains, 20% improvement is reasonable. The project duration is limited. Longer duration is required to achieve a higher change in coverage. Additionally, the baseline coverage of the various indicators will be differential with wide ranges.

Since there are multiple domains in WINGS (health, nutrition, psychosocial and WaSH). It may be noted that the target coverage of each may not be achieved concurrently at the same time point. This may happen in a phased manner. Periodic review meetings will be conducted with the state government and NITI Aayog team. The trend in coverage and outcome indicators will be monitored and a decision will be taken to move on to the next block based on the positive gradient.

All government facilities as well as the community level workers in the block will be included in implementation. These will include the district hospital, Community Health Centers (CHC), Primary Health Centers (PHC), Health and Wellness Centers (HWC) and sub-centers (SC); the medical college hospital for referral services and the ICDS system with the AWCs.

The number of iterations or the models that will be required for each intervention will be subject to the active participation and inputs from the government. It is expected that each iteration would take about 2-3 months and about 3 such models would be needed to develop optimized strategies. A period of about 9 months may be required in the first block.

It is expected that in the subsequent blocks, the period will be less compared to the first block; approximately 6 months per block. The optimized implementation strategies may need contextual adaptations in the subsequent blocks, based on the needs, as these blocks may differ to some extent from the first block.

The outcome monitoring team will continue the 3 monthly surveys, 3 such surveys, after the implementation is in place, that is after 9 months of work in the first block.

The entire duration of the preparatory phase, model optimization and implementation phase, the pre-intervention and post intervention surveys, in all the blocks, preparation of the final report and dissemination are expected to be completed in about 36 months.

REAIM framework will be used to monitor the implementation strategies. While implementing the interventions we will measure the following

– What proportions of the service providers in the facilities and in the communities and pregnant women and their families, are aware of the interventions.

– Effect of interventions on outcome indicators
– Proportion and types of service providers and beneficiaries who are willing to engage with the interventions.
– Whether health care providers and beneficiaries are complying with the various components of the interventions
– Whether the processes and outcomes of the interventions are integrated into the routine operational practices and health management information system and reviewed periodically and independently by the government implementors. This will ensure accountability, ownership, and sustainability.

REAIM framework

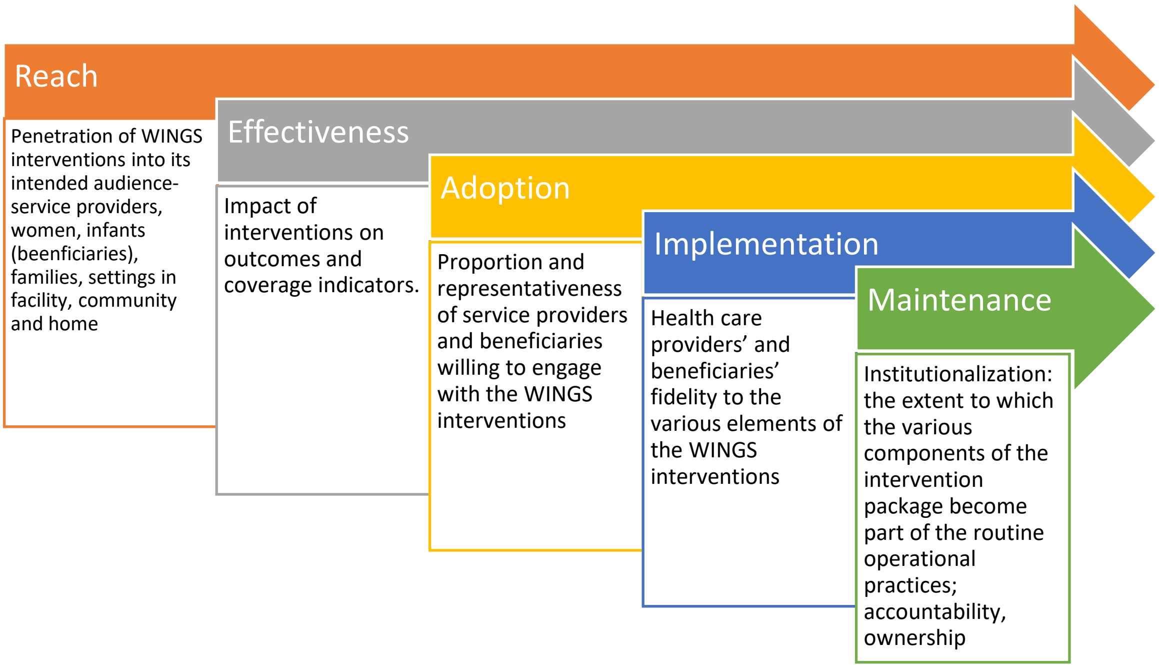

The COM-B model will be used while designing the implementation strategies, counselling messages, information, education and communication materials and activities around social behavioral change communications. The COM-B model focusses on the following:
Capability: Whether the providers and beneficiaries have the knowledge, skills and abilities to engage in a behaviour. For health care providers it would mean counselling, providing interventions and motivating women. For pregnant women it would include their comprehensibility, knowledge, attitude, mental state and physical strength to comply with the interventions.
Opportunity: Physical opportunity (financial and material resources) and social opportunity (culture and social norms). These will include availability (investigations, medicines, referral care), accessibility (geographical, social, cultural) and affordability (paid services if not available free of cost), in providing and accepting appropriate management of anemia.
Motivation: mental processes that direct behavior and influence decision making and behavior. This would involve instinctive, habitual processes- desires and habits. Explaining benefits will help women to turn a desired behavior from something they need to do, to something they want to do. Creating conviction among health care providers and beneficiaries would be important.
While implementing in the first block, the research team will share the barriers and gaps identified in the first block, with the government, so that the government can start filling in the gaps in the other blocks concurrently (e.g., vacant positions, ensuring supplies, functional equipment etc.). This will save time in the subsequent blocks.

Qualitative research
Qualitative research is an integral part of the model optimization process and an extension of the formative research conducted at the outset, for situation analyses and identification of barriers and facilitators. The Program Learning Team will conduct in-depth interviews (IDI), focus group discussions (FGD) and observations with all stakeholders including the beneficiaries, to obtain insights into the reasons why the interventions are working or not working, the barriers and enablers. They will interview the facility staff at all levels of government facilities that are involved in implementation of the WINGS interventions, the community health workers, the anganwadi workers and their supervisors, to understand the

challenges in implementation. The team will interview the women and their family members to explore the reasons for low utilization of services or non-compliance, if that is the case, or learn from those women with high compliance; they will probe into the community's expectations from the project and the government. The compiled feedback from this team will be communicated to the implementation support team on a regular basis.

Scaling up in the other 4 blocks

Activities in the remaining 4 blocks will be initiated concurrently. While the model is being implemented in the first block, the 3 rounds of baseline surveys and formative research will be conducted in the 4 blocks.

Following the baseline surveys and formative research in these blocks, the optimized model will be implemented in the **4** blocks concurrently. The model may need some contextual adaptations based on the requirement in each block. With government support and active participation, it is expected that the duration of this phase will be shorter compared to the first block. We expect the implementation to be in place in 6 months in each of these 4 blocks.

We will use the Interrupted time series design to monitor the study outcomes.

For each block, we will collect baseline data on the defined outcome indicators through 3 surveys. During this period, formative research will be conducted to identify the barriers and facilitators. These data will help develop the intervention strategies that will be implemented in the block. While the interventions are being rolled out using the improved implementation strategies, 3 monthly monitoring of the outcome indicators will be conducted. The time points for rolling out the various intervention implementation strategies may vary based on the government preparedness. Once the implementation strategies are in place, three rounds of post intervention surveys will continue for 9 months on the same lines of the baseline surveys.

We will have multiple data points (3 rounds of baseline surveys prior to intervention initiation, ongoing 3 monthly surveys during the implementation phase and 3 rounds of post-intervention implementation surveys) to ascertain the gradient and slope for the outcome and coverage indicators.

The processes and monitoring mechanisms will be integrated into the government systems for the government to be able to sustain these activities. Once a certain target coverage is achieved (will be finalized with the government and will be based on the baseline coverage), the research teams will exit from the respective blocks.

Scale up in the other districts of the state

Once the model is optimized in the first block, it will be implemented in the other 4 blocks of the district. Concurrent scale up in the other districts of the state or other states in the country that are interested to implement and scale up WINGS, is also feasible. SAS can play an advisory role if the government decides to concurrently scale up in the other districts.

Information on study participants

The study participants, i.e., the preconception women, pregnant women (in all trimesters of pregnancy) and infants (at different ages), will enter the intervention implementation phase at different time points as the interventions are rolled out in the blocks. Cross-sectional data on the participants will be available at each point of the three-monthly surveys conducted during the implementation phase in each block.

Information on the longitudinal follow up data on the participants, particularly the cohort of women from preconception till pregnancy and their infants in each block, will be collected by the government personnel, that will include the Accredited Social Activists (ASHAs), Anganwadi Workers (AWWs) and health facility staff. In this process the data collection system of the government personnel will be streamlined and strengthened. These data will be reviewed by the research teams along with the government.

Capacity building of the local government partners

While the interventions are being implemented, the government partners will learn from the processes, to be able to sustain the activities after the research teams exit. One or two local technical institutions will observe and learn from the implementation by the research teams, so that they can implement in the subsequent districts. The technical institutions will be identified by the government, taking into consideration their availability to take on this work.

DATA MANAGEMENT AND PLAN OF ANALYSIS

The periodic survey data will be collected by the outcome monitoring team, which will be a research team. The data management system for the survey data will be independent from the government data management system, that is, the

HMIS. The longitudinal follow up data of the cohort of the preconception women, through pregnancy and early childhood, will be collected by the government health workers and managed in the HMIS.

Therefore, the independent data management system will monitor the implementation progress through the ongoing 3 monthly surveys during the implementation phase. Additionally, 3 rounds of surveys will be conducted during the pre-intervention phase and the post-intervention phase. The data management system will be developed after reviewing the existing government system that is in place to ensure that we can compare data through both streams. We will work closely with the data management and the IT support team in the government and ensure that the core set of indicators are gradually integrated in the HMIS system over the duration of the implementation. The end target will be to be develop a system which can independently collect information on these indicators with good quality through the routine existing system.

Sample size estimation

We will aggregate data on the key outcomes of interest at block level and use the data collected in 5 blocks in the study district collected at 3 time points pre-intervention and 3 time points post intervention to assess the change in level of outcomes. The statistical power to detect a 20% change in the level of outcomes was estimated to be 88.3% (95%CI: 86.3 to 90.3) using "itspower" package in Stata 17.

The outcomes of interest in the study are as follows:

1. Proportion of pregnant women with adequate gestational weight gain defined as per IOM standards.
2. Proportion of pregnant women who are non anaemic i.e., Hb >= 11.0 mg/dl.
3. Proportion of pregnant women with normal BMI (>= 18.5 and <=25 kg/m2)
4. Proportion of children with adequate weight gain defined as per WHO standards.

Since we are aiming for 20% reduction compared to pre intervention status the following table shows the sample size estimates for different level of outcomes assuming that the outcome during pre-intervention phase will be as follows based on NFHS 5 data for Himachal and WINGS. This is for precisely estimating the level of outcomes in the target population during each cross-sectional round for pre and post intervention phases

Outcome	% pre-intervention	% post-intervention
Adequate GWG	55	66
Normal BMI	55	66
Non anemic	45	54
Adequate weight gain in children	60	72

A sample size of 600 women and children during each round would be the ideal and adequate. If this is not feasible a minimum of 400 women and children would be needed.

We will aggregate data on the key outcomes of interest at block level and use the data collected in 5 blocks in the study district collected at 3 time points pre-intervention and 3 time points post intervention to assess the change in level of outcomes. The statistical power to detect a 20% change compared with baseline in the level of outcomes was estimated to be 88.3% (95%CI: 86.3 to 90.3) using "*itspower*" package in Stata 17.

Plan of analysis

Quantitative data

The time series data will be analyzed using a segmented linear regression model in the R statistical environment. The data collected over nine months as part of the three rounds of pre and post intervention surveys will be cleaned before analysis. Summary statistics like mean and proportion will be estimated for pre and post intervention phase. Scatter plot of the time series will be used to identify underlying trends, seasonal patterns, and outliers. Bivariate comparisons between the outcome and potential time-varying confounders will be made.

Variable on the time elapsed (T) since the start of the study with the unit representing the frequency with which observations are taken (e.g., month or year) will be generated. A dummy variable (Xt) indicating the pre-intervention period (coded 0) or the post-intervention period (coded 1) and the outcome at time t (Yt) will be used to form the segmented regression model to estimate the magnitude of the intervention and test the statistical significance of immediate impact and trend rates:

$$Yt=\beta 0 + \beta 1T + \beta 2Xt + \beta 3TXt$$

Point estimate and 95% confidence interval will be generated using generalized linear model with Poisson family and log link for count outcomes, gaussian family with identity link for continuous outcomes, binomial family with logit link for binary outcomes. A p-value < 0.05 was considered statistically significant. The Moving Average (MA) method will used to smooth the data for the purpose of explaining temporal trends in case of outliers. Durbin-Watson test will be used to investigate the presence of autocorrelation, in addition to residual plots, Auto Correlation Function (ACF) plots, and partial-ACF plots.

Qualitative data

The information will be audio-recorded. Transcriptions will be prepared from the audio-records and field notes. N-vivo software package will be used to analyze the data that will be done simultaneously with data collection. Responses will be coded, these will be descriptive, based on the specific research question; analytical to understand the underlying meaning or concepts behind the responses, and theoretical which include cross-cutting constructs. The codes on the same theme will be grouped into themes and sub-themes. The findings will be weighted by identifying the key themes and estimating the number of times the theme appeared and the number of respondents who mentioned the theme. A framework analysis will be used that is, primarily a case and theme-based analysis. A matrix will display the information and enable us to examine information across rows which will help in maintaining the context, and down the columns which will help in theme development.

POTENTIAL RISKS OR CHALLENGES AND POSSIBLE MITIGATION STRATEGIES:

We have listed some of the anticipated challenges and possible mitigation strategies, we will have greater clarity on the barriers and unanticipated challenges after the formative research and through the ongoing program learning activities, during study implementation. While some of the challenges will need to be resolved at the outset in consultation with the government partners and TAG, the others will be done through the iterative cycles. The mitigation strategies, therefore, will be subject to availability of funds, resources including human resources, infrastructure, etc., and the scope of mobilizing additional resources. As we go along, many of the mitigation strategies will emerge and evolve.

Anticipated challenges and possible mitigation strategies

Potential risks or challenges:
Integrating WINGS interventions with the government health systems.
Integrating WINGS interventions with the government health systems: The additional interventions in WINGS, particularly those that are not in the national program, such as pre-conception screening and interventions, provision of snacks according to the preferences of women, multiple micronutrients in pregnancy, etc., will require concerted efforts from the government and political will. These interventions are currently not in the PIP for implementation in the near future.

Possible mitigation strategies:
Discussions will be conducted with the government partners, to reach a consensus on the implementation of the WINGS integrated interventions. The interventions that are already in the national programs may be strengthened and delivery strategies improved. A situation analysis will be required to obtain insights into functioning of the current health systems, the resources available, including human resources, material resources such as investigations, equipment and supplies at the various levels of the health system and identify the gaps. Accordingly, solutions can be worked out together with the partners. The feasibility of implementing the different interventions will be assessed, the proposed personnel responsible, training opportunities, procurement of supplies, will be carefully examined. The possibility of adapting any intervention or its delivery strategy, if at all, may be discussed with the TAG. The following are some suggestions for mitigation strategies; for example, rather than distributing water filters to each household, the government may take the decision to set up a water purification plant centrally with water connections to localities to improve access to clean drinking water; or construct “sulabh sauchalyaya” in case sanitary latrines are not available in every household; distribution of food for the target population through PDS/AWWs/HWCs; hot cooked meals are being served at the AW Centers for children, pregnant and lactation women; newly married women in reproductive age group or women with one or no children, may be included in the group of beneficiaries; the government may also decide to restrict observed feeding to the vulnerable women and children, that is, those with low BMI, anemia, inadequate weight gain, preterm and/or LBW babies (marked as red flag category), etc. However, any such adaptation will be reviewed to assess the extent to which the adaptation is acceptable without compromising with the integrity or diluting the interventions. The delivery strategies may need to be adapted in the government setting.
For those components that are not in the government program, the government will need to plan on implementing these and the actions needed, whether to incorporate into the state PIP, or immediate

operationalization is feasible with special sanction orders and budget availability. Additionally, in the domain of health, the implementation of Mission Parivar Vikas and Anemia Mukt Bharat (prevention and treatment of anemia and bi-annual deworming of non-pregnant women aged 19-24 years) need to be strengthened to cater to the preconception women. Overarching guidelines to promote menstrual hygiene, sanitation and hand washing, are already in place under the Adolescent Health Program and Swachh Bharat Mission.
Currently the ASHAs and ANMs maintain a list of all eligible couples in their designated village/area. This can serve as a systems level platform to deliver preconception interventions under the above national programs. The ICDS services will need to be extended to deliver the nutrition interventions for preconception women through close collaboration between the WCD and health departments. Other opportunities maybe explored with the government partners.
These will be discussed at length with the government partners during the preparatory phase and in the co-design workshops. Technical Advisory Group (TAG) with experts from NITI Aayog, ICMR and BIRAC will be involved. The possible options will be analyzed with deep government engagement and the way forward will be planned with the experts, government partners and research teams.

Potential risks or challenges:
Committed government leadership: Change in government leadership may lead to change in priorities and commitment.

Possible mitigation strategies
Signing an MoU will take care of risks associated with change in political leadership, to some extent.

Potential risks or challenges:
Funds to implement and sustain activities: Funds may not be available in the state project implementation plan (PIP). The other impediments may include the feasibility and timeliness to mobilize resources from other sources to fill in the gaps in HR, infrastructure, services, equipment and supplies and recurrent costs, that will be needed for sustained activities.

Possible mitigation strategies
Adequate funds is the cornerstone for implementation. Funds could be domestic/donor/innovative, that is, flexi pool (as mentioned in Ayushman Bharat), PPP etc. The government may need to explore mobilization of existing resources, unused or untied funds or seek funding from development partners. Resource reallocation may be discussed for optimum utilization of funds. The expected recurring costs will need to be included in the state Project Implementation Plan (PIP).

Potential risks or challenges:
Integrated interventions across 4 domains: Since the WINGS interventions cover 4 domains, multisectoral convergence will be essential. Feasibility of engaging all sectors with active participation may be an anticipated challenge.

Possible mitigation strategies:
The Government Implementation Unit (GIU) will be constituted. The government will designate personnel from the state and districts to support program implementation. The government will identify at least 2 nodal persons of contact who will be responsible for coordinating across all the departments and communicate with the other partners and the implementation research group. (Page 10).
The GIU will include nodal officers from department of health including mental health division of MOHFW (health and psychosocial support), ministry of Women and Child Development (WCD) (nutrition) and Ministry of Jal Shakti (WaSH). Their roles and responsibilities will be defined.
Additionally, the constitution of a Local Coordination Group (LCG) at grassroot implementation level will be needed to operationalize the day-to-day interactions and synergy across all concerned departments. The members of this Local Coordination Group may include representatives from the district administration, rural development, Panchayati Raj, SHFWT Center, and others.
The Poshan Abhiyan emphasizes Convergence as one of the key components. VHSND Day aims to ensure synergy across various departments and ministries, particularly nutrition related schemes of MWCD and various programs, through setting achievable targets, sector level meetings with concerned Secretaries. POSHAN tracker (common application software) will enable software based tracking of nutritional status. These activities may be leveraged.
These issues will be discussed during the preparatory phase.

We will additionally discuss constituting a Review Committee for Policy Oversight with leadership from NITI Aayog and Chief Secretary level. This will serve as another layer of mitigation strategy in case even one of the departments is unable to participate in the convergence.
**Potential risks or challenges:**<br>**Establishment of electronic tracking systems within the government**: The formative research will provide insights into the current status of the Heath Management Information System (HMIS), the completeness and processes of tracking and longitudinal follow up of each woman and child, whether electronic or based on paper forms, etc. Strong IT support will be needed to strengthen the systems.
**Possible mitigation strategies:**<br>Strong IT support will be needed to strengthen data driven action. Electronic trackers already exist, such as the MCTS in RCH, ICDA CAS, Poshan tracker, etc. A desk review of the HMIS system will be required for identifying the current status of HMIS, status of the trackers, operationalizing the trackers and ascertaining the modifications needed to incorporate the WINGS indicators. Additionally, the strategies to make the Mother and Child Tracking System (MCTS/RCH) and POSHAN trakcer interoperable, to remove overlap and increase efficiency, will be explored. This will be done during the formative research and will throw light on the existing status of the HMIS, the ANMOL app or the ABHA ID, whether these are functional, need strengthening and to what extent. Strengthening the HMIS is possibly beyond the scope of the project, therefore specialized IT agency and local IT support will be needed. Since the implementation will be done in the program setting, it is not feasible to consider the entire comprehensive list of intermediate and final outcomes that were tracked in WINGS. The core set of indictors need to be integrated into the routine HMIS; an electronic tracking system needs to be developed within the government database for efficient longitudinal follow-up, all these activities will require strong IT support
**Potential risks or challenges:**<br>**Accountability**: Establishment of systems for supervision, monitoring and quality assurance within the government, that are sustainable, may be a challenge due to workload of the supervisory cadre.
**Possible mitigation strategies:**<br>A situation analysis will be done to ascertain the current status of HR, that is, posts sanctioned and vacant. The government will need to recruit staff to fill in the vacancies. The Implementation Support Team will guide the government to define the roles and responsibilities of each cadre of government personnel, develop quality control and supervisory checklists and formats for documenting and reporting activities.
**Potential risks or challenges:**<br>**Institutionalization**: This may be another challenge that we anticipate. Institutionalization needs to be done through incorporation into the HMIS for periodic review of defined process and outcome indicators by the government partners. The extent to which the integration will be feasible will be subject to the current status of capturing the information by the Himachal government in the routine HMIS.
**Possible mitigation strategies:**<br>The extent to which the integration will be feasible will be subject to the current status of capturing the information by the Himachal government in the routine HMIS. The IT support team will facilitate streamlining the processes.

Sustainability and Exit

- The WINGS indicators will need to be integrated with the routine reporting systems and the HMIS data base, as reportable items. The proceedings of the monthly review meetings by the district authorities with all government stakeholders will be observed by the Implementation Support Team while the research activities are ongoing. Later, the Government Implementation Unit and the Local Coordination Group will take on the responsibility of overseeing the implementation at the district level till these activities become an integral part of the health systems. Similar units will need to be established at the state level. The senior district authorities will be provided with the checklist and reporting format in which the WINGS additional indicators will be incorporated. These periodic reviews by the district authorities will increase accountability and ownership.
- Additionally, establishing a technical support unit may be considered, comprising representatives from the departments of Community Medicine, OBG, Pediatrics, of the local medical college hospital, local IT team, ICMR Multidisciplinary Research Units (MRU) under the department of Health Research and others that the

government and TAG deem as relevant. This unit can continue to provide sustained technical support for program implementation.

- The implementation support team that handholds the government, will gradually withdraw support and take up advisory role (if required) till the government is able to implement all interventions, monitor and evaluate independently.
- Institutionalization is the cornerstone for sustainability. The IST will completely withdraw once the institutionalization process is strengthened with complete ownership and accountability by the government.
- Having a cadre within the government system that can be responsible for program learning or process evaluation activities, will be helpful to identify the reasons for poor implementation or low fidelity and adherence. The continuous feedback will facilitate prompt corrective actions. This is a recommendation for sustainability, we are not sure whether it will be feasible for the government to create a new cadre of workers within the existing system. This point maybe discussed in one of the review meetings, on whether any of the existing cadre can take on this additional responsibility, if at all feasible.
- Most of the WINGS interventions are already in the national guidelines. Strengthening of implementation is needed. There may be initial barriers. However, once the processes are demonstrated and adopted by the government, these become an integral component within the systems. Documentation of the processes, developing standard operating procedures, manual of operations, checklists and guides, job-aids, FAQs, well defined roles and responsibilities, etc. will facilitate sustainability.

DISSEMINATION

The project findings will be disseminated at district, state and national levels through workshops. In addition, the learnings will be shared at various platforms like national and international conferences seminars.
Additionally, study findings will be disseminated through presentations and publications at scientific meetings and in peer reviewed journals respectively.

3. Organizational capacity - (Technical, Infrastructure and Administrative capacities):

Provide a summary of the lead applicant's and organizations previous experience and expertise that is relevant, including a discussion of the partner organization(s).

CONVERGENCE BETWEEN LINE DEPARTMENTS AND OTHER DEVELOPMENT PARTNERS

Line departments and development partners will collaborate to implement a comprehensive health and nutrition intervention package. This integrated approach will cover the pre-conception period, pregnancy, and the first 24 months of a child's life. The aim is to maximize the combined efforts of government agencies and external partners to achieve better health and nutrition outcomes for mothers and children.

At various administrative levels viz State, district, and block, existing platforms will converge with development partners.

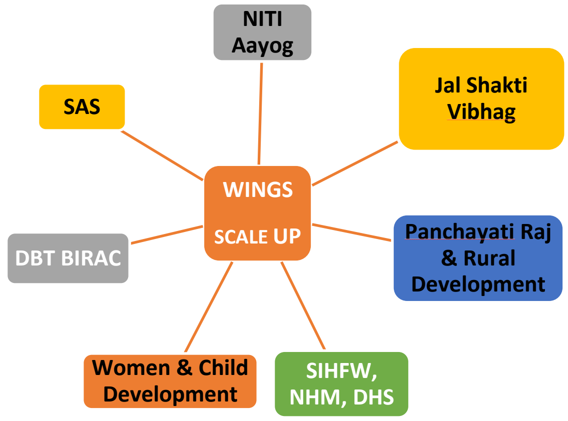

Support Units for effective implementation of WINGS

To enhance sectorial programme coordination in health and nutrition, Support Units will be created at State, district, and block as under:

A. State Level

a) Technical Support Unit:

The Technical Support Unit will consist of the following:

**Secretary, Health**	**Chairperson**
**Secretary, SJE**	**Co-chair**
Mission Director	Member
Director Women and Child Development	Member
Deputy Mission Director	Member
Representative of IGMC & AIIMS (Bilaspur)/ HOD (Gynecology, Pediatrics) / C.D Psychology	Member
Principal, State Health & Family Welfare(SIHFW) Parimahal, Shimla	Member
Representative of WHO	Member
Representative of BIRAC	Member
Representative of BMGF	Member
Representative of SAS	Member
Representative of H.P Voluntary Health Association	Member
Director Health Services	**Member Secretary**

Roles & Responsibilities:

1. The Technical Support Unit will meet Biannually.
2. Nodal technical resource centre for all matters pertaining to nutrition in the State.
3. Establish systems for monitoring .
4. Advocacy across various stakeholders.
5. Facilitate knowledge sharing, including documentation and dissemination of best practices.

b) State Implementation Unit

The State Implementation Unit will consist of the following:

**Secretary, Health**	**Chairperson**
Secretary, SJE	Co-chair
Mission Director	Member
Deputy Commissioner of Una	Member
Director Women and Child Development	Member
Engineer in Chief, Jal Shakhti Vibhag	Member
Director, Medical, Education & Research	Member
Deputy Mission Director	Member
Head of Department, Department of Psychology, HPU	Member
Director Health Services	Member Secretary
Representative from SAS	Member
Other staff: Project Manager , Accountant, Secretarial Assistant/DEO	

Roles &Responsibilities:

1. The State Technical Unit will meet Quarterly.
2. To coordinate and evaluate Key Performance Indicators for health and nutrition of the targeted beneficiaries.
3. Overall administration and coordination of the WINGS.
4. To implement the course corrections suggested by the Technical support unit.

B. District Implementation Unit

The District Implementation Unit will consist of the following:

**Deputy Commissioner**	**Chairperson**
Superintendent Engineer (IPH)	Member
Deputy Director, Food, Supplies & Consumer Affairs	Member
District Program Officer	Member
Block Medical Officer	Member
Child Development Project Officer	Member
Chief Medical Officer	Member Secretary
Representative from SAS	Member
Other staff: Nutrition & Health expert, Project assistant, Data entry operators	

Roles & Responsibilities:

1. The District Technical Unit will meet Monthly
2. Need assessment of essential interventions at the District level i.e. water, sanitation, food, health interventions, immunization, ANC/PNC, Vitamin-A, IFA, Deworming tablets, functioning of VHSNC, etc. and the availability of resources.
3. Indicate the roll-out plan, assign responsibilities of each department to avoid any over-lapping or consequent shifting of responsibility.

C. Block Implementation Support Unit

The Block Implementation Unit will consist of the following:

**Sub Divisional Magistrate**	**Chairperson**
Block Development Officer	Member
Block Medical Officer	Member
Child Development Project Officer	Member Secretary
Representative from SAS	Member
Other staff: Project assistant, Data entry operators	

Roles & Responsibilities:

1. The Block Implementation unit will meet twice in a month.
2. Need assessment of essential interventions at the Village level i.e. water, sanitation, food, health interventions, immunization, ANC/PNC, Vitamin-A, IFA, Deworming tablets, functioning of VHSNC, etc. and the availability of resources.
3. Indicate the roll-out plan, assign responsibilities to concerned department at Block level.

Technical Advisory Group (TAG)

A technical advisory group (TAG) will be constituted by GCI BIRAC with experts from NITI Aayog, ICMR, BIRAC and other eminent experts. The TAG will review the implementation progress through periodic review meetings with the HP government and the research teams.

SAS strengths: SAS specializes in community-based research, assessment and action, and product evaluation. The organization comprises of outstanding professionals and investigators dedicated to the promotion of maternal and child health. These investigators have been working for several years in the areas of childhood vaccines, child health and nutrition, development of interventions, their evaluation and development of strategies for delivery of the programs. SAS has systems in place for identifying, recruiting and training personnel for conduction of community-based field trials.
SAS has a Scientific and Program Advisory Committee that guides the group to identify important research questions, interventions, or technologies and then implement the research findings through partnership, networking with public and non-government institutions.

SAS was awarded recognition as Scientific and Industrial Research Organization (SIRO) by the Government of India in the Year 1999. SAS is a WHO Collaborating Centre for Research, Community-based Action and Programme Development in Child Health. It is also the Population Science Partnership Centre of the Translational Health Science and Technology Institute (THSTI), Faridabad, Haryana.

Our group has world class research experience and skills in design, analysis and follow up of cohorts and trials for intervention efficacy and effectiveness in populations. The number of trial participants has ranged from a few hundreds to around 100,000 children with outcomes being monitored for several years. Our research findings have been published in reputed international journals and incorporated into national programs in India and the Maternal and Child Health guidelines of the World Health Organization. Our research was central to many child health policies and programs such as the WHO Multicentre Growth Reference Study which led to the development of the current growth standards for the world's children, exclusive breastfeeding for 6 months rather than 4 months, zinc for the treatment and prevention of diarrhea and pneumonia, the indigenously developed rotavirus vaccine which is now part of the Indian immunization program, the effect of vitamin A supplementation on neonatal mortality, ready-to-use therapeutic foods for home management of severe malnutrition, and the effectiveness of the Integrated Management of Neonatal and Childhood Illnesses (IMNCI) program. Much of this research was multi-country and also multisite within India.

SAS was the group from India that participated in the WHO Multicentre Growth Reference Study (MGRS) of the World Health Organization. This has provided them with unique insight into assembly and follow up of mother-child cohorts in the type of population where childhood obesity and metabolic abnormalities are a public health problem. The cohort for the MGRS was assembled by screening 58 mid- to high- socioeconomic neighbourhoods in South Delhi (population ~250,000 spread over 250 sq kms). The Society for Applied Studies is currently undertaking an intervention trial which is very similar in design to the proposed research. The primary objective of this trial is to reduce childhood stunting at 2 years of age. In this community-based individually-randomised, factorial design study ~8500 women in the reproductive age group will be enrolled and randomized to either the periconceptional intervention or to standard care. Those who become pregnant over the next two years will then be randomized to pregnancy and postnatal interventions or to standard care. The interventions include medical care, nutrition support, water, sanitation hygiene (WASH) and social support.

SAS has three facilities dedicated for research and development activities – a Central Office in Kalu Sarai, New Delhi, a Clinical Science Centre in Devli, New Delhi and the Research Centre in Palwal, Haryana. In addition, it also maintains R&D support infrastructure in Districts Sirmaur in Himachal Pradesh and Sonepat in Haryana.

Research Coordination and Management Support Centre in Kalu Sarai, New Delhi: SAS, Delhi owns 9000 sq ft of constructed space spread over 5 floors in a centrally located area in Delhi. The facility has the following divisions:

- Big Data Facility for modelling epidemiological and biochemical data from cohorts in intervention trials in collaboration with World Health Organization and Biotechnology Industry Assistance Research Council of the Department of Biotechnology, Government of India.
- A young investigator training centre under an inter-institutional platform for grooming physician young investigators in human studies.
- Offices for several principal investigators and administrative, human resources and finance support team.
- The space houses the following divisions: a section for the study coordinators and their support staff for each ongoing trial, a secretarial division, an accounts division, and a floor for the director, visiting scientists and conference room. One whole floor is dedicated for the data management activities.

Clinical Science Centre in Devli, New Delhi: The centre is self-contained area of ~10,000 sq ft and provides facilities for screening, enrollment, randomization, assessment, collection and storage of biological specimens, patient monitoring and outpatient care for evaluation of vaccines and diagnostics and other health care technologies for the academia and industry. The centre has systems for audiovisual recording of consent procedure and emergency care as mandated by the Drugs Controller General of India. Human data can be archived on a long term basis as the biological specimens for laboratory analyses for human studies are collected from the large neighbouring populations including Sangam Vihar and Dakshinpuri. We have teams in place for conducting different study related activities.

Population Science Research Centre in Palwal, Haryana: SAS has a Research Centre (5500 sq ft covered area) in Tatarpur, District Palwal. This centre is the base for all population science studies and major intervention trials including nutritional interventions conducted by the group.

Data Management and Analysis Centre, New Delhi: SAS has established a big data management centre for storage and analysis of data from cohort and intervention studies in humans done by SAS or through multicentre and multicountry trials. These studies provide clinical, epidemiological and biological/biochemical data. This data is also used for modeling to gain

insights into pathways through which problems such as stunting, poor immune response to oral vaccines, severity of infection, cognitive development can be elucidated. The centre also supports the Tuberculosis Cohorts through the national network coordinated by NIH/ICMR and DBT. The centre also works in collaboration with the World Health Organization, Geneva. Since data from regulatory trials is also managed at the centre, specialized systems for security (restricted access), fire safety (fire resistant cabinets, smoke detectors, automatic fire alarms, fire extinguishers and water sprinklers), unauthorized data control (restricted access server rooms, antivirus and spyware, Windows Firewall) are available as per global standards.

Clinical and Research Laboratories: An in-house laboratory 'Clinical and Research Laboratories' is located in South Delhi field office of SAS. The laboratory supports investigations for the medical management of participants enrolled in studies conducted by our organization as well as for outcomes related to research. The layout and workflow has been planned and designed in accordance with the guidelines of the National **Accreditation** Board for Testing and Calibration Laboratories. It is equipped for specimen processing for serum/plasma separation, storage of specimens in -20°C deep freezers, continuous monitoring through Temperature Monitoring Systems with SMS and call alerts to designated persons for temperature deviations. The laboratory is equipped to perform basic hematology assays (hemoglobin, red and white blood cell count, platelet counts, etc.) and blood biochemistry assays (sugar, lipid profile, serum electrolytes, kidney function test, liver function test) and urine and stool tests (routine & microscopy). Facilities for conducting ELISA based assays, micronutrient assays and other assays have been established based on the requirement of future projects that will be done by the organization.

The laboratory equipment includes Hematology Automated Analyser (Sysmex XN100), Biochemistry Automated Analyzer (Cobas C111), Bactec FX40 Automated Modular Blood Culture System, EVOLIS Twin Plus Fully Automated ELISA Processor, Creamatocrit Plus Centrifuge, Millipore Water Purification System, Elix Essential Water Purification System - 30 litre reservoir, Super Pro Dual Filter Prefiltration System, Cooling Centrifuge, Autoclave, Incubator, Microscope, -20°C and -80°C Deep Freezers, Refrigerators, Biosafety Cabinet and Sartirus Analytical Weighing Balance.

Storage and Archival Facility: Essential documents related to each study, consent forms and other documents need to be retained with the investigators according to the sponsors specification and regulatory guideline. SAS has two storage and archival facilities (spread over 1800 sq ft and 2250 sq ft) in south Delhi where all study related documents are stored. The offsite data management back up is also kept in this facility. Essential documents are stored in fire resistant cupboards.

Vaccine Storage Facility: A limited access, fully air conditioned vaccine storage facility has been set up in the field office. Additional facilities with all prerequisite requirements are in place in south Delhi and Faridabad. The vaccines are stored in refrigerators or deep freezers according to the storage requirements. Calibrated continuous temperature monitoring systems are installed in each facility to maintain and record the temperature in the desired range. The temperature status is transmitted daily through SMS to the designated persons and alerts through calls are also received if the temperature goes out of the desired range. Power back up systems is in place at each of these facilities. This facility supports vaccine research both for the government agencies (Department of Biotechnology) and public-private partnership projects supported by Biotechnology Industry Research Assistance Council of the Department of Biotechnology

Capacities Relevant for the Proposed Study

SAS has expertise in conducting implementation research. We were one of the seven sites from India and Ethiopia for the WHO supported implementation research study that used a mixed-methods design to develop an adaptable strategy to improve KMC implementation. In this study, KMC was scaled up with high quality and population-based coverage in an entire district with population of 1.5 million. The KMC wards were established in 3 hospitals in Himachal Pradesh, Dr. Rajendra Prasad Govt. Medical College & Hospital, Kangra, Zila hospital in Dharamshala and Kamla Nehru Hospital in Shimla. The implementation research was conducted with deep government engagement. SAS has almost 3 decades experience of working in collaboration with the government of Haryana with remarkable achievements. This includes working at primary, secondary and tertiary levels including medical college hospitals in all the districts of Haryana, community mobilization through multi-stakeholder involvement. Our symbiotic relationship with the government has led to capacity building of government personnel and setting up of Special Newborn Care Units and Kangaroo mother care wards in government hospitals, thus providing the much-needed impetus to newborn care.

In the possible serious bacterial infections (PSBI) implementation research, Himachal Pradesh government implemented the guidelines in program settings supported by SAS. The strategy included community sensitization, skill enhancement of ASHAs, ANMs and Medical Officers, to identify (PSBI) and treat when referral was not feasible. This implementation research concluded that Implementation of the guidelines in program settings is possible and acceptable. Scaling up would require

creating community awareness, early identification and appropriate care seeking, strengthening ASHA home-visitation program, building skills and confidence of MOs and ANMs, uninterrupted supplies and a dependable referral system.

To enable the WINGS scale up, the SAS teams specialising in each of the domains of interventions, included in WINGS will be available to provide technical inputs and support to the government to effectively scale up the interventions.
The technical advisory group (TAG) will guide the implementation progress and provide valuable inputs.
The Himachal Government is committed to support implementation and scale up.

4. IP Status:

Not applicable
